# Palladium/XuPhos-catalyzed enantioselective cascade Heck/intermolecular C(sp^2^)–H alkylation reaction†

**DOI:** 10.1039/d4sc00262h

**Published:** 2024-03-09

**Authors:** Chao Fang, Quan-Pu Wang, Bing Xu, Zhan-Ming Zhang, Junliang Zhang

**Affiliations:** a Department of Chemistry, Fudan University Shanghai 200438 P. R. China Zhanmingzhang@fudan.edu.cn junliangzhang@fudan.edu.cn; b Fudan Zhangjiang Institute Shanghai 201203 P. R. China; c Zhuhai Fudan Innovation Institute Zhuhai Guangdong 519000 P. R. China; d School of Chemistry and Chemical Engineering, Henan Normal University Xinxiang Henan 453007 P. R. China

## Abstract

Palladium-catalyzed enantioselective domino Heck/intramolecular C–H functionalization reaction, as a valuable strategy for creating molecular diversity, has remained a prominent challenge. Here, we describe a Pd/XuPhos catalyst for asymmetric domino Heck/intermolecular C–H alkylation of unactivated alkenes with diverse polyfluoro- and heteroarenes in a highly chemo- and enantioselective manner. This process enables efficient synthesis of various dihydrobenzofurans, indolines and indanes, which are of interest in pharmaceutical research and other areas. Late-stage modifications of the core structures of natural products are also well showcased. Moreover, synthetic transformations create a valuable platform for preparing a series of functionalized molecules. Several control experiments for mechanistic study are conducted to pursue a further understanding of the reaction.

## Abstract 2

An asymmetric domino Heck/intermolecular C–H alkylation of unactivated alkenes with diverse polyfluoro- and heteroarenes in a highly chemo- and enantioselective manner was demonstrated, with the use of a Pd/XuPhos catalyst.

## Graphical abstract

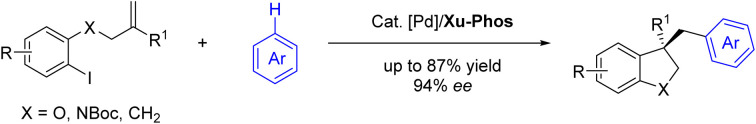

## Introduction

**Scheme 1:**
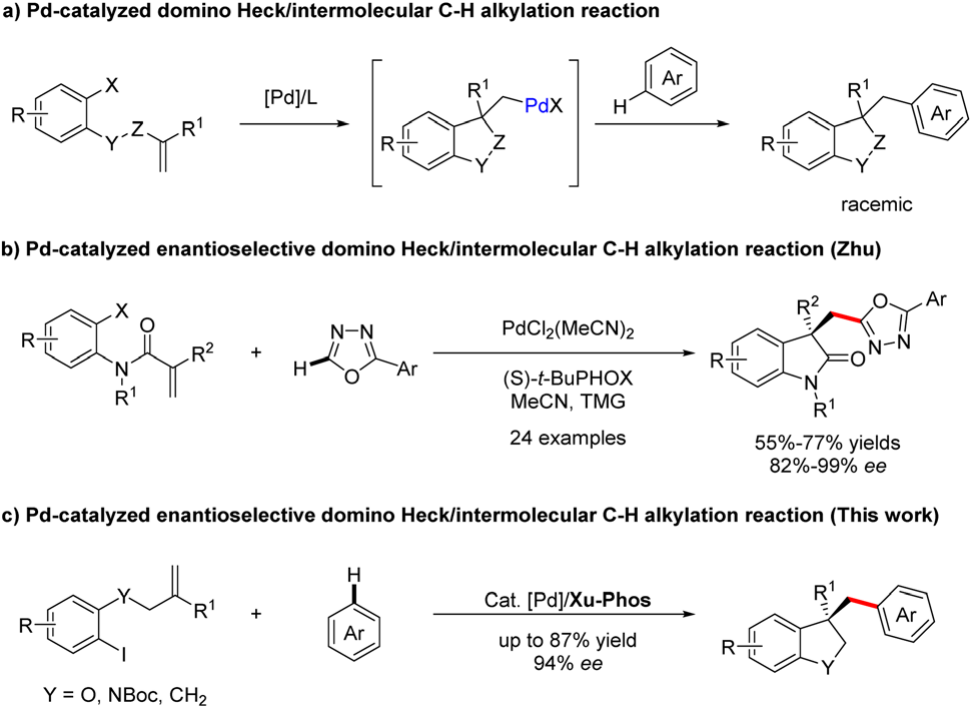
Previous work and this work on Pd-catalyzed domino Heck/intermolecular C–H alkylation reaction.

Palladium-catalyzed C–H bond functionalization, as a synthetically significant yet challenging bond-forming process, has been tremendously exploited to realize precision control of site-selectivity for fabricating densely functionalized molecules.^1,2^ Among others, palladium-catalyzed domino Heck/C–H functionalization reaction involving an σ-alkylpalladium intermediate represents one of the most powerful, step- and atom-economic tools to construct highly functionalized heterocyclics bearing quaternary carbon centers.^3–9^ Compared with Heck/intramolecular C–H functionalization,^3–9^ the intermolecular reactions are more challenging owing to the direct C–H functionalization side reactions. In 2009, the group of Fagnou reported a pioneering study on palladium-catalyzed domino Heck/intermolecular C–H alkylation reactions between aryl bromides with sulfur-containing heterocycles.^10^ Utilizing a similar strategy, Sharma and Van der Eycken demonstrated that acrylamides could react with 1,3,4-oxadiazoles to construct bis-heteroaryl frameworks under microwave irradiation.^11,12^ Later, the domino process was applied to the synthesis of alkylated polyfluoroarene derivatives employing electron-deficient polyfluoroarenes as the direct arylation coupling partner, which was accomplish by Liang and Xu.^13^ Recently, Kuram *et al.* disclosed that 1,2,3-triazoles were also suitable coupling partners to obtain bisheterocycles bearing all-carbon quaternary centers.^14^ Despite continuous development in the Heck/C–H alkylation reaction (Scheme 1a), the exploration of its asymmetric variants is still dramatically limited. To the best of our knowledge, enantioselective domino Heck/intermolecular C–H bond functionalization was only established by Zhu and co-workers, efficiently creating various 3,3-disubstituted oxindoles and bisoxindoles (Scheme 1b).^15^ Thus, the identification of new catalysts for this interesting reaction is still highly in demand.

In 2022, a remarkable example of highly enantioselective domino Heck/intramolecular C–H alkylation for the selective synthesis of chiral strained 5,4- and 5,5-spirocycles was accomplished by our group, employing our own developed Sadphos as the chiral ligand.^8^ Based on the success of the intramolecular variant and our ongoing interest on domino Heck reactions,^8,16–19^ we were intrigued to develop newly efficient catalyst systems to realize domino Heck/intermolecular C–H alkylation, which, if successful, would offer a highly efficient route for the construction of various privileged heterocycle skeletons existing in a number of natural products and drugs.^20–23^

Herein, we establish a Pd/XuPhos system as an effective catalyst for the enantioselective cascade Heck/intermolecular C(sp^2^)–H alkylation reaction of unactivated alkenes with various polyfluoroarenes, providing expedient access to a wide spectrum of structurally diverse dihydrobenzofuran-, indoline- and indane-containing polyfluoroarene compounds (Scheme 1c). Moreover, the significance of this methodology is also underscored by easily converting products to other classes of functionalized molecules.

## Results and discussion

**Scheme 2:**
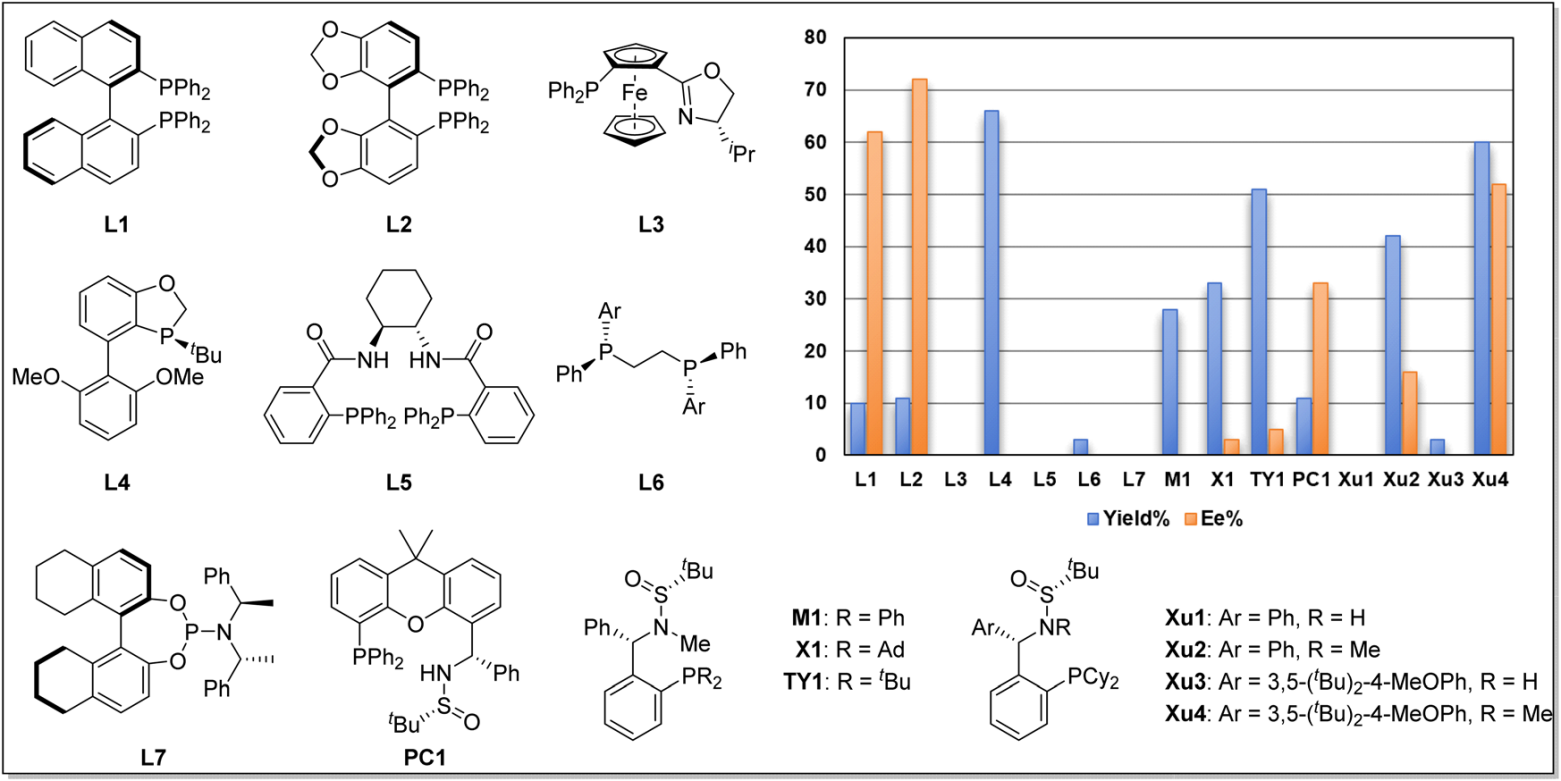
Representative diphosphorus ligands examined in this work.

We began our investigation using *o*-iodophenol-derived allyl ether 1a and pentafluorobenzene 2a as model substrates (Table 1 and Scheme 2). An exhaustive screening of various types of monodentate and bidentate commercial ligands showed that ligands L3 and L5–7 failed to deliver the desired product 3a (Scheme 2). Although ligand L1–2 showed better enantioselectivity and ligand L4 favored this transformation, both of them didn't obtain 3a with satisfactory results. Then, we turned attention to our developed ligands, which have demonstrated potential performances in palladium-catalyzed asymmetric cascade Heck reactions. The examination of the Sadphos ligand kit indicated that only *N*-Me masked ligands could deliver the desired product, in which Xu4 was the optimal choice, allowing the formation of 3a in 60% yield with 52% ee. Further screening of different solvents indicated that Et_2_O, ^i^Pr_2_O and MTBE resulted in higher ee (Table 1, entries 1–3). The use of DCM, DMF, CH_3_CN and DCE as solvent increased neither yield nor ee (entries 4–7). Subsequently, we focused on the optimization of the metal salt and base (entries 8–16). When the metal salt and base were changed to Pd_2_dba_3_·CHCl_3_ and Cs_2_CO_3_, respectively, the desired product 3a was obtained in 83% yield with 90% ee (entry 16). To our delight, lowering the temperature to 80 °C provided 3a with a slightly higher ee of 92% (entry 17). Finally, it was found that Ag_2_CO_3_ had also a considerable effect on the reactivity (entry 18).

**Table 1:** Optimization of reaction conditions^a^

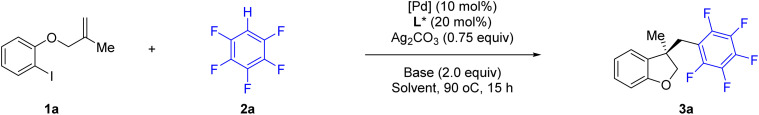					
Entry	[Pd]	Solvent	Base	Yield^b^ (%)	Ee^c^ (%)
1	Pd_2_dba_3_	Et_2_O	K_2_CO_3_	66	76
2	Pd_2_dba_3_	^i^Pr_2_O	K_2_CO_3_	80	84
3	Pd_2_dba_3_	MTBE	K_2_CO_3_	74	80
4	Pd_2_dba_3_	DCM	K_2_CO_3_	44	28
5	Pd_2_dba_3_	DMF	K_2_CO_3_	10	5
6	Pd_2_dba_3_	CH_3_CN	K_2_CO_3_	45	49
7	Pd_2_dba_3_	DCE	K_2_CO_3_	48	35
8	Pd_2_dba_3_·CHCl_3_	^i^Pr_2_O	K_2_CO_3_	82	88
9	Pd(η-allyl)Cl_2_	^i^Pr_2_O	K_2_CO_3_	78	75
10	Pd(OAc)_2_	^i^Pr_2_O	K_2_CO_3_	24	25
11	Pd(TFA)_2_	^i^Pr_2_O	K_2_CO_3_	60	32
12	Pd(acac)_2_	^i^Pr_2_O	K_2_CO_3_	15	3
13	Pd_2_dba_3_·CHCl_3_	^i^Pr_2_O	KOH	70	80
14	Pd_2_dba_3_·CHCl_3_	^i^Pr_2_O	KO^*t*^Bu	74	63
15	Pd_2_dba_3_·CHCl_3_	^i^Pr_2_O	CsOPiv	46	7
16	Pd_2_dba_3_·CHCl_3_	^i^Pr_2_O	Cs_2_CO_3_	83	90
17^d^	Pd_2_dba_3_·CHCl_3_	^i^Pr_2_O	Cs_2_CO_3_	84	92
18^d^^,^^e^	Pd_2_dba_3_·CHCl_3_	^i^Pr_2_O	Cs_2_CO_3_	Trace	—

a Unless otherwise noted, all reactions were performed with 1a (0.1 mmol), 2a (0.3 mmol), Ag_2_CO_3_ (0.075 mmol), base (0.2 mmol), 10 mol% [Pd] and 20 mol% ligand in 1.0 mL solvent at 90 °C for 15–48 h.

b NMR yield with CH_2_Br_2_ as an internal standard.

c Enantioselectivity was determined by chiral-phase HPLC.

d 80 °C.

e No Ag_2_CO_3_ added.

Having established the optimized conditions, the scope of this reaction was examined by using various *o*-iodophenol-derived allyl ethers. Different linear and branched alkyl groups on the alkene moiety proceeded smoothly to furnish 3b–3f in good yields with excellent enantioselectivities. Numerous allyl ethers bearing functional groups, such as trimethylsilyl (1g), methoxycarbonyl (1h), chloro (1i and 1j) and fluoro (1k), were compatible with the reaction to form the corresponding products in satisfactory results. To our delight, substrates with various ether, thiol ether and N-heterocycles appended to the alkyl chain were suitable for the reaction to deliver the expected products 3l–3r with 90–93% ee. Particularly noteworthy was the tolerance of the reaction conditions to the more structurally complex contexts. A variety of allyl ethers derived from the core structures of natural products were also suitable substrates, converting to the target products (3s–3u) in excellent yields with outstanding diastereoselectivities.

**Scheme 3:**
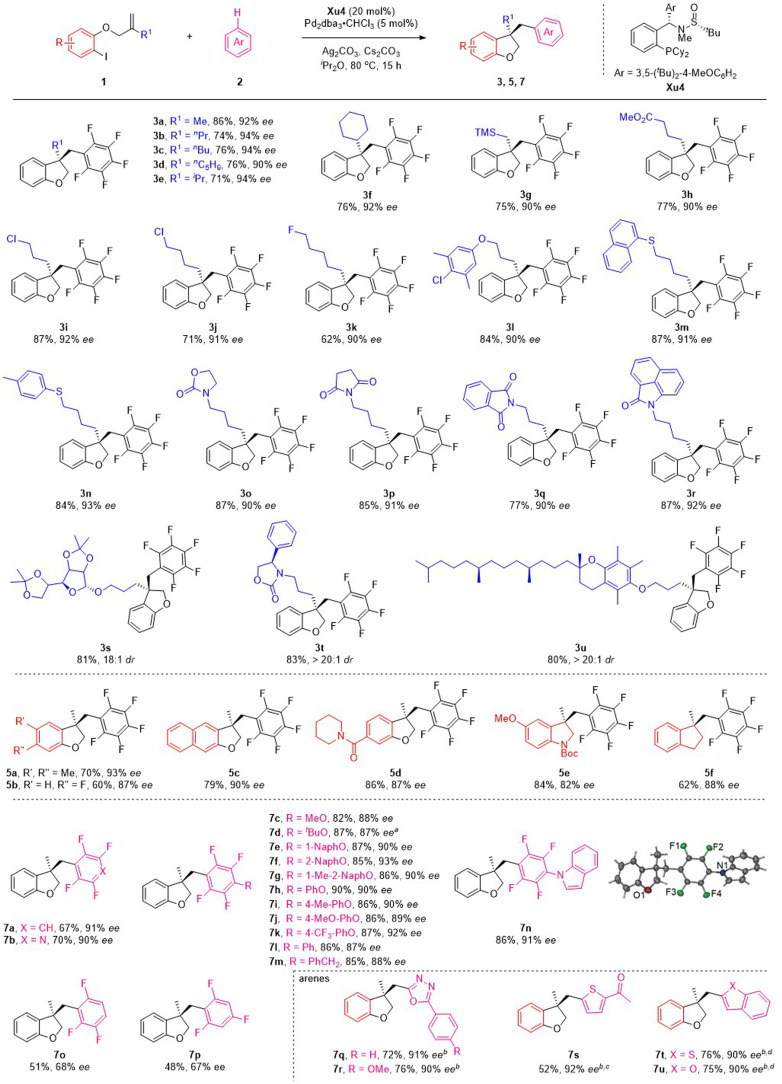
Substrate scope. Conditions: unless otherwise noted, all reactions were performed with 1a (0.3 mmol), 2a (0.9 mmol), Ag_2_CO_3_ (0.225 mmol), Cs_2_CO_3_ (0.6 mmol), 10 mol% Pd_2_dba_3_·CHCl_3_ and 20 mol% Xu4 in 3.0 mL ^i^Pr_2_O at 80 °C for 15 h. ^*a*^25 mol% Xu3 was used. ^*b*^PivOH (0.09 mmol), Pd(η-allyl)Cl_2_ (10 mol%) and Et_2_O (3 mL) were used. ^*c*^100 °C. ^*dt*^BuONa (3.0 eq.) was used.

Subsequently, the effect of substituents on the benzene ring of the *o*-iodophenol moiety was investigated under the standard reaction conditions (Scheme 3). Substituting the phenyl ring with electron-donating and electron-withdrawing groups at C4 and C5 positions appeared to have limited effects on the results, and 5a–5d were afforded in modest to good yields with excellent ee values. 3,3-Disubstituted indolines and indanes are frequently found in pharmaceuticals, natural alkaloids, and as fascinating building blocks in organic synthesis. Despite progress made in this field, the synthesis of these chiral compounds is still in high demand. Satisfactorily, the present asymmetric C–H functionalization of alkene reaction was also applicable to the substrates employing BocN and C as a tether, delivering the indoline 5e and indanes 5f with good yields and ee values.

To ascertain the scope of this method, a variety of polyfluoroarenes were further investigated (Scheme 3). Both 1,2,4,5-tetrafluorobenzene and 2,3,5,6-tetrafluoropyridine smoothly underwent the C–H functionalization process and transformed to the corresponding products (7a and 7b) in good yields with excellent ee values. For 2,3,5,6-tetrafluoroanisole derivatives, alkyl ethers (such as Me and ^*t*^Bu) and aryl ethers (such as naphthyl and phenyl groups) were also well accommodated under mild conditions, giving the desired products (7c–7h) in 82–90% yields with 87–93% ee. It is noteworthy that electron-donating groups (such as methyl and methoxy groups) and electron-withdrawing groups (such as trifluoromethyl group) on the phenyl ring were all well tolerated, furnishing the desired products (7i–7k) with good to excellent ee. Furthermore, changing the *O*-substituent to a *N*- and CH_2_-substituent on the tetrafluorobenzene ring could also smoothly drive the reaction to form products 7l–7n with satisfactory results. The absolute configuration of the product was confirmed by the X-ray diffraction analysis of 7n. Next, the scope of fluorobenzenes with fewer fluorine atoms was investigated. Unfortunately, decreasing the fluorine atoms could drive the reaction to form products with lower yield and ee (7o–7p), which might be related to the fact that more fluorine atoms can increase the p*K*_a_ value of substrates. We next investigated several heteroarene substrates. To our delight, oxadiazole 6q and 6r, thiophene 6s, benzothiophene 6t and benzofuran 6u could react smoothly, affording the corresponding products (7q–7u) with high yields (52–76%) and excellent ees (90–92%).

**Scheme 4:**
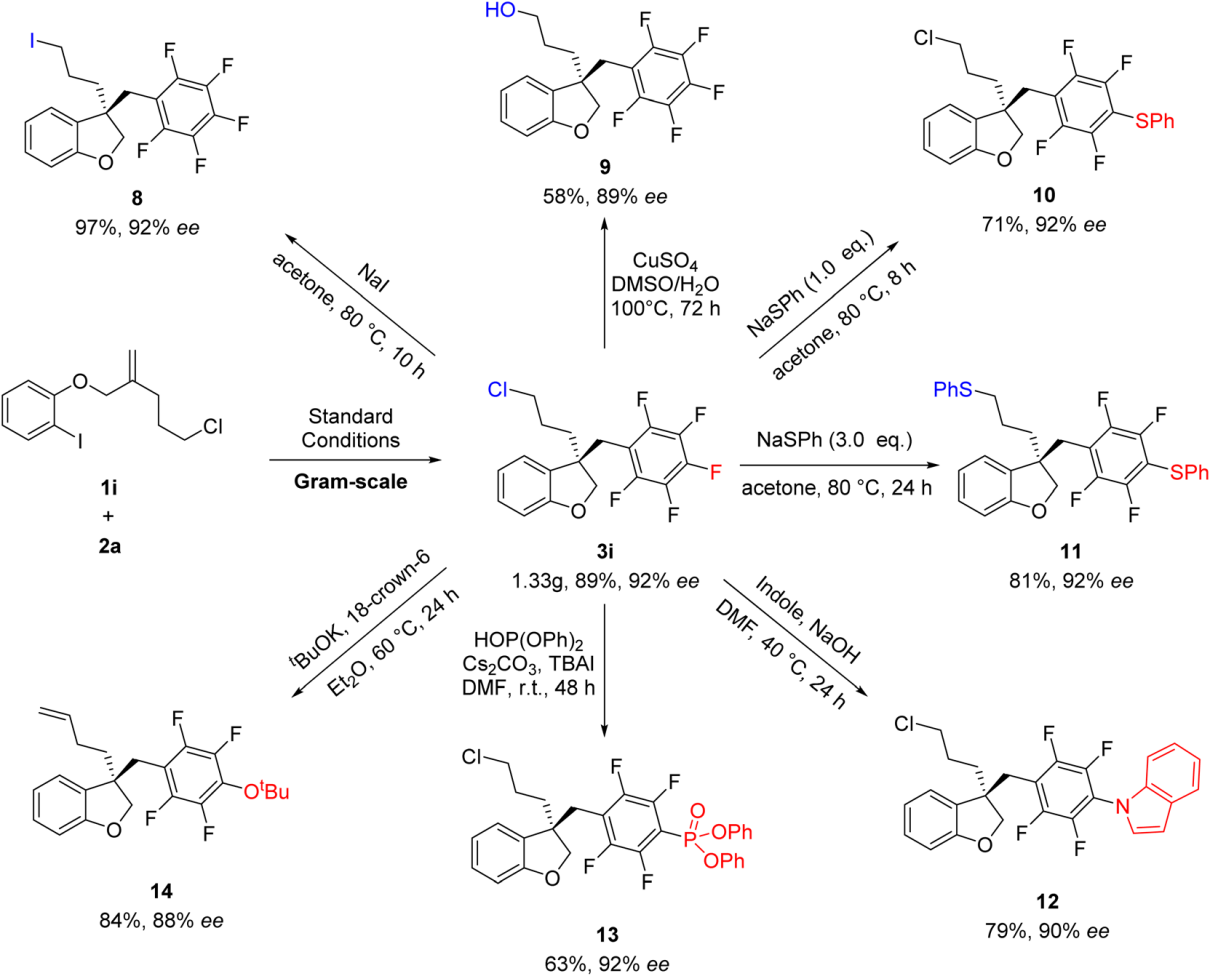
The synthetic transformation of 3i.

**Scheme 5:**
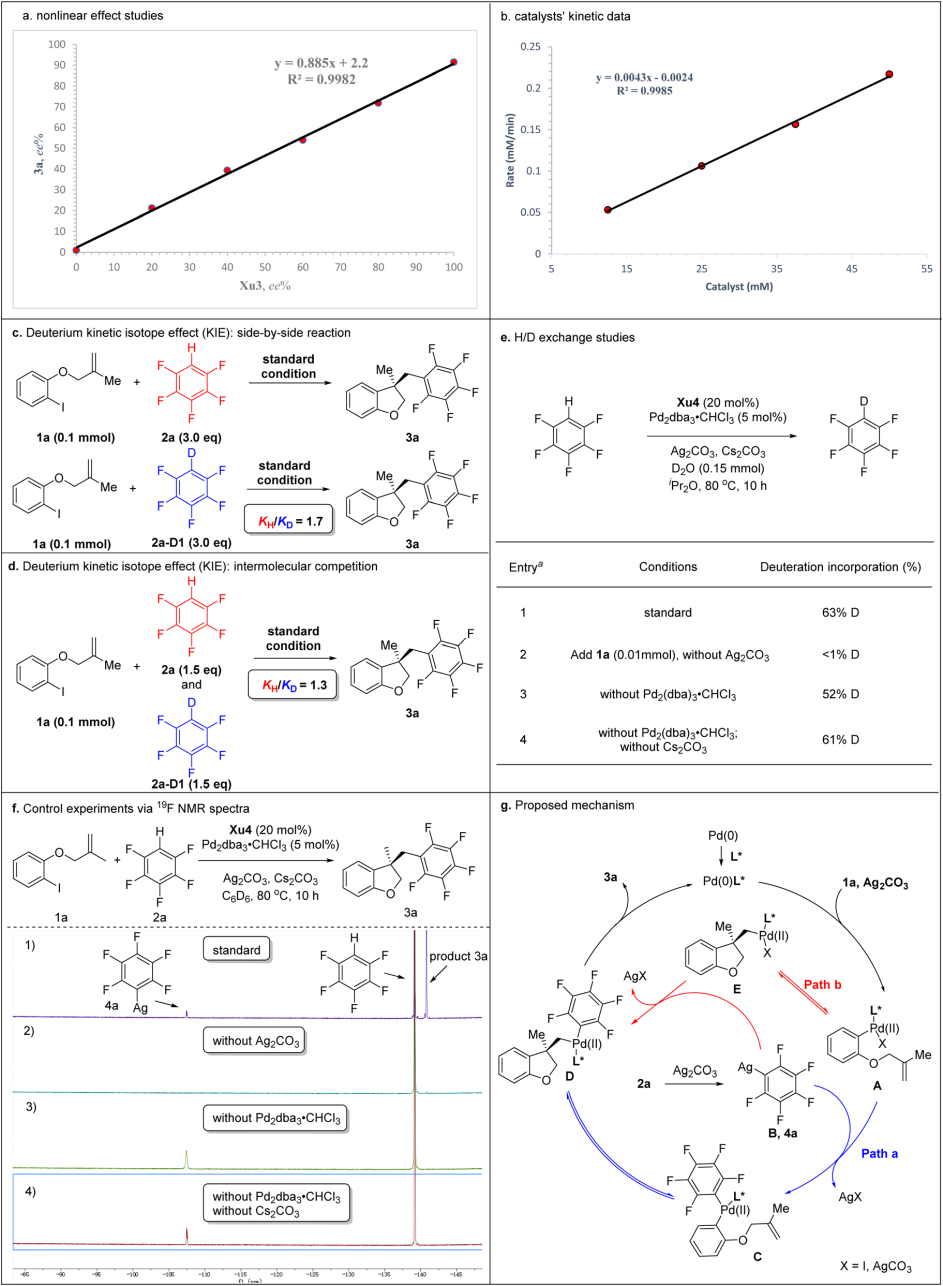
Mechanistic studies and proposed mechanism.

To further demonstrate the reliability of this method, the reaction of 1i and 2a was conducted on a larger scale of 5 mmol, affording the desired products 3i without loss of efficiency and the ee value (Scheme 4). Subsequently, synthetic transformations of 3i were carried out. As shown in Scheme 5, the Cl group could be substituted by different nucleophilic reagents, thus leading to 8 and 9 in 97 and 58% yields, respectively. It's very interesting to find that the substitution of 3i with different equivalents of NaSPh could produce 10 and 11, respectively, in high yields. Notably, 3i have two sites which can conduct nucleophilic substitution reaction. If stronger nucleophilic reagents were used, the direct functionalized of polyfluoroarenes could be selectively achieved to afford 12–14 in good yields. It was found that the C=C bond of 14 was generated through the elimination of the C–Cl bond in the presence of strong base ^*t*^BuOK.

To gain deep insight into the reaction mechanism, several control experiments were carried out (Schemes 5). Nonlinear effect studies on the enantiomeric composition of the chiral ligand Xu3 and product 3a (Scheme 5a) and initial rate experiments (Scheme 5b) indicated that there is a significant first-order dependence on the catalyst. We performed side-by-side experiments with pentafluorobenzene 2a and deuterated pentafluorobenzene 2a-[D1] to measure the initial reaction rate, respectively. The side-by-side experiments provided a *K*_H_/*K*_D_ value of 1.7 (Scheme 5c). The intermolecular competition reaction of 2a and 2a-[D1] in the same pot showed a *K*_H_/*K*_D_ value of 1.3 calculated from the consumption of 2a and 2a-[D1] (Scheme 5d). We also carried out H/D exchange experiments between C_6_F_5_H and D_2_O (5.0 equiv.). Analysis by ^2^H NMR spectroscopy showed 63% deuterium incorporation under standard conditions (Scheme 5e, entry 1). These results indicated that the C–H activation might not be the rate-determining step in this process. Moreover, adding 1a (0.1 mmol) to the reaction, <1% deuterium incorporation was detected in the absence of Ag_2_CO_3_ (Scheme 5e, entry 2). 52% and 61% deuterium incorporation was detected under standard conditions without Pd_2_dba_3_·CHCl_3_ or without Pd_2_dba_3_·CHCl_3_ and Cs_2_CO_3_, respectively (Scheme 5e, entries 3 and 4). These results suggested that Ag_2_CO_3_ was essential to activate the pentafluorobenzene. We further monitored the reaction *via*^19^F NMR spectroscopy. After 10 h, compound 4a was detected based on a diagnostic signal at approximately −107.4 ppm, which matches the C_6_F_5_Ag species chemical shift in the literature (Scheme 5f, entry 1).^24^ In addition, this same intermediate was also formed under standard conditions without Pd_2_dba_3_·CHCl_3_ or without Pd_2_dba_3_·CHCl_3_ and Cs_2_CO_3_ (Scheme 5f, entries 3 and 4), which indicated that Ag_2_CO_3_ could activate the pentafluorobenzene to afford the C_6_F_5_Ag species.

Two possible mechanisms were depicted as shown in Scheme 5g. Oxidative addition of Pd(0) with 1a afforded arylpalladium species A, followed by transmetallization with intermediate B which was generated by the reaction of 2a with Ag_2_CO_3_, resulting in the formation of complex C. The subsequent intramolecular Heck-type reaction of intermediate C provided chiral species D, which could undergo reductive elimination to produce product 3a and regenerate the Pd(0) catalyst. Alternatively, the intramolecular Heck-type reaction of intermediate A occurred firstly to generate intermediate E. Then, complex E underwent transmetallization with intermediate B to afford chiral species D. Finally, reductive elimination of D gave 3a and regenerated the Pd(0) catalyst. Notably, the mechanism involving the transformation of ArPdI(ii)L* species into positively charged ArPd(ii)L* species in the presence of silver salt could not be ruled out.

## Conclusions

In summary, with the use of diverse polyfluoro- and heteroarenes as direct arylation coupling partners, Pd/XuPhos complexes are shown to be effective catalysts for asymmetric domino Heck/intermolecular C–H alkylation of unactivated alkenes, in which, a variety of dihydrobenzofuran, indoline and indane compounds are obtained in high performance. Easily accessible substrates, mild conditions, good functional group tolerance and various synthetically transformations of the products make this protocol highly attractive. Additionally, mechanistic studies indicate that C–H activation might not be the rate-determining step in this process. We anticipate that this methodology will inspire the discovery of more novel catalyst systems for handling these valuable and challenging asymmetric transformations.

## Data availability

All data have been provided in the main text and ESI.†

## Author contributions

C. F., Q.-P. W. and B. X. carried out the experimental and data-analysis work. Z.-M. Z. and J. Z. designed the reaction, directed the project, and wrote the paper with the assistance of B. X.

## Conflicts of interest

There are no conflicts to declare.

## Supplementary Material

SC-015-D4SC00262H-s001

SC-015-D4SC00262H-s002
