# Supplementary material for: Palladium/XuPhos-catalyzed enantioselective cascade Heck/intermolecular C(sp2)–H alkylation reaction
Source: Chem Sci. 2024 Mar 9;15(15):5573–80. doi: 10.1039/d4sc00262h (PMC11023025; doi:10.1039/d4sc00262h)
Supplement: SC-015-D4SC00262H-s001 [file SC-015-D4SC00262H-s001.pdf]

Electronic Supplementary Material (ESI) for Chemical Science.
This journal is © The Royal Society of Chemistry 2024

Supporting Information

Palladium/Xu-Phos-catalyzed enantioselective cascade Heck/Intermolecular C(sp^2)−H alkylation reaction

Chao Fang[1], Quanpu Wang[1], Bing Xu[1,3], Zhan-Ming Zhang[1,2*] & Junliang Zhang[1,3,4*]

[1]Department of Chemistry, Fudan University, Shanghai, 200438, P.R.China

[2]Fudan Zhangjiang Institute, Shanghai, 201203, P.R.China

[3]Zhuhai Fudan Innovation Institute, Zhuhai, Guangdong, 519000, P.R.China

[4]School of Chemistry and Chemical Engineering, Henan Normal University, Xinxiang, Henan, 453007, P.R.China

*Corresponding Authors: zhanmingzhang@fudan.edu.cn; junliangzhang@fudan.edu.cn

Contents

1. General Information S1
2. Table S1. Screening of Solvents, Palladium Salts, Bases and silver salts for Enantioselective Cascade Heck/Intermoleculare C(sp^2)−H Alkylation Reaction .S2
3. Table S2. Screening of Solvents, Palladium Salts, additives for C(sp^2)−H Alkylation Reaction with oxadiazole S3
4. Table S3. Screening of Solvents and temperature for C(sp^2)−H Alkylation Reaction with thiophene..S3
5. Table S4. Screening of Solvents, base and temperature and for C(sp^2)−H Alkylation Reaction with benzothiophene S4
6. Table S5. Screening of Solvents, Palladium Salts, Bases for C(sp^2)−H Alkylation Reaction with other fluorobenzenes with fewer fluorine atoms. S5
7. General Procedure for the Synthesis of products **3a-3u**, **4a-4f**, **6a-6n**. S6
8. General Procedure for the Synthesis of products **8-14**. S41
9. Non-linear effect experiments..S48
10. Kinetic Data: Order in Catalyst...S49

11. Kinetic Data: Deuterium Kinetic Isotope Experiment S52

12. Control experiments of C_6F_5Ag and H/D exchanging experiments S56

13. X-Ray Structure and Crystal Data of **7n**..S58

14. ^{1}H , ^{19}F, ^{31}P, ^{13}C NMR, HPLC Spectra...S63

15. References...S196

1. General Information

All reactions were carried out under an atmosphere of argon in sealed tube with magnetic stirring. ^{1}H NMR spectra, ^{19}F NMR spectra, ^{31}P NMR spectra, ^{13}C NMR spectra were recorded on a Bruker 300, 400 and 500 MHz spectrometer in $CDCl_3$. All signals are reported in ppm with the internal TMS signal at 0 ppm as a standard. Data for ^{1}H NMR spectra are reported as follows: chemical shift (ppm, referenced to TMS; s = singlet, d = doublet, t = triplet, dd = doublet of doublets, m = multiplet), coupling constant (Hz), and intergration. Data for ^{13}C NMR are reported in terms of chemical shift (ppm) relative to residual solvent peak ($CDCl_3$: 77.0 ppm). Reactions were monitored by thin layer chromatography (TLC) using silica gel plates. Flash column chromatography was performed over silica gel (300-400 mesh). The substrates **1a-1v**, **4a-4f**, **6a-6n** were synthesized according to published procedures.[1,2] The spectral data of the substrates were consisted with that reported in the literature. A Sadphos kit was purchased from Daicel. The enantionmeric excesses of the products were determined by chiral stationary phase HPLC using a Chiralpak IA, IB, IC, IE, IF, IG, ODH, OJH, OZ3, ADH.

2. Table S1. Scr*ee*ning of Solvents, Palladium Salts, Bases and silver salts for Enantioselective Cascade Heck/Intermolecular C(sp^2)−H Alkylation Reaction[a]

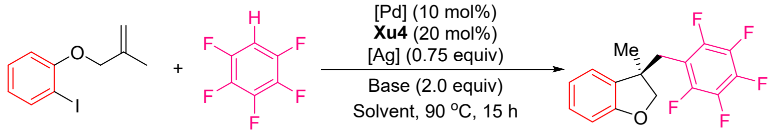

1a 2a 3a

Entry	Pd	Base	Solvent	Ag	**3a** Yield(%)[b]	**3a** *Ee*(%)[c]
1	$Pd_2(dba)_3$	K_2CO_3	Et_2O	Ag_2CO_3	66	76
2	$Pd_2(dba)_3$	K_2CO_3	$^{i}Pr_2O$	Ag_2CO_3	80	84
3	$Pd_2(dba)_3$	K_2CO_3	MTBE	Ag_2CO_3	74	80
4	$Pd_2(dba)_3$	K_2CO_3	DCM	Ag_2CO_3	44	28
5	$Pd_2(dba)_3$	K_2CO_3	DMF	Ag_2CO_3	10	5
6	$Pd_2(dba)_3$	K_2CO_3	$PhCF_3$	Ag_2CO_3	54	68
7	$Pd_2(dba)_3$	K_2CO_3	CH_3CN	Ag_2CO_3	45	49
8	$Pd_2(dba)_3$	K_2CO_3	DCE	Ag_2CO_3	48	35
9	$Pd_2(dba)_3$	K_2CO_3	1,4-Dioxane	Ag_2CO_3	70	61
10	$Pd_2(dba)_3$	K_2CO_3	DMSO	Ag_2CO_3	-	-
11	Pd-G3	K_2CO_3	$^{i}Pr_2O$	Ag_2CO_3	76	83
12	$Pd_2(dba)_3$•$CHCl_3$	K_2CO_3	$^{i}Pr_2O$	Ag_2CO_3	82	88
13	$PdCl_2$	K_2CO_3	$^{i}Pr_2O$	Ag_2CO_3	80	78
14	$[PdCl(allyl)]_2$	K_2CO_3	$^{i}Pr_2O$	Ag_2CO_3	78	75
15	$Pd(OAc)_2$	K_2CO_3	$^{i}Pr_2O$	Ag_2CO_3	24	25
16	$Pd(TFA)_2$	K_2CO_3	$^{i}Pr_2O$	Ag_2CO_3	60	32
17	$Pd(acac)_2$	K_2CO_3	$^{i}Pr_2O$	Ag_2CO_3	15	3
18	$Pd(MeCN)_2(OTs)_2$	K_2CO_3	$^{i}Pr_2O$	Ag_2CO_3	34	24
19	$Pd(dmdba)_2$	K_2CO_3	$^{i}Pr_2O$	Ag_2CO_3	40	92
20	$Pd_2(dba)_3$•$CHCl_3$	Cs_2CO_3	$^{i}Pr_2O$	Ag_2CO_3	83	90
21	$Pd_2(dba)_3$•$CHCl_3$	CsOAc	$^{i}Pr_2O$	Ag_2CO_3	52	21
22	$Pd_2(dba)_3$•$CHCl_3$	$CsCO_2H$	$^{i}Pr_2O$	Ag_2CO_3	-	-
23	$Pd_2(dba)_3$•$CHCl_3$	CsTFA	$^{i}Pr_2O$	Ag_2CO_3	48	4
24	$Pd_2(dba)_3$•$CHCl_3$	CsF	$^{i}Pr_2O$	Ag_2CO_3	78	82
25	$Pd_2(dba)_3$•$CHCl_3$	CsOPiv	$^{i}Pr_2O$	Ag_2CO_3	46	7
26	$Pd_2(dba)_3$•$CHCl_3$	NaO^tBu	$^{i}Pr_2O$	Ag_2CO_3	72	65
27	$Pd_2(dba)_3$•$CHCl_3$	KO^tBu	$^{i}Pr_2O$	Ag_2CO_3	74	63
28	$Pd_2(dba)_3$•$CHCl_3$	NaOH	$^{i}Pr_2O$	Ag_2CO_3	70	79

29	$Pd_2(dba)_3$•$CHCl_3$	KOH	$^{i}Pr_2O$	Ag_2CO_3	70	80
30	$Pd_2(dba)_3$•$CHCl_3$	Cs_2CO_3	$^{i}Pr_2O$	AgF	58	85
31	$Pd_2(dba)_3$•$CHCl_3$	Cs_2CO_3	$^{i}Pr_2O$	AgCl	68	88
33	$Pd_2(dba)_3$•$CHCl_3$	Cs_2CO_3	$^{i}Pr_2O$	AgOAc	36	50
34	$Pd_2(dba)_3$•$CHCl_3$	Cs_2CO_3	$^{i}Pr_2O$	$AgNO_3$	69	87
35	$Pd_2(dba)_3$•$CHCl_3$	Cs_2CO_3	$^{i}Pr_2O$	None	Trace	-

[a]Unless otherwise noted, all reactions were performed with 1a (0.1 mmol), 2a (0.3 mmol), Ag_2CO_3 (0.075 mmol), Base (0.2 mmol), 10 mol% [Pd] and 20 mol% ligand in 1.0 mL solvent at 90 °C for 15-48 h. [b]NMR yield with CH_2Br_2 as an internal standard. [c] Enantionselectivity Determined by chiral HPLC.

3. Table S2. Screening of Solvents, Palladium Salts, additives for C(sp^2)−H Alkylation Reaction with oxadiazole

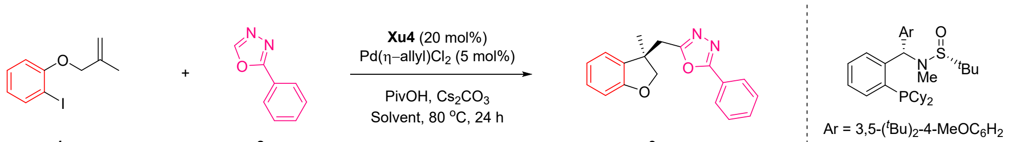

1 8a 9a Xu4

Entry[a]	Solvent	**9a** Yield(%)[b]	**9a** *Ee*(%)[c]
1	THF	76	76
2	Et_2O	72 (70)	84
3	$^{i}Pr_2O$	68	80
4	MTBE	71	28
5	DCM	40	5
6	DMF	28	68
7	$PhCF_3$	42	49
8	CH_3CN	85	35
9	DCE	55	61
10	Dixane	78	88

[a]Unless otherwise noted, all reactions were performed with 1 (0.2 mmol), 8a (0.1 mmol), PivOH (0.03 mmol), Cs_2CO_3 (0.2 mmol), 10 mol% [Pd] and 20 mol% ligand in 1.0 mL solvent at 80 °C for 24-30 h. [b]NMR yield with CH_2Br_2 as an internal standard. [c]Enantionselectivity Determined by chiral HPLC.

4. Table S3. Screening of Solvents and temperature for C(sp^2)−H Alkylation Reaction with thiophene

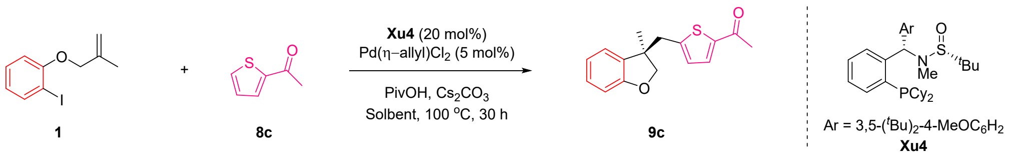

Entry[a]	Solvent	**9c** Yield(%)[b]	**9c** *Ee*(%)[c]
1	THF	24	87
2	THF	46	84
3	Et_2O	51	91
4	$^{i}Pr_2O$	57 (52)	92
5	MTBE	54	92
6	DCM	trace	-
7	$PhCF_3$	38	91
8	Tol	32	90
9	CH_3CN	53	70
10	DCE	trace	-
11	Dixane	57	87
12	DMSO	30	87
13	DMA	22	87

[a]Unless otherwise noted, all reactions were performed with 1 (0.2 mmol), 9c (0.1 mmol), PivOH (0.03 mmol), Cs_2CO_3 (0.2 mmol), 10 mol% [Pd] and 20 mol% ligand in 1.0 mL solvent at 100 °C for 30 h. [b]NMR yield with CH_2Br_2 as an internal standard. [c]Enantionselectivity Determined by chiral HPLC. [d]80 °C

5. Table S4. Screening of Solvents, base and temperature and for C(sp^2)−H Alkylation Reaction with benzothiophene

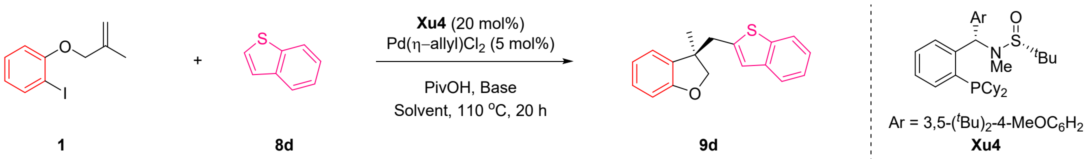

Entry	Solvent	**9d** Yield(%)[b]	**9d** *Ee*(%)[c]	Entry	Base	**9d** Yield(%)[b]	**9d** *Ee*(%)[c]
1	THF	21	70	13	K_2CO_3	66	76
2	Et_2O	23	84	14	CsTFA	80	84
3	$^{i}Pr_2O$	18	82	15	CsF	74	80
4	MTBE	24	82	16	CsOPiv	44	28
5	DCM	trace	-	17	NaO^{t}Bu	10	5
6	$PhCF_3$	12	73	18	KO^{t}Bu	54	68
7	Tol	15	78	19	NaOH	45	49
8	CH_3CN	26	60	20	KOH	48	35

9	DCE	trace	-	21[d]	Cs_2CO_3	70	61
10	Dixane	24	75	22[e]	Cs_2CO_3	-	-
11	DMSO	78	13	23[f]	NaO^tBu	76	83
12	DMA	71	42	24[g]	NaO^tBu	82	88

[a]Unless otherwise noted, all reactions were performed with 1a (0.1 mmol), 8d (0.3 mmol), Ag_2CO_3 (0.075 mmol), Base (0.2 mmol), 10 mol% [Pd] and 20 mol% ligand in 1.0 mL solvent at 110 °C for 15-48 h. [b]NMR yield with CH_2Br_2 as an internal standard. [c]Enantionselectivity Determined by chiral HPLC. [d]DMSO (10 uL). [d]DMSO (50 uL).[f] 100 °C. [g]80 °C

6. Table S5. Screening of Solvents, Palladium Salts, Bases for C(sp2)−H Alkylation Reaction with other fluorobenzenes with fewer fluorine atoms.

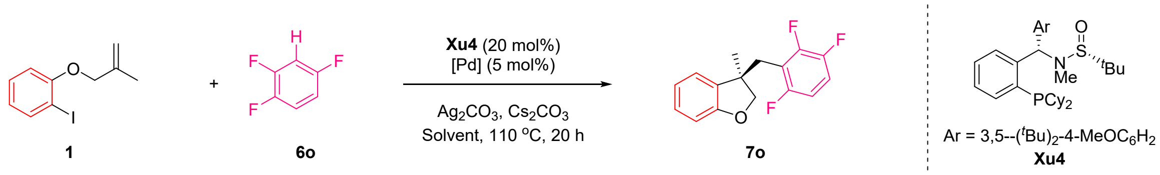

Entry [a]	Pd	Base	Solvent	Ag	**7o** Yield(%)[b]	**7o** *Ee*(%)[c]
1	$Pd_2(dba)_3$•$CHCl_3$	Cs_2CO_3	Et_2O	Ag_2CO_3	48	59
2	$Pd_2(dba)_3$•$CHCl_3$	Cs_2CO_3	$^iPr_2O$	Ag_2CO_3	51	68
3	$Pd_2(dba)_3$•$CHCl_3$	Cs_2CO_3	MTBE	Ag_2CO_3	46	64
4	$Pd_2(dba)_3$•$CHCl_3$	Cs_2CO_3	DCM	Ag_2CO_3	trace	-
5	$Pd_2(dba)_3$•$CHCl_3$	Cs_2CO_3	Tol	Ag_2CO_3	53	60
6	$Pd_2(dba)_3$•$CHCl_3$	Cs_2CO_3	$PhCF_3$	Ag_2CO_3	54	58
7	$Pd_2(dba)_3$•$CHCl_3$	Cs_2CO_3	CH_3CN	Ag_2CO_3	trace	-
8	$Pd_2(dba)_3$•$CHCl_3$	Cs_2CO_3	Dioxane	Ag_2CO_3	32	64
9	$Pd_2(dba)_3$•$CHCl_3$	Cs_2CO_3	DMSO	Ag_2CO_3	trace	-
10	$Pd_2(dba)_3$•$CHCl_3$	Cs_2CO_3	DMA	Ag_2CO_3	70	20
11	Pd-G3	Cs_2CO_3	$^iPr_2O$	Ag_2CO_3	34	60
12	$Pd_2(dba)_3$•$CHCl_3$	Cs_2CO_3	$^iPr_2O$	Ag_2CO_3	51	68
13	$PdCl_2$	Cs_2CO_3	$^iPr_2O$	Ag_2CO_3	53	66
14	$[PdCl(allyl)]_2$	Cs_2CO_3	$^iPr_2O$	Ag_2CO_3	46	67
15	$Pd(OAc)_2$	Cs_2CO_3	$^iPr_2O$	Ag_2CO_3	67	54
16	$[Pd(dmba)Cl]_2$	Cs_2CO_3	$^iPr_2O$	Ag_2CO_3	55	64
17	$Pd(cod)_2Cl_2$	Cs_2CO_3	$^iPr_2O$	Ag_2CO_3	62	60
18	$Pd(dba)_2$	Cs_2CO_3	$^iPr_2O$	Ag_2CO_3	48	67

19	$Pd(dmdba)_2$	Cs_2CO_3	$^{i}Pr_2O$	Ag_2CO_3	69	62
20	$Pd_2(dba)_3$•$CHCl_3$	K_2CO_3	$^{i}Pr_2O$	Ag_2CO_3	51	67
21	$Pd_2(dba)_3$•$CHCl_3$	CsTFA	$^{i}Pr_2O$	Ag_2CO_3	56	40
22	$Pd_2(dba)_3$•$CHCl_3$	CsF	$^{i}Pr_2O$	Ag_2CO_3	trace	-
23	$Pd_2(dba)_3$•$CHCl_3$	CsOPiv	$^{i}Pr_2O$	Ag_2CO_3	15	11
24	$Pd_2(dba)_3$•$CHCl_3$	NaO^tBu	$^{i}Pr_2O$	Ag_2CO_3	63	60
25	$Pd_2(dba)_3$•$CHCl_3$	KO^tBu	$^{i}Pr_2O$	Ag_2CO_3	21	41
28	$Pd_2(dba)_3$•$CHCl_3$	NaOH	$^{i}Pr_2O$	Ag_2CO_3	53	52
29	$Pd_2(dba)_3$•$CHCl_3$	KOH	$^{i}Pr_2O$	Ag_2CO_3	23	21
30	$Pd_2(dba)_3$•$CHCl_3$	Cs_2CO_3	$^{i}Pr_2O$	Ag_2CO_3	53	68
31	$Pd_2(dba)_3$•$CHCl_3$	CsOAc	$^{i}Pr_2O$	Ag_2CO_3	30	25

[a]Unless otherwise noted, all reactions were performed with 1a (0.1 mmol), 6o (0.3 mmol), Ag_2CO_3 (0.075 mmol), Base (0.2 mmol), 10 mol% [Pd] and 20 mol% ligand in 1.0 mL solvent at 80 °C for 20-48 h. [b]NMR yield with CH_2Br_2 as an internal standard. [c] Enantionselectivity Determined by chiral HPLC.

7. General Procedure for the Synthesis of products 3a-3u, 5a-5f, 7a-7n, 9a-9e.

Typical procedure A for the 2,3-dihydrobenzofuran derivatives.

To a 10 mL oven-dried sealed tube was added substrate **1** (0.30 mmol, 1.0 equiv.), $Pd_2(dba)_3$•$CHCl_3$ (15.5 mg, 0.015 mmol, 5 mol%), **Xu4** (38.3 mg, 0.06 mmol, 20 mol%), Cs_2CO_3 (195.5 mg, 0.6 mmol, 3.0 equiv.), Ag_2CO_3 (61.6 mg, 0.225 mmol, 0.75 equiv.). The flask was evacuated and refilled with argon. Then, substrate **2, 4 or 6** (0.90 mmol, 3.0 equiv.), $^{i}Pr_2O$ (3 mL) was added to the tube, and stirred at room temperature for 1 h. Then the mixture was stirred at 80 °C for 15-48 h. After the reaction was complete (monitored by TLC), solvent was removed under reduced pressure. The crude product was then purified by flash column chromatography on silica gel to afford the desired product **3, 5, 7**.

Typical procedure B for the Heteroarene substrates derivatives.

To a 10 mL oven-dried sealed tube was added substrate **1** (0.30 mmol, 3.0 equiv.), $[Pd(\eta\text{-allyl})Cl]_2$ (5.4 mg, 0.015 mmol, 5 mol%), **Xu4** (38.3 mg, 0.06 mmol, 20 mol%),

Cs_2CO_3 (195.5 mg, 0.6 mmol, 3.0 equiv.), PivOH (9.0 mg, 0.09 mmol, 0.03 equiv.). The flask was evacuated and refilled with argon. Then, substrate **8** (0.10 mmol, 1.0 equiv.), Et_2O (3 mL) was added to the tube, and stirred at room temperature for 1 h. Then the mixture was stirred at 80 °C for 24-48 h. After the reaction was complete (monitored by TLC), solvent was removed under reduced pressure. The crude product was then purified by flash column chromatography on silica gel to afford the desired product **9**.

7.1 Synthesis of (*R*)-3-methyl-3-((perfluorophenyl)methyl)-2,3-dihydrobenzofuran (**3a**).

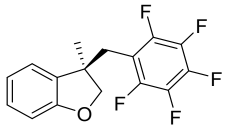

3a

Prepared according to typical procedure **A** from allyl ether **1a** (82.2 mg, 0.3 mmol), after a flash column chromatography (hexanes: Et_2O = 100:1) afforded the product **3a** as a colorless liquid (81.0 mg, 86% yield) with 92% *ee*. ^{1}H NMR (400 MHz, $CDCl_3$) δ 7.18-7.14 (m, 1 H), 7.01 (dd, J = 7.5, 1.4 Hz, 1 H), 6.90-6.86 (m, 1 H), 6.77 (d, J = 8.0 Hz, 1 H), 4.50 (d, J = 9.0 Hz, 1 H), 4.11 (d, J = 9.0 Hz, 1 H), 3.07-2.96 (m, 2 H), 1.41 (s, 3 H). ^{13}C NMR (101 MHz, $CDCl_3$) δ 159.33, 146.65, 144.21, 141.18 (m, J_F = 49.2 Hz), 138.64-138.60 (m, J_F = 79.7 Hz), 136.10, 111.57-110.24 (m, J_F = 33.0 Hz), 133.06, 128.82, 122.80, 120.74, 109.91, 82.14 (t, J_F = 2.5 Hz), 46.65, 32.79, 23.75. ^{19}F NMR (376 MHz, $CDCl_3$) δ -140.33 (dd, J = 23.2, 8.1 Hz), -155.93, -162.34 – -162.43 (m). HRMS (EI) calculated for $[C_{16}H_{11}F_5O]^+$: 314.0725 found: 314.0724. Enantiomeric excess was determined by HPLC with a Chiralpak OZ3 column (hexanes = 100, 1.0 mL/min, 210 nm); minor enantiomer tr = 20.0 min, major enantiomer tr = 22.5 min. $[\alpha]_D^{20}$ = 21.6 (*c* = 0.5, $CHCl_3$).

7.2 Synthesis of (*R*)-3-((perfluorophenyl)methyl)-3-propyl-2,3-dihydrobenzofuran (**3b**).

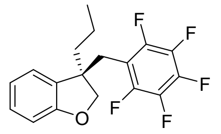

3b

Prepared according to typical procedure from allyl ether **1b** (90.7 mg, 0.3 mmol), after a flash column chromatography (hexanes: Et_2O = 100:1) afforded the product **3b** as a colorless liquid (75.8 mg, 74% yield) with 94% *ee*. ^{1}H NMR (400 MHz, $CDCl_3$) δ 7.15-7.11 (m, 1 H), 6.95 (d, J = 7.4 Hz, 1 H), 6.87-6.81 (m, 1 H), 6.72 (d, J = 8.0 Hz, 1 H), 4.44 (d, J = 9.2 Hz, 1 H), 4.28 (d, J = 9.2 Hz, 1 H), 3.11-2.99 (m, 2 H), 1.77-1.66 (m, 2 H), 1.49-1.36 (m, 1 H), 1.22-1.07 (m, 1 H), 0.92 (t, J = 7.3 Hz, 3 H). ^{13}C NMR (101 MHz, $CDCl_3$) δ 159.80, 146.72, 144.26, 141.14 (m, J_F = 49.2 Hz), 138.56(m, J_F = 79.7 Hz), 136.04, 131.37, 128.81, 123.43, 120.52, 111.43 (m, J_F = 38.0 Hz), 109.63, 79.88 (t, J_F = 2.8 Hz), 50.35, 39.75, 31.64, 17.85, 14.52. ^{19}F NMR (376 MHz, $CDCl_3$) δ -140.06 (dd, J = 22.5, 8.1 Hz), -156.10 (t, J = 20.8 Hz), -162.51– -162.65 (m). HRMS (EI) calculated for $[C_{18}H_{15}F_5O]^+$: 342.1038 found: 342.1035. Enantiomeric excess was determined by HPLC with a Chiralpak IA column (hexanes = 100, 1.0 mL/min, 210 nm); minor enantiomer tr = 10.0 min, major enantiomer tr = 11.0 min. $[\alpha]_D^{20}$ = 8.3 (*c* = 0.3, $CHCl_3$).

7.3 Synthesis of (*R*)-3-butyl-3-((perfluorophenyl)methyl)-2,3-dihydrobenzofuran (**3c**).

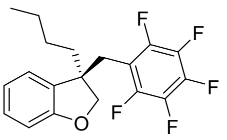

3c

Prepared according to typical procedure from allyl ether **1c** (94.9 mg, 0.3 mmol), after a flash column chromatography (hexanes: Et_2O = 100:1) afforded the product **3c** as a colorless liquid (81.2 mg, 76% yield) with 94% *ee*. ^{1}H NMR (400 MHz, $CDCl_3$) δ 7.13 (td, J = 7.7, 1.4 Hz, 1 H), 6.94 (dd, J = 7.5, 1.4 Hz, 1 H), 6.85 (d, J = 0.9 Hz, 1 H), 6.71 (d, J = 8.0 Hz, 1 H), 4.43 (d, J = 9.2 Hz, 1 H), 4.27 (d, J = 9.2 Hz, 1 H), 3.11-2.98 (m, 2 H), 1.78-1.68 (m, 2 H), 1.42-1.26 (m, 3 H), 1.18- 1.04 (m, 1 H), 0.88 (t, J = 7.2 Hz, 3 H). ^{13}C NMR (101 MHz, $CDCl_3$) δ 159.75, 146.67, 144.22, 141.10 (m, J_F = 49.2 Hz), 138.56 (m, J_F = 79.7 Hz), 136.00, 131.33, 128.78, 123.43, 120.51, 111.41 (m, J_F = 38.0

Hz), 109.62, 79.85 (d, J_F = 2.8 Hz), 50.25, 37.15, 31.68, 26.64, 23.17, 13.90. ^{19}F NMR (376 MHz, $CDCl_3$) δ -140.02 (dd, J = 22.6, 8.1 Hz), -156.04 (t, J = 21.2 Hz), -162.55 (td, J = 22.0, 8.0 Hz). HRMS (EI) calculated for $[C_{19}H_{17}F_5O]^+$: 356.1194 found: 356.1198. Enantiomeric excess was determined by HPLC with a Chiralpak IA column (hexanes = 100, 1.0 mL/min, 210 nm); minor enantiomer tr = 10.1 min, major enantiomer tr = 15.0 min. $[\alpha]_D^{20}$ = 8.3 (*c* = 0.5, $CHCl_3$).

7.4 Synthesis of (*R*)-3-pentyl-3-((perfluorophenyl)methyl)-2,3-dihydrobenzofuran (**3d**).

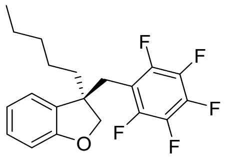

3d

Prepared according to typical procedure from allyl ether **1d** (99.1 mg, 0.3 mmol), after a flash column chromatography (hexanes: Et_2O = 100:1) afforded the product **3d** as a colorless liquid (84.0 mg, 76% yield) with 90% *ee*. ^{1}H NMR (400 MHz, $CDCl_3$) δ 7.15-7.11 (m, 1 H), 6.95-6.93 (m, 1 H), 6.88 (td, J = 7.4, 1.0 Hz, 1 H), 6.74 (dt, J = 8.0, 0.7 Hz, 1 H), 4.46 (d, J = 9.2 Hz, 1 H), 4.30 (d, J = 9.2 Hz, 1 H), 3.11-2.98 (m, 2 H), 1.79-1.68 (m, 2 H), 1.45-1.36 (m, 1 H), 1.31-1.22 (m, 4 H), 1.17-1.09 (m, 1 H), 0.89-0.85 (m, 3 H).^{13}C NMR (101 MHz, $CDCl_3$) δ 159.76, 146.66, 144.22, 141.10 (m, J_F = 49.2 Hz), 138.51(m, J_F = 79.7 Hz), 135.99, 131.34, 128.79, 123.41, 120.51, 111.43 (m, J_F = 33.0 Hz), 109.62, 79.83 (t, J_F = 2.8 Hz), 50.29, 37.37, 32.30, 31.69, 24.16, 22.45, 13.98.^{19}F NMR (376 MHz, $CDCl_3$) δ -139.98 – -140.06 (m), -156.04 (t, J = 21.0 Hz), -162.47 – -162.61 (m). HRMS (EI) calculated for $[C_{20}H_{19}F_5O]$+: 370.1351 found: 370.1351. Enantiomeric excess was determined by HPLC with a Chiralpak IA column (hexanes: 2-propanol = 99:1, 1.0 mL/min, 210 nm); minor enantiomer tr = 4.6 min, major enantiomer tr = 5.0 min. $[\alpha]_D^{20}$ = 15.3 (*c* = 0.2, $CHCl_3$).

7.5 Synthesis of (*S*)-3-isopropyl-3-((perfluorophenyl)methyl)-2,3-dihydrobenzofuran (**3e**).

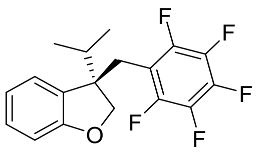

3e

Prepared according to typical procedure from allyl ether **1e** (90.7 mg, 0.3 mmol), after a flash column chromatography (hexanes: Et_2O = 100:1) afforded the product **3e** as a colorless liquid (73.2 mg, 71% yield) with 94% *ee*. ^{1}H NMR (400 MHz, $CDCl_3$) δ 7.09 (td, J = 7.7, 1.4 Hz, 1 H), 6.96 (d, J = 7.4 Hz, 1 H), 6.82 (t, J = 7.4 Hz, 1 H), 6.64 (d, J = 8.0 Hz, 1 H), 4.36 (q, J = 9.5 Hz, 2 H), 3.17-3.04 (m, 2 H), 2.20-2.13 (m, J = 6.8 Hz, 1 H), 1.08 (d, J = 6.8 Hz, 3 H), 0.89 (d, J = 6.8 Hz, 3 H). ^{13}C NMR (101 MHz, $CDCl_3$) δ 159.97, 146.64, 144.22, 140.99 (m, J_F = 49.2 Hz), 138.48 (m, J_F = 79.7 Hz), 136.08, 129.59, 128.76, 124.46 (d, J_F = 22.2 Hz), 120.19, 111.68 (m, J_F = 33.0 Hz), 109.38, 54.13, 33.83, 30.45, 18.58, 17.73. ^{19}F NMR (376 MHz, $CDCl_3$) δ -139.72 (d, J = 15.1 Hz), -156.19, -162.80 (d, J = 6.2 Hz). HRMS (EI) calculated for $[C_{18}H_{15}F_5O]^+$: 342.1038 found: 342.1040. Enantiomeric excess was determined by HPLC with a Chiralpak IA column (hexanes = 100, 1.0 mL/min, 210 nm); minor enantiomer tr = 10.0 min, major enantiomer tr = 14.0 min. $[\alpha]_D^{20}$ = 26.1 (*c* = 0.4, $CHCl_3$).

7.6 Synthesis of (*S*)-3-cyclohexyl-3-((perfluorophenyl)methyl)-2,3-dihydrobenzofuran (**3f**).

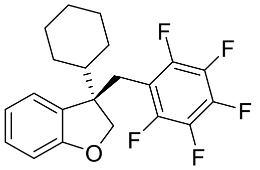

3f

Prepared according to typical procedure from allyl ether **1f** (102.7 mg, 0.3 mmol), after a flash column chromatography (hexanes: Et_2O = 100:1) afforded the product **3f** as a colorless liquid (86.7 mg, 76% yield) with 92% *ee*. ^{1}H NMR (400 MHz, $CDCl_3$) δ 7.11-7.07 (m, 1 H), 6.90 (d, J = 7.4 Hz, 1 H), 6.81 (t, J = 7.4 Hz, 1 H), 6.64 (d, J = 8.0 Hz, 1 H), 4.39 (d, J = 9.4 Hz, 1 H), 4.32 (d, J = 9.4 Hz, 1 H), 3.16-3.03 (m, 2 H), 2.12 (dt, J = 12.9, 3.2 Hz, 1 H), 1.88-1.83 (m, 1 H), 1.78-1.68 (m, 3 H), 1.60-1.56 (m, 1 H), 1.36-0.88 (m, 6 H). ^{13}C NMR (101 MHz, $CDCl_3$) δ 159.96, 146.67, 144.23,140.98 (m, J_F =

49.2 Hz), 138.37 (m, J_F = 79.7 Hz), 135.87, 129.40, 128.69, 124.59, 120.09, 111.67 (m, J_F = 33.3 Hz), 109.42, 53.95, 43.86, 30.11, 28.36, 27.93, 26.57, 26.36. ^{19}F NMR (376 MHz, $CDCl_3$) δ -139.81 – -139.89 (m), -156.25 (t, J = 20.9 Hz), -162.84 (td, J = 22.1, 7.6 Hz). HRMS (EI) calculated for $[C_{21}H_{19}F_5O]^+$: 382.1351 found: 382.1351. Enantiomeric excess was determined by HPLC with a Chiralpak IG column (hexanes: 2-propanol = 99:1, 0.3 mL/min, 210 nm); minor enantiomer tr = 14.8 min, major enantiomer tr = 18.6 min. $[\alpha]_D^{20}$ = 23.6 (*c* = 0.3, $CHCl_3$).

7.7 Synthesis of (*S*)-trimethyl((3-((perfluorophenyl)methyl)-2,3-dihydrobenzofuran-3-yl)methyl)silane (**3g**).

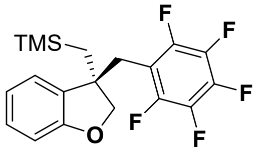

3g

Prepared according to typical procedure from allyl ether **1g** (99.7 mg, 0.3 mmol), after a flash column chromatography (hexanes: Et_2O = 100:1) afforded the product **3g** as a yellow liquid (82.4 mg, 75% yield) with 90% *ee*. ^{1}H NMR (400 MHz, $CDCl_3$) δ 7.13-7.09 (m, 1 H), 7.03 (d, J = 7.4 Hz, 1 H), 6.88-6.84 (m, 1 H), 6.68 (d, J = 8.0 Hz, 1 H), 4.51 (d, J = 9.2 Hz, 1 H), 4.23 (d, J = 9.2 Hz, 1 H), 3.13-2.97 (m, 2 H), 1.26-1.16 (m, 2 H), -0.06 (s, 9 H). ^{13}C NMR (101 MHz, $CDCl_3$) δ 159.11, 146.66, 144.27, 141.06 (m, J_F = 49.2 Hz), 138.55 (m, J_F = 79.7 Hz), 136.00, 133.17, 128.74, 123.55, 120.58, 111.58 (m, J_F = 33.2 Hz), 109.61, 81.09 (t, J_F = 3.2 Hz), 9.38, 35.28, 27.57, 0.06. ^{19}F NMR (376 MHz, $CDCl_3$) δ -139.85 (dd, J = 22.6, 8.0 Hz), -156.07 (t, J = 20.8 Hz), -162.51 – -162.65 (m). HRMS (ESI) calculated for $[M+H]^+$ = $[C_{19}H_{20}F_5OSi]^+$: 387.1198 found: 387.1204. Enantiomeric excess was determined by HPLC with a Chiralpak ODH column (hexanes = 100, 1.0 mL/min, 210 nm); minor enantiomer tr = 8.9 min, major enantiomer tr = 9.6 min. $[\alpha]_D^{20}$ = 4.2 (*c* = 0.5, $CHCl_3$).

7.8 Synthesis of methyl (*R*)-4-(3-((perfluorophenyl)methyl)-2,3-dihydrobenzofuran-3-yl)butanoate (**3h**).

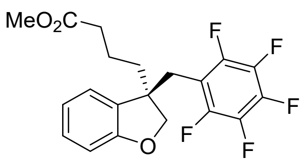

3h

Prepared according to typical procedure from allyl ether **1h** (108.1 mg, 0.3 mmol), after a flash column chromatography (hexanes: Et_2O = 20:1) afforded the product **3h** as a light yellow liquid (92.4 mg, 77% yield) with 90% *ee*. ^{1}H NMR (400 MHz, $CDCl_3$) δ 7.15-7.11 (m, 1 H), 6.95 (d, J = 7.7 Hz, 1 H), 6.88-6.83 (m, 1 H), 6.71 (dd, J = 8.2, 2.4 Hz, 1 H), 4.44 (dd, J = 9.2, 2.6 Hz, 1 H), 4.30 (dd, J = 9.2, 2.6 Hz, 1 H), 3.65 (d, J = 2.5 Hz, 3 H), 3.05 (q, J = 13.8 Hz, 2 H), 2.32-2.28 (m, 2 H), 1.76 (d, J = 6.7 Hz, 3 H), 1.49-1.40 (m, 1 H). ^{13}C NMR (101 MHz, $CDCl_3$) δ 173.50, 159.78, 146.68, 144.22, 141.20 (m, J_F = 49.2 Hz), 138.62 (d, J_F = 13.7 Hz), 136.05, 130.69, 129.02, 123.44, 120.64, 111.16 (m, J_F = 35.2 Hz), 109.78, 79.55 (t, J_F = 2.9 Hz), 51.57, 50.18, 36.71, 34.03, 31.64, 20.03. ^{19}F NMR (376 MHz, $CDCl_3$) δ -139.98 (dd, J = 22.5, 8.0 Hz), -155.76 (t, J = 20.8 Hz), -162.36 (td, J = 22.3, 8.0 Hz). HRMS (EI) calculated for $[C_{20}H_{17}F_5O_3]^+$: 400.1092 found: 400.1096. Enantiomeric excess was determined by HPLC with a Chiralpak OJH column (hexanes: 2-propanol = 95:5, 0.5 mL/min, 210 nm); minor enantiomer tr = 17.8 min, major enantiomer tr = 20.2 min. $[\alpha]_D^{20}$ = 14.2 (*c* = 0.3, $CHCl_3$).

7.9 Synthesis of (*R*)-3-(3-chloropropyl)-3-((perfluorophenyl)methyl)-2,3-dihydrobenzofuran (**3i**).

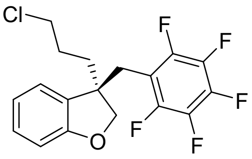

3i

Prepared according to typical procedure from allyl ether **1i** (101.0 mg, 0.3 mmol), after a flash column chromatography (hexanes: Et_2O = 100:1) afforded the product **3i** as a colorless liquid (101.0 mg, 87% yield) with 92% *ee*. ^{1}H NMR (400 M Hz, $CDCl_3$) δ 7.19–7.11 (m, 1 H), 6.99–6.95 (m, 1 H), 6.88 (td, J = 7.4, 1.0 Hz, 1 H), 6.75–6.72 (m, 1 H), 4.45 (d, J = 9.3 Hz, 1 H), 4.27 (d, J = 9.4 Hz, 1 H), 3.50 (t, J = 6.1 Hz, 2 H), 3.14–

3.02 (m, 2 H), 1.93–1.81 (m, 3 H), 1.64–1.55 (m, 1 H). ^{13}C NMR (101 MHz, $CDCl_3$) δ 159.76, 146.65, 144.20, 141.26 (m, J_F = 49.2 Hz), 138.58 (m, J_F = 79.7 Hz), 136.07, 130.42, 129.11, 123.37, 120.74, 110.99 (m, J_F = 37.4 Hz), 109.82, 79.46 (t, J_F = 2.9 Hz), 49.91, 44.92, 34.73, 31.83, 27.77. ^{19}F NMR (376 MHz, $CDCl_3$) δ -139.84 (dd, J = 22.7, 8.0 Hz), -155.51 (t, J = 20.9 Hz), -162.07– -162.23 (m). HRMS (EI) calculated for $[C_{18}H_{14}ClF_5O]^+$: 376.0648 found: 376.0646. Enantiomeric excess was determined by HPLC with a Chiralpak ADH column (hexanes: 2-propanol = 99:1, 0.5 mL/min, 210 nm); minor enantiomer tr = 13.6 min, major enantiomer tr = 15.4 min. $[\alpha]_D^{20}$ = 15.5 (*c* = 0.5, $CHCl_3$).

7.10 Synthesis of (*R*)-3-(4-chlorobutyl)-3-((perfluorophenyl)methyl)-2,3-dihydrobenzofuran (**3j**).

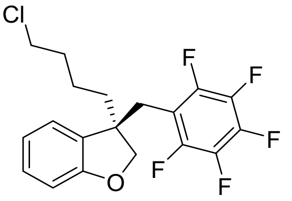

3j

Prepared according to typical procedure from allyl ether **1j** (105.2 mg, 0.3 mmol), after a flash column chromatography (hexanes: Et_2O = 100:1) afforded the product **3j** as a colorless liquid (83.3 mg, 71% yield) with 91% *ee*. ^{1}H NMR (400 MHz, $CDCl_3$) δ 7.17-7.12 (m, 1 H), 6.94 (dd, J = 7.5, 1.6 Hz, 1 H), 6.86 (t, J = 7.4 Hz, 1 H), 6.73 (d, J = 8.0 Hz, 1 H), 4.45 (d, J = 9.3 Hz, 1 H), 4.28 (d, J = 9.2 Hz, 1 H), 3.50 (td, J = 6.6, 2.0 Hz, 2 H), 3.12-3.00 (m, 2 H), 1.80-1.72 (m, 4 H), 1.61-1.50 (m, 1 H), 1.38-1.22 (m, 1 H). ^{13}C NMR (101 MHz, $CDCl_3$) δ 159.74, 146.65, 144.19, 141.19 (m, J_F = 49.2 Hz), 138.67 (d, J_F = 13.2 Hz), 136.04, 130.87, 128.99, 123.33, 120.63, 111.16(m, J_F = 35.3 Hz), 109.77, 79.66 (t, J_F = 2.8 Hz), 50.19, 44.50, 36.60, 32.81, 31.61, 21.86. ^{19}F NMR (376 MHz, $CDCl_3$) δ -139.98 (dd, J = 22.8, 7.9 Hz), -155.69 (t, J = 20.8 Hz), -162.11 – -162.34 (m). HRMS (EI) calculated for $[C_{19}H_{16}ClF_5O]^+$: 390.0804 found: 390.0805. Enantiomeric excess was determined by HPLC with a Chiralpak OZ3 column (hexanes: 2-propanol = 99.5:0.5, 0.3 mL/min, 210 nm); minor enantiomer tr = 28.5 min, major enantiomer tr = 30.1 min. $[\alpha]_D^{20}$ = 3.6 (*c* = 0.5, $CHCl_3$).

7.11 Synthesis of (*R*)-3-(5-fluoropentyl)-3-((perfluorophenyl)methyl)-2,3-dihydrobenzofuran (**3k**).

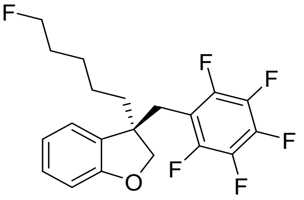

3k

Prepared according to typical procedure from allyl ether **1k** (104.5 mg, 0.3 mmol), after a flash column chromatography (hexanes: Et_2O = 100:1) afforded the product **3k** as a colorless liquid (72.0 mg, 62% yield) with 90% *ee*. ^{1}H NMR (400 MHz, $CDCl_3$) δ 7.17-7.12 (m, 1 H), 6.94 (d, J = 7.3 Hz, 1 H), 6.89-6.84 (m, 1 H), 6.73 (dd, J = 8.1, 3.1 Hz, 1 H), 4.49-4.43 (m, 2 H), 4.35 (t, J = 6.0 Hz, 1 H), 4.27 (dd, J = 9.3, 2.9 Hz, 1 H), 3.11-2.99 (m, 2 H), 1.77-1.60 (m, 4 H), 1.46-1.39 (m, 3 H), 1.21-1.11 (m, 1 H). ^{13}C NMR (101 MHz, $CDCl_3$) δ 159.76, 146.65, 144.22, 141.15 (m, J_F = 49.2 Hz), 138.55 (m, J_F = 79.7 Hz), 136.02, 131.09, 128.89, 123.36, 120.58, 111.27(m, J_F = 35.4 Hz), 109.70, 83.90 (d, J_F= 164.3 Hz), 79.73 (t, J_F = 2.8 Hz), 50.23, 37.31, 31.70, 30.19 (d, J_F = 19.6 Hz), 25.75 (d, J_F = 5.1 Hz), 24.15. ^{19}F NMR (376 MHz, $CDCl_3$) δ -140.01 (dd, J = 24.6, 8.2 Hz), -155.90 (t, J = 21.1 Hz), -162.43 (td, J = 23.5, 6.5 Hz), -218.42 (dd, J = 48.3, 23.9 Hz). HRMS (EI) calculated for $[C_{20}H_{18}F_6O]^+$: 388.1256 found: 388.1254. Enantiomeric excess was determined by HPLC with a Chiralpak OZ3 column (hexanes: 2-propanol = 99.5:0.5, 0.3 mL/min, 210 nm); minor enantiomer tr = 27.5 min, major enantiomer tr = 28.8 min. $[\alpha]_D^{20}$ = 5.3 (*c* = 0.5, $CHCl_3$).

7.12 Synthesis of (*R*)-3-(3-(4-chloro-3,5-dimethylphenoxy)propyl)-3-((perfluorophenyl)methyl)-2,3-dihydrobenzofuran (**3l**).

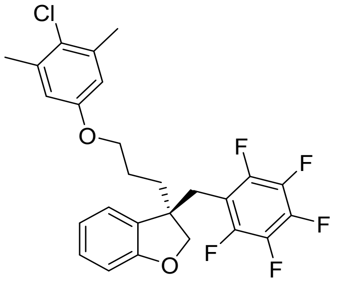

3l

Prepared according to typical procedure from allyl ether **1l** (137.0 mg, 0.3 mmol), after a flash column chromatography (hexanes: Et_2O = 10:1) afforded the product **3**l as a yellow liquid (152.8 mg, 84% yield) with 90% *ee*. ^{1}H NMR (400 MHz, $CDCl_3$) δ 7.19–7.14 (m, 1 H), 7.00–6.97 (m, 1 H), 6.91–6.86(m, 1 H), 6.76 (dd, J = 8.2, 3.5 Hz, 1 H), 6.60 (d, J = 3.4 Hz, 2 H), 4.48 (dd, J = 9.4, 3.6 Hz, 1 H), 4.33 (dd, J = 9.5, 3.4 Hz, 1 H), 3.89–3.86 (m, 2 H), 3.17-3.04 (m, 2 H), 2.34 (s, 6 H), 1.94–1.83 (m, 3 H), 1.64–1.57 (m, 1 H). ^{13}C NMR (101 MHz, $CDCl_3$) δ 159.80, 156.60, 146.64, 144.22, 141.15 (m, J_F = 49.2 Hz), 138.54 (m, J_F = 79.7 Hz), 137.06, 136.04, 130.71, 129.02, 126.22, 123.40, 120.66, 114.40, 111.12 (d, J_F = 3.5 Hz), 109.78, 79.60 (t, J_F = 2.9 Hz), 67.77, 50.04, 33.75, 31.71, 24.60, 20.86. ^{19}F NMR (376 MHz, $CDCl_3$) δ -139.82 (dd, J = 23.4, 8.2 Hz), -155.66 (dd, J = 23.7, 18.2 Hz), -162.24 (td, J = 22.2, 8.0 Hz). HRMS (EI) calculated for $[C_{26}H_{22}ClF_5O_2]^+$: 496.1223 found: 496.1224. Enantiomeric excess was determined by HPLC with a Chiralpak OZ3 column (hexanes: 2-propanol = 99:1, 0.3 mL/min, 210 nm); major enantiomer tr = 19.1 min, minor enantiomer tr = 25.4 min. $[\alpha]_D^{20}$ = 16.0 (*c* = 0.5, $CHCl_3$).

7.13 Synthesis of (*R*)-3-(4-(naphthalen-1-ylthio)butyl)-3-((perfluorophenyl)methyl)-2,3-dihydrobenzofuran (**3m**).

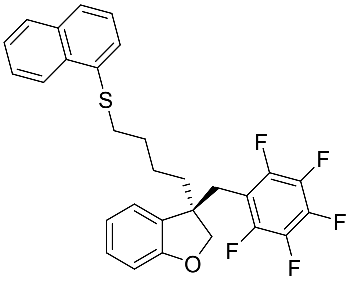

3m

Prepared according to typical procedure from allyl ether **1m** (142.3 mg, 0.3 mmol), after a flash column chromatography (hexanes: Et_2O = 50:1) afforded the product **3m** as a blue liquid (133.8 mg, 87% yield) with 91% *ee*. ^{1}H NMR (400 MHz, $CDCl_3$) δ 7.79–7.70 (m, 4 H), 7.52–7.38 (m, 3 H), 7.14 (td, J = 7.7, 1.5 Hz, 1 H), 6.92 (d, J = 6.6 Hz, 1 H), 6.85 (td, J = 7.4, 1.0 Hz, 1 H), 6.72 (d, J = 8.0 Hz, 1 H), 4.43 (d, J = 9.2 Hz, 1 H), 4.26 (d, J = 9.2 Hz, 1 H), 3.09–2.93 (m, 4 H), 1.75–1.64 (m, 4 H), 1.56–1.52 (m, 1 H), 1.34–1.24 (m, 1 H). ^{13}C NMR (101 MHz, $CDCl_3$) δ 159.81, 146.68, 144.23, 141.19 (m, J_F = 49.2 Hz), 138.57 (m, J_F = 79.7 Hz), 136.07, 134.10, 133.81, 131.76, 131.01, 128.96, 128.37, 127.72, 127.39, 127.01, 126.89, 126.56, 125.63, 123.40, 120.62, 111.26 (t, J_F = 18.3 Hz), 109.76, 79.72, 50.24, 36.96, 33.33, 31.67, 29.56, 23.74. ^{19}F NMR (376 MHz, $CDCl_3$) δ -139.91 (dt, J = 22.4, 6.9 Hz), -155.74 (t, J = 20.9 Hz), -162.28 (td, J = 22.1, 7.9 Hz). HRMS (EI) calculated for $[C_{29}H_{23}F_5OS]^+$: 514.1384 found: 514.1390. Enantiomeric excess was determined by HPLC with a Chiralpak IG column (hexanes: 2-propanol = 95:5, 0.5 mL/min, 254 nm); minor enantiomer tr = 14.4 min, major enantiomer tr = 15.4 min. $[\alpha]_D^{20}$ = 7.3 (*c* = 0.5, $CHCl_3$).

7.14 Synthesis of (*R*)-3-((perfluorophenyl)methyl)-3-(3-(p-tolylthio)propyl)-2,3-dihydrobenzofuran (**3n**).

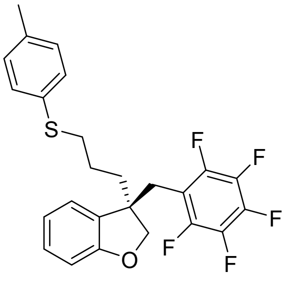

3n

Prepared according to typical procedure from allyl ether **1n** (127.3 mg, 0.3 mmol), after a flash column chromatography (hexanes: Et_2O = 50:1) afforded the product **3n** as a colorless liquid (116.5 mg, 84% yield) with 93% *ee*. ^{1}H NMR (400 MHz, $CDCl_3$) δ 7.21–7.18 (m, 2 H), 7.16–7.11 (m, 1 H), 7.09–7.07 (m, 2 H), 6.92 (dd, J = 7.4, 1.4 Hz, 1 H), 6.85 (td, J = 7.4, 1.0 Hz, 1 H), 6.71 (dd, J = 8.0, 0.9 Hz, 1 H), 4.41 (d, J = 9.3 Hz, 1 H), 4.22 (d, J = 9.3 Hz, 1 H), 3.10–2.98 (m, 2 H), 2.93–2.78 (m, 2 H), 2.32 (s, 3 H),

1.97–1.81 (m, 2 H), 1.76–1.65 (m, 1 H), 1.50–1.39 (m, 1 H). ^{13}C NMR (101 MHz, $CDCl_3$) δ 159.75, 146.66, 144.20, 141.18 (m, J_F = 49.2 Hz), 138.70 (m, J_F = 79.7 Hz), 136.30, 136.06, 132.35, 130.75, 130.16, 129.71, 129.00, 123.44, 120.67, 111.18(m, J_F = 35.2 Hz), 109.76, 79.60 (t, J_F = 2.9 Hz), 50.16, 36.25, 34.79, 31.80, 24.26, 20.99. ^{19}F NMR (376 MHz, $CDCl_3$) δ -139.87 (dd, J = 23.2, 8.2 Hz), -155.72 (t, J = 20.9 Hz), -162.27 (td, J = 22.3, 8.0 Hz). HRMS (EI) calculated for $[C_{25}H_{21}F_5OS]^+$: 464.1228 found: 464.1231. Enantiomeric excess was determined by HPLC with a Chiralpak OZ3 column (hexanes: 2-propanol = 80:20, 0.5 mL/min, 254 nm); major enantiomer tr = 8.4 min, minor enantiomer tr = 9.1 min. $[\alpha]_D^{20}$ = 2.4 (*c* = 0.5, $CHCl_3$).

7.15 Synthesis of (*R*)-3-(4-(3-((perfluorophenyl)methyl)-2,3-dihydrobenzofuran-3-yl)butyl)oxazolidin-2-one (**3o**).

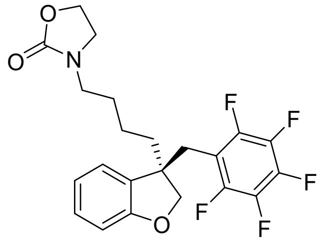

3o

Prepared according to typical procedure from allyl ether **1o** (120.3 mg, 0.3 mmol), after a flash column chromatography (hexanes: Et_2O = 10:1) afforded the product **3o** as a colorless liquid (115.1 mg, 87% yield) with 90% *ee*. ^{1}H NMR (400 MHz, $CDCl_3$) δ 7.12 (t, J = 7.7 Hz, 1 H), 6.92 (d, J = 7.4 Hz, 1 H), 6.84 (t, J = 7.4 Hz, 1 H), 6.70 (d, J = 8.0 Hz, 1 H), 4.41 (d, J = 9.3 Hz, 1 H), 4.28–4.23 (m, 3H), 3.52–3.45 (m, 2 H), 3.21 (t, J = 7.2 Hz, 2 H), 3.04 (q, J = 13.9 Hz, 2 H), 1.79 –1.72 (m, 2 H), 1.57-1.49 (m, 2 H), 1.45–1.34 (m, 1 H), 1.19–1.06 (m, 1 H). ^{13}C NMR (101 MHz, $CDCl_3$) δ 159.67, 158.38, 146.55, 144.11 (m, J_F = 49.2 Hz), 141.06, 138.44 (m, J_F = 79.7 Hz), 135.94, 130.79, 128.83, 123.31, 120.50, 111.37 – 110.90 (m, J_F = 53.6 Hz), 109.59, 79.54 (d, J_F = 3.0 Hz), 61.54, 50.09, 44.33, 43.66, 36.73, 31.51, 27.52, 21.33. ^{19}F NMR (376 MHz, $CDCl_3$) δ -139.98 (dd, J = 23.2, 8.0 Hz), -155.88 (t, J = 20.9 Hz), -162.42 (td, J = 22.2, 7.8 Hz). HRMS (ESI) calculated for $[M+H]^+$ = $[C_{22}H_{21}F_5NO_3]$+: 442.1437 found: 442.1442. Enantiomeric excess was determined by HPLC with a Chiralpak OJH

column (hexanes: 2-propanol = 60:40, 0.5 mL/min, 210 nm); minor enantiomer tr = 17.7 min, major enantiomer tr = 19.2 min. $[\alpha]_D^{20}$ = 7.1 (*c* = 0.5, $CHCl_3$).

7.16 Synthesis of (*R*)-1-(4-(3-((perfluorophenyl)methyl)-2,3-dihydrobenzofuran-3-yl)butyl)pyrrolidine-2,5-dione (**3p**).

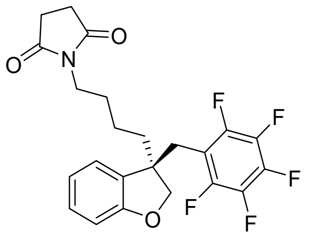

3p

Prepared according to typical procedure from allyl ether **1p** (124.0 mg, 0.3 mmol), after a flash column chromatography (hexanes: Et_2O = 10:1) afforded the product **3p** as a colorless liquid (112.0 mg, 85% yield) with 91% *ee*. ^1H NMR (400 MHz, $CDCl_3$) δ 7.14-7.10 (m, 1 H), 6.94-6.92 (m, 1 H), 6.85 (td, J = 7.4, 1.0 Hz, 1 H), 6.71 (dt, J = 8.0, 0.7 Hz, 1 H), 4.41 (d, J = 9.2 Hz, 1 H), 4.24 (d, J = 9.3 Hz, 1 H), 3.47-3.43 (m, 2 H), 3.09-2.98 (m, 2 H), 2.66 (s, 4 H), 1.78-1.70 (m, 2 H), 1.59 -1.52 (m, 2 H), 1.41-1.30 (m, 1 H), 1.14-1.01 (m, 1 H).^{13}C NMR (101 MHz, $CDCl_3$) δ 177.11, 159.72, 146.60, 144.15, 141.11 (m, J_F = 49.2 Hz), 138.47 (m, J_F = 79.7 Hz), 135.92 (d, J_F = 14.3 Hz), 130.76, 128.88, 123.37, 120.52, 111.97 – 110.88 (m, J_F = 109.2 Hz), 109.64, 79.54 (t, J_F = 2.7 Hz), 60.31, 50.12, 38.23, 36.75, 31.62, 27.98 (d, J_F = 13.1 Hz), 21.59. ^{19}F NMR (376 MHz, $CDCl_3$) δ -139.96 (dd, J = 22.8, 8.0 Hz), -155.81 (t, J = 21.0 Hz), -162.27 – -162.42 (m). HRMS (ESI) calculated for $[M+H]^+$ = $[C_{23}H_{21}F_5NO_3]^+$: 454.1437 found: 454.1442. Enantiomeric excess was determined by HPLC with a Chiralpak ODH column (hexanes: 2-propanol = 60:40, 0.5 mL/min, 210 nm); minor enantiomer tr = 26.8 min, major enantiomer tr = 29.7 min. $[\alpha]_D^{20}$ = 6.2 (*c* = 0.4, $CHCl_3$).

7.17 Synthesis of (*R*)-2-(3-(3-((perfluorophenyl)methyl)-2,3-dihydrobenzofuran-3-yl)propyl)isoindoline-1,3-dione (**3q**).

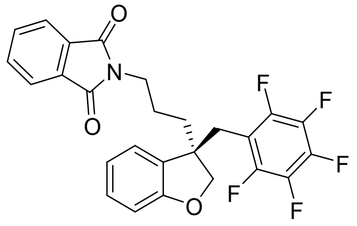

3q

Prepared according to typical procedure from allyl ether **1q** (134.2 mg, 0.3 mmol), after a flash column chromatography (hexanes: Et_2O = 10:1) afforded the product **3q** as a white solid (112.3 mg, 77% yield) with 90% *ee*. ^{1}H NMR (400 MHz, $CDCl_3$) δ 7.85–7.78(m, 2 H), 7.73–7.69 (m, 2 H), 7.11 (td, J = 7.7, 1.4 Hz, 1 H), 6.93 (d, J = 7.4 Hz, 1 H), 6.83 (td, J = 7.4, 1.0 Hz, 1 H), 6.70 (s, 1 H), 4.41 (d, J = 9.3 Hz, 1 H), 4.24 (d, J = 9.3 Hz, 1 H), 3.72–3.63 (m, 2 H), 3.10–2.98 (m, 2 H), 1.84–1.72 (m, 3 H), 1.57–1.47 (m, 1 H). ^{13}C NMR (101 MHz, $CDCl_3$) δ 168.31, 159.71, 146.65, 144.20, 141.22 (m, J_F = 49.2 Hz), 138.56 (m, J_F = 79.7 Hz), 136.04, 133.99, 132.02, 130.55, 129.07, 123.43, 123.34, 120.74, 111.05 (m, J_F = 36.3 Hz), 109.81, 79.64(t, J_F = 2.9 Hz), 49.95, 37.99, 34.46, 31.55, 23.88. ^{19}F NMR (376 MHz, $CDCl_3$) δ -139.90 (dd, J = 23.3, 8.2 Hz), -155.64 (t, J = 20.8 Hz), -162.25 (td, J = 22.2, 8.1 Hz). HRMS (EI) calculated for $[C_{26}H_{18}F_5NO_3]^+$: 487.1201 found: 487.1204. Enantiomeric excess was determined by HPLC with a Chiralpak OZ3 column (hexanes: 2-propanol = 80:20, 0.5 mL/min, 210 nm); major enantiomer tr = 18.7 min, minor enantiomer tr = 20.7 min. $[\alpha]_D^{20}$ = 6.1 (*c* = 0.5, $CHCl_3$).

7.18 Synthesis of (*R*)-1-(4-(3-((perfluorophenyl)methyl)-2,3-dihydrobenzofuran-3-yl)butyl)benzo[cd]indol-2(1H)-one (**3r**).

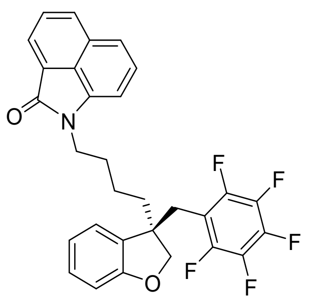

3r

Prepared according to typical procedure from allyl ether **1r** (145.0 mg, 0.3 mmol), after

a flash column chromatography (hexanes: Et_2O = 10:1) afforded the product **3r** as a light yellow liquid (136.6 mg, 87% yield) with 92% *ee*. ^{1}H NMR (400 MHz, $CDCl_3$) δ 8.02 (dd, J = 19.7, 7.5 Hz, 2 H), 7.70 (dd, J = 8.1, 7.0 Hz, 1 H), 7.52 (d, J = 8.4 Hz, 1 H), 7.43 (dd, J = 8.5, 7.0 Hz, 1 H), 7.10 (td, J = 7.7, 1.4 Hz, 1 H), 6.93 (dd, J = 7.5, 1.3 Hz, 1 H), 6.85–6.77 (m, 2 H), 6.69 (d, J = 8.0 Hz, 1 H), 4.40 (d, J = 9.3 Hz, 1 H), 4.25 (d, J = 9.3 Hz, 1 H), 3.88 (t, J = 7.2 Hz, 2 H), 3.06 (dt, J = 13.8, 1.8 Hz, 2 H), 1.86–1.71 (m, 4 H), 1.56–1.44 (m, 1 H), 1.34–1.17 (m, 1 H). ^{13}C NMR (101 MHz, $CDCl_3$) δ 167.99, 159.77, 146.64, 144.20, 141.16 (m, J_F = 49.2 Hz), 139.34, 138.57 (m, J_F = 79.7 Hz), 136.02, 130.90, 130.77, 129.13, 128.91, 128.68, 128.44, 126.63, 125.15, 124.24, 123.44, 120.59, 120.24, 111.24 (t, J_F = 18.2 Hz), 109.69, 104.87, 79.64 (t, J_F = 2.8 Hz), 50.22, 39.81, 37.07, 31.69, 29.09, 21.84. ^{19}F NMR (376 MHz, $CDCl_3$) δ -139.89 (dd, J = 23.7, 8.0 Hz), -155.81 (t, J = 20.9 Hz), -162.26 – -162.41 (m, J = 27.1, 16.1, 6.1 Hz). HRMS (EI) calculated for $[C_{30}H_{22}F_5NO_2]^+$: 523.1565 found: 523.1567. Enantiomeric excess was determined by HPLC with a Chiralpak ODH column (hexanes: 2-propanol = 80:20, 0.5 mL/min, 254 nm); minor enantiomer tr = 20.5 min, major enantiomer tr = 22.0 min. $[\alpha]_D^{20}$ = 11.6 (*c* = 0.5, $CHCl_3$).

7.19 Synthesis of (*3R*)-3-(3-(((*4R,6S*)-6-(2,2-dimethyl-1,3-dioxolan-4-yl)-2,2-dimethyltetrahydrofuro[3,4-d][1,3]dioxol-4-yl)oxy)propyl)-3-((perfluorophenyl)methyl)-2,3-dihydrobenzofura (**3s**).

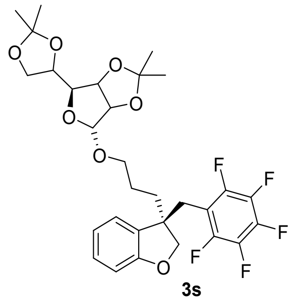

3s

Prepared according to typical procedure from allyl ether **1s** (168.0 mg, 0.3 mmol), after a flash column chromatography (hexanes: Et_2O = 50:1) afforded the product **4s** as a colorless liquid (118.1 mg, 81% yield) with 18:1 dr. ^{1}H NMR (400 MHz, $CDCl_3$) δ 7.15–7.09 (m, 1 H), 6.95 (d, J = 7.4 Hz, 1 H), 6.87–6.83 (m, 1 H), 6.71 (d, J = 8.0 Hz,

1 H), 4.92 (s, 1 H), 4.76 (dd, J = 5.9, 3.6 Hz, 1 H), 4.54 (d, J = 5.9 Hz, 1 H), 4.44–4.33 (m, 2 H), 4.26 (d, J = 9.3 Hz, 1 H), 4.09 (dd, J = 8.7, 6.3 Hz, 1 H), 4.00 (dd, J = 8.7, 4.5 Hz, 1 H), 3.88 (dd, J = 7.6, 3.6 Hz, 1 H), 3.58 (dt, J = 9.8, 6.3 Hz, 1 H), 3.33 (dt, J = 9.7, 6.3 Hz, 1 H), 3.11–2.99 (m, 2 H), 1.76 (q, J = 7.9 Hz, 2 H), 1.69–1.60 (m, 2 H), 1.45 (d, J = 3.4 Hz, 6 H), 1.37 (s, 3 H), 1.31 (s, 3 H). ^{13}C NMR (101 MHz, $CDCl_3$) δ 159.74, 146.63, 144.19, 141.18 (m, J_F = 49.2 Hz), 138.60 (m, J_F = 79.7 Hz), 136.02, 130.76, 128.95, 123.39, 120.61, 112.58, 111.16 (t, J = 19.0 Hz), 109.73, 109.17, 106.26, 84.99, 80.27, 79.61, 79.49 (d, J = 4.1 Hz), 73.10, 67.21, 66.86, 49.98, 33.98, 31.58, 26.84, 25.83, 25.12, 24.65, 24.46. ^{19}F NMR (376 MHz, $CDCl_3$) δ -139.89 (dd, J = 23.3, 8.2 Hz), -155.72 (t, J = 20.8 Hz), -162.31 (td, J = 22.0, 7.9 Hz). HRMS (ESI) calculated for $[M+H]^+ = [C_{30}H_{34}F_5O_7]^+$: 601.2220 found: 601.2225. $[\alpha]_D^{20}$ = 46.7 (*c* = 0.3, $CHCl_3$).

7.20 Synthesis of (*R*)-3-(3-((*R*)-3-((perfluorophenyl)methyl)-2,3-dihydrobenzofuran-3-yl)propyl)-4-phenyloxazolidin-2-one (**3t**).

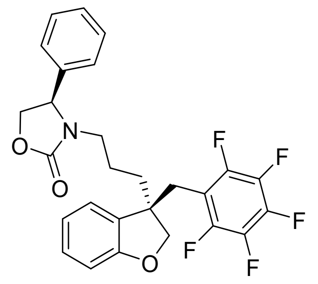

3t

Prepared according to typical procedure from allyl ether **1t** (139.0 mg, 0.3 mmol), after a flash column chromatography (hexanes: Et_2O = 2:1) afforded the product **4t** as a white solid (124.7 mg, 83% yield) with >20:1 dr. Mp: 161-162 °C. ^{1}H NMR (400 MHz, $CDCl^3$) δ 7.39 (dd, J = 5.2, 1.9 Hz, 3 H), 7.20 (dd, J = 6.8, 2.7 Hz, 2 H), 7.16-7.11 (m, 1 H), 6.85 (d, J = 6.7 Hz, 2 H), 6.71 (d, J = 8.0 Hz, 1 H), 4.64–4.54 (m, 2 H), 4.37 (d, J = 9.2 Hz, 1 H), 4.16 (d, J = 9.3 Hz, 1 H), 4.10 (dd, J = 8.2, 6.3 Hz, 1 H), 3.39-3.32 (m, 1 H), 3.00 (q, J = 13.9 Hz, 2 H), 2.78-2.71 (m, 1 H), 1.71-1.56 (m, 2 H), 1.52-1.44 (m, 1 H), 1.33-1.21 (m, 1 H). ^{13}C NMR (101 MHz, $CDCl^3$) δ 159.70, 158.18, 146.57, 144.14, 141.16 (m, J_F = 49.2 Hz), 138.61 (d, J_F = 15.1 Hz), 137.61, 136.02, 130.41, 129.29, 129.15, 129.01, 127.02, 123.34, 120.63, 110.94, 109.75, 79.44 (t, J_F = 2.8 Hz),

69.72, 59.89, 49.97, 42.38, 34.37, 31.49, 22.22. ^{19}F NMR (376 MHz, CDCl^3) δ -139.88 (dd, J = 23.0, 8.1 Hz), -155.49 (t, J = 21.0 Hz), -162.10 (td, J = 22.4, 7.9 Hz). HRMS (ESI) calculated for $[M+H]^+$ = $[C_{27}H_{23}F_5NO_3]^+$: 504.1593 found: 504.1598. $[\alpha]_D^{20}$ = 44.637 (*c* = 0.5, $CHCl_3$).

7.21 Synthesis of (*S*)-2,5,7,8-tetramethyl-6-(3-((*R*)-3-((perfluorophenyl)methyl)-2,3-dihydrobenzofuran-3-yl)propoxy)-2-((*4S*,*8S*)-4,8,12-trimethyltridecyl)chromane (**3u**).

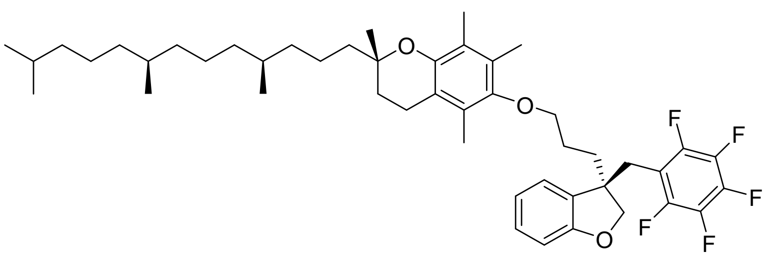

3u

Prepared according to typical procedure from allyl ether **1v** (219.3 mg, 0.3 mmol), after a flash column chromatography (hexanes: Et_2O = 40:1) afforded the product **3u** as a yellow liquid (184.1 mg, 80% yield) with >20:1 dr. ^{1}H NMR (400 MHz, CDCl3) δ 7.18 (td, J = 7.7, 1.4 Hz, 1 H), 7.05 (d, J = 7.4 Hz, 1 H), 6.91 (t, J = 7.4 Hz, 1 H), 6.78 (d, J = 8.0 Hz, 1 H), 4.52 (d, J = 9.3 Hz, 1 H), 4.39 (d, J = 9.3 Hz, 1 H), 3.62 (q, J = 5.8 Hz, 2 H), 3.21–3.08 (m, 2 H), 2.59 (t, J = 6.8 Hz, 2 H), 2.15 (s, 3 H), 2.10 (s, 6 H), 2.06–2.02 (m, 2 H), 1.94-1.72 (m, 3 H), 1.64–1.50 (m, 5 H), 1.44–1.28 (m, 11 H), 1.22–1.05 (m, 8 H), 0.98–0.87 (m, 13 H). ^{13}C NMR (101 MHz, CDCl3) δ 159.89, 147.99, 147.77, 146.69, 144.25, 141.22 (m, J_F = 49.2 Hz), 138.63 (m, J_F = 79.7 Hz), 136.09, 130.89, 128.94, 127.67, 125.65, 123.45, 122.86, 120.66, 117.51, 111.29(m, J_F = 35.2 Hz), 109.74, 79.61(t, J_F = 2.9 Hz), 74.75, 72.47, 50.16, 40.03, 39.37, 37.47, 37.45, 37.41, 37.28, 34.11, 32.79, 32.69, 32.11, 31.29, 27.97, 25.53, 24.79, 24.43, 23.84, 22.70, 22.61, 21.01, 20.63, 19.73, 19.64, 12.60, 11.74. ^{19}F NMR (376 MHz, CDCl3) δ -139.73 (dd, J = 22.9, 8.1 Hz), -155.76 (t, J = 21.0 Hz), -162.32 (td, J = 22.4, 7.9 Hz). HRMS (ESI) calculated for $[M+H]^+$ = $[C_{47}H_{64}F_5O_3]^+$: 771.4770 found: 771.4791. $[\alpha]_D^{20}$ = 15.719 (*c* = 0.5, $CHCl_3$).

7.22 Synthesis of (*S*)-5-chloro-3'-methyl-2H-spiro[benzofuran-3,7'-bicyclo[4.2.0] octane]-1'(6'),2',4'-triene (**5a**).

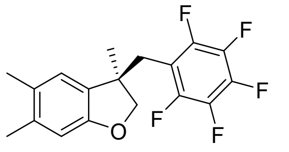

5a

Prepared according to typical procedure from allyl ether **4a** (90.7 mg, 0.3 mmol), after a flash column chromatography (hexanes: Et_2O = 100:1) afforded the product **5a** as a colorless liquid (71.7 mg, 70% yield) with 93% *ee*. ^{1}H NMR (400 MHz, $CDCl_3$) δ 6.77 (s, 1 H), 6.59 (s, 1 H), 4.44 (d, J = 8.9 Hz, 1 H), 4.05 (d, J = 8.9 Hz, 1 H), 3.06 – 2.91 (m, 2 H), 2.22 (s, 3 H), 2.19 (s, 3 H), 1.36 (d, J = 1.6 Hz, 3 H). ^{13}C NMR (101 MHz, $CDCl_3$) δ 157.57, 146.66, 144.19, 138.62, 137.12, 136.10, 130.55, 128.59, 123.58, 111.66(m, J_F = 36.3 Hz), 111.00,107.14, 82.14 (t, J_F = 2.8 Hz), 46.64, 32.68, 23.90, 20.14, 19.28. ^{19}F NMR (376 MHz, $CDCl_3$) δ -140.24 (dd, J = 23.0, 8.4 Hz), -156.09 (t, J = 20.8 Hz), -162.45 (td, J = 22.2, 8.0 Hz). HRMS (EI) calculated for $[C_{18}H_{15}F_5O]^+$: 342.1038 found: 342.1037. Enantiomeric excess was determined by HPLC with a Chiralpak OZ3 column (hexanes = 100, 1.0 mL/min, 210 nm); minor enantiomer tr = 20.5 min, major enantiomer tr = 31.1 min. $[\alpha]_D^{20}$ = -23.4 (*c* = 0.3, $CHCl_3$).

7.23 Synthesis of (*R*)-5-fluoro-3-methyl-3-((perfluorophenyl)methyl)-2,3-dihydrobenzofuran (**5b**).

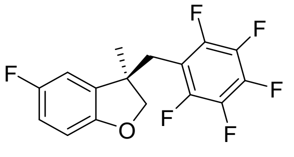

5b

Prepared according to typical procedure from allyl ether **4b** (87.6 mg, 0.3 mmol), after a flash column chromatography (hexanes: Et_2O = 100:1) afforded the product **5b** as a white solid (58.6 mg, 60% yield) with 87% *ee*. Mp: 80-82 ^{o}C. ^{1}H NMR (400 MHz, $CDCl_3$) δ 6.83 (td, J = 8.8, 2.7 Hz, 1 H), 6.72-6.65 (m, 2 H), 4.50 (d, J = 9.0 Hz, 1 H), 4.13 (d, J = 9.0 Hz, 1 H), 3.05-2.95 (m, 2 H), 1.40 (t, J = 1.2 Hz, 3 H). ^{13}C NMR (101 MHz, $CDCl_3$) δ 158.84, 156.48, 155.22, 146.58 (d, J_F = 10.5 Hz), 144.13 (d, J_F = 9.2

Hz), 141.31, 138.72 (d, J_F = 15.0 Hz), 136.05 (d, J_F = 16.3 Hz), 134.56 (d, J_F = 7.7 Hz), 115.03 (d, J_F = 24.1 Hz), 111.03 (t, J_F = 17.1 Hz), 110.24-109.91 (m, J_F = 33.1 Hz), 82.59 (t, J_F = 2.5 Hz), 47.04, 32.62, 23.61. ^{19}F NMR (376 MHz, $CDCl_3$) δ -123.49 (d, J = 8.2 Hz), -140.42 (dd, J = 23.1, 8.3 Hz), -155.48 (t, J = 21.1 Hz), -162.03 – -162.17 (m). HRMS (EI) calculated for $[C_{16}H_{10}F_6O]^+$: 332.0630 found: 332.0628. Enantiomeric excess was determined by HPLC with a Chiralpak IA column (hexanes = 100, 1.0 mL/min, 210 nm); major enantiomer tr = 15.7 min, minor enantiomer tr = 22.4 min. $[\alpha]_D^{20}$ = -15.5 (*c* = 0.5, $CHCl_3$).

7.24 Synthesis of methyl (*R*)-3-methyl-3-((perfluorophenyl)methyl)-2,3-dihydronaphtho[2,3-b]furan (**5c**).

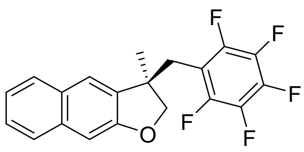

5c

Prepared according to typical procedure from allyl ether **4c** (97.3 mg, 0.3 mmol), after a flash column chromatography (hexanes: Et_2O = 100:1) afforded the product **5c** as a white solid (86.1 mg, 79% yield) with 90% *ee*. Mp: 151-152 °C. ^{1}H NMR (400 MHz, CDCl3) δ 7.74-7.69 (m, 2 H), 7.45-7.39 (m, 2 H), 7.32 (t, J = 7.6 Hz, 1 H), 7.11 (s, 1 H), 4.56 (d, J = 8.9 Hz, 1 H), 4.18 (d, J = 8.9 Hz, 1 H), 3.11 (q, J = 13.8 Hz, 2 H), 1.48 (s, 3 H). ^{13}C NMR (101 MHz, CDCl3) δ 157.88, 146.75, 144.20, 141.28, 138.65, 136.00, 136.16, 134.65, 129.55, 127.78, 126.85, 126.10, 123.49, 121.81, 111.12 (m, J_F = 33.6 Hz), 104.47, 82.21 (t, J_F = 2.8 Hz), 46.27, 32.72, 23.71. ^{19}F NMR (376 MHz, CDCl3) δ -140.14 (dd, J = 22.6, 8.3 Hz), -155.53 (t, J = 20.9 Hz), -162.03 (td, J = 22.4, 8.0 Hz). HRMS (EI) calculated for $[C_{20}H_{13}F_5O]^+$: 364.0881 found: 364.0876. Enantiomeric excess was determined by HPLC with a Chiralpak IB column (hexanes: 2-propanol = 99:1, 1.0 mL/min, 210 nm); minor enantiomer tr = 6.5 min, major enantiomer tr = 7.6 min. $[\alpha]_D^{20}$ = 40.8 (*c* = 0.3, $CHCl_3$).

7.25 Synthesis of (*R*)-(3-methyl-3-((perfluorophenyl)methyl)-2,3-

dihydrobenzofuran-6-yl)(piperidin-1-yl)methanone (**5d**).

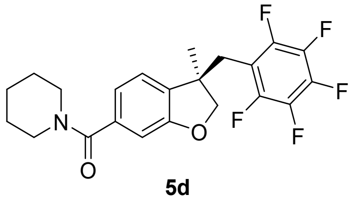

5d

Prepared according to typical procedure from allyl ether **1y** (115.3 mg, 0.3 mmol), after a flash column chromatography (hexanes: Et_2O = 10:1) afforded the product **4y** as a colorless liquid (109.3 mg, 86% yield) with 87% *ee*. ^{1}H NMR (400 MHz, $CDCl_3$) δ 7.00 (d, J = 7.6 Hz, 1 H), 6.89 (dd, J = 7.6, 1.4 Hz, 1 H), 6.76 (d, J = 1.3 Hz, 1 H), 4.53 (d, J = 9.1 Hz, 1 H), 4.14 (d, J = 9.1 Hz, 1 H), 3.68 (s, 2 H), 3.30 (s, 2 H), 3.09-2.92 (m, 2 H), 1.70–1.52 (m, 6 H), 1.42 (s, 3 H). ^{13}C NMR (101 MHz, $CDCl_3$) δ 169.80, 159.29, 146.55, 144.10, 141.15, 138.50, 137.51, 136.00, 134.20, 122.87, 119.20, 111.13(m, J_F = 35.4 Hz), 108.22, 82.34 (d, J_F = 2.6 Hz), 48.58, 46.59, 43.01, 32.81, 26.39, 25.57, 24.51, 23.51. ^{19}F NMR (376 MHz, $CDCl_3$) δ -140.31 (dd, J = 22.6, 8.0 Hz), -155.72 (td, J = 20.8, 11.0 Hz), -162.42 (td, J = 22.1, 10.5 Hz). HRMS (ESI) calculated for $[M+H]^+$ = $[C_{22}H_{21}F_5NO_2]^+$: 426.1487 found: 426.1492. Enantiomeric excess was determined by HPLC with a Chiralpak IA column (hexanes: 2-propanol = 70:30, 0.5 mL/min, 210 nm); major enantiomer tr = 16.8 min, minor enantiomer tr = 22.8 min. $[\alpha]_D^{20}$ = -35.4 (*c* = 0.4, $CHCl_3$).

7.26 Synthesis of tert-butyl (*R*)-5-methoxy-3-methyl-3-((perfluorophenyl)methyl)indoline-1-carboxylate (**5e**).

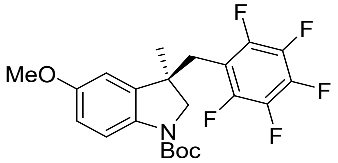

5e

Prepared according to typical procedure from allyl ether **4e** (121.0 mg, 0.3 mmol), after a flash column chromatography (hexanes: Et_2O = 10:1) afforded the product **5e** as a yellow liquid (111.4 mg, 84% yield) with 82% *ee*. ^{1}H NMR (400 MHz, $CDCl_3$) δ 7.66-7.28 (m, 1 H), 6.72 (dd, J = 8.8, 2.6 Hz, 1 H), 6.62 (d, J = 27.6 Hz, 1 H), 4.02 (d, J = 11.4 Hz, 1 H), 3.75 (s, 3 H), 3.54 (d, J = 20.4 Hz, 1 H), 2.92 (d, J = 6.1 Hz, 2 H), 1.53

(s, 9 H), 1.37 (d, J = 1.4 Hz, 3 H). ^{13}C NMR (101 MHz, $CDCl_3$) δ 155.60, 152.02, 146.61, 144.16, 141.07, 138.50 (dd, J_F = 17.3, 10.9 Hz), 138.07, 135.98 (t, J_F = 15.6 Hz), 135.51, 115.27, 112.87, 111.91-110.33 (m, J_F = 158.2 Hz), 109.10, 80.34, 59.85 (d, J = 2.7 Hz), 55.63, 44.22, 33.59, 28.24, 24.16. ^{19}F NMR (376 MHz, $CDCl_3$) δ -139.91 – -140.39 (m), -155.83 – -156.13 (m), -162.52 (td, J = 22.1, 8.1 Hz). HRMS (EI) calculated for $[C_{22}H_{22}F_5NO_3]^+$: 443.1514 found: 443.1517. Enantiomeric excess was determined by HPLC with a Chiralpak ASH column (hexanes: 2-propanol = 99:1, 0.3 mL/min, 210 nm); major enantiomer tr = 21.7 min, minor enantiomer tr = 24.0 min. $[\alpha]_D^{20}$ = -12.5 (*c* = 0.5, $CHCl_3$).

7.27 Synthesis of (*R*)-1-methyl-1-((perfluorophenyl)methyl)-2,3-dihydro-1H-indene (**5f**).

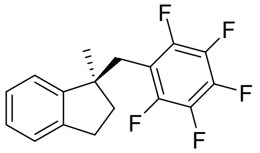

5f

Prepared according to typical procedure from allyl **4f** (81.6mg, 0.3 mmol), after a flash column chromatography (hexanes = 1) afforded the product **5f** as a colorless liquid (57.9 mg, 62% yield) with 88 % *ee*. ^{1}H NMR (400 MHz, $CDCl_3$) δ 7.25-7.16 (m, 3 H), 7.06-7.02 (m, 1 H), 2.99-2.83 (m, 4 H), 2.20-2.11 (m, 1 H), 1.84 (dt, J = 12.8, 8.5 Hz, 1 H), 1.32 (t, J = 1.7 Hz, 3 H). ^{13}C NMR (101 MHz, $CDCl_3$) δ 149.46, 146.72, 144.29, 142.97, 140.88, 138.57, 136.08, 127.02, 126.35, 124.71, 122.38, 112.73 (d, J_F = 19.3 Hz), 48.73, 39.03, 33.02, 29.95, 25.57. ^{19}F NMR (376 MHz, $CDCl_3$) δ -140.38 (dd, J = 22.8, 8.2 Hz), -156.96 (d, J = 20.7 Hz), -162.90 (dd, J = 30.9, 21.5 Hz). HRMS (EI) calculated for $[C_{17}H_{13}F_5]^+$: 312.0932 found: 312.0935. Enantiomeric excess was determined by HPLC with a Chiralpak IE column (hexanes = 100, 0.3 mL/min, 210 nm); major enantiomer tr = 17.5 min, minor enantiomer tr = 19.8 min. $[\alpha]_D^{20}$ = -4.5 (*c* = 0.5, $CHCl_3$).

7.28 Synthesis of (*R*)-3-methyl-3-(2,3,5,6-tetrafluorobenzyl)-2,3-

dihydrobenzofuran (**7a**).

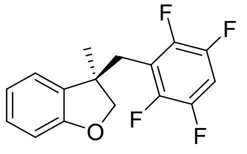

7a

Prepared according to typical procedure from **6a** (135.1 mg, 0.9 mmol), after a flash column chromatography (hexanes: Et_2O = 100:1) afforded the product **6a** as a colorless liquid (59.4 mg, 67% yield) with 91% *ee*. ^{1}H NMR (400 MHz, $CDCl_3$) δ 7.16 (td, J = 7.7, 1.4 Hz, 1 H), 7.03 (dd, J = 7.5, 1.4 Hz, 1 H), 6.99-6.94 (m, J = 9.7, 7.3, 2.3 Hz, 1 H), 6.92 – 6.87 (m, 1 H), 6.79 (d, J = 8.0 Hz, 1 H), 4.53 (d, J = 8.9 Hz, 1 H), 4.12 (d, J = 9.0 Hz, 1 H), 3.13-3.01 (m, J = 13.5, 2.0 Hz, 2 H), 1.41 (d, J = 1.8 Hz, 3 H). ^{13}C NMR (101 MHz, $CDCl_3$) δ 159.31, 147.57- 145.79 (dm, J_F = 21.2 Hz), 145.04 – 143.33 (dm, J_F = 171.3 Hz), 133.45, 128.70, 122.81, 120.67, 117.42 (t, J_F = 18.3 Hz), 109.85, 104.53 (t, J_F = 22.7 Hz), 82.19 (t, J_F = 2.6 Hz), 46.72, 33.21 (d, J_F = 2.1 Hz), 23.84 (d, J_F = 2.1 Hz). ^{19}F NMR (376 MHz, $CDCl_3$) δ -139.34 – -139.55 (m), -140.87 – -140.99 (m). HRMS (EI) calculated for $[C_{16}H_{12}F_4O]^+$: 296.0819 found: 296.0819. Enantiomeric excess was determined by HPLC with a Chiralpak OZ3 column (hexanes = 100, 1.0 mL/min, 210 nm); minor enantiomer tr = 26.8 min, major enantiomer tr = 32.2 min. $[\alpha]_D^{20}$ = 31.0 (*c* = 0.5, $CHCl_3$).

7.29 Synthesis of (*R*)-2,3,5,6-tetrafluoro-4-((3-methyl-2,3-dihydrobenzofuran-3-yl)methyl)pyridine (**7b**).

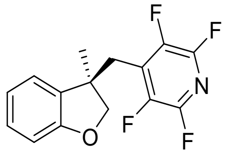

7b

Prepared according to typical procedure from **6b** (136.0 mg, 0.9 mmol), after a flash column chromatography (hexanes: Et_2O = 50:1) afforded the product **7b** as a white solid (62.7 mg, 70% yield) with 90% *ee*. Mp: 80-82 ^{o}C. ^{1}H NMR (400 MHz, $CDCl^3$) δ 7.17 (td, J = 7.7, 1.4 Hz, 1 H), 7.01 (d, J = 7.4 Hz, 1 H), 6.89 (t, J = 7.4 Hz, 1 H), 6.77 (d, J = 8.0 Hz, 1 H), 4.53 (d, J = 9.1 Hz, 1 H), 4.13 (d, J = 9.1 Hz, 1 H), 3.18-3.06 (m,

2 H), 1.47 (s, 3 H). ^{13}C NMR (101 MHz, $CDCl^3$) δ 159.23, 143.44 (d, J_F = 258.4 Hz), 139.31, 132.33, 131.17 (d, J_F = 17.4 Hz), 129.13, 122.71, 120.92, 110.06, 82.06 (t, J_F = 2.5 Hz), 46.85, 34.19, 23.73. ^{19}F NMR (376 MHz, $CDCl^3$) δ -91.27 – -91.45 (m), -141.91 – -142.09 (m). HRMS (EI) calculated for $[C_{15}H_{11}F_4NO]^+$: 297.0771 found: 297.0773. Enantiomeric excess was determined by HPLC with a Chiralpak IA column (hexanes: 2-propanol = 95:5, 0.5 mL/min, 210 nm); minor enantiomer tr = 12.1 min, major enantiomer tr = 15.9 min. $[\alpha]_D^{20}$ = -13.3 (*c* = 0.4, $CHCl_3$).

7.30 Synthesis of (*R*)-3-methyl-3-(2,3,5,6-tetrafluoro-4-methoxybenzyl)-2,3-dihydrobenzofuran (**7c**).

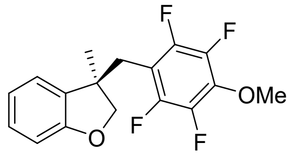

7c

Prepared according to typical procedure from **6c** (162.1 mg, 0.9 mmol), after a flash column chromatography (hexanes: Et_2O = 50:1) afforded the product **7c** as a colorless liquid (80.0 mg, 82% yield) with 88% *ee*. ^1H NMR (400 MHz, $CDCl_3$) δ 7.16 (td, J = 7.6, 1.4 Hz, 1 H), 7.03 (dd, J = 7.4, 1.4 Hz, 1 H), 6.89 (td, J = 7.4, 1.0 Hz, 1 H), 6.79 (dd, J = 8.0, 0.9 Hz, 1 H), 4.51 (d, J = 8.9 Hz, 1 H), 4.11 (d, J = 8.9 Hz, 1 H), 4.06 (t, J = 1.3 Hz, 3 H), 3.05-2.95 (m, 2 H), 1.39 (d, J = 1.3 Hz, 3 H). ^{13}C NMR (101 MHz, $CDCl_3$) δ 159.31, 146.77, 144.30, 141.84, 139.39, 133.59, 128.63 (d, J_F = 2.8 Hz), 122.82, 120.63, 109.80, 82.15 (d, J_F = 2.9 Hz), 62.04 (t, J_F = 3.6 Hz), 46.67, 32.62, 23.88.^{19}F NMR (376 MHz, $CDCl_3$) δ -142.03 – -142.37 (m), -158.21 – -158.55 (m). HRMS (EI) calculated for $[C_{17}H_{14}F_4O_2]^+$: 326.0924 found: 326.0929. Enantiomeric excess was determined by HPLC with a Chiralpak IC column (hexanes: 2-propanol = 99:1, 0.3 mL/min, 254 nm); minor enantiomer tr = 15.9 min, major enantiomer tr = 17.6 min. $[\alpha]_D^{20}$ = -20.1 (*c* = 0.5, $CHCl_3$).

7.31 Synthesis of (*R*)-3-(4-(tert-butoxy)-2,3,5,6-tetrafluorobenzyl)-3-methyl-2,3-dihydrobenzofuran (**7d**).

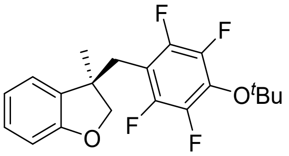

7d

Prepared according to typical procedure from allyl ether **6d** (200.0 mg, 0.9 mmol), after a flash column chromatography (hexanes: Et_2O = 50:1) afforded the product **7d** as a colorless liquid (95.8 mg, 87% yield) with 87% *ee*. ^{1}H NMR (400 MHz, $CDCl_3$) δ 7.19-7.11 (m, 1 H), 6.99 (dd, J = 7.4, 1.4 Hz, 1 H), 6.86 (td, J = 7.4, 1.0 Hz, 1 H), 6.76 (dt, J = 7.9, 0.8 Hz, 1 H), 4.53 (d, J = 8.9 Hz, 1 H), 4.11 (d, J = 8.9 Hz, 1 H), 3.06-2.95 (m, 2 H), 1.40 (dt, J = 2.3, 1.2 Hz, 12 H). ^{13}C NMR (101 MHz, $CDCl_3$) δ 159.38, 146.70, 144.48-144.28 (m, J_F = 20.0 Hz)), 141.88, 133.50, 132.70, 128.65, 122.87, 120.61, 111.20 (t, J_F = 18.9 Hz), 109.76, 84.73, 82.35(t, J_F = 2.9 Hz), 46.74, 32.91, 28.33, 23.82. ^{19}F NMR (376 MHz, $CDCl_3$) δ -142.62 (dd, J = 22.6, 9.3 Hz), -152.11 (dd, J = 23.0, 9.3 Hz). HRMS (EI) calculated for $[C_{20}H_{20}F_4O_2]^+$: 368.1394 found: 368.1395. Enantiomeric excess was determined by HPLC with a Chiralpak IG column (hexanes: 2-propanol = 99:1, 0.3 mL/min, 210 nm); minor enantiomer tr = 15.7 min, major enantiomer tr = 16.6 min. $[\alpha]_D^{20}$ = -22.5 (*c* = 0.5, $CHCl_3$).

7.32 Synthesis of (*R*)-3-methyl-3-(2,3,5,6-tetrafluoro-4-(naphthalen-1-yloxy)benzyl)-2,3-dihydrobenzofuran (**7e**).

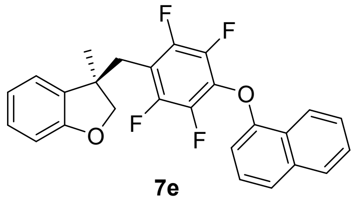

7e

Prepared according to typical procedure from allyl ether **6e** (263.0 mg, 0.9 mmol), after a flash column chromatography (hexanes: Et_2O = 50:1) afforded the product **7e** as a white solid (114.4 mg, 87% yield) with 90% *ee*. Mp: 120-122 ^{o}C. ^{1}H NMR (400 MHz, $CDCl_3$) δ 8.45-8.42 (m, 1 H), 7.94-7.88 (m, 1 H), 7.66-7.58 (m, 3 H), 7.41-7.32 (m, 1 H), 7.24-7.18 (m, 1 H), 7.10 (dd, J = 7.4, 1.4 Hz, 1 H), 6.94 (td, J = 7.4, 1.0 Hz, 1 H), 6.85 (dt, J = 8.0, 0.8 Hz, 1 H), 6.68-6.63 (m, 1 H), 4.60 (d, J = 9.0 Hz, 1 H), 4.18 (d, J

= 9.0 Hz, 1 H), 3.17-3.06 (m, 2 H), 1.49 (t, J = 1.2 Hz, 3 H).^{13}C NMR (101 MHz, $CDCl_3$) δ 159.39, 153.15, 146.98, 144.51 (dm, J_F = 12.8 Hz), 142.59 (dm, J_F = 17.4 Hz), 140.08 (dm, J_F = 12.5 Hz), 134.73, 133.25, 132.27, 128.79, 127.62, 126.93, 126.18, 125.15, 124.80, 123.43, 122.88, 121.59, 120.73, 112.77 (t, J_F = 18.9 Hz), 109.83, 107.44, 82.25 (t, J_F = 2.5 Hz), 46.78, 33.04, 23.85. ^{19}F NMR (376 MHz, $CDCl_3$) δ -140.57 (dd, J = 22.2, 9.6 Hz), -154.55 – -154.68 (m). HRMS (EI) calculated for $[C_{26}H_{18}F_4O_2]^+$: 438.1237 found: 438.1237. Enantiomeric excess was determined by HPLC with a Chiralpak ADH column (hexanes: 2-propanol = 99:1, 1.0 mL/min, 230 nm); minor enantiomer tr = 8.6 min, major enantiomer tr = 9.5 min. $[\alpha]_D^{20}$ = -10.5 (c = 0.5, $CHCl_3$).

7.33 Synthesis of (*R*)-3-methyl-3-(2,3,5,6-tetrafluoro-4-(naphthalen-2-yloxy)benzyl)-2,3-dihydrobenzofuran (**7f**).

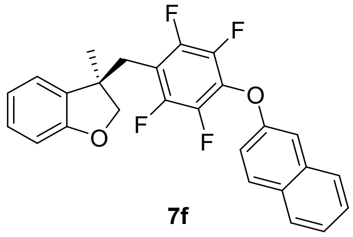

7f

Prepared according to typical procedure from allyl ether **6f** (263.0 mg, 0.9 mmol), after a flash column chromatography (hexanes: Et_2O = 50:1) afforded the product **7f** as a white solid (112.2 mg, 85% yield) with 93% *ee*. Mp: 125-127 °C. ^{1}H NMR (400 MHz, $CDCl_3$) δ 7.85 (t, J = 8.3 Hz, 2 H), 7.72 (dd, J = 8.2, 1.2 Hz, 1 H), 7.53 – 7.38 (m, 2 H), 7.31 (dd, J = 9.0, 2.6 Hz, 1 H), 7.19 – 7.15 (m, 2 H), 7.08 (dd, J = 7.5, 1.4 Hz, 1 H), 6.92 (td, J = 7.4, 1.0 Hz, 1 H), 6.83 (dt, J = 8.0, 0.7 Hz, 1 H), 4.59 (d, J = 9.0 Hz, 1 H), 4.17 (d, J = 9.0 Hz, 1 H), 3.15 – 3.04 (m, 2 H), 1.48 (d, J = 1.2 Hz, 3 H). ^{13}C NMR (101 MHz, $CDCl_3$) δ 159.47, 155.11, 147.02, 144.58, 142.71 (dm, J_F = 14.9 Hz), 140.30 (dm, J_F = 18.0 Hz), 133.97, 133.29, 130.34, 130.19, 128.87, 127.84, 127.12, 126.94, 125.03, 122.97, 120.79, 117.20, 113.03, 112.85 (d, J_F = 18.0 Hz), 110.16, 109.89, 82.34, 46.87, 33.16, 23.95. ^{19}F NMR (376 MHz, $CDCl_3$) δ -140.59 (dt, J = 19.8, 8.9 Hz), -154.60 (dd, J = 21.5, 9.4 Hz). HRMS (EI) calculated for $[C_{26}H_{18}F_4O_2]^+$: 438.1237 found: 438.1237.

Enantiomeric excess was determined by HPLC with a Chiralpak ADH column (hexanes: 2-propanol = 99:1, 1.0 mL/min, 230 nm); minor enantiomer tr = 7.6 min, major enantiomer tr = 8.2 min. $[\alpha]_D^{20}$ = -2.5 (c = 0.3, $CHCl_3$).

7.34 Synthesis of ethyl (*R*)-3-methyl-3-(2,3,5,6-tetrafluoro-4-((1-methylnaphthalen-2-yl)oxy)benzyl)-2,3-dihydrobenzofuran (**7g**).

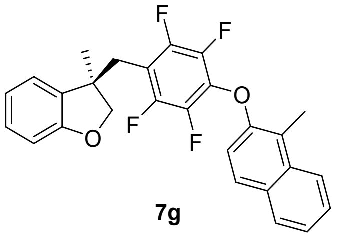

7g

Prepared according to typical procedure from allyl ether **6g** (275.7 mg, 0.9 mmol), after a flash column chromatography (hexanes: Et_2O = 50:1) afforded the product **7g** as a white solid (116.4 mg, 86% yield) with 90% *ee*. Mp: 81-83 °C. ^1H NMR (400 MHz, $CDCl_3$) δ 8.06 (d, J = 8.5 Hz, 1 H), 7.84-7.81 (m, 1 H), 7.67 (d, J = 9.0 Hz, 1 H), 7.61-7.56 (m, 1 H), 7.52-7.44 (m, 1 H), 7.19 (td, J = 7.7, 1.4 Hz, 1 H), 7.07 (dd, J = 7.5, 1.4 Hz, 1 H), 6.93 (q, J = 8.1 Hz, 2 H), 6.83 (d, J = 8.0 Hz, 1 H), 4.57 (d, J = 9.0 Hz, 1 H), 4.16 (d, J = 9.0 Hz, 1 H), 3.08 (qt, J = 13.6, 1.9 Hz, 2 H), 2.75 (s, 3 H), 1.46 (s, 3 H). ^{13}C NMR (101 MHz, $CDCl_3$) δ 159.37, 152.04, 146.94, 144.50, 142.62(dm, J_F = 15.2 Hz), 140.15 (dm, J_F = 13.0 Hz), 138.01, 133.61, 133.33, 130.49, 128.76, 128.44, 127.51, 126.69, 124.67, 123.88, 122.87, 121.03, 120.72, 115.09, 111.92 (d, J_F = 19.0 Hz)), 109.84, 82.25 (t, J_F = 2.8 Hz), 46.77, 32.96, 23.86, 10.90. ^{19}F NMR (376 MHz, $CDCl_3$) δ -140.96 (dd, J = 22.4, 9.5 Hz), -155.72 – -155.80 (m). HRMS (EI) calculated for $[C_{27}H_{20}F_4O_2]^+$: 452.1394 found: 452.1393. Enantiomeric excess was determined by HPLC with a Chiralpak IG column (hexanes: 2-propanol = 99:1, 0.3 mL/min, 210 nm); minor enantiomer tr = 21.2 min, major enantiomer tr = 27.0 min. $[\alpha]_D^{20}$ = -11.4 (c = 0.5, $CHCl_3$).

7.35 Synthesis of (*R*)-3-methyl-3-(2,3,5,6-tetrafluoro-4-phenoxybenzyl)-2,3-dihydrobenzofuran (**7h**).

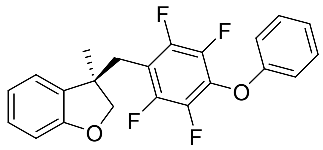

7h

Prepared according to typical procedure from allyl ether **6h** (218.0 mg, 0.9 mmol), after a flash column chromatography (hexanes: Et_2O = 50:1) afforded the product **7h** as a white solid (105.6 mg, 90% yield) with 90% *ee*. Mp: 59-60 °C. ^{1}H NMR (400 MHz, $CDCl_3$) δ 7.41-7.31 (m, 2 H), 7.15-7.06 (m, 2 H), 7.05 (dd, J = 7.4, 1.4 Hz, 1 H), 6.97-6.88 (m, 3 H), 6.80 (dt, J = 8.0, 0.8 Hz, 1 H), 4.56 (d, J = 9.0 Hz, 1 H), 4.15 (d, J = 9.0 Hz, 1 H), 3.12-3.01 (m, 2 H), 1.45 (t, J = 1.2 Hz, 3 H). ^{13}C NMR (101 MHz, $CDCl_3$) δ 159.37, 157.18, 146.97 (dm, J_F = 11.7 Hz), 144.47, 142.60 (dm, J_F = 16.0 Hz), 140.11 (dm, J_F = 16.5 Hz), 133.27, 132.12, 129.77, 128.78, 123.64, 122.87, 120.72, 115.44, 112.58 (t, J_F = 18.9 Hz), 109.83, 82.26 (t, J_F = 2.9 Hz), 46.78, 33.02, 23.86. ^{19}F NMR (376 MHz, $CDCl_3$) δ -140.81 (dd, J = 22.3, 9.5 Hz), -154.69 – -154.81 (m). HRMS (EI) calculated for $[C_{22}H_{16}F_4O_2]^+$: 388.1081 found: 388.1083. Enantiomeric excess was determined by HPLC with a Chiralpak IG column (hexanes: 2-propanol = 99:1, 0.3 mL/min, 210 nm); minor enantiomer tr = 19.9 min, major enantiomer tr = 21.8 min. $[\alpha]_D^{20}$ = -15.6 (c = 0.5, $CHCl_3$).

7.36 Synthesis of (*R*)-3-methyl-3-(2,3,5,6-tetrafluoro-4-(p-tolyloxy)benzyl)-2,3-dihydrobenzofuran (**7i**).

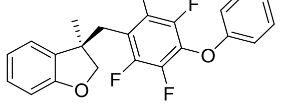

7i

Prepared according to typical procedure from allyl ether **6i** (230.6 mg, 0.9 mmol), after a flash column chromatography (hexanes: Et_2O = 50:1) afforded the product **7i** as a white solid (103.7 mg, 86% yield) with 90% *ee*. Mp: 92-94 °C. ^{1}H NMR (400 MHz, $CDCl_3$) δ 7.19-7.11 (m, 3 H), 7.05 (dd, J = 7.4, 1.4 Hz, 1 H), 6.92 -6.79 (m, 4 H), 4.56 (d, J = 9.0 Hz, 1 H), 4.15 (d, J = 9.0 Hz, 1 H), 3.12-3.01 (m, 2 H), 2.33 (s, 3 H), 1.45 (t, J = 1.2 Hz, 3 H). ^{13}C NMR (101 MHz, $CDCl_3$) δ 159.42, 155.28, 146.97–146.81 (dm,

J_F = 16.0 Hz), 144.62-144.37 (dm, J_F = 25.3 Hz), 142.67 (dm, J $_F$ = 16.0 Hz), 140.18 (dm, J_F = 16.4 Hz), 133.35, 133.23, 132.56, 130.21, 128.80, 122.91, 120.75, 115.40, 112.37 (t, J_F = 19.0 Hz), 109.86, 82.31 (t, J_F = 2.9 Hz), 46.82, 33.04, 23.90, 20.60. ^{19}F NMR (376 MHz, $CDCl_3$) δ -140.97 (dd, J = 22.2, 9.3 Hz), -154.86 – -154.94 (m). HRMS (EI) calculated for $[C_{23}H_{18}F_4O_2]^+$: 402.1237 found: 402.1238. Enantiomeric excess was determined by HPLC with a Chiralpak OJH column (hexanes: 2-propanol = 95:5, 0.5 mL/min, 210 nm); minor enantiomer tr = 17.3 min, major enantiomer tr = 20.1 min. $[\alpha]_D^{20}$ = -10.3 (c = 0.5, $CHCl_3$).

7.37 Synthesis of (*R*)-3-methyl-3-(2,3,5,6-tetrafluoro-4-(4-methoxyphenoxy)benzyl)-2,3-dihydrobenzofuran (**7j**) .

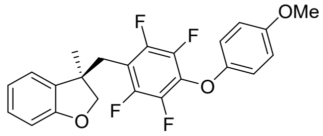

7j

Prepared according to typical procedure from allyl ether **6j** (245.0 mg, 0.9 mmol), after a flash column chromatography (hexanes: Et_2O = 30:1) afforded the product **7j** as a white solid (107.5 mg, 86% yield) with 89% *ee*. Mp: 60-62 °C. ^{1}H NMR (400 MHz, $CDCl_3$) δ 7.21-7.14 (m, 1 H), 7.06 (dd, J = 7.4, 1.4 Hz, 1 H), 6.96-6.91 (m, 3 H), 6.89-6.84 (m, 2 H), 6.81 (dt, J = 8.0, 0.8 Hz, 1 H), 4.56 (d, J = 9.0 Hz, 1 H), 4.15 (d, J = 9.0 Hz, 1 H), 3.79 (s, 3 H), 3.12-3.01 (m, 2 H), 1.45 (t, J = 1.2 Hz, 3 H). ^{13}C NMR (101 MHz, $CDCl_3$) δ 159.35, 155.85, 151.28, 146.95-146.75 (dm, J_F = 20.2 Hz), 144.55-144.35 (dm, J_F = 20.2 Hz), 142.65-142.50 (dm, J_F = 15.2 Hz), 140.17-139.97 (dm, J_F = 20.2 Hz), 133.30, 133.02, 128.72, 122.84, 120.67, 116.80, 114.69, 112.15 (t, J_F = 18.9 Hz), 109.78, 82.20 (t, J_F = 2.8 Hz), 55.58, 46.72, 32.92, 23.80. ^{19}F NMR (376 MHz, $CDCl_3$) δ -140.97 (dd, J = 22.3, 9.4 Hz), -155.01 – -155.16 (m). HRMS (EI) calculated for $[C_{23}H_{18}F_4O_3]^+$: 418.1187 found: 418.1189. Enantiomeric excess was determined by HPLC with a Chiralpak IA column (hexanes: 2-propanol = 99:1, 0.3 mL/min, 210 nm); minor enantiomer tr = 27.0 min, major enantiomer tr = 28.4 min. $[\alpha]_D^{20}$ = -10.0 (c = 0.5, $CHCl_3$).

7.38 Synthesis of (*R*)-3-methyl-3-(2,3,5,6-tetrafluoro-4-(4-(trifluoromethyl)phenoxy)benzyl)-2,3-dihydrobenzofuran (**7k**).

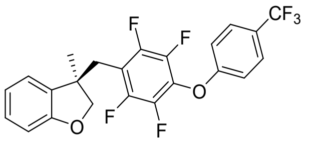

7k

Prepared according to typical procedure from allyl ether **6k** (279.2 mg, 0.9 mmol), after a flash column chromatography (hexanes: Et_2O = 50:1) afforded the product **7k** as a white solid (119.1 mg, 87% yield) with 92% *ee*. Mp: 79-81 °C. ^{1}H NMR (400 MHz, $CDCl_3$) δ 7.63-7.60 (m, 2 H), 7.19-7.14 (m, 1 H), 7.09-7.01 (m, 3 H), 6.91 (td, J = 7.4, 1.0 Hz, 1 H), 6.79 (dt, J = 8.0, 0.8 Hz, 1 H), 4.57 (d, J = 9.0 Hz, 1 H), 4.15 (d, J = 9.0 Hz, 1 H), 3.14-3.03 (m, 2 H), 1.47 (t, J = 1.1 Hz, 3 H). ^{13}C NMR (101 MHz, $CDCl_3$) δ 159.40, 159.30, 147.09-146.84 (tm, J_F = 25.3 Hz), 144.63-144.39 (tm, J_F = 24.2 Hz), 142.46-142.23 (dm, J_F = 20.2 Hz), 139.97-139.73 (dm, J_F = 24.2 Hz), 133.06, 131.13, 128.85, 127.33 (q, J_F = 3.7 Hz), 126.02 (q, J_F = 33.0 Hz), 123.91 (q, J_F = 270.0 Hz), 122.90, 120.76, 115.53, 113.54 (t, J_F = 18.7 Hz), 109.81, 82.25 (d, J_F = 2.8 Hz), 46.82, 33.16, 23.87. ^{19}F NMR (376 MHz, $CDCl_3$) δ -61.93, -140.08 (dd, J = 22.3, 9.6 Hz), -154.46 – -154.55 (m). HRMS (EI) calculated for $[C_{23}H_{15}F_7O_2]^+$: 456.0955 found: 456.0957. Enantiomeric excess was determined by HPLC with a Chiralpak ODH column (hexanes: 2-propanol = 99:1, 0.3 mL/min, 210 nm); minor enantiomer tr = 24.4 min, major enantiomer tr = 26.6 min. $[\alpha]_D^{20}$ = -7.6 (c = 0.5, $CHCl_3$). ^{13}C NMR (101 MHz, $CDCl_3$) δ 126.02 (q, *J* = 33.0 Hz).

7.39 Synthesis of (*R*)-3-methyl-3-((2,3,5,6-tetrafluoro-[1,1'-biphenyl]-4-yl)methyl)-2,3-dihydrobenzofuran (**7l**).

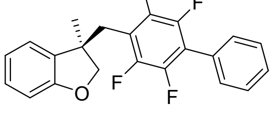

7l

Prepared according to typical procedure from allyl ether **6l** (203.6 mg, 0.9 mmol), after

a flash column chromatography (hexanes: Et_2O = 100:1) afforded the product **7l** as a white solid (96.1 mg, 86% yield) with 87% *ee*. Mp: 79-80 °C. ^1H NMR (400 MHz, $CDCl_3$) δ 7.55-7.44 (m, 5 H), 7.20 (td, J = 7.7, 1.4 Hz, 1 H), 7.12 (dd, J = 7.4, 1.4 Hz, 1 H), 6.93 (td, J = 7.4, 1.0 Hz, 1 H), 6.84 (d, J = 8.0 Hz, 1 H), 4.60 (d, J = 8.9 Hz, 1 H), 4.18 (d, J = 8.9 Hz, 1 H), 3.18-3.08 (m, 2 H), 1.47 (t, J = 1.3 Hz, 3 H). ^{13}C NMR (101 MHz, $CDCl_3$) δ 159.32, 146.72 (tm, J_F = 9.6 Hz), 144.83 (dm, J_F = 15.5 Hz), 144.38-144.27 (tm, J_F = 12.1 Hz), 142.50-142.30 (dm, J_F = 20.2 Hz), 133.66, 130.13-130.09 (tm, J_F = 4.0 Hz), 129.03, 128.69, 128.56, 127.44, 122.84, 120.70, 119.44-119.11 (tm, J_F = 33.3 Hz), 115.54 (t, J_F = 18.6 Hz), 109.87, 82.15 (t, J_F = 2.6 Hz), 46.80, 33.10, 23.99. ^{19}F NMR (376 MHz, $CDCl_3$) δ -141.16 (dd, J = 23.2, 12.5 Hz), -144.47 (dd, J = 22.7, 12.7 Hz). HRMS (EI) calculated for $[C_{22}H_{16}F_4O]^+$: 372.1132 found: 372.1132. Enantiomeric excess was determined by HPLC with a Chiralpak IF column (hexanes: 2-propanol = 99:1, 0.3 mL/min, 190 nm); minor enantiomer tr = 18.4 min, major enantiomer tr = 19.5 min. $[\alpha]_D^{20}$ = -26.2 (c = 0.5, $CHCl_3$).

7.40 Synthesis of (*R*)-3-(4-benzyl-2,3,5,6-tetrafluorobenzyl)-3-methyl-2,3-dihydrobenzofuran (**7m**).

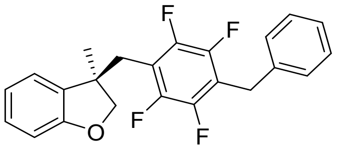

7m

Prepared according to typical procedure from allyl ether **6m** (279.2 mg, 0.9 mmol), after a flash column chromatography (hexanes: Et_2O = 100:1) afforded the product **7m** as a white solid (98.5 mg, 85% yield) with 88% *ee*. Mp: 90-92 °C. ^1H NMR (400 MHz, $CDCl_3$) δ 7.33-7.22 (m, 5 H), 7.18-7.11 (m, 1 H), 7.04 (dd, J = 7.4, 1.4 Hz, 1 H), 6.89 (td, J = 7.4, 1.0 Hz, 1 H), 6.79 (dt, J = 8.0, 0.8 Hz, 1 H), 4.52 (d, J = 9.0 Hz, 1 H), 4.10 (d, J = 9.0 Hz, 1 H), 4.05 (d, J = 1.8 Hz, 2 H), 3.10-2.96 (m, 2 H), 1.39 (t, J = 1.3 Hz, 3 H). ^{13}C NMR (101 MHz, $CDCl_3$) δ 159.30, 146.25 (dm, J_F = 16.2 Hz), 145.94, 143.84, 143.51 (dm, J_F = 33.3 Hz), 137.77, 133.71, 128.69, 128.65, 128.43, 126.79, 122.82, 120.65, 118.11 (t, J_F = 18.7 Hz), 114.66 (t, J_F = 18.6 Hz), 109.84, 82.16 (t, J_F = 2.8 Hz),

46.74, 33.00, 28.59, 23.92. ^{19}F NMR (376 MHz, $CDCl_3$) δ -141.43 (dd, J = 22.7, 12.8 Hz), -144.25 (dd, J = 22.7, 12.7 Hz). HRMS (EI) calculated for $[C_{23}H_{18}F_4O_3]^+$: 386.1288 found: 386.1288. Enantiomeric excess was determined by HPLC with a Chiralpak IG column (hexanes: 2-propanol = 99:1, 0.3 mL/min, 210 nm); minor enantiomer tr = 18.0 min, major enantiomer tr = 21.9 min. $[\alpha]_D^{20}$ = -22.5 (c = 0.5, $CHCl_3$).

7.41 Synthesis of (*R*)-1-(2,3,5,6-tetrafluoro-4-((3-methyl-2,3-dihydrobenzofuran-3-yl)methyl)phenyl)-1H-indole (**7n**).

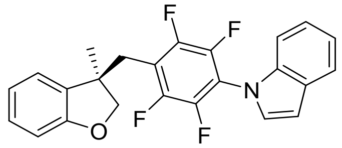

7n

Prepared according to typical procedure from allyl ether **6n** (238.7 mg, 0.9 mmol), after a flash column chromatography (hexanes: Et_2O = 50:1) afforded the product **7n** as a white solid (105.7 mg, 86% yield) with 91% *ee*. Mp: 130-132 °C. ^{1}H NMR (400 MHz, $CDCl_3$) δ 7.72-7.71 (m, 1 H), 7.33-7.27 (m, 1 H), 7.26-7.11 (m, 5 H), 6.95 (td, J = 7.4, 1.0 Hz, 1 H), 6.84 (dt, J = 8.0, 0.6 Hz, 1 H), 6.80 (dd, J = 3.3, 0.9 Hz, 1 H), 4.62 (d, J = 9.0 Hz, 1 H), 4.20 (d, J = 9.0 Hz, 1 H), 3.22-3.11 (m, 2 H), 1.51 (t, J = 1.2 Hz, 3 H). ^{13}C NMR (101 MHz, $CDCl_3$) δ 159.43, 146.98 (tm, J_F = 74.7 Hz), 144.52, 143.84 (dm, J_F = 16.3 Hz), 141.41-141.25 (dm, J_F = 16.2 Hz), 136.31, 133.25, 128.96, 128.72, 128.27, 123.14, 122.93, 121.25, 121.24, 120.87, 117.35, 116.01 (tm, J_F = 18.8 Hz), 110.45, 109.99, 105.49, 82.25 (t, J_F = 2.6 Hz), 46.97, 33.37, 24.01. ^{19}F NMR (376 MHz, $CDCl_3$) δ -139.70, -146.64 (dd, J = 21.9, 10.2 Hz). HRMS (EI) calculated for $[C_{24}H_{17}F_4NO]^+$: 411.1241 found: 411.1245. Enantiomeric excess was determined by HPLC with a Chiralpak IG column (hexanes: 2-propanol = 99:1, 0.3 mL/min, 254 nm); minor enantiomer tr = 23.7 min, major enantiomer tr = 24.9 min. $[\alpha]_D^{20}$ = -18.1 (c = 0.5, $CHCl_3$).

7.42 Synthesis of (*R*)-3-methyl-3-(2,3,6-trifluorobenzyl)-2,3-dihydrobenzofuran

(**7o**).

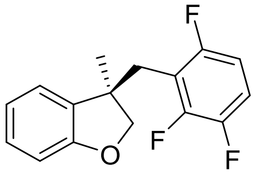

7o

Prepared according to typical procedure from allyl ether **6o** (118.8 mg, 0.9 mmol), after a flash column chromatography (hexanes: Et_2O = 50:1) afforded the product **7o** as a colorless liquid (41.5 mg, 51% yield) with 68% *ee*. ^{1}H NMR (400 MHz, $CDCl_3$) δ 7.19 -7.13 (m, 1 H), 7.08-6.98 (m, 2 H), 6.88 (t, J = 7.4 Hz, 1 H), 6.81-6.75 (m, 2 H), 4.55 (d, J = 8.9 Hz, 1 H), 4.11 (d, J = 8.9 Hz, 1 H), 3.04 (qt, J = 13.6, 1.9 Hz, 2 H), 1.39 (s, 3 H). ^{13}C NMR (101 MHz, $CDCl_3$) 159.39, 158.13 (d, J_F = 6.4 Hz), 155.68, 151.05–148.22 (dm, J_F = 230.3 Hz), 148.24–145.29 (dm, J_F = 231.3 Hz), 134.02, 128.55, 122.94, 120.59, 116.03 (dd, J_F = 22.4, 16.7 Hz), 115.26 (m, J_F = 19.3 Hz), 110.36 (m, J = 25.9 Hz), 109.82, 82.34 (t, J_F = 2.8 Hz), 46.75, 32.94 (d, J_F = 1.6 Hz), 24.01 (d, J_F = 1.9 Hz). ^{19}F NMR (376 MHz, $CDCl_3$) δ -117.24 (dt, J = 14.1, 6.3 Hz), -135.06 (dd, J = 21.5, 8.8 Hz), -142.31– -142.45 (m). HRMS (EI) calculated for $[C_{24}H_{17}F_4NO]^+$: 278.0918 found: 278.0922. Enantiomeric excess was determined by HPLC with a Chiralpak OJH column (hexanes: 2-propanol = 98:2, 1.0 mL/min, 210 nm); minor enantiomer tr = 7.4 min, major enantiomer tr = 8.2 min. $[\alpha]_D^{20}$ = -12.0 (c = 0.4, $CHCl_3$).

7.43 Synthesis of (*R*)-3-methyl-3-(2,4,6-trifluorobenzyl)-2,3-dihydrobenzofuran (**7p**).

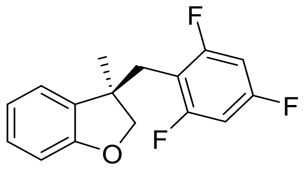

7p

Prepared according to typical procedure from allyl ether **6p** (118.8 mg, 0.9 mmol), after a flash column chromatography (hexanes: Et_2O = 50:1) afforded the product **7p** as a

colorless liquid (39.9 mg, 51% yield) with 67% *ee*. ^{1}H NMR (400 MHz, $CDCl_3$) δ 7.14 (td, J = 7.7, 1.4 Hz, 1 H), 7.01 (dd, J = 7.5, 1.4 Hz, 1 H), 6.87 (td, J = 7.4, 1.0 Hz, 1 H), 6.78 (d, J = 8.0 Hz, 1 H), 6.67 – 6.55 (m, 2 H), 4.52 (d, J = 8.9 Hz, 1 H), 4.10 (d, J = 8.8 Hz, 1 H), 2.90-3.01 (m, 2 H), 1.37 (d, J = 1.3 Hz, 3 H). ^{13}C NMR (101 MHz, $CDCl_3$) δ 163.41-162.20 (dm, J_F = 122.2 Hz), 161.57-159.95 (dm, J_F = 163.6 Hz), 159.42, 134.07, 128.46, 122.98, 120.51, 110.10, 109.77, 101.35-96.86 (dm, J_F = 453.5 Hz), 82.38 (t, J_F = 2.8 Hz), 46.68, 32.27, 23.97 (t, J_F = 1.8 Hz). ^{19}F NMR (376 MHz, $CDCl_3$) δ -109.01 – -109.18 (m), -109.88 – -110.10 (m). HRMS (EI) calculated for $[C_{24}H_{17}F_4NO]^+$: 278.0918 found: 278.0915. Enantiomeric excess was determined by HPLC with a Chiralpak OJH column (hexanes: 2-propanol = 98:2, 1.0 mL/min, 210 nm); minor enantiomer tr = 5.7 min, major enantiomer tr = 6.7 min. $[\alpha]_D^{20}$ = -14.4 (c = 0.4, $CHCl_3$).

7.44 Synthesis of (*R*)-2-((3-methyl-2,3-dihydrobenzofuran-3-yl)methyl)-5-phenyl-1,3,4-oxadiazole (**9a**).

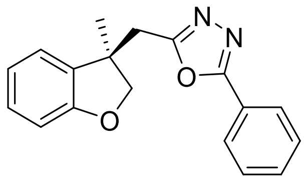

9a

Prepared according to typical procedure B from **8a** (44.0 mg, 0.3 mmol), after a flash column chromatography (hexanes: Et_2O = 5:1) afforded the product **9a** as a white solid (62.0 mg, 72% yield) with 91% *ee*. Mp: 85-86 °C. ^{1}H NMR (400 MHz, $CDCl_3$) δ 7.95-7.93 (m, 2 H), 7.55–7.46 (m, 3 H), 7.21-7.14 (m, 2 H), 6.95-6.80 (m, 2 H), 4.71 (d, J = 9.1 Hz, 1 H), 4.28 (d, J = 9.1 Hz, 1 H), 3.45-2.89 (m, 2 H), 1.52 (s, 3 H).^{13}C NMR (101 MHz, $CDCl_3$) δ 164.90, 164.05, 159.30, 131.69, 128.99, 126.80, 123.80, 122.80, 120.93, 110.14, 81.71, 45.07, 36.15, 24.67. HRMS (EI) calculated for $[C_{24}H_{17}F_4NO]^+$: 292.1212 found: 292.1210. Enantiomeric excess was determined by HPLC with a Chiralpak OJH column (hexanes: 2-propanol = 85:15, 0.5 mL/min, 254 nm); major enantiomer tr = 32.1 min, minor enantiomer tr = 36.4 min. $[\alpha]_D^{20}$ = -11.5 (c = 0.3, $CHCl_3$).

7.45 Synthesis of (*R*)-2-(4-methoxyphenyl)-5-((3-methyl-2,3-dihydrobenzofuran-3-yl)methyl)-1,3,4-oxadiazole (**9b**).

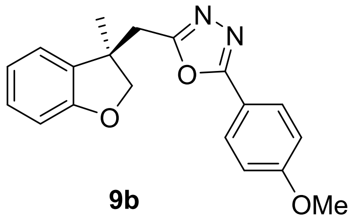

9b

Prepared according to typical procedure B from **8b** (52.4 mg, 0.3 mmol), after a flash column chromatography (hexanes: Et_2O = 3:1) afforded the product **9b** as a white solid (68.8 mg, 72% yield) with 90% *ee*. Mp: 91-92 °C. ^1H NMR (400 MHz, $CDCl_3$) δ 7.80 (dd, J = 8.6, 1.6 Hz, 2 H), 7.13-7.07 (m, 2 H), 6.94–6.84 (m, 3 H), 6.75–6.72 (m, 1 H), 4.63 (dd, J = 9.1, 1.1 Hz, 1 H), 4.20 (dd, J = 9.1, 1.2 Hz, 1 H), 3.80 (d, J = 1.8 Hz, 3 H), 3.22-3.04 (m, 2 H), 1.45 (d, J = 1.1 Hz, 3 H). ^{13}C NMR (101 MHz, $CDCl_3$) δ 164.89, 163.52, 162.30, 159.34, 133.30, 128.93, 128.53, 122.83, 120.89, 116.28, 114.48, 110.08, 81.71, 55.45, 45.03, 36.09, 24.68. HRMS (EI) calculated for $[C_{24}H_{17}F_4NO]^+$: 322.1317 found: 322.1314. Enantiomeric excess was determined by HPLC with a Chiralpak ODH column*2 (hexanes: 2-propanol = 75:25, 0.5 mL/min, 254 nm); minor enantiomer tr = 48.8 min, major enantiomer tr = 57.6 min. $[\alpha]_D^{20}$ = -9.1 (c = 0.4, $CHCl_3$).

7.46 Synthesis of (*R*) -1-(5-((3-methyl-2,3-dihydrobenzofuran-3-yl)methyl)thiophen-2-yl)ethan-1-one (**9c**).

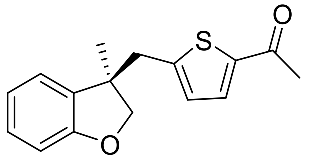

9c

Prepared according to typical procedure B from **8c** (40.2 mg, 0.3 mmol), after a flash column chromatography (hexanes: Et_2O = 10:1) afforded the product **9c** as a yellow solid (41.4 mg, 52% yield) with 92% *ee*. Mp: 78-79 °C. ^1H NMR (400 MHz, $CDCl_3$) δ 7.31 (d, J = 5.4 Hz, 1 H), 7.14 (td, J = 7.1, 1.5 Hz, 2 H), 7.02 (d, J = 5.4 Hz, 1 H), 6.91 (td, J = 7.4, 1.0 Hz, 1 H), 6.70 (dd, J = 8.4, 1.0 Hz, 1 H), 4.50 (d, J = 9.0 Hz, 1 H), 4.14

(d, J = 9.0 Hz, 1 H), 4.05 (d, J = 14.1 Hz, 1 H), 3.21 (d, J = 14.1 Hz, 1 H), 2.47 (s, 3 H), 1.46 (s, 3 H). ^{13}C NMR (101 MHz, $CDCl_3$) 13C NMR (101 MHz, CDCl3) δ 194.67, 159.99, 148.64, 137.30, 133.83, 128.58, 128.41, 123.27, 123.25, 120.46, 109.66, 81.39, 47.20, 38.49, 30.26, 26.16. HRMS (EI) calculated for $[C_{24}H_{17}F_4NO]^+$: 272.0871 found: 272.0873. Enantiomeric excess was determined by HPLC with a Chiralpak OZ3 column (hexanes: 2-propanol = 95:5, 0.5 mL/min, 254 nm); minor enantiomer tr =14.9 min, major enantiomer tr = 16.4 min. $[\alpha]_D^{20}$ = 85.6 (c = 0.3, $CHCl_3$).

7.47 Synthesis of (*R*) -3-(benzo[b]thiophen-2-ylmethyl)-3-methyl-2,3-dihydrobenzofurane (**9d**).

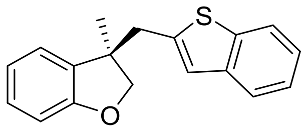

9d

Prepared according to typical procedure from **8d** (120.6 mg, 0.9 mmol), after a flash column chromatography (hexanes: Et_2O = 20:1) afforded the product **9d** as a yellow solid (64.0 mg, 76% yield) with 90% *ee*. Mp: 62-63 °C ^{1}H NMR (400 MHz, $CDCl_3$) δ 1H NMR (400 MHz, CDCl3) δ 7.79-7.75 (m, 1 H), 7.70–7.61 (m, 1 H), 7.38-7.26 (m, 2 H), 7.21-7.09 (m, 2 H), 7.03-6.92 (m, 2 H), 6.83 (dd, J = 8.0, 0.9 Hz, 1 H), 4.62 (d, J = 8.8 Hz, 1 H), 4.20 (d, J = 8.9 Hz, 1 H), 3.27–3.16 (m, 2 H), 1.50 (s, 3 H). ^{13}C NMR (101 MHz, $CDCl_3$) δ 13C NMR (101 MHz, CDCl3) δ 159.66, 140.69, 139.93, 139.76, 134.38, 128.62, 124.19, 123.84, 123.08, 122.97, 122.05, 120.62, 109.93, 81.76, 46.22, 41.62, 25.54. HRMS (EI) calculated for $[C_{24}H_{17}F_4NO]^+$: 280.0922 found: 280.0926. Enantiomeric excess was determined by HPLC with a Chiralpak ODH column*2 (hexanes: 2-propanol = 95:5, 0.5 mL/min, 254 nm); major enantiomer tr = 27.2 min, minor enantiomer tr = 28.6 min. $[\alpha]_D^{20}$ = 9.0 (c = 0.3, $CHCl_3$).

7.48 Synthesis of (*R*)-2-((3-methyl-2,3-dihydrobenzofuran-3-yl)methyl)benzofurane (**9e**).

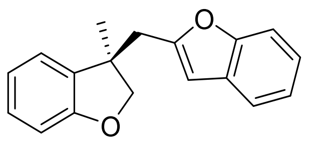

9e

Prepared according to typical procedure from **8e** (120.6 mg, 0.9 mmol), after a flash column chromatography (hexanes: Et_2O = 20:1) afforded the product **9e** as a yellow solid (59.2 mg, 76% yield) with 90% *ee*. ^1H NMR (400 MHz, $CDCl_3$) δ 7.50-7.48 (m, 1 H), 7.44-7.41 (m, 1 H), 7.23-7.12 (m, 3 H), 7.09 (dd, J = 7.4, 1.5 Hz, 1 H), 6.92-6.88 (m, 1 H), 6.82 (dd, J = 7.9, 1.0 Hz, 1 H), 6.36 (s, 1 H), 4.68 (dd, J = 8.9, 1.6 Hz, 1 H), 4.20 (dd, J = 8.9, 1.5 Hz, 1 H), 3.14-3.00 (m, 2 H), 1.43 (d, J = 1.5 Hz, 3 H). ^{13}C NMR (101 MHz, $CDCl_3$) δ 159.39, 155.63, 154.81, 134.65, 128.47, 123.57, 122.86, 122.62, 120.61, 120.48, 110.93, 109.88, 104.94, 82.23, 45.74, 39.16, 24.93. HRMS (EI) calculated for $[C_{24}H_{17}F_4NO]^+$: 264.1150 found: 264.1153. Enantiomeric excess was determined by HPLC with a Chiralpak ODH column (hexanes: 2-propanol = 99:1, 0.5 mL/min, 254 nm); major enantiomer tr = 16.4 min, minor enantiomer tr = 17.8 min. $[\alpha]_D^{20}$ = 3.7 (c = 0.3, $CHCl_3$).

8. General Procedure for the Synthesis of products 8-14

8.1 Synthesis of (*R*)-3-(3-iodopropyl)-3-((perfluorophenyl)methyl)-2,3-dihydrobenzofuran (**8**) [1].

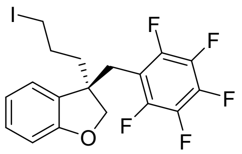

8

3i (112.8 mg, 0.3 mmol) and $CuSO_4$ (48 mg, 0.3 mmol, 1.0 equiv) were suspended in acetone (0.5 mL). The reaction mixture was stirred under **3i** (112.8 mg, 0.3 mmol) and NaI (225.0 mg, 1.5 mmol, 5.0 equiv) were suspended in acetone (1.2 mL) and the reaction mixture was stirred under 80℃ for 10 h. The solvent was removed under reduced pressure and the residue was purified by flash column chromatography (hexanes: Et_2O = 40:1) to provide analytical pure product **8** as colorless liquid (100 mg, 97% yield) with 92% *ee*. ^1H NMR (400 MHz, $CDCl_3$) δ 7.15 (td, J = 7.7, 1.4 Hz, 1 H),

6.98 (d, J = 7.4 Hz, 1 H), 6.88 (t, J = 7.4 Hz, 1 H), 6.73 (d, J = 8.0 Hz, 1 H), 4.44 (d, J = 9.3 Hz, 1 H), 4.26 (d, J = 9.3 Hz, 1 H), 3.26-3.01 (m, 4 H), 1.93–1.83 (m, 3 H), 1.68–1.56 (m, 1 H). ^{13}C NMR (101 MHz, $CDCl_3$) δ 159.76, 146.68, 144.24, 141.26, 138.77, 136.11, 130.46, 129.17, 123.41, 120.77, 110.98 (tm, J_F = 38.4 Hz), 109.87, 79.56 (d, J_F = 3.2 Hz), 49.89, 38.34, 31.89, 28.53, 6.27. ^{19}F NMR (376 MHz, $CDCl_3$) δ -139.81 (dd, J = 23.2, 8.2 Hz), -155.45 (t, J = 21.0 Hz), -162.11 (td, J = 22.1, 7.7 Hz). HRMS (EI) calculated for $[C_{18}H_{14}F_5IO]^+$: 468.0004 found: 467.9992. Enantiomeric excess was determined by HPLC with a Chiralpak ODH column (hexanes: 2-propanol = 98:2, 0.5 mL/min, 210 nm); minor enantiomer tr = 17.7 min, major enantiomer tr = 20.4 min. $[\alpha]_D^{20}$ = 12.5 (c = 0.5, $CHCl_3$).

8.2 Synthesis of (*R*)-3-(3-((perfluorophenyl)methyl)-2,3-dihydrobenzofuran-3-yl)propan-1-ol (**9**) [2].

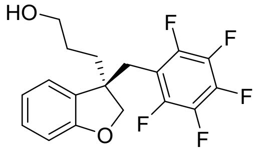

9

3i (112.8 mg, 0.3 mmol) and $CuSO_4$ (48 mg, 0.3 mmol, 1.0 equiv) were suspended in H_2O (0.35 mL) and DMSO (0.8 mL). The reaction mixture was stirred under 100°C for 72 h and extracted with EA. The combined organic phases were washed with brine, dried over anhydrous Na_2SO_4, and concentrated at the reduced pressure. The residue was purified by flash silica gel column chromatography (hexanes: Et_2O = 3:1) to provide analytical pure product **9** as colorless liquid (62 mg, 58% yield) with 89% *ee*. ^{1}H NMR (400 MHz, CDCl3) δ 7.13 (t, J = 7.7 Hz, 1 H), 6.97 (d, J = 7.4 Hz, 1 H), 6.86 (t, J = 7.4 Hz, 1 H), 6.72 (d, J = 8.0 Hz, 1 H), 4.44 (d, J = 9.2 Hz, 1 H), 4.28 (d, J = 9.2 Hz, 1 H), 3.63-3.59 (m, 2 H), 3.13–3.01 (m, 2 H), 1.86–1.76 (m, 2 H), 1.73–1.58 (m, 1 H), 1.42–1.32 (m, 2 H). ^{13}C NMR (101 MHz, CDCl3) δ 159.81, 146.68, 144.25, 141.20, 138.71, 130.89, 128.99, 123.42, 120.67, 111.20 (tm, J_F = 38 Hz), 109.76, 79.64 (t, J_F = 2.9 Hz), 62.82, 50.06, 33.64, 31.82, 27.79. ^{19}F NMR (376 MHz, CDCl3) δ -139.87 (dd, J = 23.0, 8.2 Hz), -155.74 (t, J = 21.0 Hz), -162.28 (td, J = 22.5, 7.9 Hz). HRMS (EI)

calculated for $[C_{18}H_{15}F_5O]^+$: 358.0987 found: 358.0989. Enantiomeric excess was determined by HPLC with a Chiralpak OJH column (hexanes: 2-propanol = 80:20, 0.5 mL/min, 210 nm); minor enantiomer tr = 9.1 min, major enantiomer tr = 10.3 min. $[\alpha]_D^{20}$ = 16.6 (c = 0.5, $CHCl_3$).

8.3 Synthesis of (*R*)-3-(3-chloropropyl)-3-(2,3,5,6-tetrafluoro-4-(phenylthio)benzyl)-2,3-dihydrobenzofuran (**10**) [1].

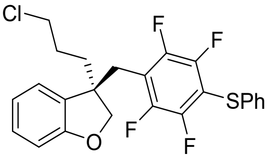

10

3i (75.2 mg, 0.2 mmol, 1.0 equiv) and NaSPh (31.7 mg, 0.24 mmol, 1.2 equiv) were suspended in acetone (1 mL). The reaction mixture was stirred under 80°C for 8 h. After the indicated time the reaction mixture was quenched with water and extracted with EA. The combined organic phases were washed with brine, dried over anhydrous Na_2SO_4, and concentrated at the reduced pressure. The residue was purified by flash silica gel column chromatography (hexanes: Et_2O = 30:1) to provide analytical pure product **10** as colorless liquid (65.9 mg, 71% yield) with 92% *ee*. ^{1}H NMR (400 MHz, $CDCl_3$) δ 7.33–7.21 (m, 5 H), 7.15 (t, J = 7.7 Hz, 1 H), 7.00 (d, J = 7.4 Hz, 1 H), 6.87 (t, J = 7.4 Hz, 1 H), 6.73 (d, J = 8.0 Hz, 1 H), 4.50 (d, J = 9.3 Hz, 1 H), 4.28 (d, J = 9.4 Hz, 1 H), 3.50 (t, J = 6.1 Hz, 2 H), 3.19–3.08 (m, 2 H), 1.97-1.80 (m, 3 H), 1.63–1.57 (m, 1 H). ^{13}C NMR (101 MHz, $CDCl_3$) δ 159.80, 148.06 (dm, J_F = 14.9 Hz), 146.68, 145.60 (dm, J_F = 14.5 Hz), 144.29, 133.30, 130.59, 130.15, 129.32, 129.13, 127.66, 123.46, 120.75, 117.77(t, J_F = 39.4 Hz), 112.25 (tm, J_F = 38.0 Hz), 109.81, 79.70 (t, J_F = 3.1 Hz), 50.16, 44.98, 34.87, 32.54, 27.84. ^{19}F NMR (376 MHz, $CDCl_3$) δ -133.24 – -133.38 (m), -139.14 (dd, J = 24.3, 12.0 Hz). HRMS (ESI) calculated for $[M+H]^+$ = $[C_{24}H_{20}ClF_4OS]^+$: 467.0855 found: 467.0916. Enantiomeric excess was determined by HPLC with a Chiralpak OZ3 column (hexanes: 2-propanol = 99:1, 0.5 mL/min, 210 nm); minor enantiomer tr = 18.0 min, major enantiomer tr = 21.7 min. $[\alpha]_D^{20}$ = 24.5 (c = 0.3, $CHCl_3$).

8.4 Synthesis of (*R*)-3-(3-(phenylthio)propyl)-3-(2,3,5,6-tetrafluoro-4-(phenylthio)benzyl)-2,3-dihydrobenzofuran (**11**) [1].

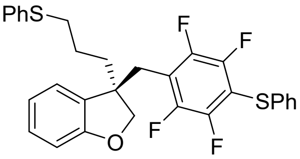

11

3i (75.2 mg, 0.2 mmol, 1.0 equiv) and NaSPh (79.2 mg, 0.6 mmol, 3.0 equiv) were suspended in acetone (1 mL). The reaction mixture was stirred under 80°C for 24 h. After the indicated time the reaction mixture was quenched with water and extracted with EA. The combined organic phases were washed with brine, dried over anhydrous Na_2SO_4, and concentrated at the reduced pressure. The residue was purified by flash silica gel column chromatography (hexanes: Et_2O = 30:1) to provide analytical pure product **11** as colorless liquid (87.2 mg, 81% yield) with 92% *ee*. ^{1}H NMR (400 MHz, $CDCl_3$) δ 7.33–7.24 (m, 9 H), 7.21–7.11 (m, 2 H), 6.94 (d, J = 7.4 Hz, 1 H), 6.84 (td, J = 7.5, 1.1 Hz, 1 H), 6.72 (d, J = 8.0 Hz, 1 H), 4.46 (d, J = 9.3 Hz, 1 H), 4.23 (d, J = 9.3 Hz, 1 H), 3.16–3.04 (m, 2 H), 2.95–2.84 (m, 2 H), 1.91 (dt, J = 10.2, 4.7 Hz, 2 H), 1.79-1.70 (m, 1 H), 1.56–1.43 (m, 1 H). ^{13}C NMR (101 MHz, $CDCl_3$) δ 159.78, 148.06 (dm, J_F = 18.8 Hz), 146.70, 145.59 (dm, J_F = 15.2 Hz), 144.24, 136.23, 133.38, 130.83, 130.10, 129.33, 129.29, 129.01, 128.94, 127.64, 126.07, 123.47, 120.67, 117.96 (t, J_F = 39.4 Hz), 111.97 (d, J_F = 20.2 Hz), 109.73, 79.79 (t, J_F = 2.9 Hz), 50.39, 36.37, 34.04, 32.50, 24.24. ^{19}F NMR (376 MHz, $CDCl_3$) δ -133.31 – -133.40 (m), -139.12 (dd, J = 24.4, 12.2 Hz). HRMS (ESI) calculated for $[M+H]^+$ = $[C_{30}H_{25}F_4OS_2]^+$: 541.1278 found: 541.1610. Enantiomeric excess was determined by HPLC with a Chiralpak ID column (hexanes: 2-propanol = 99:1, 0.5 mL/min, 210 nm); minor enantiomer tr = 19.1 min, major enantiomer tr = 20.5 min. $[\alpha]_D^{20}$ = 7.7 (c = 0.5, $CHCl_3$).

8.5 Synthesis of (*R*)-1-(4-((3-(3-chloropropyl)-2,3-dihydrobenzofuran-3-yl)methyl)-2,3,5,6-tetrafluorophenyl)-1H-indole (**12**) [4].

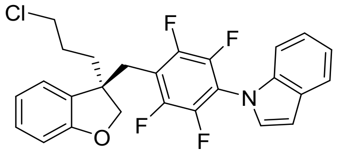

12

Under nitrogen atmosphere, **3i** (75.2 mg, 0.2 mmol, 1.0 equiv), indole (46.8 mg, 0.4 mmol, 2.0 equiv), NaOH (16 mg, 0.4 mmol) and DMF (2 mL) were added to a Schlenk flask. The reaction mixture was stirred under 70°C for 24 h. After the indicated time the reaction mixture was quenched with water and extracted with EA. The combined organic phases were washed with brine, dried over anhydrous Na_2SO_4, and concentrated at the reduced pressure. The residue was purified by flash silica gel column chromatography (hexanes: Et_2O = 30:1) to provide analytical pure product **12** as yellow liquid (74.8 mg, 79% yield) with 90% *ee*. ^{1}H NMR (400 MHz, $CDCl^3$) δ 7.71 (d, J = 7.7 Hz, 1 H), 7.29–7.27 (m, 1 H), 7.24–7.18 (m, 3 H), 7.11 (dd, J = 12.7, 7.8 Hz, 2 H), 6.96-6.90 (m, 1 H), 6.80–6.78 (m, 2 H), 4.56 (d, J = 9.3 Hz, 1 H), 4.35 (d, J = 9.3 Hz, 1 H), 3.54 (t, J = 6.2 Hz, 2 H), 3.22 (q, J = 13.6 Hz, 2 H), 2.05-1.85 (m, 3 H), 1.70-1.61 (m, 1 H). ^{13}C NMR (101 MHz, $CDCl^3$) δ 159.80, 146.92, 144.50, 143.83 (dm, J_F = 17.2 Hz), 141.32 (dm, J_F = 16.2 Hz), 136.21, 130.52, 129.17, 128.65, 128.17, 123.46, 123.07, 121.19, 121.17, 120.82, 120.79, 115.48 (t, J_F = 38.2 Hz), 110.38, 109.83, 105.44, 79.57, 50.16, 44.95, 34.94, 32.36, 27.81. ^{19}F NMR (376 MHz, $CDCl^3$) δ -139.24, -146.45 (dt, J = 23.6, 11.1 Hz). HRMS (EI) calculated for $[C_{26}H_{20}ClF_4NO]^+$: 473.1164 found: 473.1162. Enantiomeric excess was determined by HPLC with a Chiralpak IG column (hexanes: 2-propanol = 99:1, 0.5 mL/min, 210 nm); major enantiomer tr = 26.2 min, minor enantiomer tr = 28.8 min. $[\alpha]_D^{20}$ = 20.7 (c = 0.5, $CHCl_3$).

8.6 Synthesis of diphenyl (*R*)-(4-((3-(3-chloropropyl)-2,3-dihydrobenzofuran-3-yl)methyl)-2,3,5,6-tetrafluorophenyl)phosphonate[5].

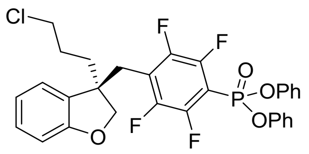

13

Under nitrogen atmosphere, dimethyl phosphite (70.2 mg, 0.3 mmol, 1.0 equiv), Cs_2CO_3 (293.4 mg, 0. 9 mmol), tetrabutylammonium iodide (333 mg, 0.9 mmol) and DMF (2 mL) were added to a Schlenk flask. The reaction mixture was stirred for 1 h at room temperature. After this time period, **3i** (135.4 mg, 0.3 mmol, 1.0 equiv) in DMF (1 mL) was added and stirred for an additional 48 h. The reaction mixture was quenched with water and extracted with EA. The combined organic phases were washed with brine, dried over anhydrous Na_2SO_4, and concentrated at the reduced pressure. The residue was purified by flash silica gel column chromatography (hexanes: Et_2O = 20:1) to provide analytical pure product **13** as colorless liquid (111.8 mg, 63% yield) with 92% *ee*. ^{1}H NMR (400 MHz, $CDCl_3$) δ 7.36-7.26 (m, 4 H), 7.19–7.10 (m, 2 H)), 7.03 (d, J = 7.4 Hz, 1 H), 6.97–6.93 (m, 3 H), 6.91–6.86 (m, 3 H), 6.77 (d, J = 8.0 Hz, 1 H), 4.55 (d, J = 9.3 Hz, 1 H), 4.36 (d, J = 9.3 Hz, 1 H), 3.95 (t, J = 6.0 Hz, 2 H), 3.21–3.10 (m, 2 H), 2.06–1.85 (m, 3 H), 1.71–1.61 (m, 1 H). ^{13}C NMR (101 MHz, $CDCl_3$) δ 159.87, 158.80, 157.16, 146.93, 144.50 (dm, J_F = 22.2 Hz), 142.64 (dm, J_F = 14.2 Hz), 139.99, 130.95, 129.76, 129.43, 128.96, 123.63, 123.53, 120.71, 120.64, 115.43, 114.43, 112.33 (t, J_F = 38.5 Hz), 109.68, 79.85 (d, J_F = 3.0 Hz), 67.59, 50.20, 33.88, 32.00, 24.68. ^{19}F NMR (376 MHz, $CDCl_3$) δ -140.34 (dd, J = 22.4, 9.3 Hz), -154.61 – -154.71 (m). ^{31}P NMR (162 MHz, $CDCl_3$) δ 31.50. HRMS (ESI) calculated for $[M+H]^+$ = $[C_{30}H_{25}ClF_4O_4P]^+$: 591.1110 found: 591.1347. Enantiomeric excess was determined by HPLC with a Chiralpak OZ3 column (hexanes: 2-propanol = 98:2, 0.5 mL/min, 210 nm); major enantiomer tr = 15.0 min, minor enantiomer tr = 15.9 min. $[\alpha]_D^{20}$ = 22.5 (c = 0.5, $CHCl_3$).

8.7 Synthesis of (*R*)-3-allyl-3-(4-(tert-butoxy)-2,3,5,6-tetrafluorobenzyl)-2,3-dihydrobenzofuran (**14**)[6].

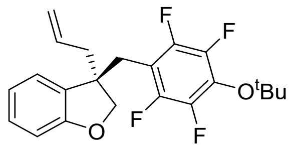

14

Under nitrogen atmosphere, **3i** (112.8 mg, 0.3 mmol, 1.0 equiv), ^{t}BuOK (67.3 mg, 0.6 mmol), 18-crown-6 (39.6 mg, 0.15 mmol) and Et_2O (3 mL) were added to a Schlenk flask. The reaction mixture was stirred under 60°C for 24 h. After the indicated time the reaction mixture was quenched with water and extracted with EA. The combined organic phases were washed with brine, dried over anhydrous Na_2SO_4, and concentrated at the reduced pressure. The residue was purified by flash silica gel column chromatography (hexanes: Et_2O = 20:1) to provide analytical pure product **14** as colorless liquid (118.2 mg, 84% yield) with 88% *ee*. ^{1}H NMR (400 MHz, $CDCl_3$) δ 7.12 (td, J = 7.7, 1.4 Hz, 1 H), 6.99 (d, J = 7.4 Hz, 1 H), 6.85 (td, J = 7.4, 1.1 Hz, 1 H), 6.70 (d, J = 8.0 Hz, 1 H), 5.73-5.63 (m, 1 H), 5.14 – 5.08 (m, 2 H), 4.45 (d, J = 9.2 Hz, 1 H), 4.32 (d, J = 9.2 Hz, 1 H), 3.12-3.02 (m, 2 H), 2.61 – 2.47 (m, 2 H), 1.38 (t, J = 1.2 Hz, 9 H). ^{13}C NMR (101 MHz, $CDCl_3$) δ 159.90, 146.74, 144.38 (dm, J_F = 13.4 Hz), 141.85 (dm, J_F = 18.2 Hz), 133.31, 131.08, 128.83, 123.58, 120.45, 118.97, 110.97 (t, J_F = 18.9 Hz), 109.56, 84.74, 79.52, 50.04, 41.89, 31.90, 28.32. ^{19}F NMR (376 MHz, $CDCl_3$) δ -142.17 (dd, J = 22.9, 9.0 Hz), -152.18 (dd, J = 23.0, 9.1 Hz). HRMS (ESI) calculated for $[M+H]^+$ = $[C_{22}H_{23}F_4O_2]^+$: 395.1629 found: 395.1274. Enantiomeric excess was determined by HPLC with a Chiralpak OZ3 column (hexanes: 2-propanol = 99:1, 0.3 mL/min, 210 nm); minor enantiomer tr = 16.1 min, major enantiomer tr = 17.4 min. $[\alpha]_D^{20}$ = -10.1 (c = 0.5, $CHCl_3$).

9. Non-linear effect experiments [8]

Non-linear effect reactions were set up with standard substrates (0.1 mmol), catalyst and ***N*-Me-Xu3** with varied *ee* following the standard procedure as described in previous section. In this case, the amount of palladium precatalyst and ***N*-Me-Xu3** enantiomers was weighed on an analytical balance, and the amount of solvent was measured with volumetric syringe. The reactions were allowed to run for 15 h, purification as described in standard procedure. Each experiment was repeated three times. The relationship between enantiomeric excess of ***N*-Me-Xu3** ligand and product is listed here.

Table S6. Non-linear effect experiments

**Ligand *ee***	**(*S*,*R*)-*N*-Me-Xu3 amount weighed (mg)**	**(*R*,*S*)-*N*-Me-Xu3 amount weighed (mg)**	**Product first *ee***	**Product second *ee***	**Product third *ee***	**Product average *ee***
0	6.4	6.4	0.3%	1.0%	1.5%	0.9%
20%	7.7	5.1	20.8%	22.8%	20.0%	21.2%
40%	9.0	3.8	41.8%	37.4%	38.8%	39.3%
60%	10.2	2.6	51.2%	54.4%	56.4%	54.0%
80%	11.5	1.3	71.4%	73.2%	70.8%	71.8%
100%	12.8	0	91.4%	92.0%	91.2%	91.5%

10. Kinetic Data: Order in Catalyst

General procedure: Catalyst was measured from a stock solution (64.7 mg $Pd_2(dba)_3$ and 159.5 mg **Xu4** dissolved in 5 mL $^iPr_2O$ using a Hamilton gastight syringe into 10 mL oven-dried sealed tube. Solvent was removed under reduced pressure, to the oven-dried sealed tube containing the resulting solid was added substrate **1a** (27.4 mg, 0.10 mmol, 1.0 equiv.), Cs_2CO_3 (65.2 mg, 0.2 mmol, 2.0 equiv.), Ag_2CO_3 (20.5 mg, 0.075 mmol, 0.75 equiv.). The flask was evacuated and refilled with argon. Then, **2a** (50.4 mg, 0.30 mmol, 3.0 equiv.), 1,3-dimethoxybenzene (13.8 mg, 0.10 mmol, 1.0 equiv.), $^iPr_2O$ (1 mL) was added to the tube. The tube was tightly sealed with a Teflon-lined screw cap and heated to 80 °C in a preheated aluminum heating block. After the desired reaction time (measured precisely by a timer), the reaction was flash-cooled in a liquid nitrogen bath until frozen solid (about 45 s). The reaction was then allowed to warm back up to room temperature and analyzed by GC. Yields and concentrations of **3a** are reported as averages of two independent vial reactions. The concentrations of **3a** were used to obtain initial rates and the rates were then plotted as a function of concentration of the varied catalyst.

Table S7. Amounts of catalyst and results used to determine the order in catalyst

Entry	Concentration of catalyst	Reaction time (min)	Average Yield (%)	**3a** (mM)
1	100 µL of stock solution = 2.5 mol%, 12.5 mM	30	2.9	2.9
2		60	4.9	4.9
3		90	6.4	6.4
4		120	8.1	8.1
5		180	10.9	10.9
6	200 µL of stock solution = 5.0 mol%, 25 mM	25	3.1	3.1
7		35	3.9	3.9
8		45	5.0	5.0
9		55	6.3	6.3

10		65	7.2	7.2
11	300 μL of stock solution = 7.5 mol%, 37.5 mM	25	3.6	3.6
12		35	4.7	4.7
13		45	7.3	7.3
14		55	8.3	8.3
15		65	9.6	9.6
16	400 μL of stock solution = 10.0 mol%, 50 mM	15	3.2	3.2
17		25	4.2	4.2
18		35	7.4	7.4
19		55	11.6	11.6
20		65	13.6	13.6

Figure S1. Plot of **3a** (mM) verse time (min) using different concentrations of catalyst

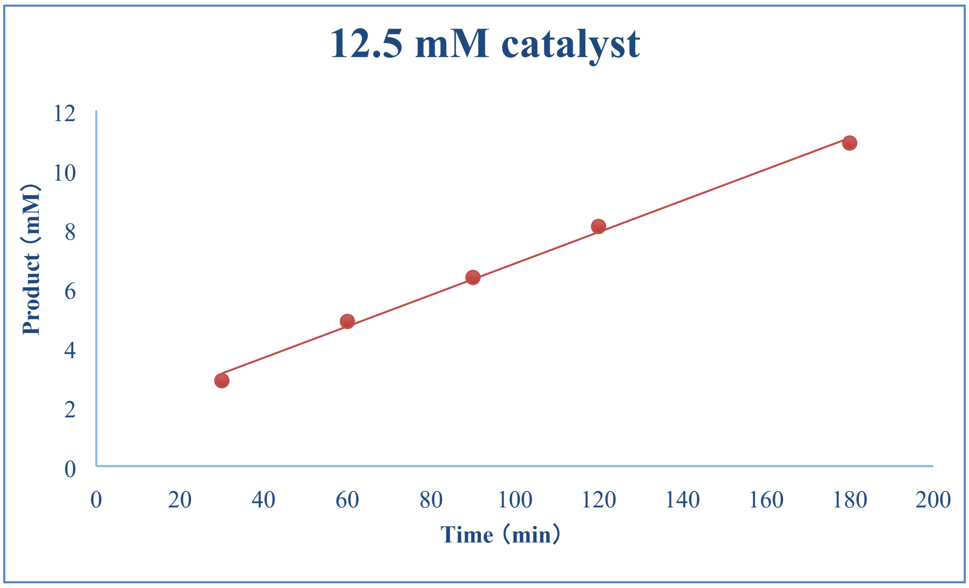

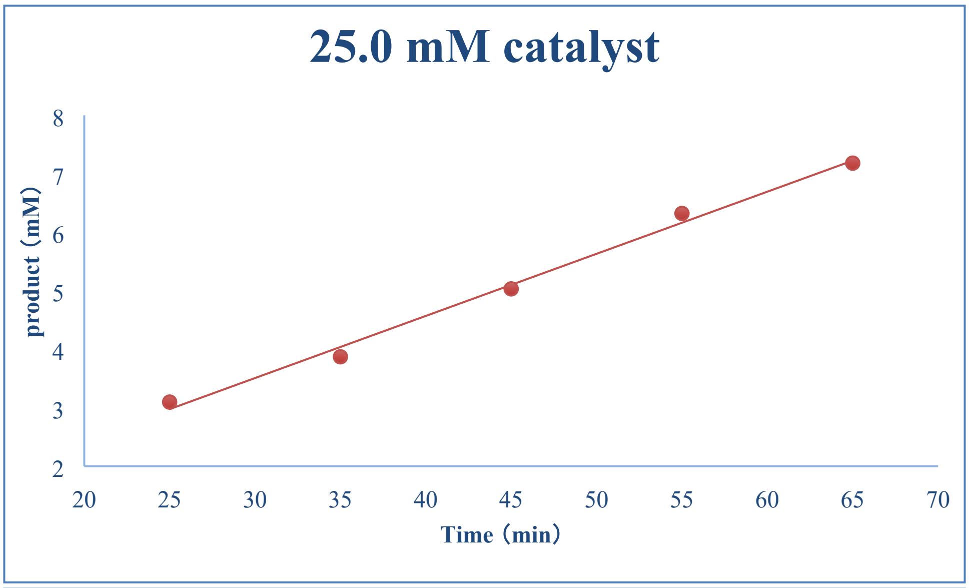
25.0 mM catalyst
product (mM)
8
7
6
5
4
3
2
20 25 30 35 40 45 50 55 60 65 70
Time (min)

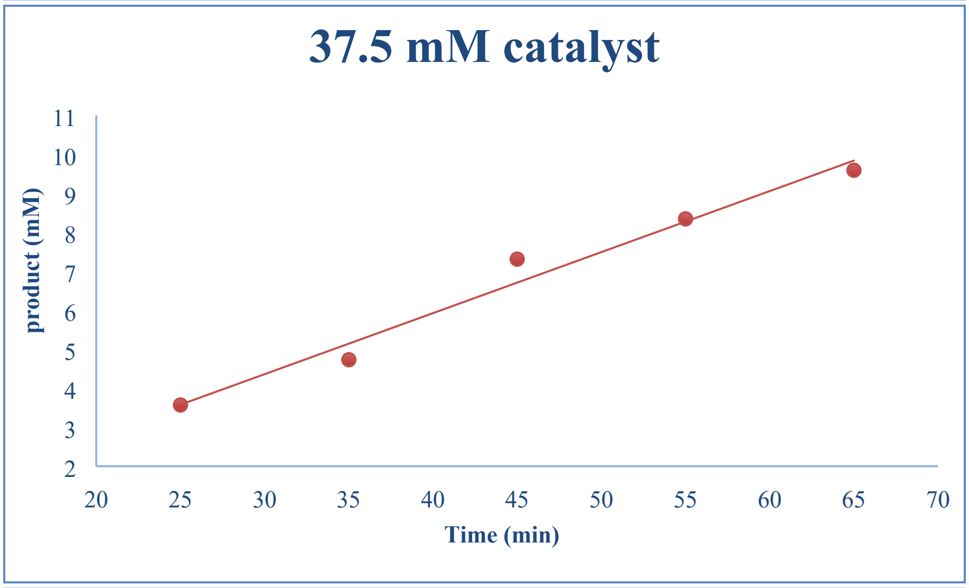
37.5 mM catalyst
product (mM)
11
10
9
8
7
6
5
4
3
2
20 25 30 35 40 45 50 55 60 65 70
Time (min)

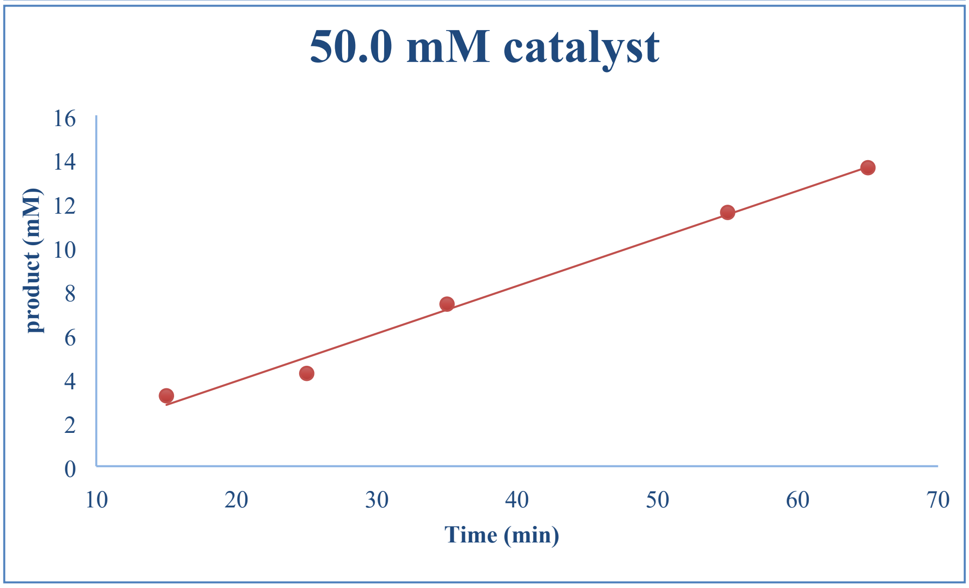
50.0 mM catalyst
product (mM)
16
14
12
10
8
6
4
2
0
10 20 30 40 50 60 70
Time (min)

Table S8. Rates determined while varying the concentrations of catalyst

Entry	Concentration of catalyst (mM)	Rate (mM/min)
1	12.5	0.053
2	25.0	0.1062
3	37.5	0.1565
4	50.0	0.217

Figure S2. Rates determined while varying the concentrations of catalyst.

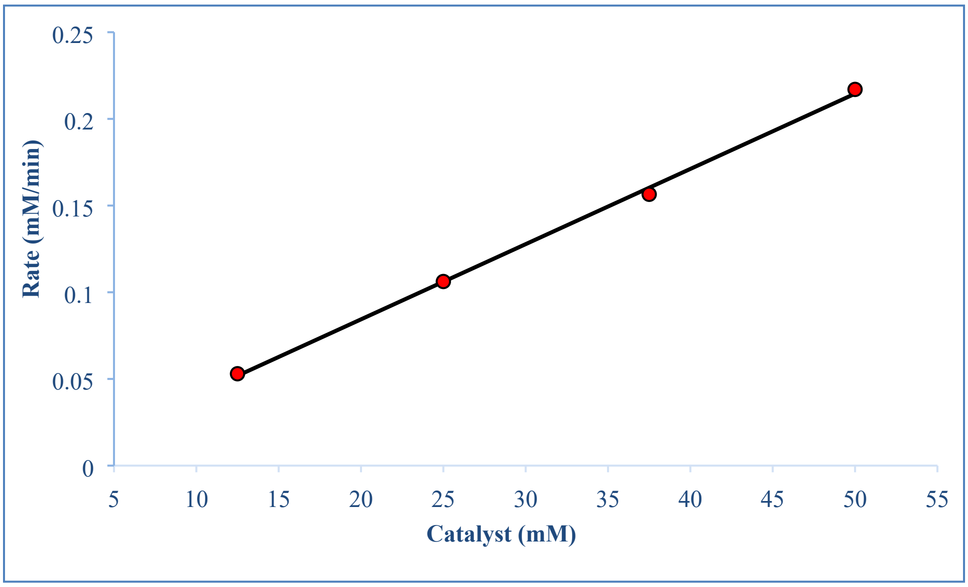

11. Kinetic Data: Deuterium Kinetic Isotope Experiment [9]

Side-by- side reaction

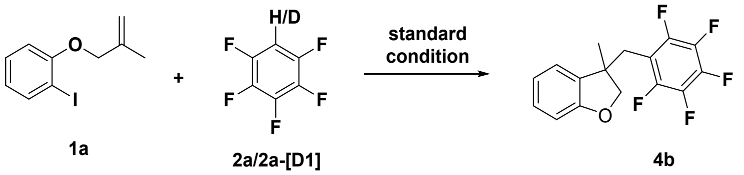

General procedure: Catalyst was measured from a stock solution (103.5 mg $Pd_2(dba)_3$•$CHCl_3$ and 255.2 mg N-CD_3-**Xu4** dissolved in 2.0 mL $^iPr_2O$) using a Hamilton gastight syringe into 10 mL oven-dried sealed tube. Solvent was removed under reduced pressure, to the oven-dried sealed tube containing the resulting solid was

added substrate **1a** (27.4 mg, 0.10 mmol, 1.0 equiv.), Cs_2CO_3 (65.2 mg, 0.2 mmol, 2.0 equiv.), Ag_2CO_3 (20.5 mg, 0.075 mmol, 0.75 equiv.). The flask was evacuated and refilled with argon. Then, **2a** (50.4 mg, 0.30 mmol, 3.0 equiv.) or **2a-[D1]** (50.7 mg, 0.30 mmol, 3.0 equiv.), $^{i}Pr_2O$ (1 mL) was added to the tube. The tube was tightly sealed with a Teflon-lined screw cap and heated to 80 °C in a preheated aluminum heating block. After the desired reaction time (measured precisely by a timer), the reaction was flash-cooled in a liquid nitrogen bath until frozen solid (about 45 s). The reaction was then allowed to warm back up to room temperature and analyzed by NMR. Yields and concentrations of **4a** are reported as averages of two independent vial reactions. The concentrations of **4a** were used to obtain initial rates and the rates were then plotted as a function of concentration of the varied catalyst.

Table S9. Yield(%) verse time (h) using **2a-[D1]** as a substrate.

Entry	Concentration of catalyst	Reaction time (h)	Average Yield (%)
1	100 μL of stock solution = 2.5 mol%	2	7
2		4	16
3		6	25
4		8	31

Figure S3. Plot of yield(%) verse time (h) using **2a-[D1]** as a substrate.

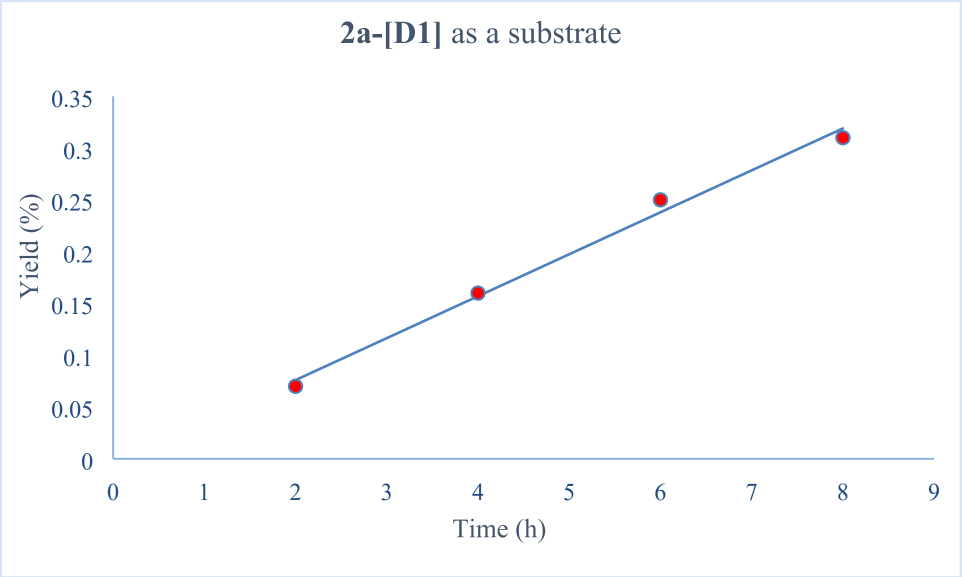

Table S10. Yield(%) verse time (h) using **2a** as a substrate.

Entry	Concentration of catalyst	Reaction time (h)	Average Yield (%)
1	100 µL of stock solution = 2.5 mol%	2	29
2		4	42
3		6	59
4		8	70

Figure S4. Plot of yield(%) verse time (h) using **2a** as a substrate.

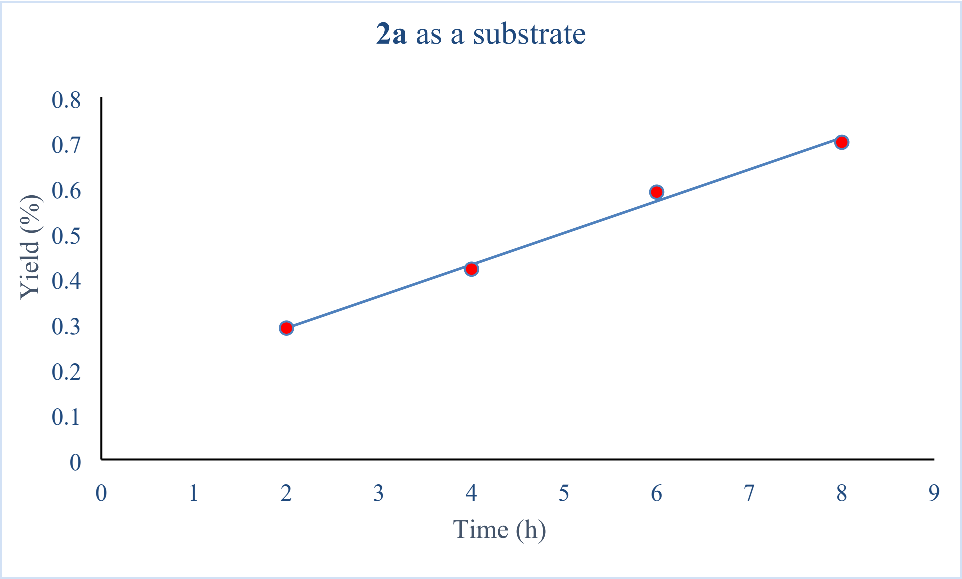

Intermolecular competition reaction

General procedure: The general procedure for reaction monitoring by ^{19}F NMR was used, with the use of internal standard α,α,α-trifluorotoluene (10 µL) and using 1.5 equiv of both **2a** and **2a-[D1]**. The KIE value was obtained using the consumption of (**2a** or **2a-[D1]**).

Table S11. Intermolecular competition experiments

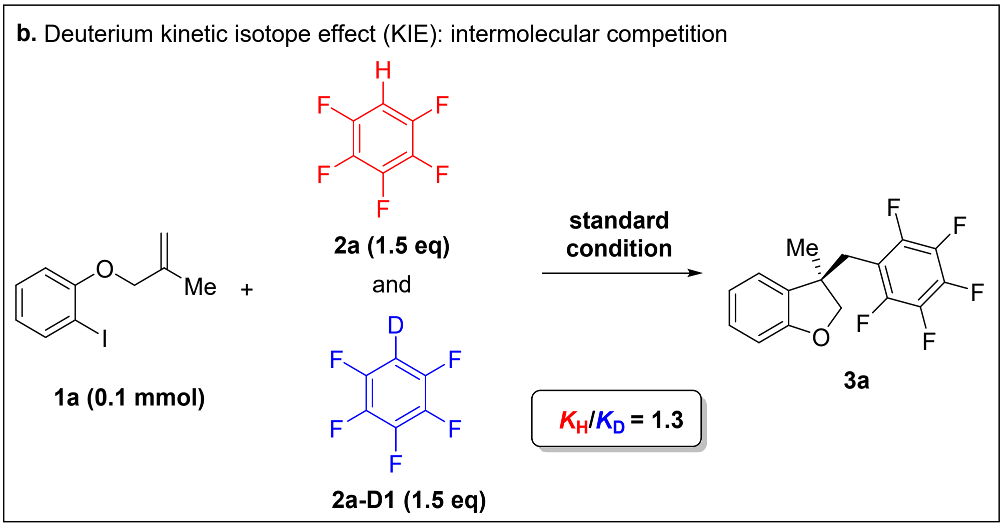

Figure S5. Intermolecular competition reaction monitoring by ^{19}F NMR.

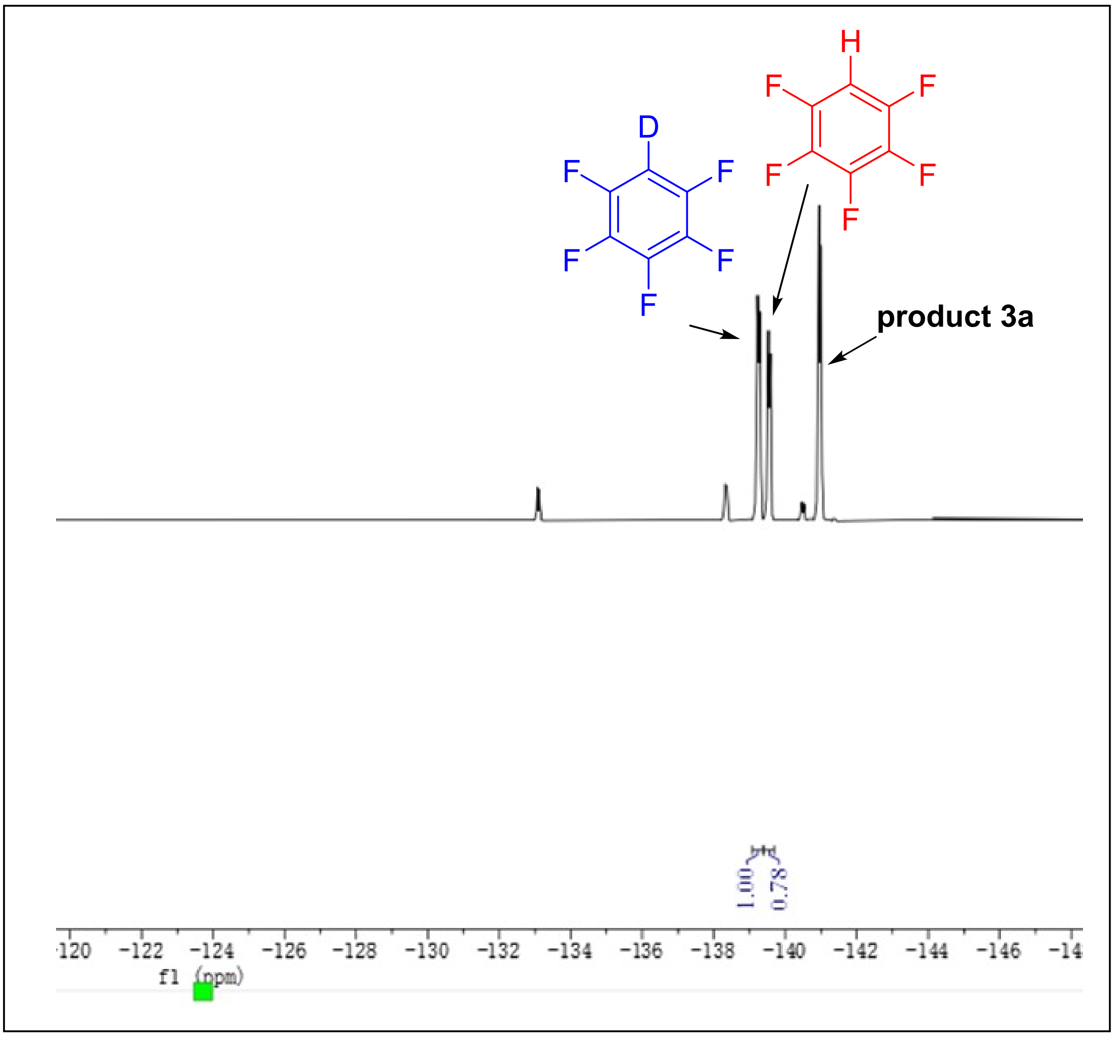

12. Control experiments of C_6F_5Ag and H/D exchanging control experiments.

General procedure: To a 10 mL oven-dried sealed tube was added substrate 1 (0.10 mmol, 1.0 equiv.), $Pd_2(dba)_3$•$CHCl_3$ (5.2 mg, 0.005 mmol, 5 mol%), Xu4 (12.8 mg, 0.02 mmol, 20 mol%), Cs_2CO_3 (65.2 mg, 0.2 mmol, 2.0 equiv.), Ag_2CO_3 (20.5 mg, 0.075 mmol, 0.75 equiv.). The flask was evacuated and refilled with argon. Then, substrate 2 (0.30 mmol, 3.0 equiv.), C_6D_6 (1 mL) was added to the tube, and stirred at room temperature for 1 h. Then the mixture was stirred at 80 °C for 10 h. The resulting slurry was cooled to room temperature, and a ^{19}F NMR spectrum was acquired at 25 ºC, showing the presence of C_6F_5Ag.

Table S12.C_6F_5Ag species control experiments.

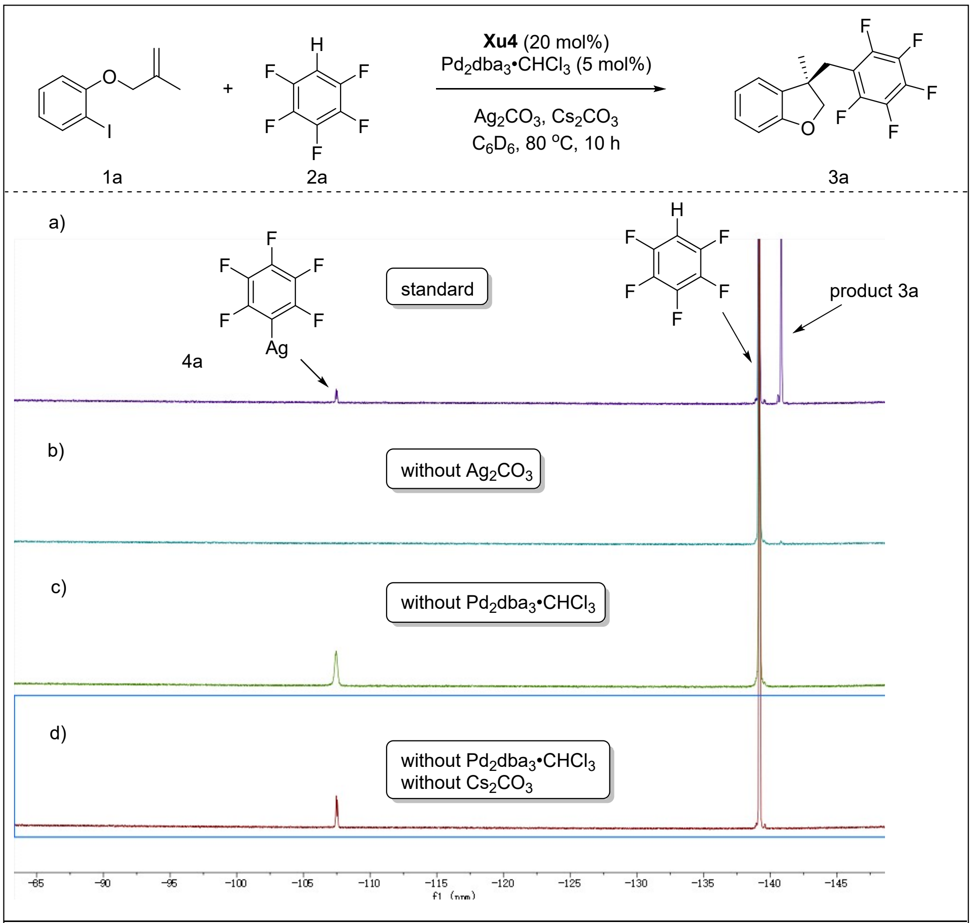

^{19}F NMR spectra (between −95 and−140 ppm): (a)Standard condition; (b)without Ag_2CO_3 (c) without Pd_2dba_3•$CHCl_3$ (d) without Pd_2dba_3•$CHCl_3$ and Cs_2CO_3

H/D exchanging control experiments.

General procedure: To a 10 mL oven-dried sealed tube was added $Pd_2(dba)_3$•$CHCl_3$ (5.2 mg, 0.005 mmol, 1.7 mol%), **Xu4** (12.8 mg, 0.02 mmol, 6.7 mol%), Cs_2CO_3 (65.2 mg, 0.2 mmol, 0.7 equiv.), Ag_2CO_3 (20.5 mg, 0.075 mmol, 0.25 equiv.). The flask was evacuated and refilled with argon. Then C_6F_5H (0.3 mmol, 1.0 eq), $^iPr_2O$ (1 mL) and D_2O (27 µL, 1.5 mmol, 5 equiv) was added to the tube, and stirred at room temperature for 1 h. Then the mixture was stirred at 80 °C for 10 h. The reaction mixture was cooled to room temperature and filtered through a plug of Celite. Acetone-d6 (20 µL, 272 µmol) was added as an internal standard. ^{2}H NMR spectra were acquired at 25 °C to determine the amount of deuterium incorporation.

Table S13. H/D exchanging control experiments.

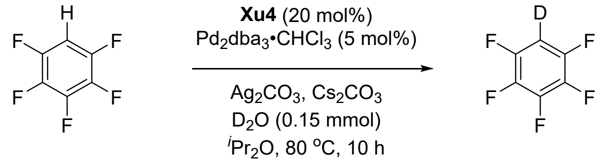

Entry^a		Deuteration incorporation (%)
1	standard	63% D
2	without Ag_2CO_3+ **1a** (0.01mmol)	<1% D
3	without $Pd_2(dba)_3$•$CHCl_3$	52% D
4	without $Pd_2(dba)_3$•$CHCl_3$; without Cs_2CO_3	61% D

^{a} Standard condition: Ag_2CO_3 (0.075 mmol), Cs_2CO_3 (0.2 mmol), D_2O (0.15 mmol), 10 mol% [Pd] and 20 mol% ligand in 1.0 mL $^iPr_2O$ at 80 °C for 10 h.

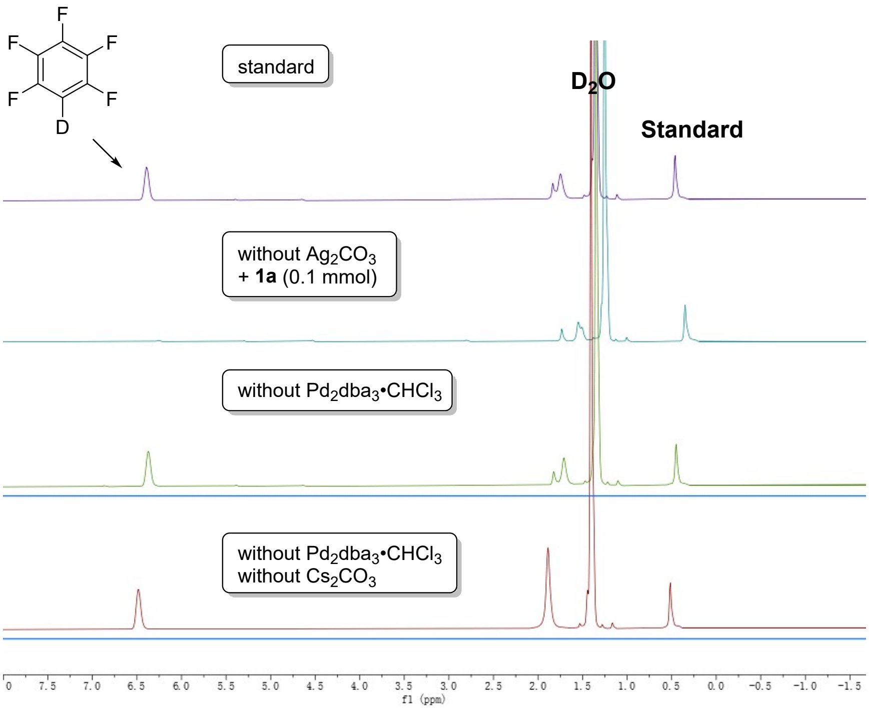

^{2}H NMR spectra for H/D exchange studies of pentafluorobenzene: (a)Standard condition; (b)without Ag_2CO_3 but with **1a** (0.1 mmol) (c) without Pd_2dba_3•$CHCl_3$ (d) without Pd_2dba_3•$CHCl_3$ and Cs_2CO_3

13. X-Ray Structure and Crystal Data of 7n

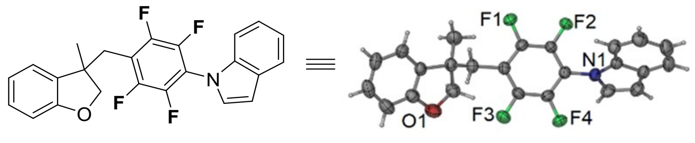

7n

Table 1 Crystal data and structure refinement for 7n.

Identification code	7n
Empirical formula	$C_{24}H_{17}F_4NO$
Formula weight	411.38
Temperature/K	296.3(6)
Crystal system	orthorhombic
Space group	$P2_12_12_1$
a/Å	6.42860(10)
b/Å	8.7411(2)
c/Å	33.5094(6)
α/°	90
β/°	90
γ/°	90
Volume/Å^3	1882.99(6)
Z	4
ρ_{calc}g/cm^3	1.451
μ/mm^{-1}	0.985
F(000)	848.0
Crystal size/mm^3	0.26 × 0.22 × 0.18
Radiation	CuKα (λ = 1.54184)
2Θ range for data collection/°	10.46 to 134.134
Index ranges	$-7 \le h \le 7$, $-10 \le k \le 10$, $-40 \le l \le 40$
Reflections collected	40249
Independent reflections	3362 [R_{int} = 0.0608, R_{sigma} = 0.0250]
Data/restraints/parameters	3362/0/273
Goodness-of-fit on F^2	1.060
Final R indexes [I>=2σ (I)]	R_1 = 0.0302, wR_2 = 0.0769
Final R indexes [all data]	R_1 = 0.0334, wR_2 = 0.0790
Largest diff. peak/hole / e Å^{-3}	0.10/-0.11
Flack parameter	-0.07(7)

Table 2 Fractional Atomic Coordinates (×10^4) and Equivalent Isotropic Displacement Parameters (Å^2×10^3) for 7n. U_{eq} is defined as 1/3 of of the trace of the orthogonalised U_{IJ} tensor.

Atom	*x*	*y*	*z*	U(eq)
F1	8260(2)	3636(2)	6426.7(4)	67.4(4)

F2	7504(2)	2975.4(18)	7184.6(4)	62.7(4)
F3	1848(2)	6245.2(18)	6348.3(4)	66.8(4)
F4	1188(2)	5696.7(19)	7116.2(4)	68.3(4)
O1	1414(3)	4899(2)	5367.6(6)	69.7(5)
N1	4000(3)	4069(2)	7586.2(5)	48.3(4)
C1	2910(4)	5304(3)	5091.8(7)	55.0(6)
C2	2525(5)	6107(3)	4745.9(8)	69.1(7)
C3	4199(6)	6399(3)	4503.5(8)	71.3(8)
C4	6184(5)	5924(3)	4602.0(7)	69.6(8)
C5	6536(4)	5140(3)	4956.3(7)	60.4(6)
C6	4861(4)	4837(3)	5201.4(6)	48.5(5)
C7	4740(4)	4103(3)	5609.8(6)	48.2(5)
C8	2392(4)	3803(3)	5630.0(7)	57.0(6)
C9	5456(4)	5318(3)	5920.7(6)	48.5(5)
C10	6009(5)	2641(3)	5640.7(8)	64.8(7)
C11	5060(3)	4931(3)	6353.1(6)	46.3(5)
C12	6455(3)	4129(3)	6587.3(6)	47.9(5)
C13	6104(3)	3809(3)	6983.0(6)	47.6(5)
C14	4336(3)	4336(3)	7176.4(6)	45.8(5)
C15	2920(3)	5148(3)	6947.5(6)	48.6(5)
C16	3275(4)	5421(3)	6548.9(6)	48.4(5)
C17	2230(4)	3426(3)	7755.4(7)	56.4(6)
C18	2404(4)	3445(3)	8155.3(7)	60.0(6)
C19	4323(4)	4163(3)	8253.7(6)	51.1(5)
C20	5311(5)	4584(3)	8609.1(7)	64.3(7)
C21	7158(5)	5359(3)	8593.6(7)	68.6(7)
C22	8091(4)	5715(3)	8232.5(8)	64.9(7)
C23	7180(4)	5312(3)	7876.0(7)	54.4(6)
C24	5305(3)	4549(3)	7892.1(6)	45.4(5)

Table 3 Anisotropic Displacement Parameters (Å^2×10^3) for 7n. The Anisotropic displacement factor exponent takes the form: $-2\pi^2[h^2a^{*2}U_{11}+2hka^*b^*U_{12}+\ldots]$.

Atom	U_{11}	U_{22}	U_{33}	U_{23}	U_{13}	U_{12}
F1	47.6(8)	102.2(12)	52.4(7)	0.8(8)	7.9(6)	15.8(8)
F2	55.5(8)	83.1(11)	49.5(7)	5.0(7)	-5.1(6)	21.9(8)
F3	72.5(9)	71.1(10)	56.9(8)	5.4(7)	-6.6(7)	25.8(8)
F4	59.1(8)	86.1(11)	59.8(8)	-2.4(7)	10.3(6)	24.9(8)
O1	51.8(9)	79.4(13)	78.0(12)	12.7(10)	-2.7(9)	0.7(10)
N1	45.6(9)	60.3(11)	39.1(9)	0.8(8)	3.6(7)	-4.2(9)
C1	56.8(14)	53.8(13)	54.4(12)	-2.6(11)	-6.1(11)	-1.4(12)
C2	75.8(18)	65.3(17)	66.3(15)	4.0(13)	-21.1(15)	3.0(15)
C3	103(2)	62.9(17)	47.6(13)	3.4(12)	-16.0(15)	-5.7(17)
C4	88(2)	78.0(19)	42.4(12)	-2.5(12)	8.6(13)	-10.1(18)

C5	62.1(14)	71.5(17)	47.4(12)	-6.5(11)	2.9(11)	6.1(14)
C6	58.6(13)	46.8(12)	40.2(10)	-5.4(9)	-2.9(9)	0.9(11)
C7	54.5(12)	45.9(12)	44.2(11)	-0.9(9)	-1.2(10)	-0.4(11)
C8	57.9(14)	57.6(15)	55.5(13)	0.9(11)	-3.7(11)	-6.3(13)
C9	55.7(13)	49.2(12)	40.5(11)	2.0(9)	1.0(9)	-4.4(11)
C10	75.7(18)	53.4(14)	65.2(15)	-0.2(12)	-0.4(14)	8.2(14)
C11	50.4(12)	48.5(12)	40.0(10)	-1.6(9)	-0.6(9)	-2.3(10)
C12	40.5(10)	59.5(14)	43.7(11)	-3.5(10)	2.7(9)	1.3(11)
C13	44.0(11)	57.2(13)	41.5(11)	1.3(9)	-3.4(9)	3.7(11)
C14	46.2(11)	52.1(13)	39.2(10)	-2.3(9)	1.1(9)	-1.4(10)
C15	45.3(12)	52.9(13)	47.4(11)	-5.9(10)	4.7(9)	6.1(11)
C16	50.1(12)	49.2(12)	45.9(11)	1.3(10)	-5.6(9)	8.0(11)
C17	46.1(12)	64.0(15)	58.9(13)	-1.3(11)	8.4(11)	-7.5(12)
C18	60.7(15)	66.2(16)	53.2(13)	4.2(11)	15.0(11)	-3.5(13)
C19	60.1(13)	50.9(13)	42.3(11)	3.2(10)	8.9(10)	4.7(12)
C20	88.5(19)	64.8(15)	39.8(11)	3.2(11)	3.9(12)	6.9(16)
C21	89(2)	68.0(16)	49.0(13)	-7.5(12)	-11.4(13)	1.2(16)
C22	62.4(15)	64.1(16)	68.3(15)	-7.2(13)	-8.8(13)	-6.6(13)
C23	55.7(13)	60.5(14)	47.0(11)	-0.9(11)	3.8(10)	-7.8(12)
C24	49.6(12)	46.5(12)	40.1(10)	-0.3(9)	2.4(9)	3.7(10)

Table 4 Bond Lengths for 7n.

Atom	Atom	Length/Å	Atom	Atom	Length/Å
F1	C12	1.350(3)	C7	C9	1.557(3)
F2	C13	1.341(3)	C7	C10	1.520(4)
F3	C16	1.347(3)	C9	C11	1.509(3)
F4	C15	1.338(3)	C11	C12	1.383(3)
O1	C1	1.380(3)	C11	C16	1.389(3)
O1	C8	1.444(3)	C12	C13	1.373(3)
N1	C14	1.410(3)	C13	C14	1.387(3)
N1	C17	1.390(3)	C14	C15	1.386(3)
N1	C24	1.389(3)	C15	C16	1.376(3)
C1	C2	1.377(4)	C17	C18	1.345(3)
C1	C6	1.369(3)	C18	C19	1.423(4)
C2	C3	1.372(4)	C19	C20	1.399(4)
C3	C4	1.382(4)	C19	C24	1.407(3)
C4	C5	1.389(4)	C20	C21	1.369(4)
C5	C6	1.380(3)	C21	C22	1.386(4)
C6	C7	1.513(3)	C22	C23	1.376(3)
C7	C8	1.534(3)	C23	C24	1.378(3)

Table 5 Bond Angles for 7n.

Atom	Atom	Atom	Angle/°	Atom	Atom	Atom	Angle/°
C1	O1	C8	105.92(19)	F1	C12	C13	117.4(2)
C17	N1	C14	126.16(19)	C13	C12	C11	123.0(2)
C24	N1	C14	125.17(18)	F2	C13	C12	119.2(2)
C24	N1	C17	108.39(18)	F2	C13	C14	119.65(18)
C2	C1	O1	124.7(2)	C12	C13	C14	121.2(2)
C6	C1	O1	112.5(2)	C13	C14	N1	121.8(2)
C6	C1	C2	122.9(2)	C15	C14	N1	121.53(19)
C3	C2	C1	116.9(3)	C15	C14	C13	116.71(19)
C2	C3	C4	121.8(2)	F4	C15	C14	119.77(19)
C3	C4	C5	120.2(3)	F4	C15	C16	119.1(2)
C6	C5	C4	118.4(2)	C16	C15	C14	121.1(2)
C1	C6	C5	119.9(2)	F3	C16	C11	119.46(19)
C1	C6	C7	108.8(2)	F3	C16	C15	117.7(2)
C5	C6	C7	131.3(2)	C15	C16	C11	122.8(2)
C6	C7	C8	99.35(18)	C18	C17	N1	109.5(2)
C6	C7	C9	107.50(18)	C17	C18	C19	108.0(2)
C6	C7	C10	113.0(2)	C20	C19	C18	135.0(2)
C8	C7	C9	112.2(2)	C20	C19	C24	117.8(2)
C10	C7	C8	112.4(2)	C24	C19	C18	107.2(2)
C10	C7	C9	111.7(2)	C21	C20	C19	119.5(2)
O1	C8	C7	106.8(2)	C20	C21	C22	121.3(2)
C11	C9	C7	116.07(19)	C23	C22	C21	121.1(3)
C12	C11	C9	123.3(2)	C22	C23	C24	117.5(2)
C12	C11	C16	115.06(19)	N1	C24	C19	106.96(19)
C16	C11	C9	121.6(2)	C23	C24	N1	130.17(19)
F1	C12	C11	119.56(18)	C23	C24	C19	122.8(2)

Table 6 Hydrogen Atom Coordinates (Å×10^4) and Isotropic Displacement Parameters (Å^2×10^3) for 7n.

Atom	*x*	*y*	*z*	U(eq)
H2	1193.1	6434.96	4679.77	83
H3	3991.94	6931.38	4266.47	86
H4	7287.15	6129.43	4430.7	84
H5	7869.49	4826.27	5026.57	72
H8A	2081.94	2768.65	5543.92	68
H8B	1890.7	3932.66	5900.97	68
H9A	6936.76	5485.6	5886.3	58
H9B	4758.63	6275.06	5860.88	58
H10A	5561.55	1932.85	5439.28	97

H10B	5812.54	2193.59	5899.63	97
H10C	7455.55	2872.74	5602.77	97
H17	1100.68	3040.88	7613.96	68
H18	1439.62	3057.2	8335.83	72
H20	4716.33	4339.25	8853.94	77
H21	7801	5653.31	8830.08	82
H22	9353.62	6234.2	8230.96	78
H23	7805.69	5545.03	7633.27	65

14. ^{1}H , ^{19}F, ^{31}P, ^{13}C NMR and HPLC Spectra

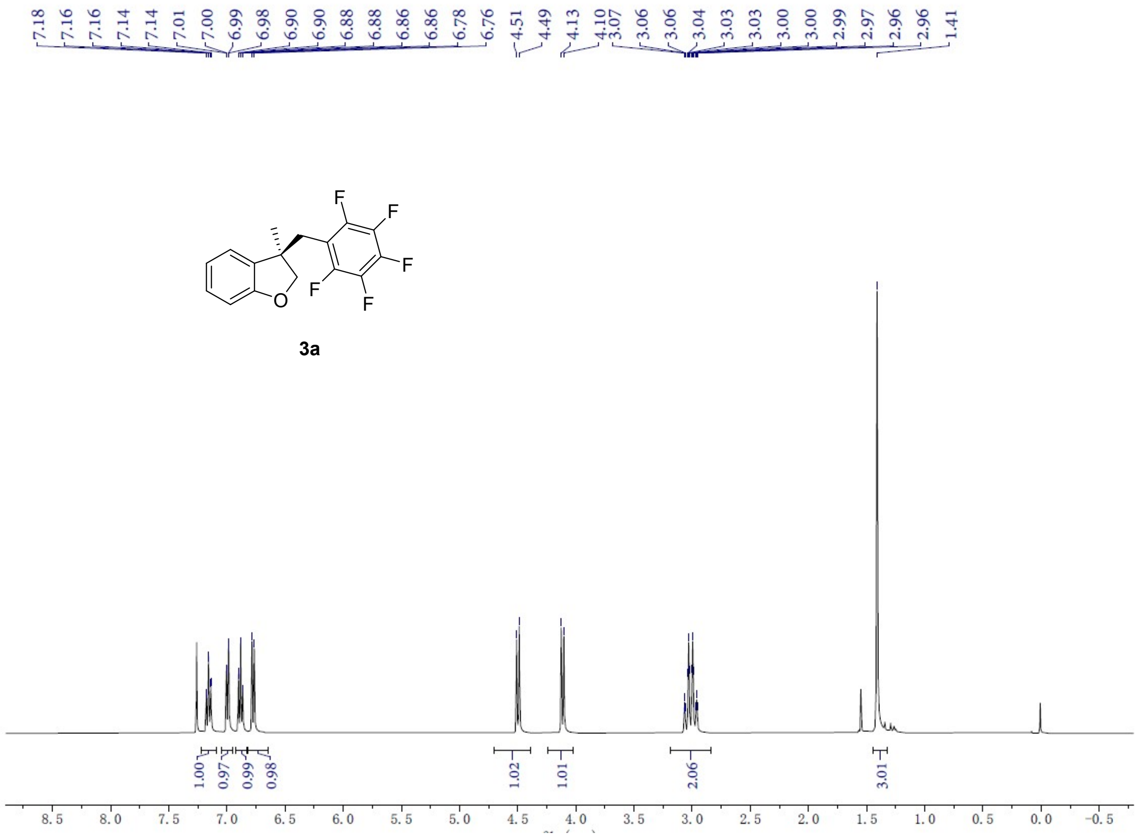

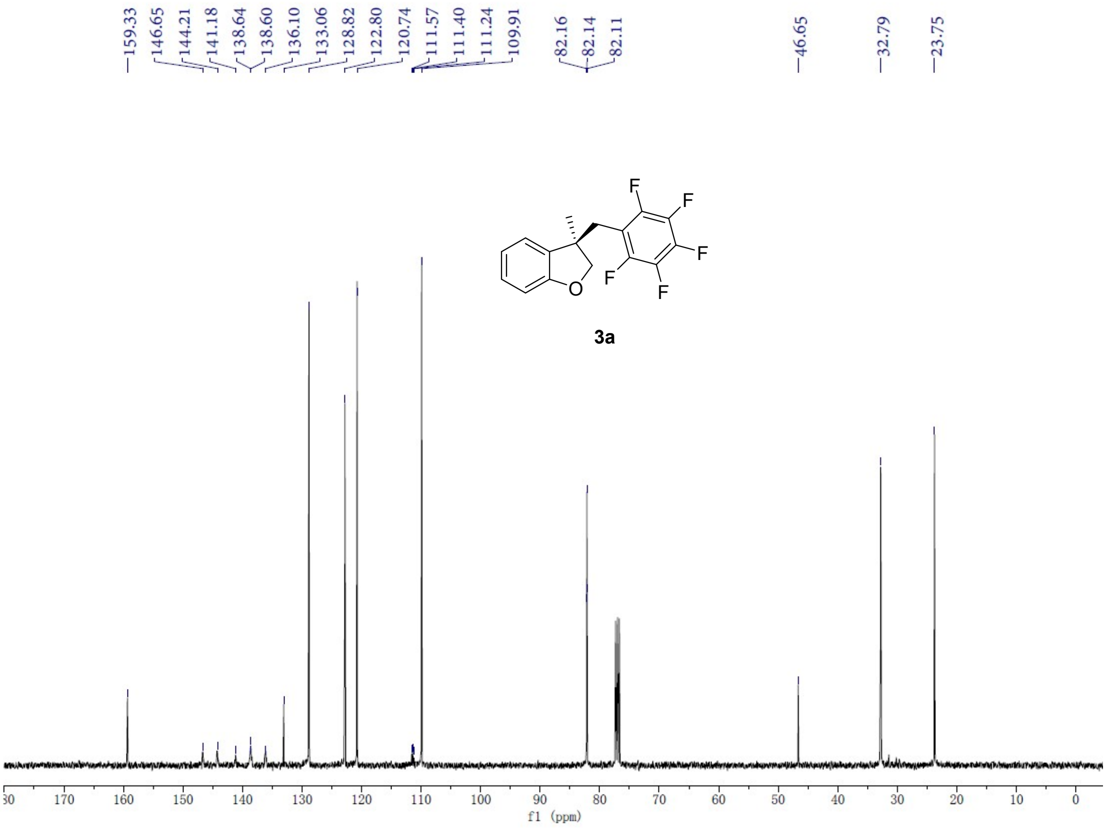
159.33
146.65
144.21
141.18
138.64
138.60
136.10
133.06
128.82
122.80
120.74
111.57
111.40
111.24
109.91
82.16
82.14
82.11
46.65
32.79
23.75
3a
f1 (ppm)

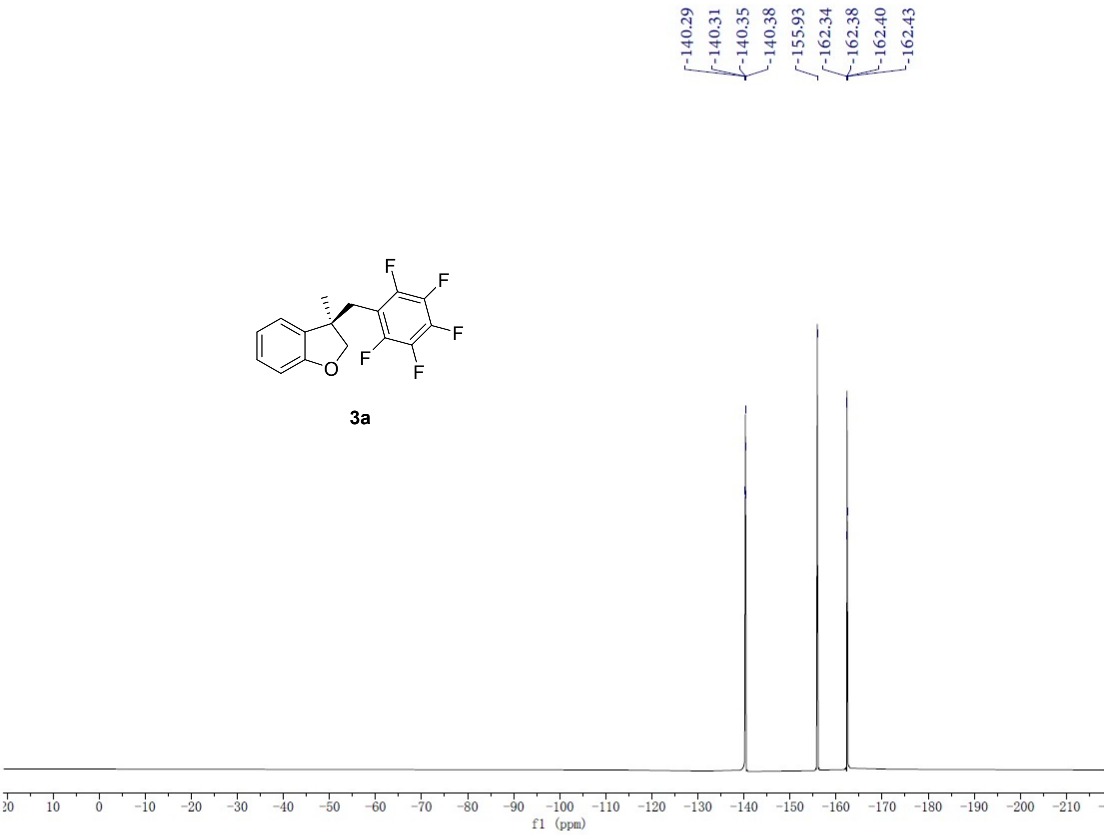
-140.29
-140.31
-140.35
-140.38
-155.93
-162.34
-162.38
-162.40
-162.43
3a
f1 (ppm)

〈Chromatogram〉

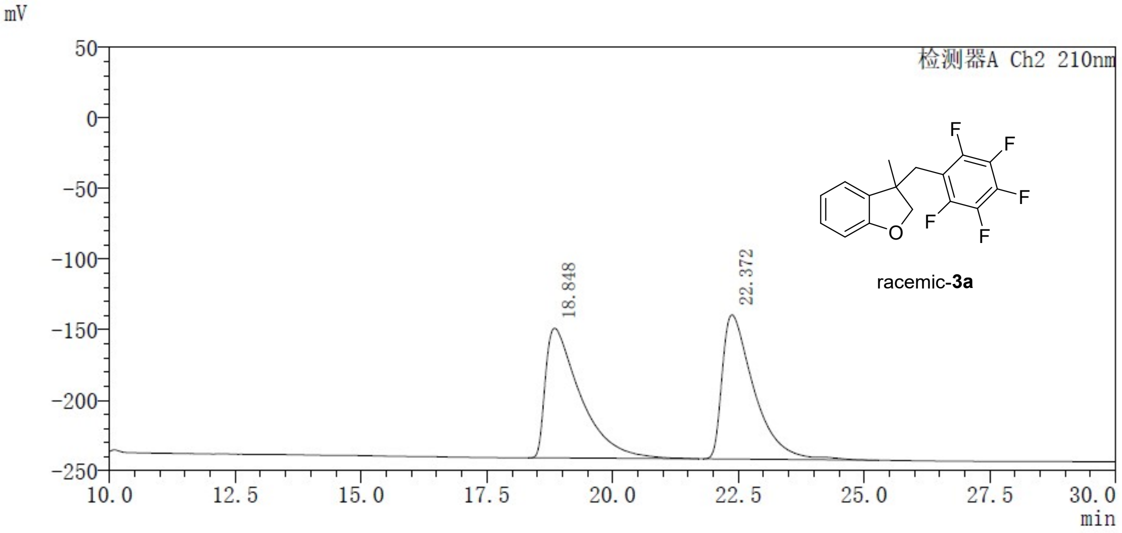

〈Peak Table〉

检测器A Ch2 210nm

No.	Ret. Time (min)	Height	Height%	Area	Area%
1	18.848	91867	47.342	4474509	49.803
2	22.372	102185	52.658	4509948	50.197
Total		194052	100.000	8984457	100.000

〈Chromatogram〉

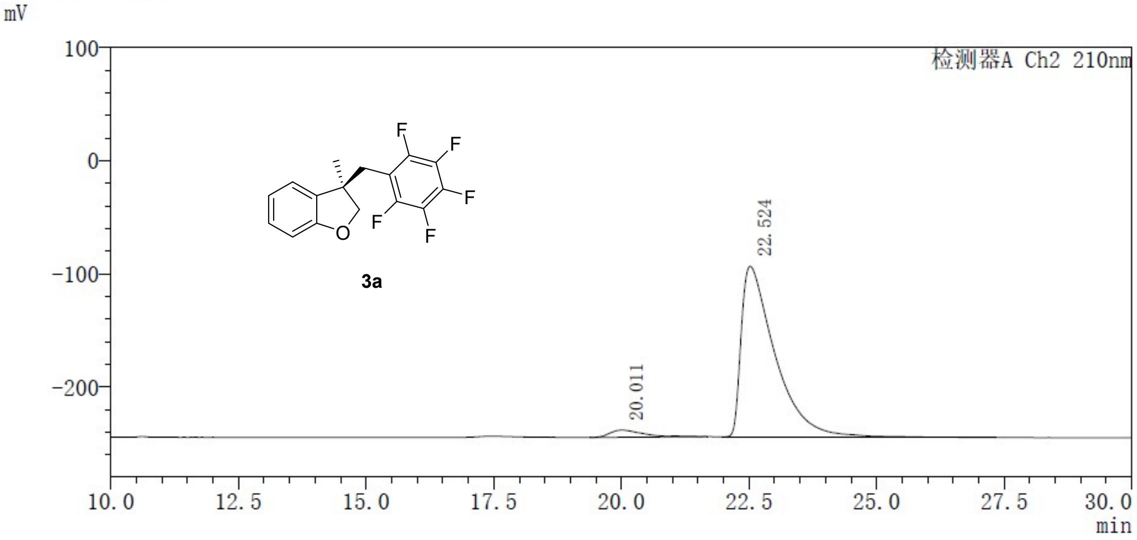

〈Peak Table〉

检测器A Ch2 210nm

No.	Ret. Time (min)	Height	Height%	Area	Area%
1	20.011	6346	4.018	298261	4.040
2	22.524	151585	95.982	7085097	95.960
Total		157931	100.000	7383358	100.000

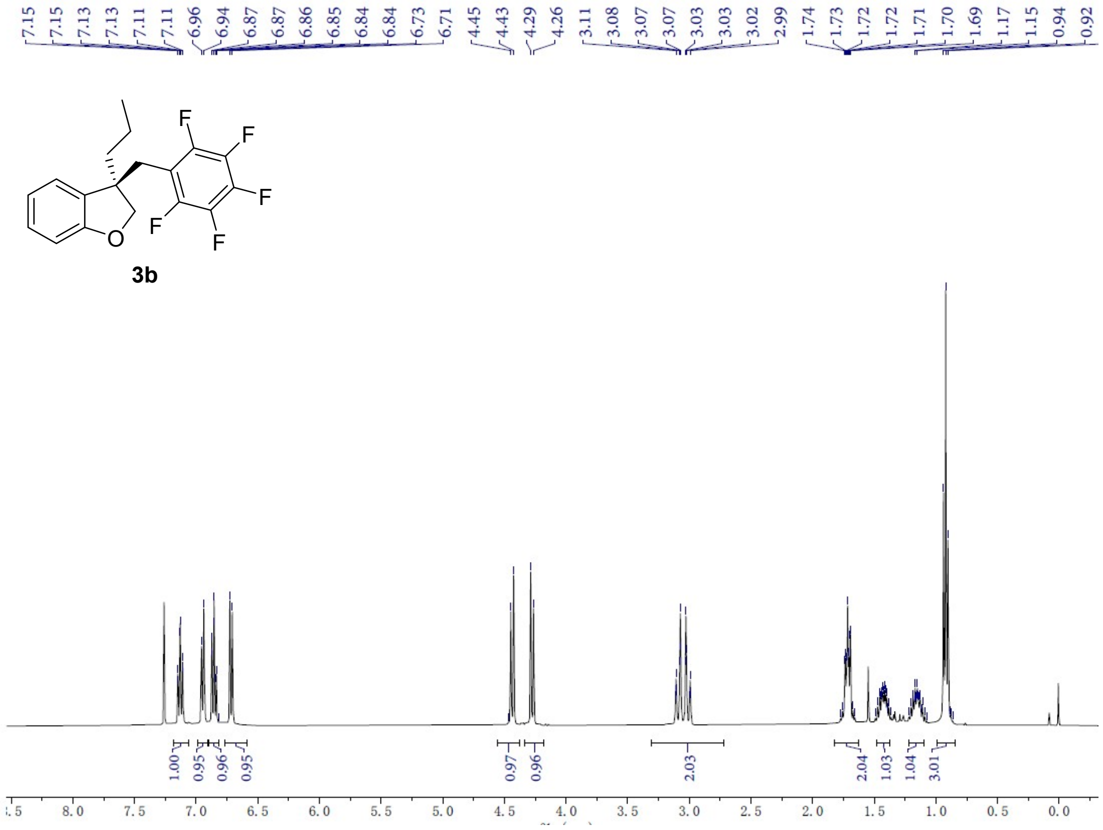
7.15 7.15 7.13 7.13 7.11 7.11 6.96 6.94 6.87 6.87 6.86 6.85 6.84 6.84 6.73 6.71 4.45 4.43 4.29 4.26 3.11 3.08 3.07 3.07 3.03 3.03 3.02 2.99 1.74 1.73 1.72 1.72 1.71 1.70 1.69 1.17 1.15 0.94 0.92
F
F
F
F
F
O
3b
1.00 0.95 0.96 0.95 0.97 0.96 2.03 2.04 1.03 1.04 3.01
.5 8.0 7.5 7.0 6.5 6.0 5.5 5.0 4.5 4.0 3.5 3.0 2.5 2.0 1.5 1.0 0.5 0.0

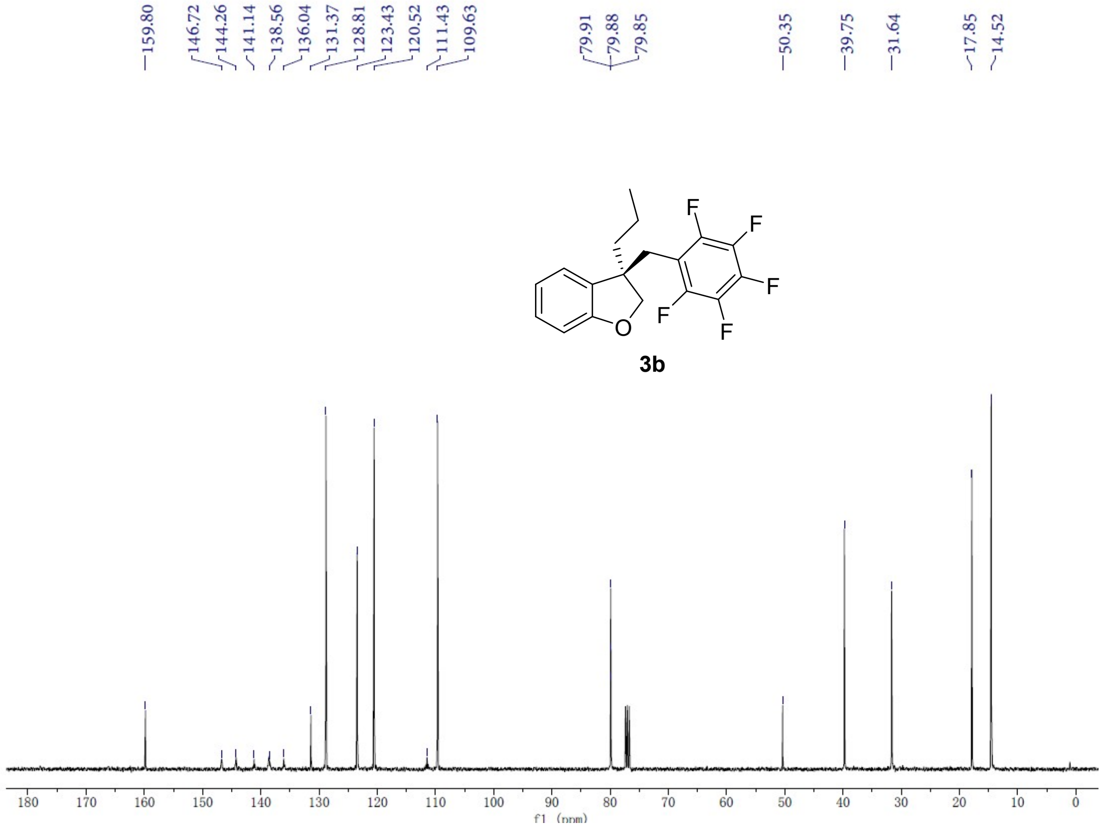
159.80 146.72 144.26 141.14 138.56 136.04 131.37 128.81 123.43 120.52 111.43 109.63 79.91 79.88 79.85 50.35 39.75 31.64 17.85 14.52
F
F
F
F
F
O
3b
180 170 160 150 140 130 120 110 100 90 80 70 60 50 40 30 20 10 0
f1 (ppm)

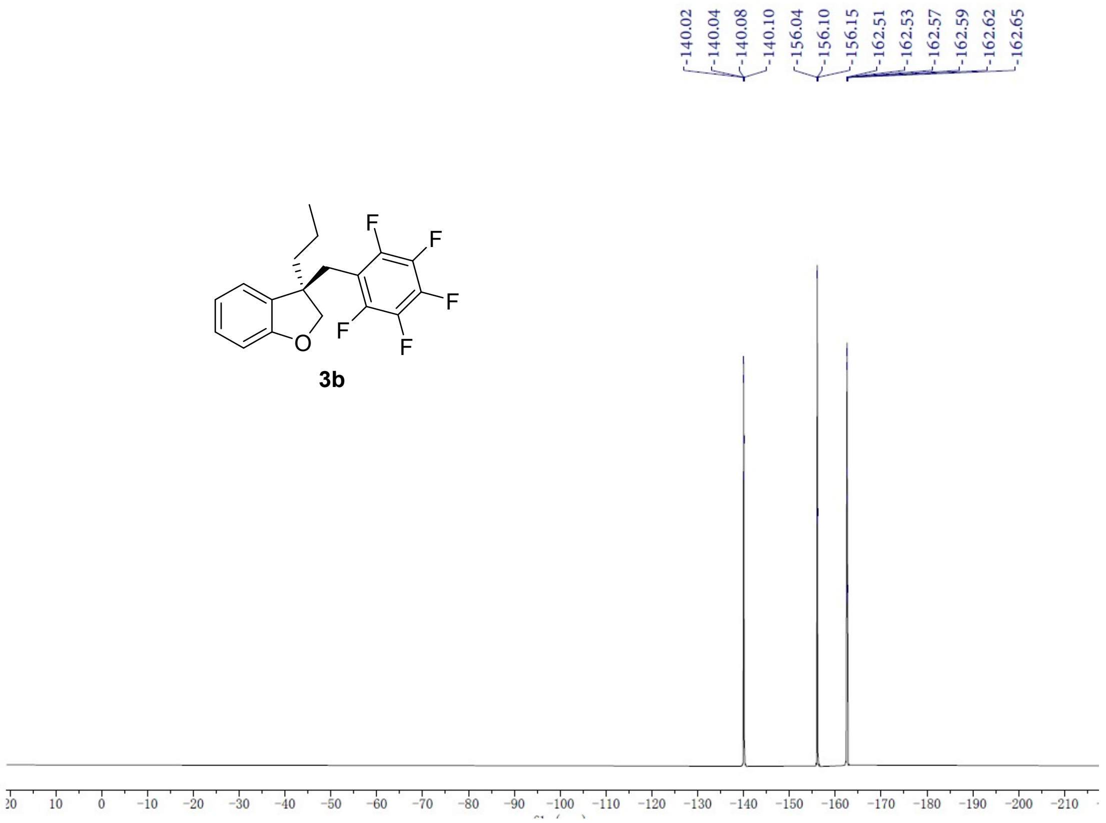

〈Chromatogram〉

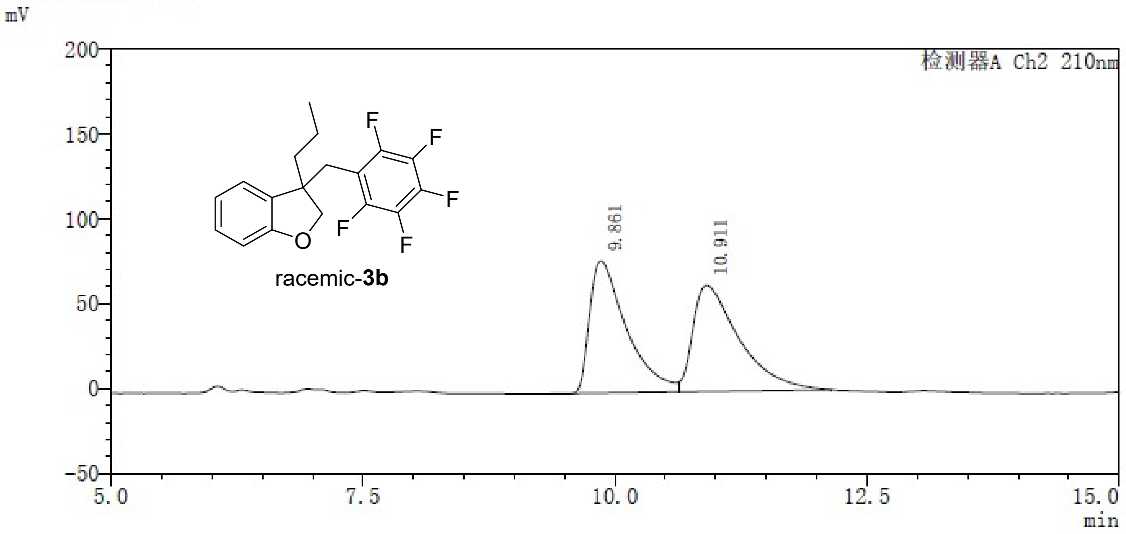

〈Peak Table〉

检测器A Ch2 210nm

No.	Ret. Time (min)	Height	Height%	Area	Area%
1	9.861	77704	55.426	1973809	49.743
2	10.911	62490	44.574	1994177	50.257
Total		140195	100.000	3967987	100.000

<Chromatogram>

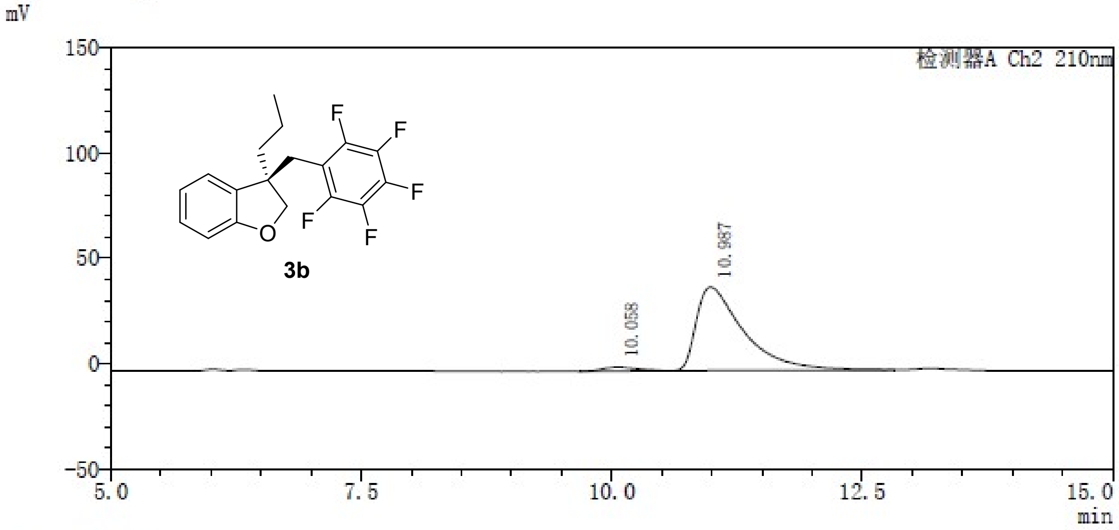

<Peak Table>

检测器A Ch2 210nm

No.	Ret. Time(min)	Height	Height%	Area	Area%
1	10.058	1841	4.455	42794	3.195
2	10.987	39489	95.545	1296548	96.805
Total		41330	100.000	1339343	100.000

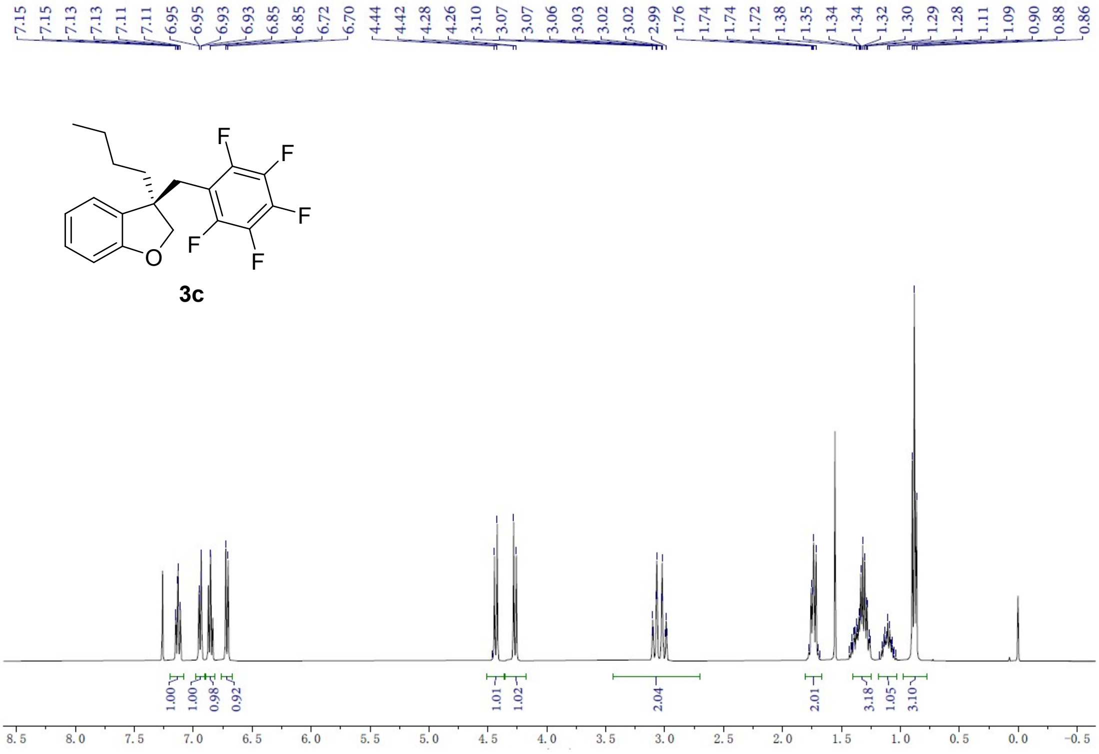

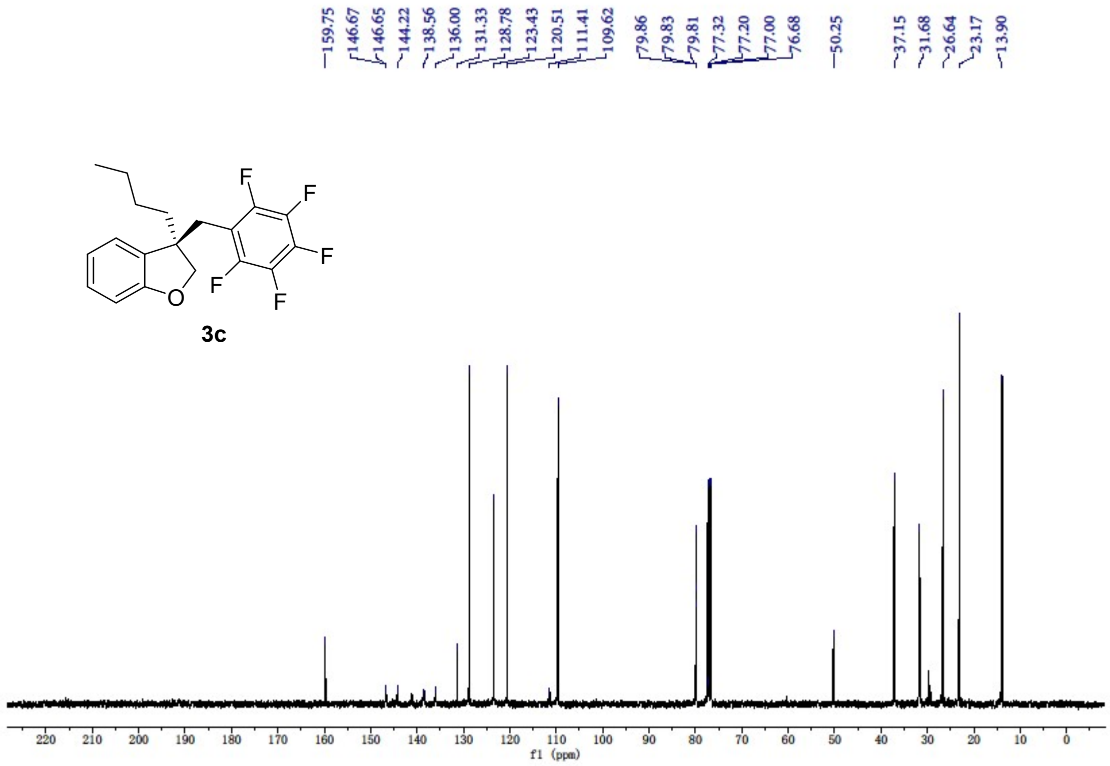
159.75
146.67
146.65
144.22
138.56
136.00
131.33
128.78
123.43
120.51
111.41
109.62
79.86
79.83
79.81
77.32
77.20
77.00
76.68
50.25
37.15
31.68
26.64
23.17
13.90
F
F
F
F
F
O
3c
f1 (ppm)

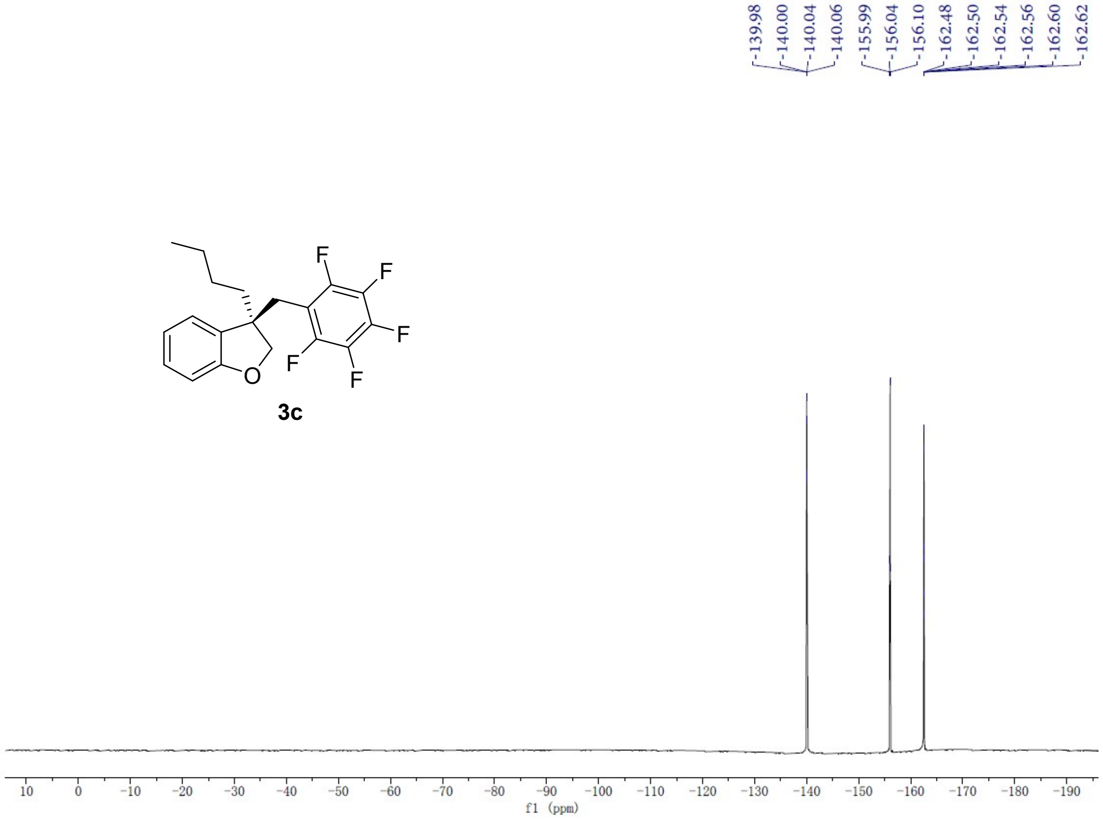
-139.98
-140.00
-140.04
-140.06
-155.99
-156.04
-156.10
-162.48
-162.50
-162.54
-162.56
-162.60
-162.62
F
F
F
F
F
O
3c
f1 (ppm)

<Chromatogram>

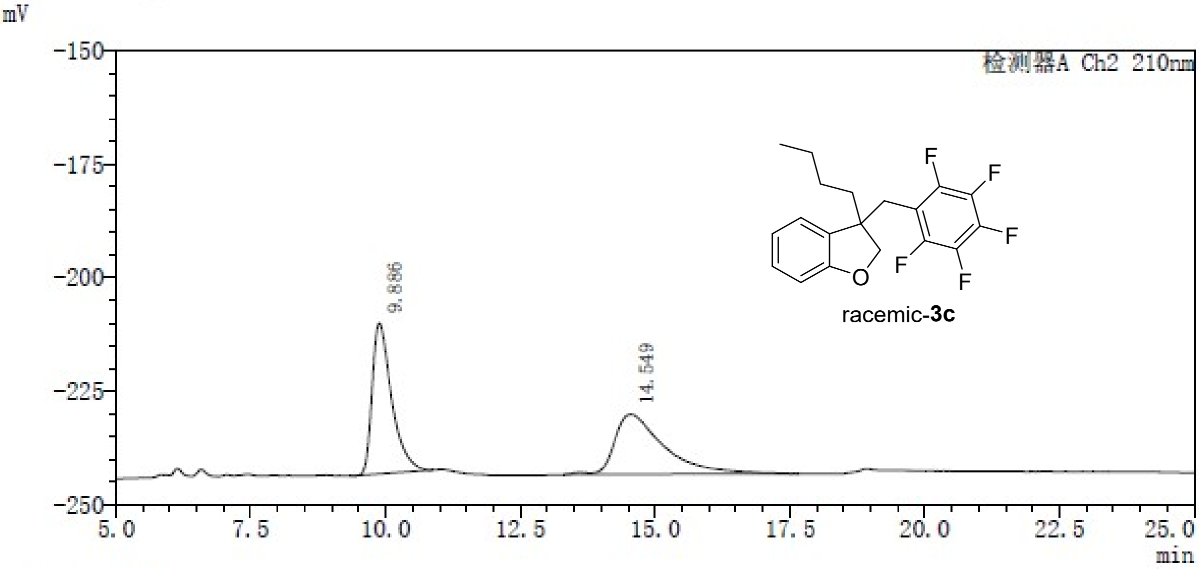

<Peak Table>

检测器A Ch2 210nm

No.	Ret. Time(min)	Height	Height%	Area	Area%
1	9.886	33271	71.617	839602	49.846
2	14.549	13186	28.383	844799	50.154
Total		46456	100.000	1684401	100.000

<Chromatogram>

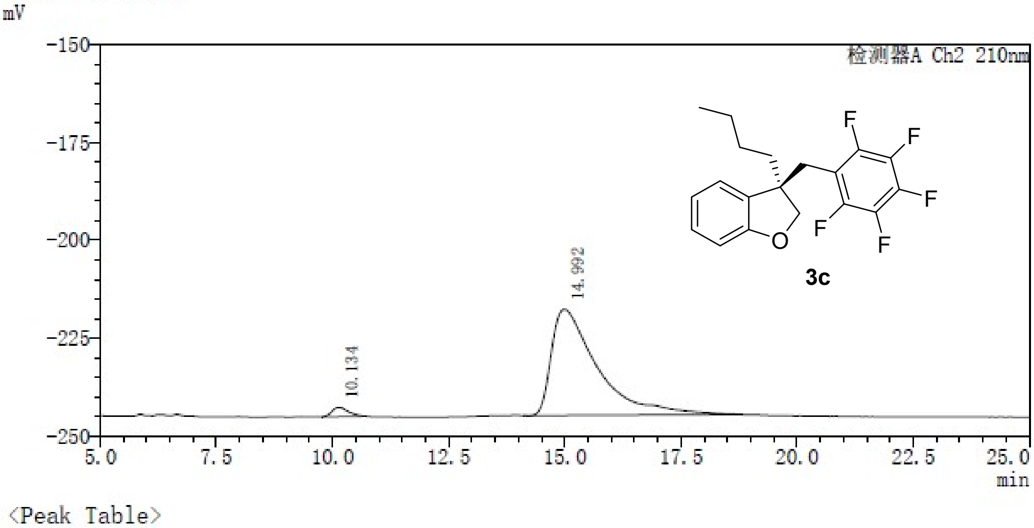

<Peak Table>

检测器A Ch2 210nm

No.	Ret. Time(min)	Height	Height%	Area	Area%
1	10.134	2374	8.059	57603	2.998
2	14.992	27082	91.941	1863609	97.002
Total		29455	100.000	1921212	100.000

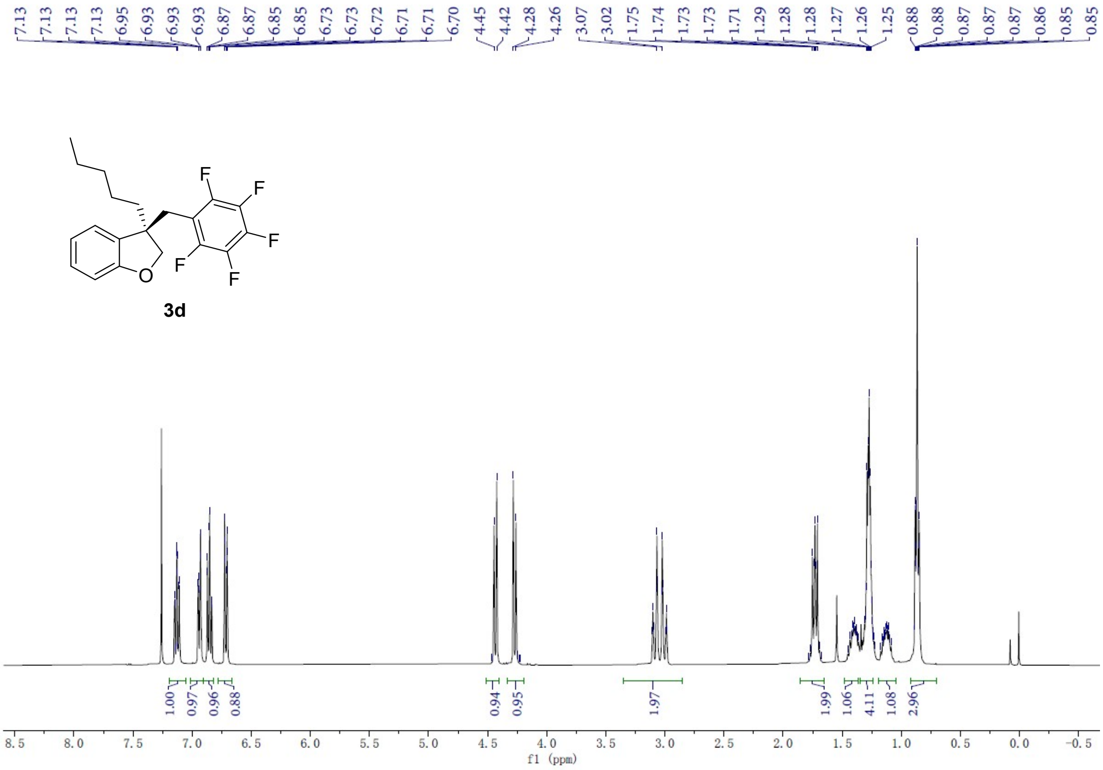
7.13 7.13 7.13 7.13 6.95 6.93 6.93 6.93 6.87 6.87 6.85 6.85 6.73 6.73 6.72 6.71 6.71 6.70 4.45 4.42 4.28 4.26 3.07 3.02 1.75 1.74 1.73 1.73 1.71 1.29 1.28 1.28 1.27 1.26 1.25 0.88 0.88 0.87 0.87 0.87 0.86 0.85 0.85
F
F
F
F
F
O
3d
1.00
0.97
0.96
0.88
0.94
0.95
1.97
1.99
1.06
4.11
1.08
2.96
8.5 8.0 7.5 7.0 6.5 6.0 5.5 5.0 4.5 4.0 3.5 3.0 2.5 2.0 1.5 1.0 0.5 0.0 -0.5
f1 (ppm)

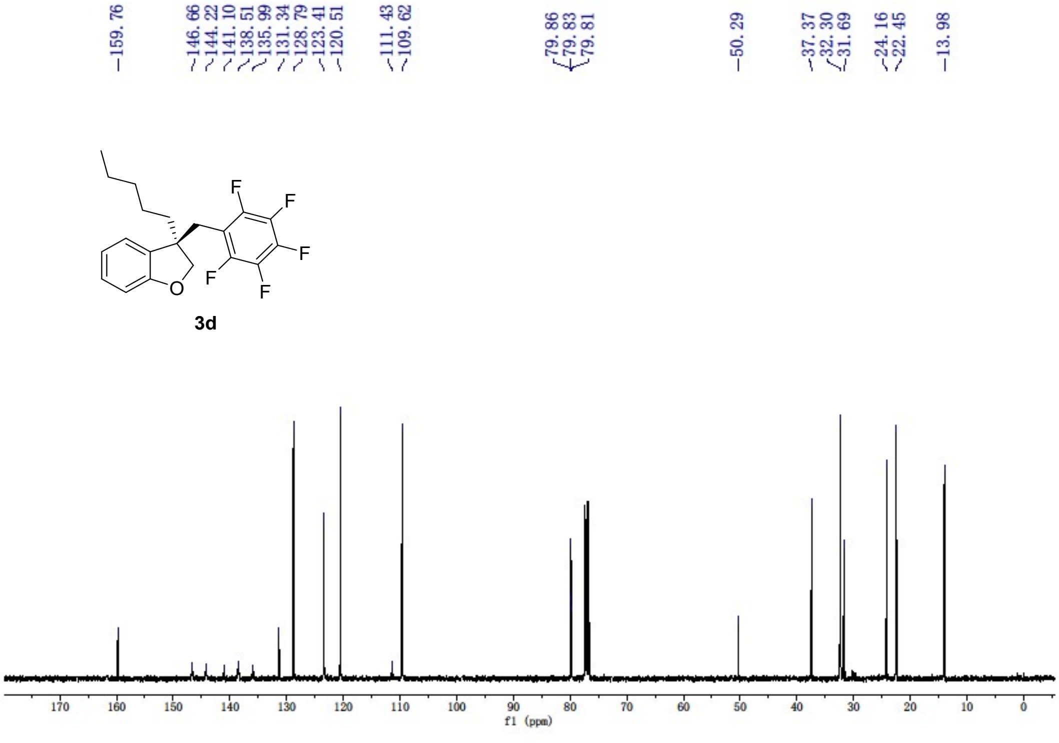
159.76
146.66 144.22 141.10 138.51 135.99 131.34 128.79 123.41 120.51
111.43 109.62
79.86 79.83 79.81
50.29
37.37 32.30 31.69
24.16 22.45
13.98
F
F
F
F
F
O
3d
170 160 150 140 130 120 110 100 90 80 70 60 50 40 30 20 10 0
f1 (ppm)

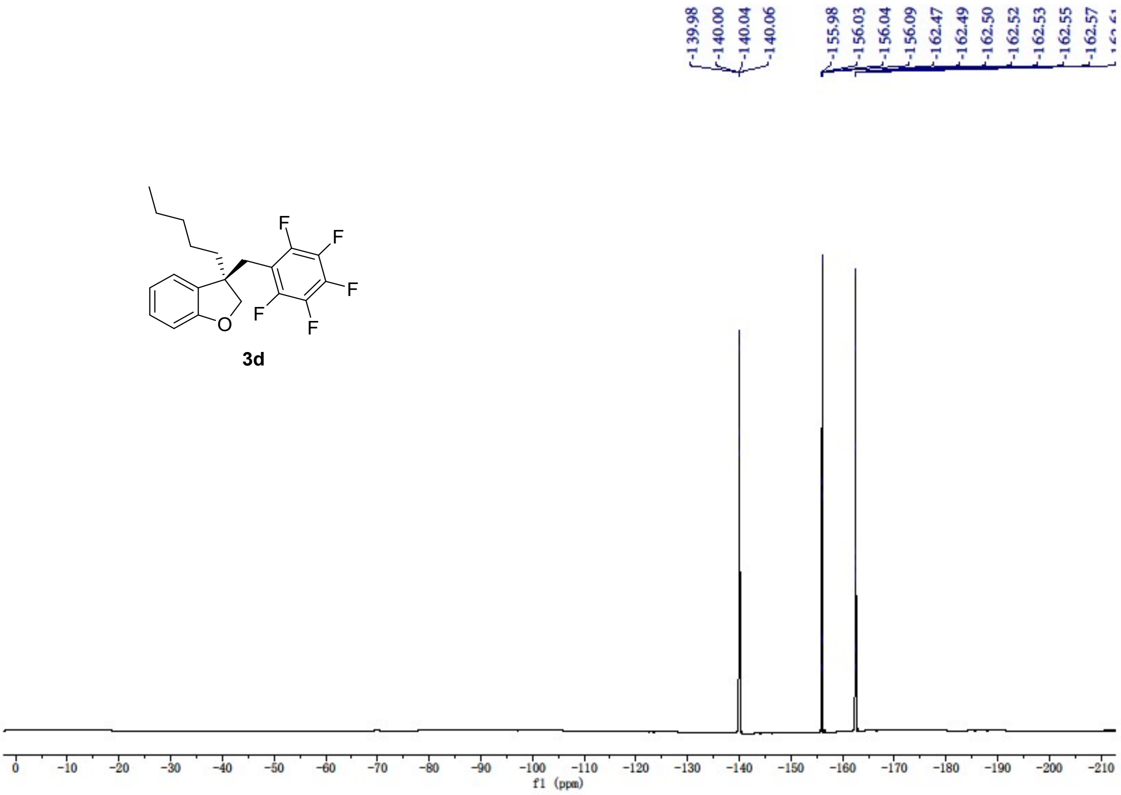

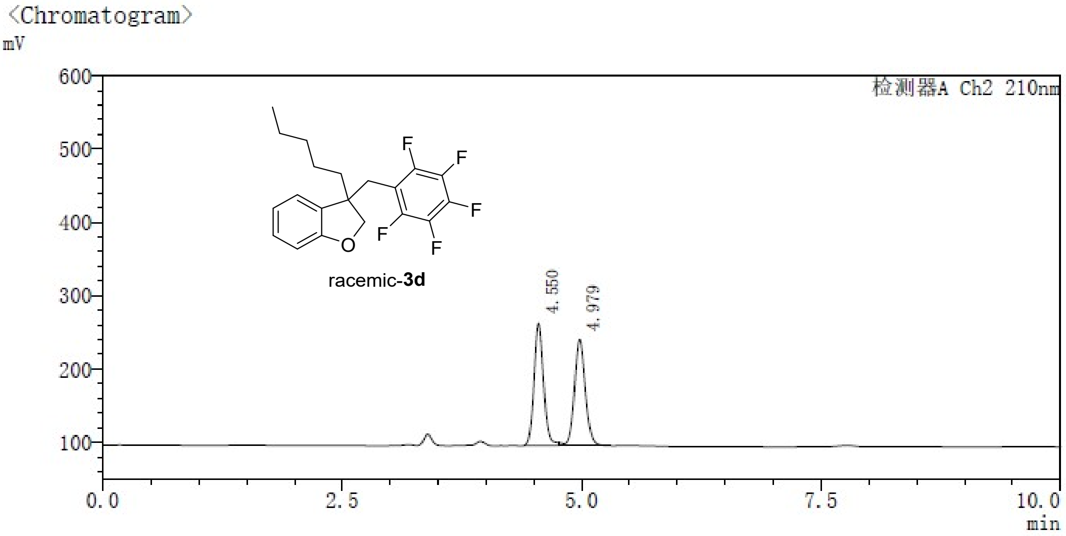

<Peak Table>

检测器A Ch2 210nm

No.	Ret. Time (min)	Height	Height%	Area	Area%
1	4.550	167029	53.558	1168686	50.180
2	4.979	144836	46.442	1160303	49.820
Total		311865	100.000	2328989	100.000

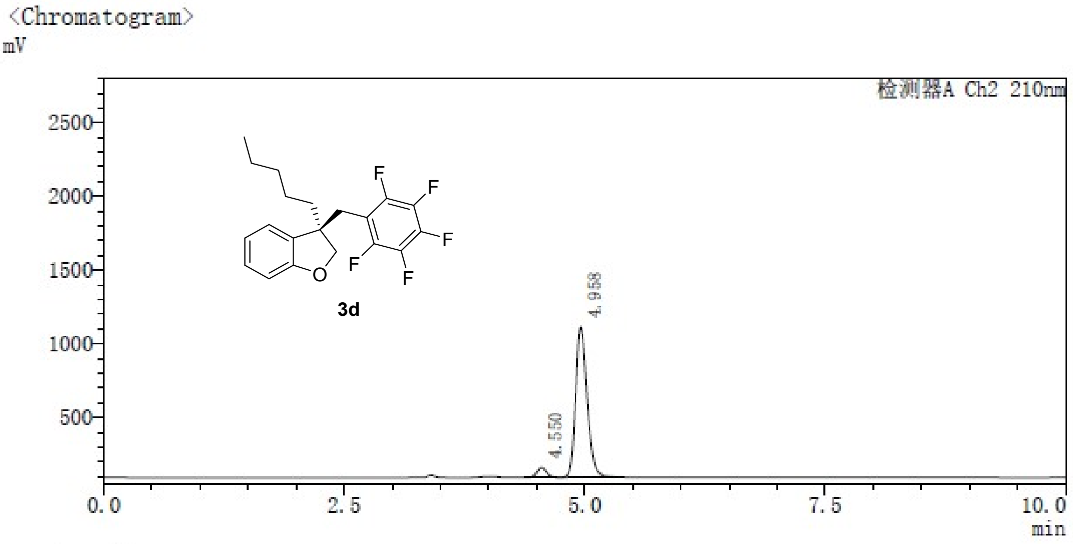

<Peak Table>
检测器A Ch2 210nm

No.	Ret. Time(min)	Height	Height%	Area	Area%
1	4.550	65557	6.049	431049	4.869
2	4.958	1018245	93.951	8422027	95.131
Total		1083802	100.000	8853076	100.000

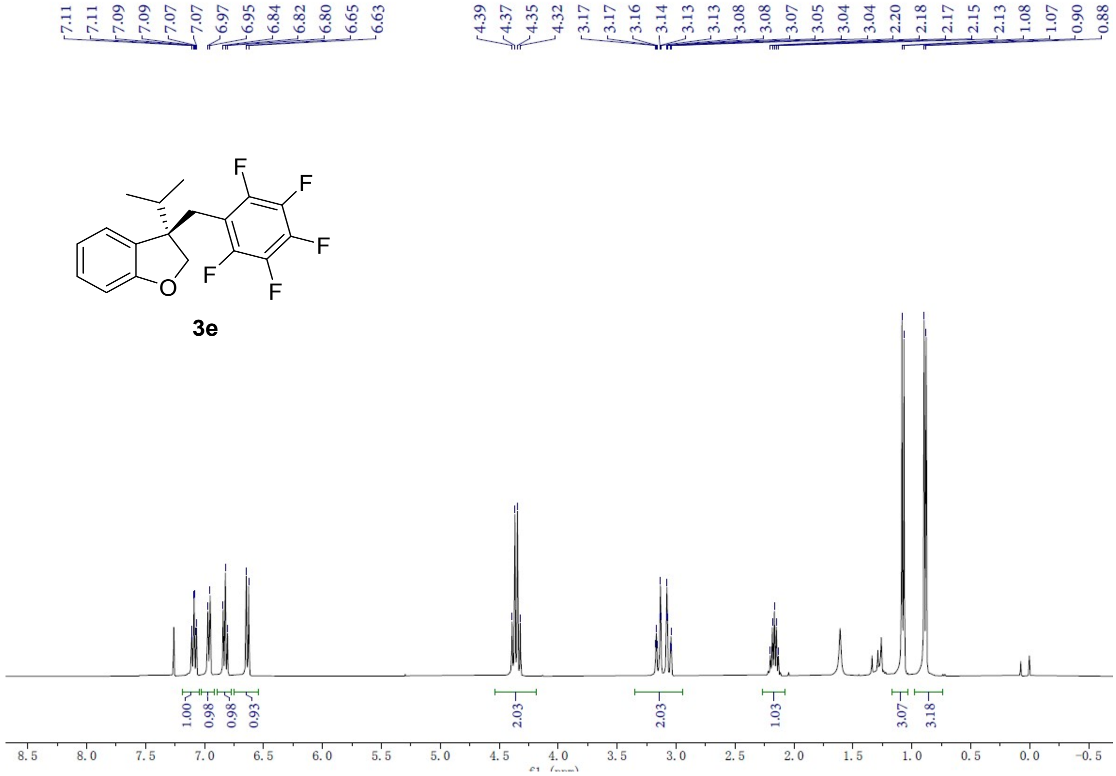

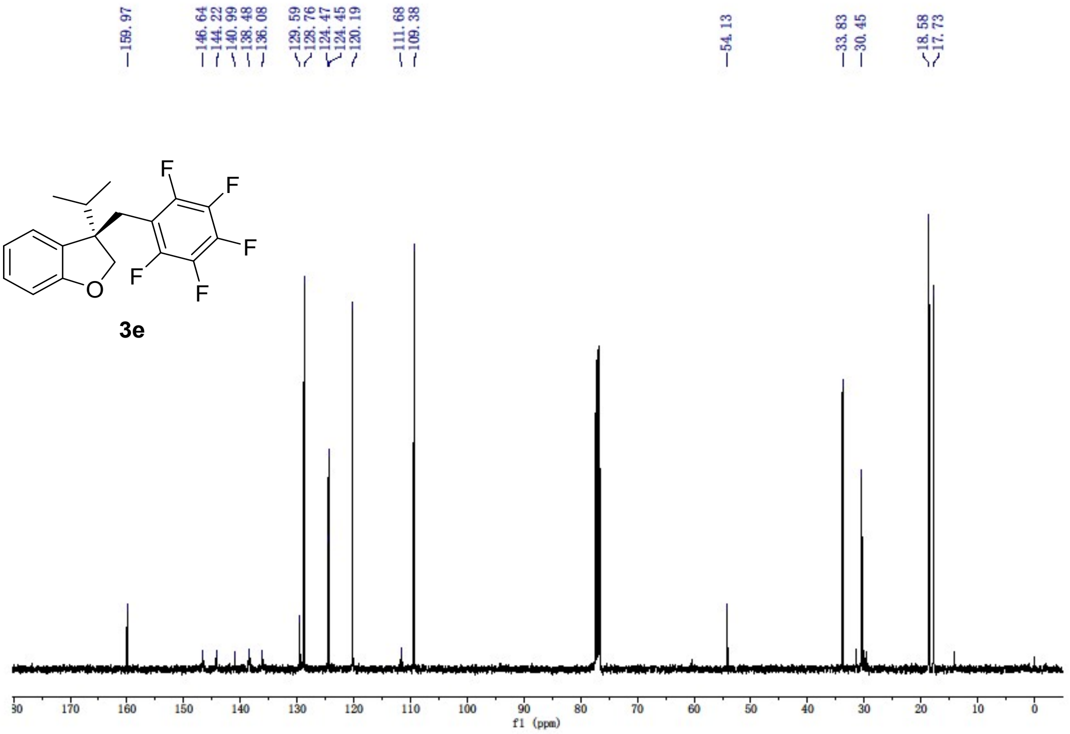
159.97
146.64
144.22
140.99
138.48
136.08
129.59
128.76
124.47
124.45
120.19
111.68
109.38
54.13
33.83
30.45
18.58
17.73
F
F
F
F
F
O
3e
f1 (ppm)

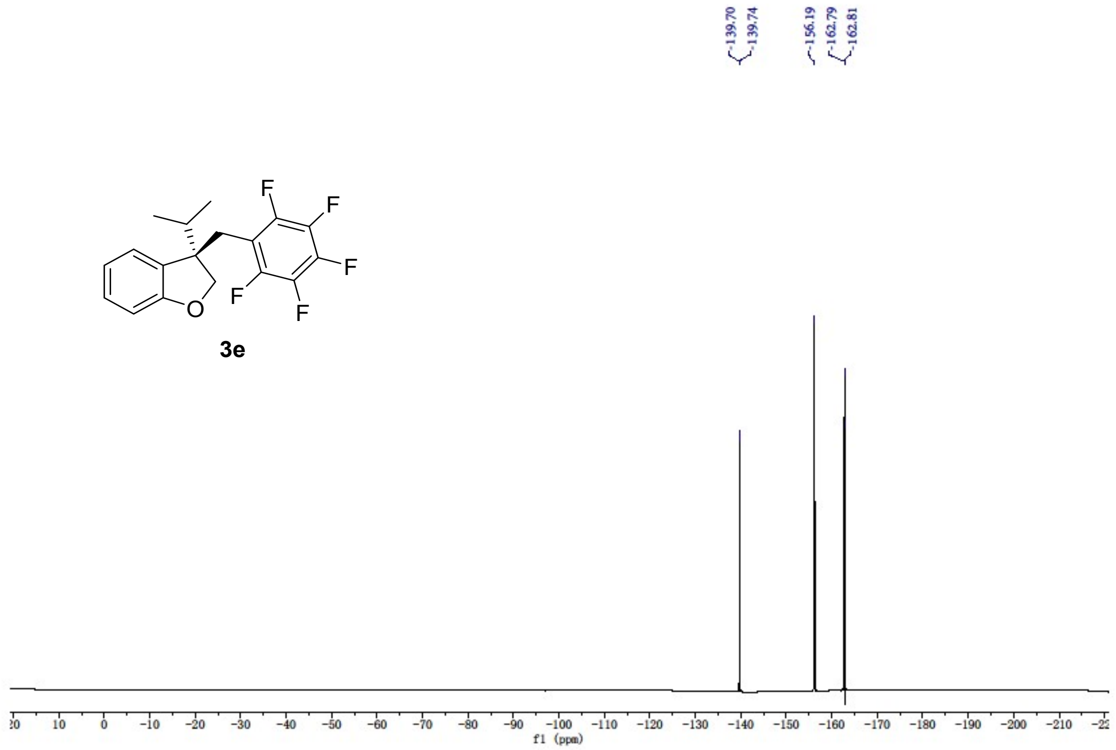
-139.70
-139.74
-156.19
-162.79
-162.81
F
F
F
F
F
O
3e
f1 (ppm)

<Chromatogram>

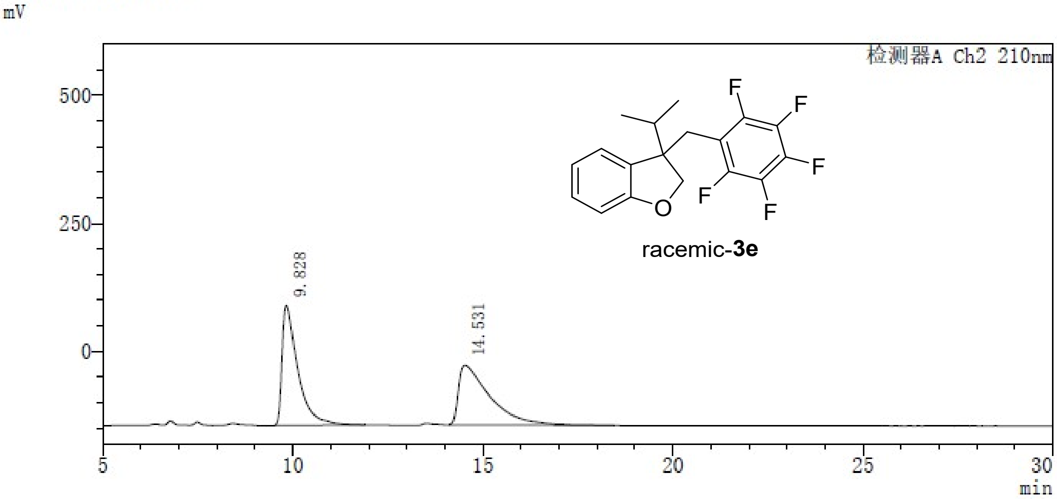

<Peak Table>

检测器A Ch2 210nm

No.	Ret. Time(min)	Height	Height%	Area	Area%
1	9.828	234376	66.839	6768372	50.205
2	14.531	116279	33.161	6713205	49.795
Total		350655	100.000	13481577	100.000

<Chromatogram>

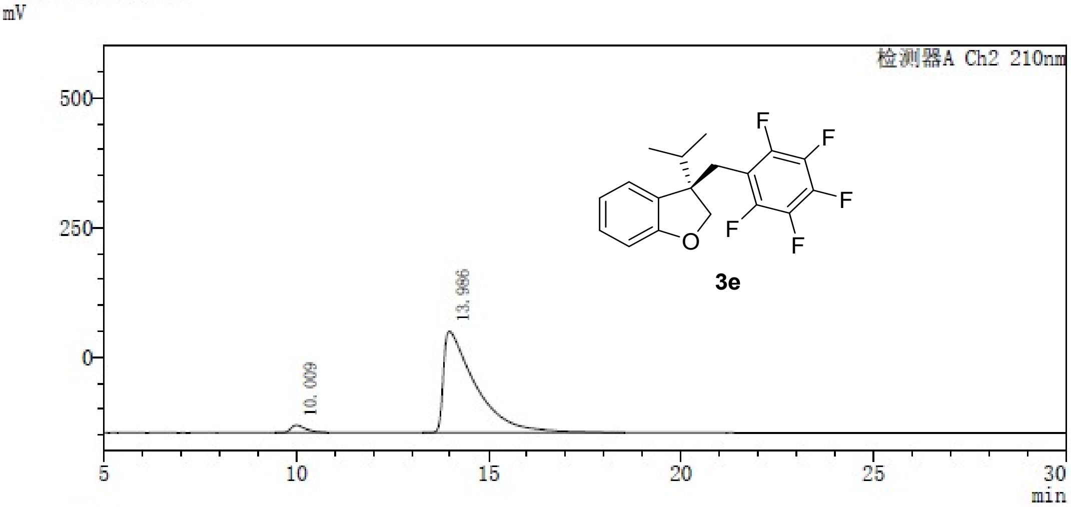

<Peak Table>

检测器A Ch2 210nm

No.	Ret. Time(min)	Height	Height%	Area	Area%
1	10.009	14282	6.821	376417	3.203
2	13.986	195102	93.179	11376753	96.797
Total		209384	100.000	11753171	100.000

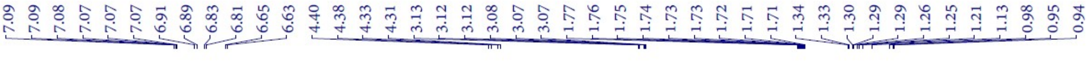
7.09
7.09
7.08
7.07
7.07
7.07
6.91
6.89
6.83
6.81
6.65
6.63
4.40
4.38
4.33
4.31
3.13
3.12
3.12
3.08
3.07
3.07
1.77
1.76
1.75
1.74
1.73
1.73
1.72
1.71
1.71
1.34
1.33
1.30
1.29
1.29
1.26
1.25
1.21
1.13
0.98
0.95
0.94

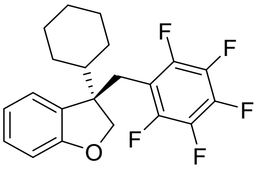
F
F
F
F
F
O

3f

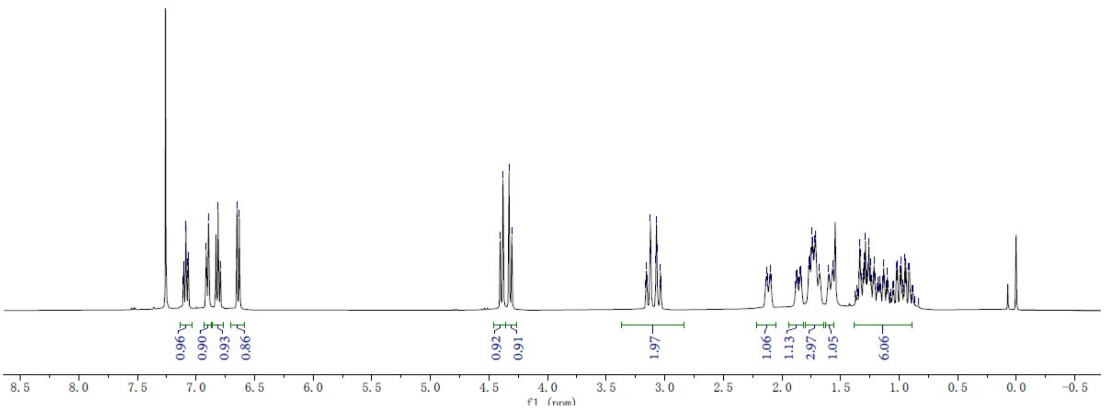
0.96
0.90
0.93
0.86
0.92
0.91
1.97
1.06
1.13
2.97
1.05
6.06
f1 (ppm)

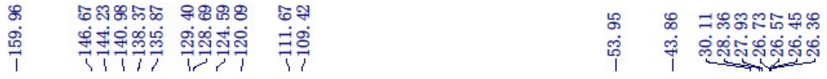
159.96
146.67
144.23
140.98
138.37
135.87
129.40
128.69
124.59
120.09
111.67
109.42
53.95
43.86
30.11
28.36
27.93
26.73
26.57
26.45
26.36

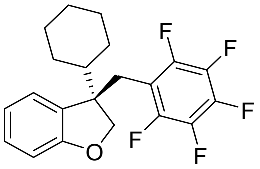
F
F
F
F
F
O

3f

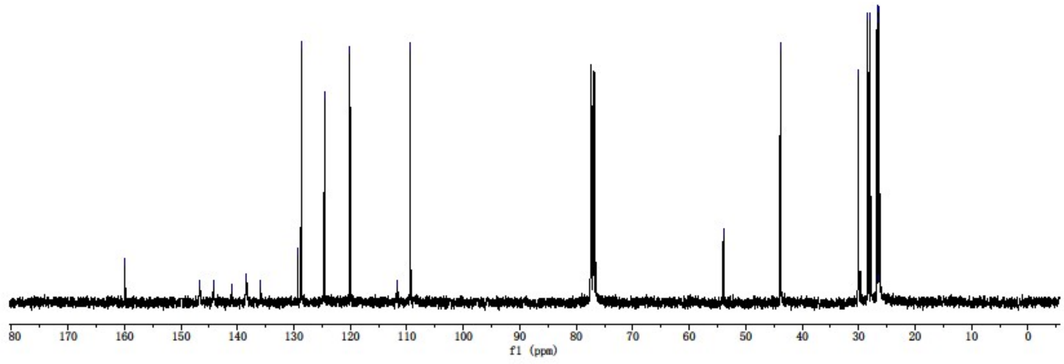
f1 (ppm)

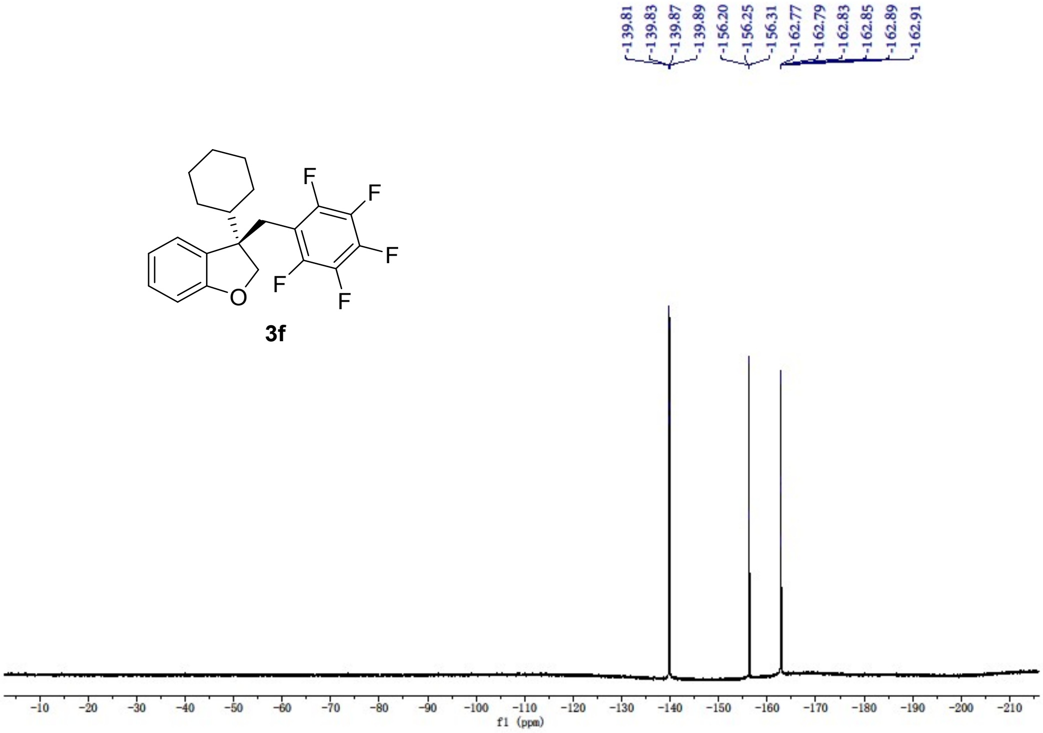

<Chromatogram>

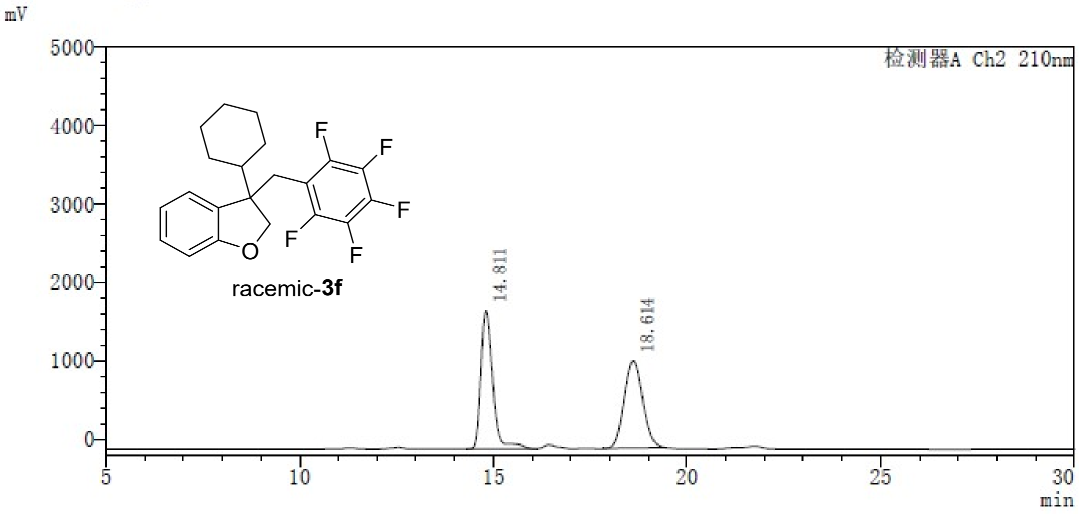

<Peak Table>

检测器A Ch2 210nm

No.	Ret. Time (min)	Height	Height%	Area	Area%
1	14.811	1760480	61.359	37711092	50.367
2	18.614	1108656	38.641	37161924	49.633
Total		2869136	100.000	74873017	100.000

<Chromatogram>

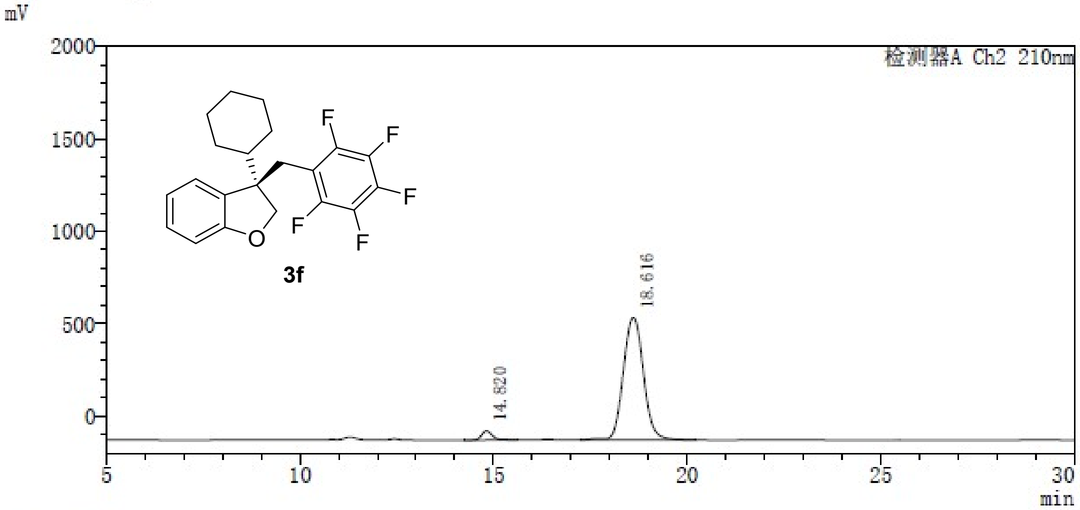

<Peak Table>

检测器A Ch2 210nm

No.	Ret. Time (min)	Height	Height%	Area	Area%
1	14.820	50514	7.089	977411	3.908
2	18.616	662058	92.911	24036209	96.092
Total		712572	100.000	25013621	100.000

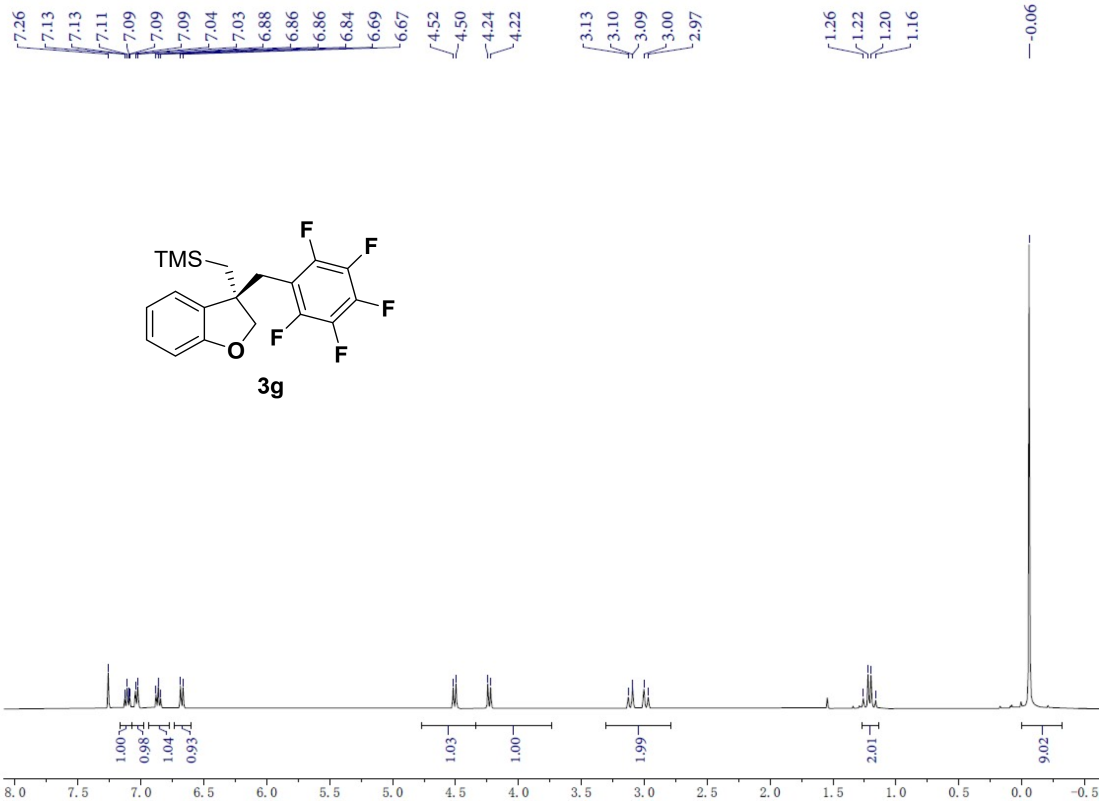

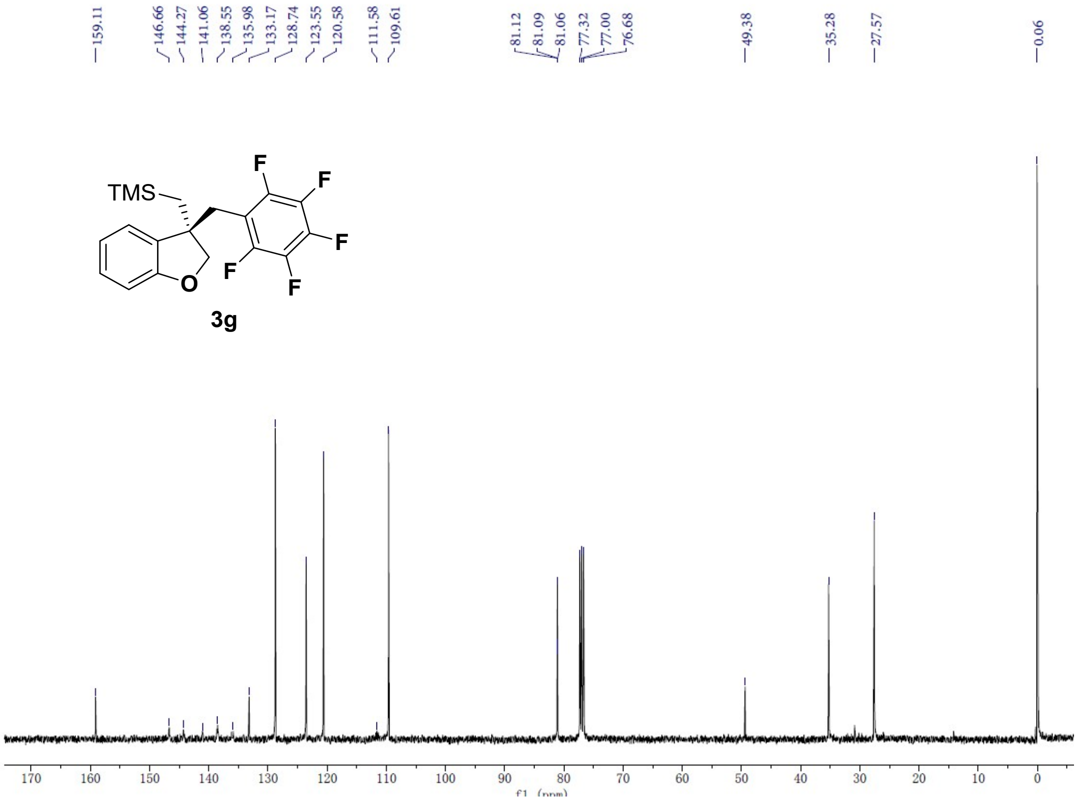
159.11
146.66
144.27
141.06
138.55
135.98
133.17
128.74
123.55
120.58
111.58
109.61
81.12
81.09
81.06
77.32
77.00
76.68
49.38
35.28
27.57
0.06
TMS
F
F
F
F
F
O
3g
170 160 150 140 130 120 110 100 90 80 70 60 50 40 30 20 10 0
f1 (ppm)

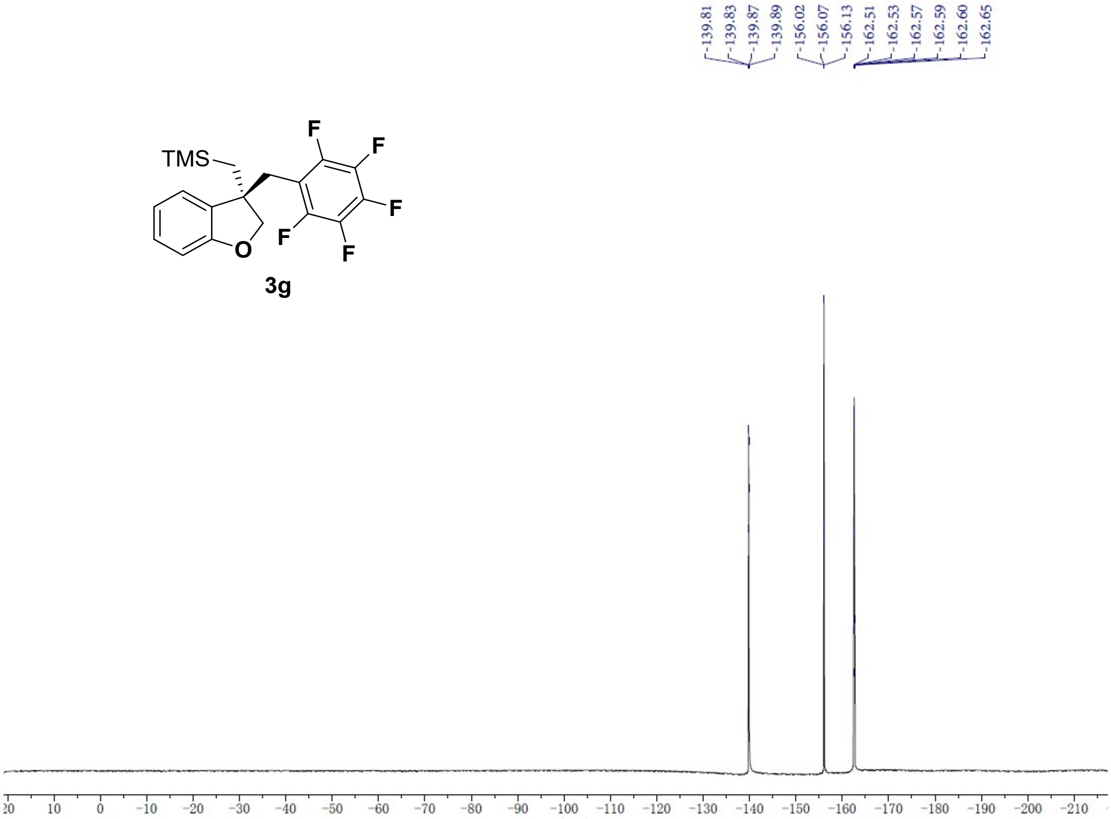
-139.81
-139.83
-139.87
-139.89
-156.02
-156.07
-156.13
-162.51
-162.53
-162.57
-162.59
-162.60
-162.65
TMS
F
F
F
F
F
O
3g
20 10 0 -10 -20 -30 -40 -50 -60 -70 -80 -90 -100 -110 -120 -130 -140 -150 -160 -170 -180 -190 -200 -210

〈Chromatogram〉

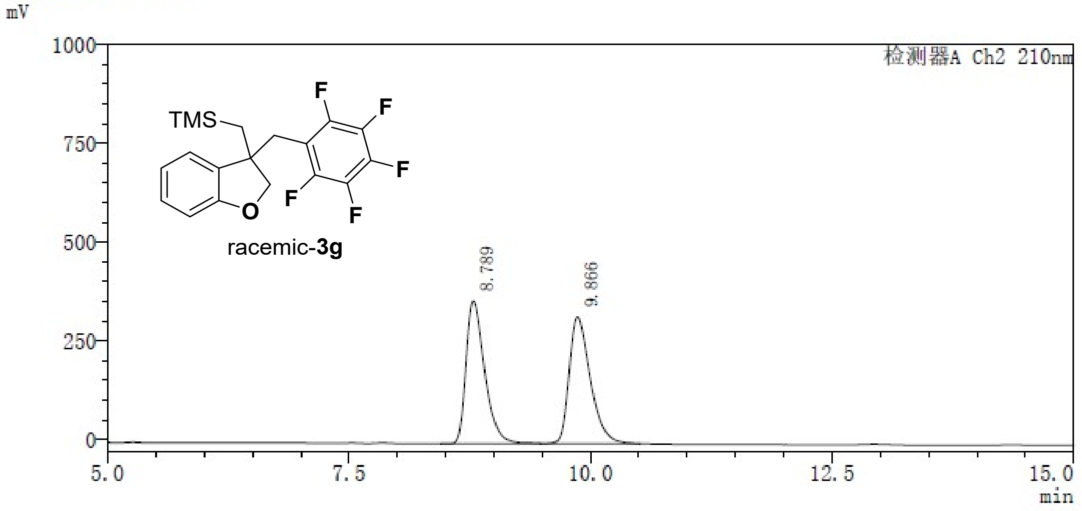

〈Peak Table〉

检测器A Ch2 210nm

No.	Ret. Time (min)	Height	Height%	Area	Area%
1	8.789	361357	53.007	4826023	49.799
2	9.866	320352	46.993	4864993	50.201
Total		681709	100.000	9691016	100.000

〈Chromatogram〉

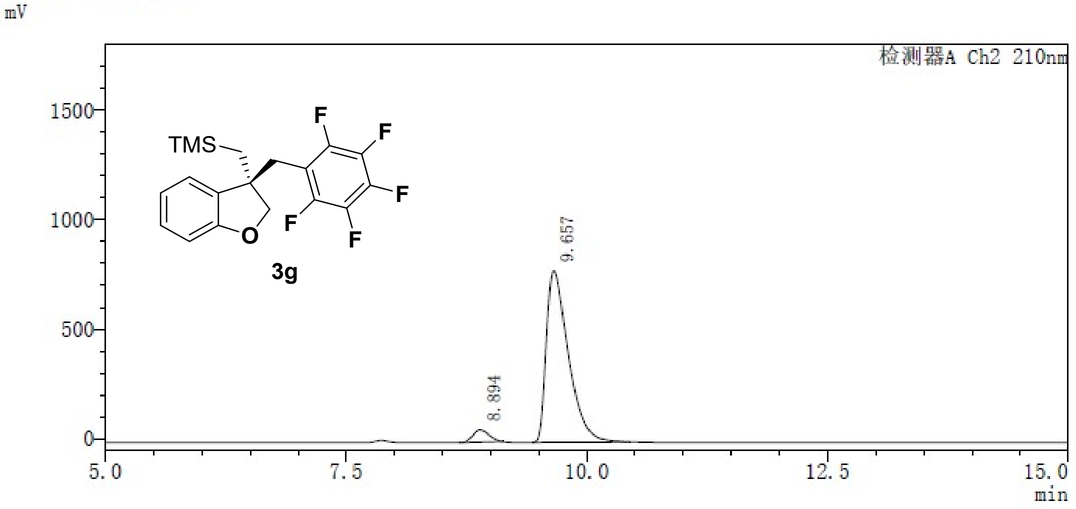

〈Peak Table〉

检测器A Ch2 210nm

No.	Ret. Time (min)	Height	Height%	Area	Area%
1	8.894	55699	6.628	652953	4.964
2	9.657	784612	93.372	12500499	95.036
Total		840310	100.000	13153452	100.000

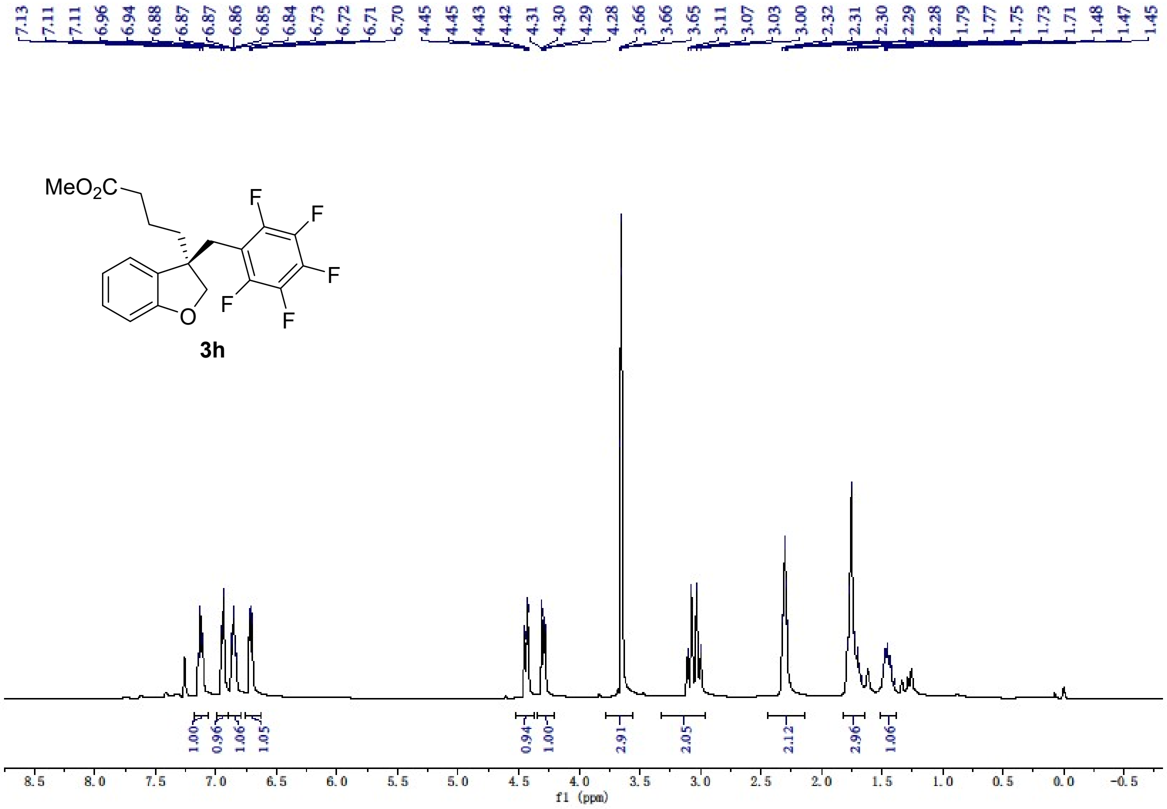
7.13 7.11 7.11 6.96 6.94 6.88 6.87 6.87 6.86 6.85 6.84 6.73 6.72 6.71 6.70 4.45 4.45 4.43 4.42 4.31 4.30 4.29 4.28 3.66 3.66 3.65 3.11 3.07 3.03 3.00 2.32 2.31 2.30 2.29 2.28 1.79 1.77 1.75 1.73 1.71 1.48 1.47 1.45
MeO_2C
F
F
F
F
F
O
3h
1.00 0.96 1.06 1.05 0.94 1.00 2.91 2.05 2.12 2.96 1.06
f1 (ppm)

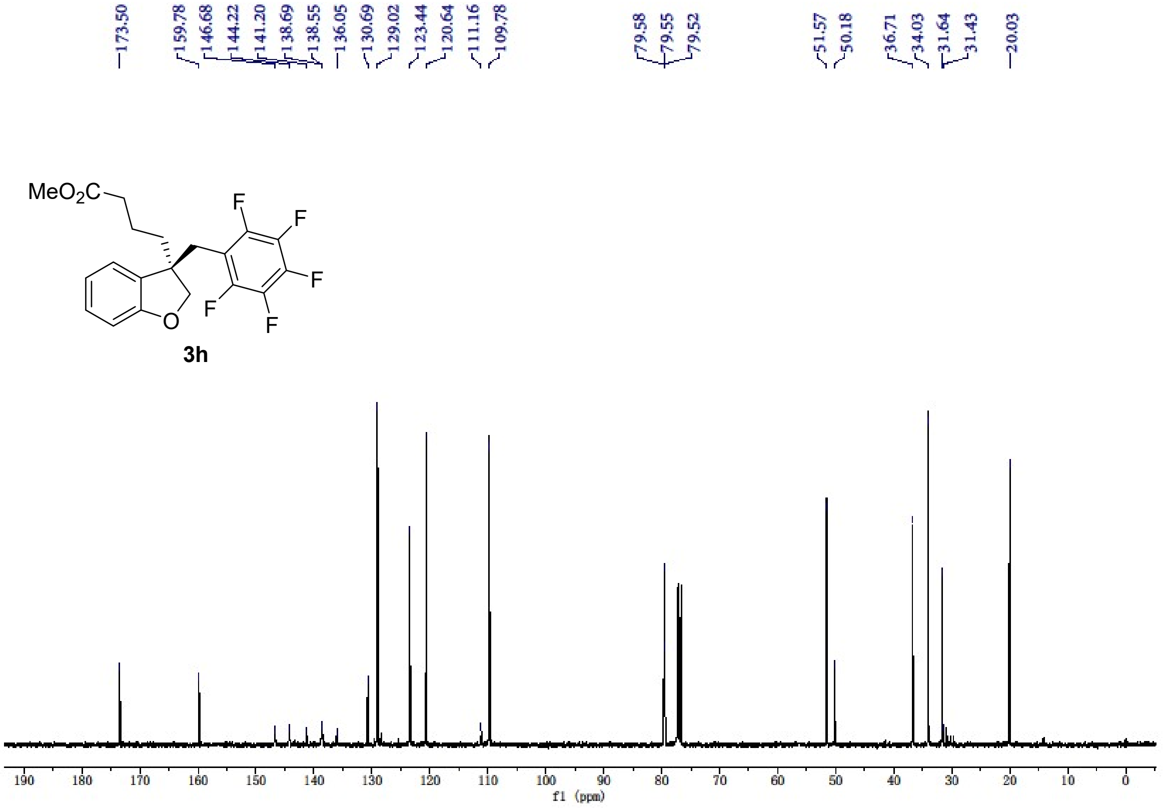
173.50 159.78 146.68 144.22 141.20 138.69 138.55 136.05 130.69 129.02 123.44 120.64 111.16 109.78 79.58 79.55 79.52 51.57 50.18 36.71 34.03 31.64 31.43 20.03
MeO_2C
F
F
F
F
F
O
3h
f1 (ppm)

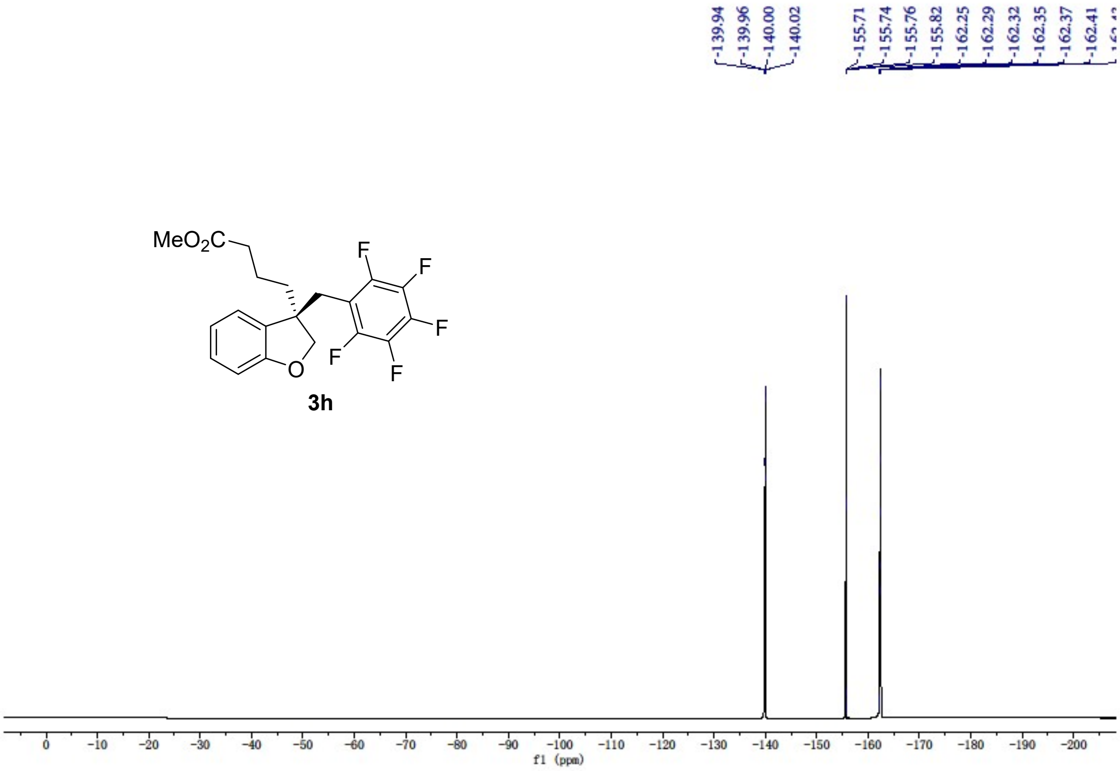

〈Chromatogram〉

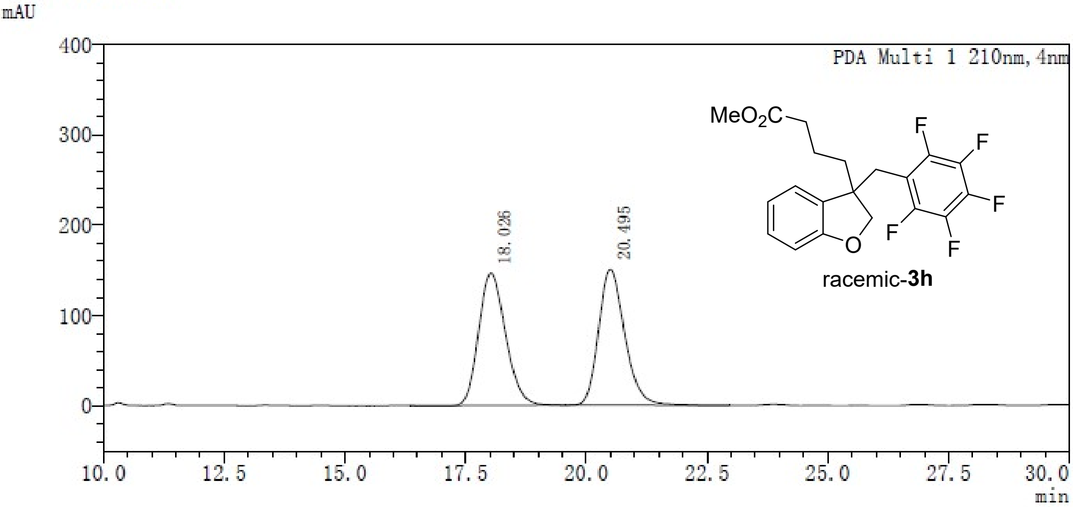

〈Peak Table〉

PDA Ch1 210nm

No.	Ret.Time(min)	Height	Height%	Area	Area%
1	18.026	146415	49.335	5676632	49.981
2	20.495	150362	50.665	5680863	50.019
Total		296777	100.000	11357495	100.000

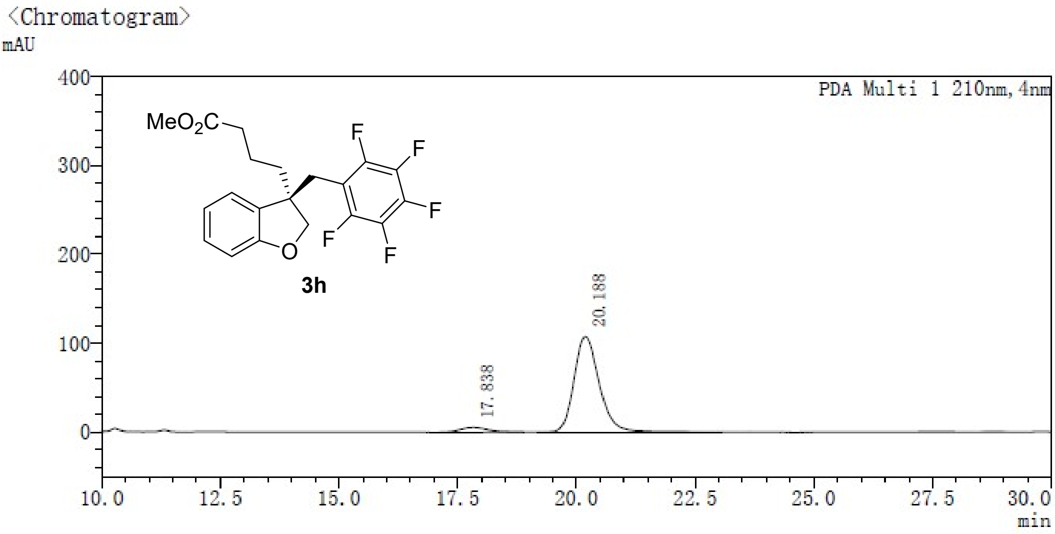

<Peak Table>

PDA Ch1 210nm

No.	Ret. Time (min)	Height	Height%	Area	Area%
1	17.838	5408	4.780	207684	4.979
2	20.188	107726	95.220	3963353	95.021
Total		113133	100.000	4171036	100.000

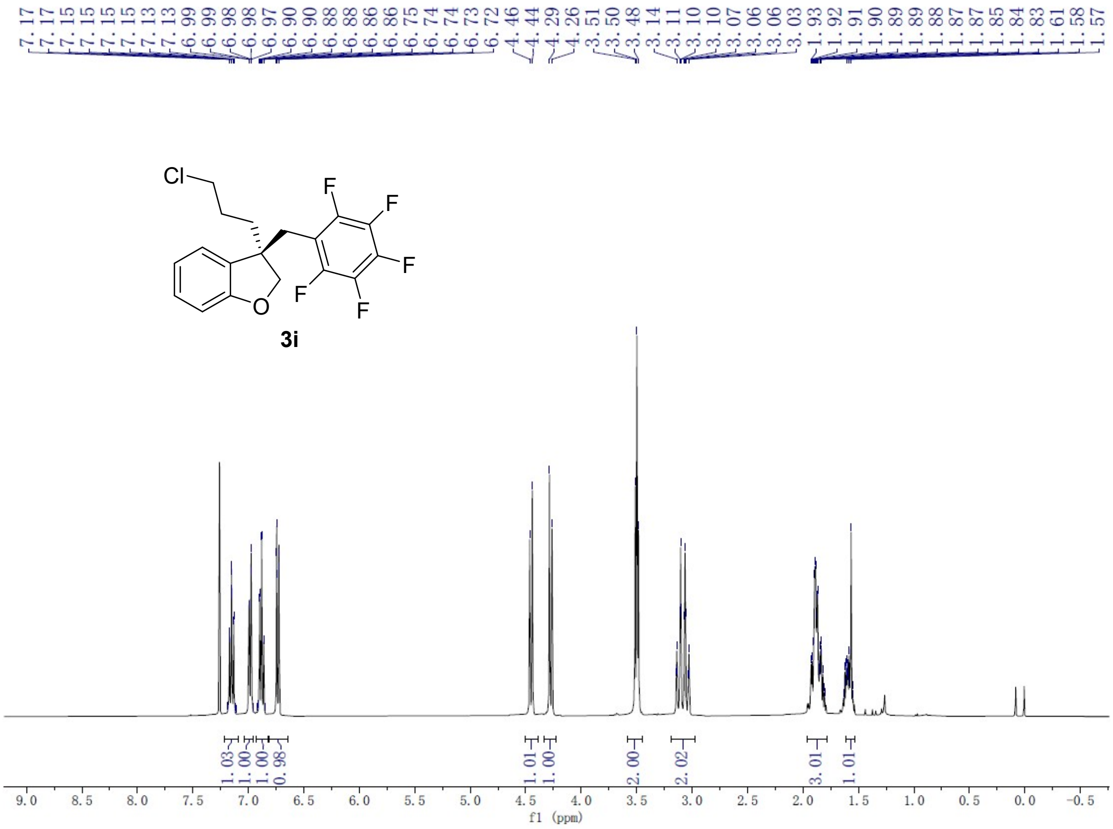

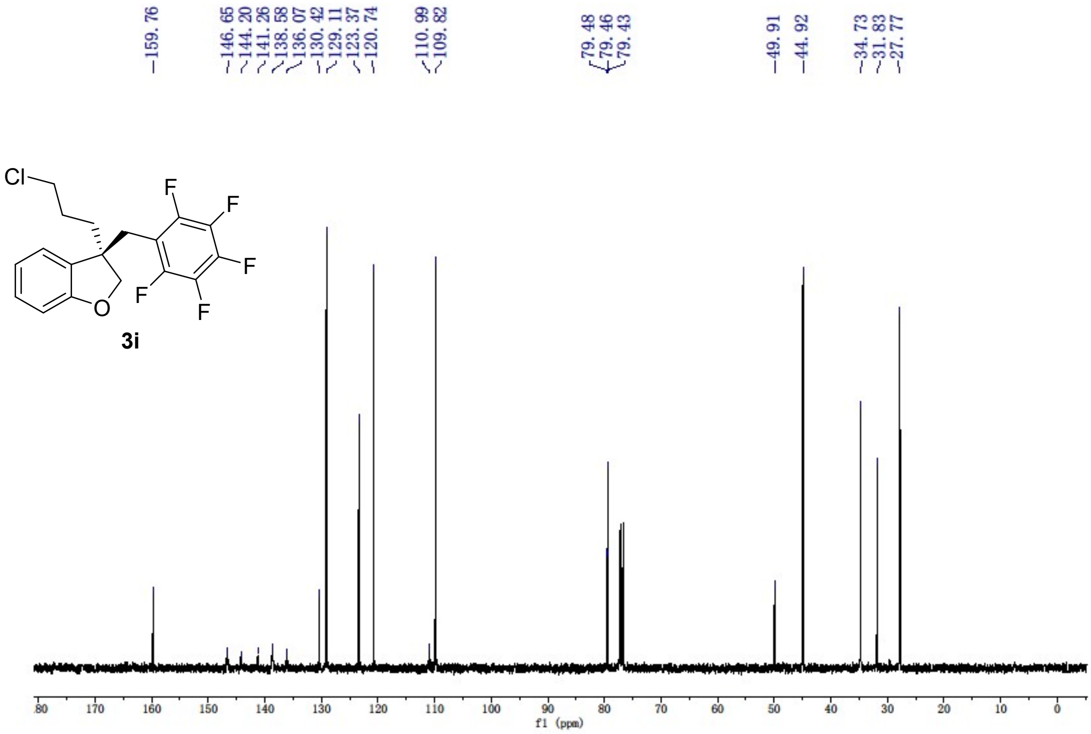
159.76
146.65
144.20
141.26
138.58
136.07
130.42
129.11
123.37
120.74
110.99
109.82
79.48
79.46
79.43
49.91
44.92
34.73
31.83
27.77
Cl
F
O
3i
f1 (ppm)

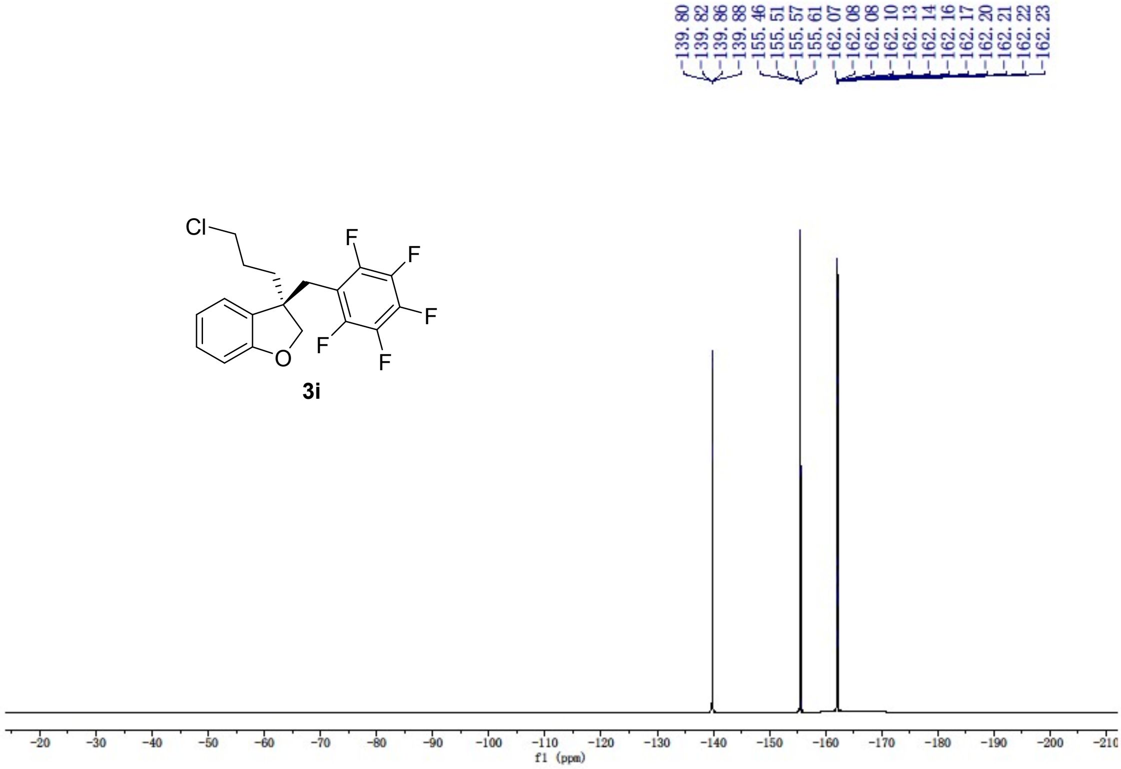
-139.80
-139.82
-139.86
-139.88
-155.46
-155.51
-155.57
-155.61
-162.07
-162.08
-162.08
-162.10
-162.13
-162.14
-162.16
-162.17
-162.20
-162.21
-162.22
-162.23
Cl
F
O
3i
f1 (ppm)

〈Chromatogram〉

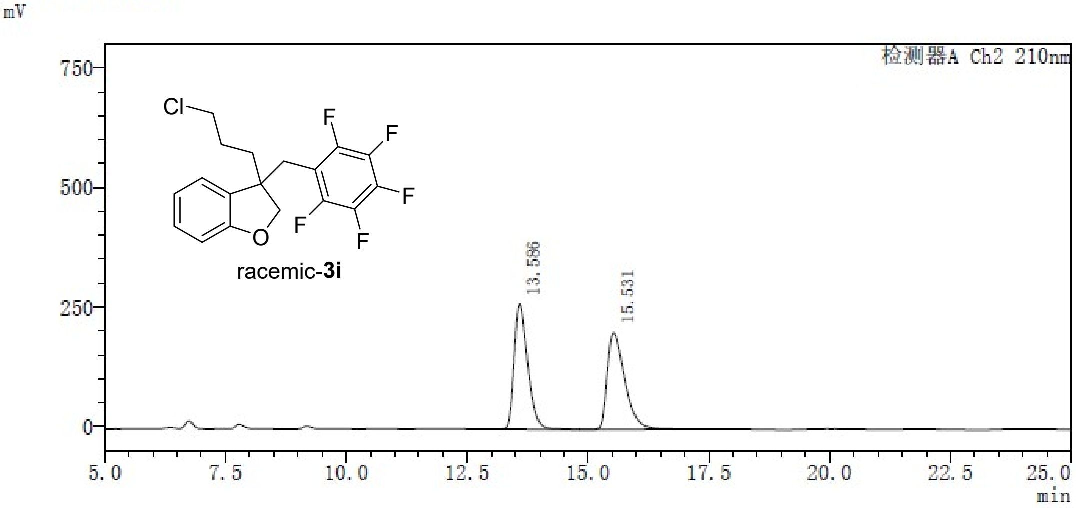

〈Peak Table〉

检测器A Ch2 210nm

No.	Ret. Time(min)	Height	Height%	Area	Area%
1	13.586	262008	56.404	5114316	49.978
2	15.531	202511	43.596	5118815	50.022
Total		464519	100.000	10233131	100.000

〈Chromatogram〉

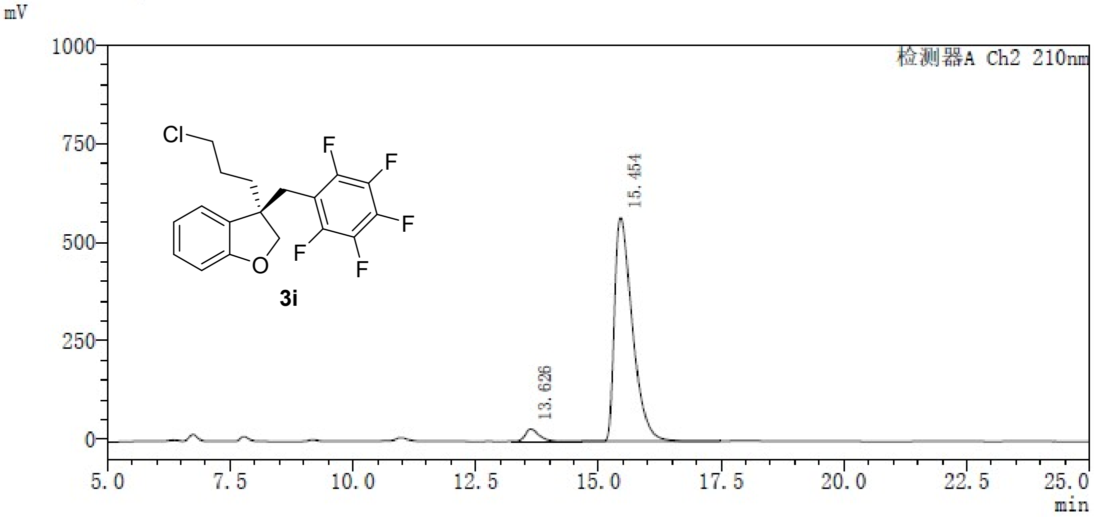

〈Peak Table〉

检测器A Ch2 210nm

No.	Ret. Time(min)	Height	Height%	Area	Area%
1	13.626	31179	5.209	611807	4.080
2	15.454	567439	94.791	14383470	95.920
Total		598618	100.000	14995277	100.000

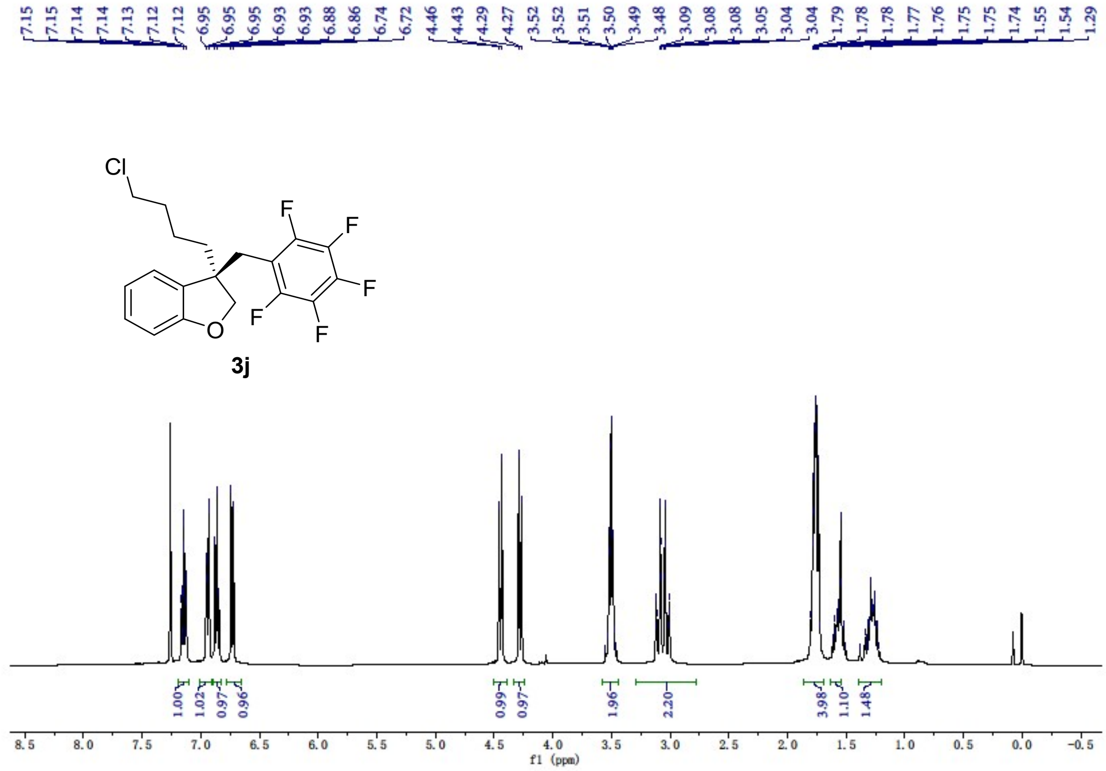
7.15 7.15 7.14 7.14 7.13 7.12 7.12 6.95 6.95 6.95 6.93 6.93 6.88 6.86 6.74 6.72 4.46 4.43 4.29 4.27 3.52 3.52 3.51 3.50 3.49 3.48 3.09 3.08 3.08 3.05 3.04 3.04 1.79 1.78 1.78 1.77 1.76 1.75 1.75 1.74 1.55 1.54 1.29
Cl
F
F
F
F
F
O
3j
1.00 1.02 0.97 0.96 0.99 0.97 1.96 2.20 3.98 1.10 1.48
f1 (ppm)

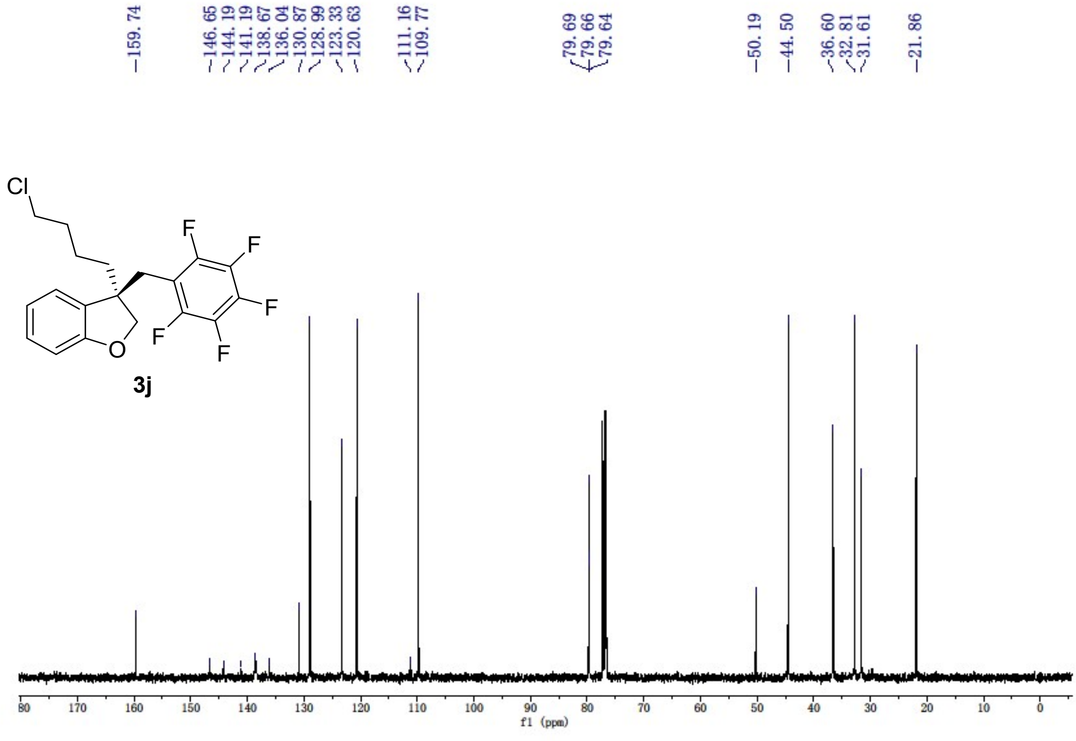
159.74 146.65 144.19 141.19 138.67 136.04 130.87 128.99 123.33 120.63 111.16 109.77 79.69 79.66 79.64 50.19 44.50 36.60 32.81 31.61 21.86
Cl
F
F
F
F
F
O
3j
f1 (ppm)

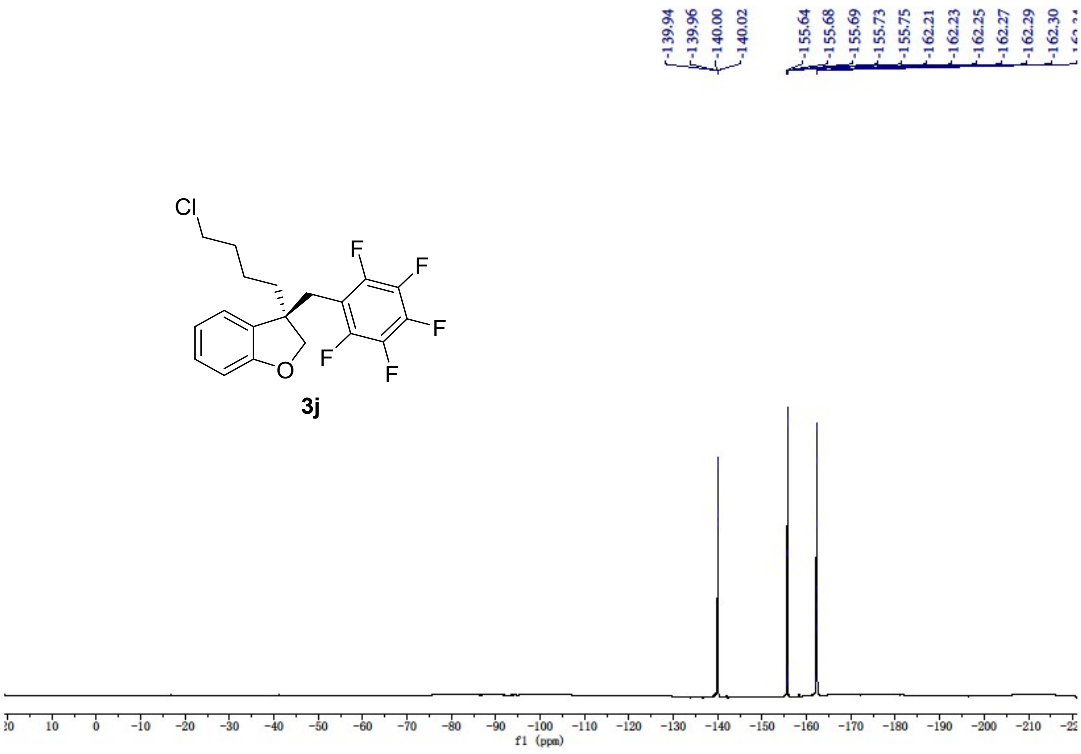

〈Chromatogram〉

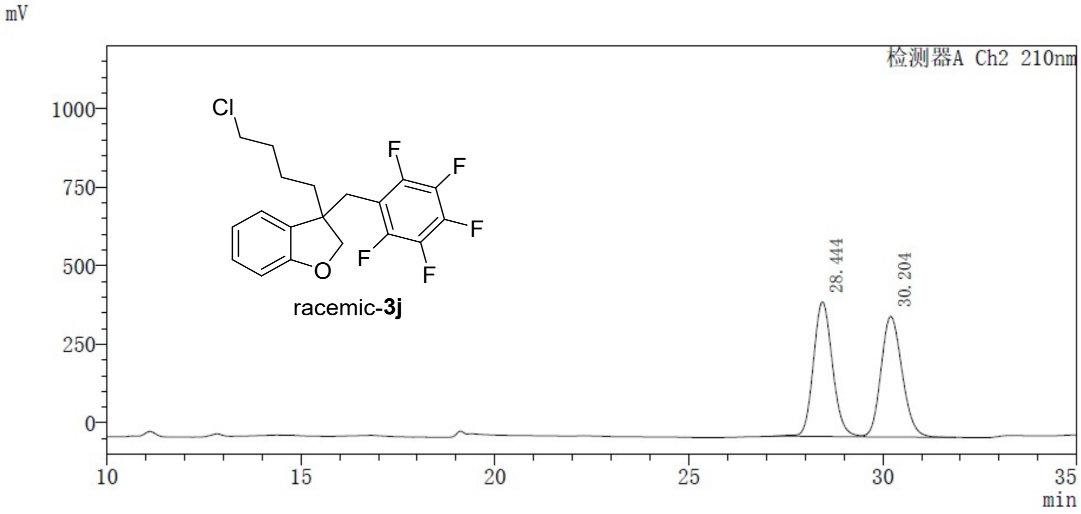

〈Peak Table〉

检测器A Ch2 210nm

No.	Ret.Time(min)	Height	Height%	Area	Area%
1	28.444	428489	52.757	14702874	50.245
2	30.204	383701	47.243	14559552	49.755
Total		812190	100.000	29262426	100.000

<Chromatogram>

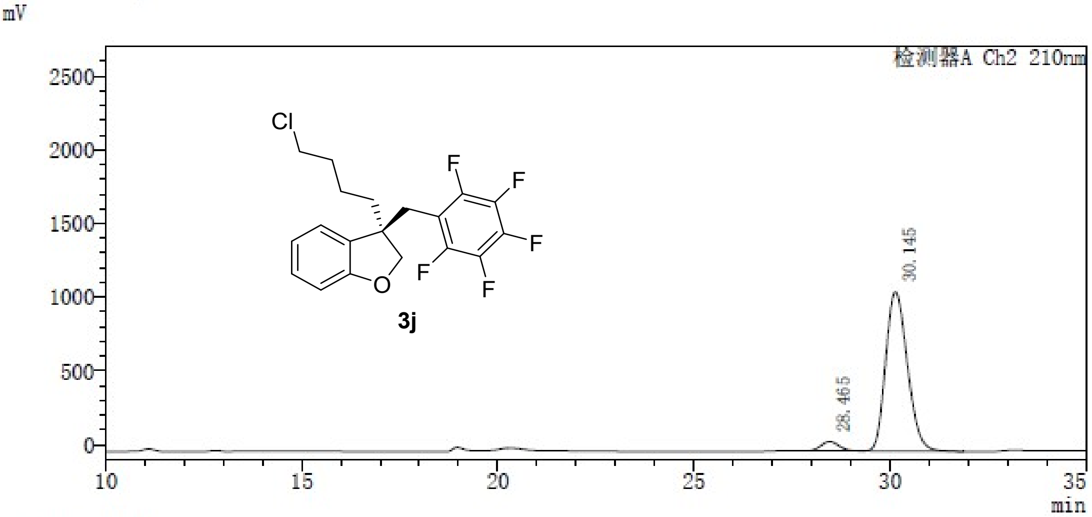

<Peak Table>

检测器A Ch2 210nm

No.	Ret. Time(min)	Height	Height%	Area	Area%
1	28.465	63231	5.533	2026910	4.636
2	30.145	1079487	94.467	41696622	95.364
Total		1142718	100.000	43723533	100.000

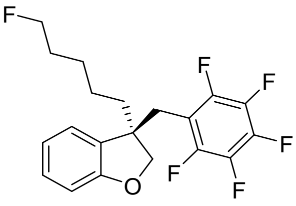

3k

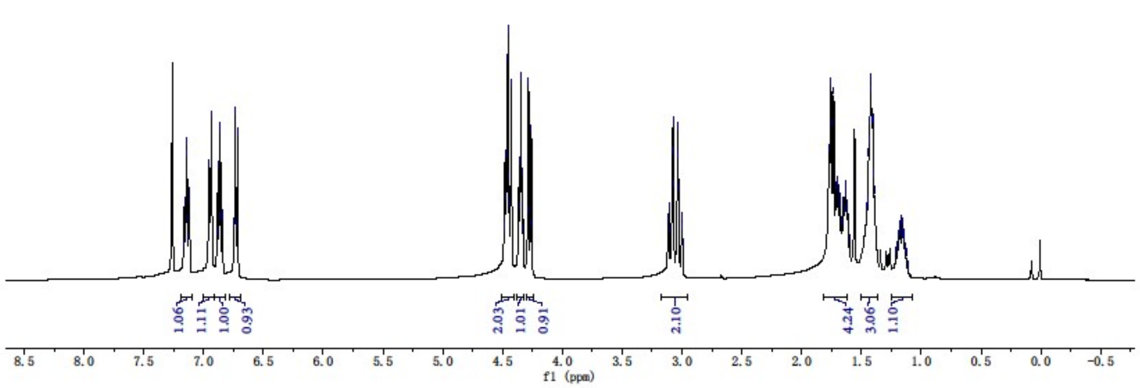

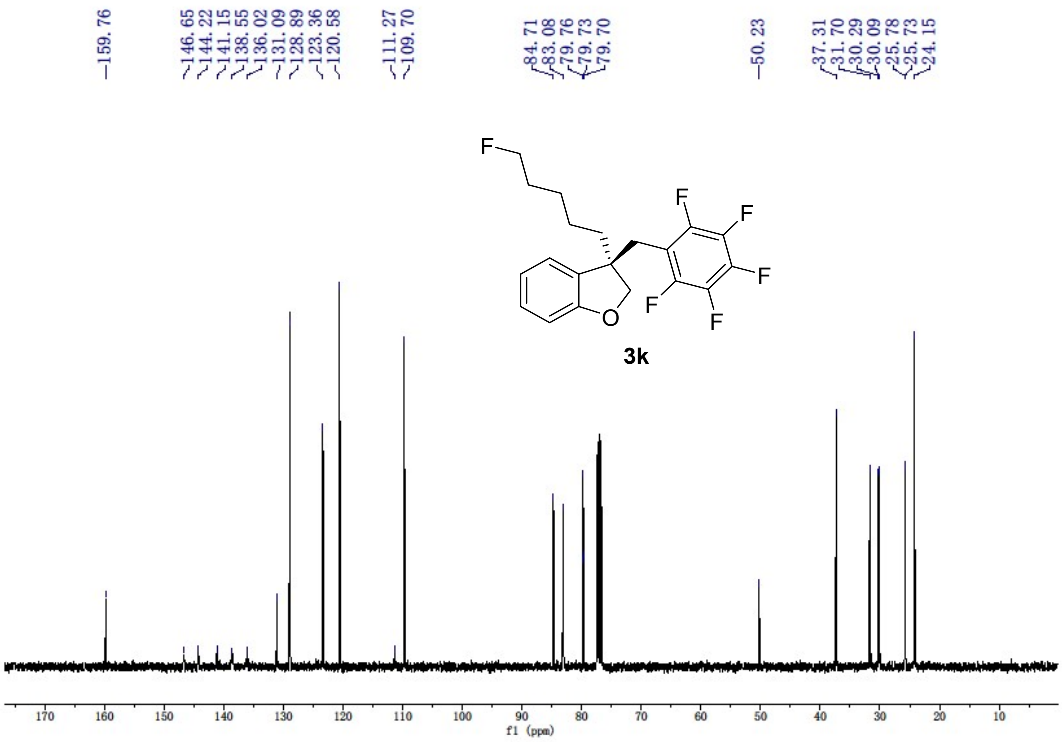
159.76
146.65
144.22
141.15
138.55
136.02
131.09
128.89
123.36
120.58
111.27
109.70
84.71
83.08
79.76
79.73
79.70
50.23
37.31
31.70
30.29
30.09
25.78
25.73
24.15
F
O
3k
f1 (ppm)

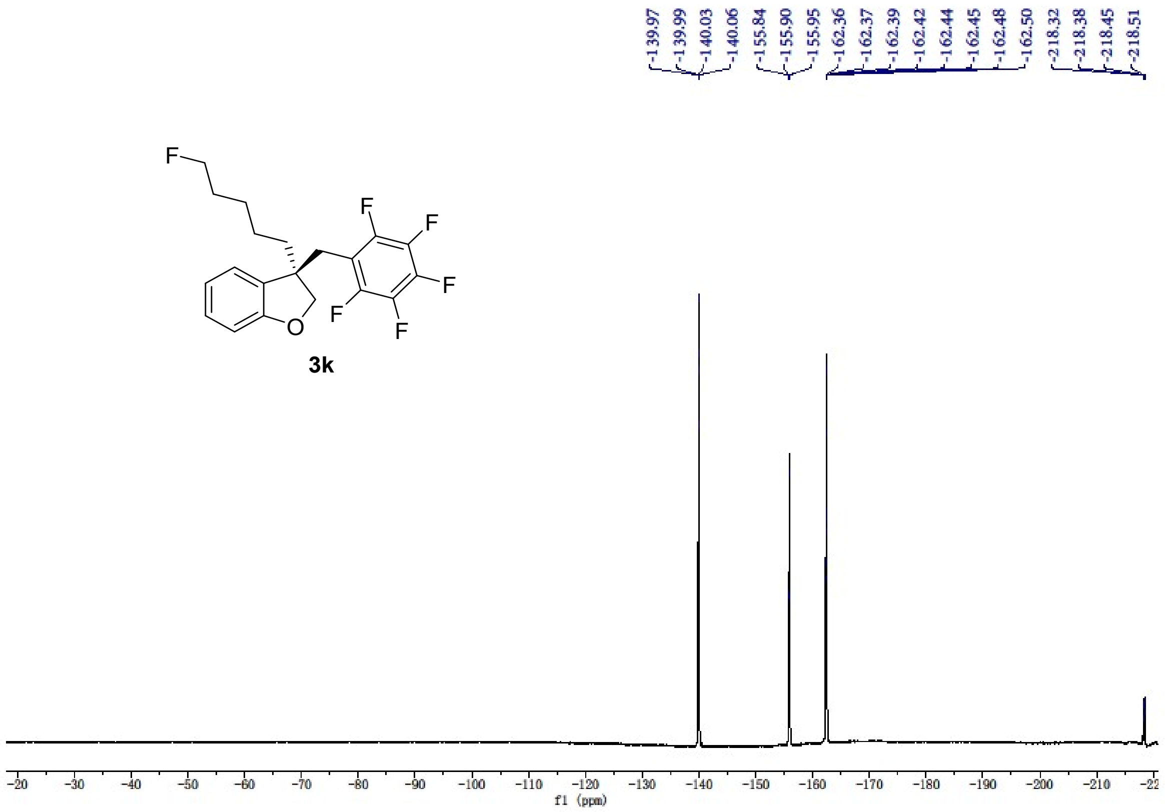
-139.97
-139.99
-140.03
-140.06
-155.84
-155.90
-155.95
-162.36
-162.37
-162.39
-162.42
-162.44
-162.45
-162.48
-162.50
-218.32
-218.38
-218.45
-218.51
F
O
3k
f1 (ppm)

<Chromatogram>

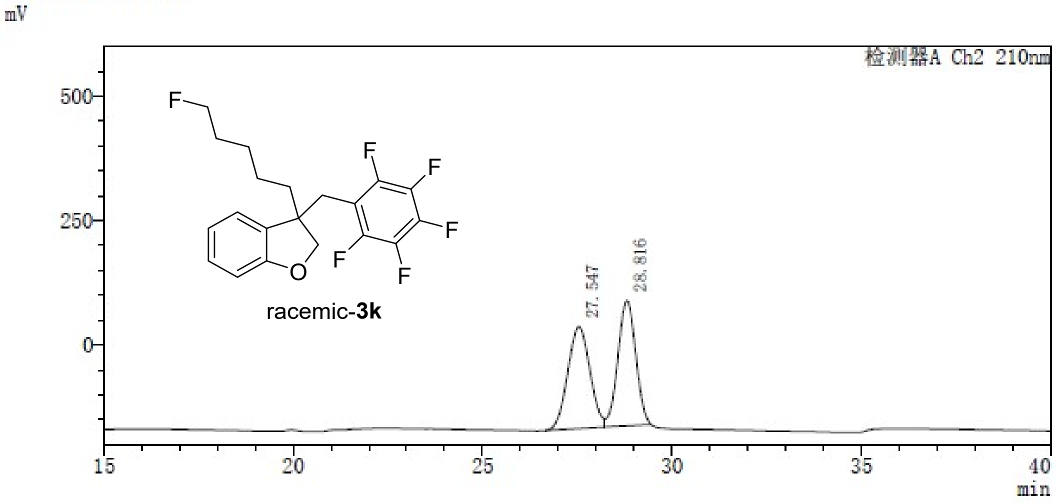

<Peak Table>

检测器A Ch2 210nm

No.	Ret. Time (min)	Height	Height%	Area	Area%
1	27.547	204236	44.809	8320998	50.193
2	28.816	251552	55.191	8256943	49.807
Total		455788	100.000	16577941	100.000

<Chromatogram>

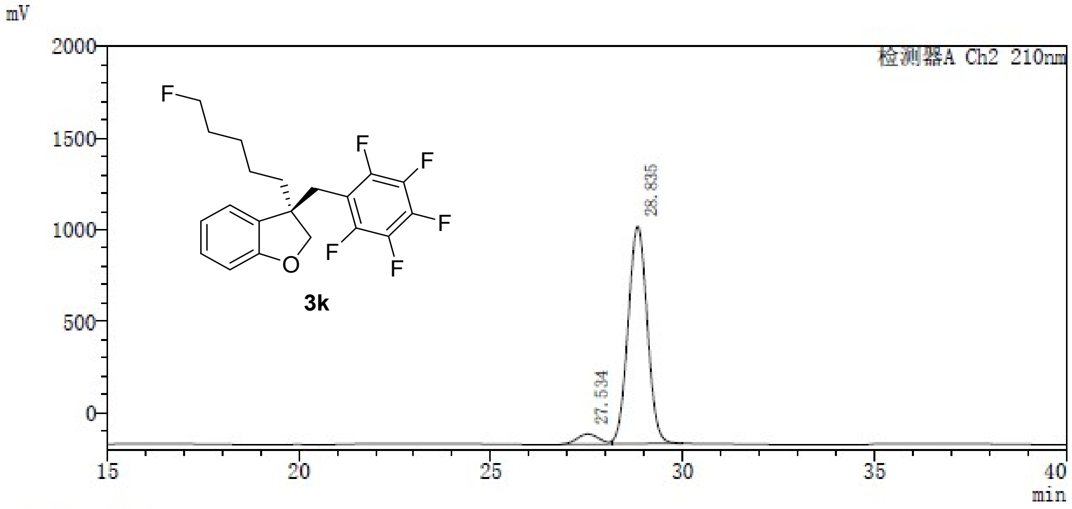

<Peak Table>

检测器A Ch2 210nm

No.	Ret. Time (min)	Height	Height%	Area	Area%
1	27.534	56083	4.517	2257787	5.197
2	28.835	1185611	95.483	41182564	94.803
Total		1241694	100.000	43440351	100.000

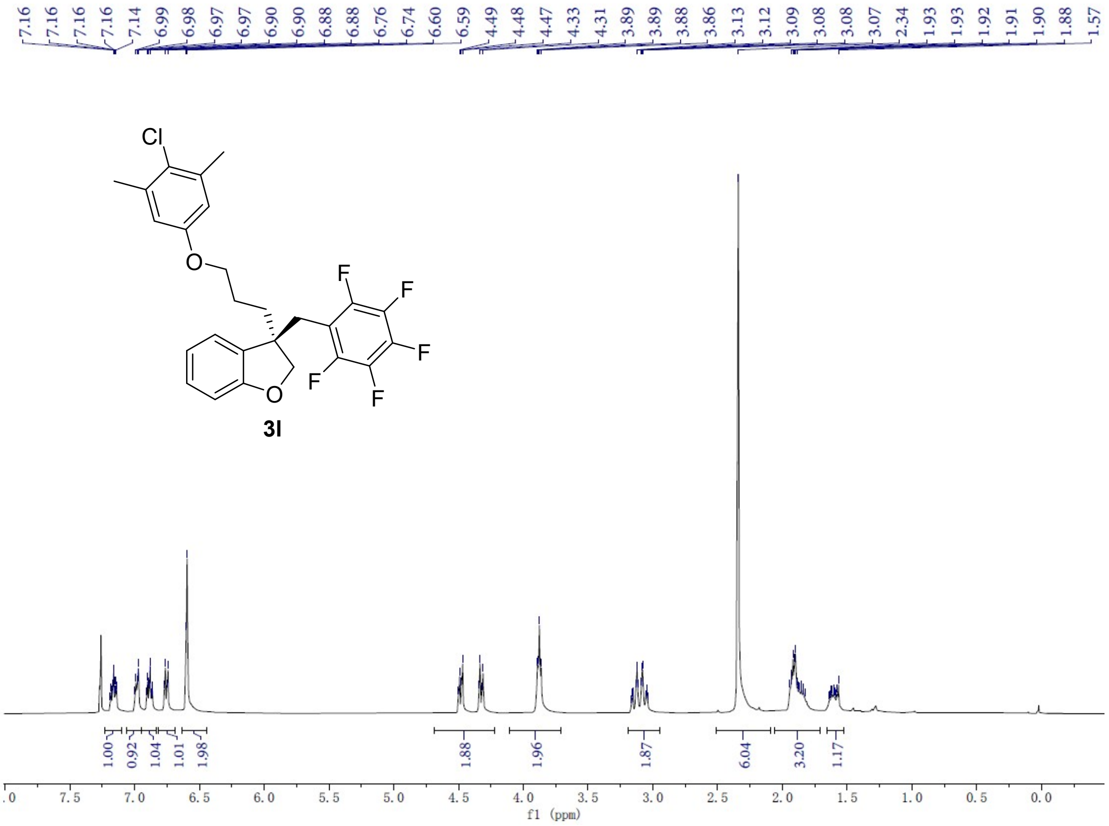

7.16 7.16 7.16 7.16 7.14 6.99 6.98 6.97 6.97 6.90 6.90 6.88 6.88 6.76 6.74 6.60 6.59 4.49 4.48 4.47 4.33 4.31 3.89 3.89 3.88 3.86 3.13 3.12 3.09 3.08 3.08 3.07 2.34 1.93 1.93 1.92 1.91 1.90 1.88 1.57
Cl
O
F
3l
1.00 0.92 1.04 1.01 1.98 1.88 1.96 1.87 6.04 3.20 1.17
f1 (ppm)

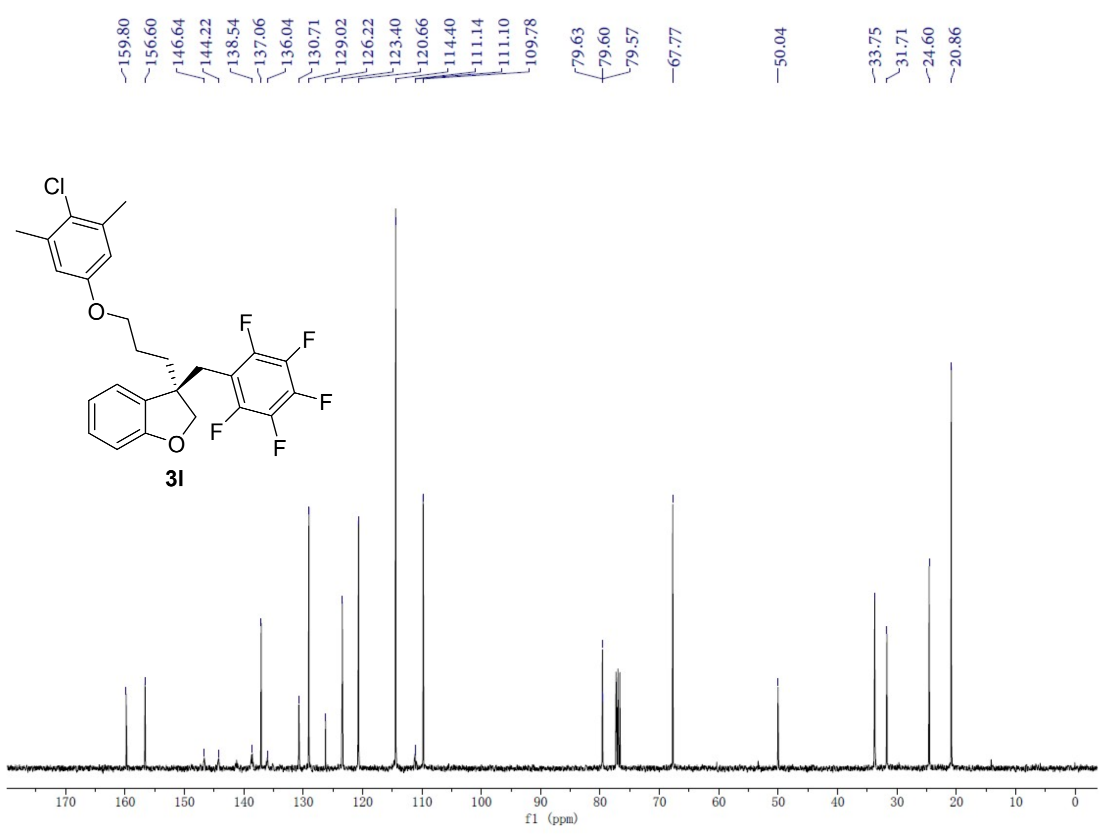

159.80 156.60 146.64 144.22 138.54 137.06 136.04 130.71 129.02 126.22 123.40 120.66 114.40 111.14 111.10 109.78 79.63 79.60 79.57 67.77 50.04 33.75 31.71 24.60 20.86
Cl
O
F
3l
f1 (ppm)

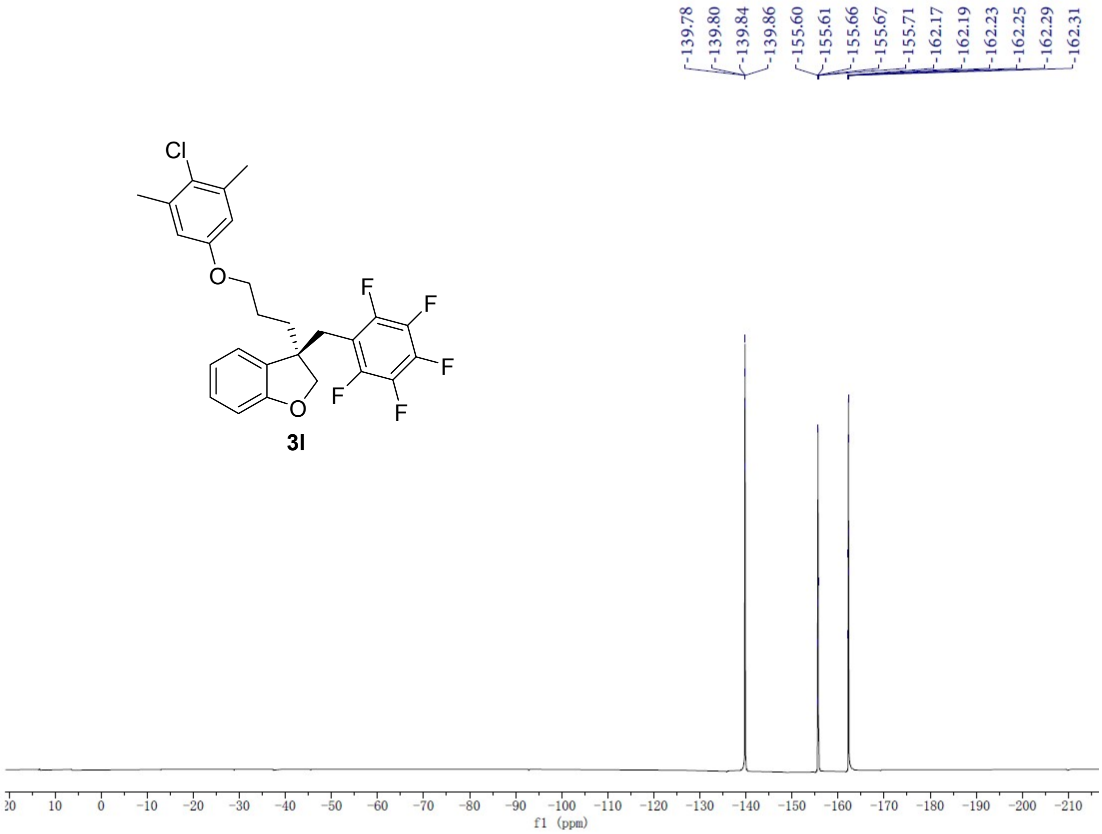

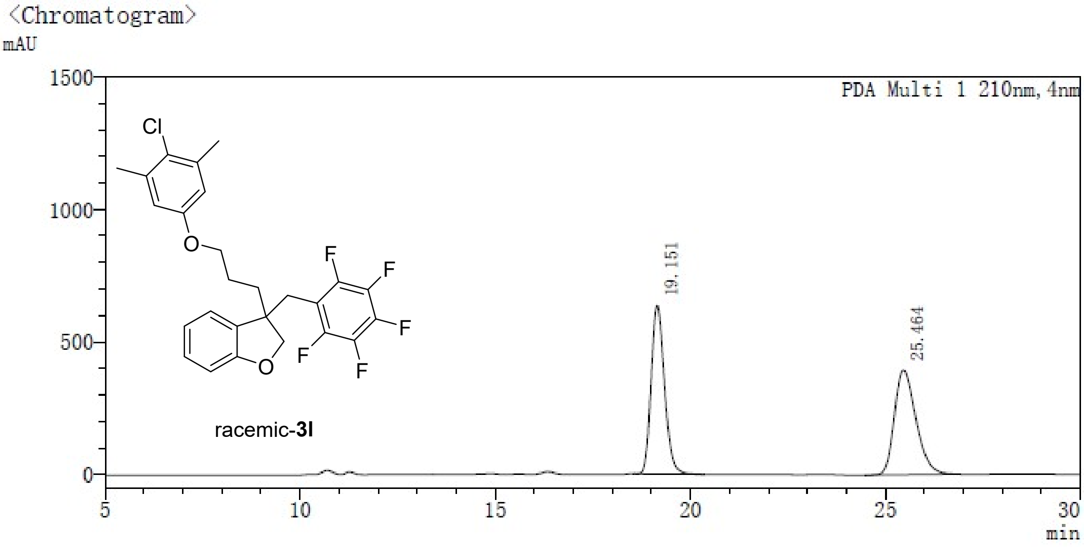

<Peak Table>

PDA Ch1 210nm

No.	Ret. Time(min)	Height	Height%	Area	Area%
1	19.151	634879	61.686	15131763	49.937
2	25.464	394330	38.314	15169993	50.063
Total		1029209	100.000	30301756	100.000

<Chromatogram>

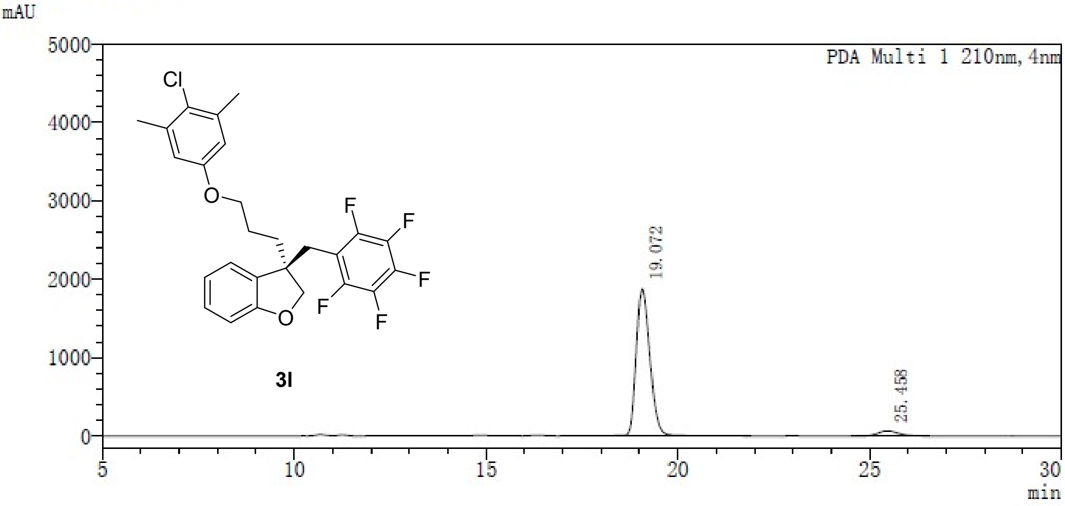

<Peak Table>

PDA Ch1 210nm

No.	Ret. Time (min)	Height	Height%	Area	Area%
1	19.072	1877447	96.590	47002215	94.981
2	25.458	66289	3.410	2483930	5.019
Total		1943736	100.000	49486144	100.000

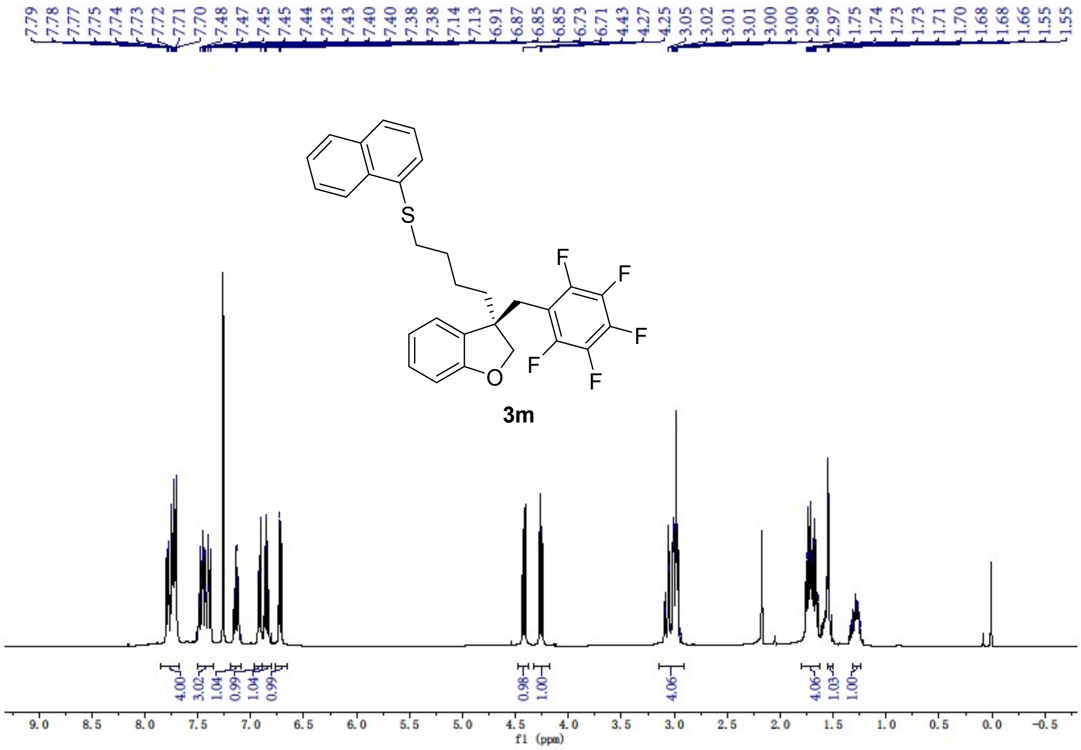

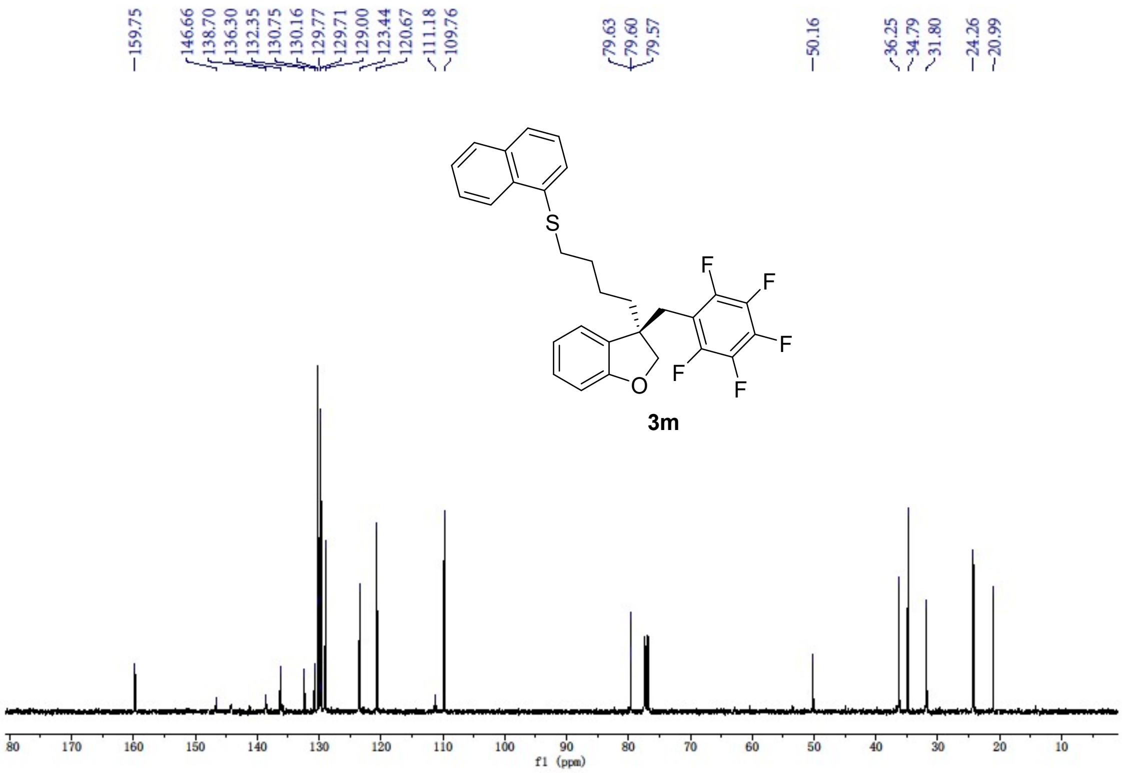
159.75
146.66
138.70
136.30
132.35
130.75
130.16
129.77
129.71
129.00
123.44
120.67
111.18
109.76
79.63
79.60
79.57
50.16
36.25
34.79
31.80
24.26
20.99
S
F
F
F
F
F
O
f1 (ppm)

3m

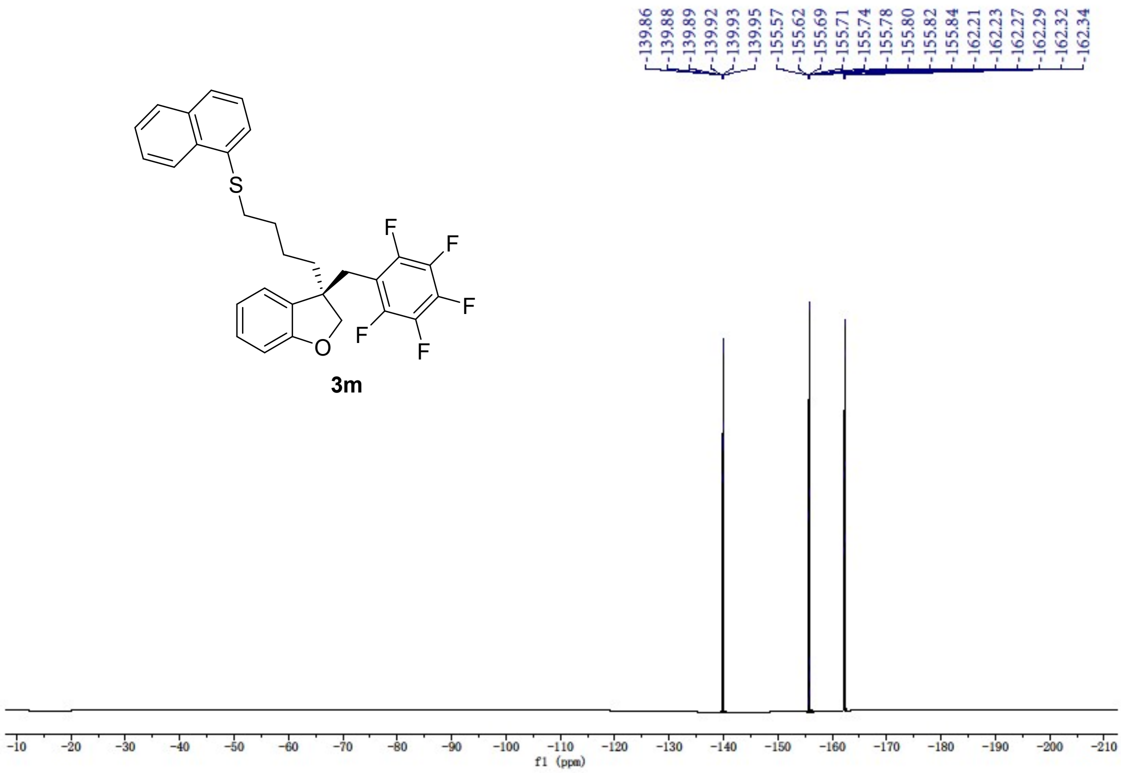
-139.86
-139.88
-139.89
-139.92
-139.93
-139.95
-155.57
-155.62
-155.69
-155.71
-155.74
-155.78
-155.80
-155.82
-155.84
-162.21
-162.23
-162.27
-162.29
-162.32
-162.34
S
F
F
F
F
F
O
f1 (ppm)

3m

〈Chromatogram〉

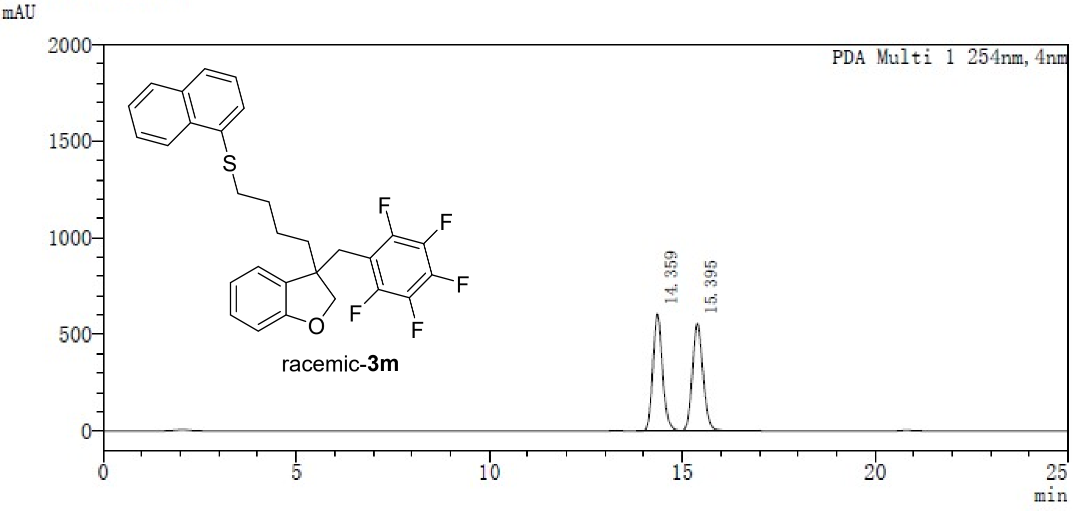

〈Peak Table〉

PDA Ch1 254nm

No.	Ret.Time(min)	Height	Height%	Area	Area%
1	14.359	603764	52.064	10600889	49.700
2	15.395	555894	47.936	10728756	50.300
Total		1159657	100.000	21329645	100.000

〈Chromatogram〉

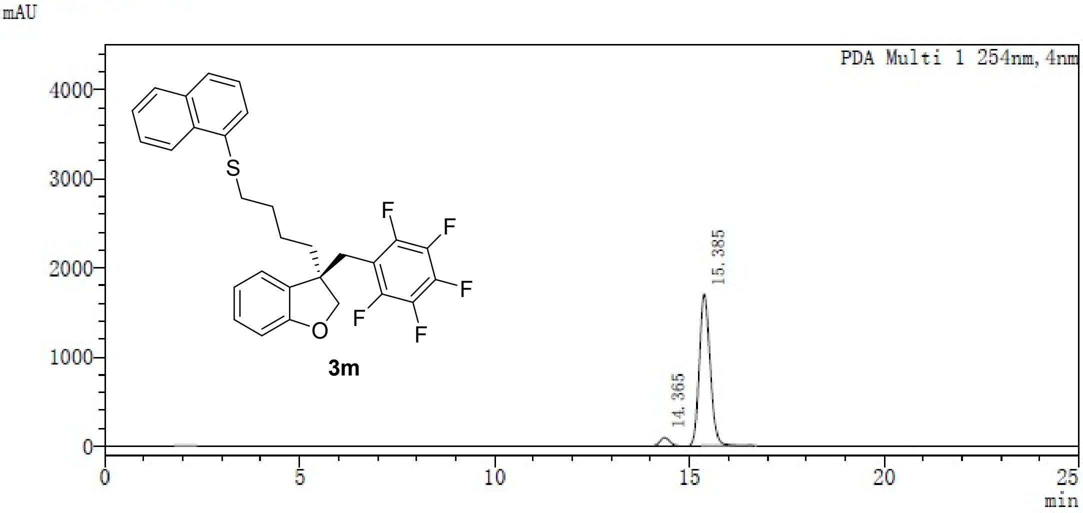

〈Peak Table〉

PDA Ch1 254nm

No.	Ret.Time(min)	Height	Height%	Area	Area%
1	14.365	94357	5.257	1598394	4.631
2	15.385	1700593	94.743	32915480	95.369
Total		1794950	100.000	34513874	100.000

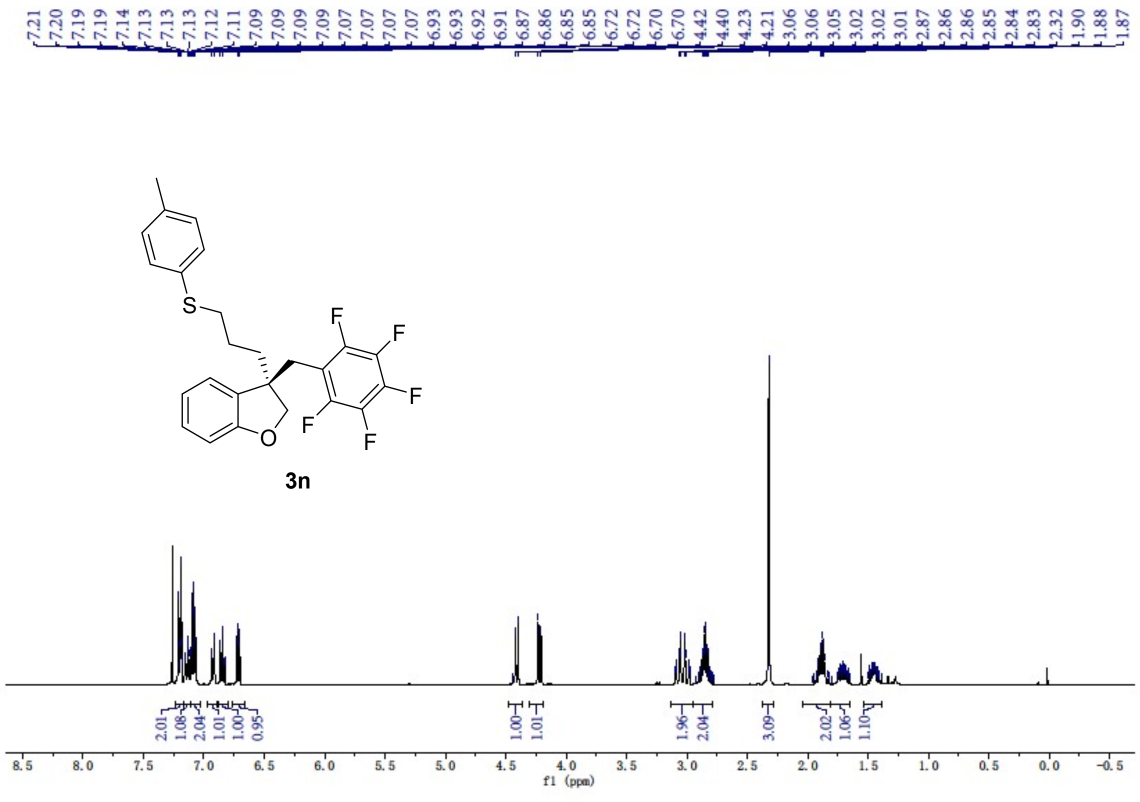
3n
f1 (ppm)

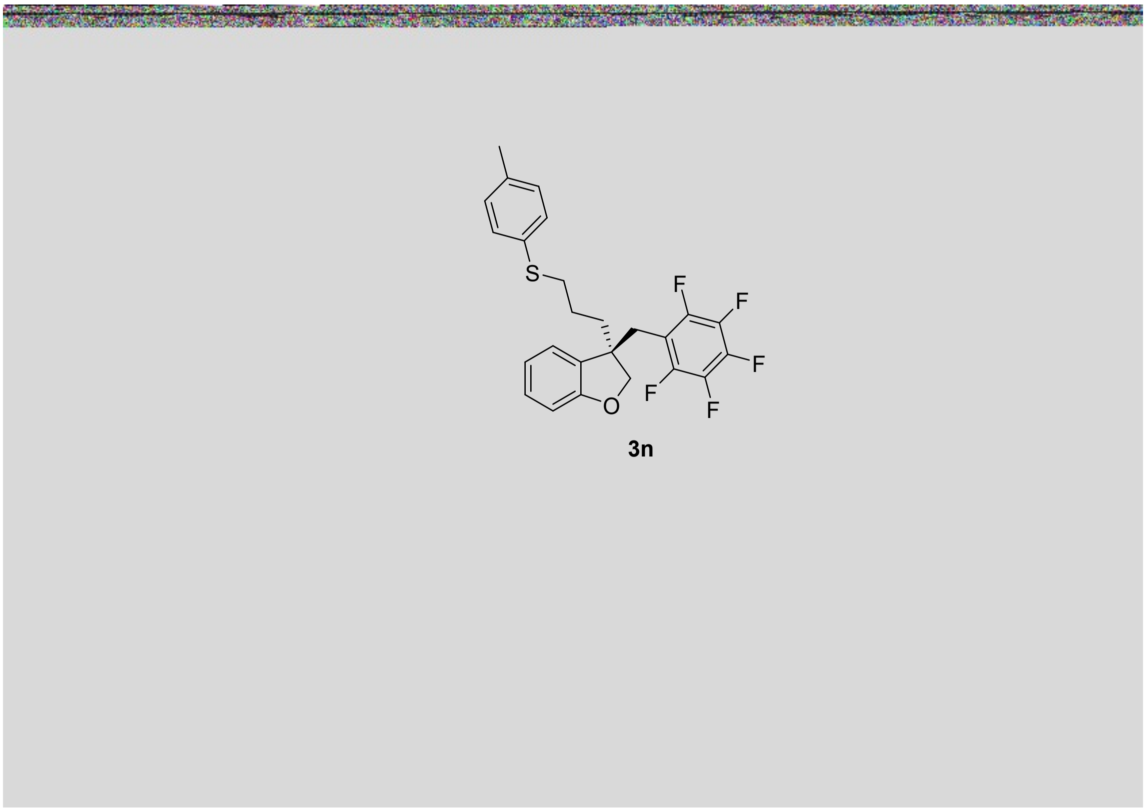
3n

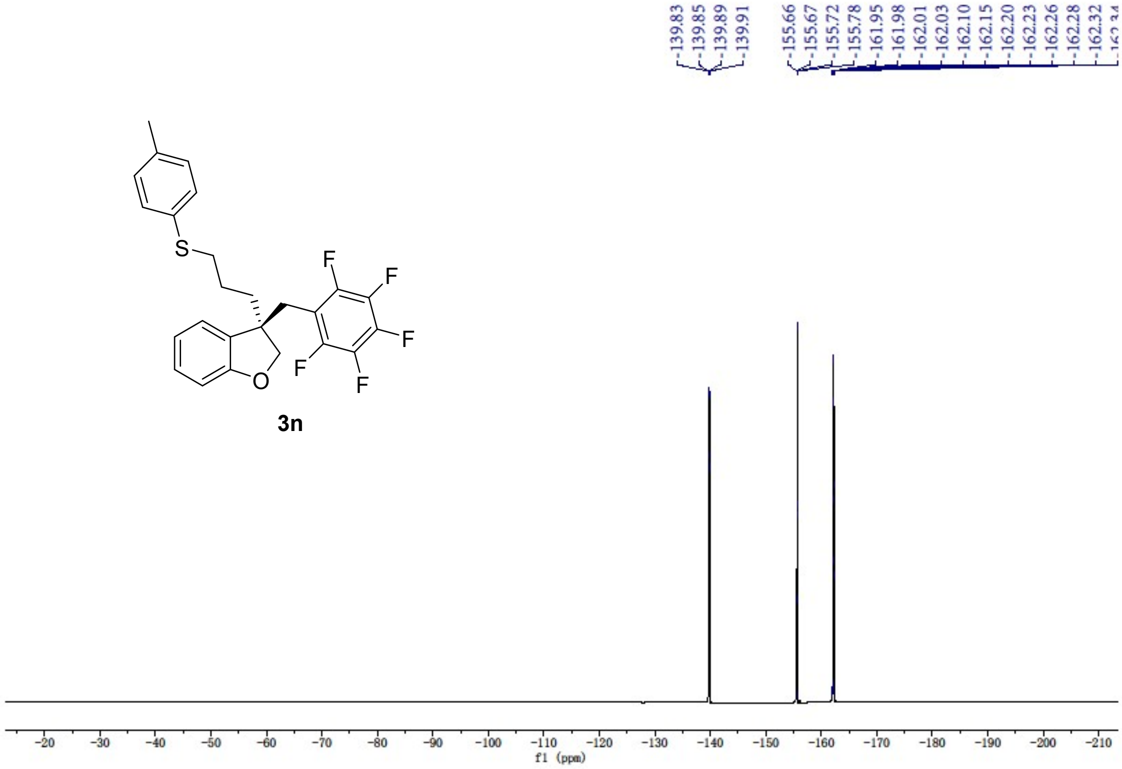

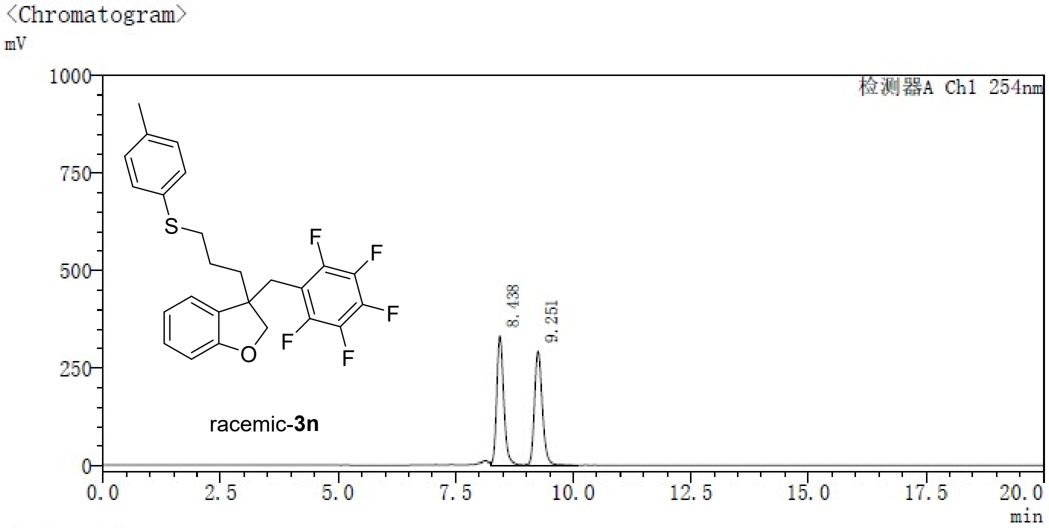

<Peak Table>

检测器A Ch1 254nm

No.	Ret. Time(min)	Height	Height%	Area	Area%
1	8.438	330374	53.144	3518989	50.430
2	9.251	291282	46.856	3458943	49.570
Total		621656	100.000	6977932	100.000

<Chromatogram>

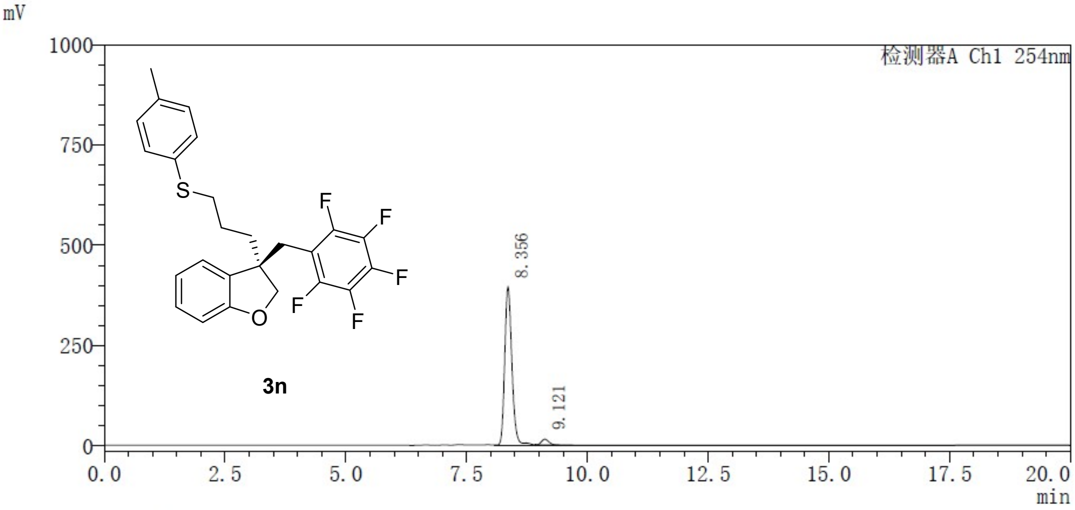

<Peak Table>

检测器A Ch1 254nm

No.	Ret. Time (min)	Height	Height%	Area	Area%
1	8.356	395550	96.540	4264119	96.557
2	9.121	14176	3.460	152050	3.443
Total		409726	100.000	4416169	100.000

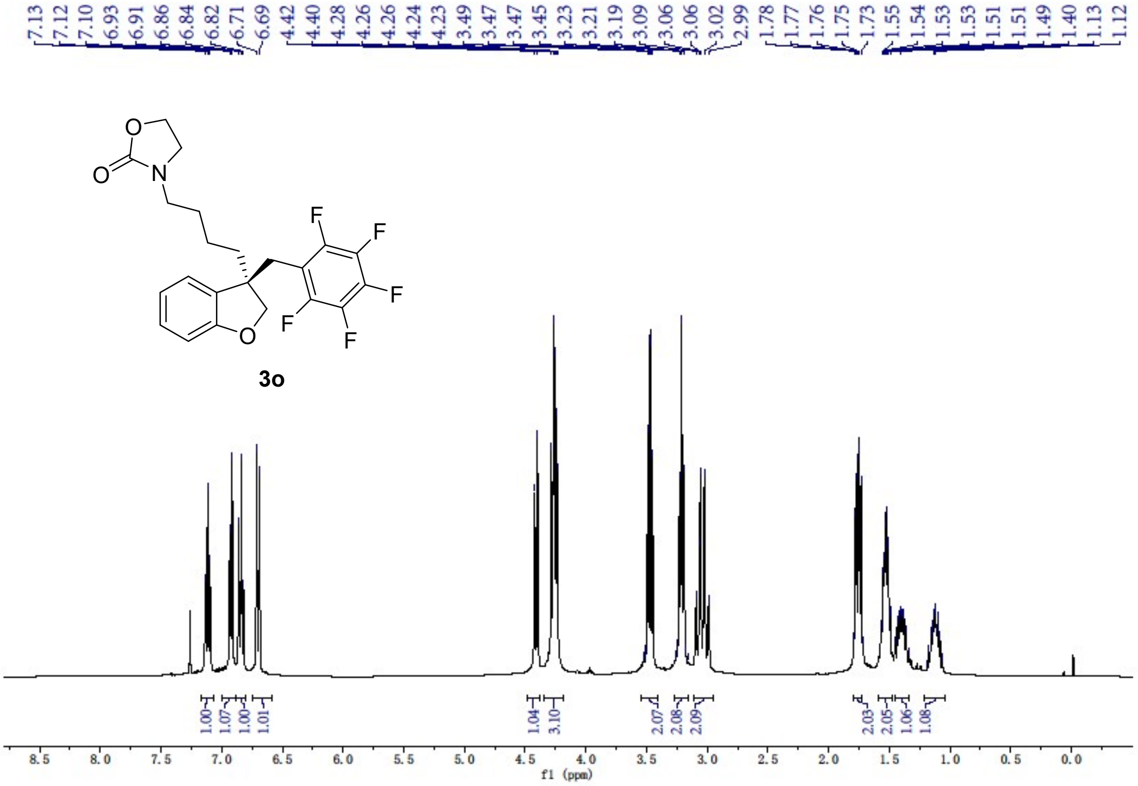

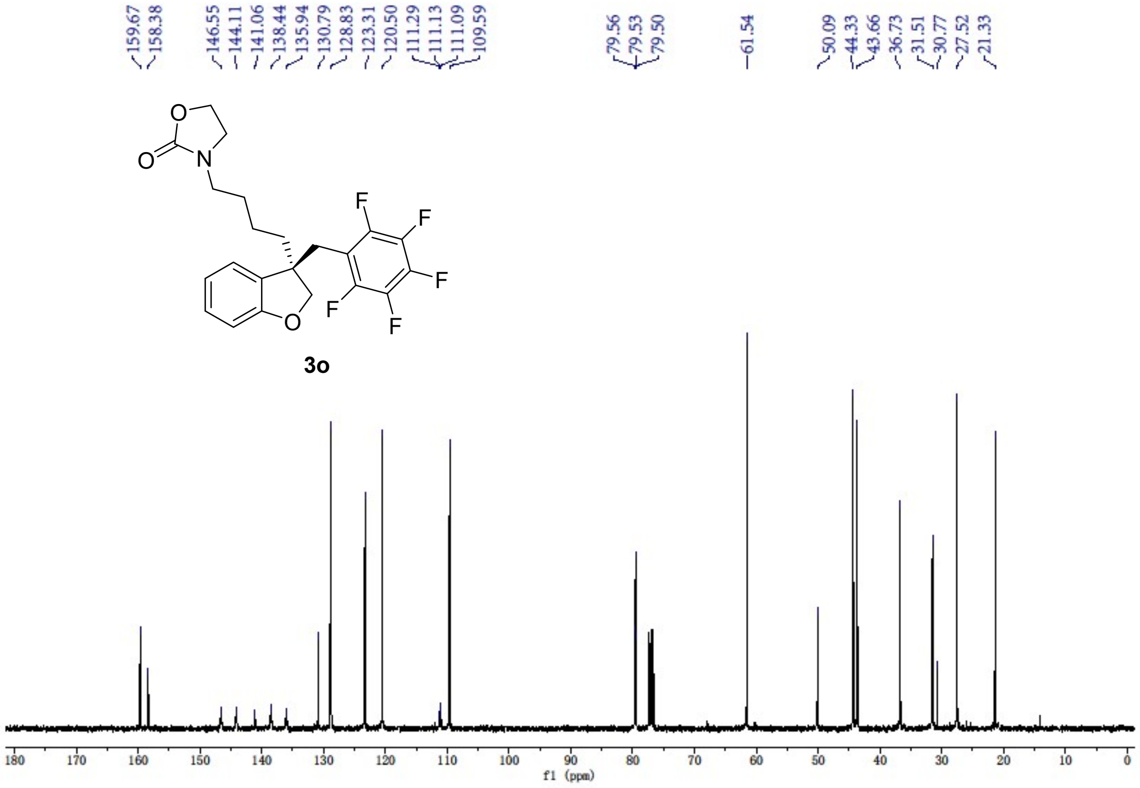
159.67
158.38
146.55
144.11
141.06
138.44
135.94
130.79
128.83
123.31
120.50
111.29
111.13
111.09
109.59
79.56
79.53
79.50
61.54
50.09
44.33
43.66
36.73
31.51
30.77
27.52
21.33
3o
f1 (ppm)

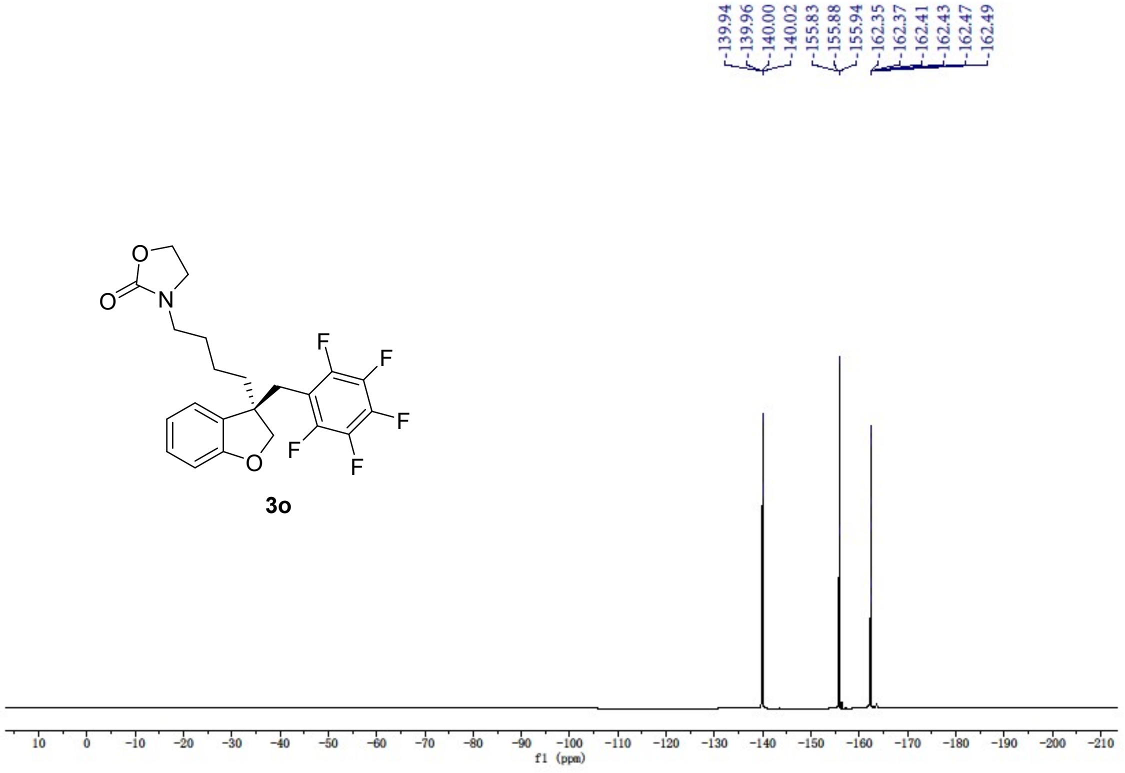
-139.94
-139.96
-140.00
-140.02
-155.83
-155.88
-155.94
-162.35
-162.37
-162.41
-162.43
-162.47
-162.49
3o
f1 (ppm)

<Chromatogram>

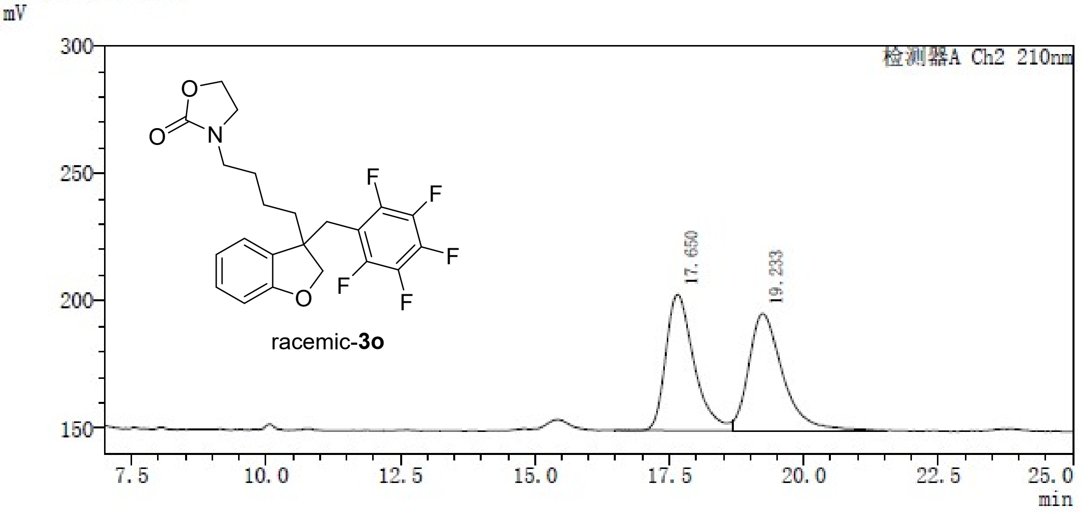

<Peak Table>

检测器A Ch2 210nm

No.	Ret. Time(min)	Height	Height%	Area	Area%
1	17.650	53354	53.760	1951328	49.232
2	19.233	45890	46.240	2012238	50.768
Total		99243	100.000	3963565	100.000

<Chromatogram>

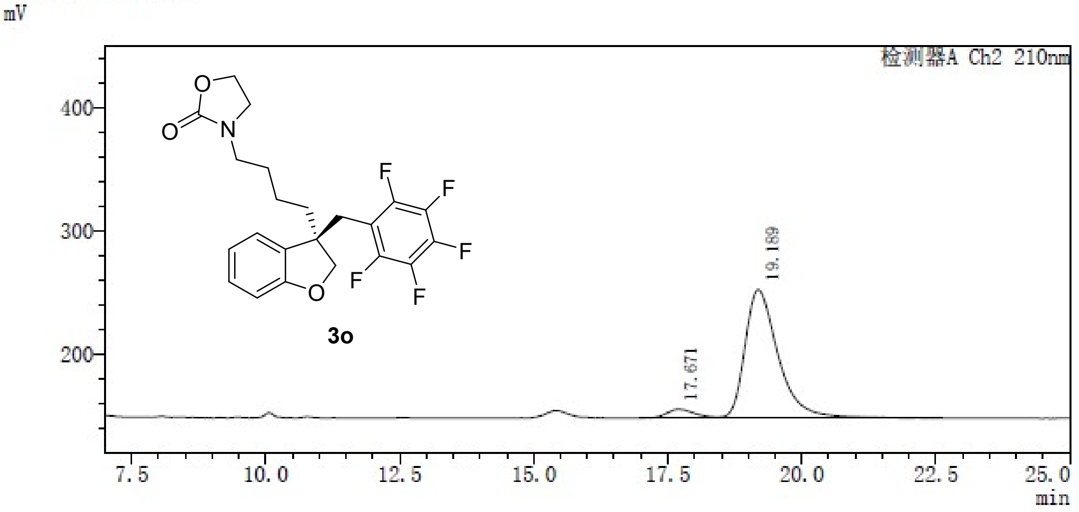

<Peak Table>

检测器A Ch2 210nm

No.	Ret. Time(min)	Height	Height%	Area	Area%
1	17.671	7096	6.371	255826	5.398
2	19.189	104293	93.629	4483590	94.602
Total		111390	100.000	4739416	100.000

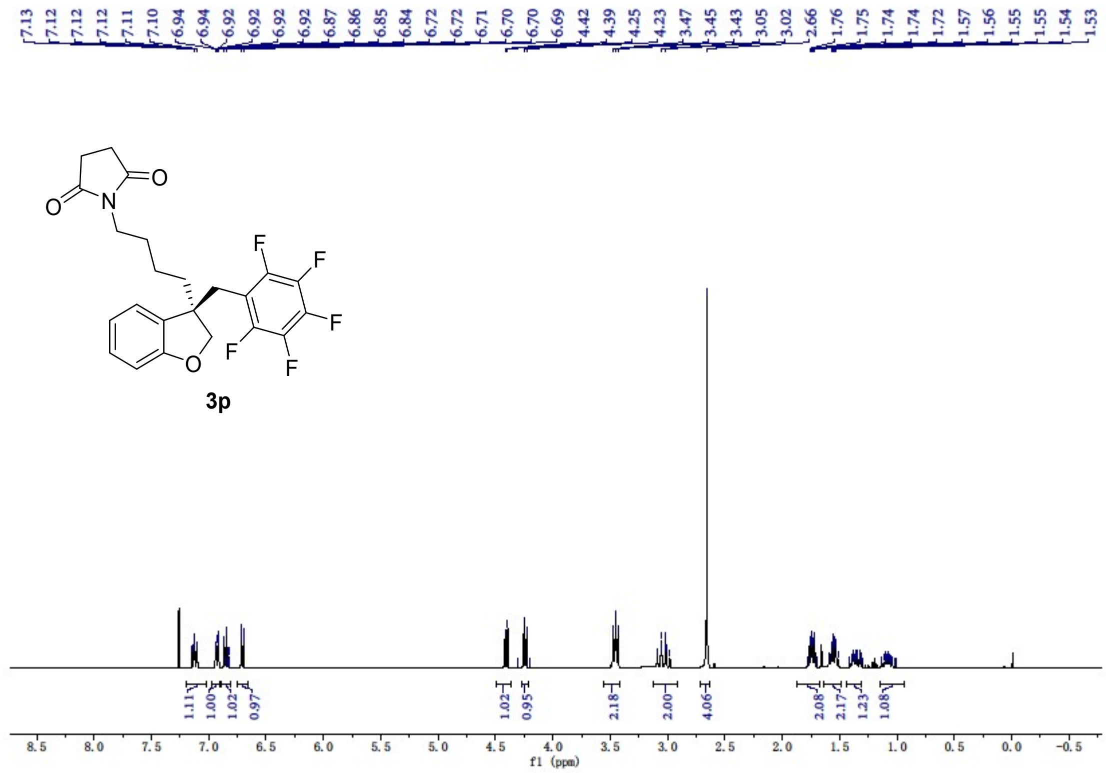
7.13 7.12 7.12 7.12 7.11 7.10 6.94 6.94 6.92 6.92 6.92 6.92 6.87 6.86 6.85 6.84 6.72 6.72 6.71 6.70 6.70 6.69 4.42 4.39 4.25 4.23 3.47 3.45 3.43 3.05 3.02 2.66 1.76 1.75 1.74 1.74 1.72 1.57 1.56 1.55 1.55 1.54 1.53
3p
1.11 1.00 1.02 0.97 1.02 0.95 2.18 2.00 4.06 2.08 2.17 1.23 1.08
f1 (ppm)

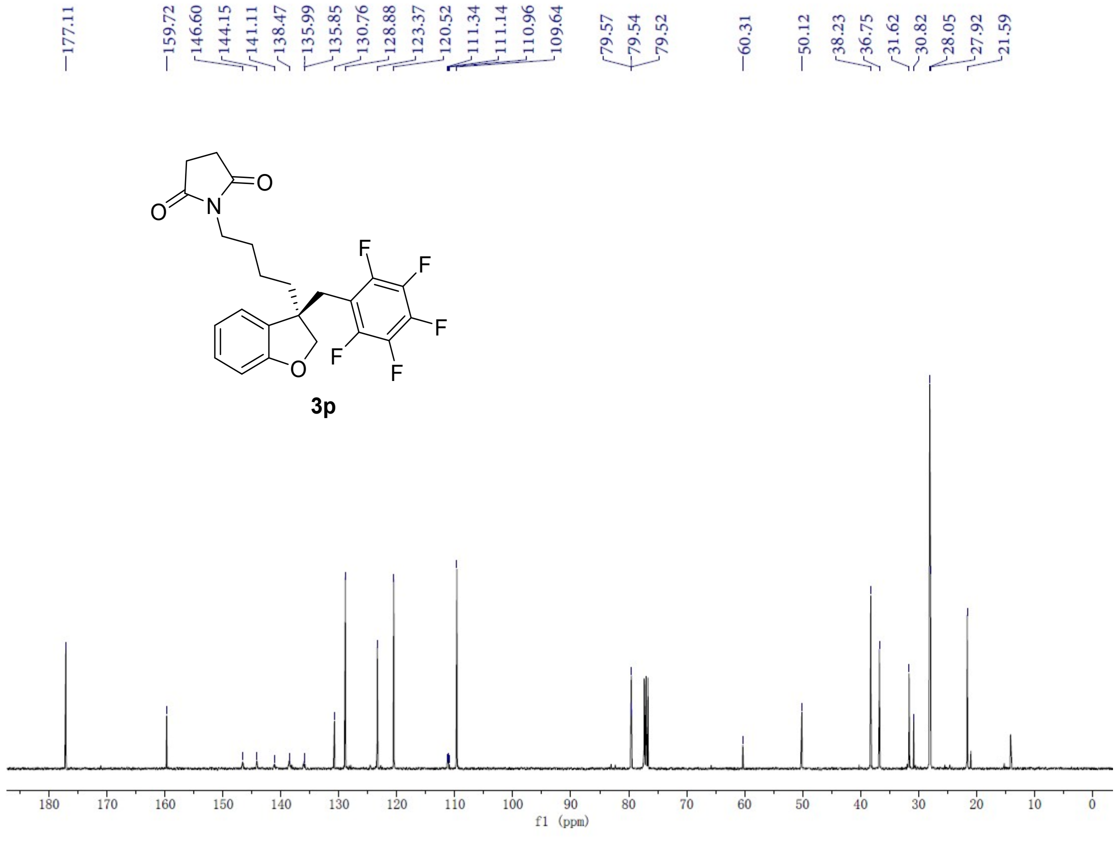
177.11 159.72 146.60 144.15 141.11 138.47 135.99 135.85 130.76 128.88 123.37 120.52 111.34 111.14 110.96 109.64 79.57 79.54 79.52 60.31 50.12 38.23 36.75 31.62 30.82 28.05 27.92 21.59
3p
f1 (ppm)

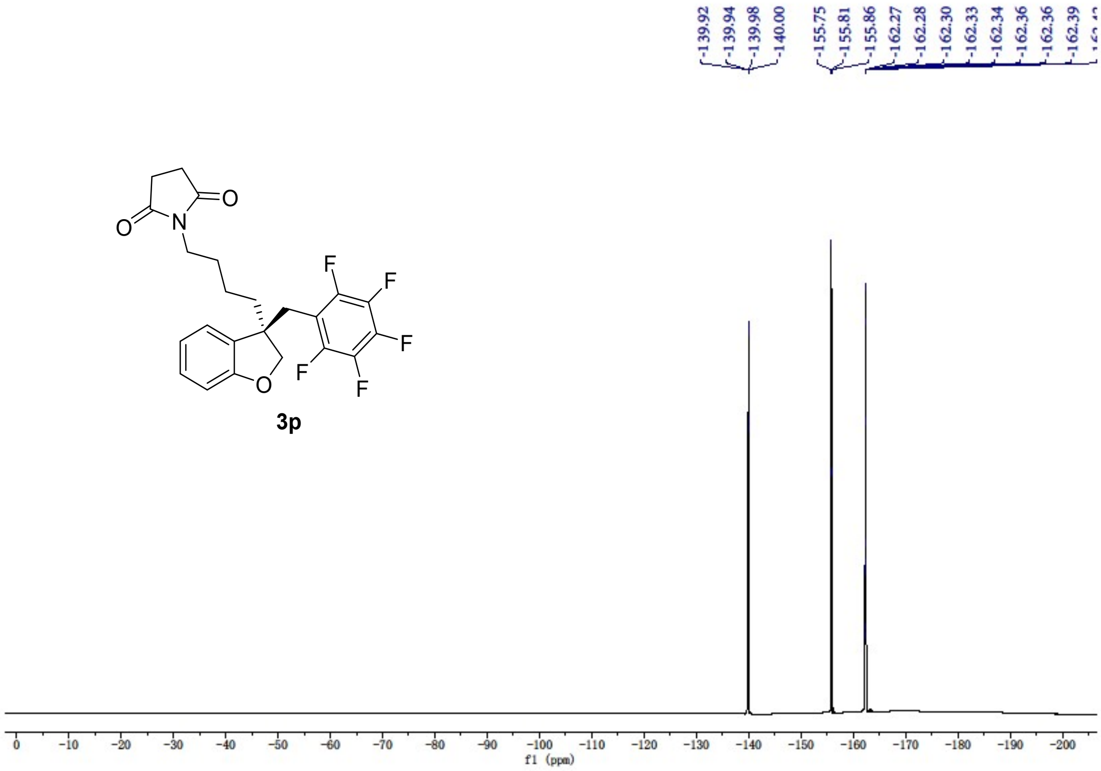

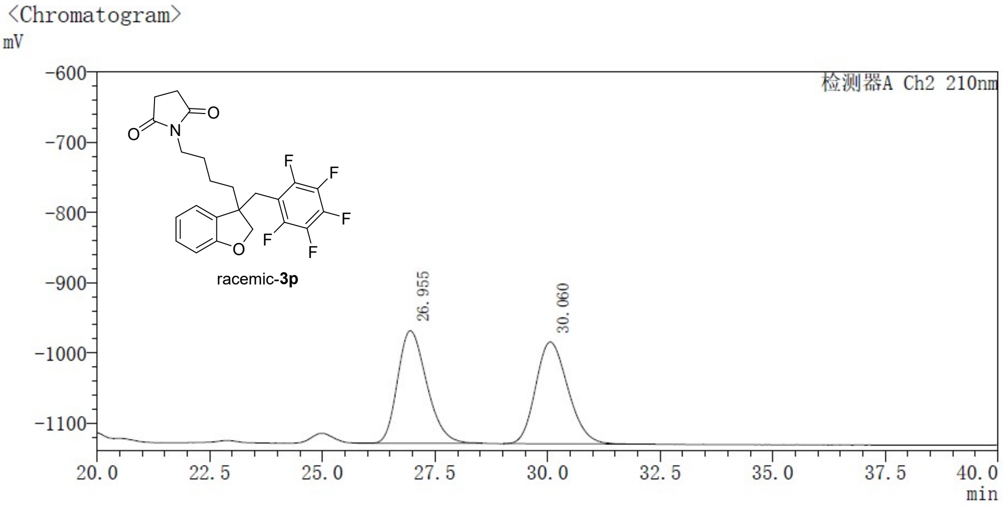

<Peak Table>

检测器A Ch2 210nm

No.	Ret. Time (min)	Height	Height%	Area	Area%
1	26.955	160300	52.468	7212174	49.889
2	30.060	145218	47.532	7244241	50.111
Total		305518	100.000	14456414	100.000

<Chromatogram>

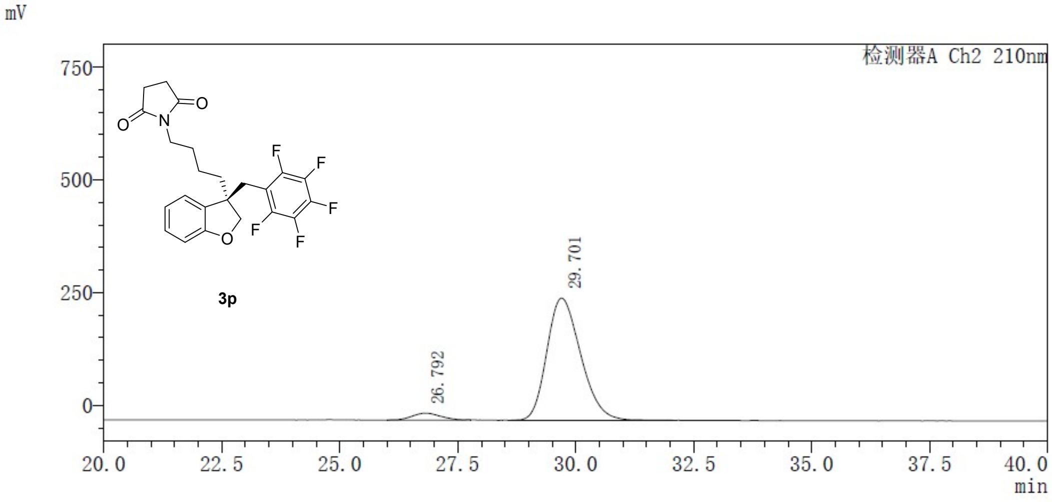

<Peak Table>

检测器A Ch2 210nm

No.	Ret. Time (min)	Height	Height%	Area	Area%
1	26.792	15003	5.246	639111	4.514
2	29.701	270962	94.754	13520168	95.486
Total		285964	100.000	14159280	100.000

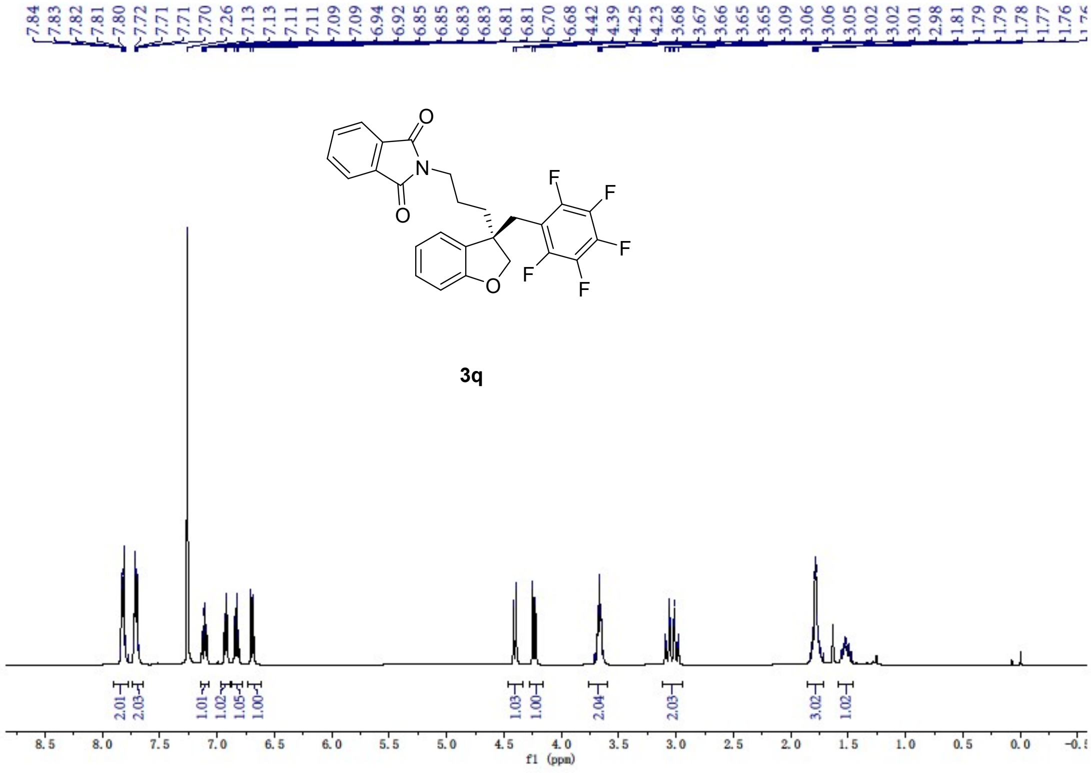

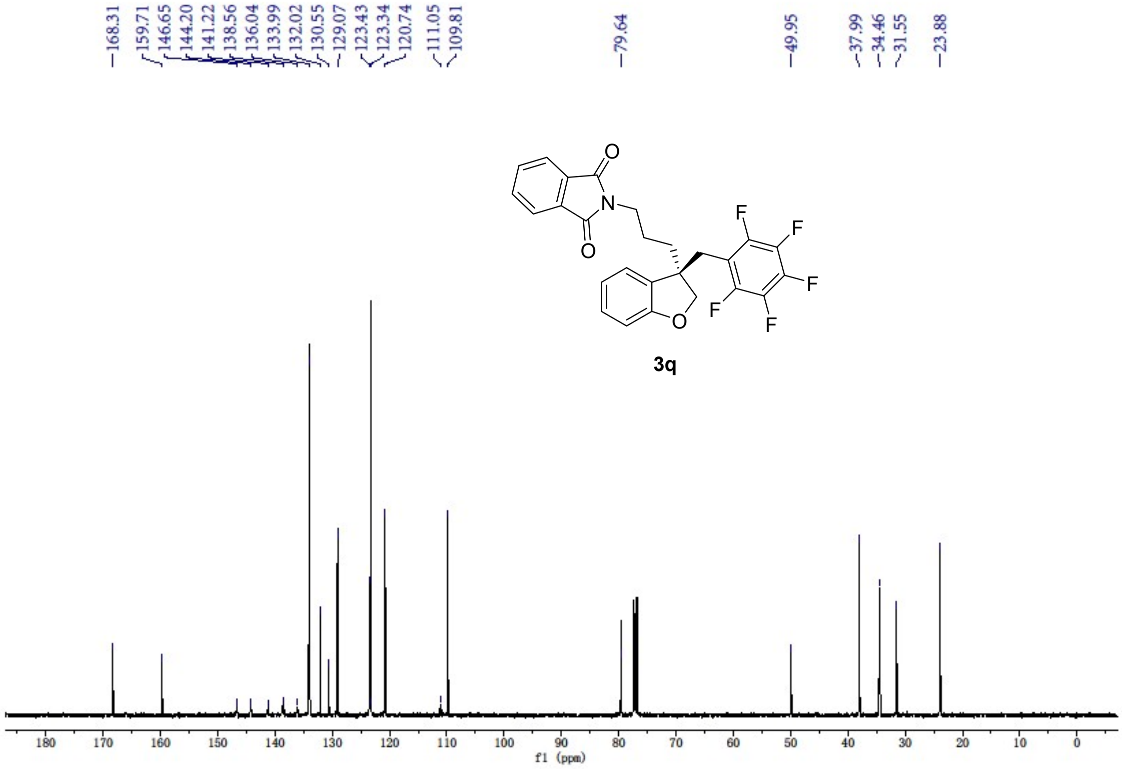
168.31
159.71
146.65
144.20
141.22
138.56
136.04
133.99
132.02
130.55
129.07
123.43
123.34
120.74
111.05
109.81
79.64
49.95
37.99
34.46
31.55
23.88
3q
f1 (ppm)

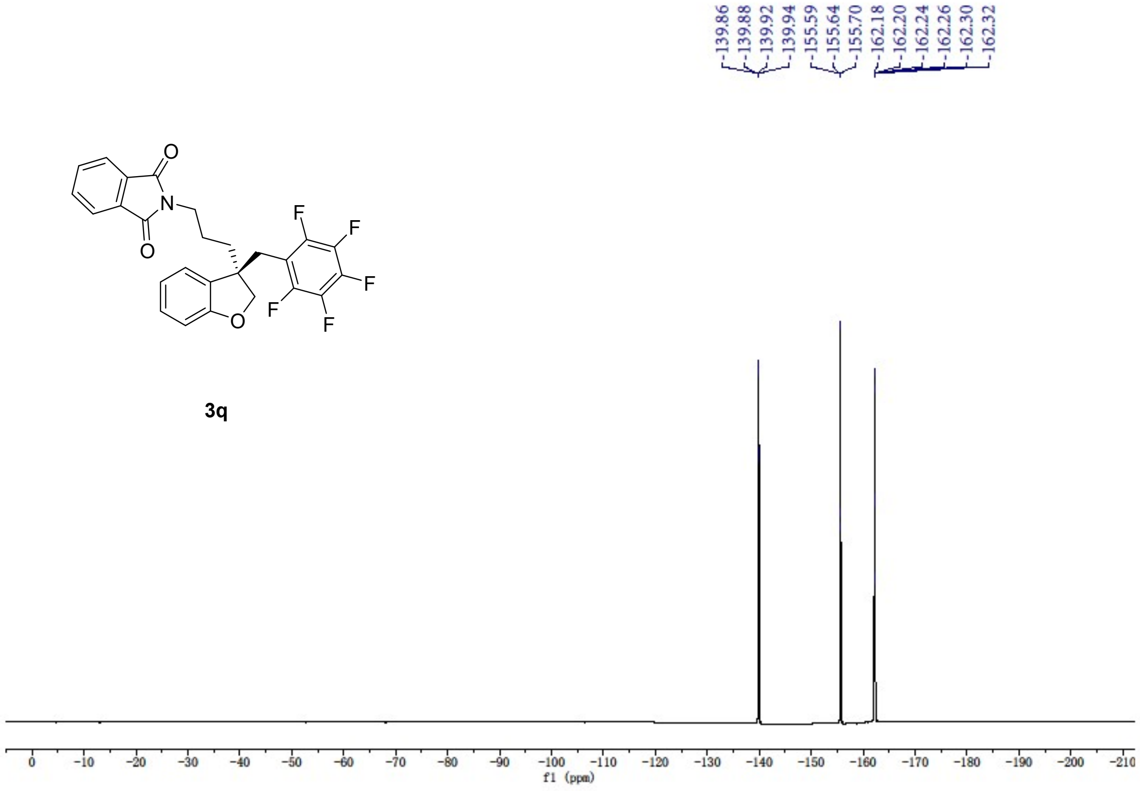
-139.86
-139.88
-139.92
-139.94
-155.59
-155.64
-155.70
-162.18
-162.20
-162.24
-162.26
-162.30
-162.32
3q
f1 (ppm)

<Chromatogram>

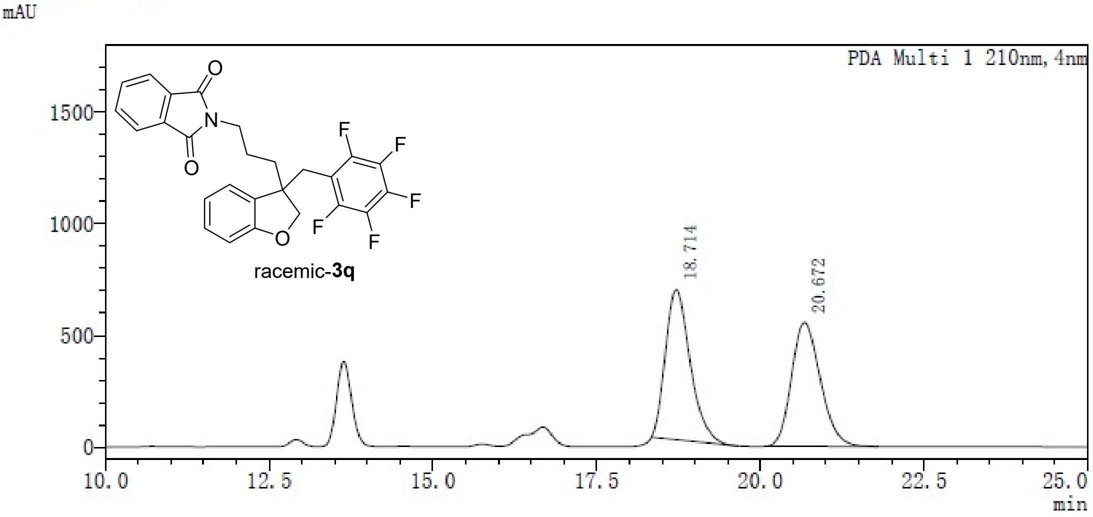

<Peak Table>

PDA Ch1 210nm

No.	Ret. Time (min)	Height	Height%	Area	Area%
1	18.714	672354	54.813	17713947	50.964
2	20.672	554279	45.187	17043750	49.036
Total		1226633	100.000	34757696	100.000

<Chromatogram>

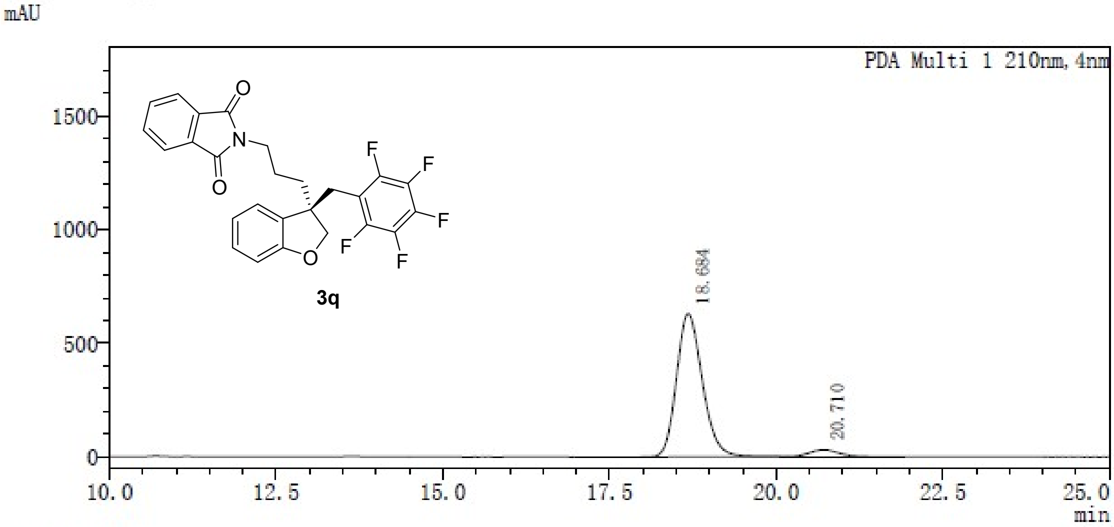

<Peak Table>

PDA Ch1 210nm

No.	Ret. Time (min)	Height	Height%	Area	Area%
1	18.684	629709	95.318	17061797	94.498
2	20.710	30929	4.682	993337	5.502
Total		660638	100.000	18055135	100.000

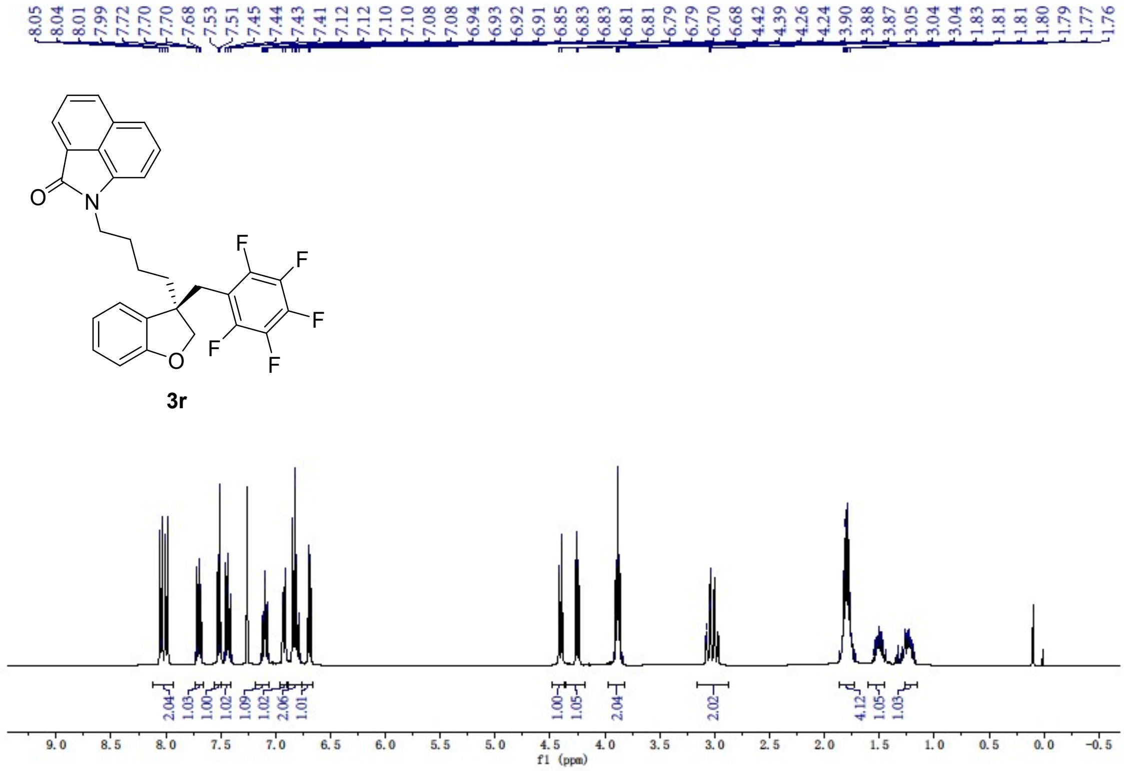
8.05
8.04
8.01
7.99
7.72
7.70
7.70
7.68
7.53
7.51
7.45
7.44
7.43
7.41
7.12
7.12
7.10
7.10
7.08
7.08
6.94
6.93
6.92
6.91
6.85
6.83
6.83
6.81
6.81
6.79
6.79
6.70
6.68
4.42
4.39
4.26
4.24
3.90
3.88
3.87
3.05
3.04
3.04
1.83
1.81
1.81
1.80
1.79
1.77
1.76
O
N
F
F
F
F
F
O
3r
2.04
1.03
1.00
1.02
1.09
1.02
2.06
1.01
1.00
1.05
2.04
2.02
4.12
1.05
1.03
f1 (ppm)

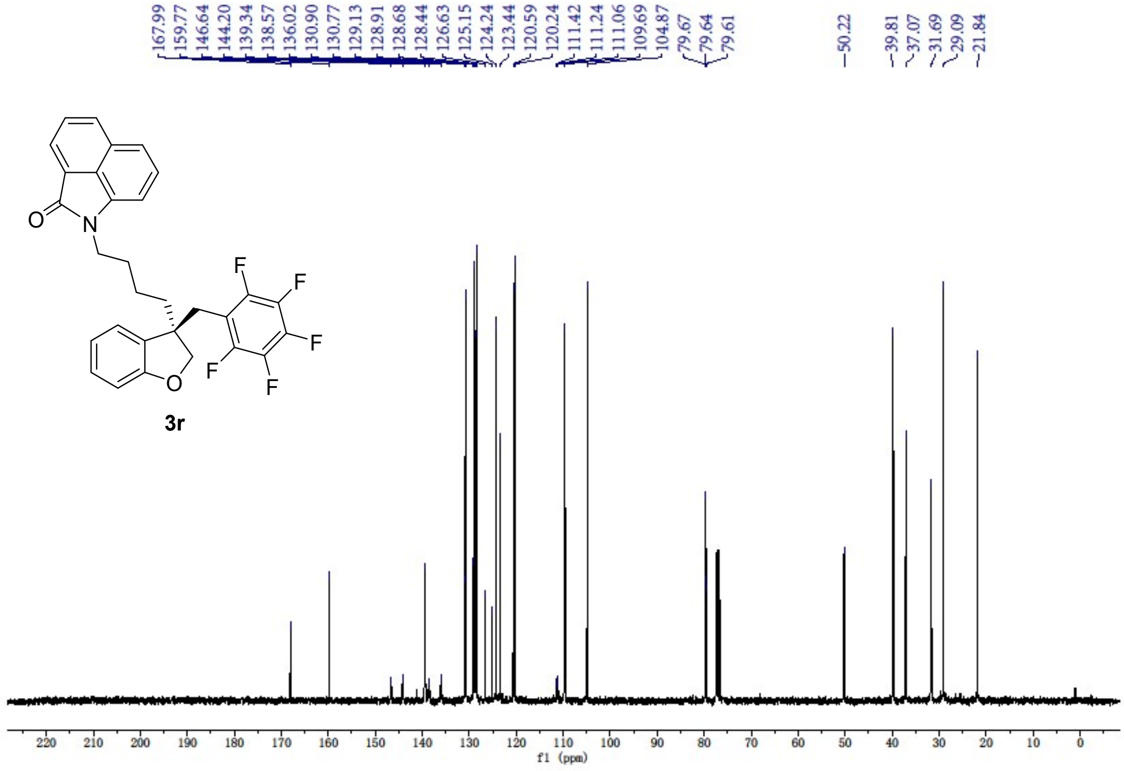
167.99
159.77
146.64
144.20
139.34
138.57
136.02
130.90
130.77
129.13
128.91
128.68
128.44
126.63
125.15
124.24
123.44
120.59
120.24
111.42
111.24
111.06
109.69
104.87
79.67
79.64
79.61
50.22
39.81
37.07
31.69
29.09
21.84
O
N
F
F
F
F
F
O
3r
f1 (ppm)

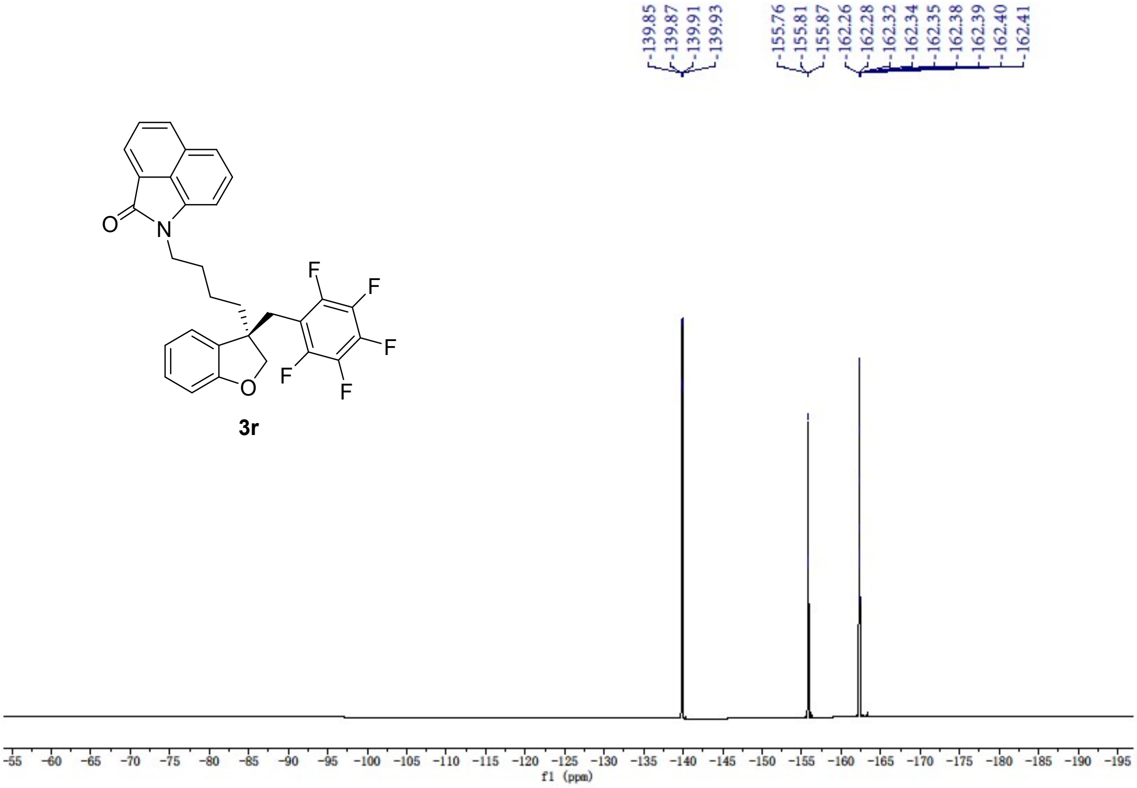

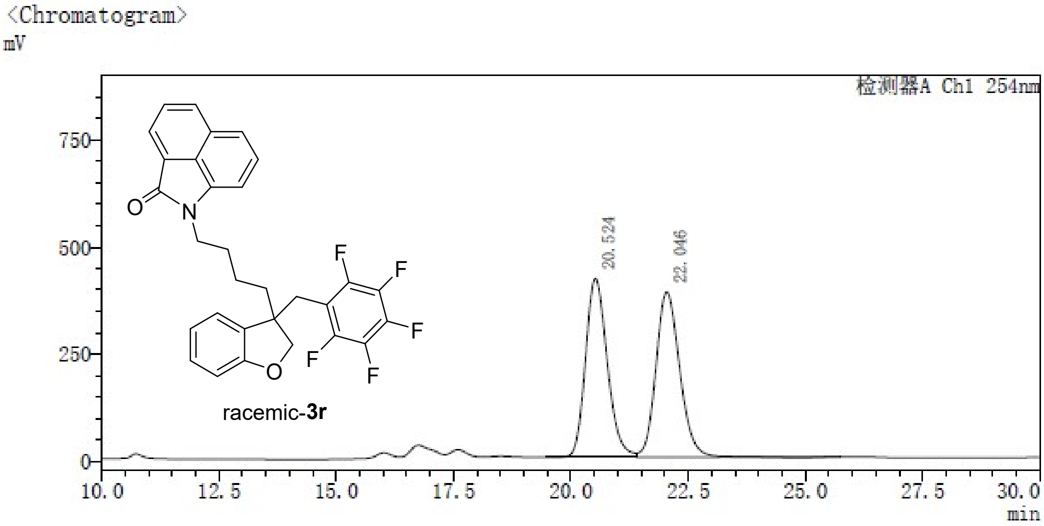

<Peak Table>

检测器A Ch1 254nm

No.	Ret. Time(min)	Height	Height%	Area	Area%
1	20.524	417622	51.986	13518143	50.085
2	22.046	385717	48.014	13472005	49.915
Total		803340	100.000	26990148	100.000

〈Chromatogram〉

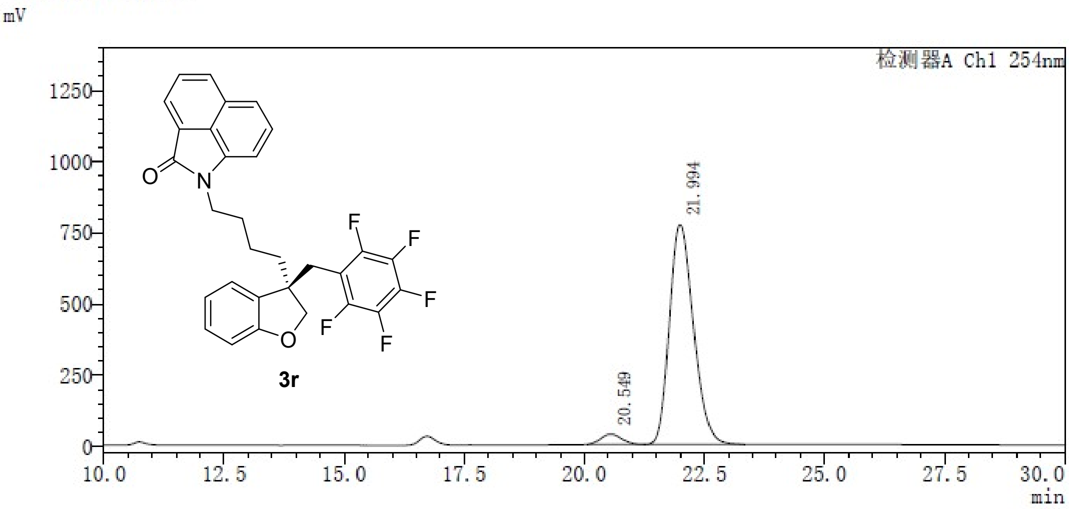

〈Peak Table〉

检测器A Ch1 254nm

No.	Ret. Time (min)	Height	Height%	Area	Area%
1	20.549	36612	4.532	1100871	3.932
2	21.994	771238	95.468	26893828	96.068
Total		807849	100.000	27994700	100.000

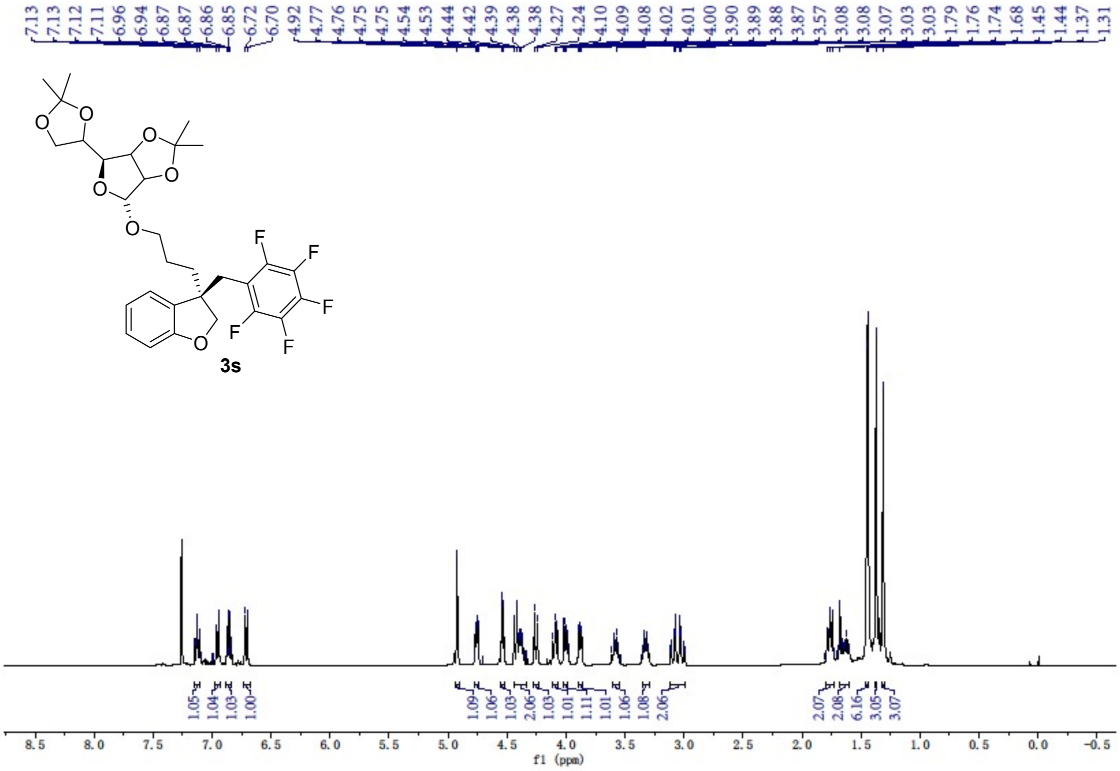

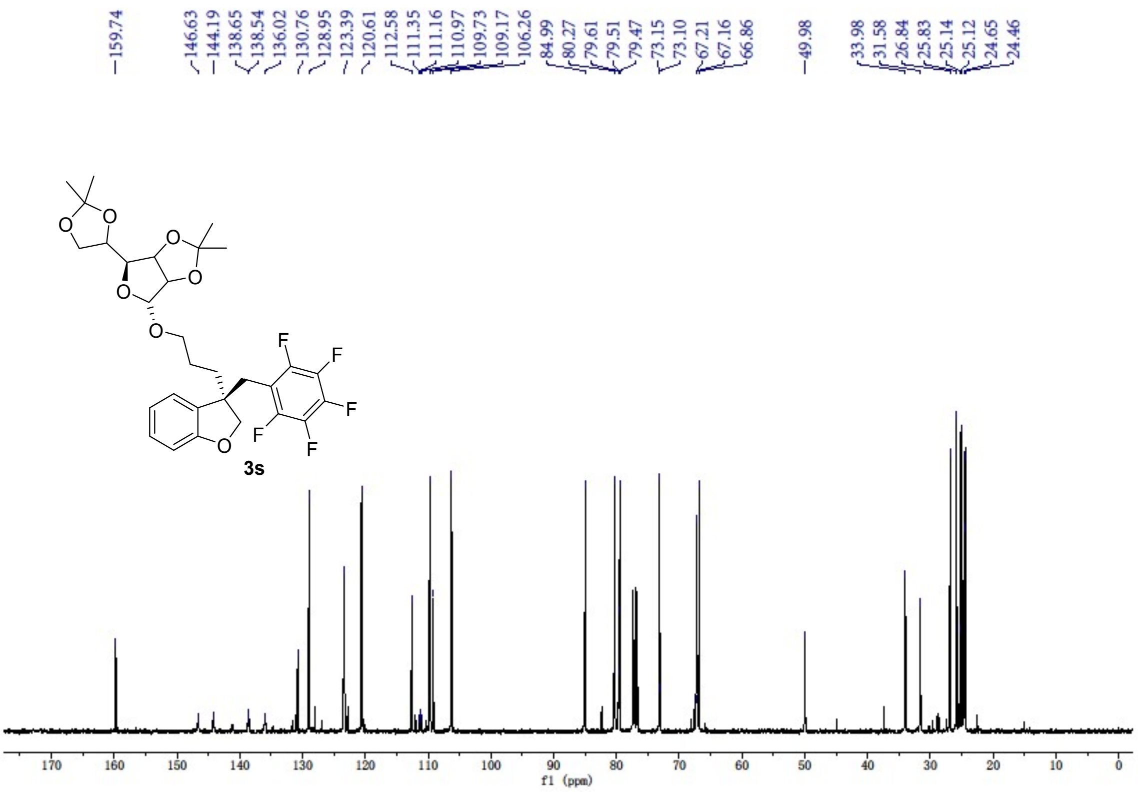
159.74
146.63
144.19
138.65
138.54
136.02
130.76
128.95
123.39
120.61
112.58
111.35
111.16
110.97
109.73
109.17
106.26
84.99
80.27
79.61
79.51
79.47
73.15
73.10
67.21
67.16
66.86
49.98
33.98
31.58
26.84
25.83
25.14
25.12
24.65
24.46
3s
f1 (ppm)

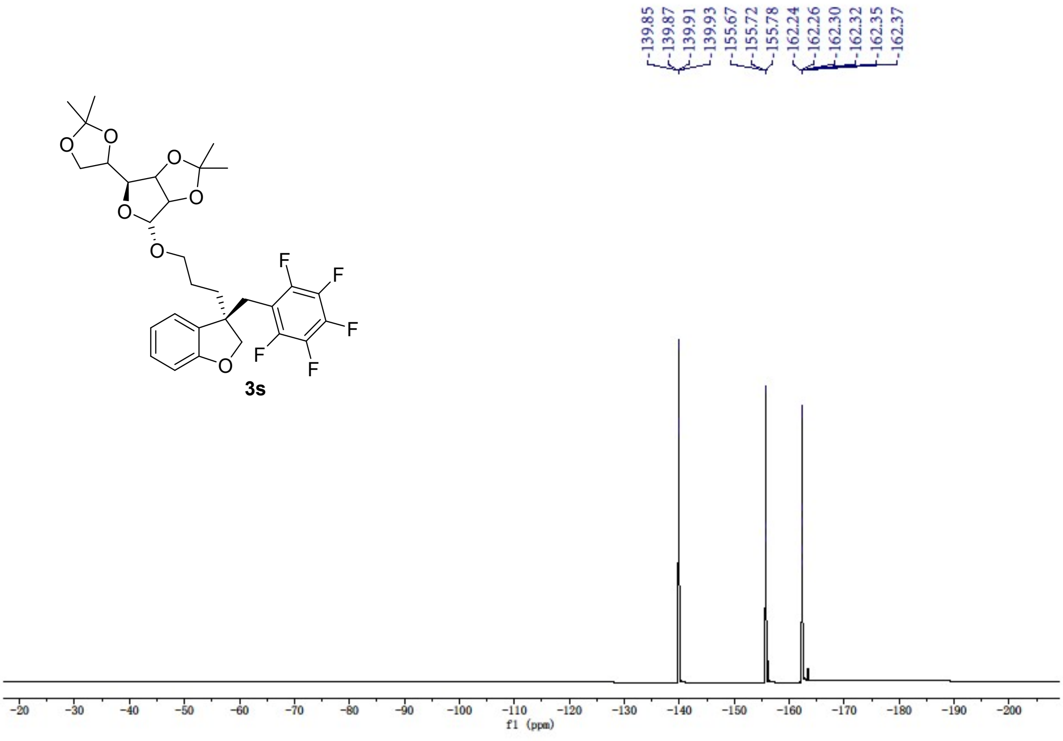
-139.85
-139.87
-139.91
-139.93
-155.67
-155.72
-155.78
-162.24
-162.26
-162.30
-162.32
-162.35
-162.37
3s
f1 (ppm)

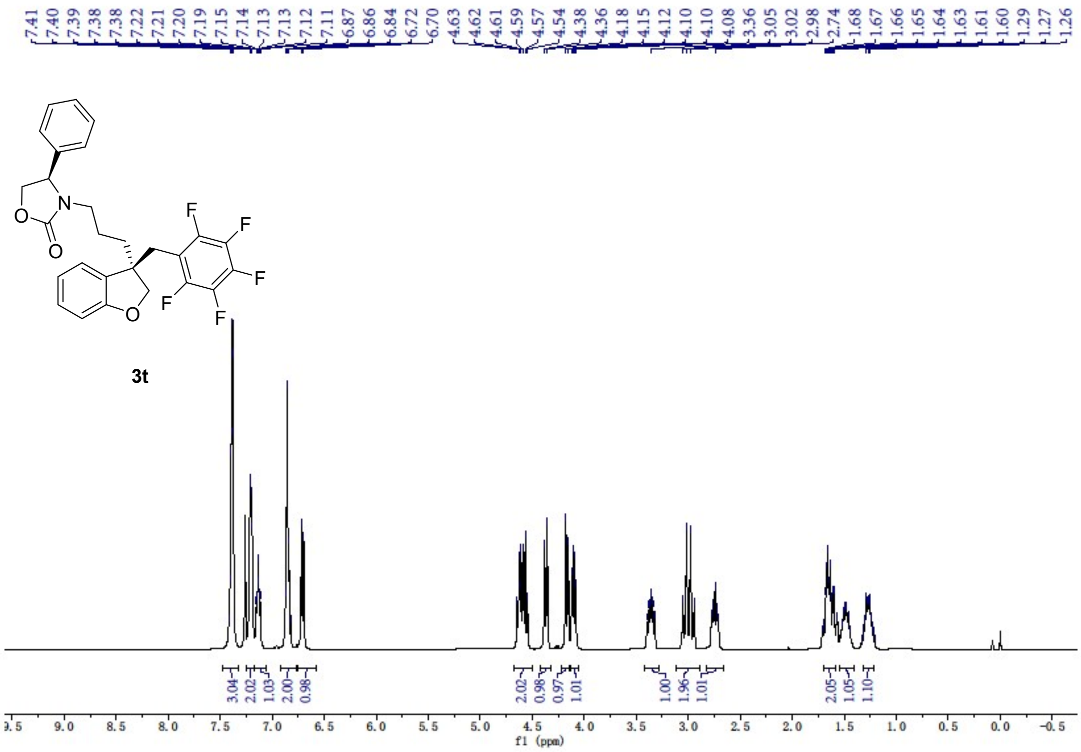
7.41 7.40 7.39 7.38 7.38 7.22 7.21 7.20 7.19 7.15 7.14 7.13 7.13 7.12 7.11 6.87 6.86 6.84 6.72 6.70 4.63 4.62 4.61 4.59 4.57 4.54 4.38 4.36 4.18 4.15 4.12 4.10 4.10 4.08 3.36 3.05 3.02 2.98 2.74 1.68 1.67 1.66 1.65 1.64 1.63 1.61 1.60 1.29 1.27 1.26
3t
3.04 2.02 1.03 2.00 0.98 2.02 0.98 0.97 1.01 1.00 1.96 1.01 2.05 1.05 1.10
f1 (ppm)

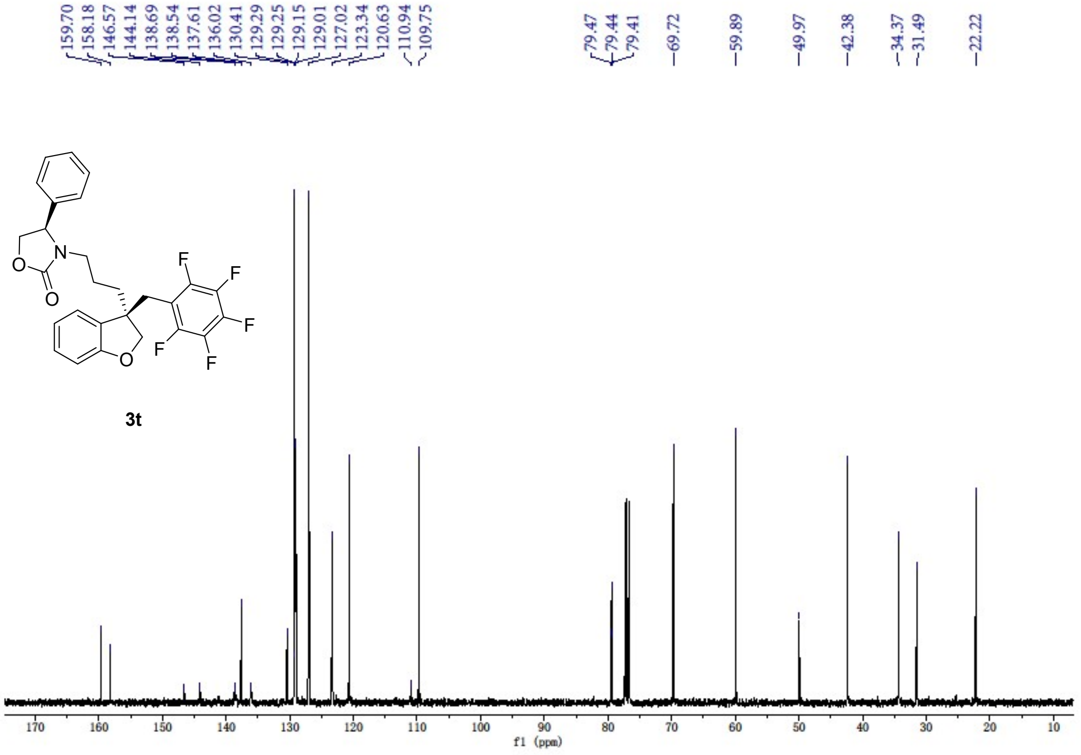
159.70 158.18 146.57 144.14 138.69 138.54 137.61 136.02 130.41 129.29 129.25 129.15 129.01 127.02 123.34 120.63 110.94 109.75 79.47 79.44 79.41 69.72 59.89 49.97 42.38 34.37 31.49 22.22
3t
f1 (ppm)

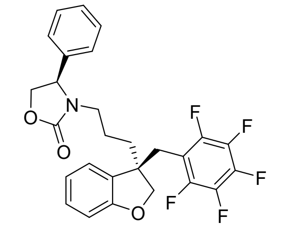

3t

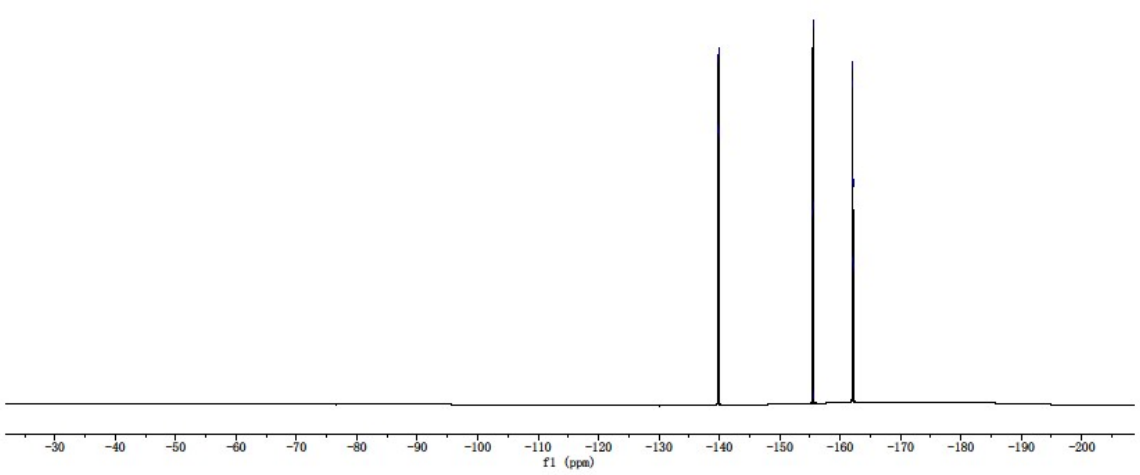
-139.84
-139.86
-139.90
-139.92
-155.44
-155.49
-155.55
-155.59
-162.03
-162.06
-162.09
-162.11
-162.15
-162.17
f1 (ppm)

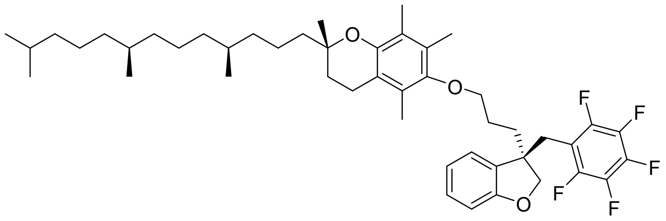

3u

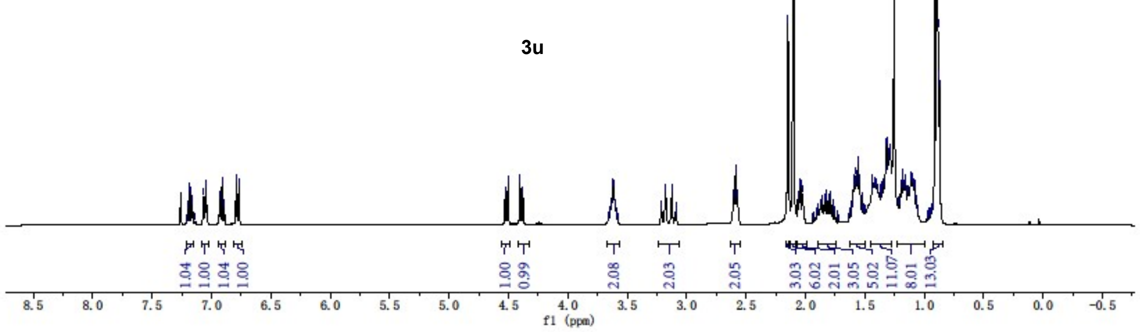
f1 (ppm)

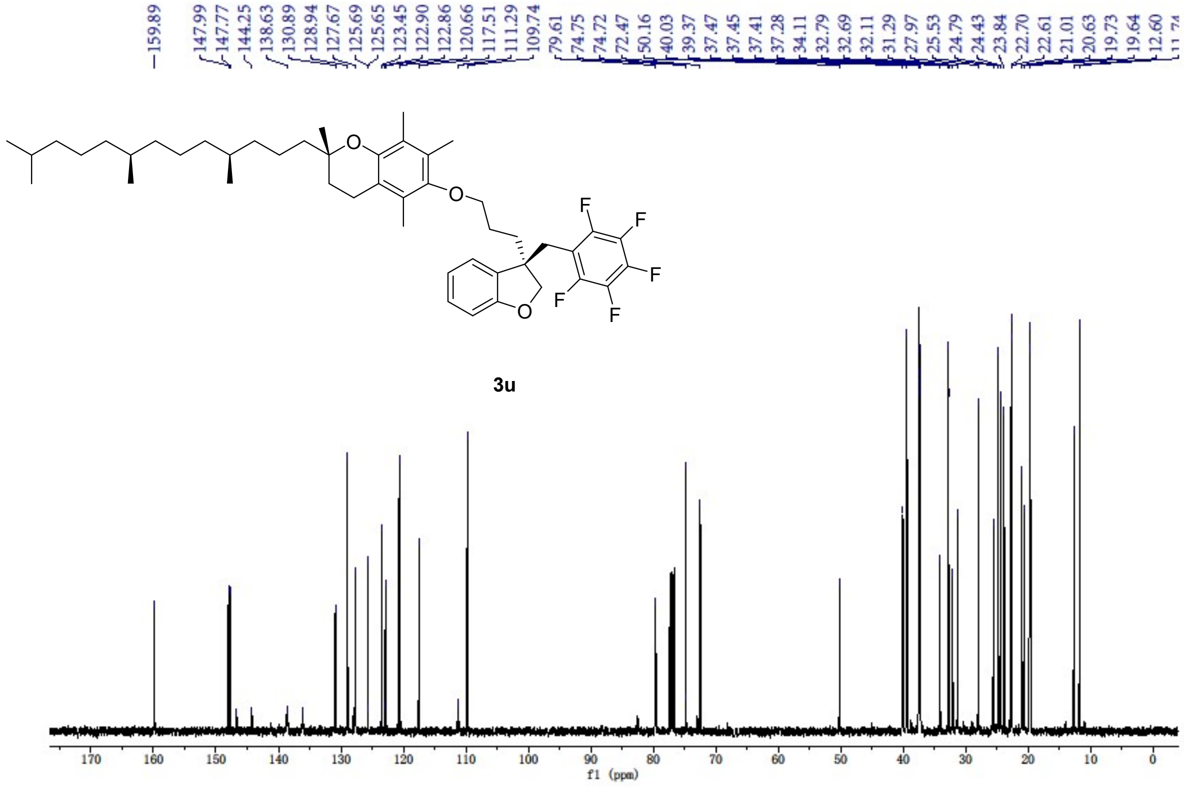
159.89
147.99
147.77
144.25
138.63
130.89
128.94
127.67
125.69
125.65
123.45
122.90
122.86
120.66
117.51
111.29
109.74
79.61
74.75
74.72
72.47
50.16
40.03
39.37
37.47
37.45
37.41
37.28
34.11
32.79
32.69
32.11
31.29
27.97
25.53
24.79
24.43
23.84
22.70
22.61
21.01
20.63
19.73
19.64
12.60
O
O
O
F
F
F
F
F
f1 (ppm)

3u

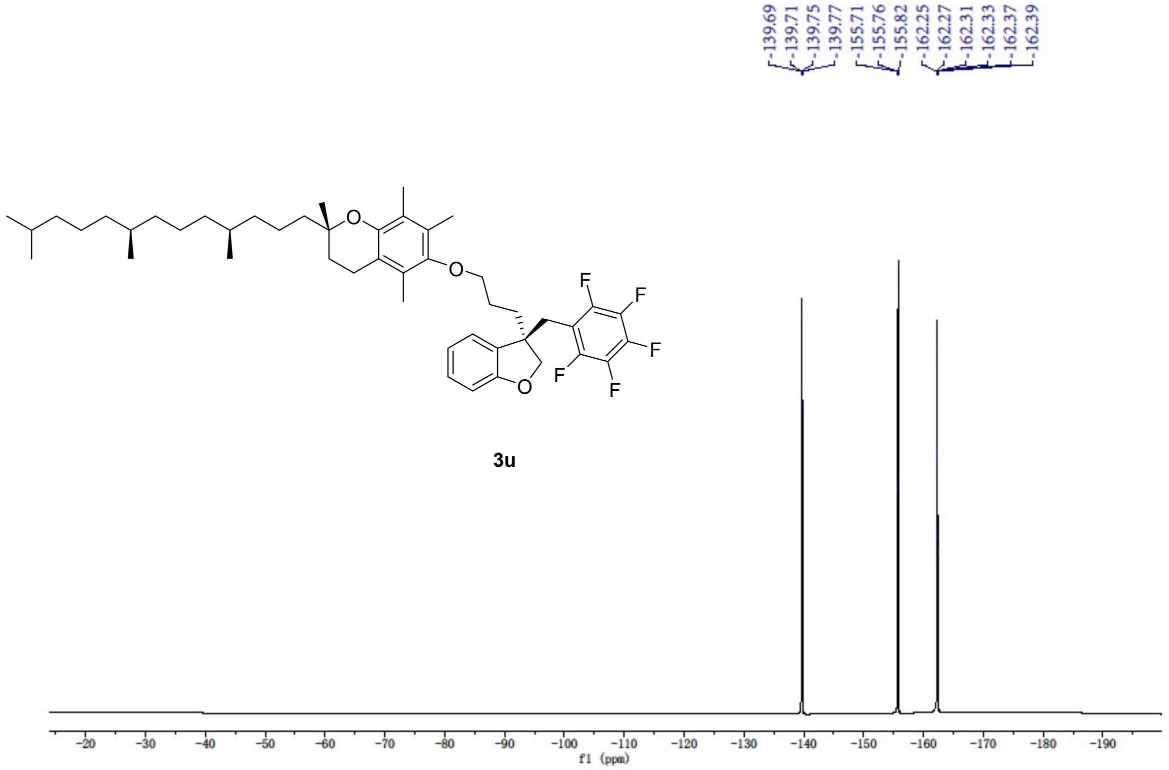
-139.69
-139.71
-139.75
-139.77
-155.71
-155.76
-155.82
-162.25
-162.27
-162.31
-162.33
-162.37
-162.39
O
O
O
F
F
F
F
F
f1 (ppm)

3u

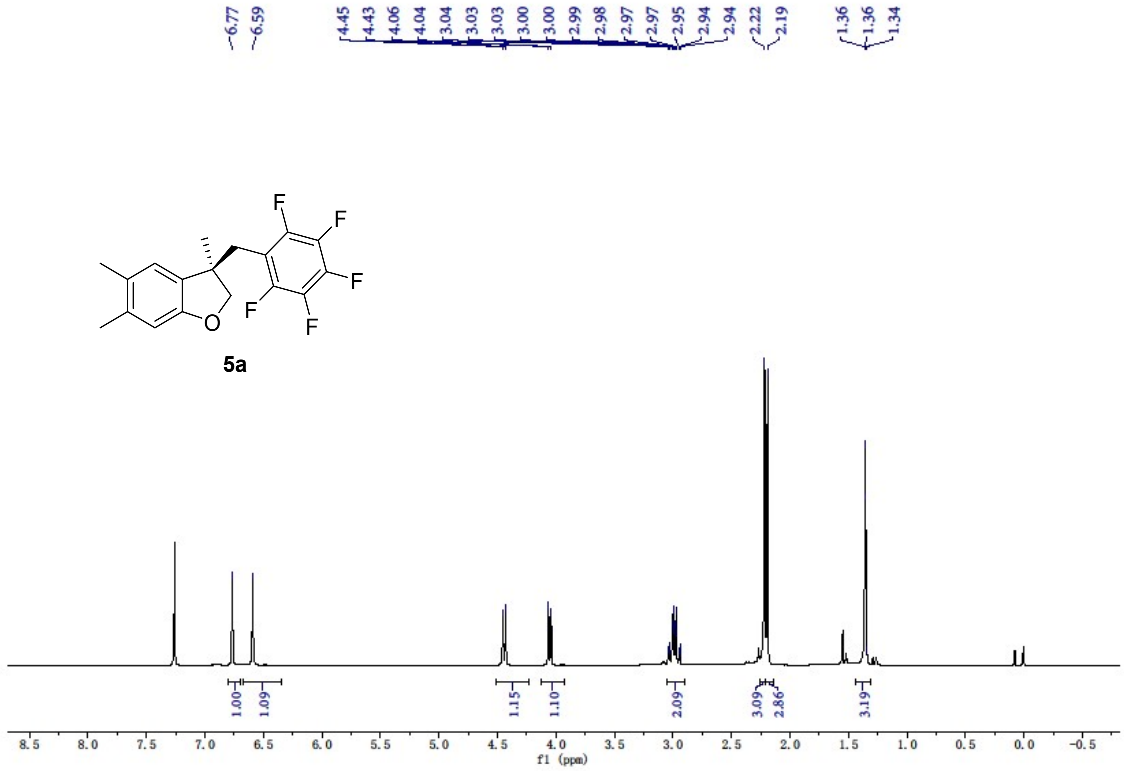
6.77
6.59
4.45
4.43
4.06
4.04
3.04
3.03
3.03
3.00
3.00
2.99
2.98
2.97
2.97
2.95
2.94
2.94
2.22
2.19
1.36
1.36
1.34
5a
1.00
1.09
1.15
1.10
2.09
3.09
2.86
3.19
f1 (ppm)

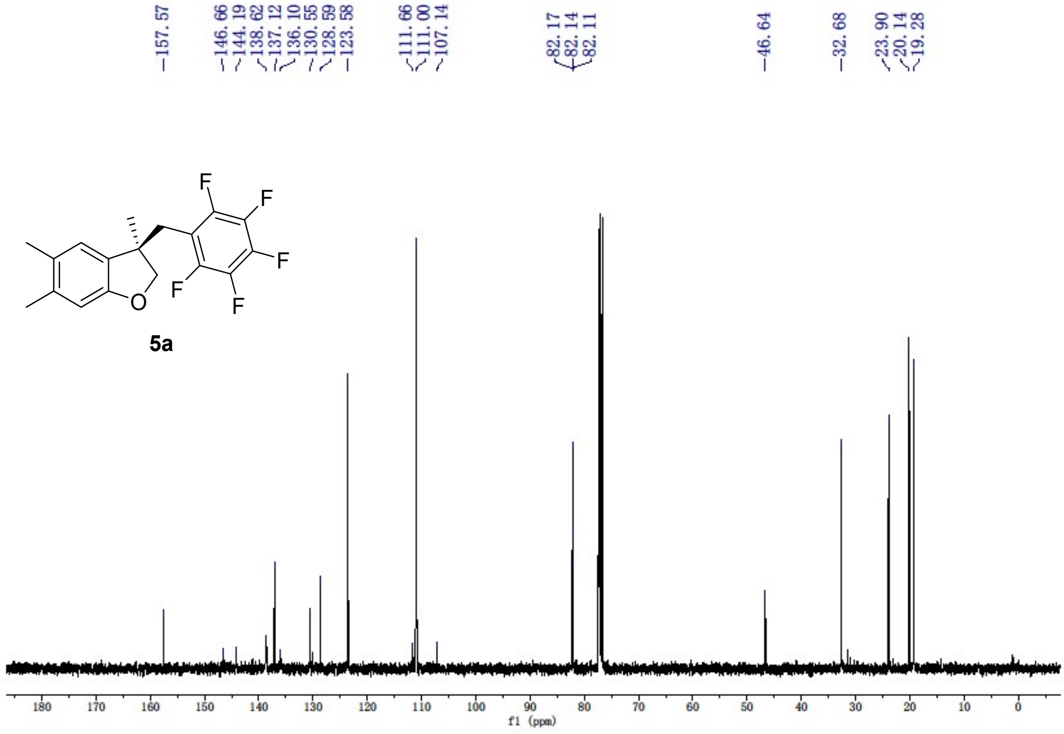
157.57
146.66
144.19
138.62
137.12
136.10
130.55
128.59
123.58
111.66
111.00
107.14
82.17
82.14
82.11
46.64
32.68
23.90
20.14
19.28
5a
f1 (ppm)

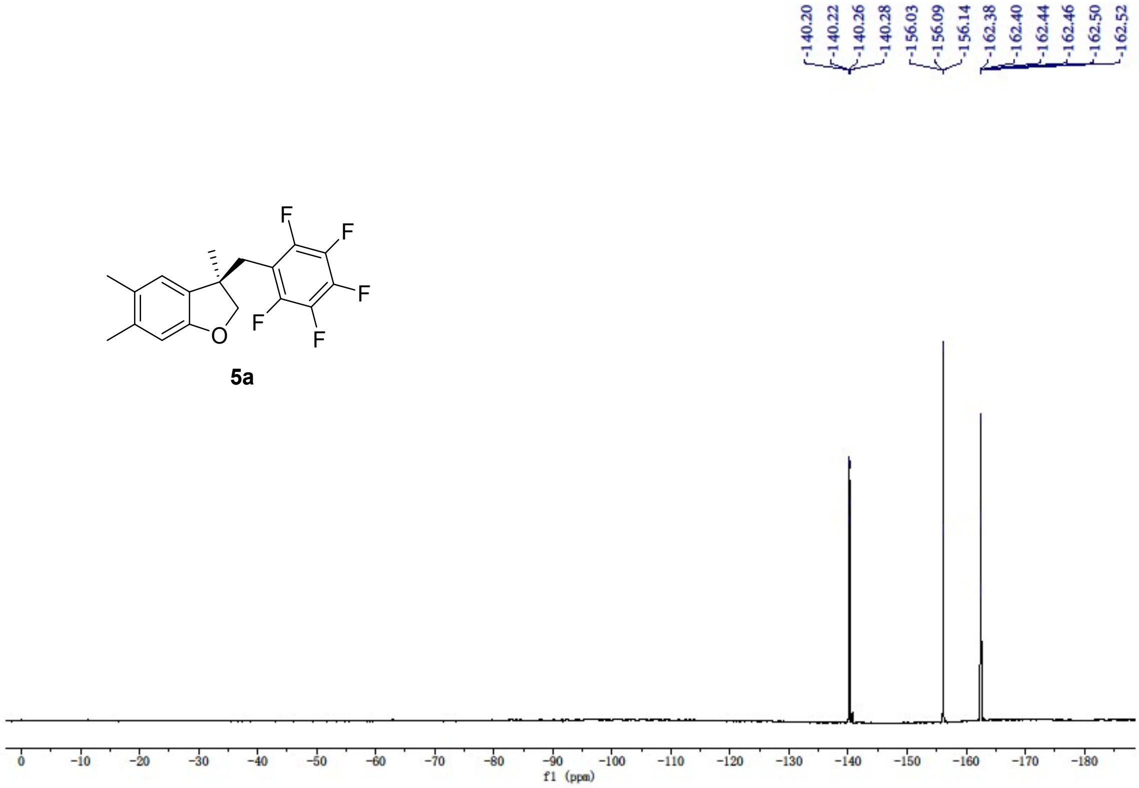

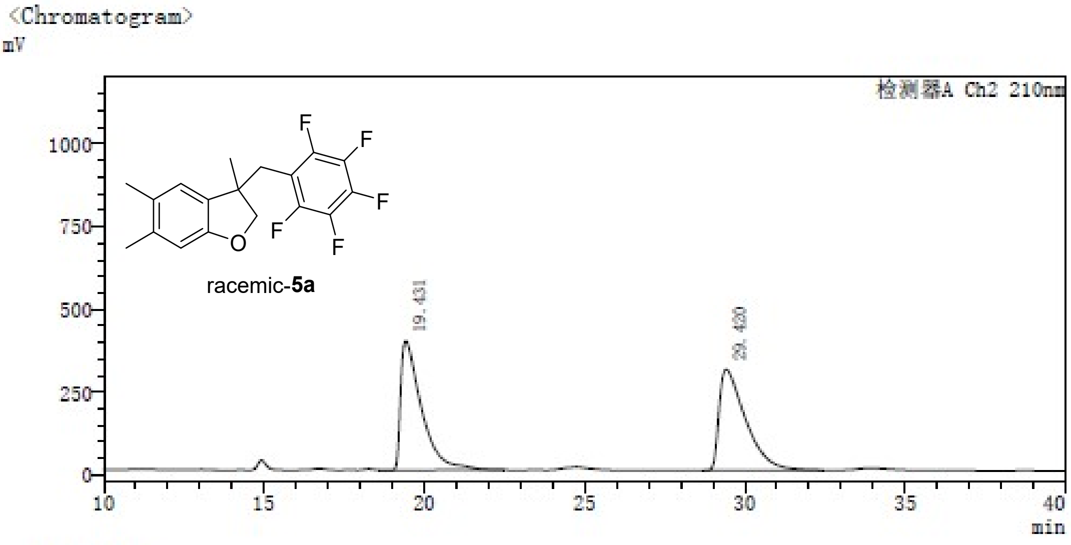

<Peak Table>

检测器A Ch2 210nm

No.	Ret. Time(min)	Height	Height%	Area	Area%
1	19.431	389567	56.127	17686414	50.203
2	29.420	304512	43.873	17543719	49.797
Total		694079	100.000	35230133	100.000

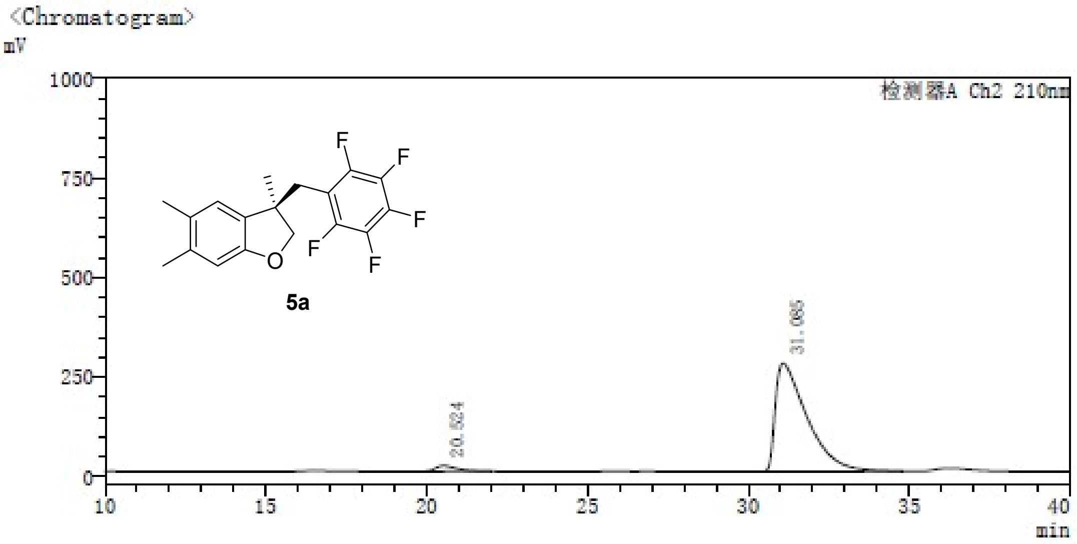

<Peak Table>
检测器A Ch2 210nm

No.	Ret. Time (min)	Height	Height%	Area	Area%
1	20.524	13992	4.894	646014	3.351
2	31.085	271913	95.106	18629591	96.649
Total		285905	100.000	19275605	100.000

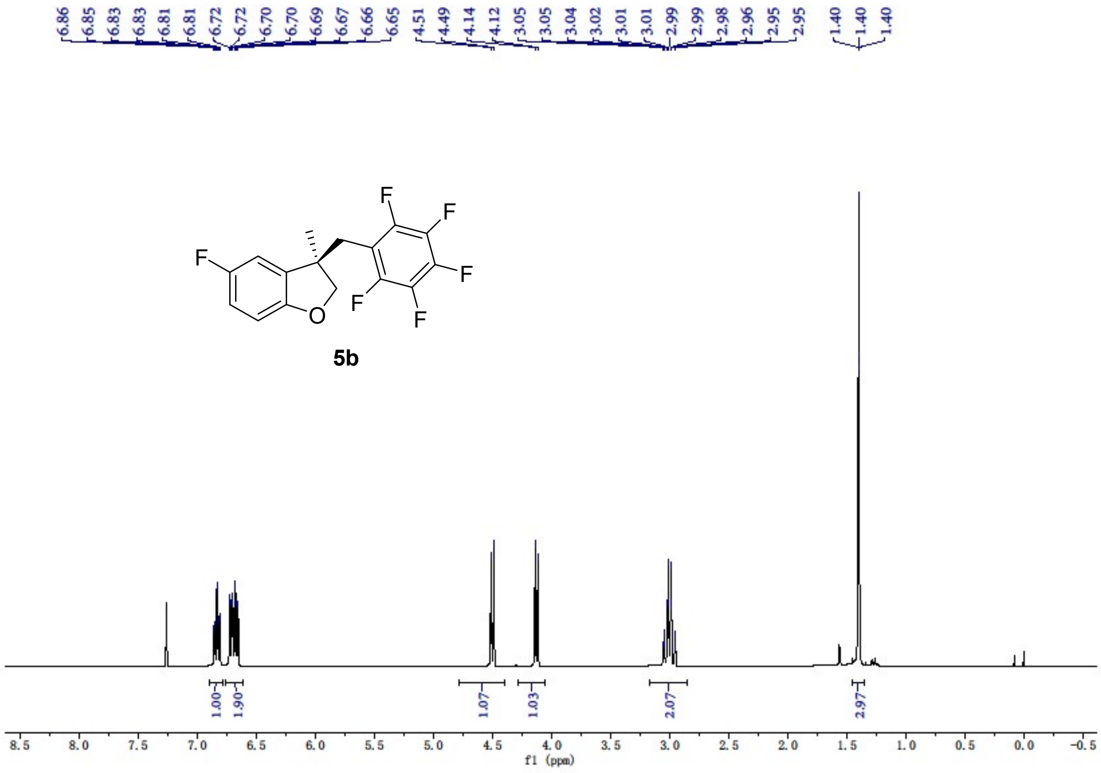

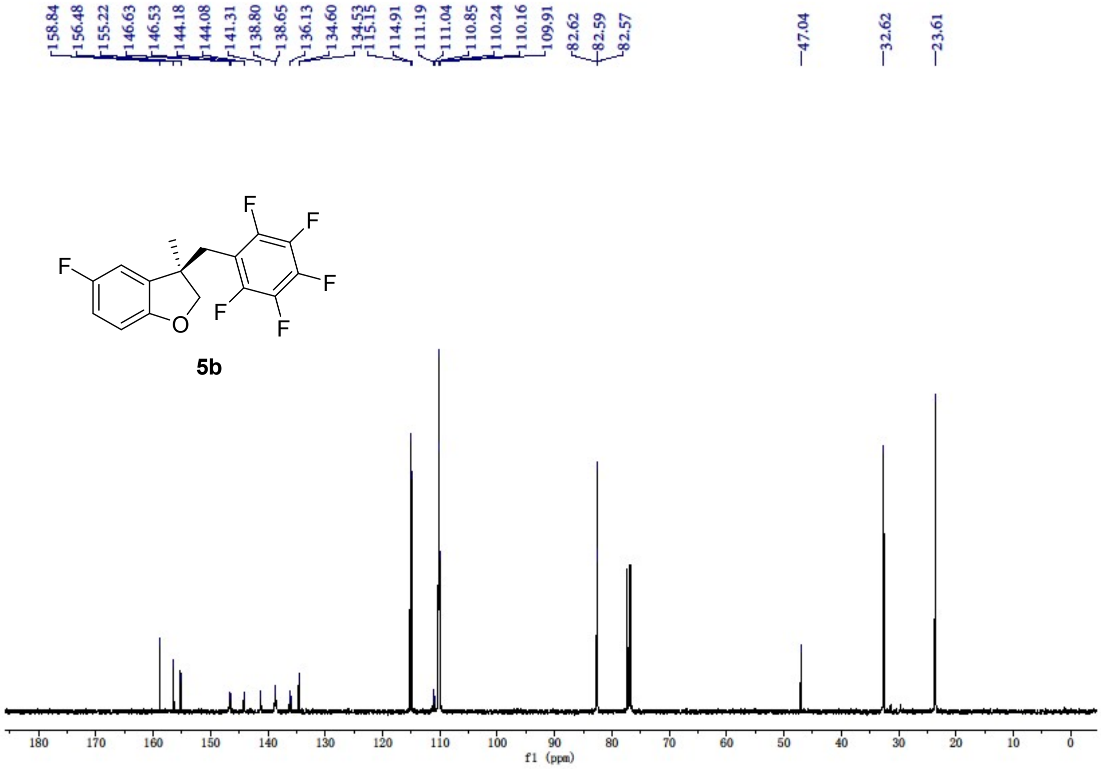

<Chromatogram>

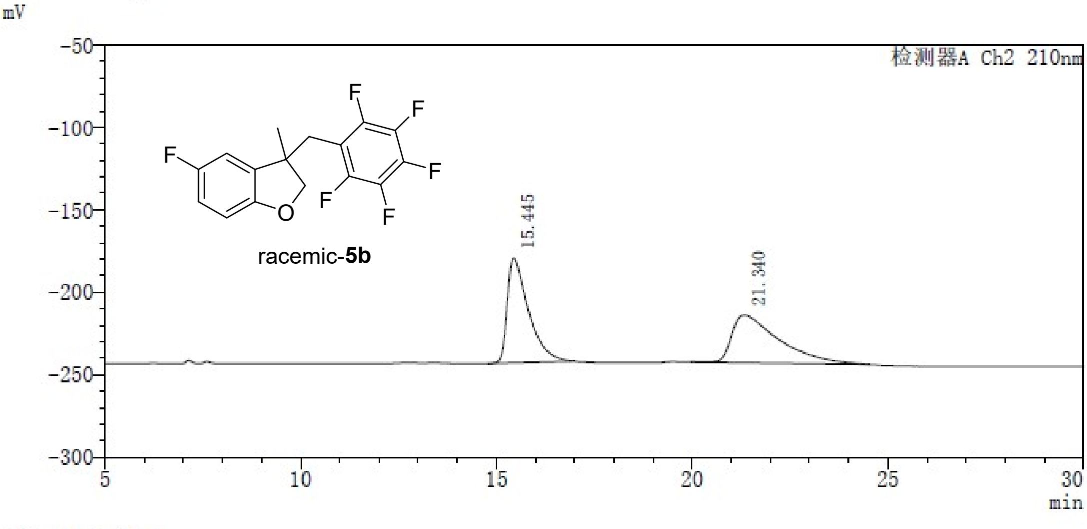

<Peak Table>

検測器A Ch2 210nm

No.	Ret. Time (min)	Height	Height%	Area	Area%
1	15.445	63481	68.730	2401003	49.796
2	21.340	28882	31.270	2420682	50.204
Total		92364	100.000	4821685	100.000

<Chromatogram>

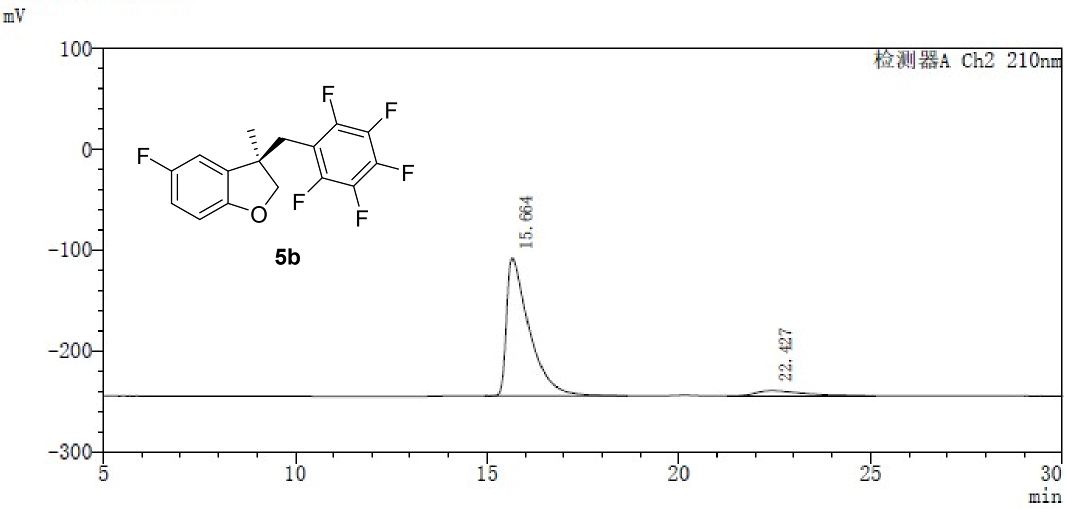

<Peak Table>

检测器A Ch2 210nm

No.	Ret. Time (min)	Height	Height%	Area	Area%
1	15.664	136322	96.292	5630016	93.023
2	22.427	5250	3.708	422287	6.977
Total		141572	100.000	6052304	100.000

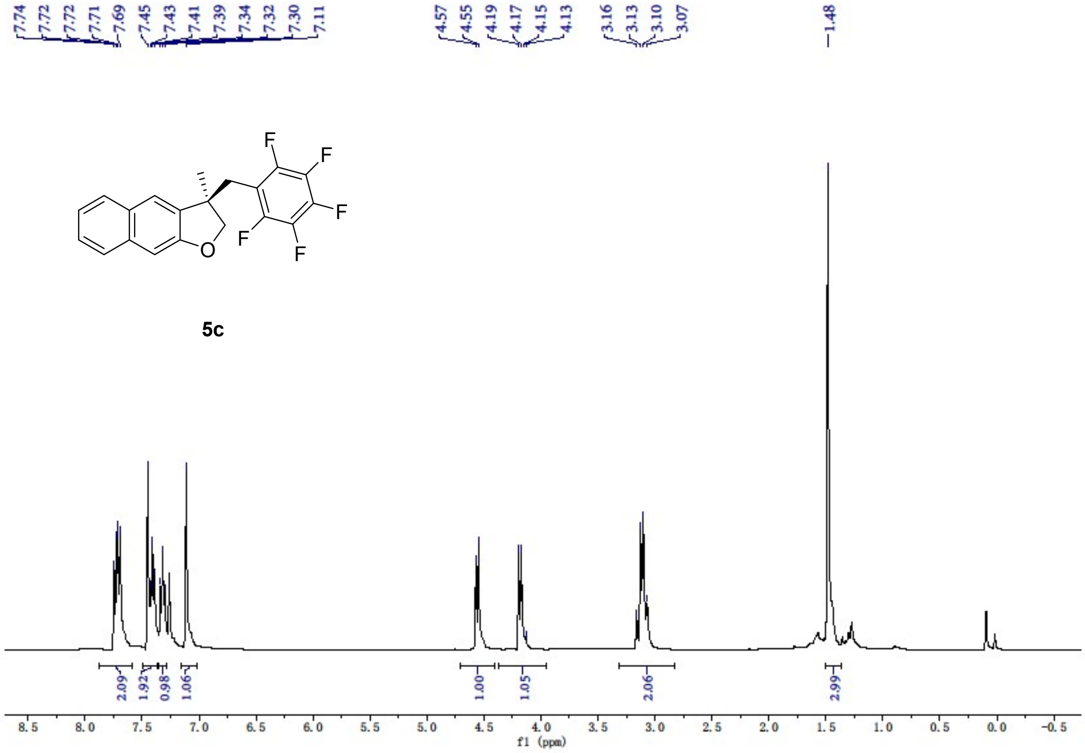

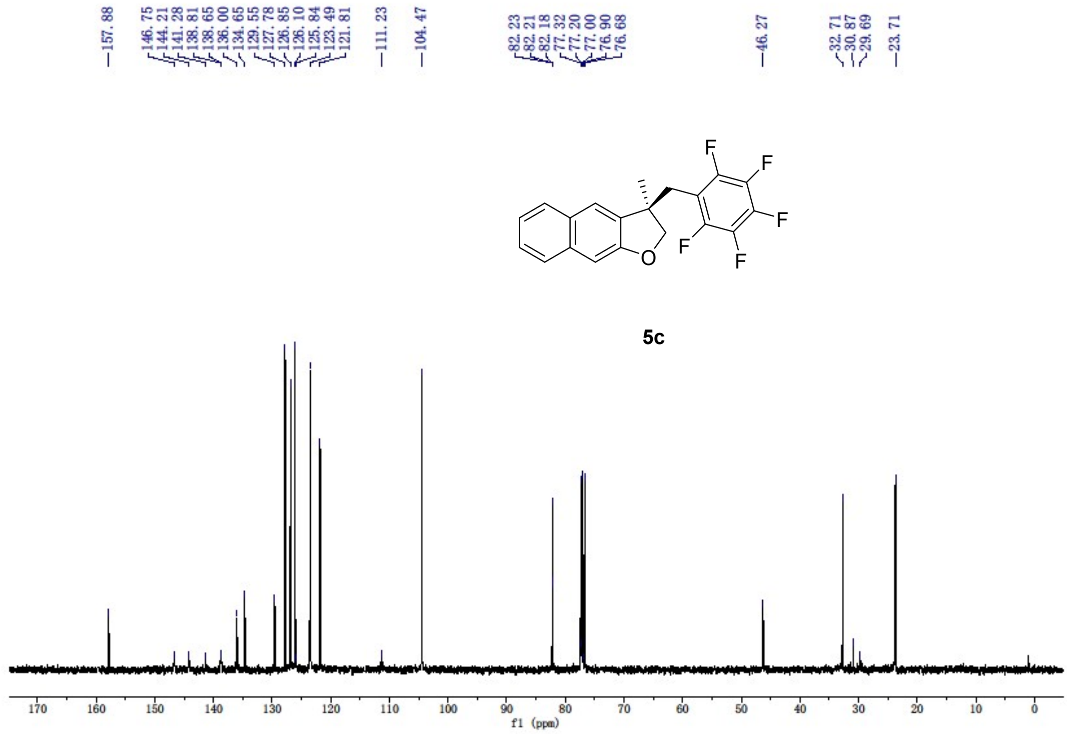
157.88
146.75
144.21
141.28
138.81
138.65
136.00
134.65
129.55
127.78
126.85
126.10
125.84
123.49
121.81
111.23
104.47
82.23
82.21
82.18
77.32
77.20
77.00
76.90
76.68
46.27
32.71
30.87
29.69
23.71
F
F
F
F
F
O
5c
170 160 150 140 130 120 110 100 90 80 70 60 50 40 30 20 10 0
f1 (ppm)

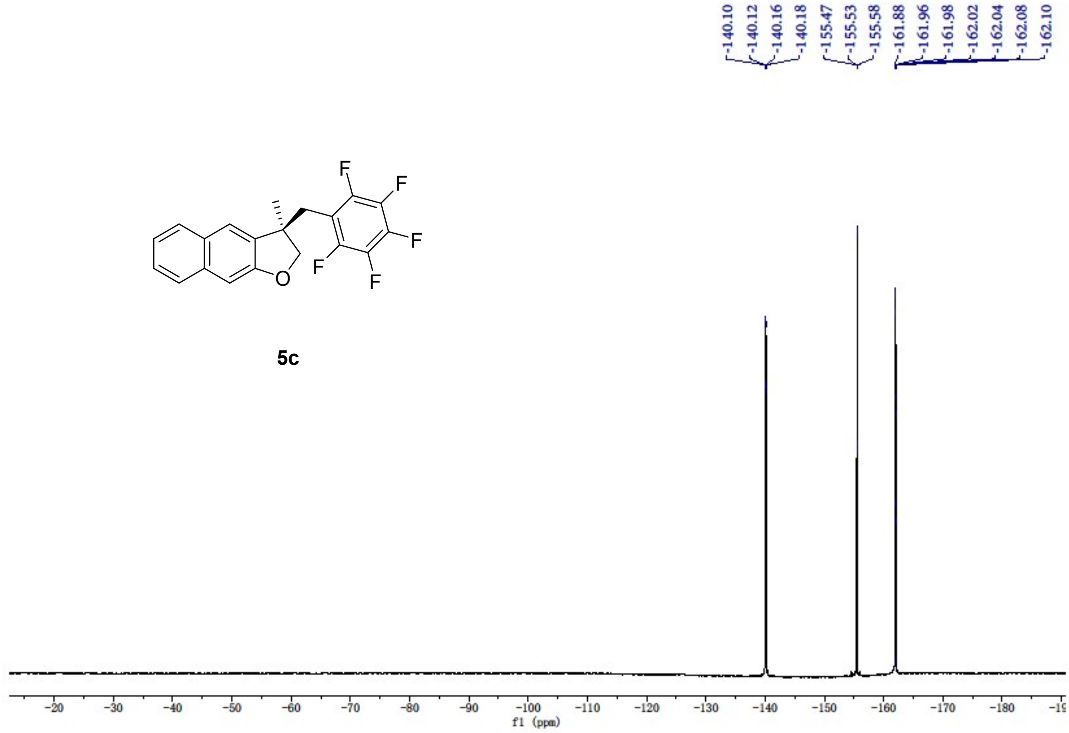
-140.10
-140.12
-140.16
-140.18
-155.47
-155.53
-155.58
-161.88
-161.96
-161.98
-162.02
-162.04
-162.08
-162.10
F
F
F
F
F
O
5c
-20 -30 -40 -50 -60 -70 -80 -90 -100 -110 -120 -130 -140 -150 -160 -170 -180
f1 (ppm)

<Chromatogram>

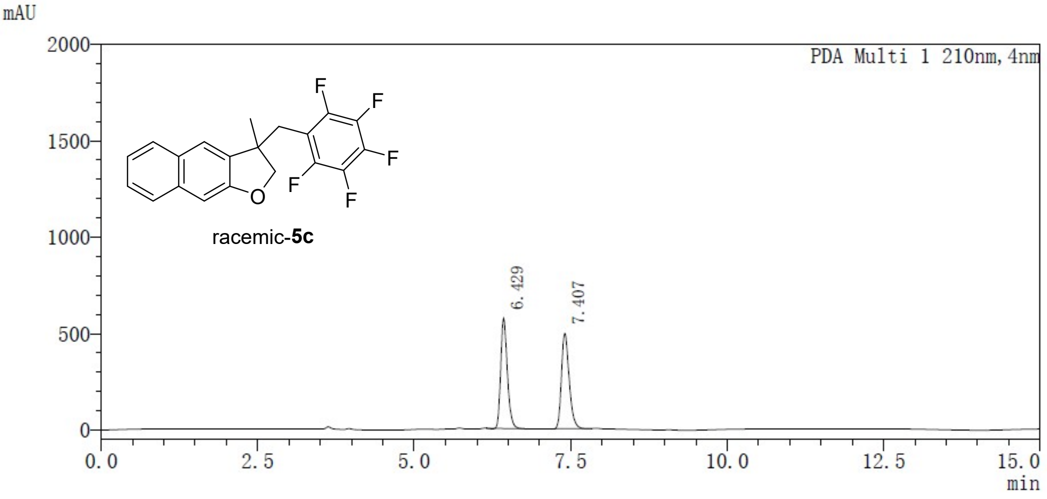

<Peak Table>

PDA Ch1 210nm

No.	Ret. Time (min)	Height	Height%	Area	Area%
1	6.429	571480	53.576	4192264	49.723
2	7.407	495187	46.424	4239026	50.277
Total		1066667	100.000	8431290	100.000

<Chromatogram>

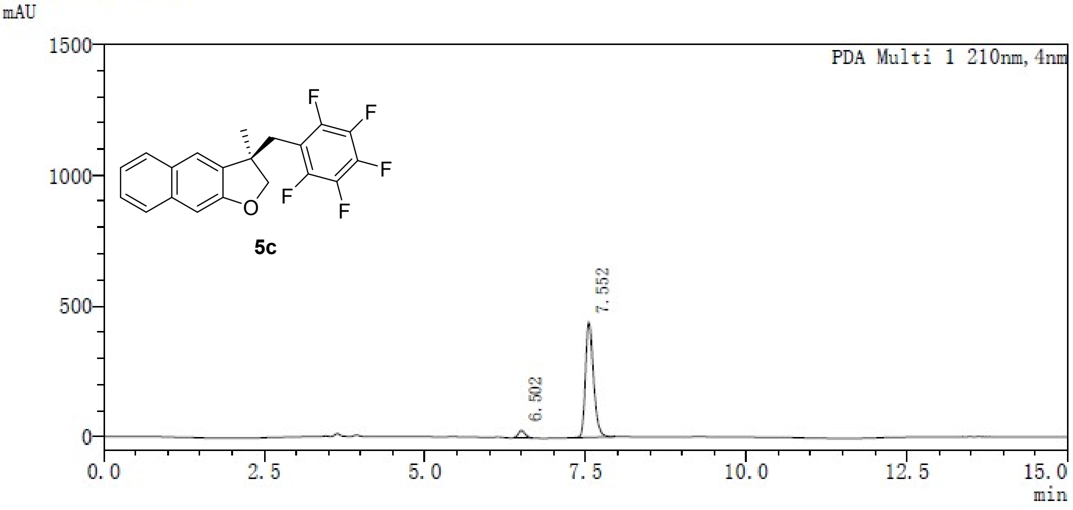

<Peak Table>

PDA Ch1 210nm

No.	Ret. Time (min)	Height	Height%	Area	Area%
1	6.502	27399	5.860	205035	5.093
2	7.552	440197	94.140	3820646	94.907
Total		467596	100.000	4025681	100.000

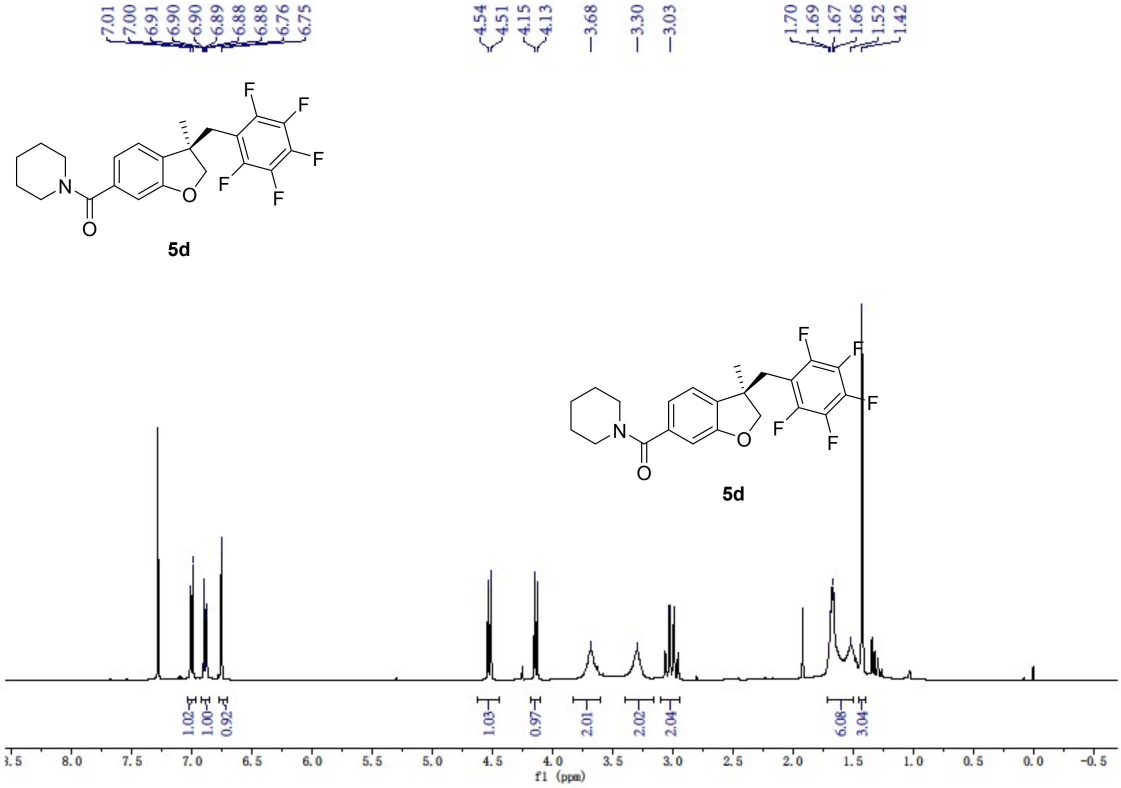
7.01 7.00 6.91 6.90 6.90 6.89 6.88 6.88 6.76 6.75
4.54 4.51 4.15 4.13
3.68
3.30
3.03
1.70 1.69 1.67 1.66 1.52 1.42
5d
5d
1.02 1.00 0.92 1.03 0.97 2.01 2.02 2.04 6.08 3.04
f1 (ppm)

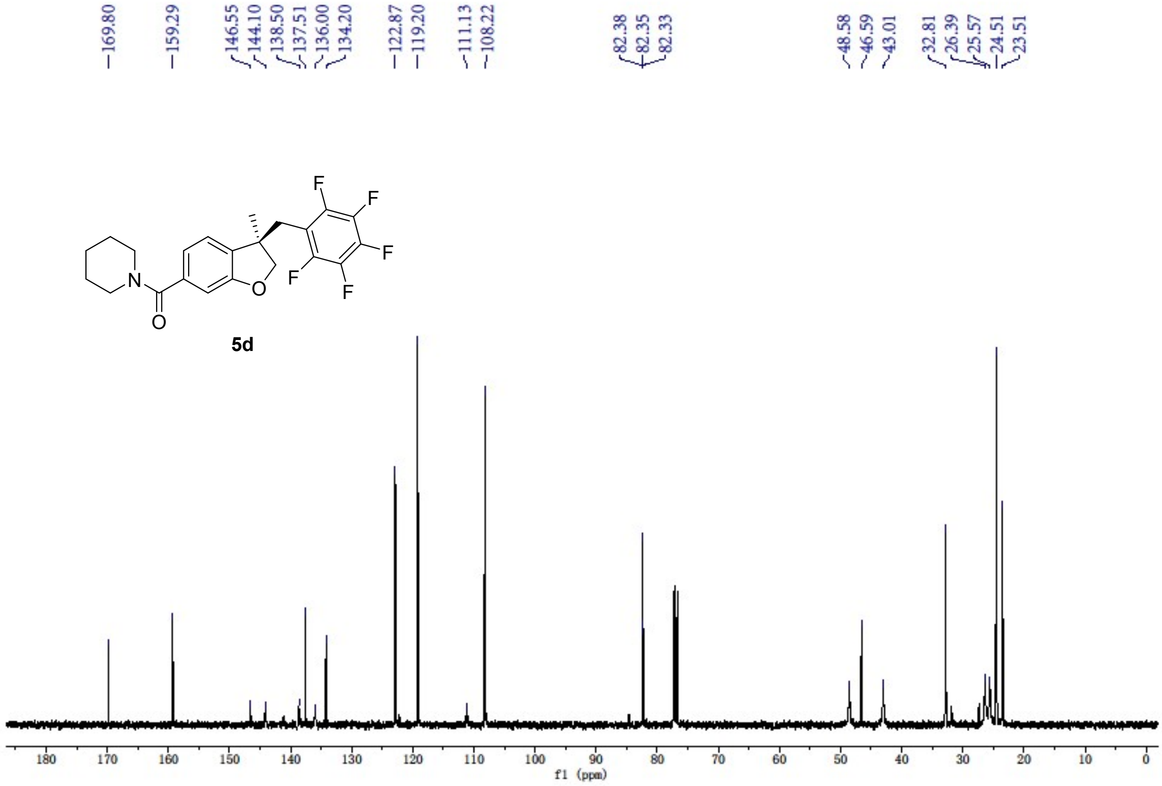
169.80
159.29
146.55 144.10 138.50 137.51 136.00 134.20
122.87 119.20
111.13 108.22
82.38 82.35 82.33
48.58 46.59 43.01
32.81 26.39 25.57 24.51 23.51
5d
f1 (ppm)

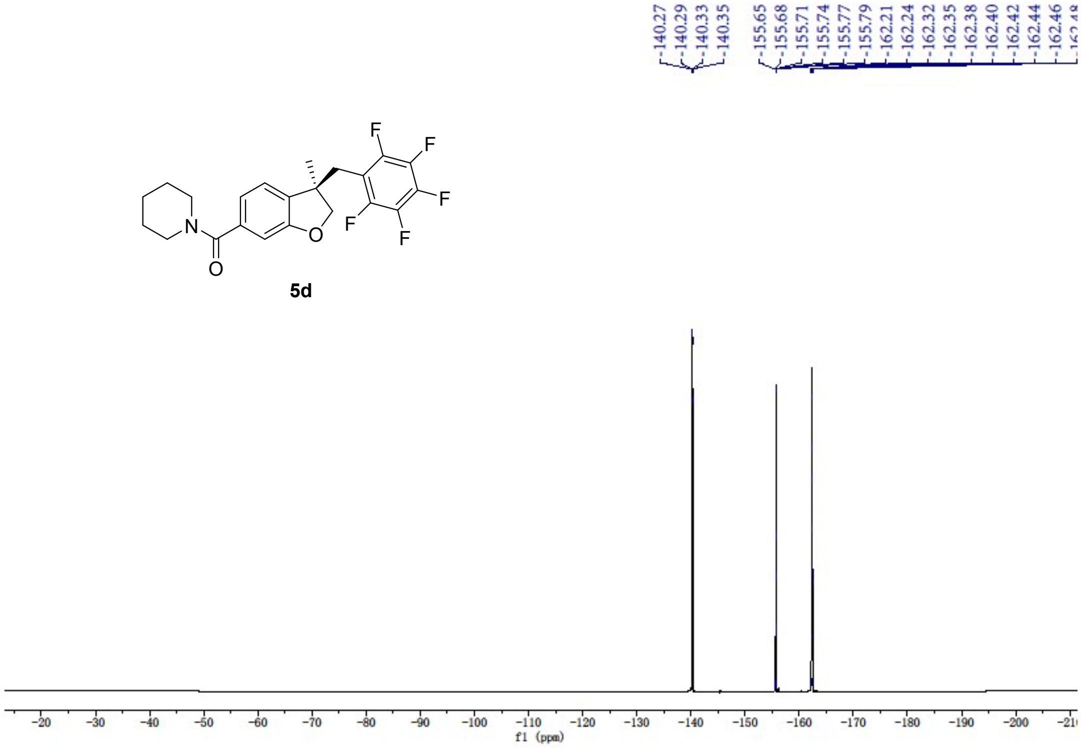

<Chromatogram>

mAU

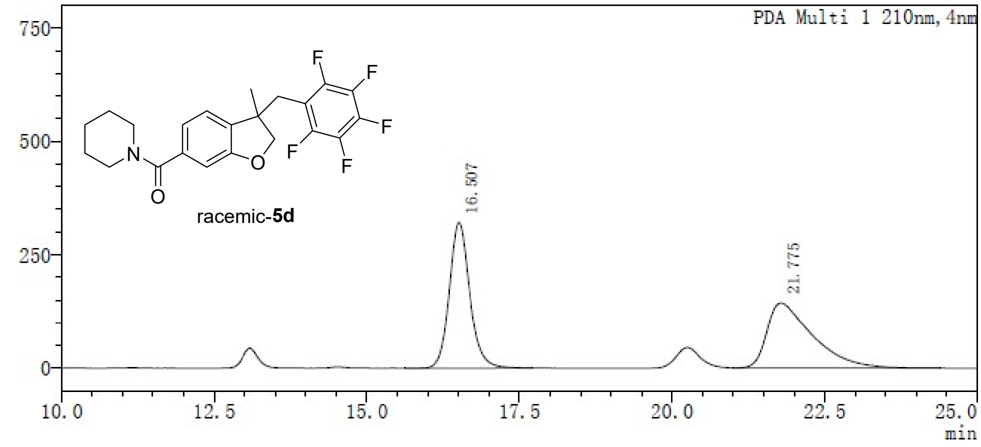

<Peak Table>

PDA Ch1 210nm

No.	Ret. Time (min)	Height	Height%	Area	Area%
1	16.507	321378	69.219	7510249	50.314
2	21.775	142917	30.781	7416518	49.686
Total		464295	100.000	14926766	100.000

<Chromatogram>

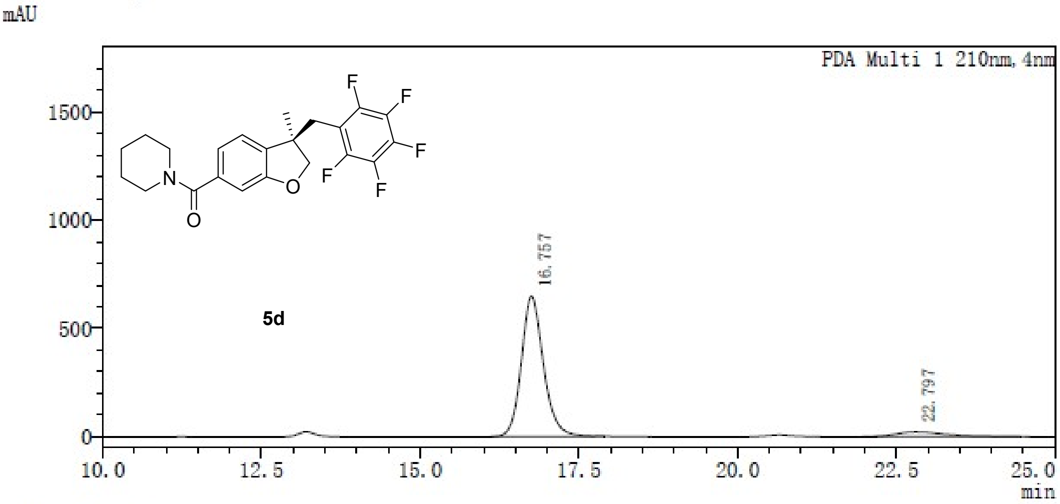

<Peak Table>

PDA Ch1 210nm

No.	Ret. Time(min)	Height	Height%	Area	Area%
1	16.757	648003	96.695	15730956	92.989
2	22.797	22146	3.305	1186085	7.011
Total		670149	100.000	16917041	100.000

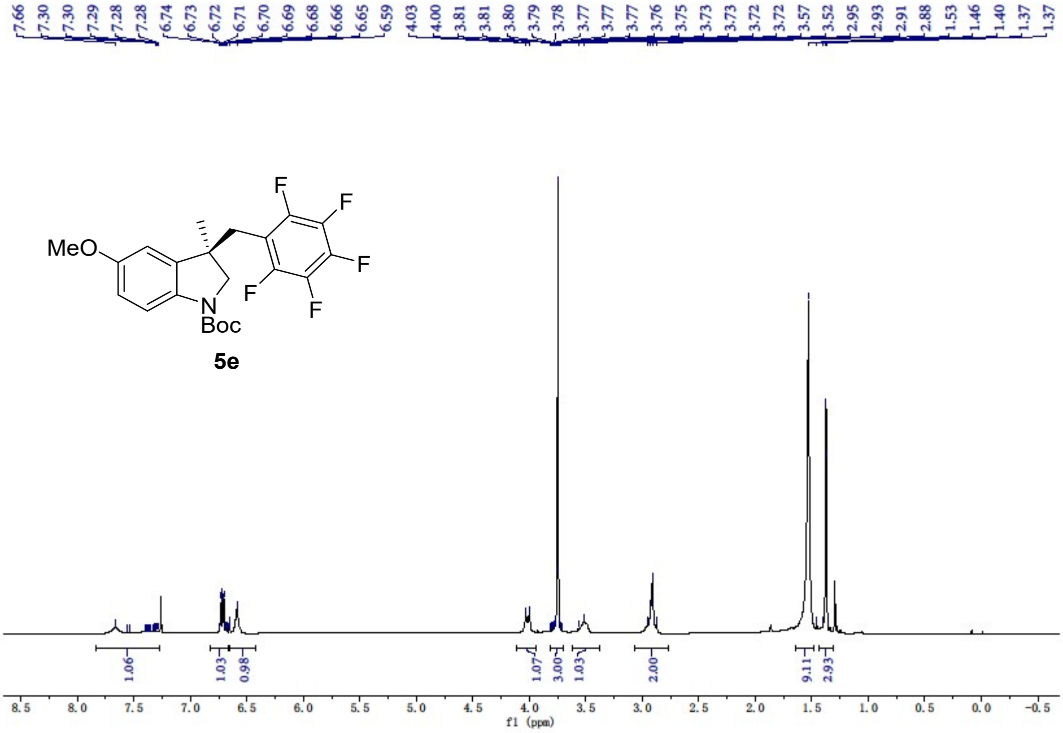

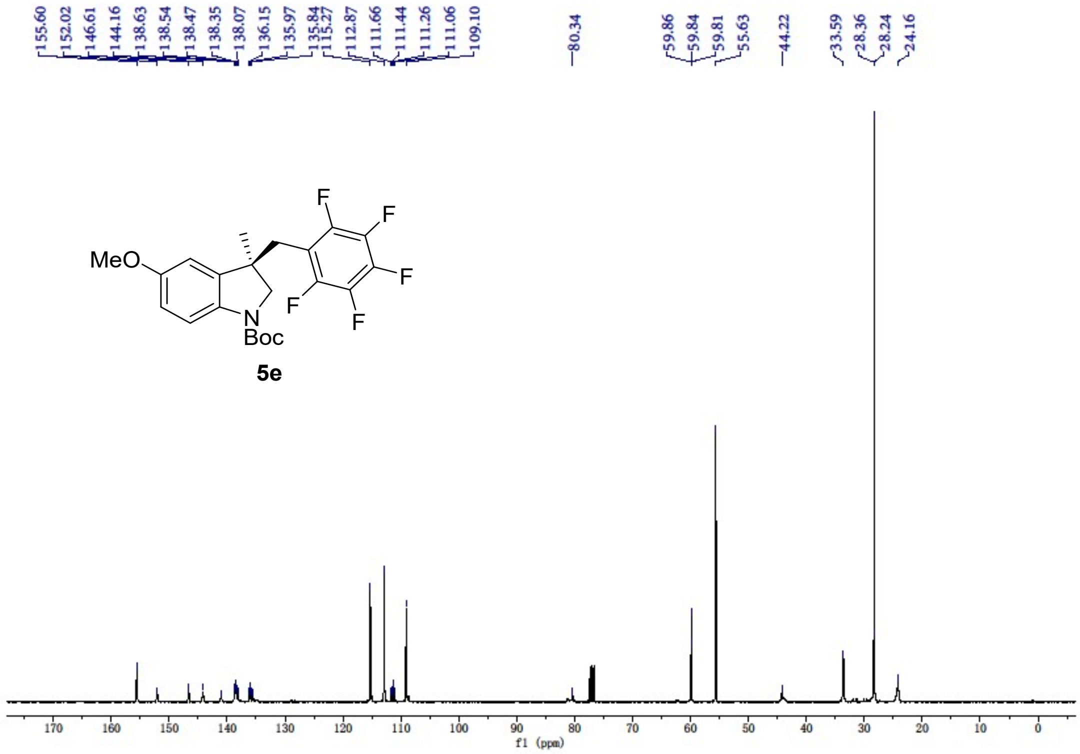
155.60
152.02
146.61
144.16
138.63
138.54
138.47
138.35
138.07
136.15
135.97
135.84
115.27
112.87
111.66
111.44
111.26
111.06
109.10
80.34
59.86
59.84
59.81
55.63
44.22
33.59
28.36
28.24
24.16
MeO
N
Boc
F
F
F
F
F
5e
f1 (ppm)

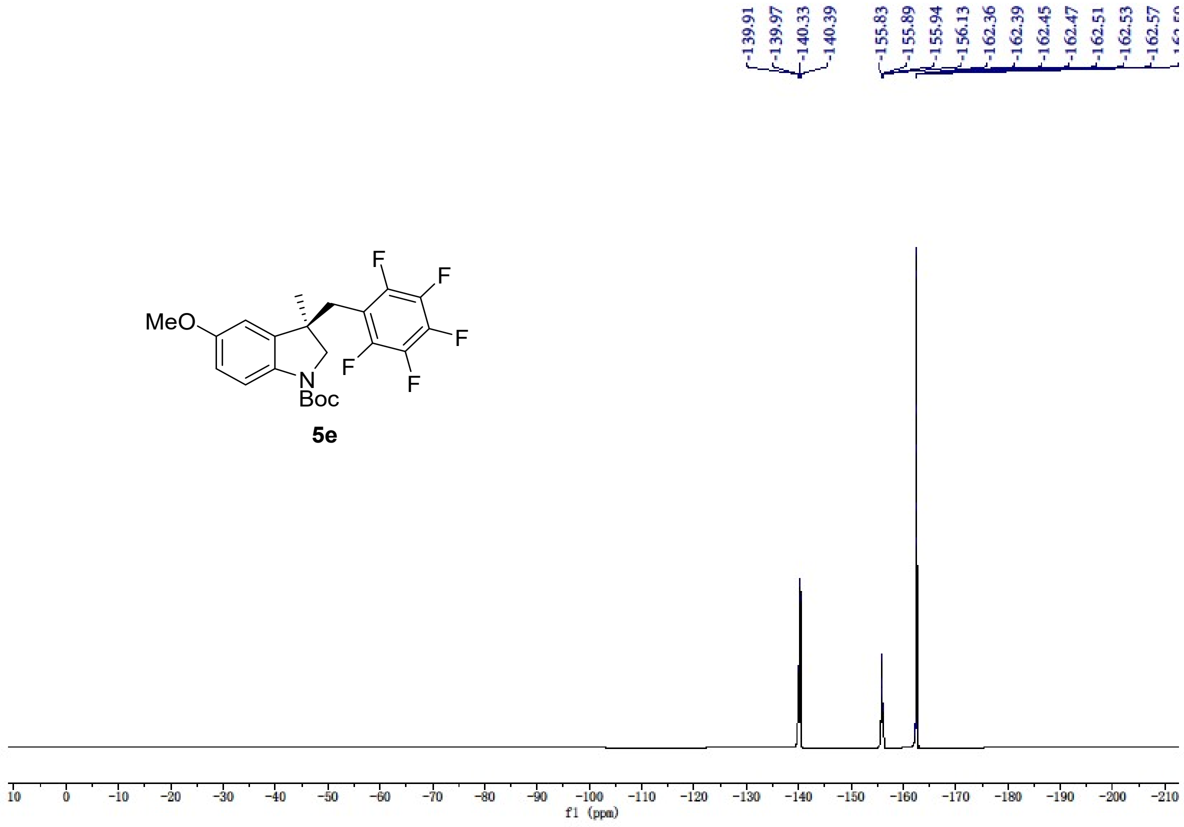
-139.91
-139.97
-140.33
-140.39
-155.83
-155.89
-155.94
-156.13
-162.36
-162.39
-162.45
-162.47
-162.51
-162.53
-162.57
MeO
N
Boc
F
F
F
F
F
5e
f1 (ppm)

<Chromatogram>

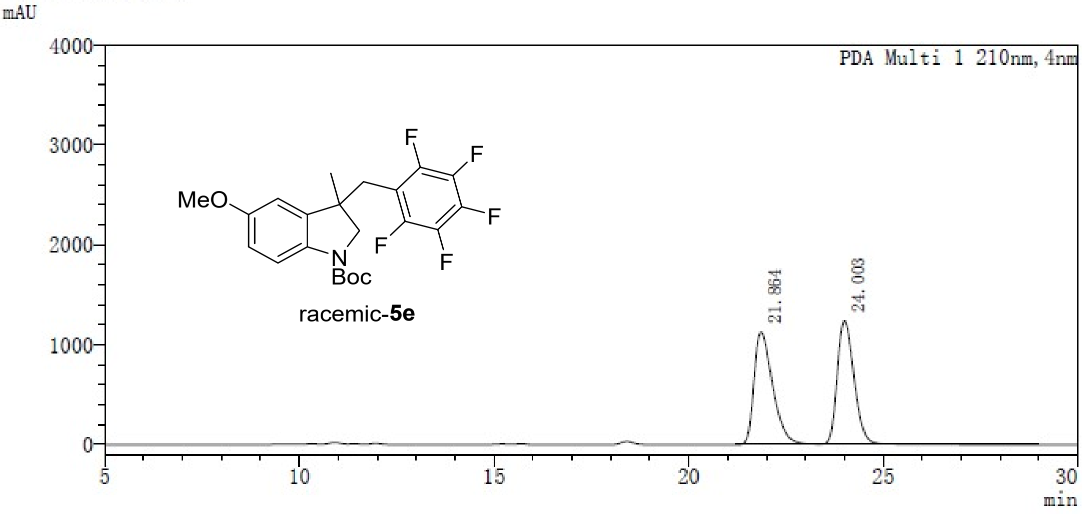

<Peak Table>

PDA Ch1 210nm

No.	Ret. Time (min)	Height	Height%	Area	Area%
1	21.864	1127208	47.620	37897519	49.976
2	24.003	1239897	52.380	37934401	50.024
Total		2367105	100.000	75831920	100.000

<Chromatogram>

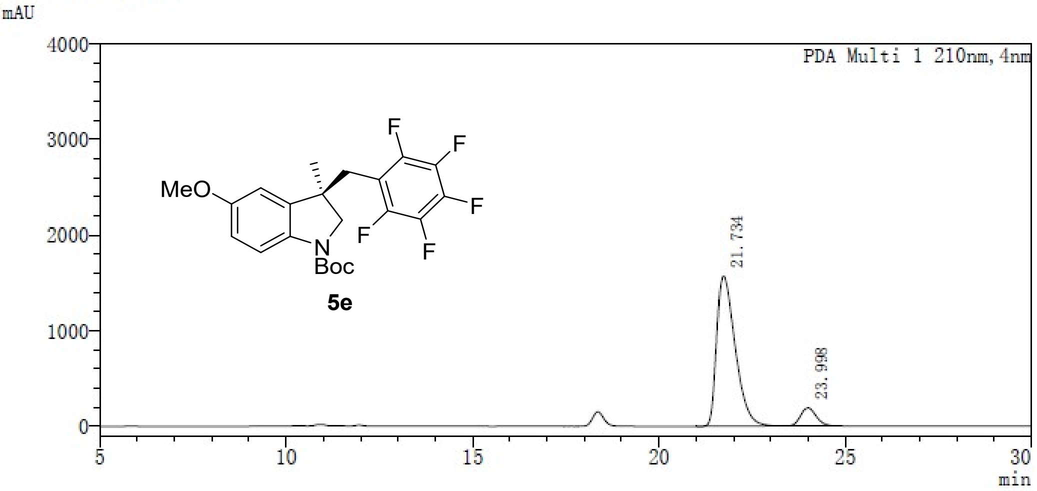

<Peak Table>

PDA Ch1 210nm

No.	Ret. Time (min)	Height	Height%	Area	Area%
1	21.734	1569451	89.264	53829598	90.971
2	23.998	188771	10.736	5342637	9.029
Total		1758222	100.000	59172235	100.000

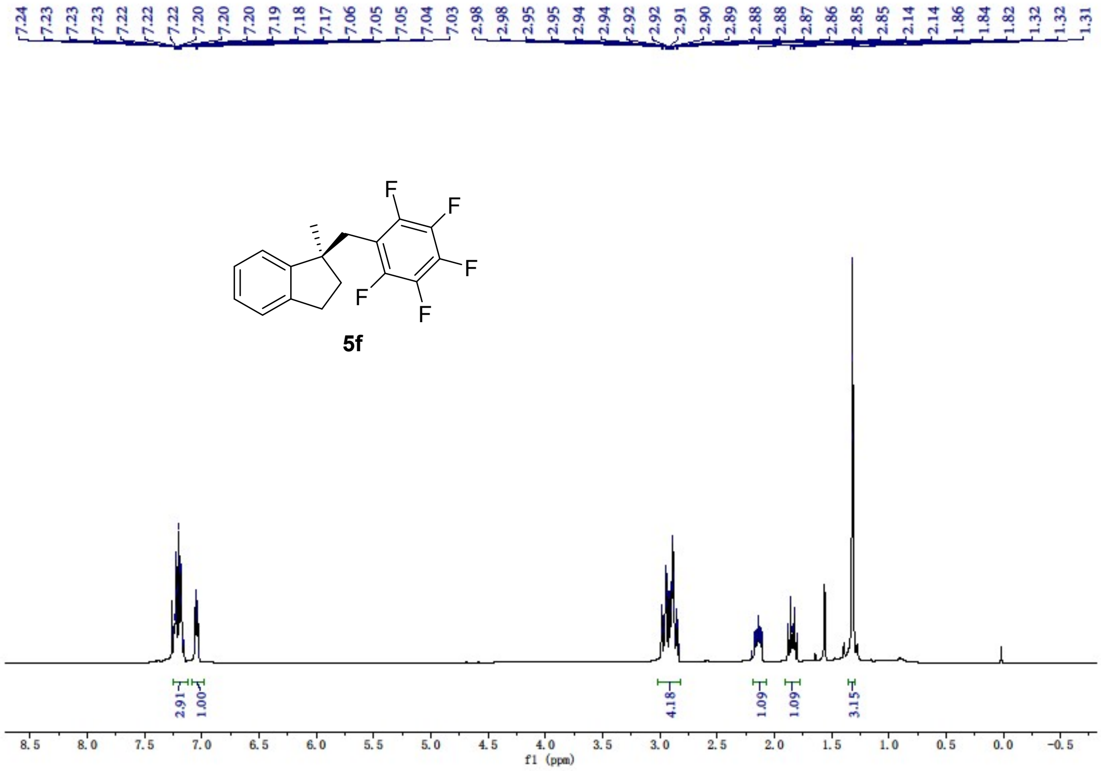
7.24 7.23 7.23 7.23 7.22 7.22 7.22 7.20 7.20 7.20 7.19 7.18 7.17 7.06 7.05 7.05 7.04 7.03 2.98 2.98 2.95 2.95 2.94 2.94 2.92 2.92 2.91 2.90 2.89 2.88 2.88 2.87 2.86 2.85 2.85 2.14 2.14 1.86 1.84 1.82 1.32 1.32 1.31
5f
2.91 1.00 4.18 1.09 1.09 3.15
f1 (ppm)

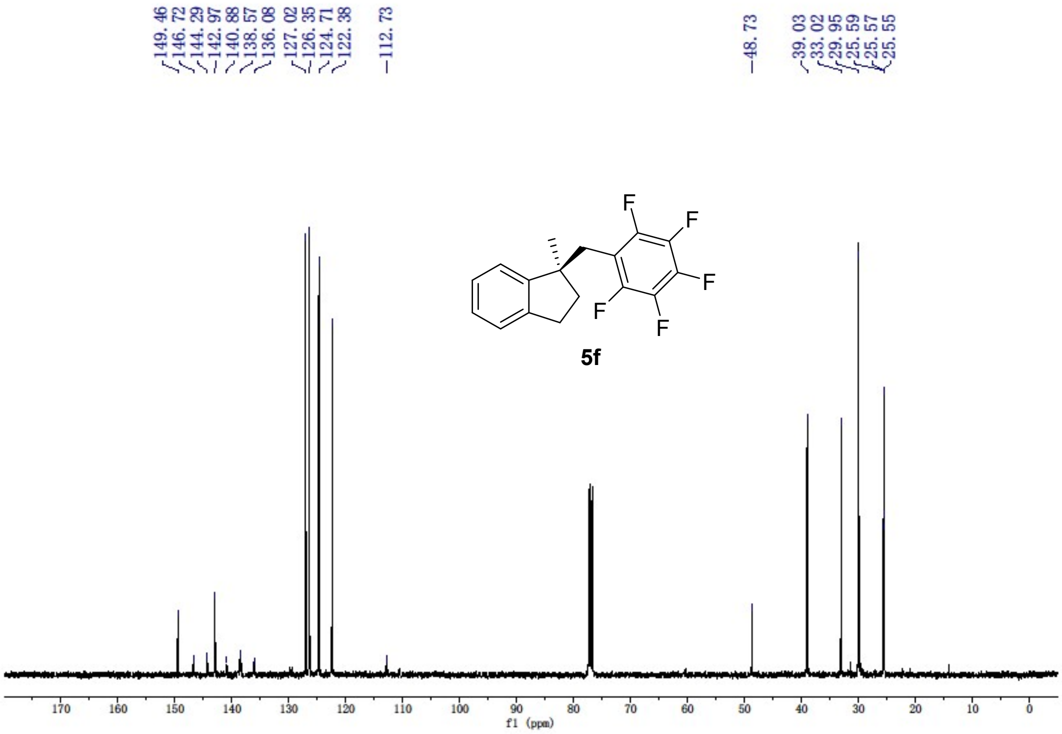
149.46 146.72 144.29 142.97 140.88 138.57 136.08 127.02 126.35 124.71 122.38 112.73 48.73 39.03 33.02 29.95 25.59 25.57 25.55
5f
f1 (ppm)

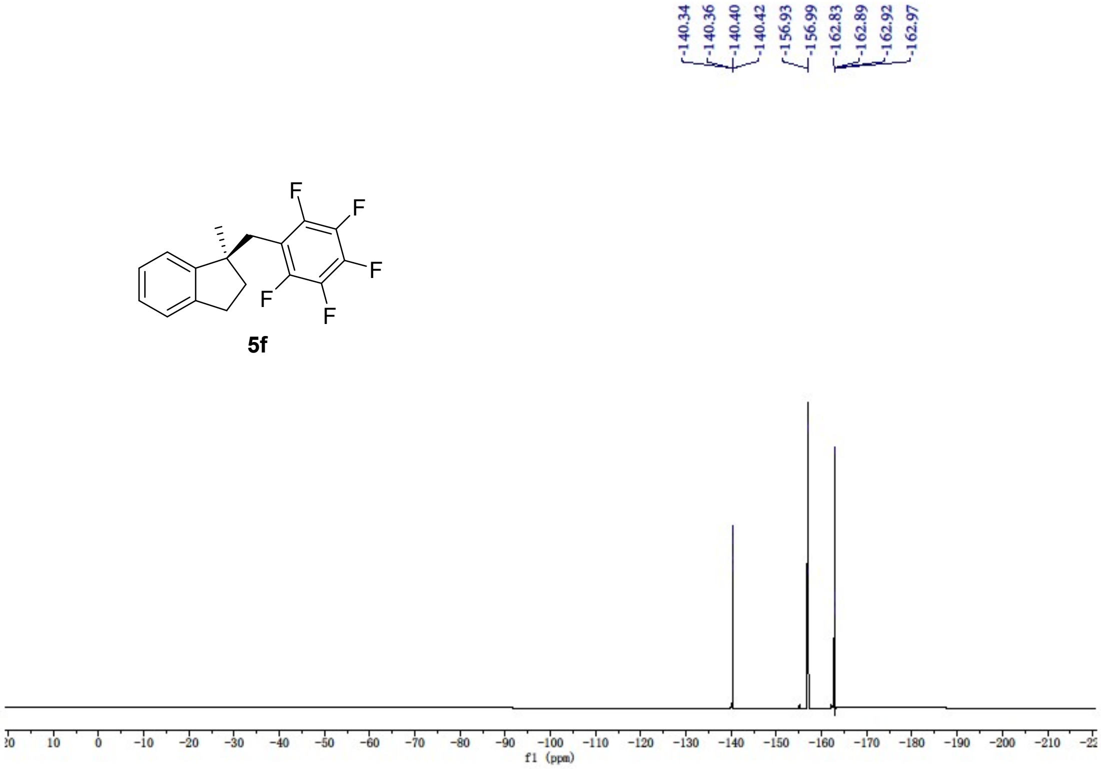

<Chromatogram>

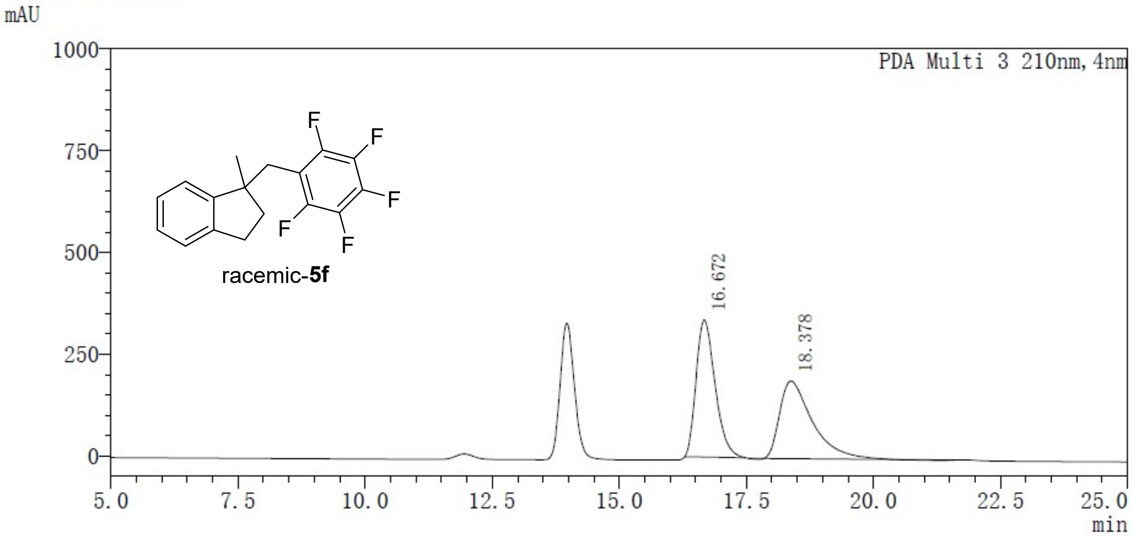

<Peak Table>

PDA Ch3 210nm

No.	Ret.Time(min)	Height	Height%	Area	Area%
1	16.672	336510	63.838	8758341	50.754
2	18.378	190621	36.162	8498183	49.246
Total		527132	100.000	17256524	100.000

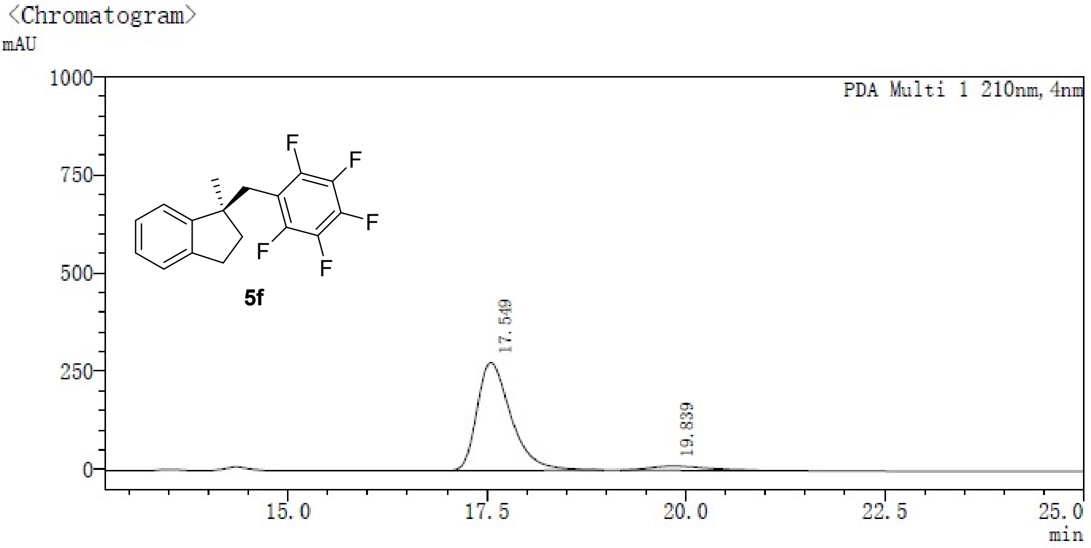

<Peak Table>

PDA Ch1 210nm

No.	Ret. Time(min)	Height	Height%	Area	Area%
1	17.549	274868	96.354	8157942	93.961
2	19.839	10401	3.646	524312	6.039
Total		285270	100.000	8682254	100.000

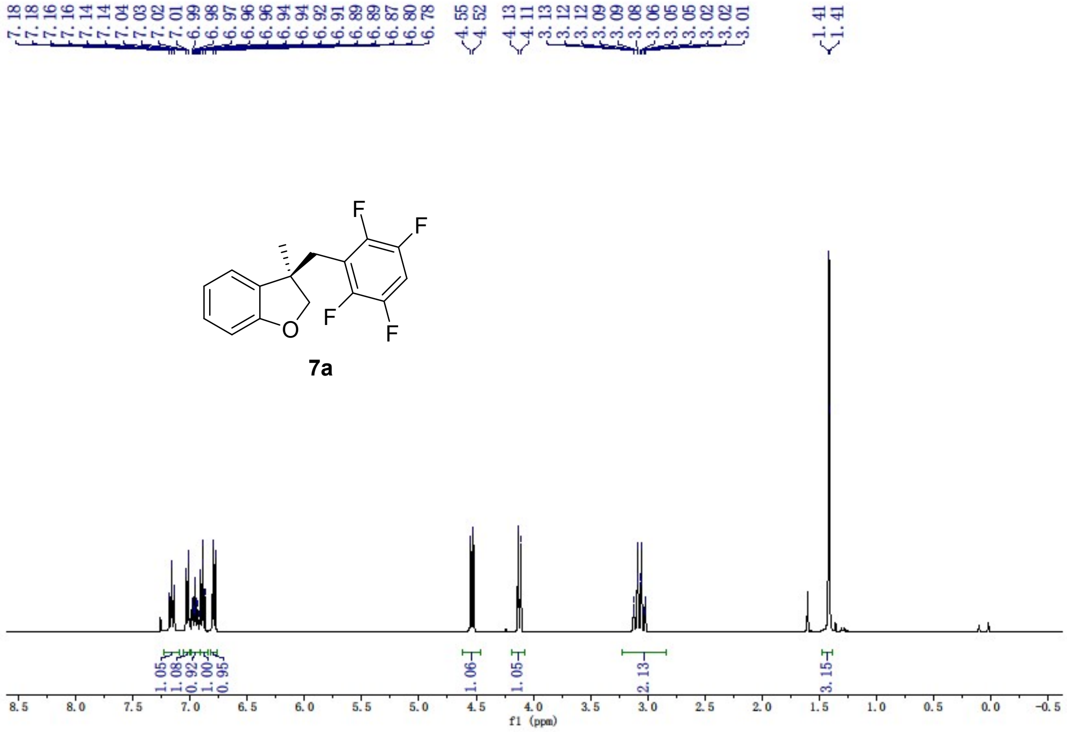

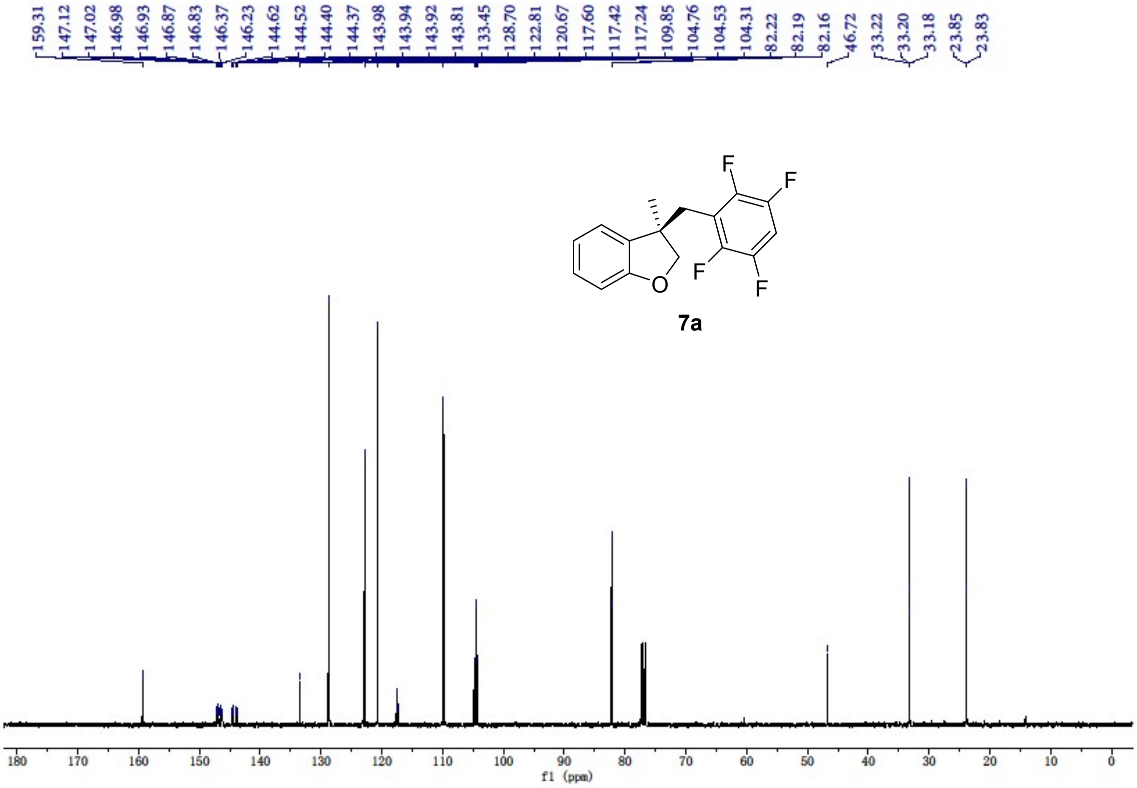
159.31
147.12
147.02
146.98
146.93
146.87
146.83
146.37
146.23
144.62
144.52
144.40
144.37
143.98
143.94
143.92
143.81
133.45
128.70
122.81
120.67
117.60
117.42
117.24
109.85
104.76
104.53
104.31
82.22
82.19
82.16
46.72
33.22
33.20
33.18
23.85
23.83
F
F
F
F
O
7a
180 170 160 150 140 130 120 110 100 90 80 70 60 50 40 30 20 10 0
f1 (ppm)

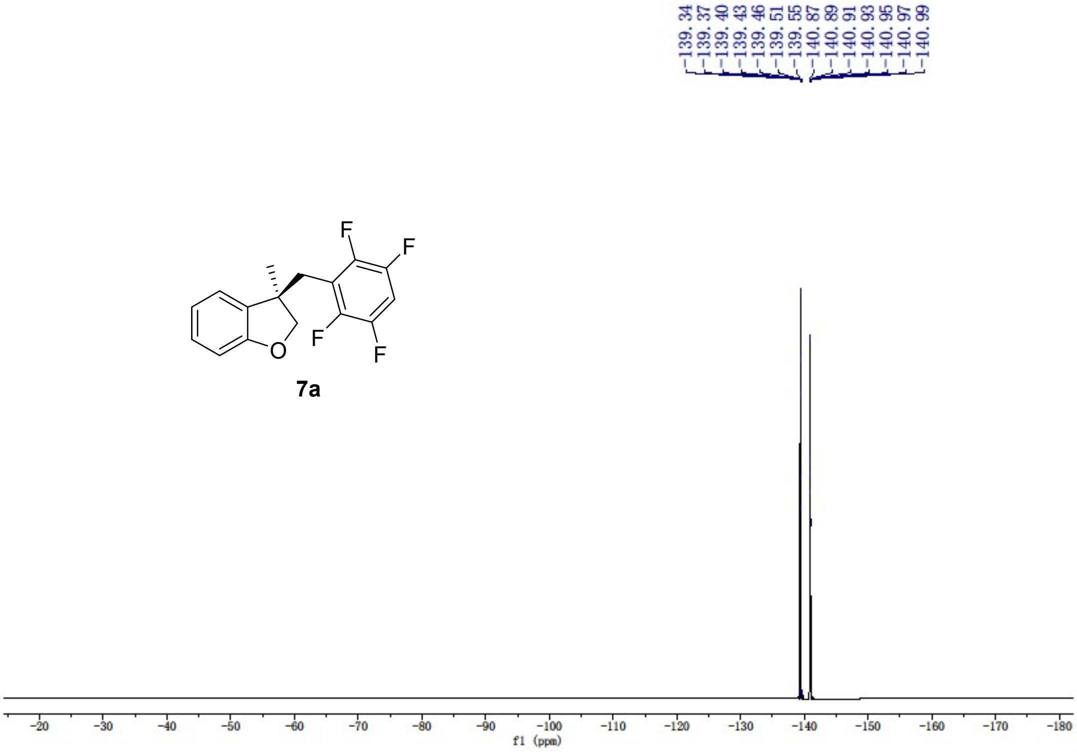
-139.34
-139.37
-139.40
-139.43
-139.46
-139.51
-139.55
-140.87
-140.89
-140.91
-140.93
-140.95
-140.97
-140.99
F
F
F
F
O
7a
-20 -30 -40 -50 -60 -70 -80 -90 -100 -110 -120 -130 -140 -150 -160 -170 -180
f1 (ppm)

<Chromatogram>

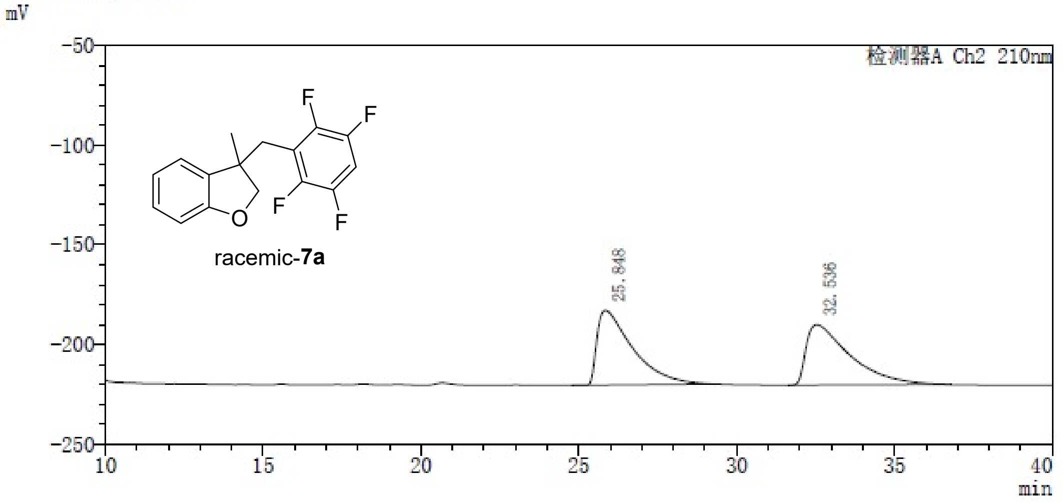

<Peak Table>

检测器A Ch2 210nm

No.	Ret.Time(min)	Height	Height%	Area	Area%
1	25.848	37454	55.260	2949380	50.061
2	32.536	30323	44.740	2942142	49.939
Total		67777	100.000	5891522	100.000

<Chromatogram>

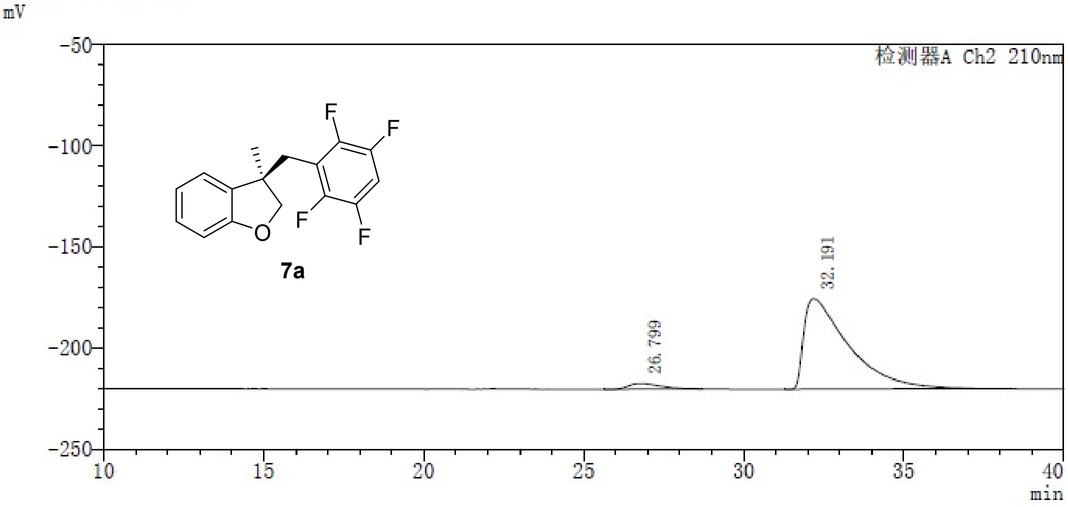

<Peak Table>

检测器A Ch2 210nm

No.	Ret.Time(min)	Height	Height%	Area	Area%
1	26.799	2898	6.073	198478	4.354
2	32.191	44830	93.927	4359740	95.646
Total		47728	100.000	4558218	100.000

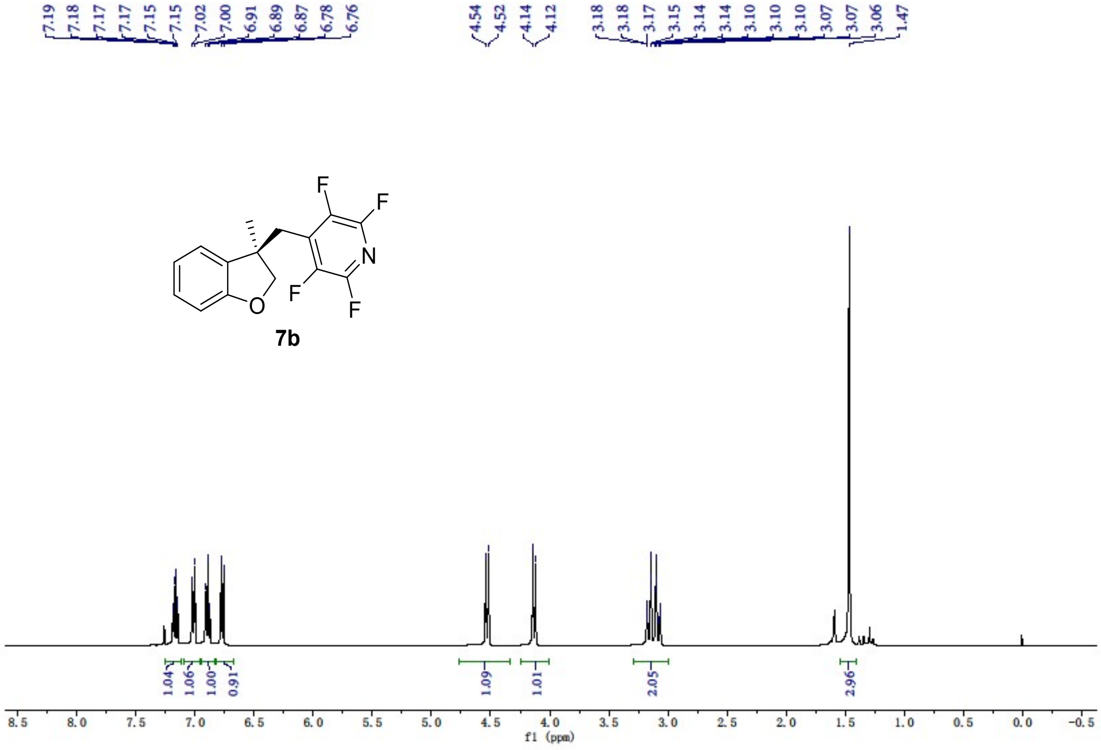
7.19 7.18 7.17 7.17 7.15 7.15 7.02 7.00 6.91 6.89 6.87 6.78 6.76
4.54 4.52 4.14 4.12
3.18 3.18 3.17 3.15 3.14 3.14 3.10 3.10 3.10 3.07 3.07 3.06 1.47
F
F
N
F
F
O
7b
1.04 1.06 1.00 0.91 1.09 1.01 2.05 2.96
f1 (ppm)

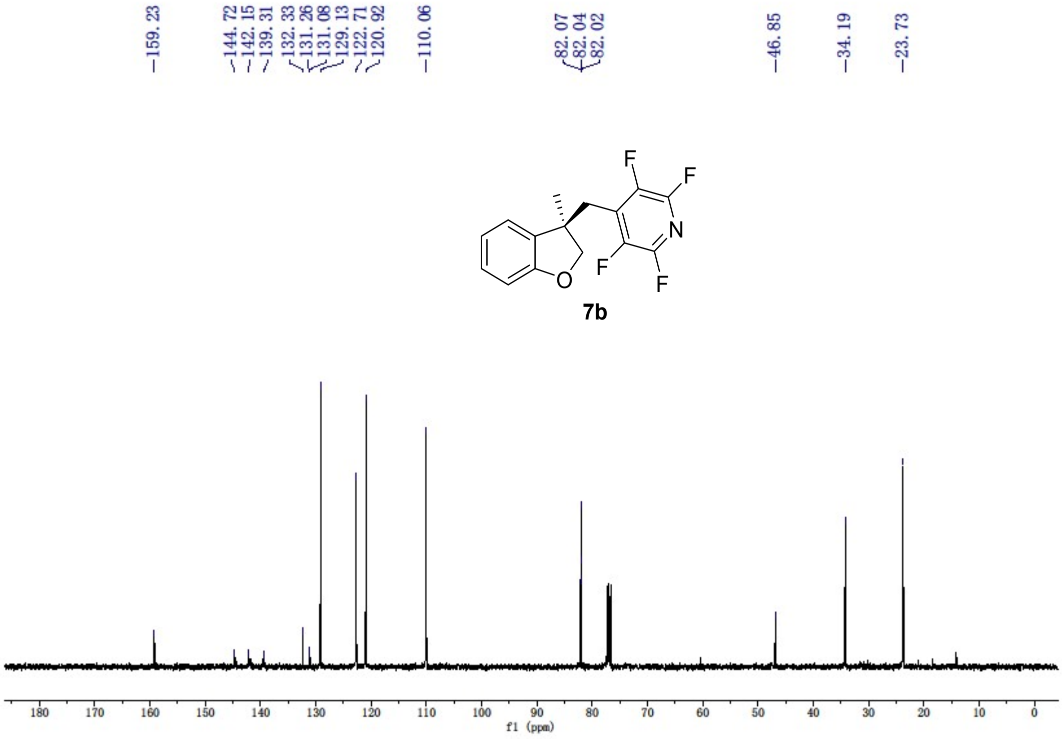
159.23
144.72 142.15 139.31 132.33 131.26 131.08 129.13 122.71 120.92
110.06
82.07 82.04 82.02
46.85
34.19
23.73
F
F
N
F
F
O
7b
f1 (ppm)

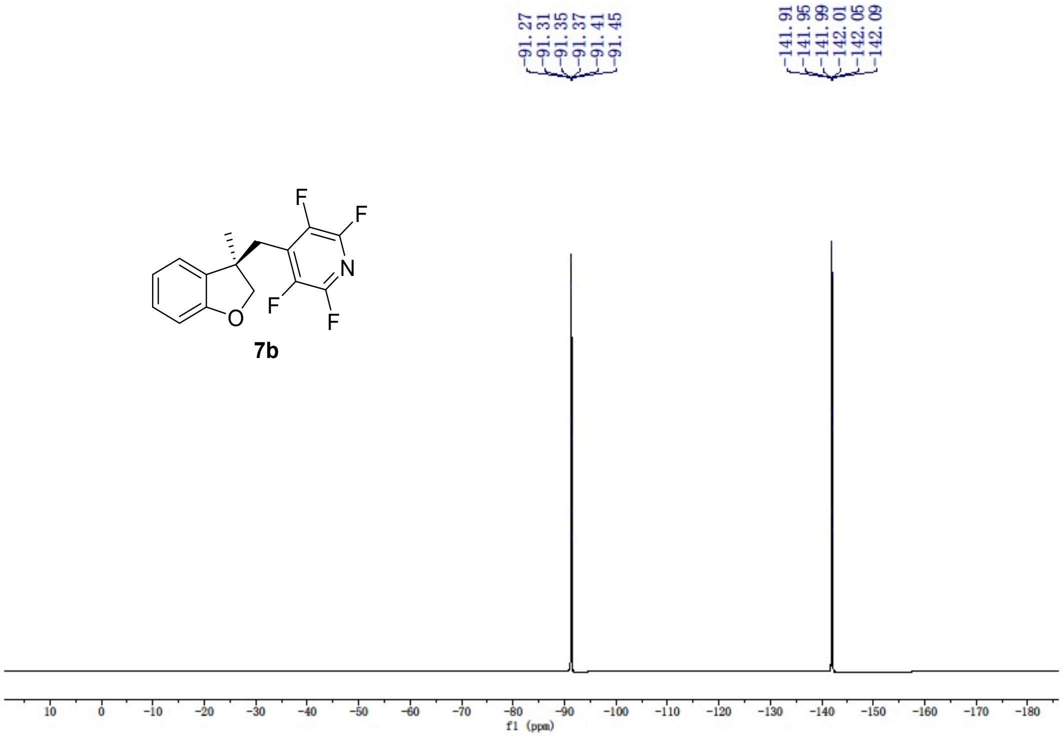

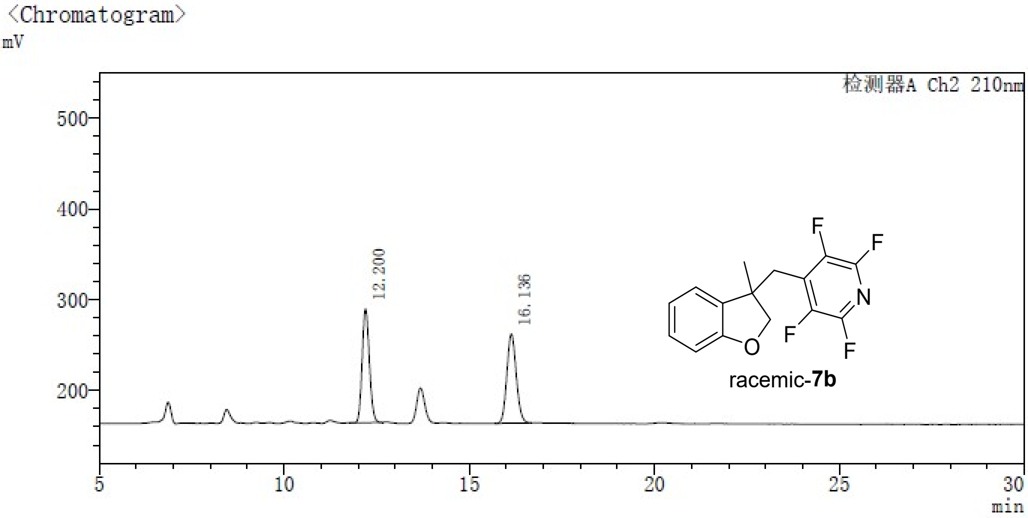

<Peak Table>

检测器A Ch2 210nm

No.	Ret. Time (min)	Height	Height%	Area	Area%
1	12.200	124859	56.076	1692671	49.472
2	16.136	97802	43.924	1728814	50.528
Total		222662	100.000	3421484	100.000

<Chromatogram>

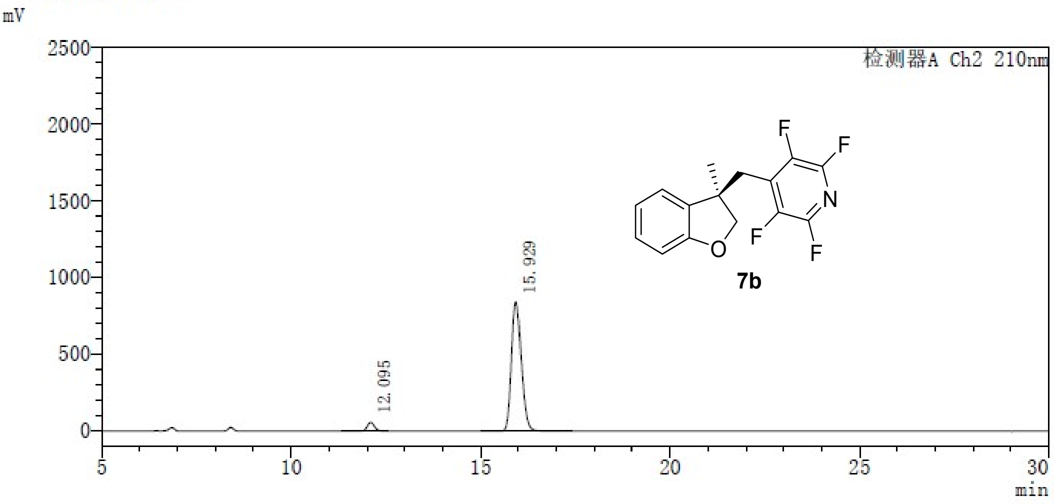

<Peak Table>

检测器A Ch2 210nm

No.	Ret. Time (min)	Height	Height%	Area	Area%
1	12.095	58242	6.456	801235	4.972
2	15.929	843853	93.544	15313729	95.028
Total		902095	100.000	16114965	100.000

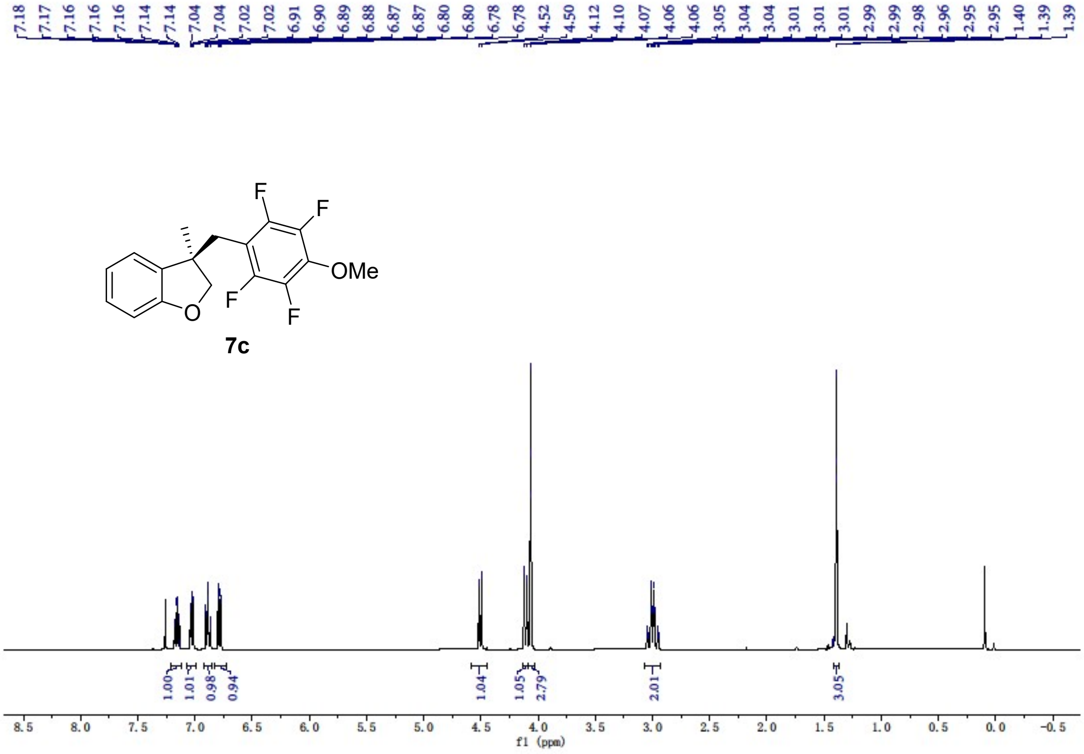

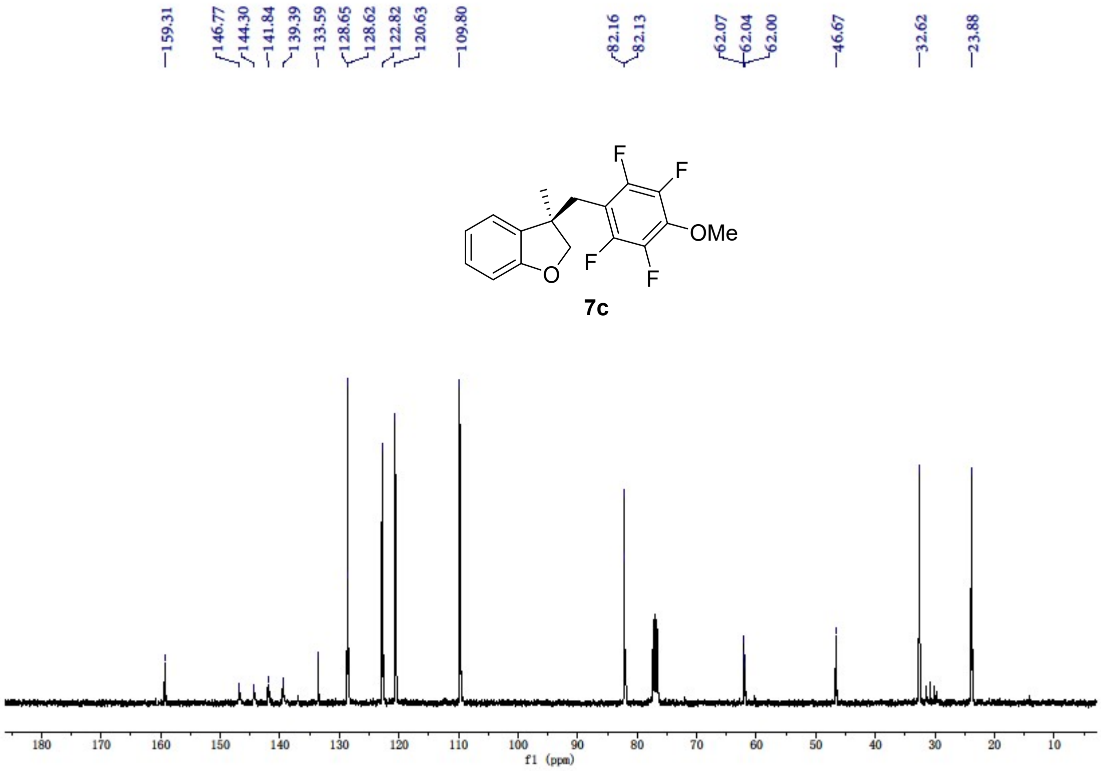
159.31
146.77
144.30
141.84
139.39
133.59
128.65
128.62
122.82
120.63
109.80
82.16
82.13
62.07
62.04
62.00
46.67
32.62
23.88
F
F
OMe
F
F
O
7c
f1 (ppm)

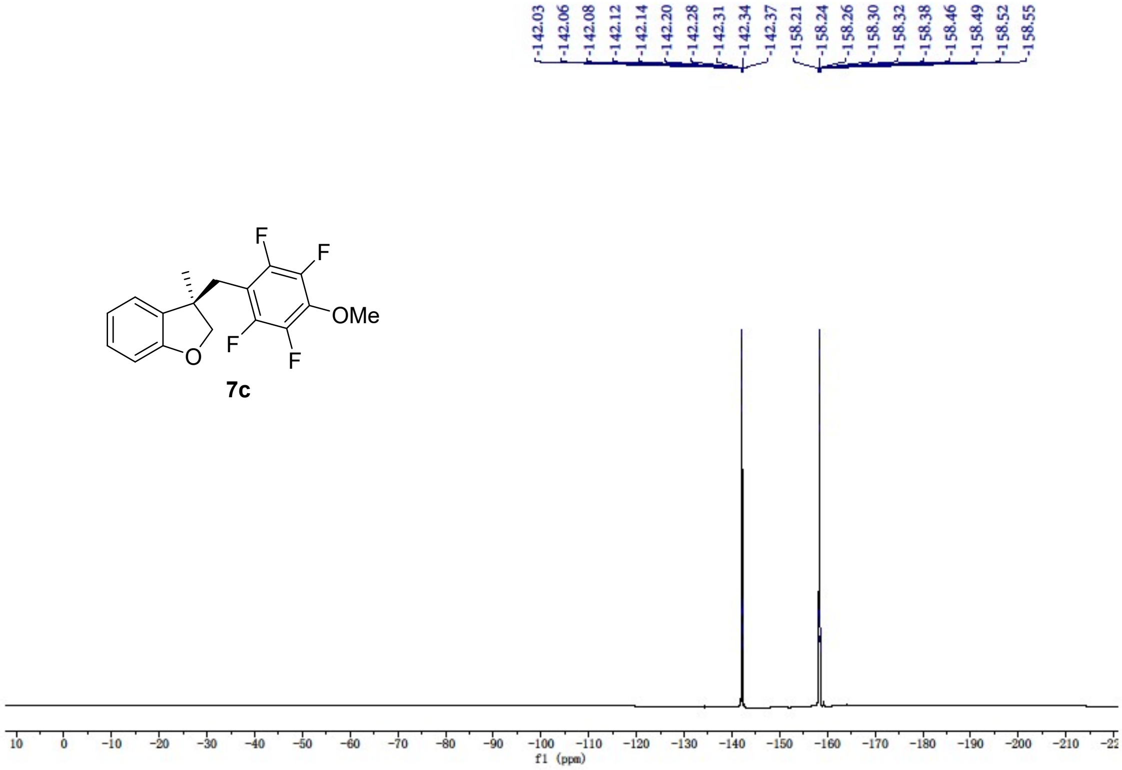
-142.03
-142.06
-142.08
-142.12
-142.14
-142.20
-142.28
-142.31
-142.34
-142.37
-158.21
-158.24
-158.26
-158.30
-158.32
-158.38
-158.46
-158.49
-158.52
-158.55
F
F
OMe
F
F
O
7c
f1 (ppm)

<Chromatogram>

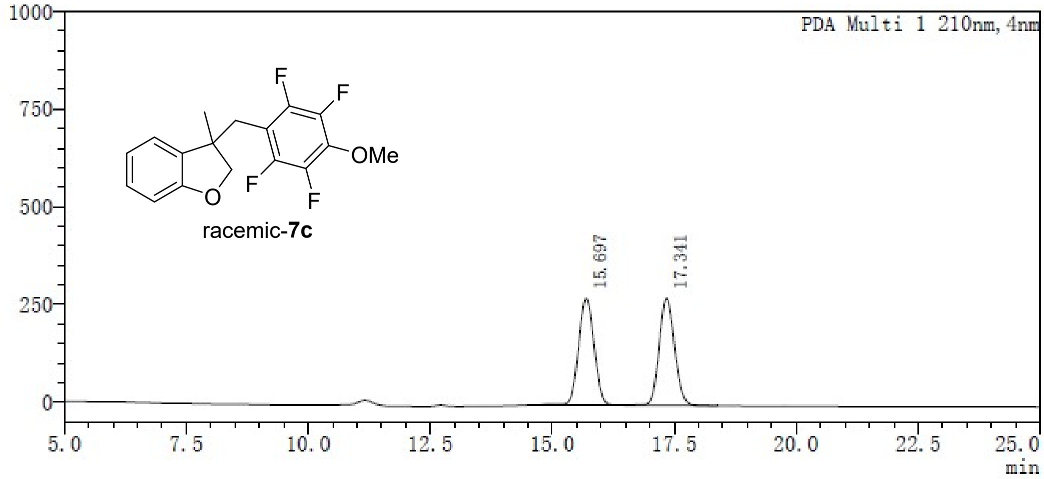

<Peak Table>

PDA Ch1 210nm

No.	Ret. Time(min)	Height	Height%	Area	Area%
1	15.697	273395	49.884	6067721	50.125
2	17.341	274668	50.116	6037466	49.875
Total		548063	100.000	12105188	100.000

<Chromatogram>

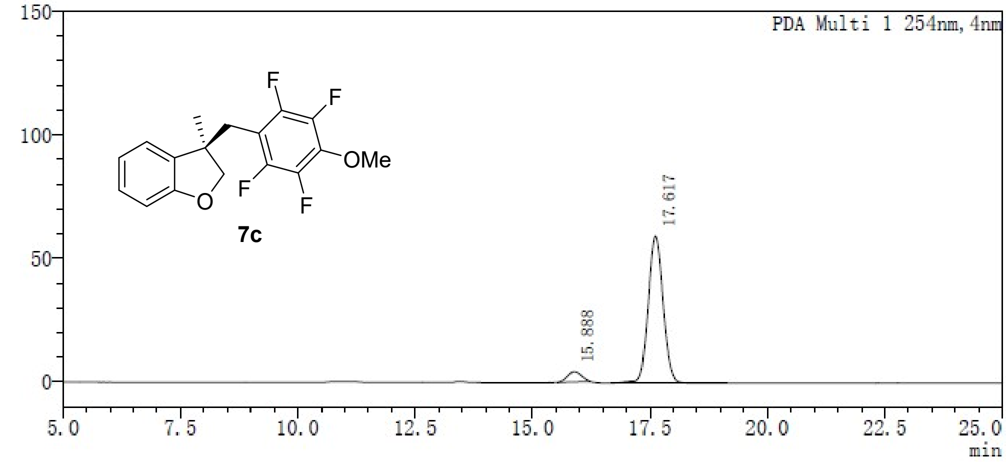

<Peak Table>

PDA Ch1 254nm

No.	Ret. Time(min)	Height	Height%	Area	Area%
1	15.888	4120	6.474	83952	5.980
2	17.617	59519	93.526	1319909	94.020
Total		63639	100.000	1403862	100.000

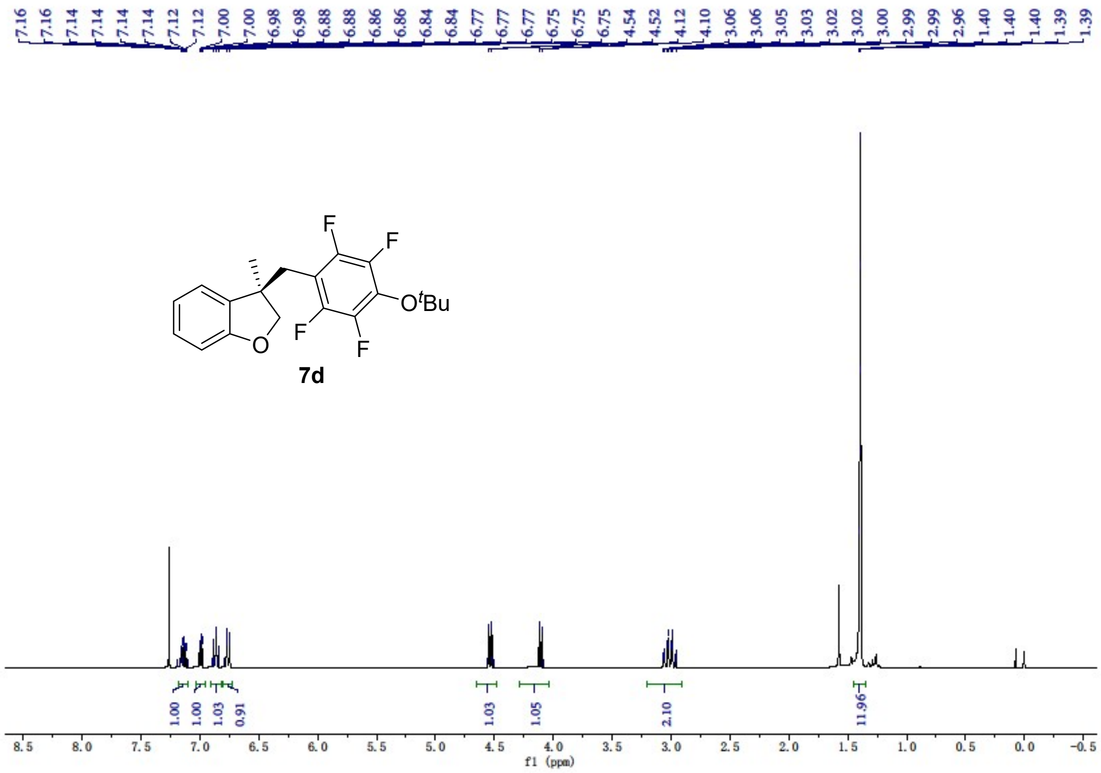
7.16 7.16 7.14 7.14 7.14 7.12 7.12 7.00 7.00 6.98 6.98 6.88 6.88 6.86 6.86 6.84 6.84 6.77 6.77 6.77 6.75 6.75 6.75 4.54 4.52 4.12 4.10 3.06 3.06 3.05 3.03 3.02 3.02 3.00 2.99 2.99 2.96 1.40 1.40 1.40 1.39 1.39
F
F
O^tBu
F
F
O
7d
1.00 1.00 1.03 0.91 1.03 1.05 2.10 11.96
8.5 8.0 7.5 7.0 6.5 6.0 5.5 5.0 4.5 4.0 3.5 3.0 2.5 2.0 1.5 1.0 0.5 0.0 -0.5
f1 (ppm)

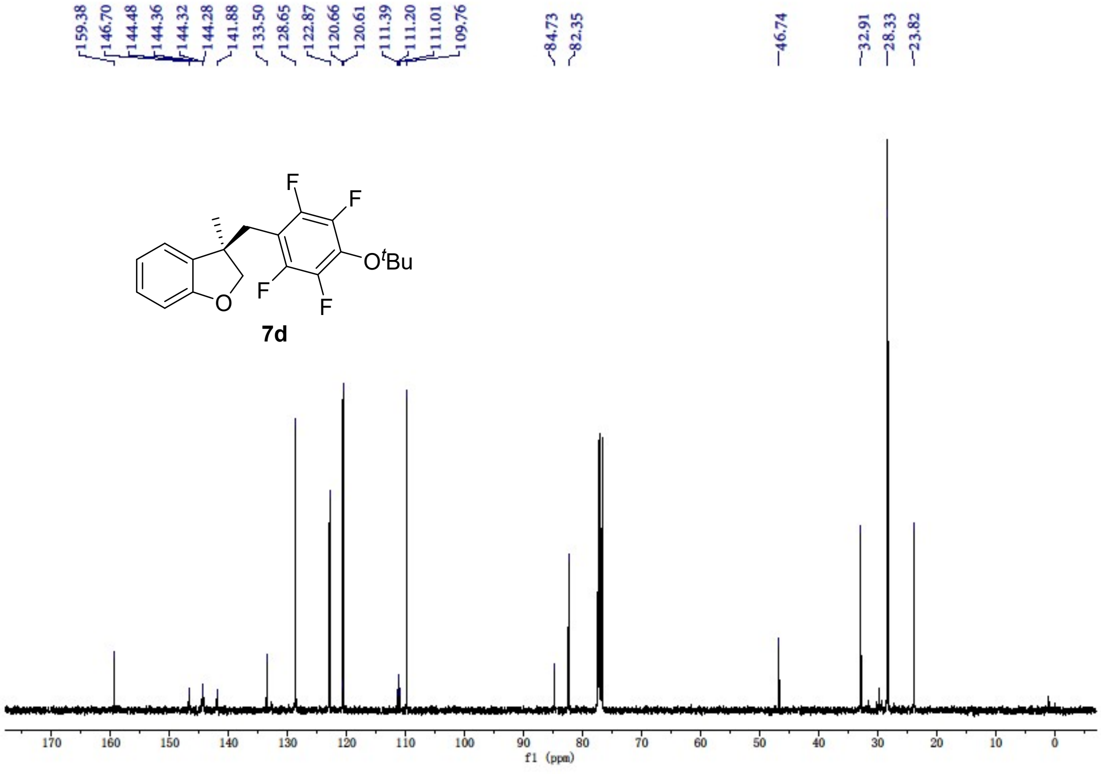
159.38 146.70 144.48 144.36 144.32 144.28 141.88 133.50 128.65 122.87 120.66 120.61 111.39 111.20 111.01 109.76
84.73 82.35
46.74
32.91 28.33 23.82
F
F
O^tBu
F
F
O
7d
170 160 150 140 130 120 110 100 90 80 70 60 50 40 30 20 10 0
f1 (ppm)

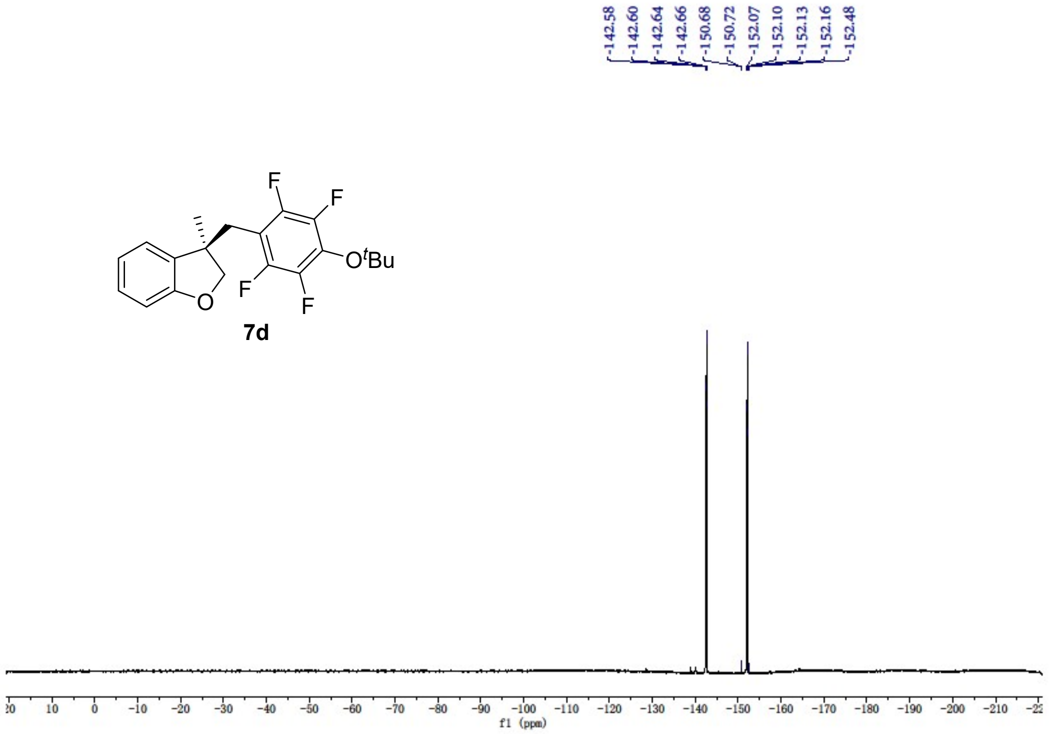

<Chromatogram>

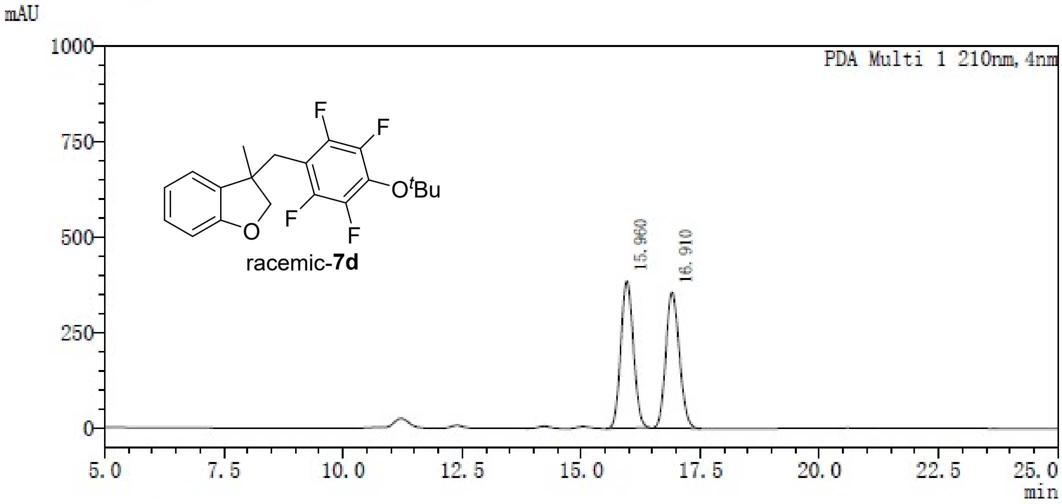

<Peak Table>

PDA Ch1 210nm

No.	Ret. Time (min)	Height	Height%	Area	Area%
1	15.960	386409	52.001	7090008	49.992
2	16.910	356677	47.999	7092228	50.008
Total		743086	100.000	14182236	100.000

<Chromatogram>

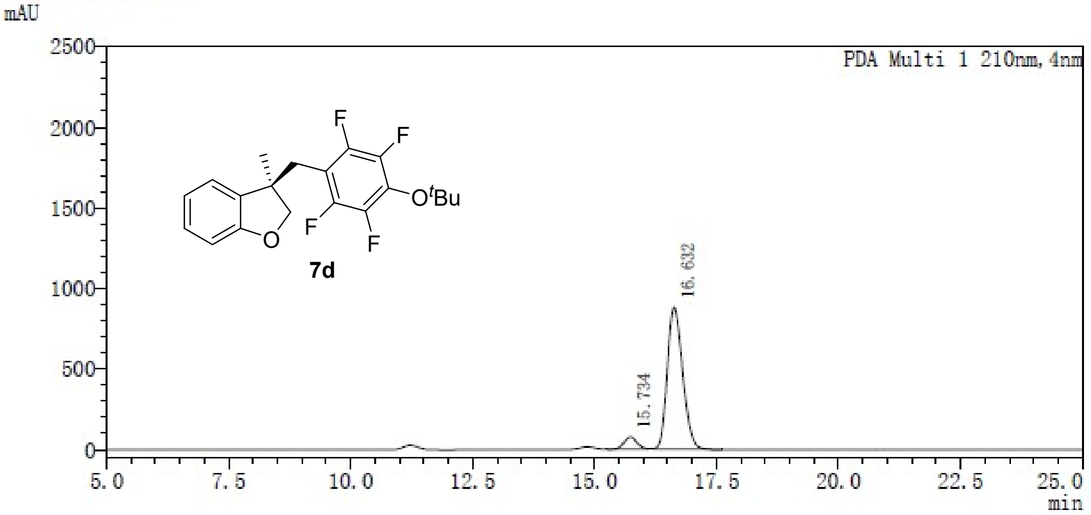

<Peak Table>

PDA Ch1 210nm

No.	Ret. Time(min)	Height	Height%	Area	Area%
1	15.734	75638	7.919	1364831	6.627
Total	16.632	879472	92.081	19231212	93.373
总计		955109	100.000	20596042	100.000

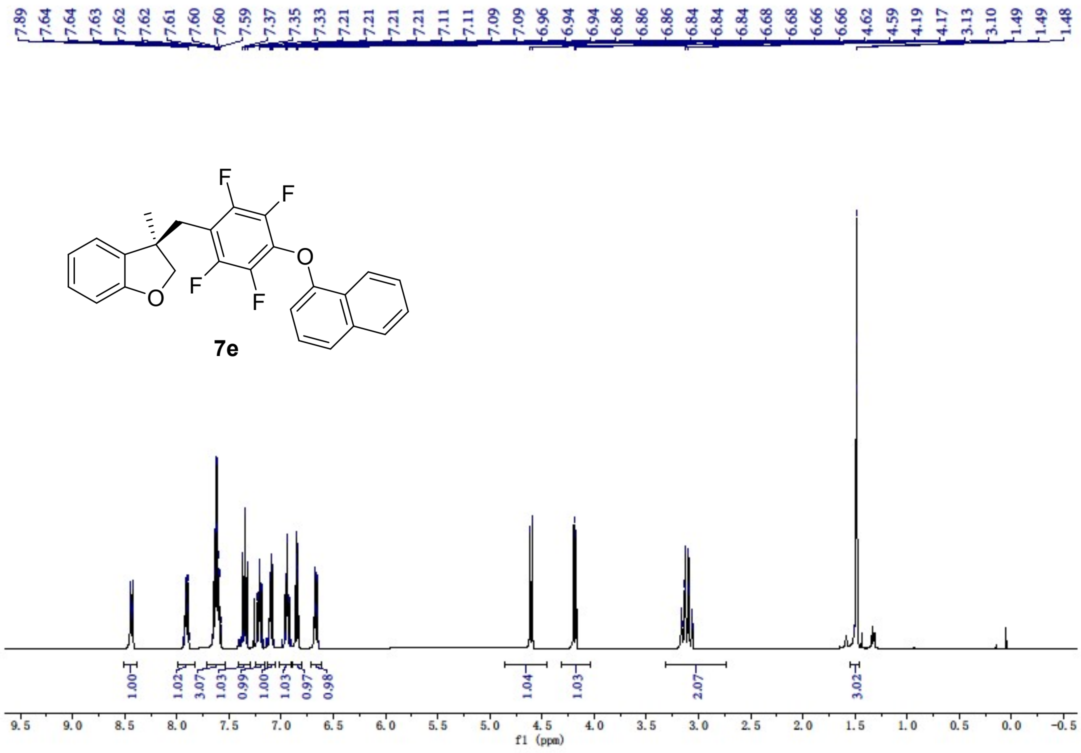

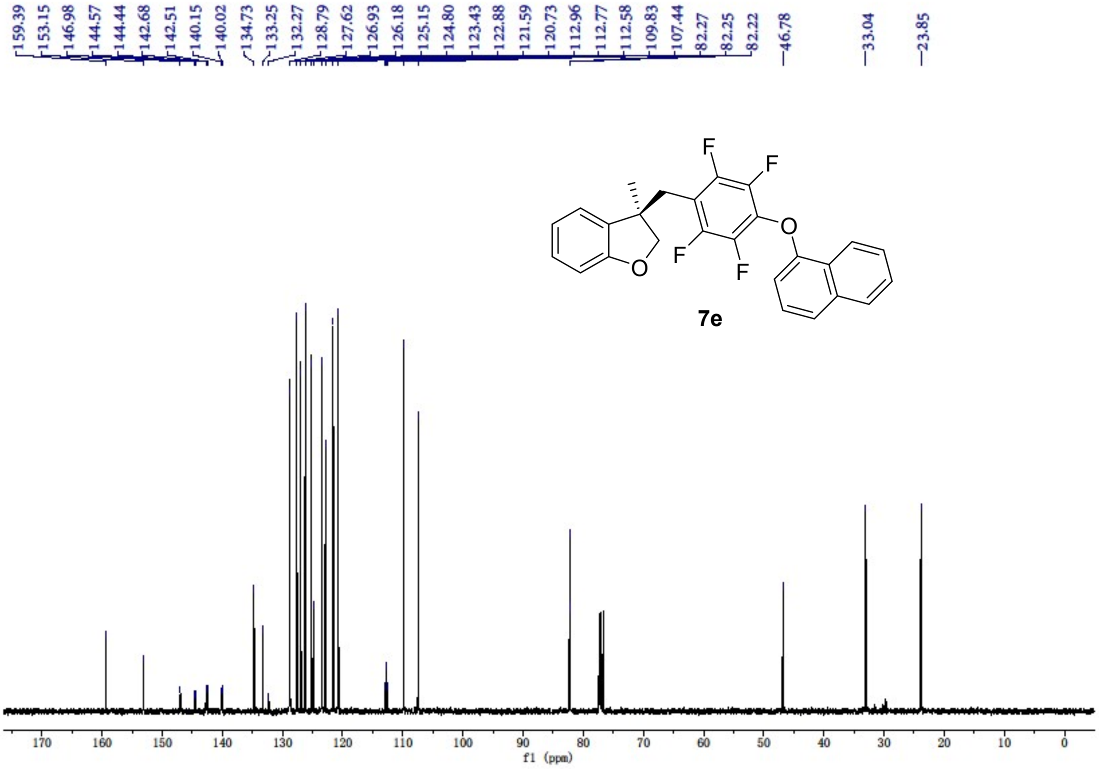
159.39
153.15
146.98
144.57
144.44
142.68
142.51
140.15
140.02
134.73
133.25
132.27
128.79
127.62
126.93
126.18
125.15
124.80
123.43
122.88
121.59
120.73
112.96
112.77
112.58
109.83
107.44
82.27
82.25
82.22
46.78
33.04
23.85
F
F
O
O
F
F
7e
f1 (ppm)

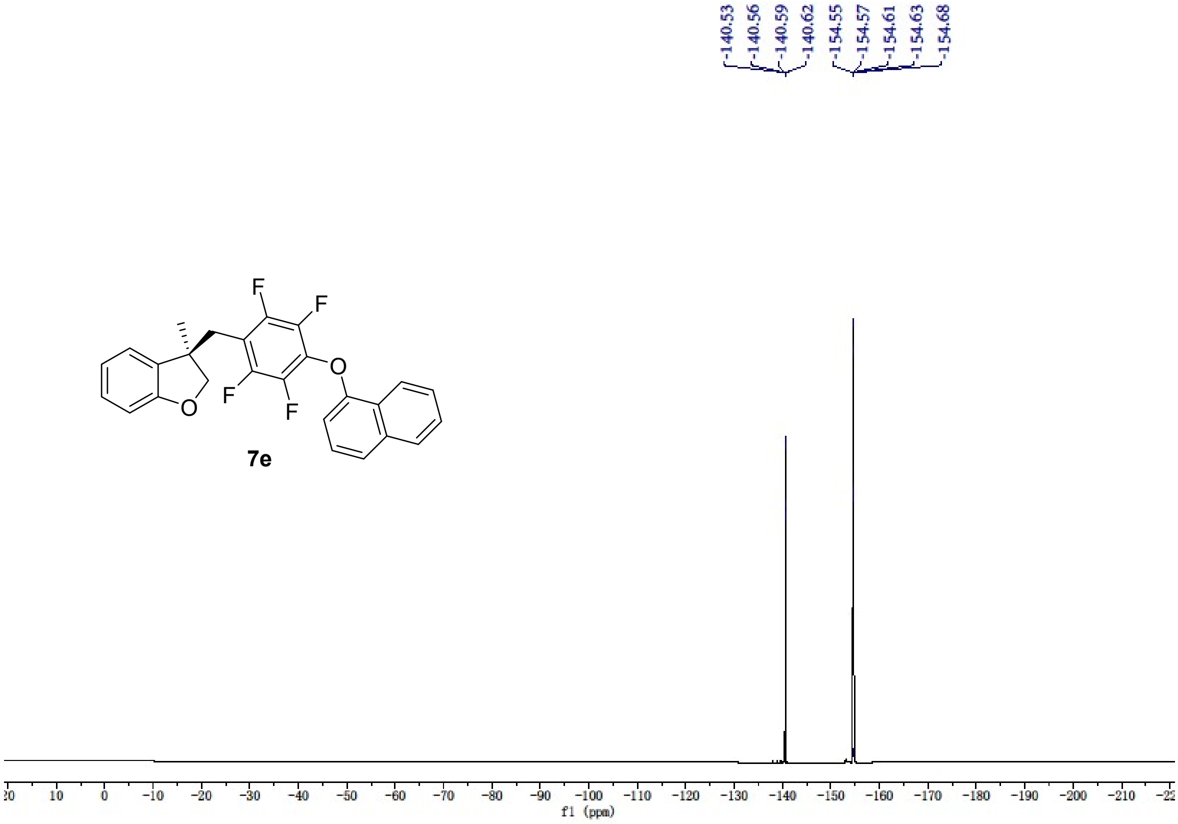
-140.53
-140.56
-140.59
-140.62
-154.55
-154.57
-154.61
-154.63
-154.68
F
F
O
O
F
F
7e
f1 (ppm)

〈Chromatogram〉

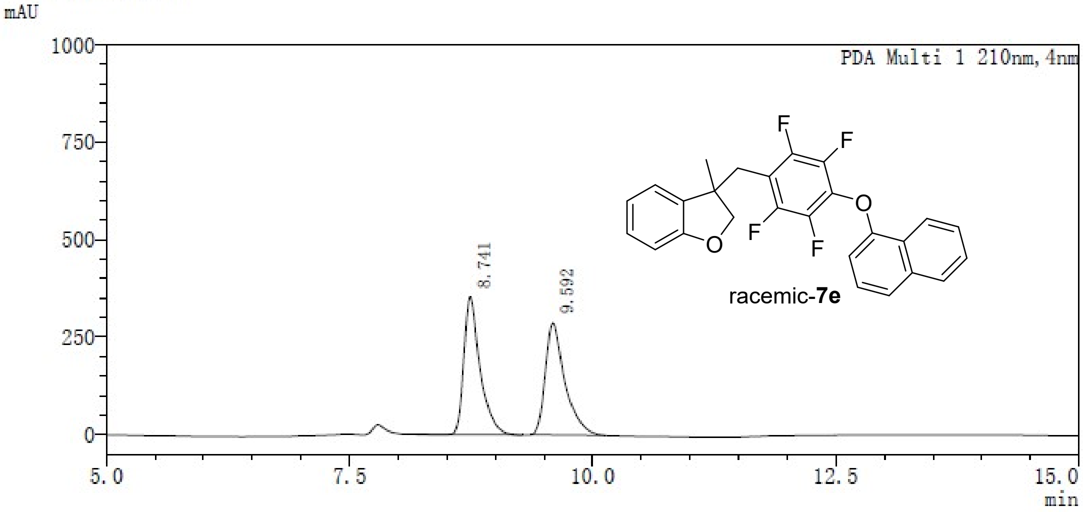

〈Peak Table〉

PDA Ch1 210nm

No.	Ret. Time (min)	Height	Height%	Area	Area%
1	8.741	352827	55.185	4082899	50.385
2	9.592	286528	44.815	4020496	49.615
Total		639355	100.000	8103395	100.000

〈Chromatogram〉

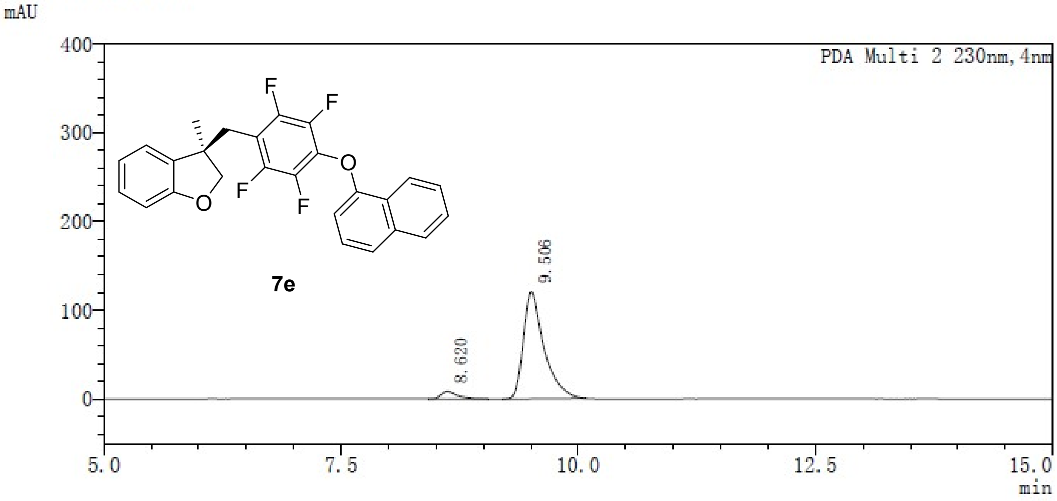

〈Peak Table〉

PDA Ch2 230nm

No.	Ret. Time (min)	Height	Height%	Area	Area%
1	8.620	8343	6.472	98781	5.114
2	9.506	120565	93.528	1832677	94.886
Total		128908	100.000	1931458	100.000

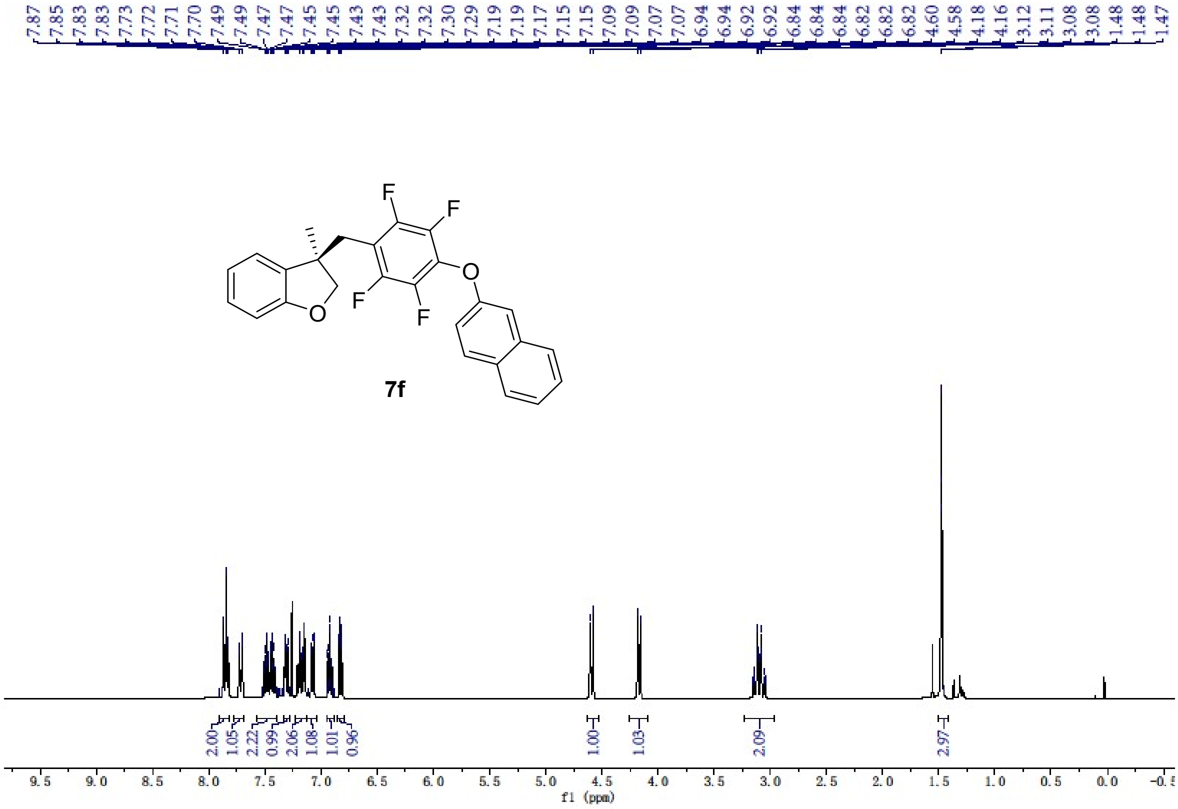
7.87 7.85 7.83 7.83 7.73 7.72 7.71 7.70 7.49 7.49 7.47 7.47 7.45 7.45 7.43 7.43 7.32 7.30 7.29 7.19 7.19 7.17 7.15 7.15 7.09 7.09 7.07 7.07 6.94 6.94 6.92 6.92 6.84 6.84 6.84 6.82 6.82 6.82 4.60 4.58 4.18 4.16 3.12 3.11 3.08 3.08 1.48 1.48 1.47
F
F
O
F
F
O
7f
2.00 1.05 2.22 0.99 2.06 1.08 1.01 0.96 1.00 1.03 2.09 2.97
f1 (ppm)

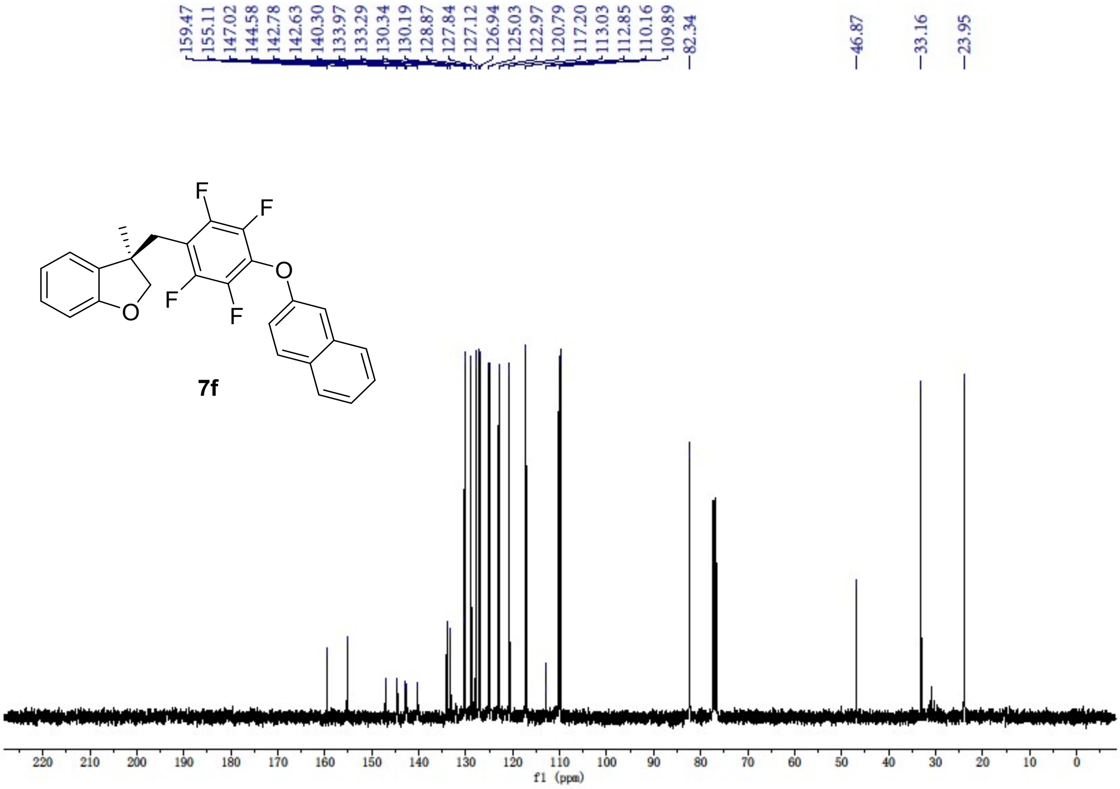
159.47 155.11 147.02 144.58 142.78 142.63 140.30 133.97 133.29 130.34 130.19 128.87 127.84 127.12 126.94 125.03 122.97 120.79 117.20 113.03 112.85 110.16 109.89 82.34
46.87
33.16
23.95
F
F
O
F
F
O
7f
f1 (ppm)

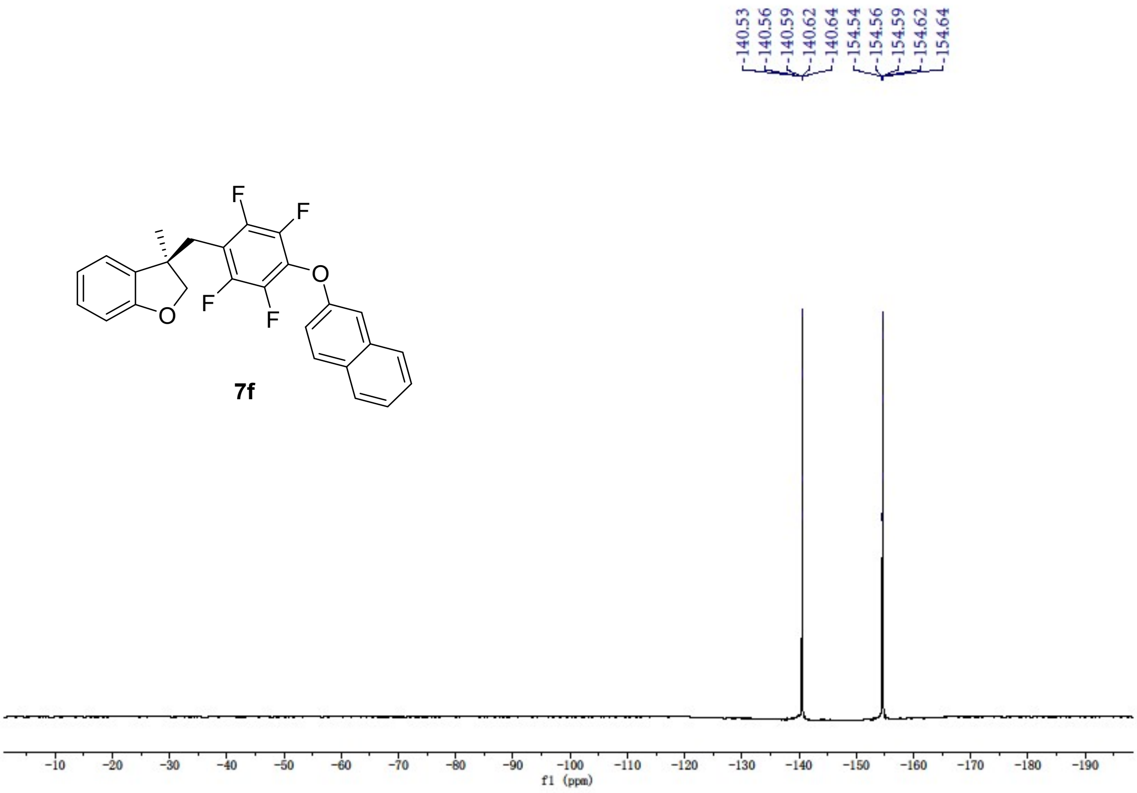

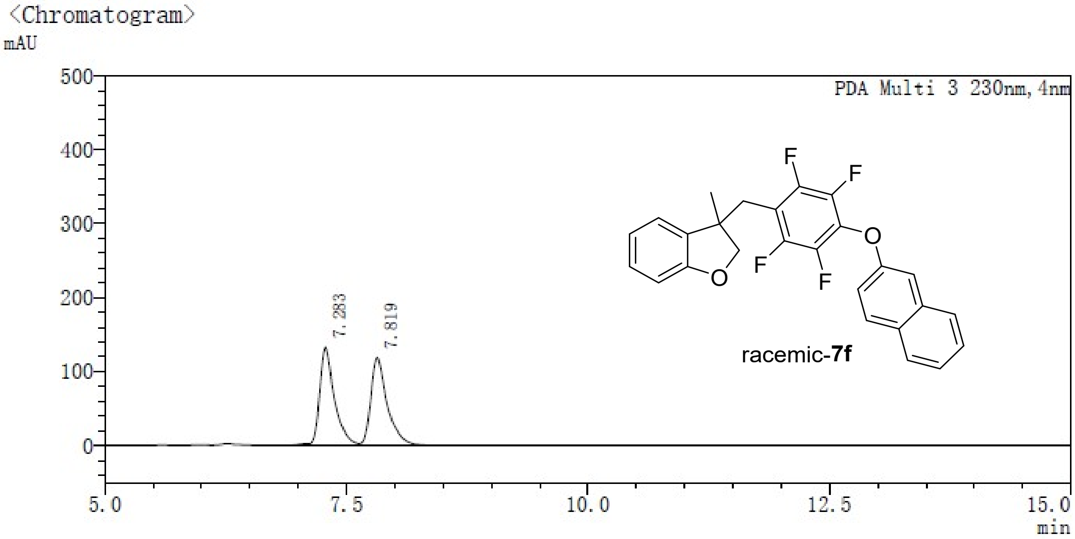

<Peak Table>

PDA Ch3 230nm

No.	Ret. Time (min)	Height	Height%	Area	Area%
1	7.283	132265	52.722	1410528	50.166
2	7.819	118609	47.278	1401178	49.834
Total		250874	100.000	2811706	100.000

<Chromatogram>

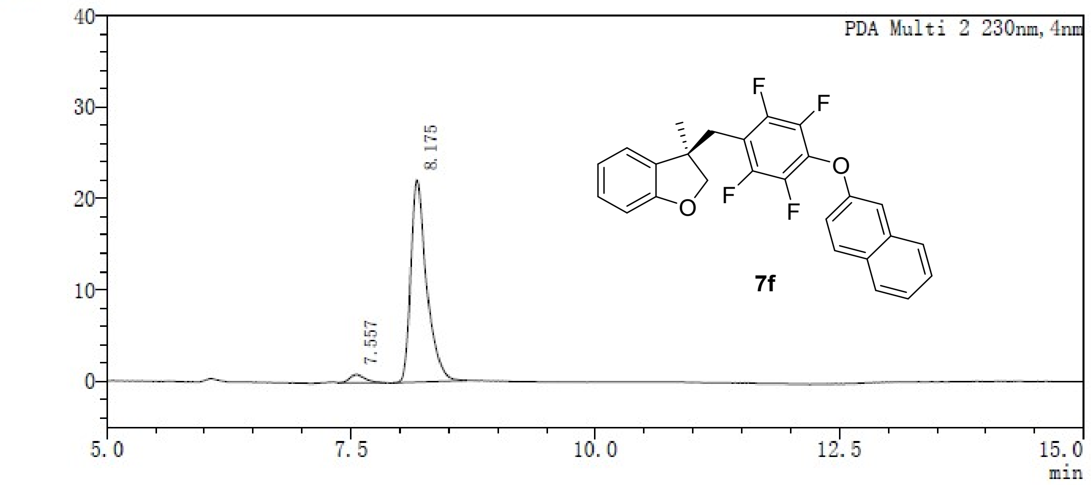

<Peak Table>

PDA Ch2 230nm

No.	Ret. Time (min)	Height	Height%	Area	Area%
1	7.557	904	3.925	9808	3.775
2	8.175	22117	96.075	250023	96.225
Total		23021	100.000	259831	100.000

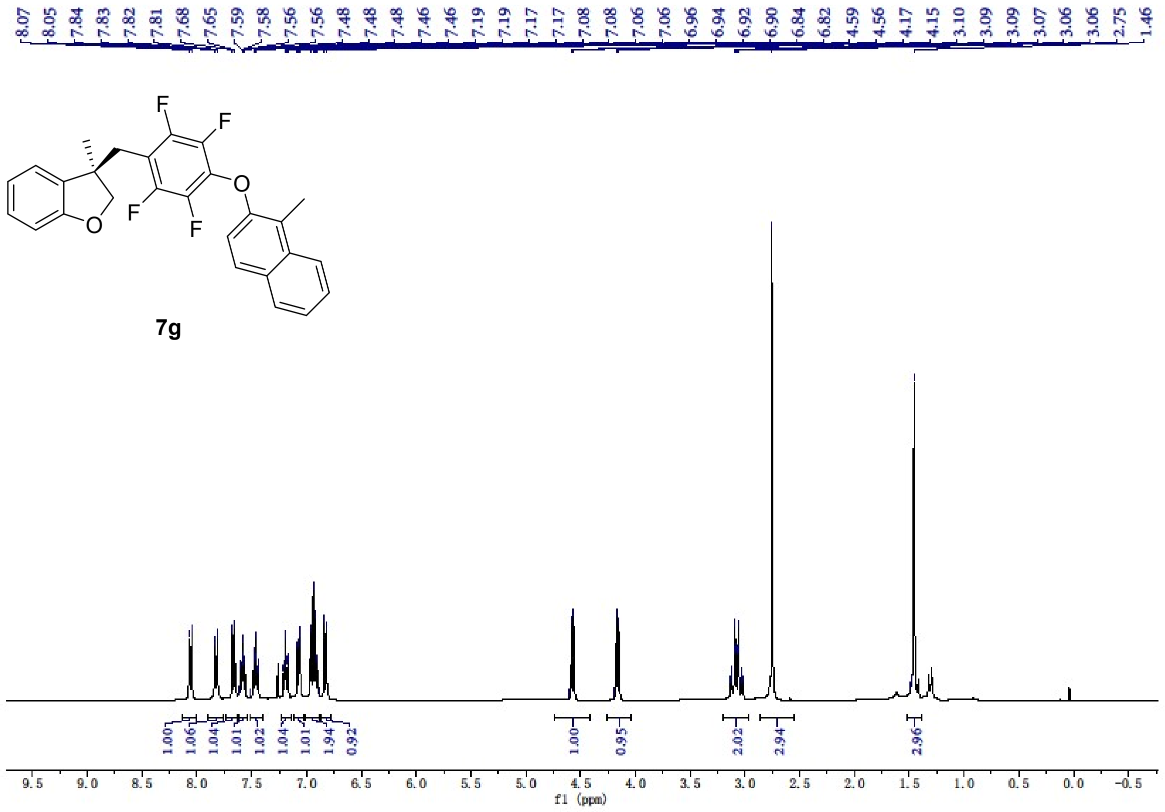

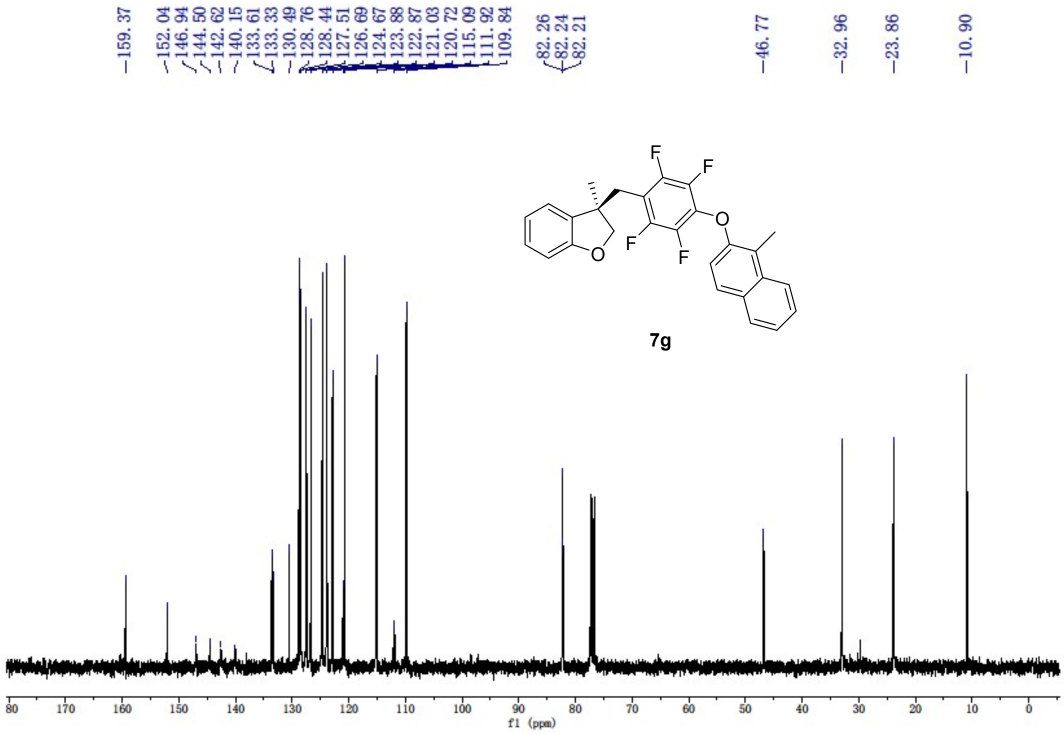
159.37
152.04
146.94
144.50
142.62
140.15
133.61
133.33
130.49
128.76
128.44
127.51
126.69
124.67
123.88
122.87
121.03
120.72
115.09
111.92
109.84
82.26
82.24
82.21
46.77
32.96
23.86
10.90
F
F
F
F
O
O
7g
f1 (ppm)

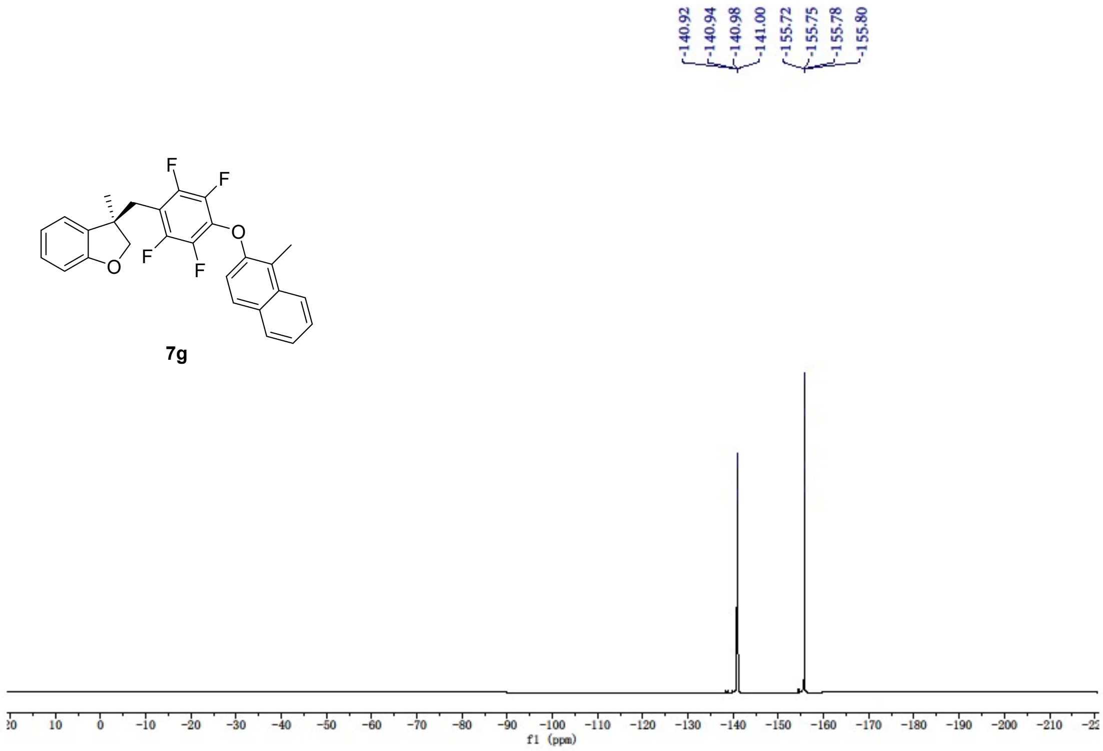
-140.92
-140.94
-140.98
-141.00
-155.72
-155.75
-155.78
-155.80
F
F
F
F
O
O
7g
f1 (ppm)

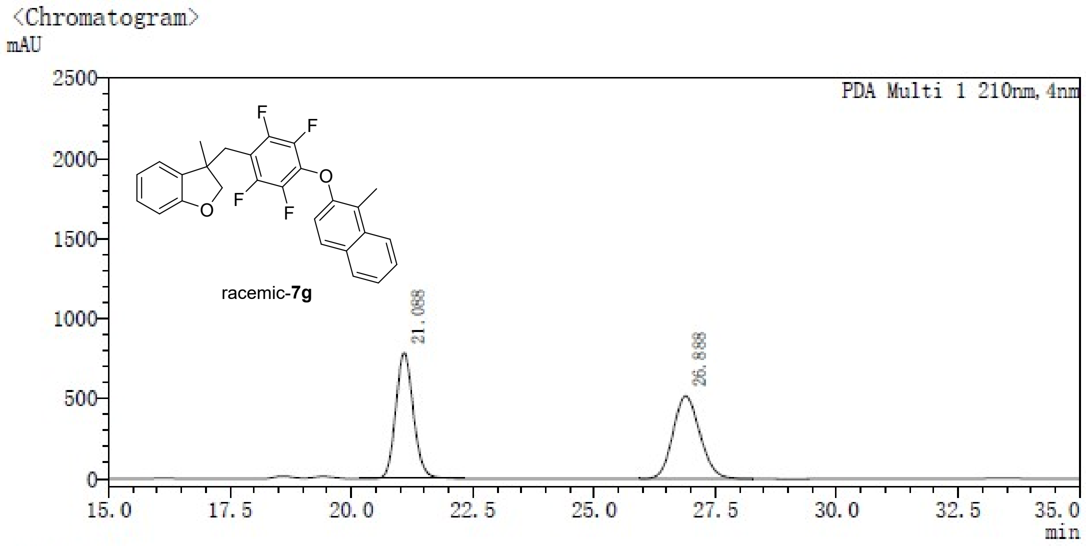

<Peak Table>

PDA Ch1 210nm

No.	Ret. Time(min)	Height	Height%	Area	Area%
1	21.088	786132	60.353	19839404	49.830
2	26.888	516430	39.647	19974385	50.170
Total		1302561	100.000	39813789	100.000

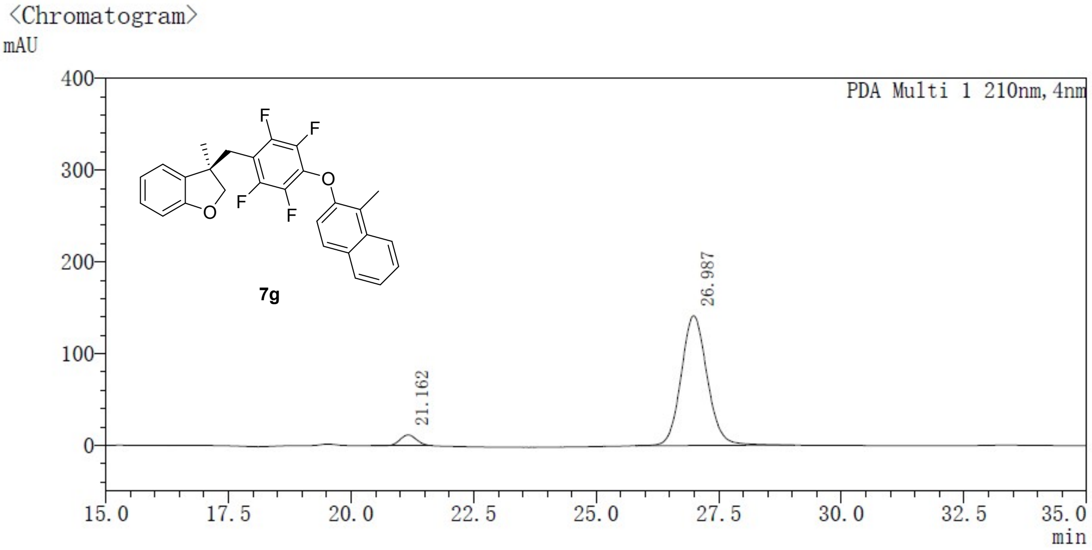

<Peak Table>

PDA Ch1 210nm

No.	Ret. Time(min)	Height	Height%	Area	Area%
1	21.162	11869	7.743	281337	5.095
2	26.987	141412	92.257	5240612	94.905
Total		153281	100.000	5521949	100.000

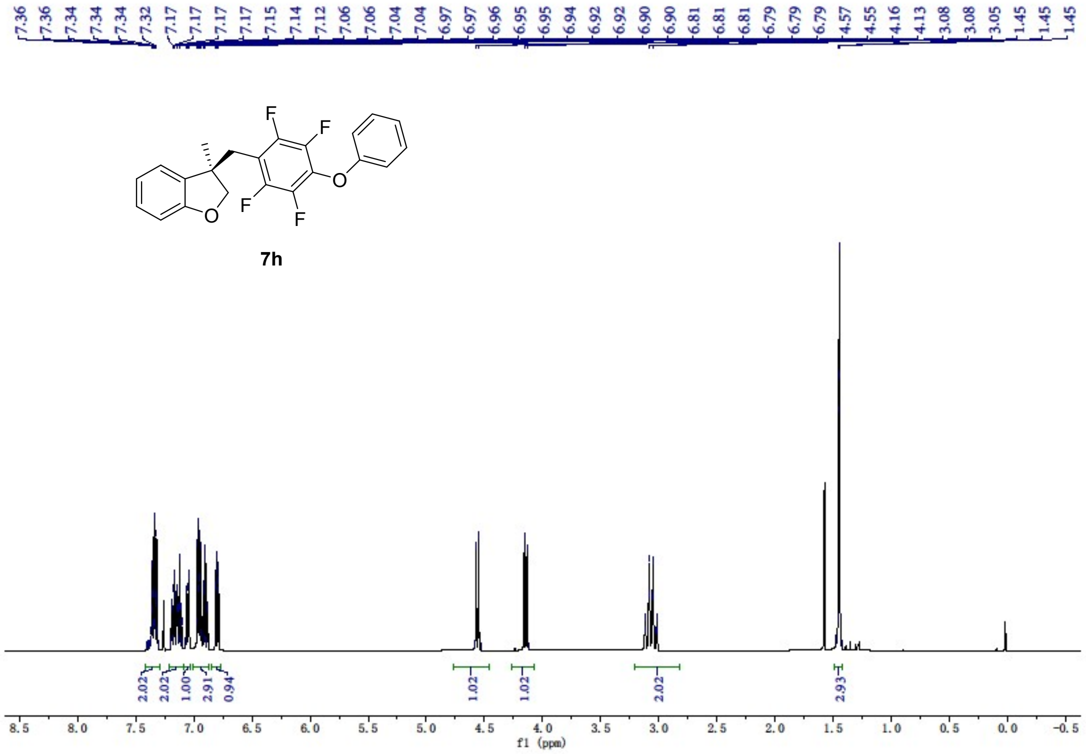
7.36 7.36 7.34 7.34 7.34 7.32 7.17 7.17 7.17 7.17 7.15 7.14 7.12 7.06 7.06 7.04 7.04 6.97 6.97 6.96 6.95 6.95 6.94 6.92 6.92 6.90 6.90 6.81 6.81 6.81 6.79 6.79 6.79 4.57 4.55 4.16 4.13 3.08 3.08 3.05 1.45 1.45 1.45
F
F
O
F
F
O
7h
2.02 2.02 1.00 2.91 0.94 1.02 1.02 2.02 2.93
f1 (ppm)

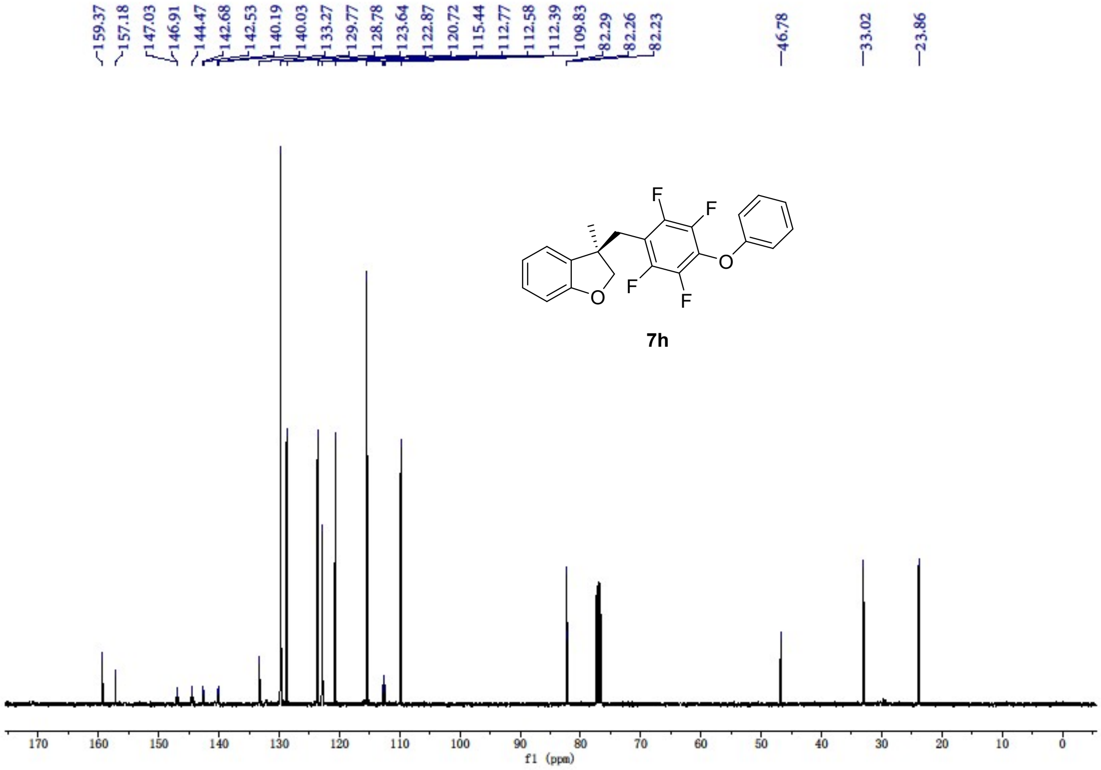
159.37 157.18 147.03 146.91 144.47 142.68 142.53 140.19 140.03 133.27 129.77 128.78 123.64 122.87 120.72 115.44 112.77 112.58 112.39 109.83 82.29 82.26 82.23 46.78 33.02 23.86
F
F
O
F
F
O
7h
f1 (ppm)

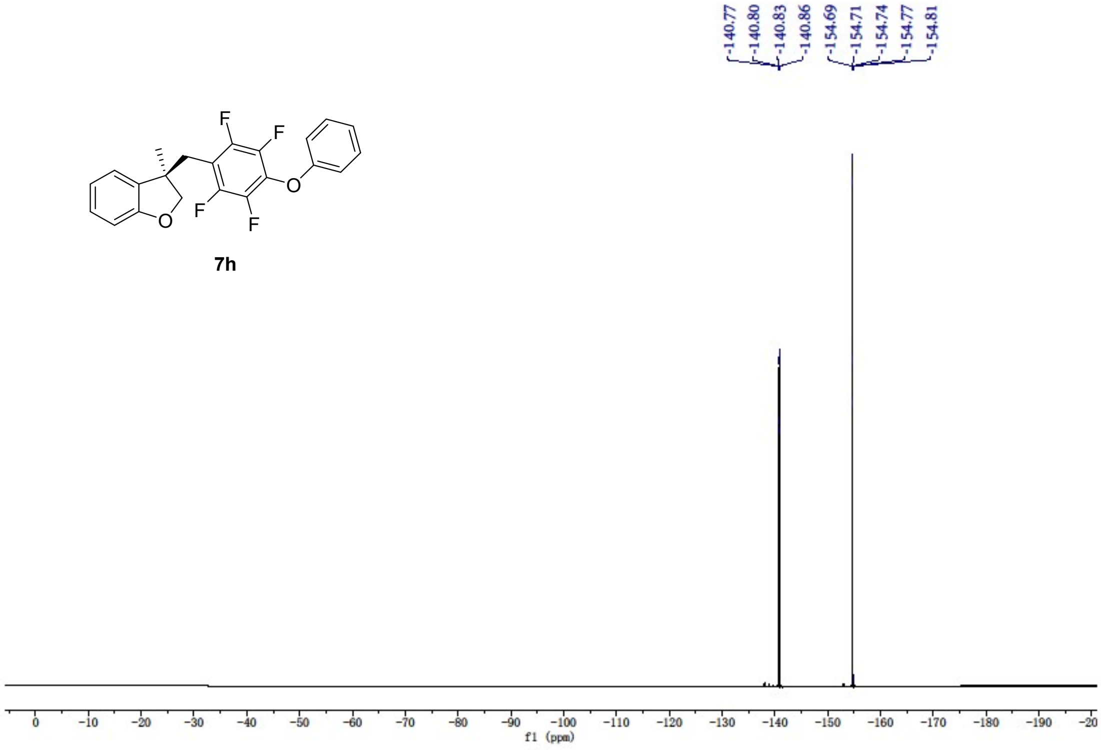

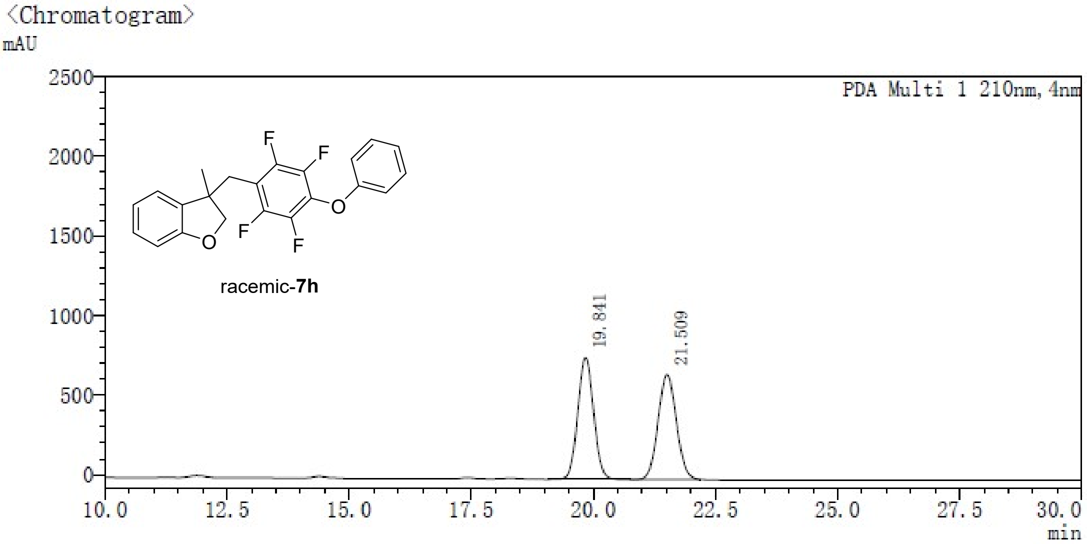

〈Peak Table〉

PDA Ch1 210nm

No.	Ret. Time(min)	Height	Height%	Area	Area%
1	19.841	762873	53.694	17218868	50.036
2	21.509	657895	46.306	17193771	49.964
Total		1420768	100.000	34412639	100.000

<Chromatogram>

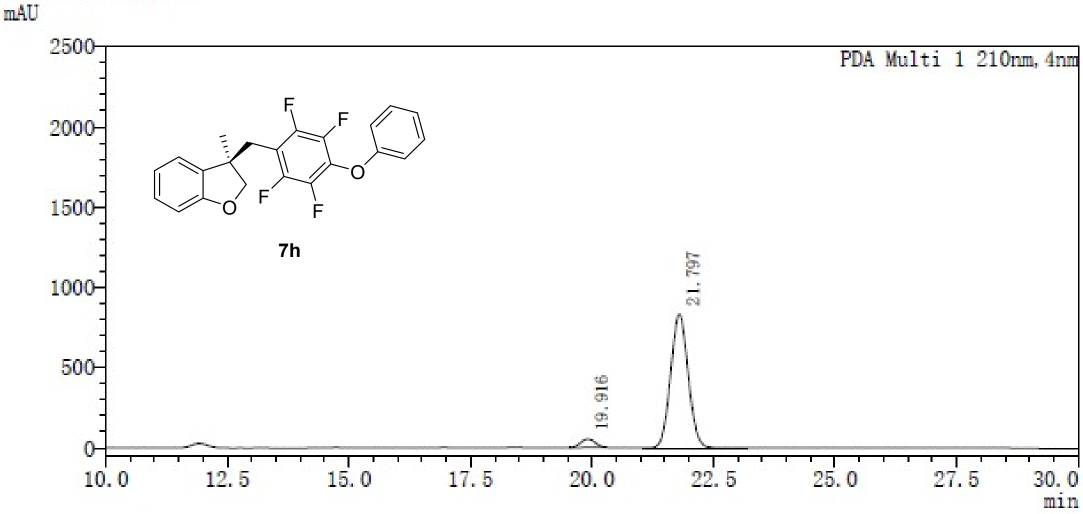

<Peak Table>

PDA Ch1 210nm

No.	Ret.Time(min)	Height	Height%	Area	Area%
1	19.916	53794	6.073	1106647	5.006
2	21.797	831984	93.927	20999612	94.994
Total		885778	100.000	22106258	100.000

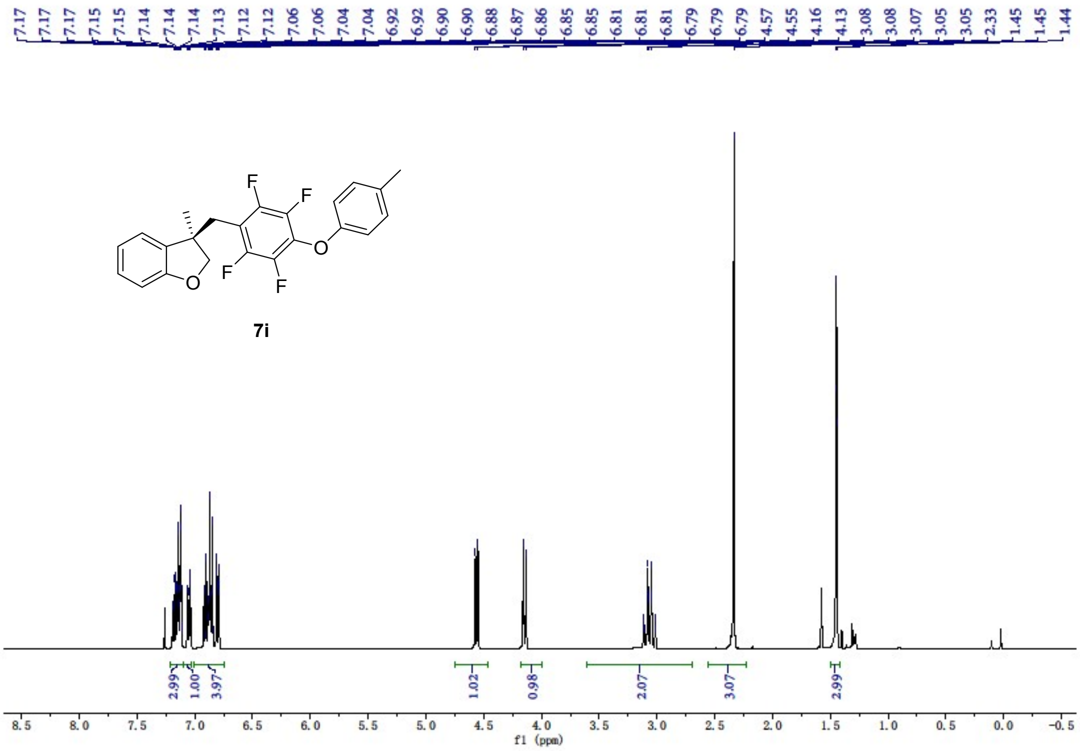

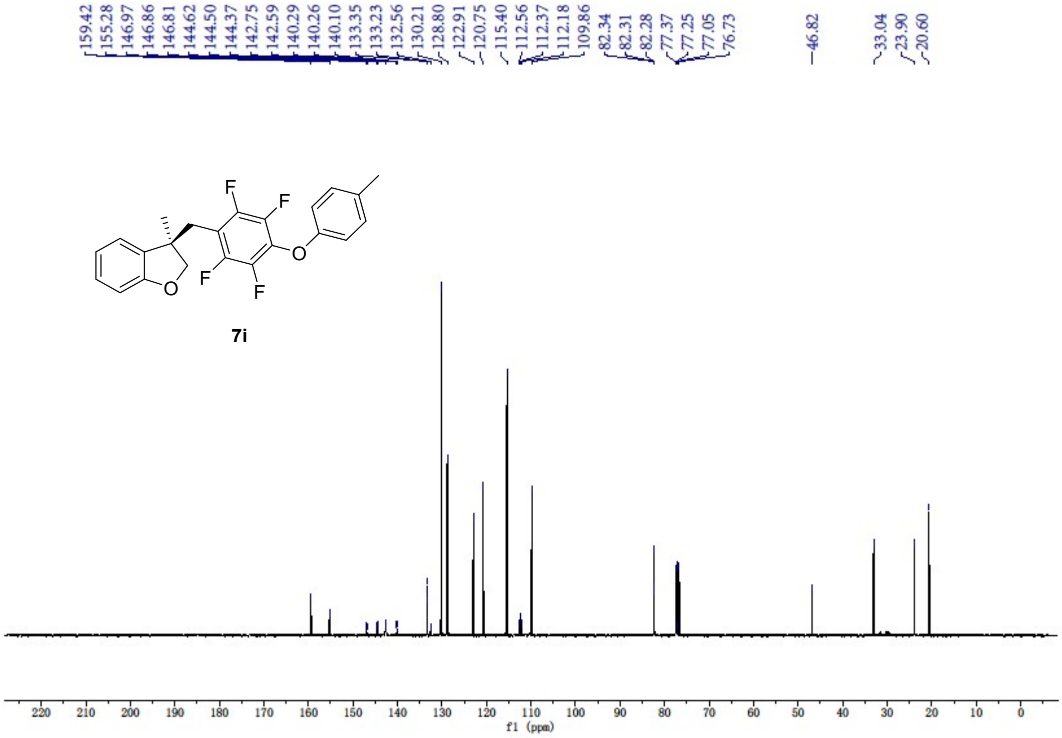
159.42
155.28
146.97
146.86
146.81
144.62
144.50
144.37
142.75
142.59
140.29
140.26
140.10
133.35
133.23
132.56
130.21
128.80
122.91
120.75
115.40
112.56
112.37
112.18
109.86
82.34
82.31
82.28
77.37
77.25
77.05
76.73
46.82
33.04
23.90
20.60
7i
f1 (ppm)

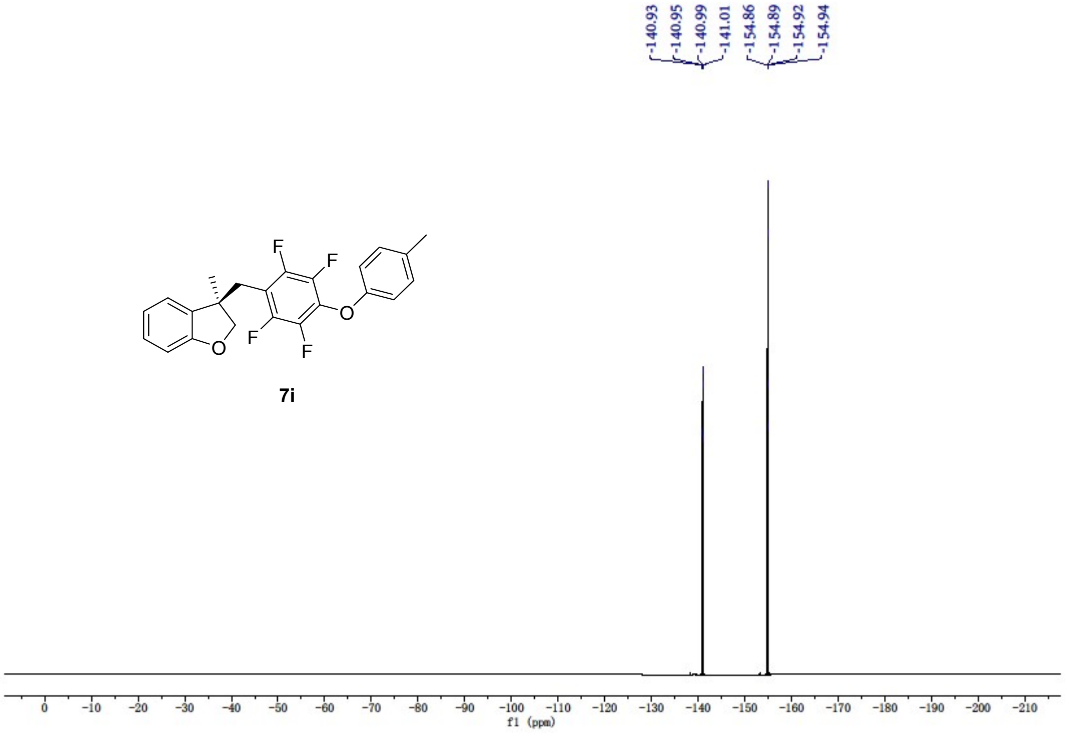
-140.93
-140.95
-140.99
-141.01
-154.86
-154.89
-154.92
-154.94
7i
f1 (ppm)

〈Chromatogram〉

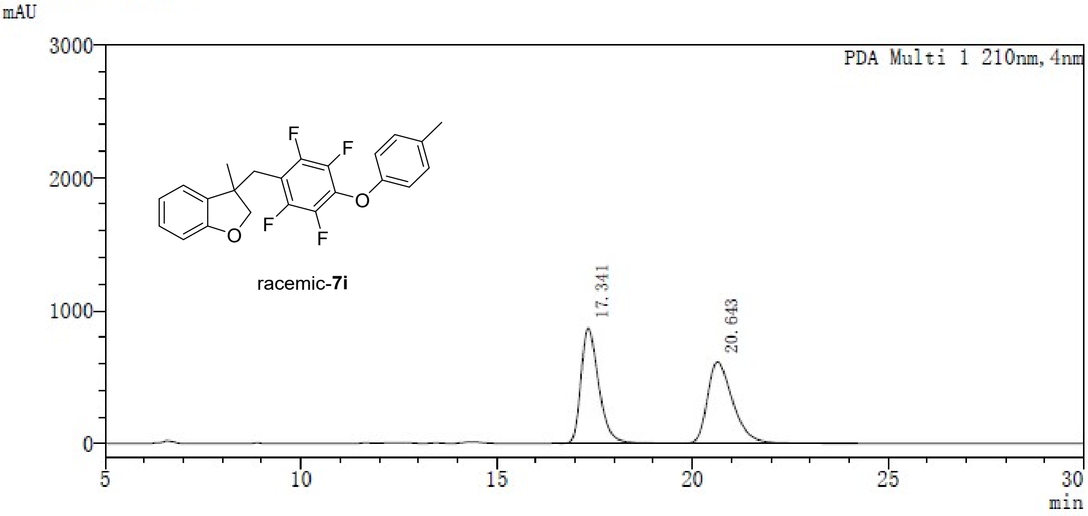

〈Peak Table〉

PDA Ch1 210nm

No.	Ret. Time(min)	Height	Height%	Area	Area%
1	17.341	866840	58.627	27580992	49.911
2	20.643	611727	41.373	27679429	50.089
Total		1478567	100.000	55260421	100.000

〈Chromatogram〉

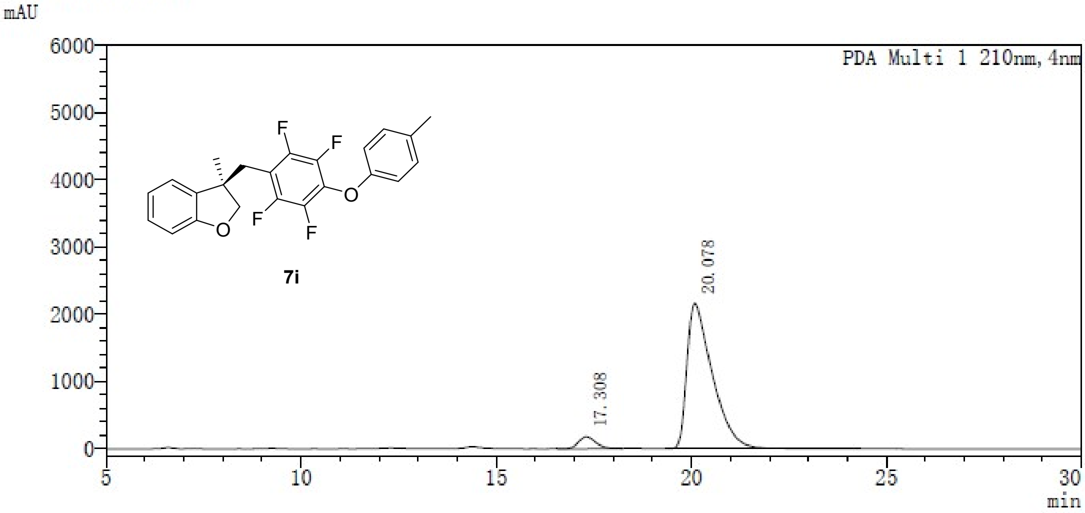

〈Peak Table〉

PDA Ch1 210nm

No.	Ret. Time(min)	Height	Height%	Area	Area%
1	17.308	178894	7.657	5542919	5.353
2	20.078	2157480	92.343	98004924	94.647
Total		2336374	100.000	103547843	100.000

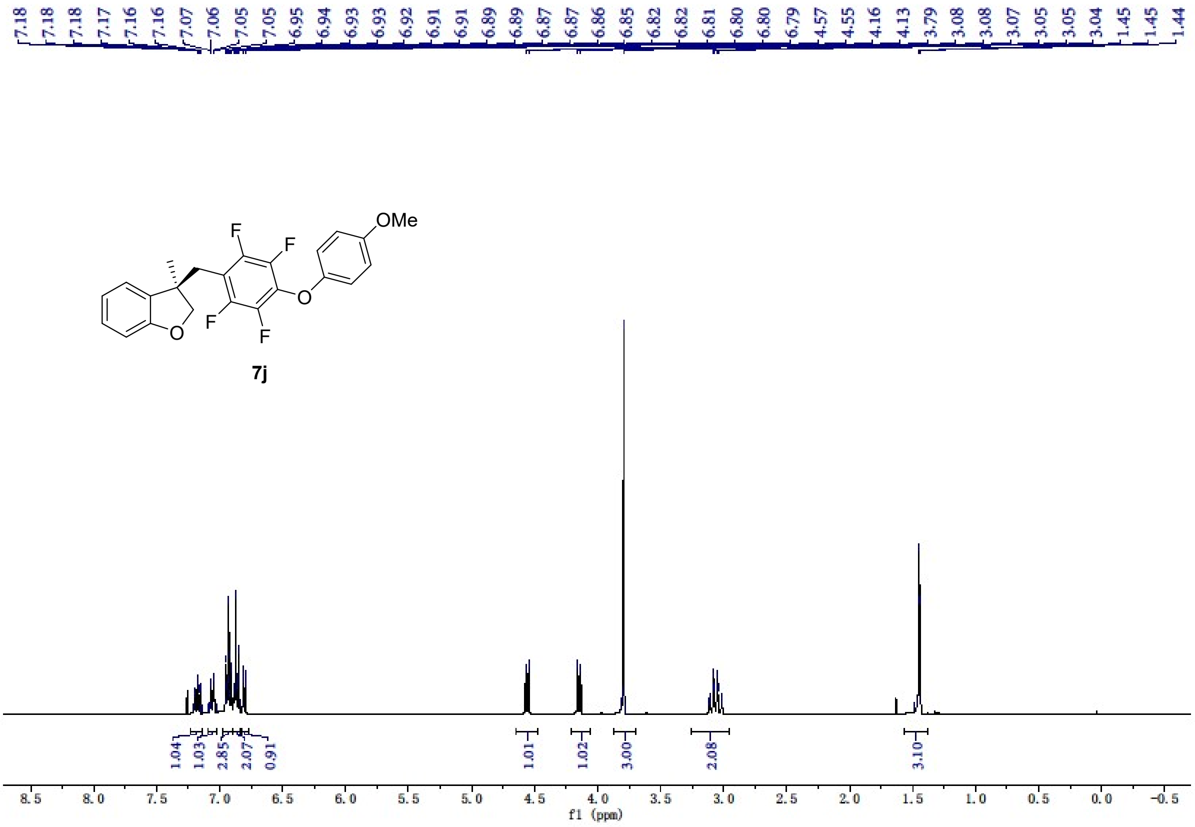
7.18 7.18 7.18 7.17 7.16 7.16 7.07 7.06 7.05 7.05 6.95 6.94 6.93 6.93 6.92 6.91 6.91 6.89 6.89 6.87 6.87 6.86 6.85 6.82 6.82 6.81 6.80 6.80 6.79 4.57 4.55 4.16 4.13 3.79 3.08 3.08 3.07 3.05 3.05 3.04 1.45 1.45 1.44
F
F
OMe
O
F
F
O
7j
1.04 1.03 2.85 2.07 0.91 1.01 1.02 3.00 2.08 3.10
f1 (ppm)

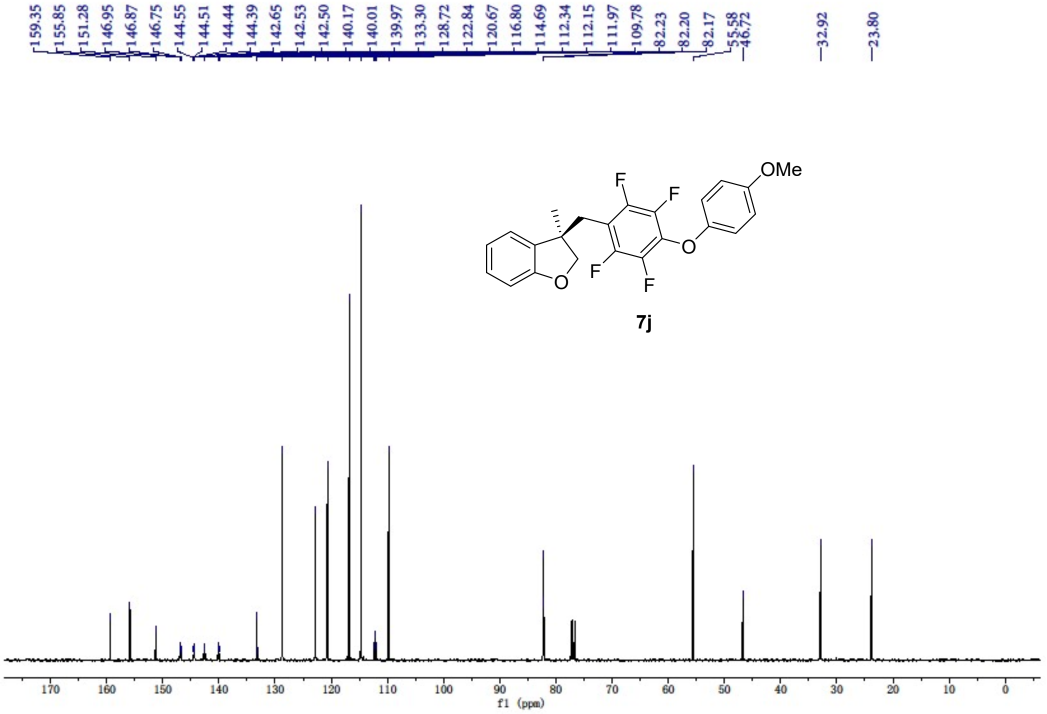
159.35 155.85 151.28 146.95 146.87 146.75 144.55 144.51 144.44 144.39 142.65 142.53 142.50 140.17 140.01 139.97 133.30 128.72 122.84 120.67 116.80 114.69 112.34 112.15 111.97 109.78 82.23 82.20 82.17 55.58 46.72 32.92 23.80
F
F
OMe
O
F
F
O
7j
f1 (ppm)

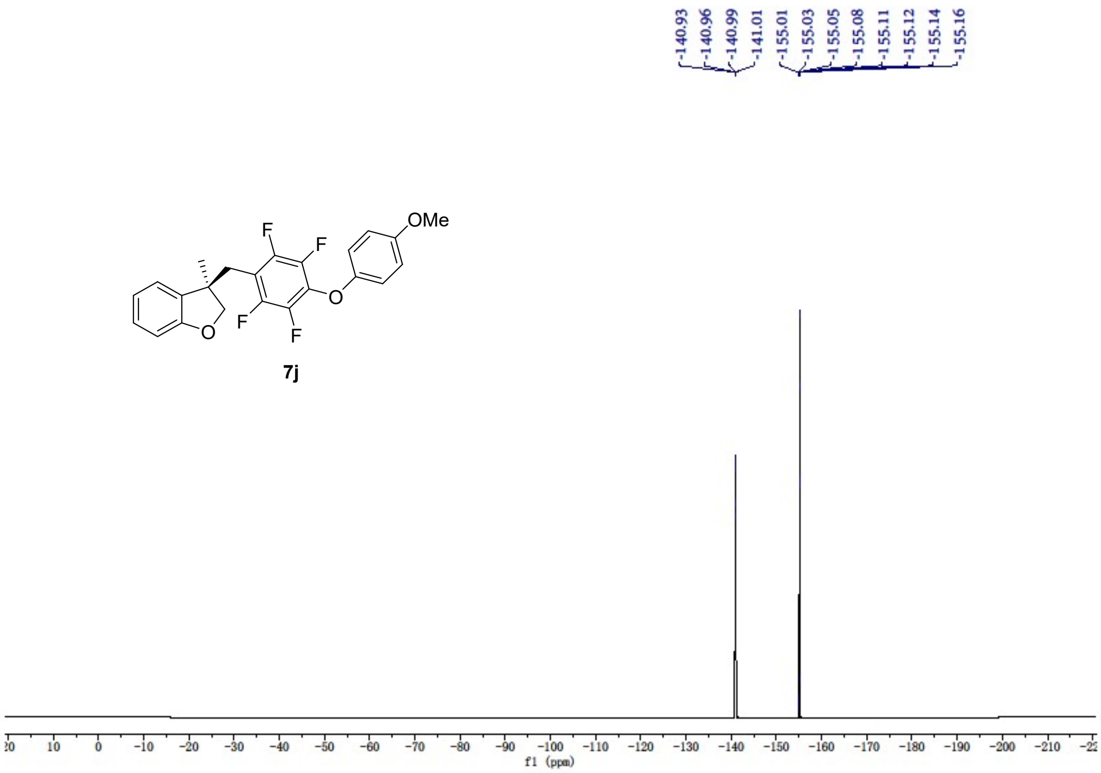

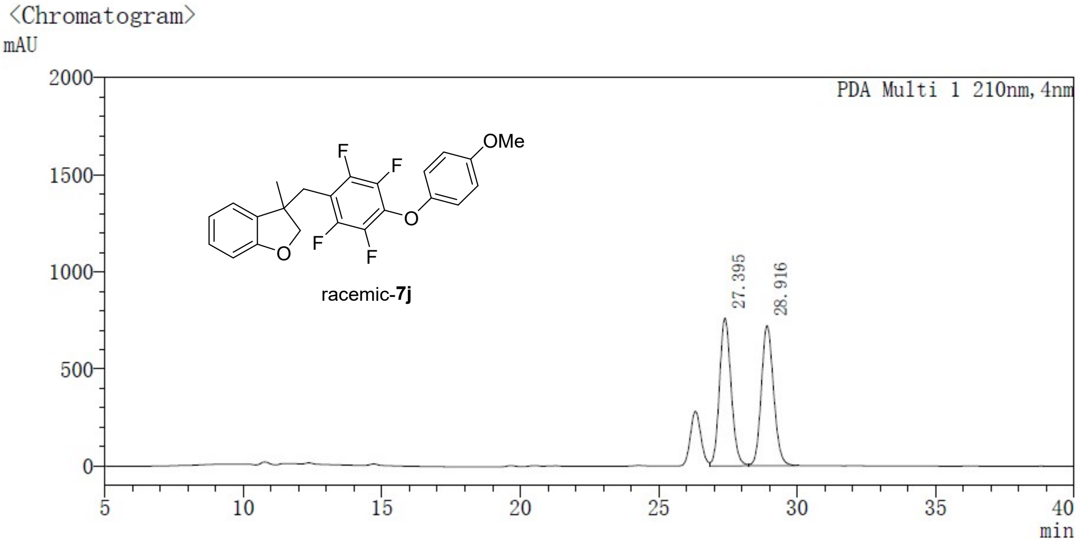

<Peak Table>

PDA Ch1 210nm

No.	Ret.Time(min)	Height	Height%	Area	Area%
1	27.395	760543	51.326	22207130	49.912
2	28.916	721254	48.674	22285139	50.088
Total		1481797	100.000	44492269	100.000

<Chromatogram>

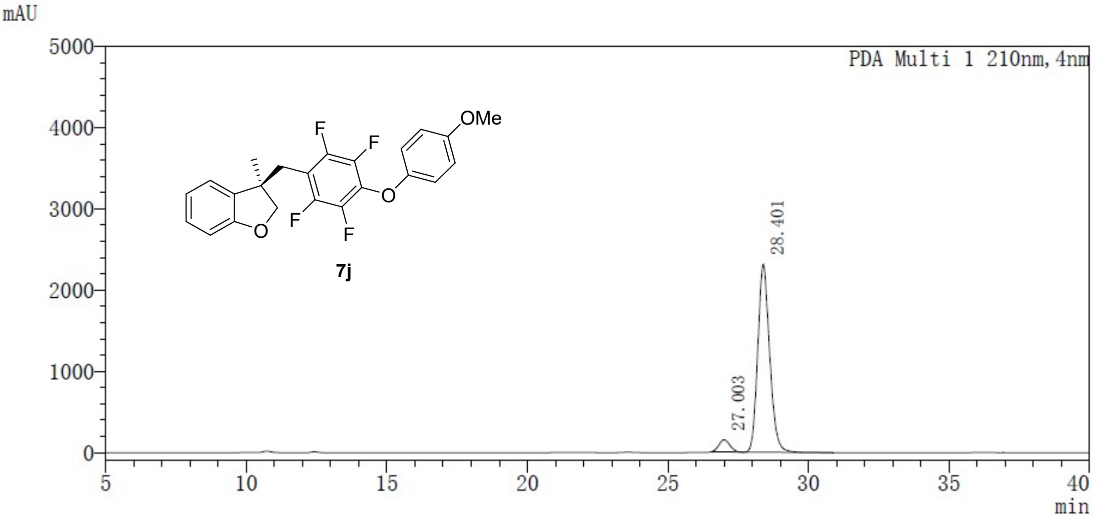

<Peak Table>

PDA Ch1 210nm

No.	Ret.Time(min)	Height	Height%	Area	Area%
1	27.003	150541	6.101	4081788	5.656
2	28.401	2316803	93.899	68088196	94.344
Total		2467344	100.000	72169985	100.000

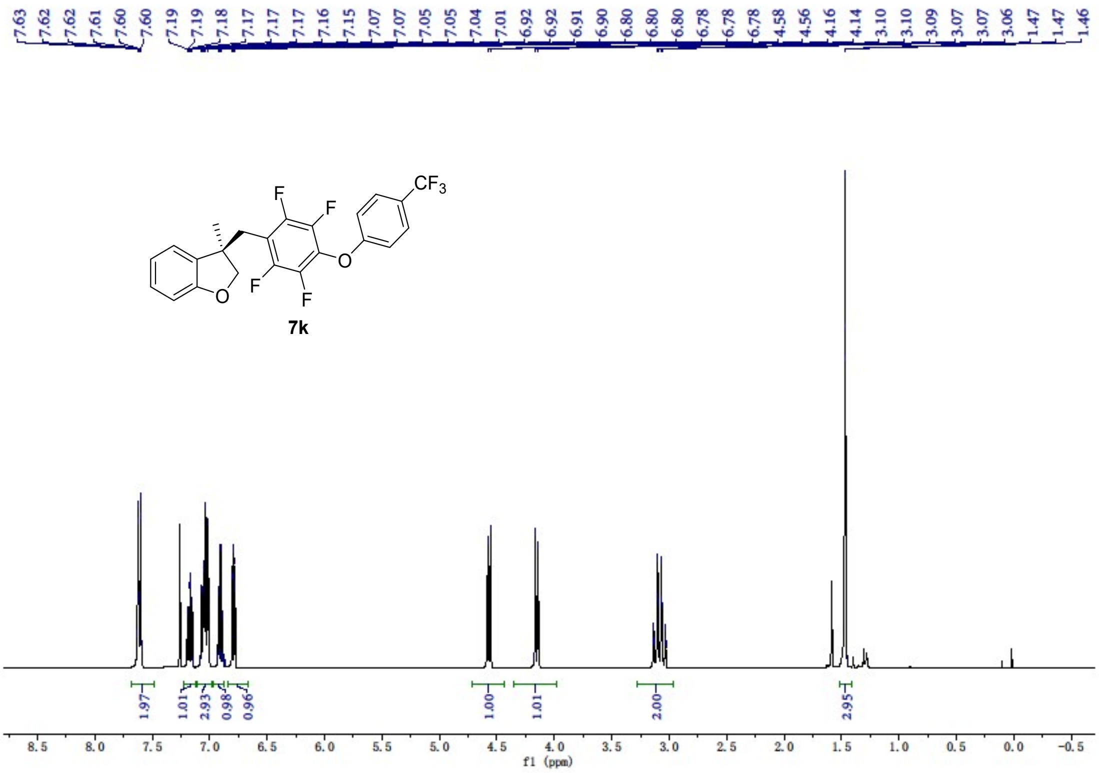

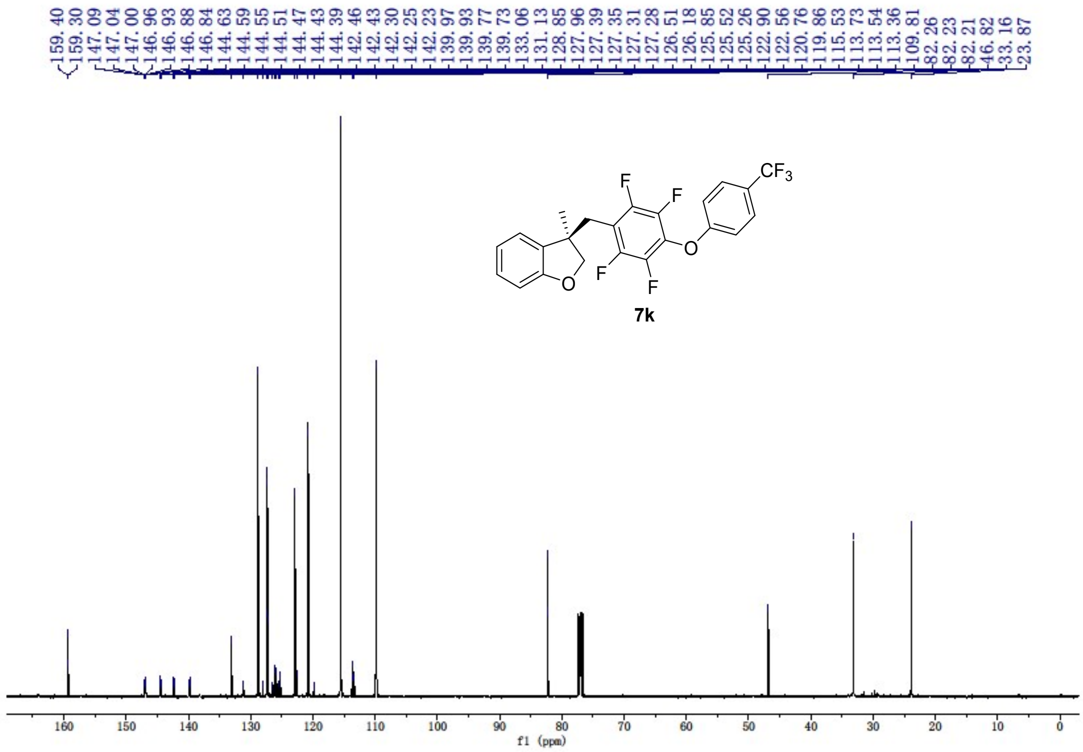
159.40
159.30
147.09
147.04
147.00
146.96
146.93
146.88
146.84
144.63
144.59
144.55
144.51
144.47
144.43
144.39
142.46
142.43
142.30
142.25
142.23
139.97
139.93
139.77
139.73
133.06
131.13
128.85
127.96
127.39
127.35
127.31
127.28
126.51
126.18
125.85
125.52
125.26
122.90
122.56
120.76
119.86
115.53
113.73
113.54
113.36
109.81
82.26
82.23
82.21
46.82
33.16
23.87
CF3
F
F
F
F
O
O
7k
f1 (ppm)

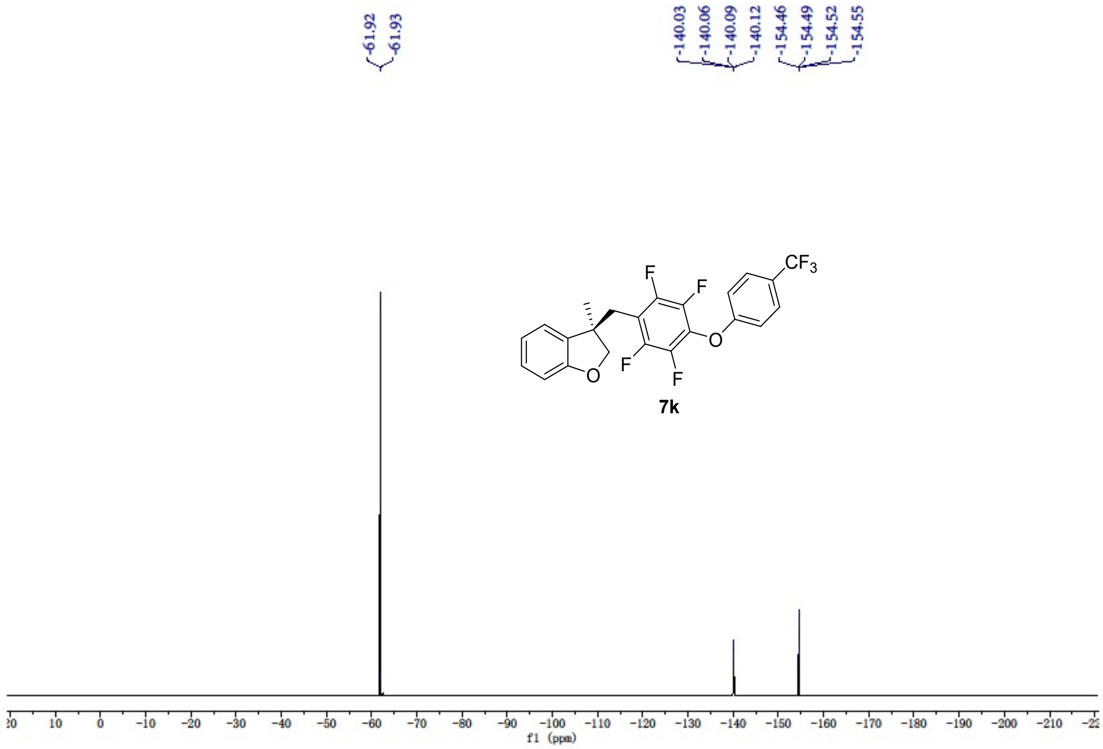
-61.92
-61.93
-140.03
-140.06
-140.09
-140.12
-154.46
-154.49
-154.52
-154.55
CF3
F
F
F
F
O
O
7k
f1 (ppm)

<Chromatogram>

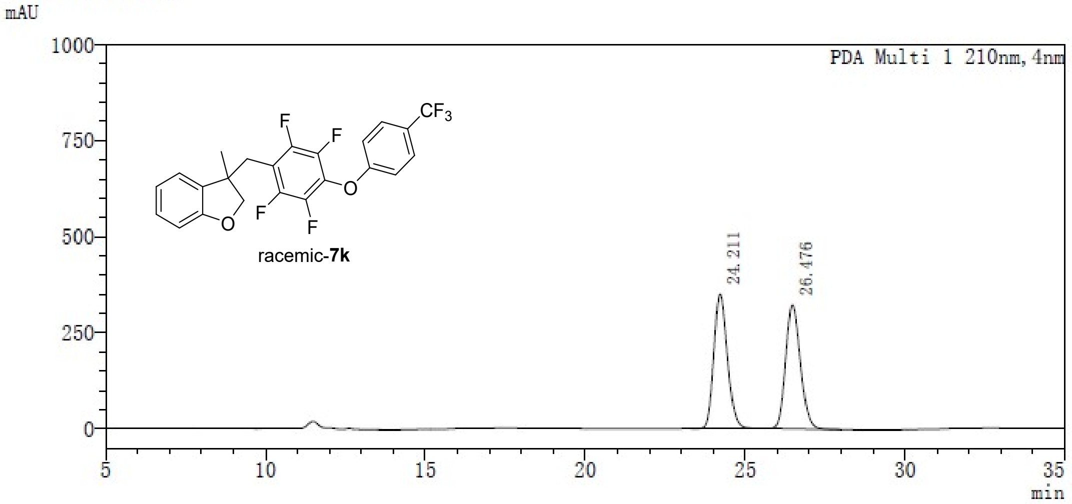

<Peak Table>

PDA Ch1 210nm

No.	Ret. Time (min)	Height	Height%	Area	Area%
1	24.211	349514	52.006	10315712	49.914
2	26.476	322545	47.994	10351293	50.086
Total		672060	100.000	20667004	100.000

<Chromatogram>

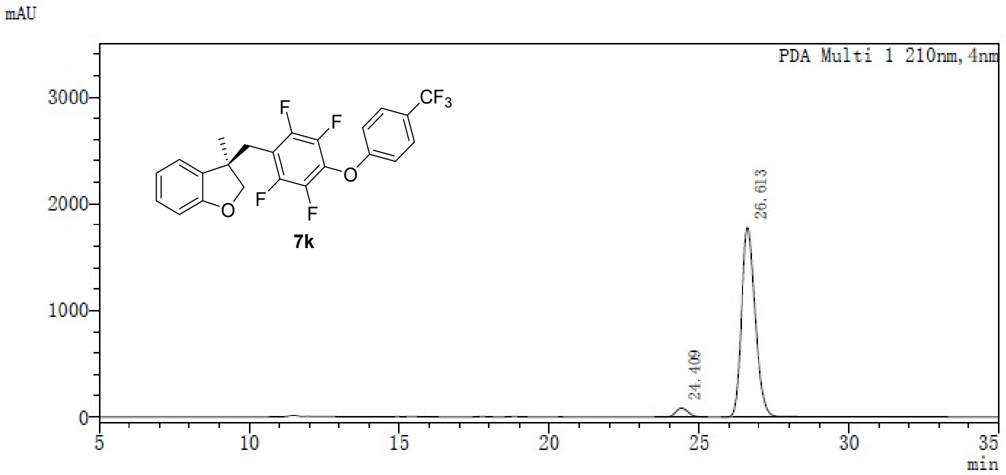

<Peak Table>

PDA Ch1 210nm

No.	Ret. Time (min)	Height	Height%	Area	Area%
1	24.409	84324	4.531	2499006	4.240
2	26.613	1776731	95.469	56440430	95.760
Total		1861055	100.000	58939436	100.000

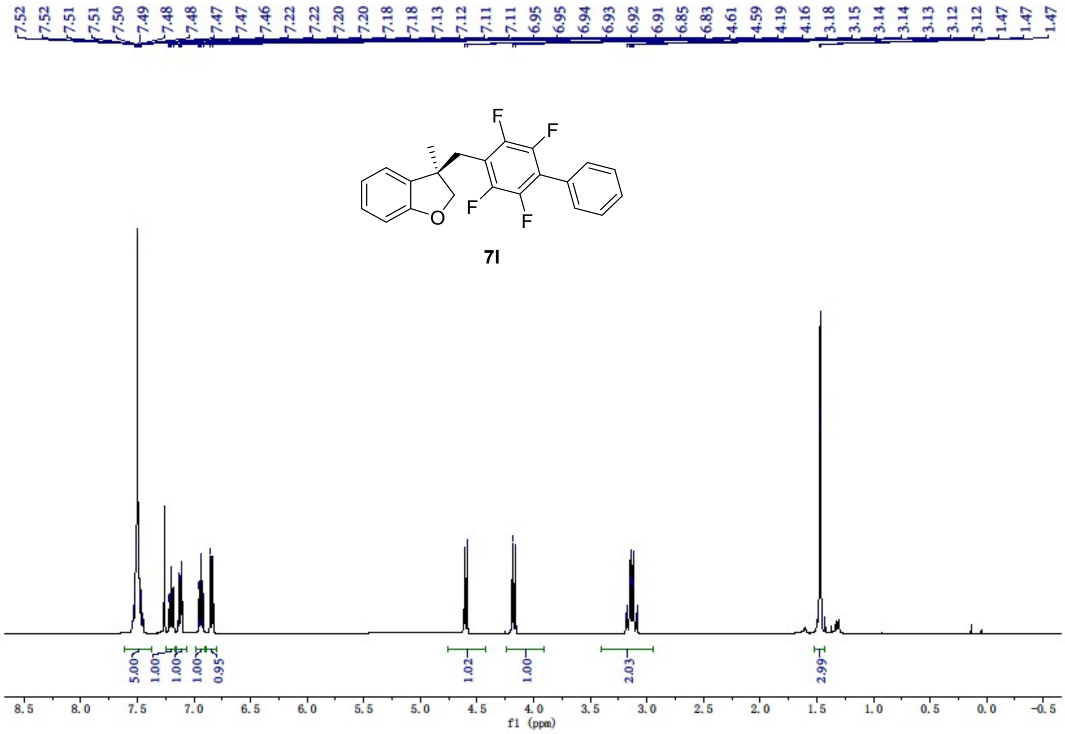
7.52 7.52 7.51 7.51 7.50 7.49 7.48 7.48 7.47 7.47 7.46 7.22 7.22 7.20 7.20 7.18 7.18 7.13 7.12 7.11 7.11 6.95 6.95 6.94 6.93 6.92 6.91 6.85 6.83 4.61 4.59 4.19 4.16 3.18 3.15 3.14 3.14 3.13 3.12 3.12 1.47 1.47 1.47
F
F
F
F
O
7l
5.00 1.00 1.00 1.00 0.95 1.02 1.00 2.03 2.99
8.5 8.0 7.5 7.0 6.5 6.0 5.5 5.0 4.5 4.0 3.5 3.0 2.5 2.0 1.5 1.0 0.5 0.0 -0.5
f1 (ppm)

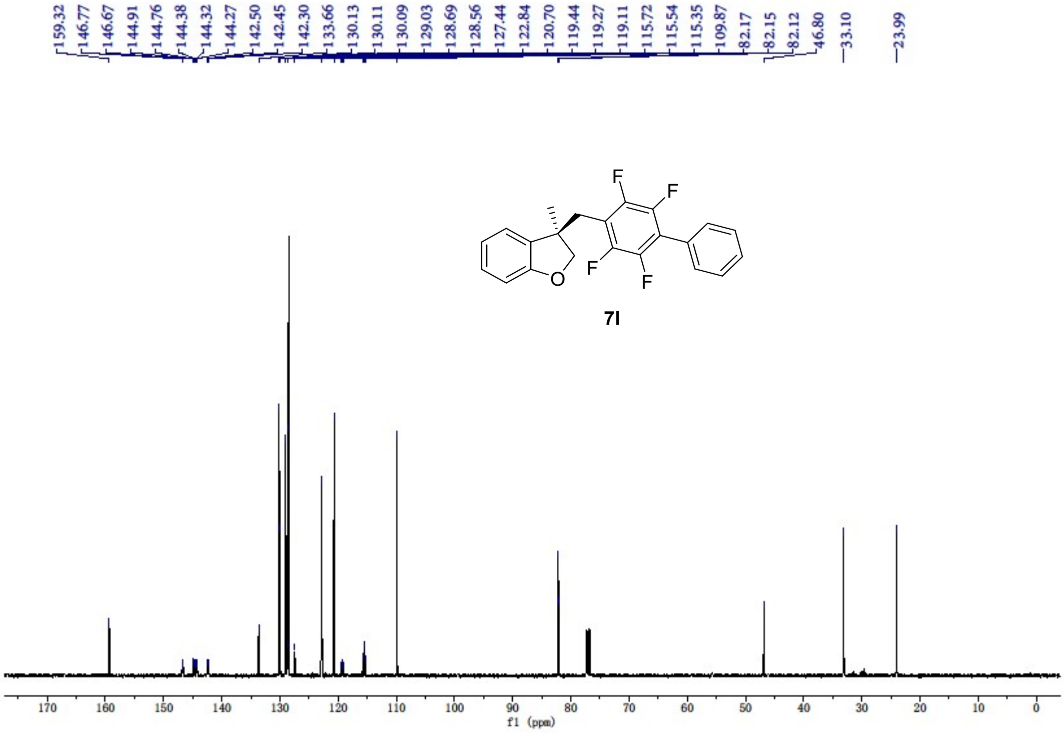
159.32 146.77 146.67 144.91 144.76 144.38 144.32 144.27 142.50 142.45 142.30 133.66 130.13 130.11 130.09 129.03 128.69 128.56 127.44 122.84 120.70 119.44 119.27 119.11 115.72 115.54 115.35 109.87 82.17 82.15 82.12 46.80 33.10 23.99
F
F
F
F
O
7l
170 160 150 140 130 120 110 100 90 80 70 60 50 40 30 20 10 0
f1 (ppm)

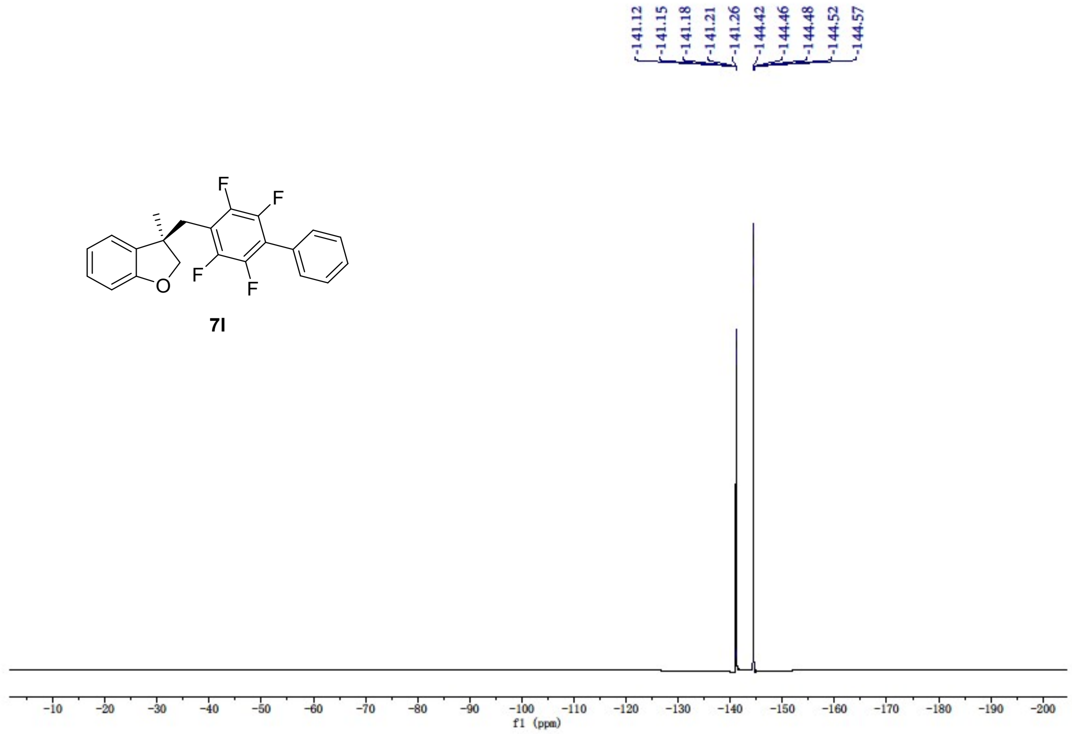

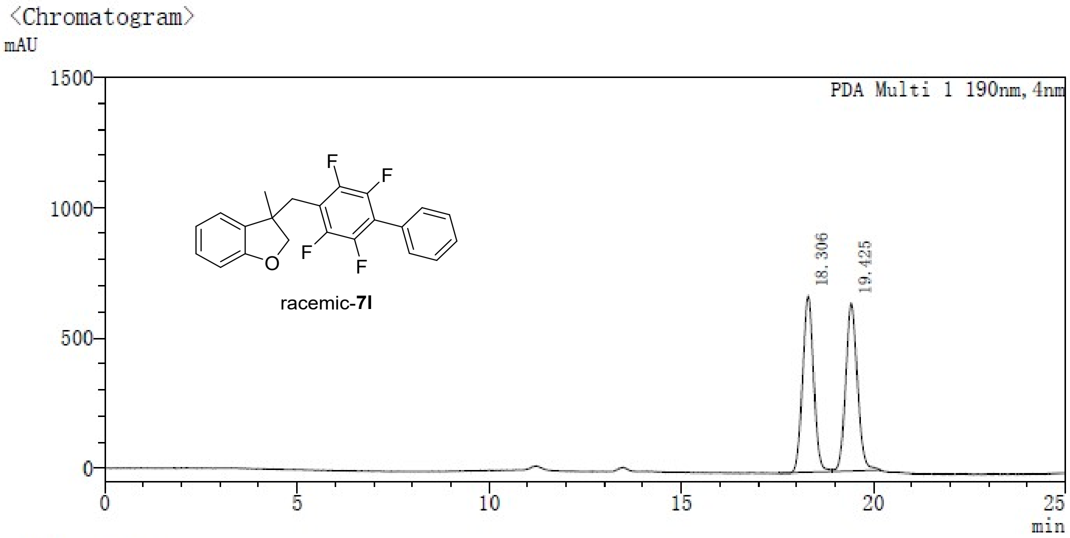

<Peak Table>

PDA Ch1 190nm

No.	Ret.Time(min)	Height	Height%	Area	Area%
1	18.306	675724	51.200	13566758	49.673
2	19.425	644051	48.800	13745373	50.327
Total		1319774	100.000	27312131	100.000

<Chromatogram>

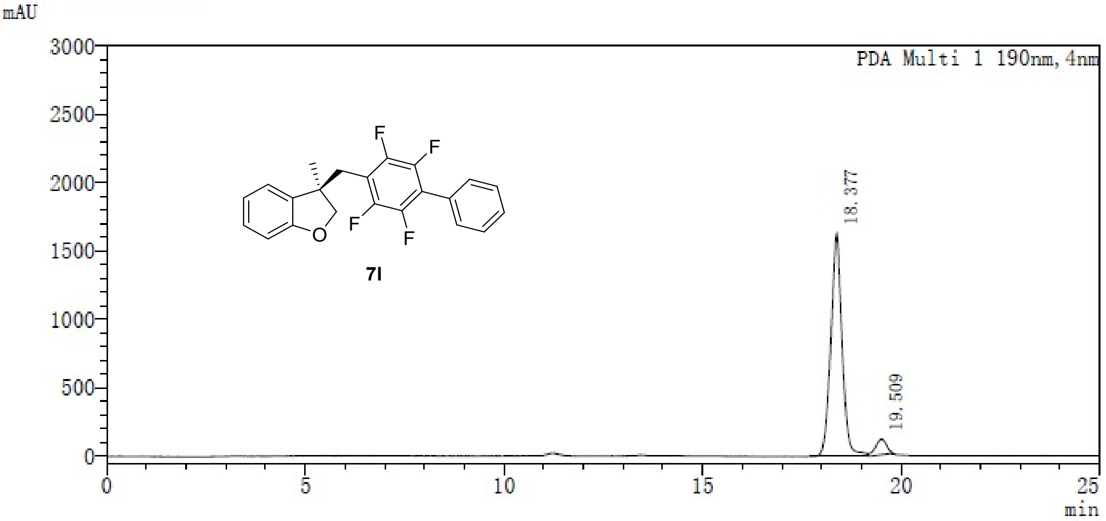

<Peak Table>

PDA Ch1 190nm

No.	Ret. Time (min)	Height	Height%	Area	Area%
1	18.377	1627633	93.526	31996404	93.628
2	19.509	112664	6.474	2177743	6.372
Total		1740297	100.000	34174147	100.000

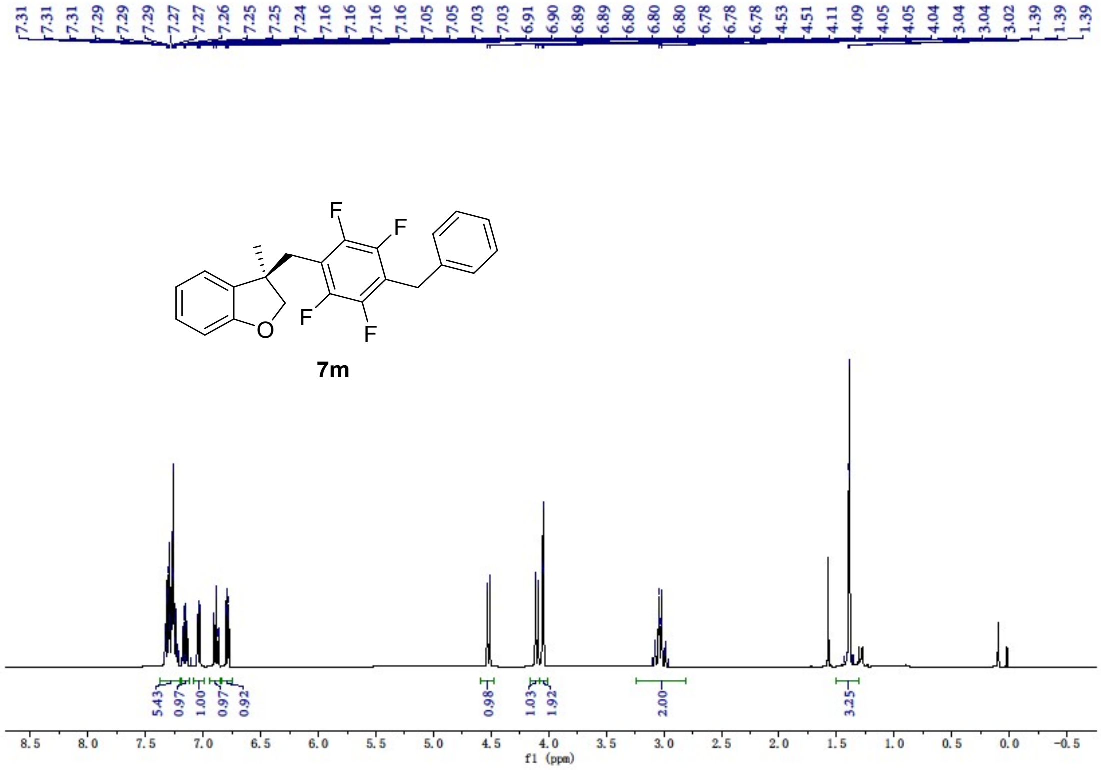

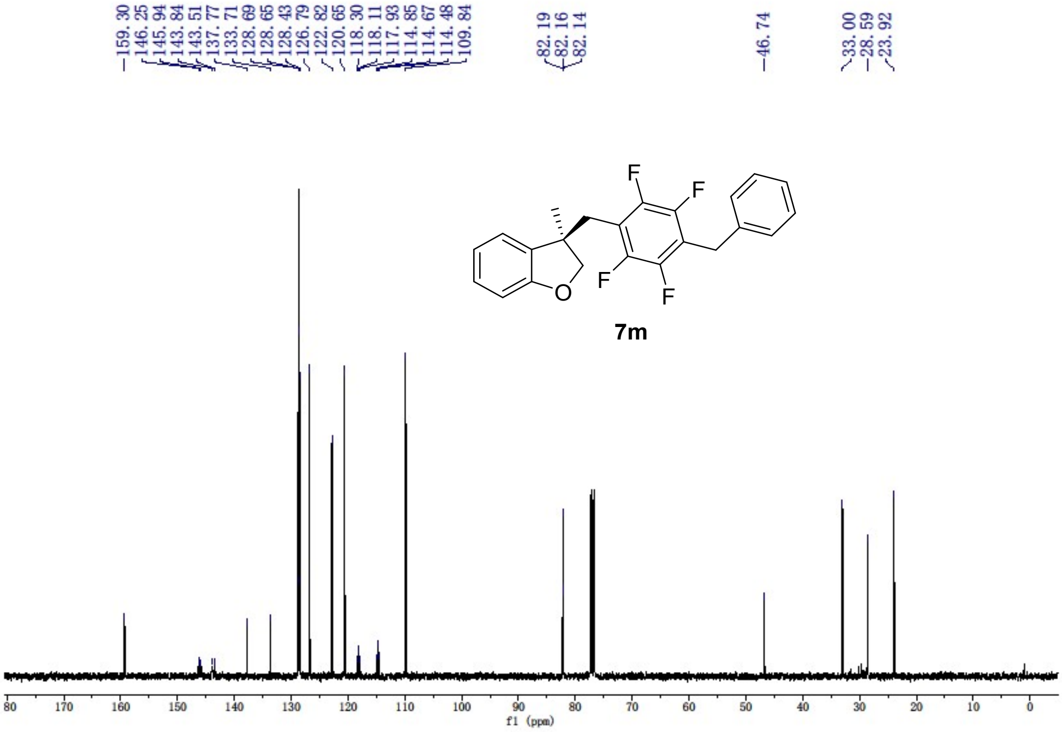
159.30
146.25
145.94
143.84
143.51
137.77
133.71
128.69
128.65
128.43
126.79
122.82
120.65
118.30
118.11
117.93
114.85
114.67
114.48
109.84
82.19
82.16
82.14
46.74
33.00
28.59
23.92
F
F
F
F
O
7m
f1 (ppm)

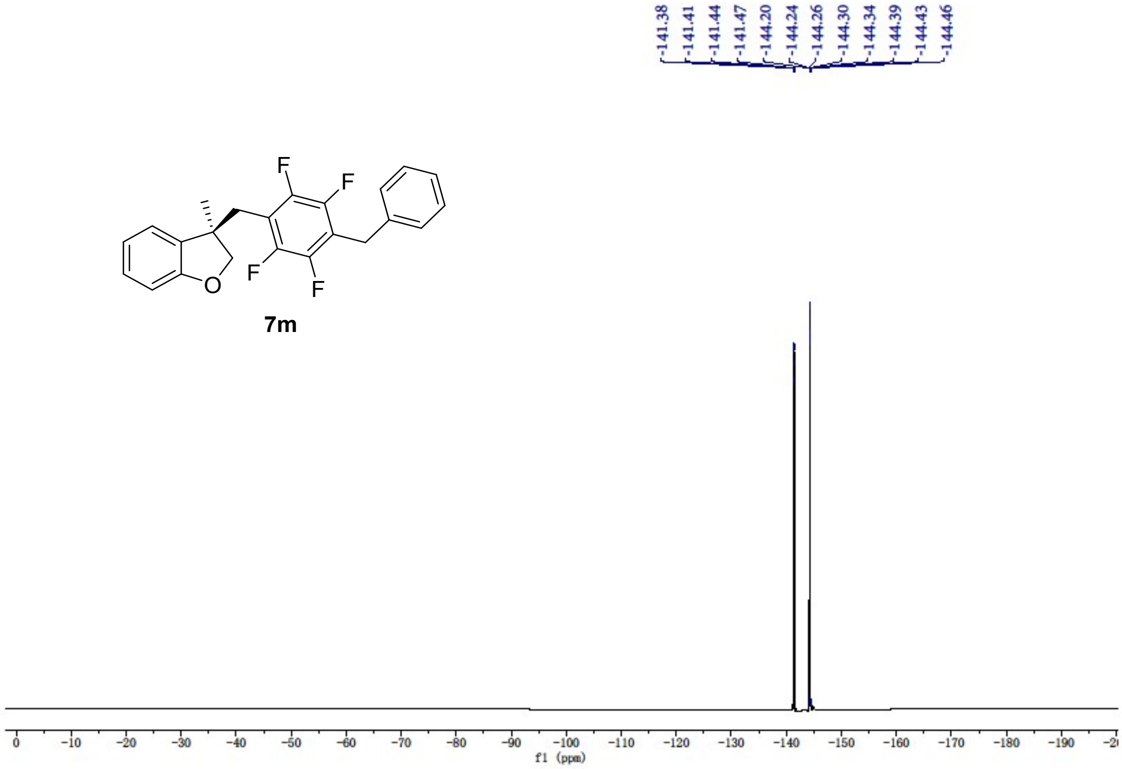
-141.38
-141.41
-141.44
-141.47
-144.20
-144.24
-144.26
-144.30
-144.34
-144.39
-144.43
-144.46
F
F
F
F
O
7m
f1 (ppm)

<Chromatogram>

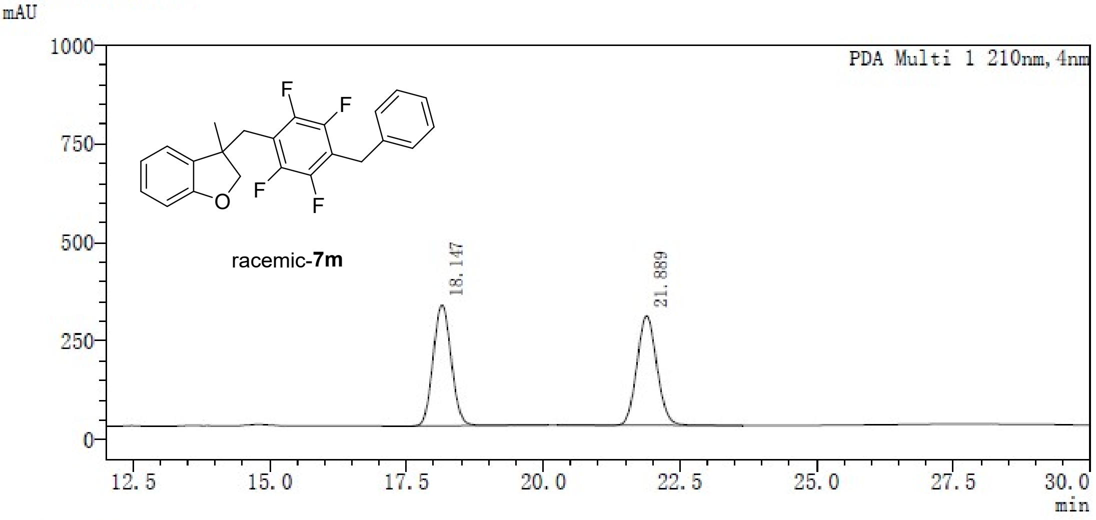

<Peak Table>

PDA Ch1 210nm

No.	Ret. Time (min)	Height	Height%	Area	Area%
1	18.147	306262	52.515	7081566	50.220
2	21.889	276929	47.485	7019657	49.780
Total		583191	100.000	14101223	100.000

<Chromatogram>

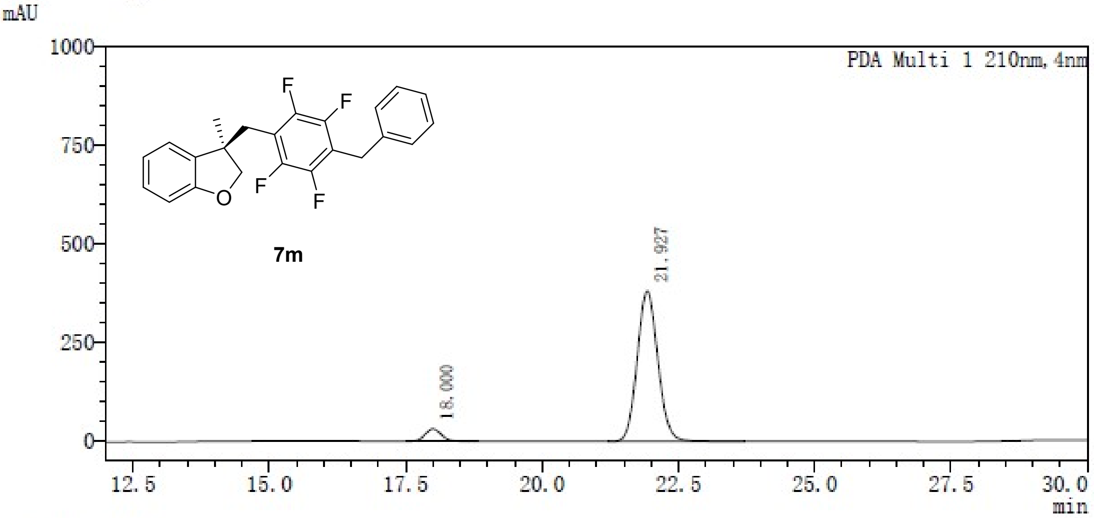

<Peak Table>

PDA Ch1 210nm

No.	Ret. Time (min)	Height	Height%	Area	Area%
1	18.000	31304	7.579	649078	6.043
2	21.927	381746	92.421	10091898	93.957
Total		413051	100.000	10740976	100.000

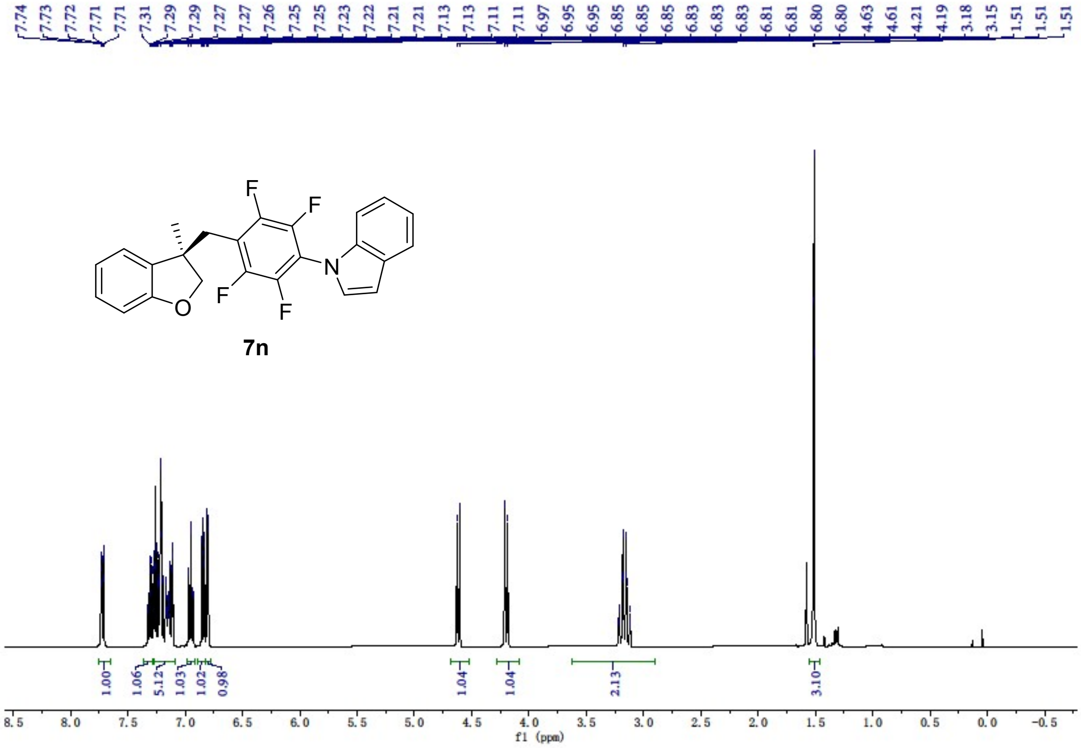
7.74 7.73 7.72 7.71 7.71 7.31 7.29 7.29 7.27 7.27 7.26 7.25 7.25 7.23 7.22 7.21 7.21 7.13 7.13 7.11 7.11 6.97 6.95 6.95 6.85 6.85 6.85 6.83 6.83 6.83 6.81 6.81 6.80 6.80 4.63 4.61 4.21 4.19 3.18 3.15 1.51 1.51 1.51
F
F
N
F
F
O
7n
1.00 1.06 5.12 1.03 1.02 0.98 1.04 1.04 2.13 3.10
f1 (ppm)

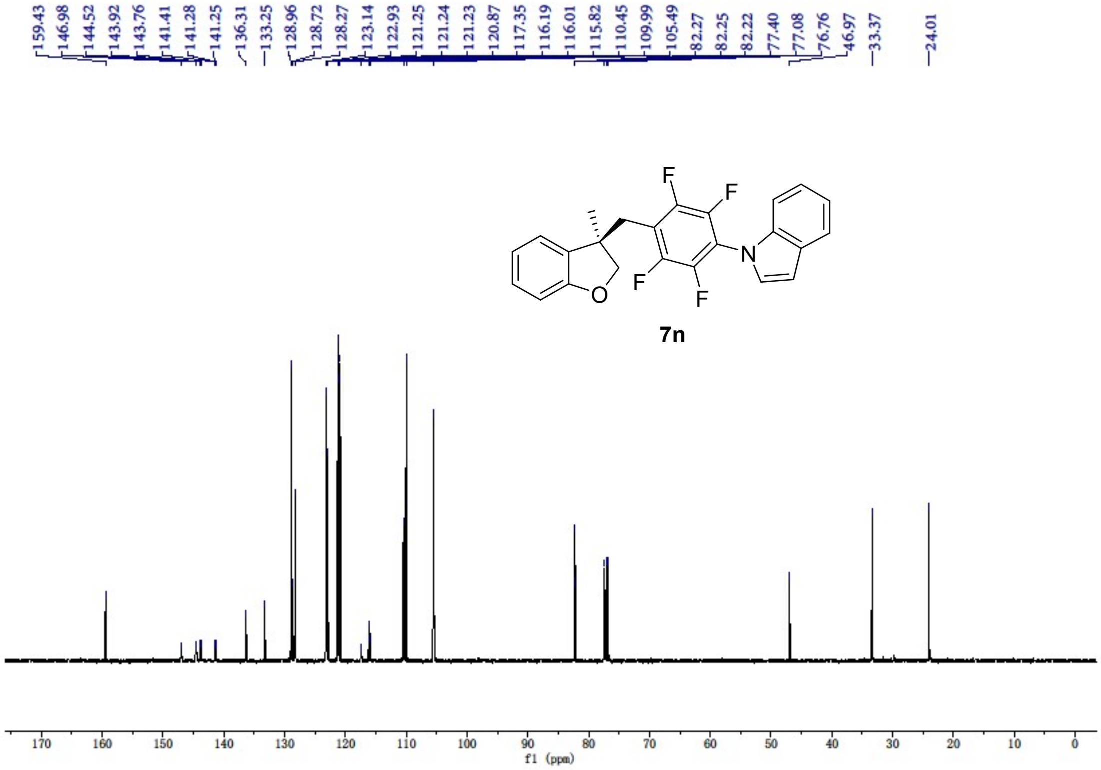
159.43 146.98 144.52 143.92 143.76 141.41 141.28 141.25 136.31 133.25 128.96 128.72 128.27 123.14 122.93 121.25 121.24 121.23 120.87 117.35 116.19 116.01 115.82 110.45 109.99 105.49 82.27 82.25 82.22 77.40 77.08 76.76 46.97 33.37 24.01
F
F
N
F
F
O
7n
f1 (ppm)

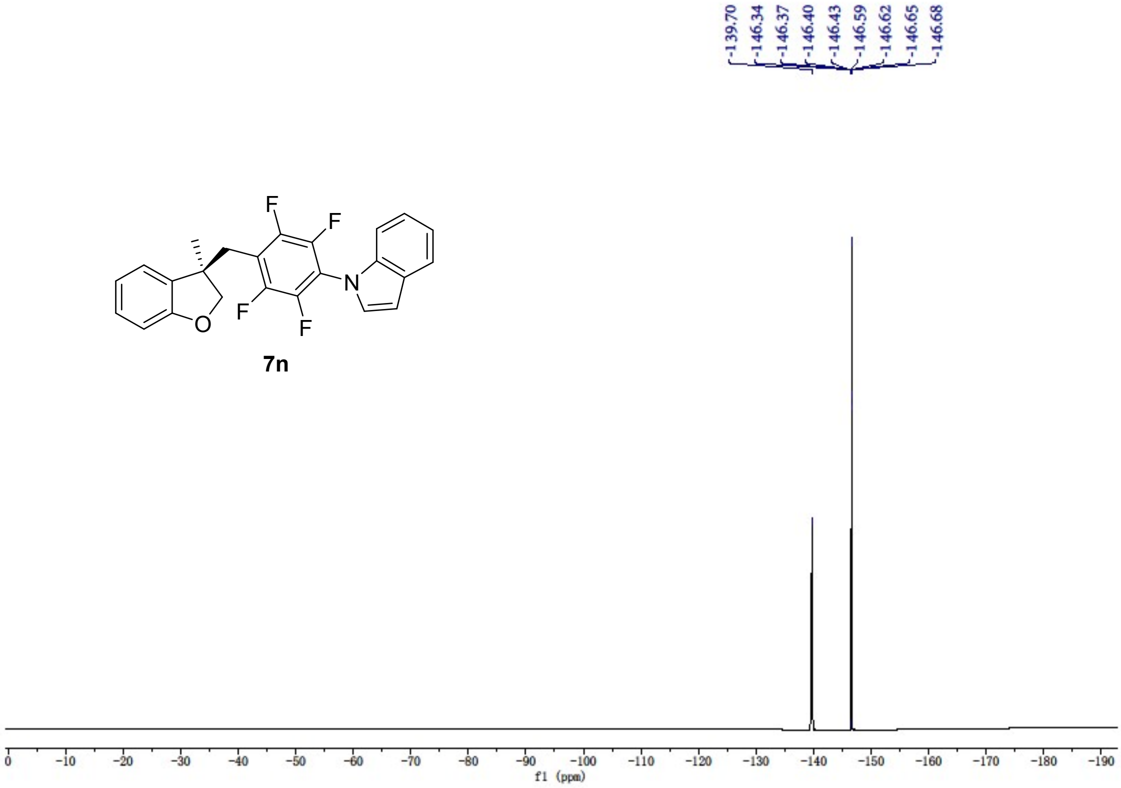

<Chromatogram>

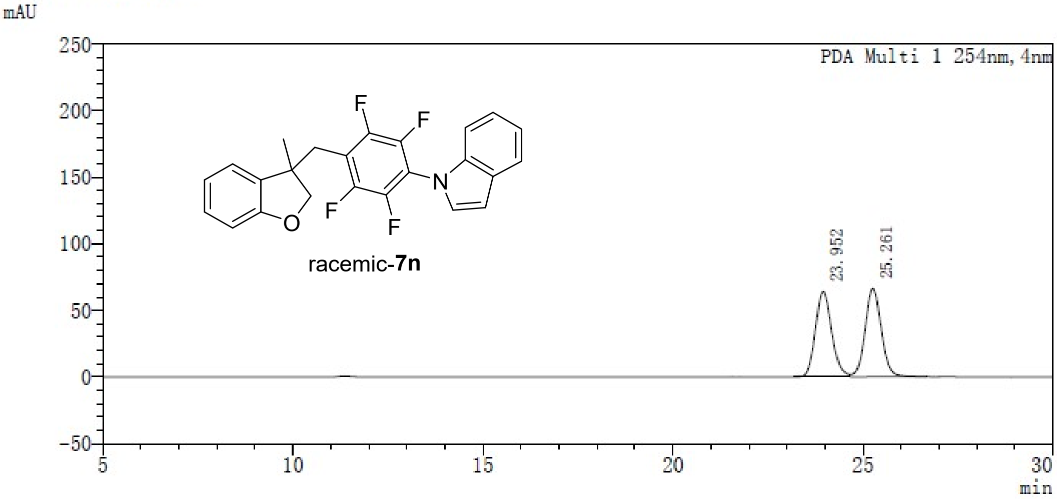

<Peak Table>

PDA Ch1 254nm

No.	Ret. Time (min)	Height	Height%	Area	Area%
1	23.952	64254	49.093	1921184	49.350
2	25.261	66629	50.907	1971763	50.650
Total		130883	100.000	3892946	100.000

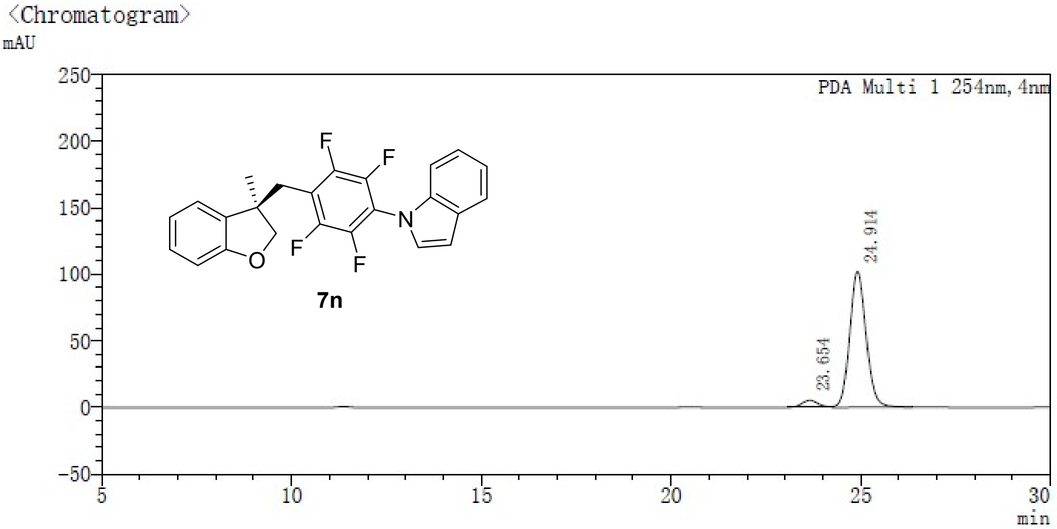

<Peak Table>

PDA Ch1 254nm

No.	Ret. Time (min)	Height	Height%	Area	Area%
1	23.654	5327	4.960	152929	4.731
2	24.914	102071	95.040	3079232	95.269
Total		107397	100.000	3232161	100.000

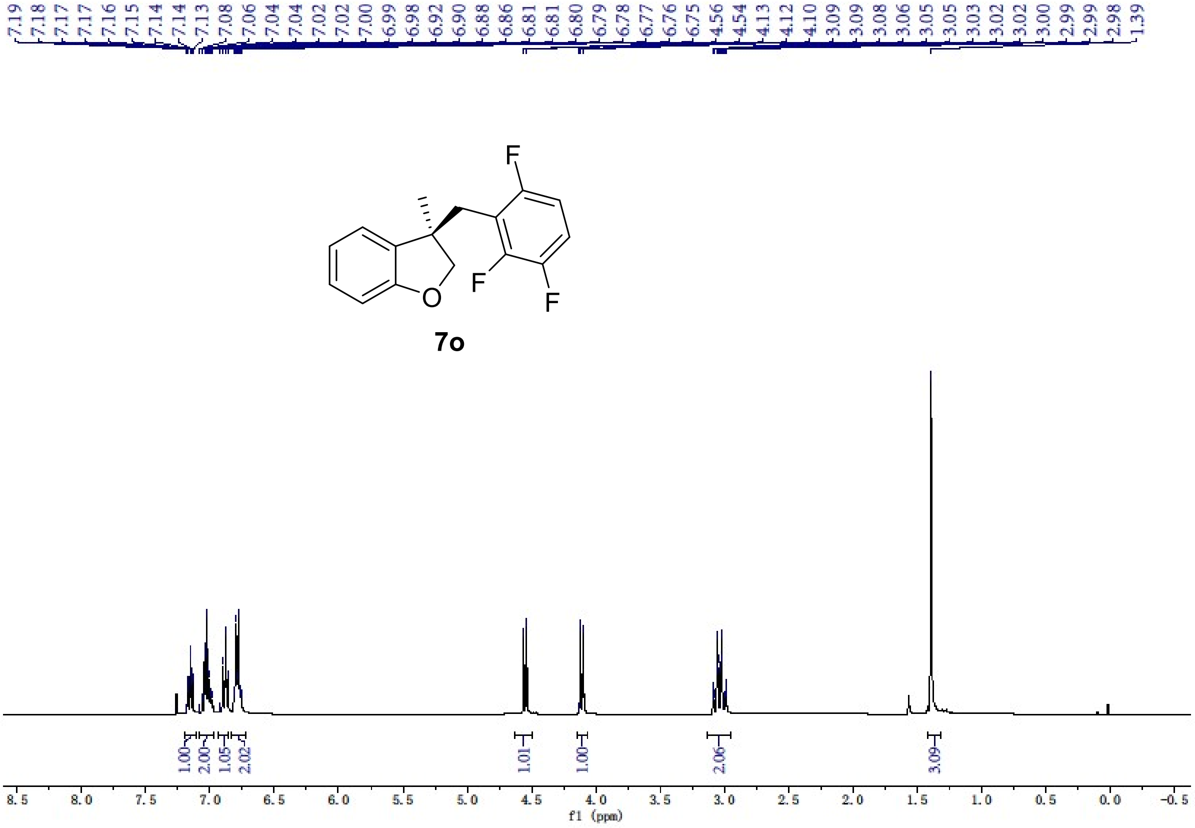

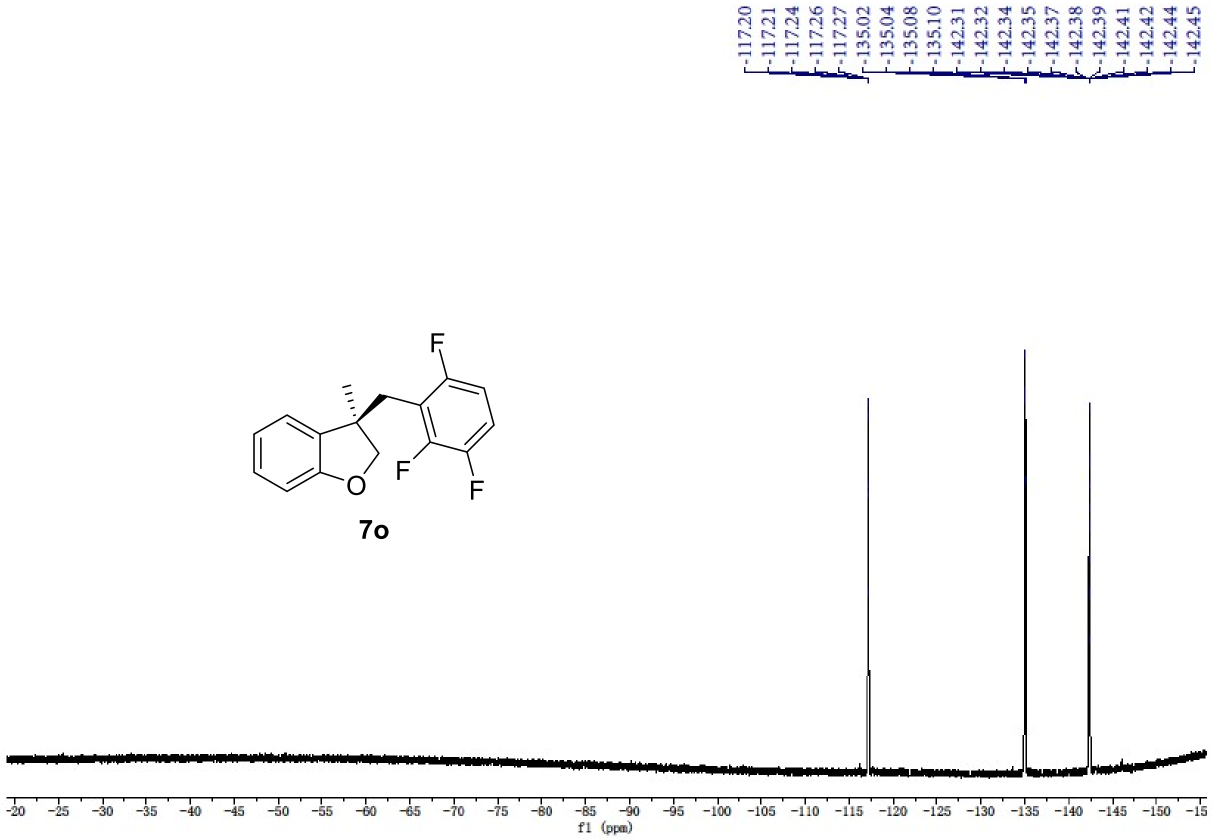
-117.20
-117.21
-117.24
-117.26
-117.27
-135.02
-135.04
-135.08
-135.10
-142.31
-142.32
-142.34
-142.35
-142.37
-142.38
-142.39
-142.41
-142.42
-142.44
-142.45
F
F
F
O
7o
f1 (ppm)

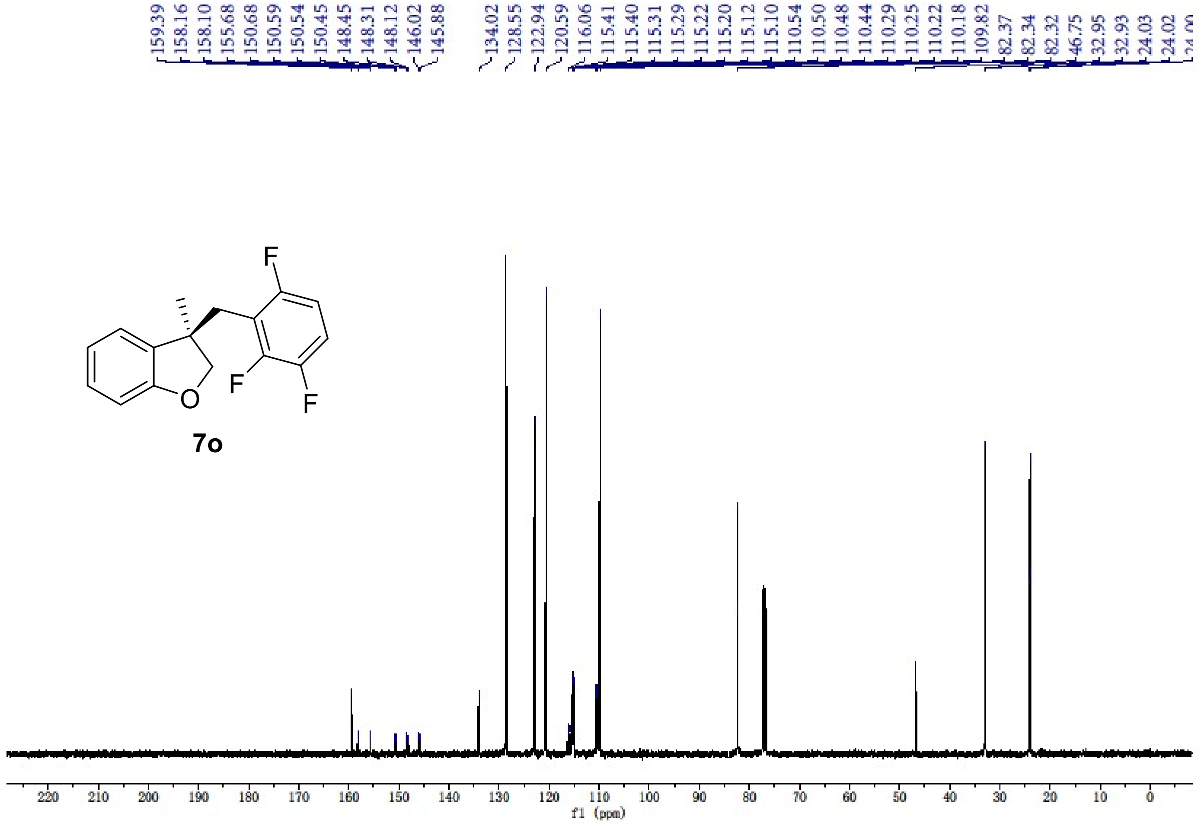
159.39
158.16
158.10
155.68
150.68
150.59
150.54
150.45
148.45
148.31
148.12
146.02
145.88
134.02
128.55
122.94
120.59
116.06
115.41
115.40
115.31
115.29
115.22
115.20
115.12
115.10
110.54
110.50
110.48
110.44
110.29
110.25
110.22
110.18
109.82
82.37
82.34
82.32
46.75
32.95
32.93
24.03
24.02
F
F
F
O
7o
f1 (ppm)

<Chromatogram>

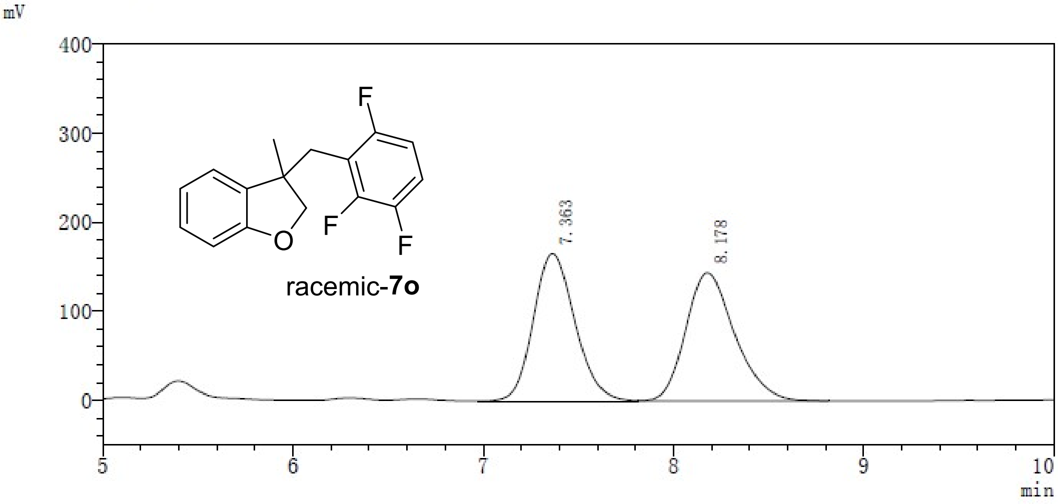

<Peak Table>

检测器A Ch2 210nm

No.	Ret. Time(min)	Height	Height%	Area	Area%
1	7.363	165694	53.493	2522916	49.450
2	8.178	144053	46.507	2579075	50.550
Total		309747	100.000	5101991	100.000

<Chromatogram>

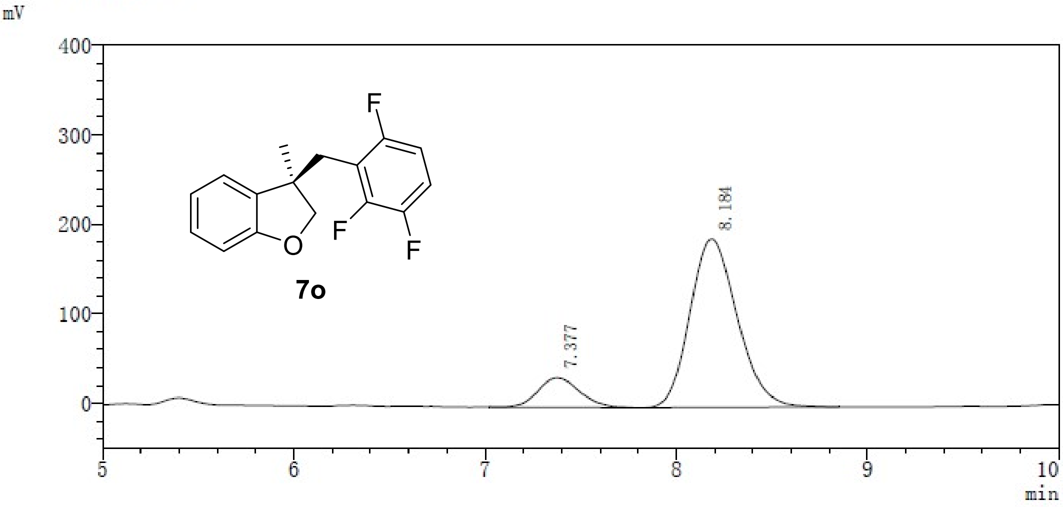

<Peak Table>

检测器A Ch2 210nm

No.	Ret. Time(min)	Height	Height%	Area	Area%
1	7.377	33122	14.965	507079	13.516
2	8.184	188206	85.035	3244711	86.484
Total		221328	100.000	3751790	100.000

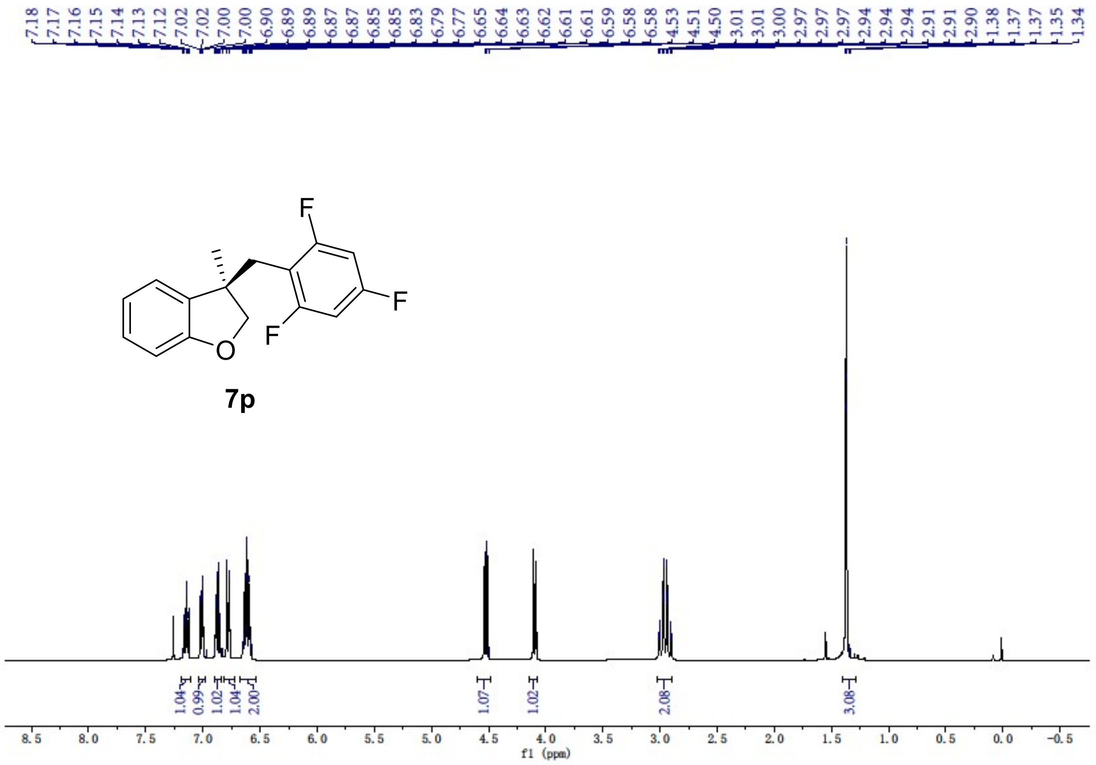
7.18 7.17 7.16 7.15 7.14 7.13 7.12 7.02 7.02 7.00 7.00 6.90 6.89 6.89 6.87 6.87 6.85 6.83 6.79 6.77 6.65 6.64 6.63 6.62 6.61 6.61 6.59 6.58 6.58 4.53 4.51 4.50 3.01 3.01 3.00 2.97 2.97 2.97 2.94 2.94 2.94 2.91 2.90 1.38 1.37 1.37 1.35 1.34
F
F
F
O
7p
1.04 0.99 1.02 1.04 2.00 1.07 1.02 2.08 3.08
8.5 8.0 7.5 7.0 6.5 6.0 5.5 5.0 4.5 4.0 3.5 3.0 2.5 2.0 1.5 1.0 0.5 0.0 −0.5
f1 (ppm)

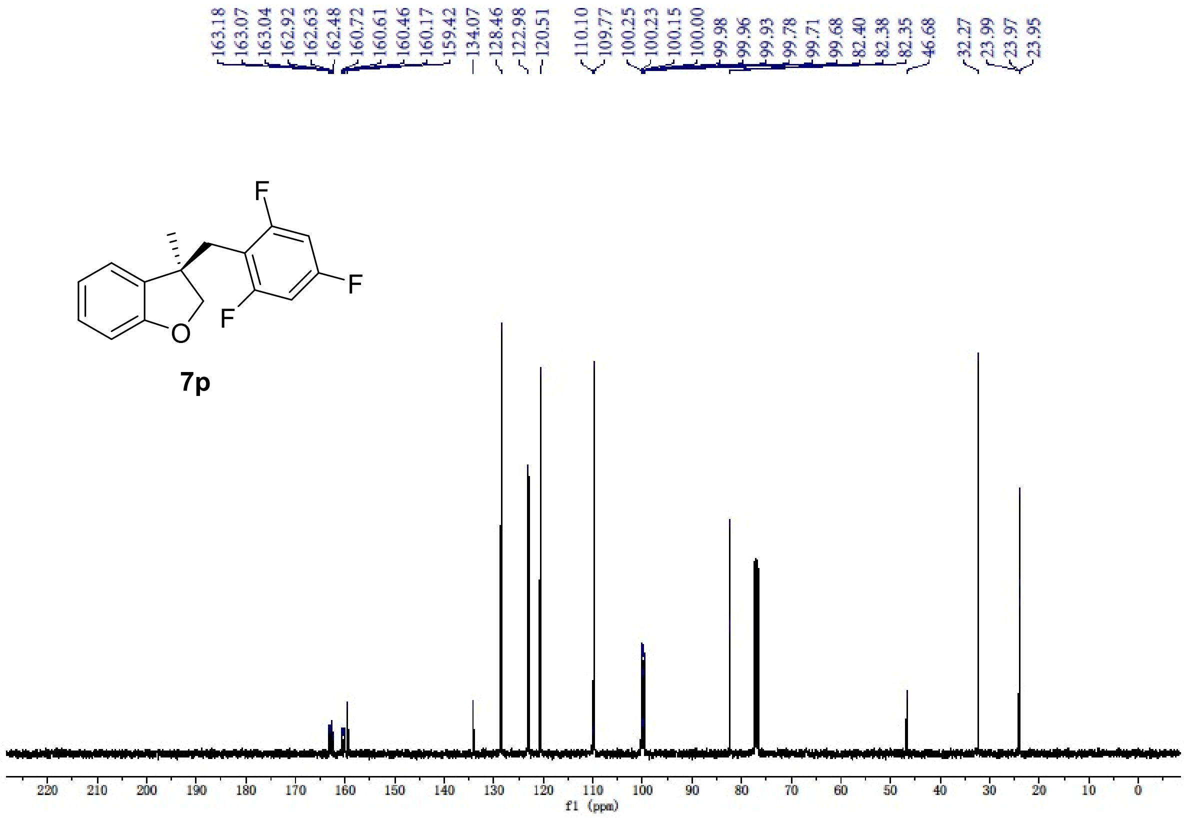
163.18 163.07 163.04 162.92 162.63 162.48 160.72 160.61 160.46 160.17 159.42 134.07 128.46 122.98 120.51 110.10 109.77 100.25 100.23 100.15 100.00 99.98 99.96 99.93 99.78 99.71 99.68 82.40 82.38 82.35 46.68 32.27 23.99 23.97 23.95
F
F
F
O
7p
220 210 200 190 180 170 160 150 140 130 120 110 100 90 80 70 60 50 40 30 20 10 0
f1 (ppm)

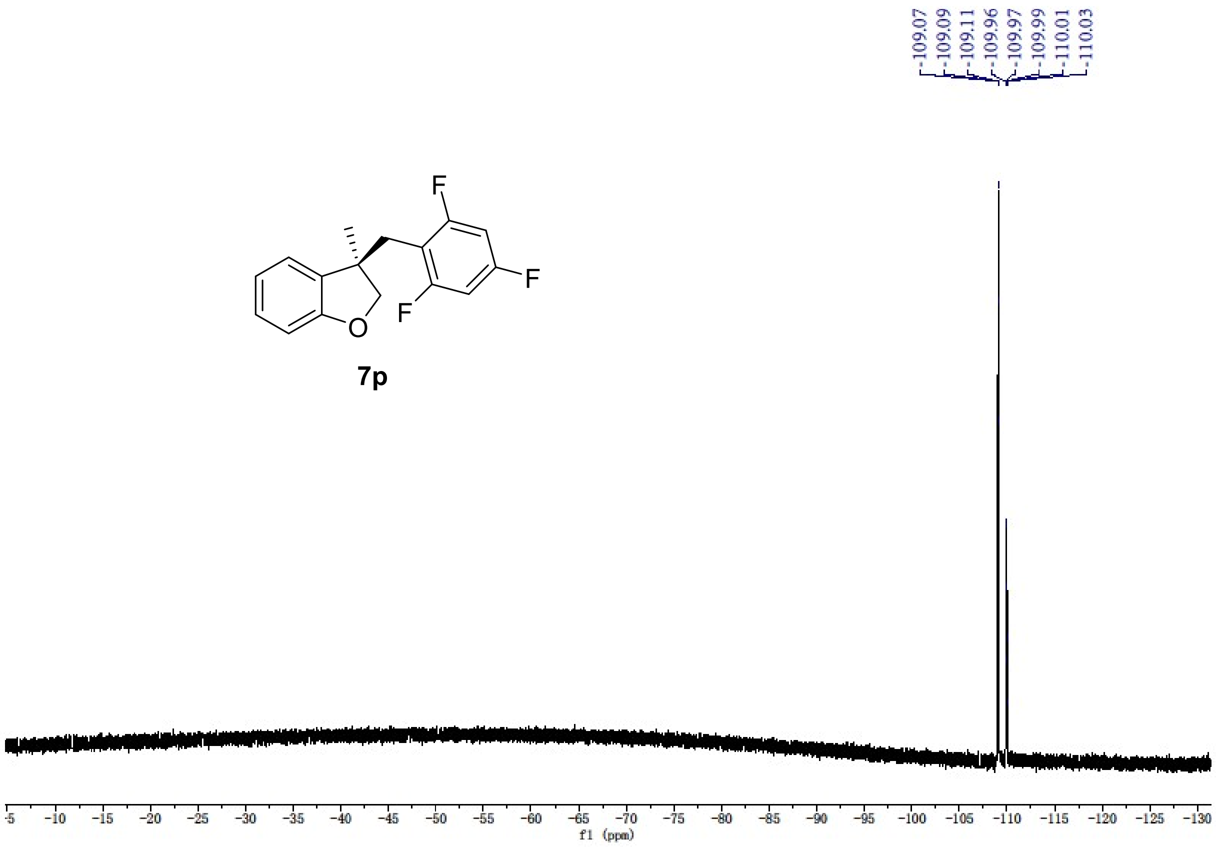

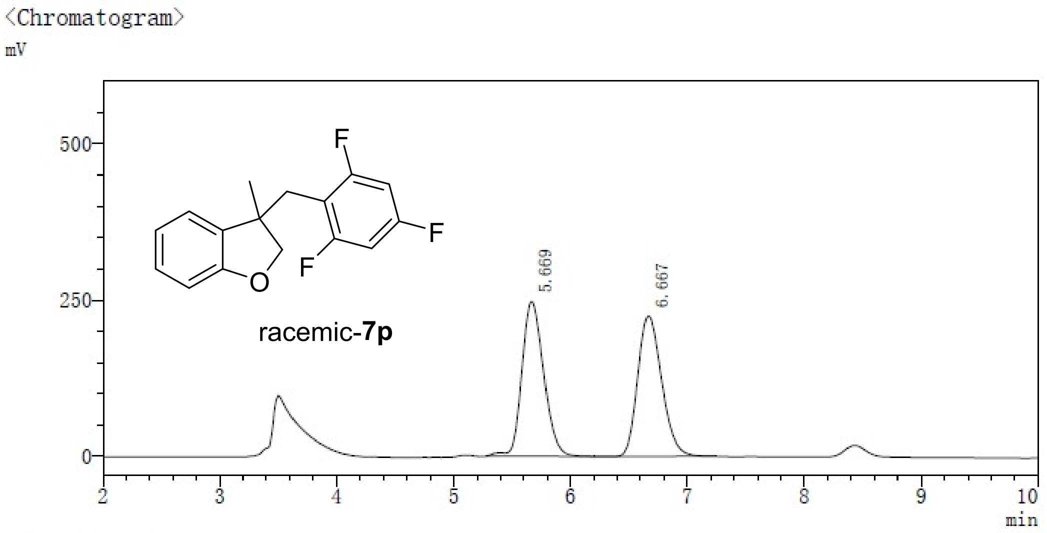

<Peak Table>

检测器A Ch2 210nm

No.	Ret. Time(min)	Height	Height%	Area	Area%
1	5.669	246979	52.355	3216619	50.267
2	6.667	224764	47.645	3182459	49.733
Total		471744	100.000	6399078	100.000

<Chromatogram>

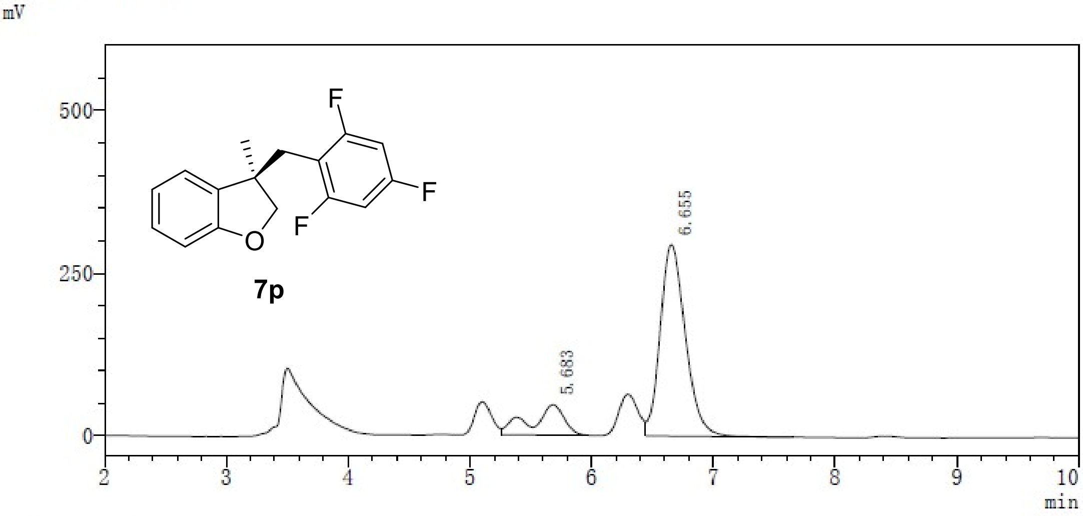

<Peak Table>

检测器A Ch2 210nm

No.	Ret. Time (min)	Height	Height%	Area	Area%
1	5.683	46284	13.615	863389	16.922
2	6.655	293662	86.385	4238701	83.078
Total		339947	100.000	5102090	100.000

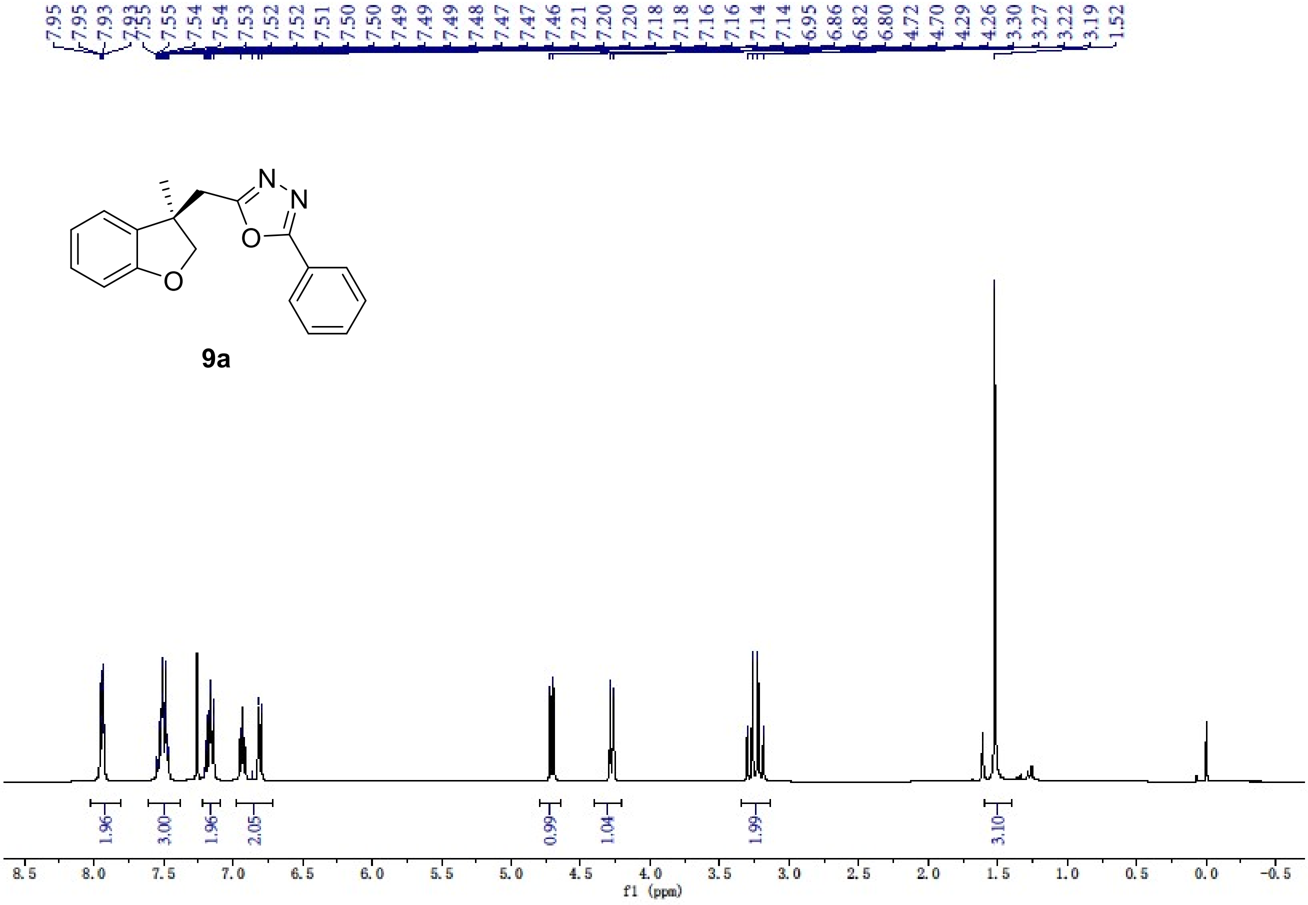

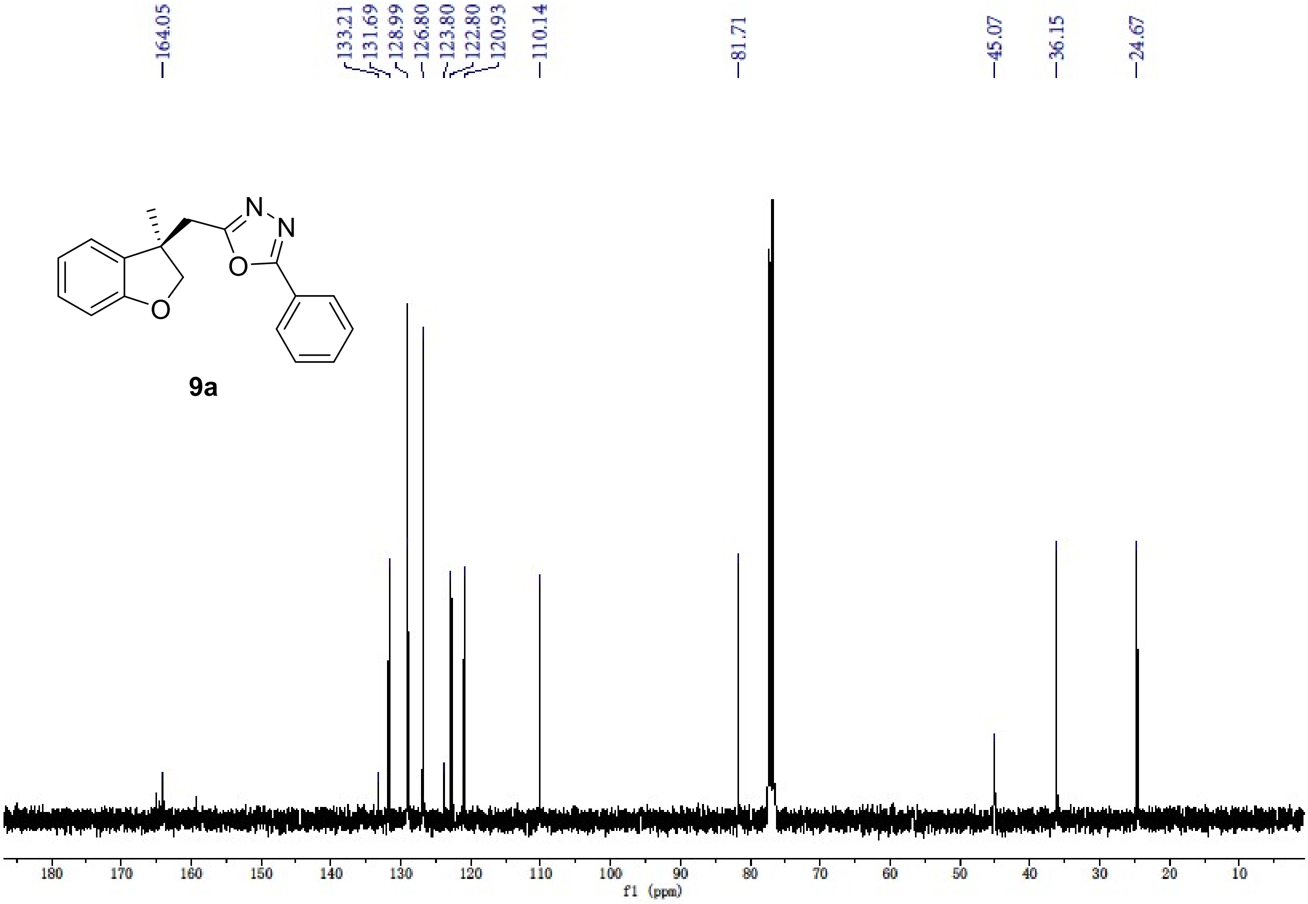

<Chromatogram>

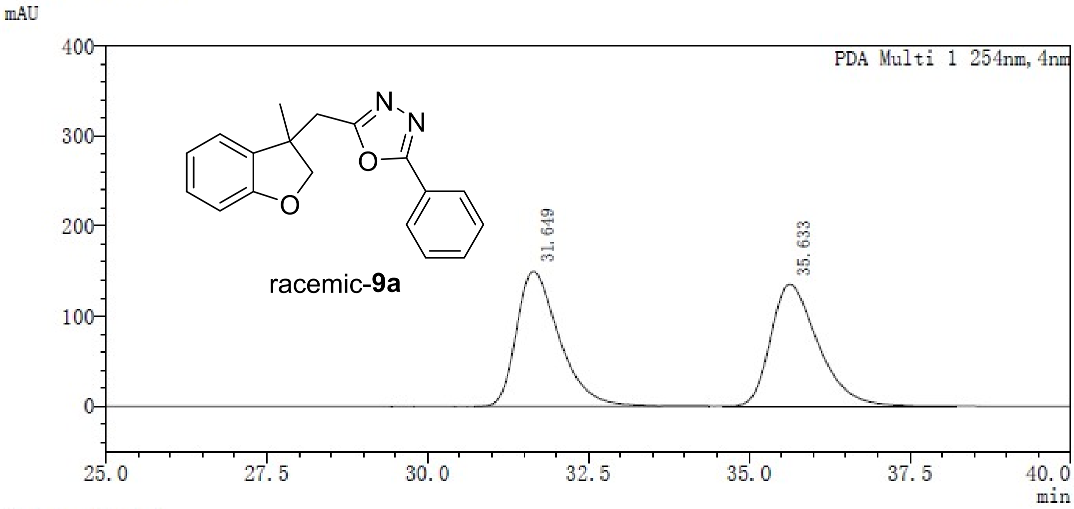

<Peak Table>

PDA Ch1 254nm

No.	Ret. Time(min)	Height	Height%	Area	Area%
1	31.649	149627	52.565	6943698	50.028
2	35.633	135023	47.435	6935901	49.972
Total		284650	100.000	13879599	100.000

<Chromatogram>

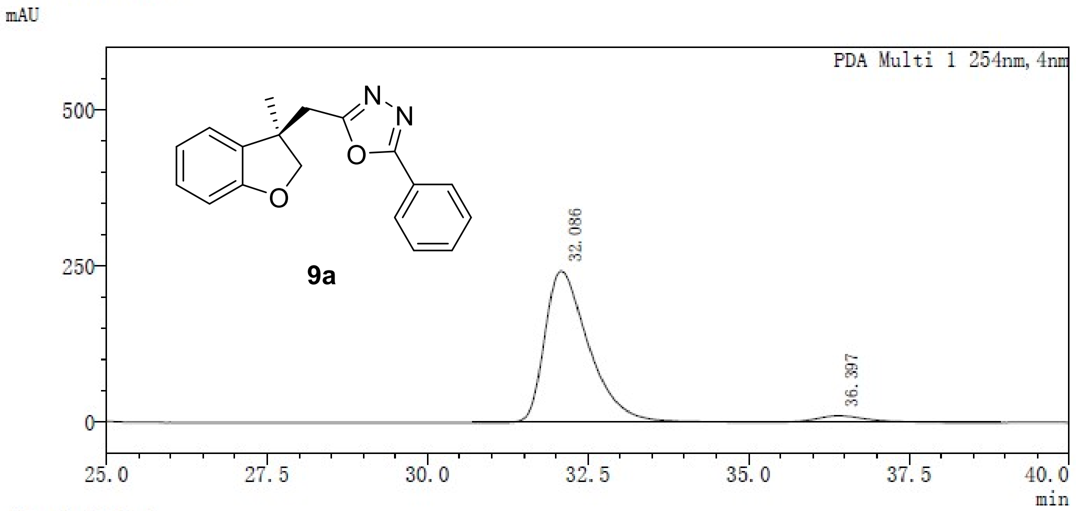

<Peak Table>

PDA Ch1 254nm

No.	Ret.Time(min)	Height	Height%	Area	Area%
1	32.086	242709	95.821	11646596	95.259
2	36.397	10586	4.179	579658	4.741
Total		253295	100.000	12226255	100.000

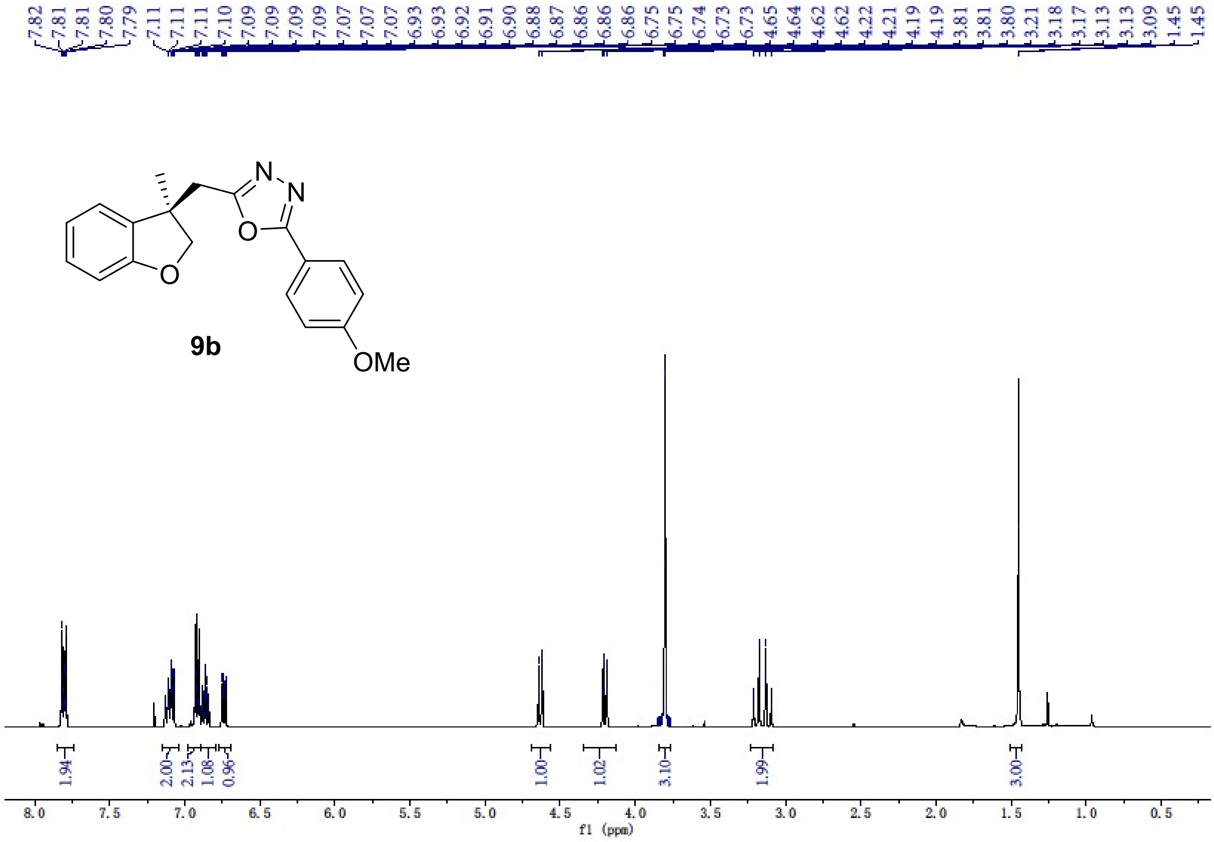

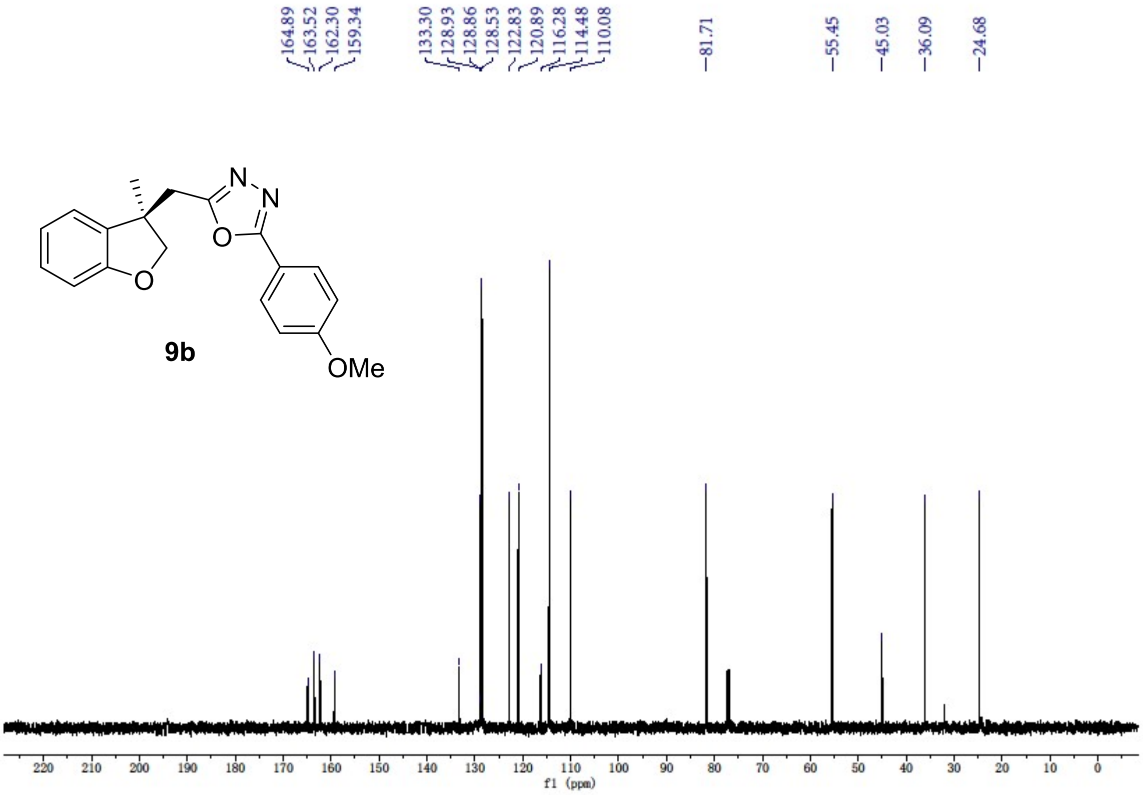

〈Chromatogram〉

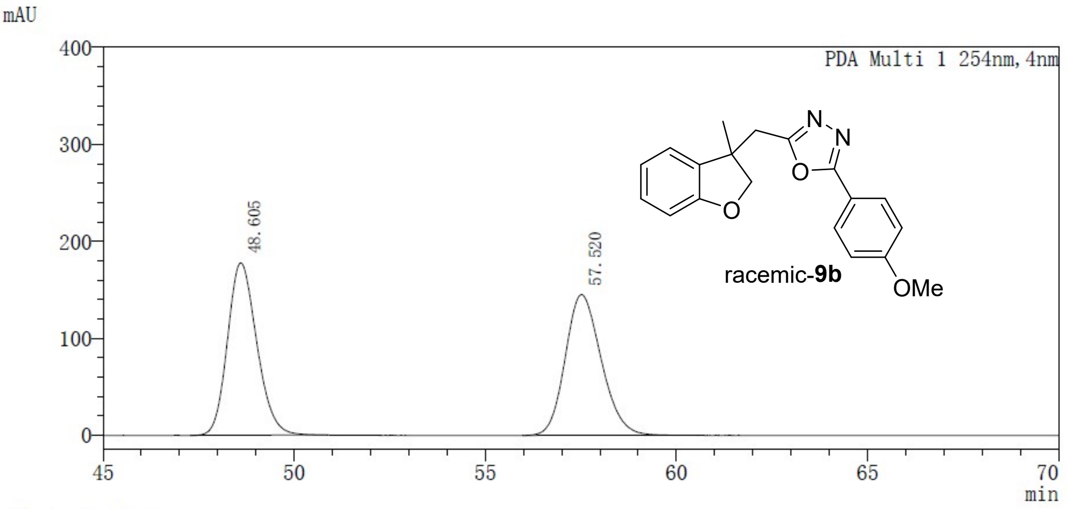

〈Peak Table〉

PDA Ch1 254nm

No.	Ret. Time (min)	Height	Height%	Area	Area%
1	48.605	177611	55.036	9495713	49.977
2	57.520	145108	44.964	9504556	50.023
Total		322719	100.000	19000269	100.000

<Chromatogram>

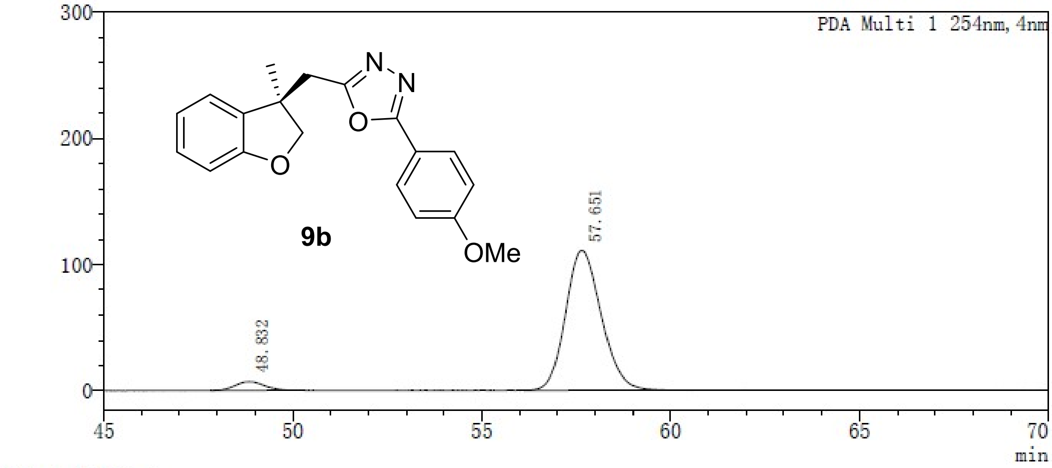

<Peak Table>

PDA Ch1 254nm

No.	Ret. Time(min)	Height	Height%	Area	Area%
1	48.832	7150	6.080	386336	5.047
2	57.651	110454	93.920	7268720	94.953
Total		117604	100.000	7655055	100.000

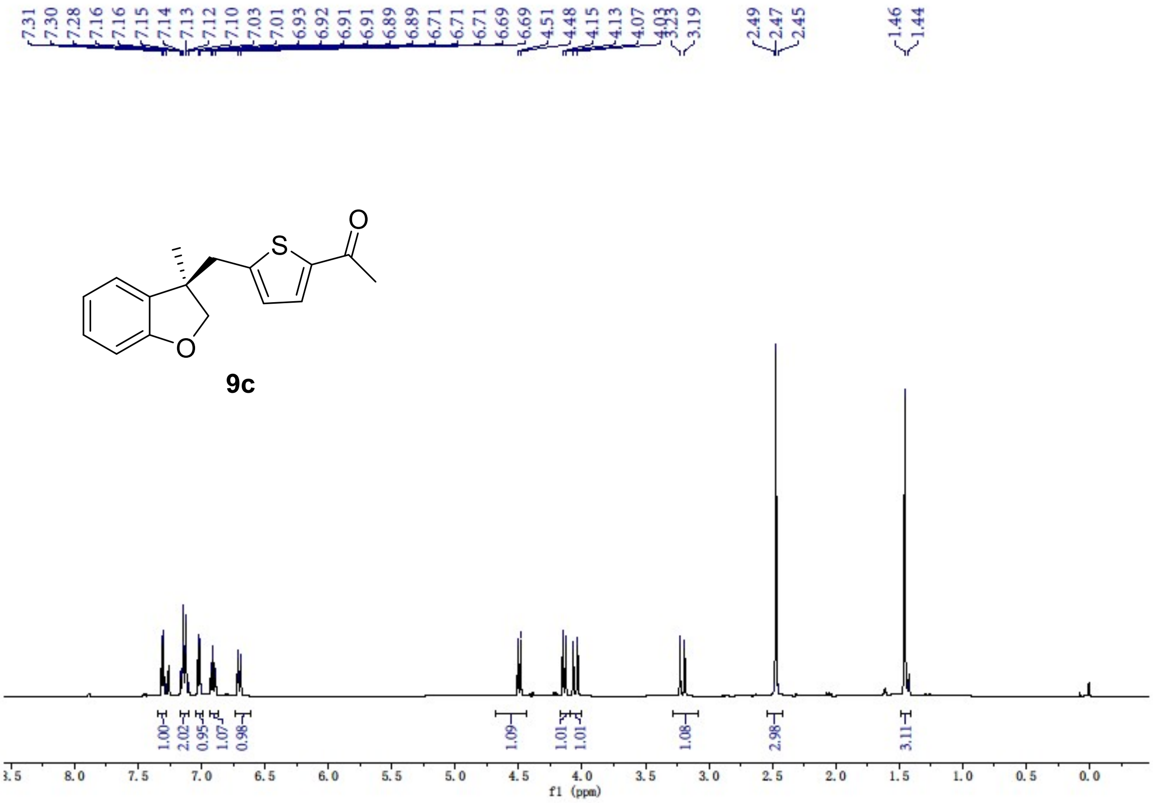

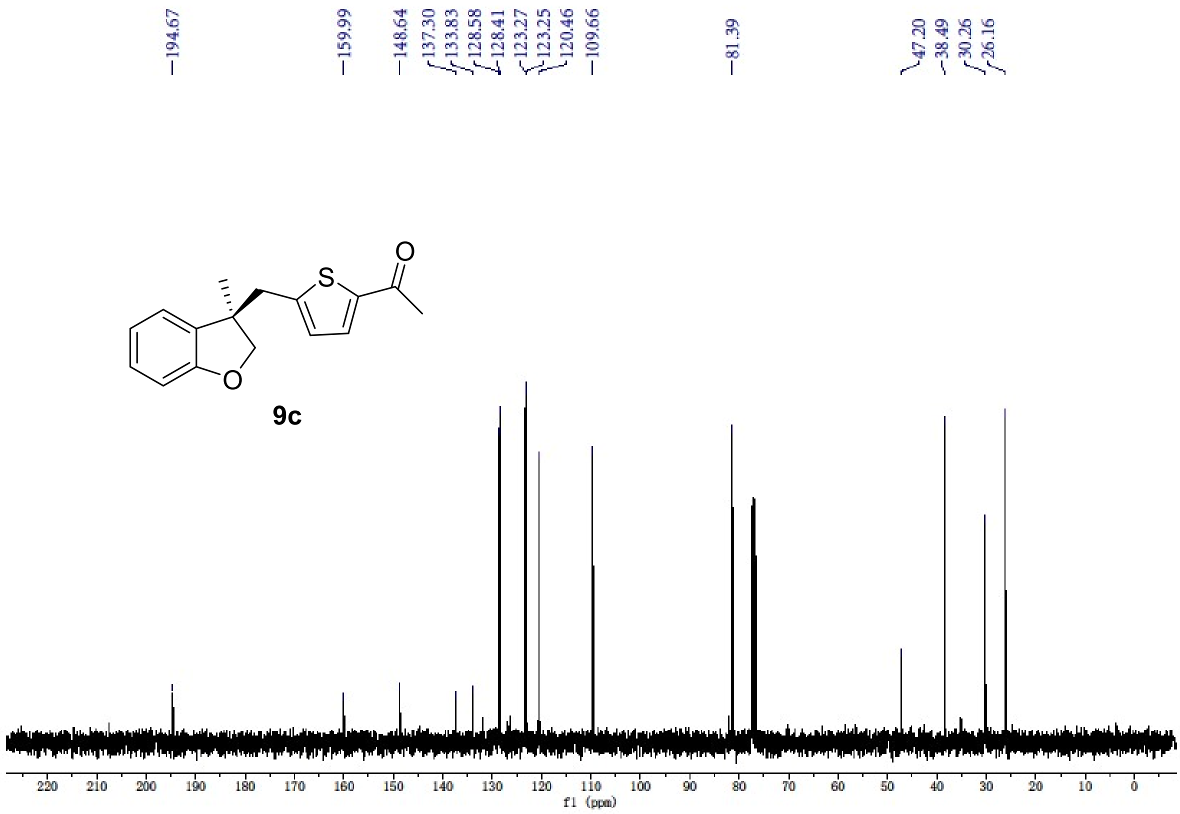

<Chromatogram>

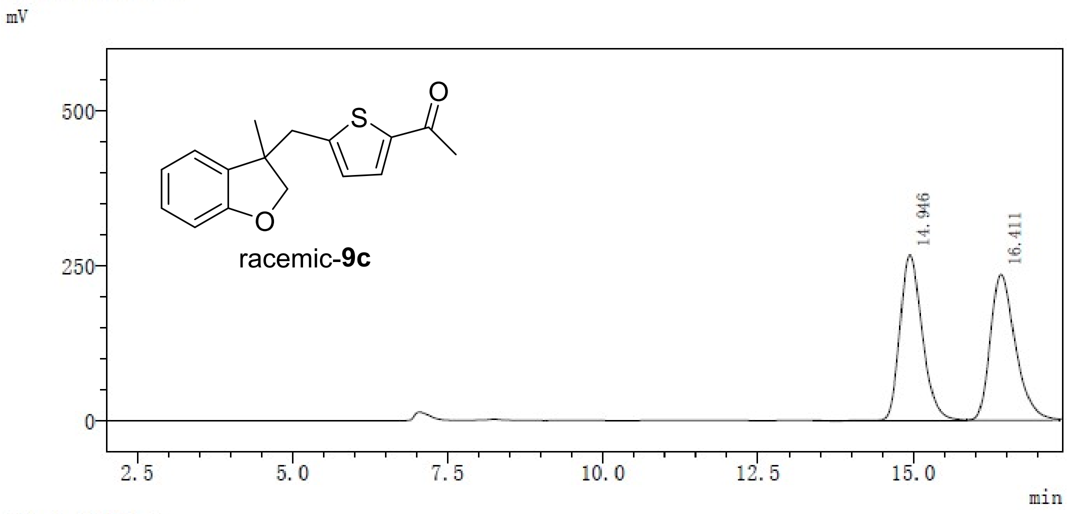

<Peak Table>

检测器A Ch1 254nm

No.	Ret. Time(min)	Height	Height%	Area	Area%
1	14.946	266947	53.074	6661053	49.950
2	16.411	236021	46.926	6674519	50.050
Total		502968	100.000	13335572	100.000

<Chromatogram>

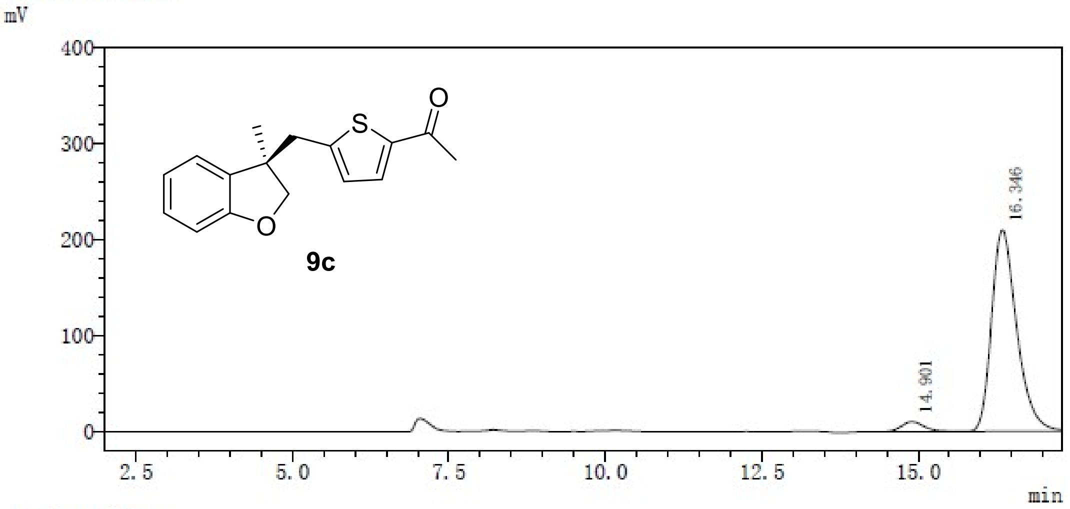

<Peak Table>

检测器A Ch1 254nm

No.	Ret. Time (min)	Height	Height%	Area	Area%
1	14.901	10381	4.706	259941	4.214
2	16.346	210198	95.294	5908828	95.786
Total		220580	100.000	6168769	100.000

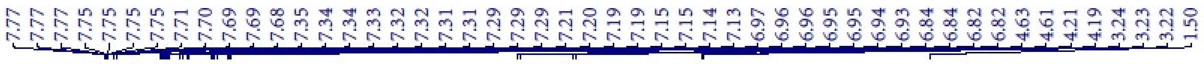

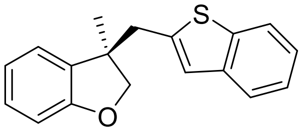

9d

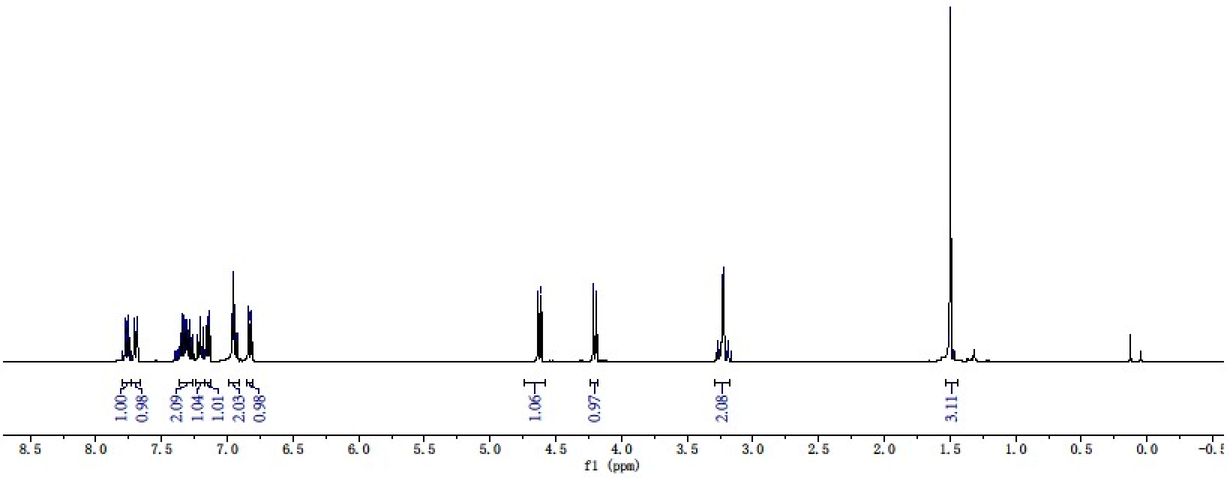

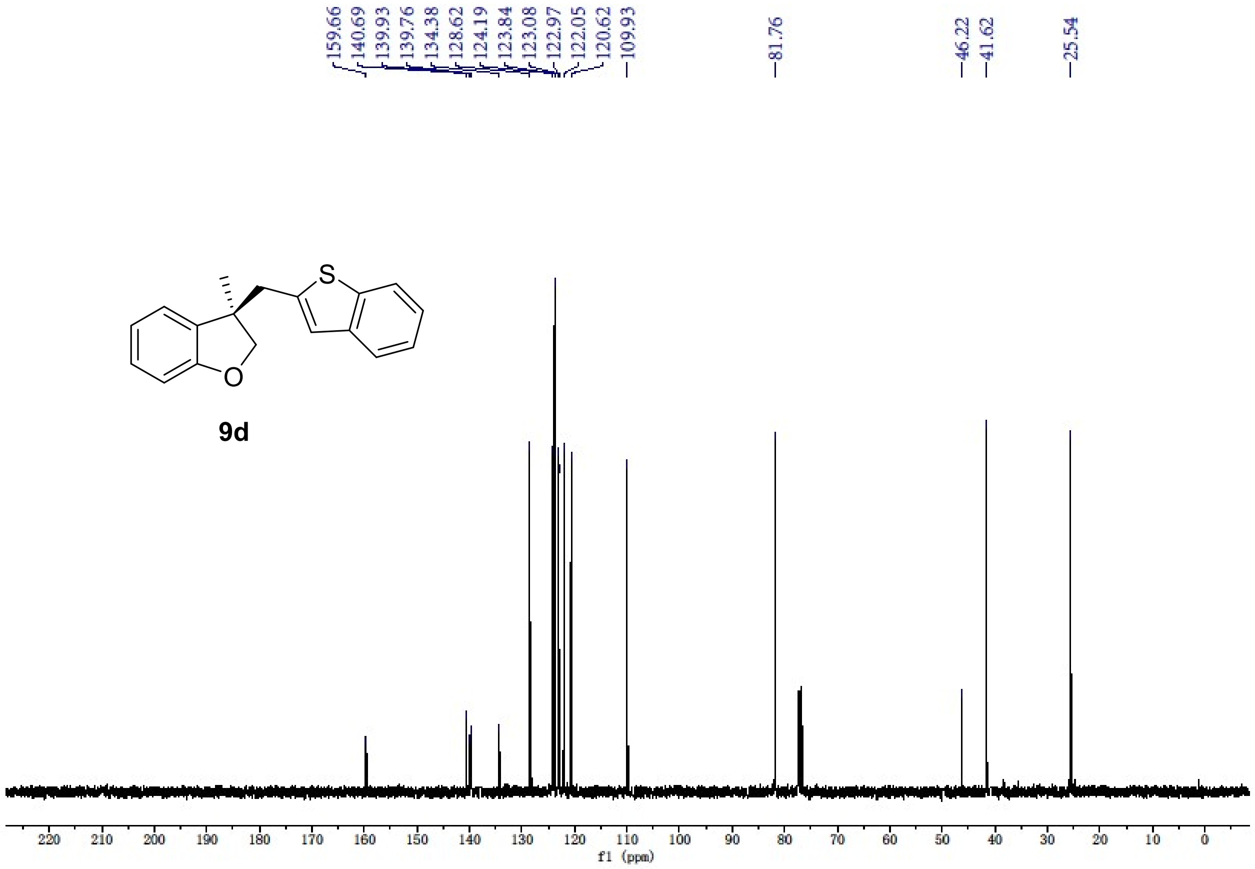

<Chromatogram>

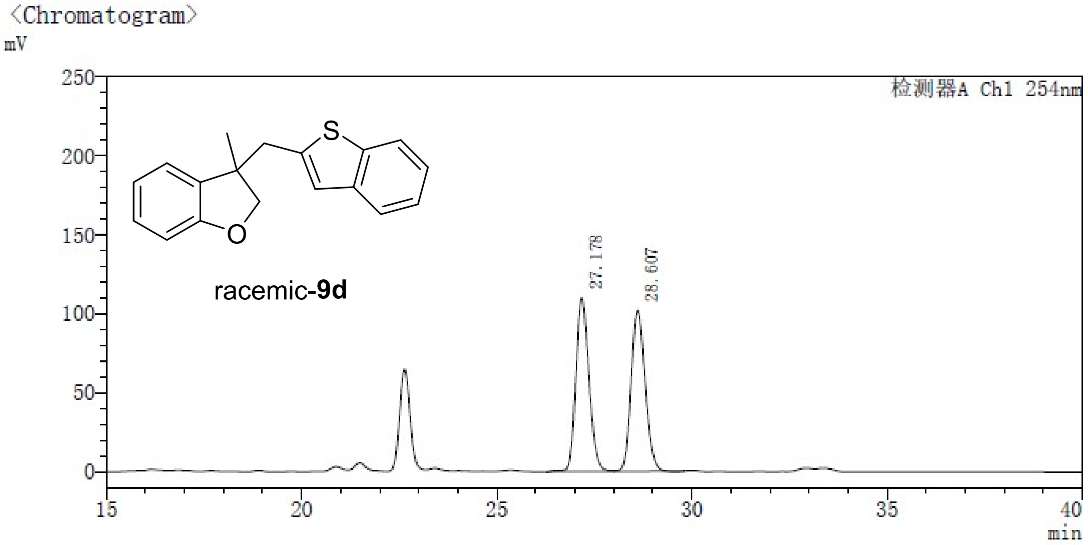

<Peak Table>

检测器A Ch1 254nm

No.	Ret.Time(min)	Height	Height%	Area	Area%
1	27.178	109719	51.915	2574957	50.390
2	28.607	101625	48.085	2535127	49.610
Total		211344	100.000	5110084	100.000

<Chromatogram>

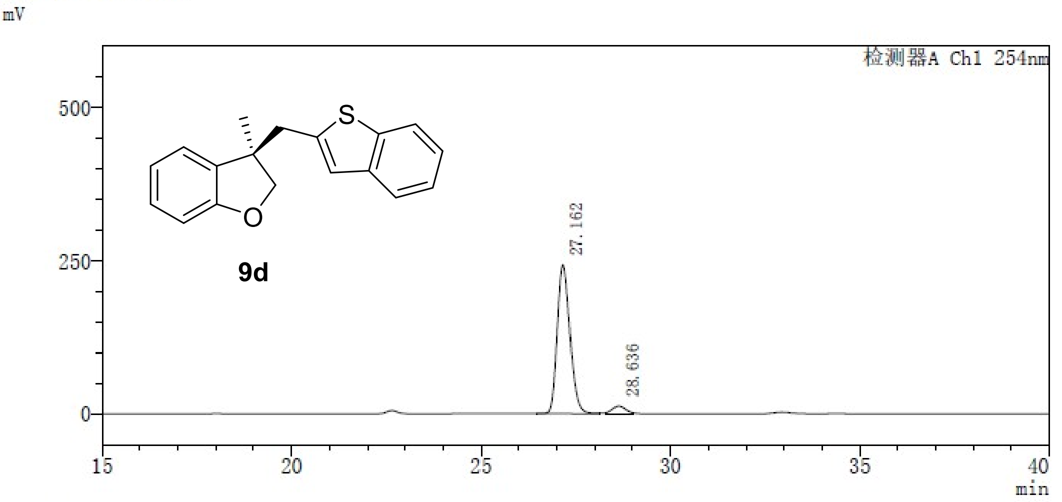

<Peak Table>

检测器A Ch1 254nm

No.	Ret. Time (min)	Height	Height%	Area	Area%
1	27.162	242305	94.994	5731983	94.785
2	28.636	12770	5.006	315345	5.215
Total		255074	100.000	6047328	100.000

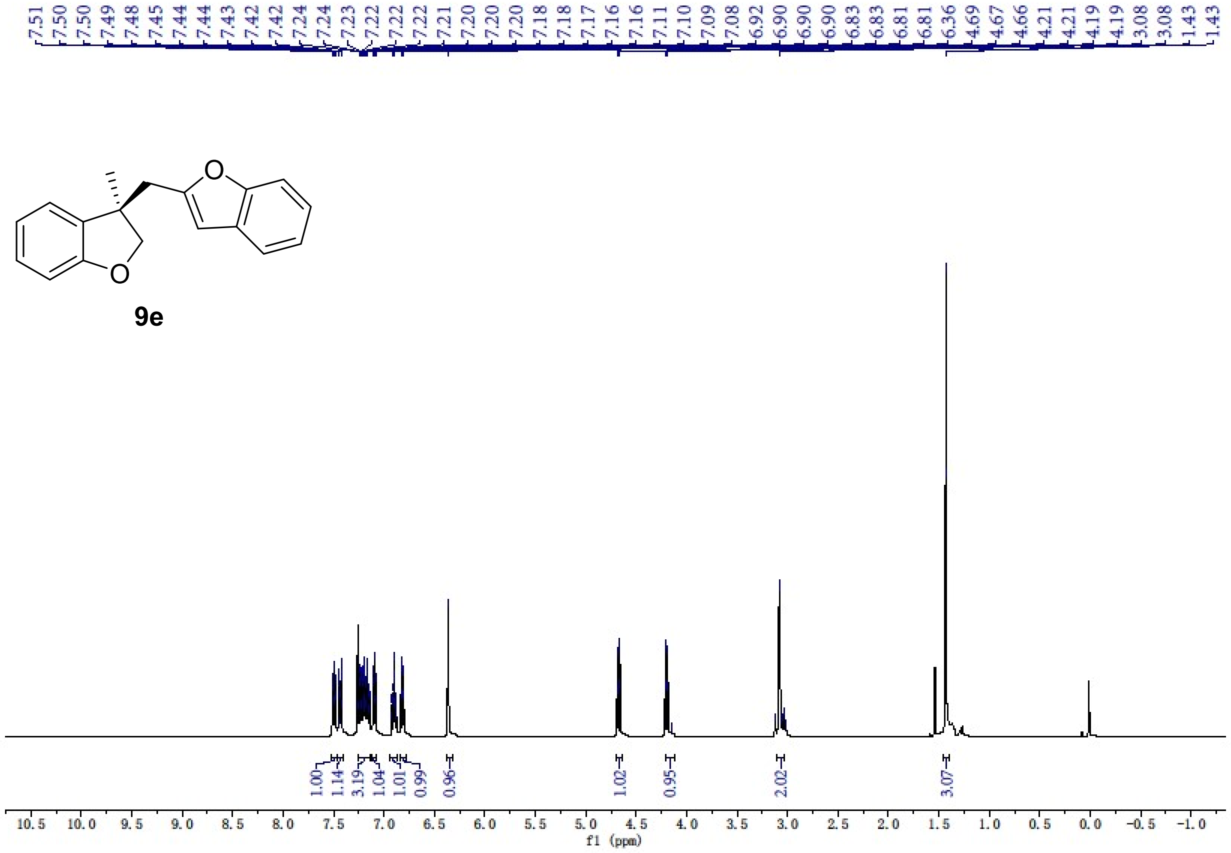

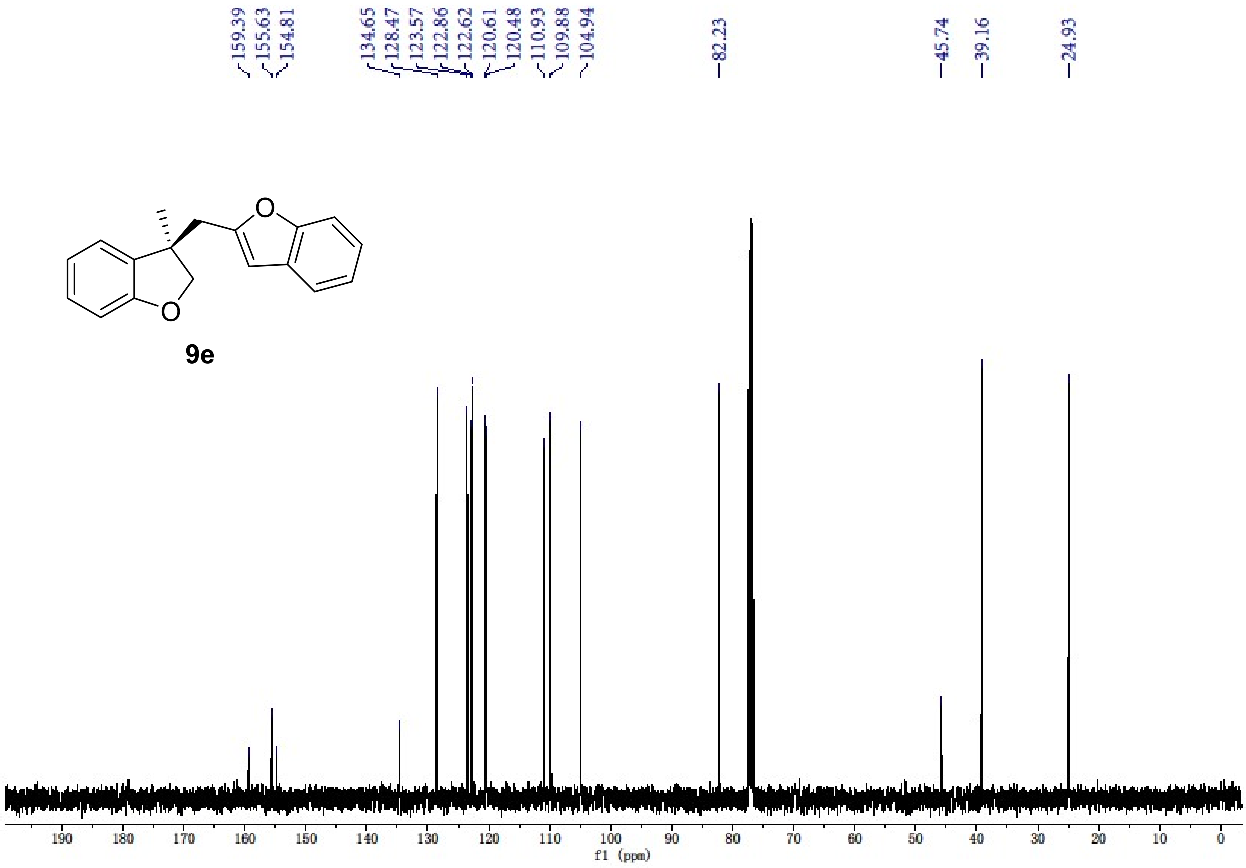

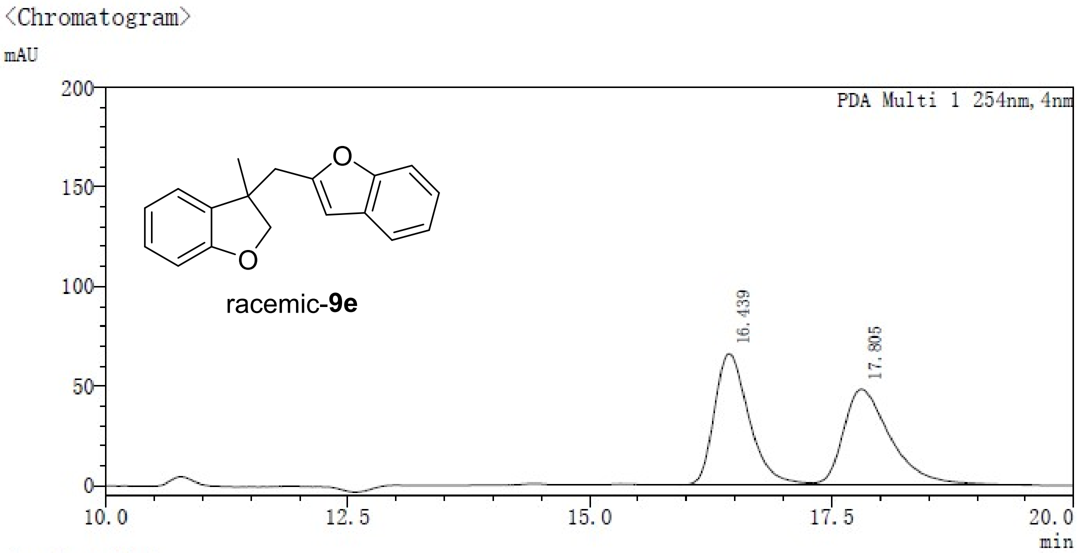

<Peak Table>

PDA Ch1 254nm

No.	Ret. Time(min)	Height	Height%	Area	Area%
1	16.439	65885	57.798	1640250	50.098
2	17.805	48107	42.202	1633860	49.902
Total		113992	100.000	3274110	100.000

<Chromatogram>

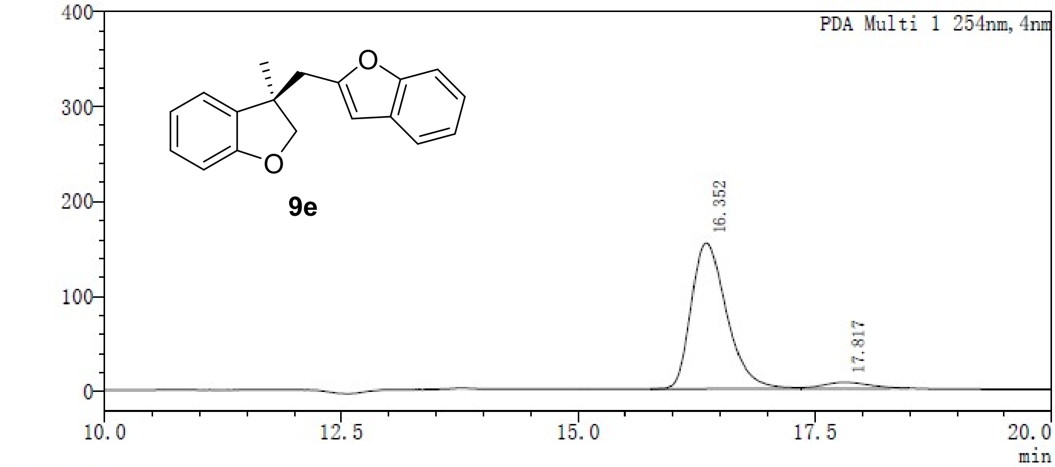

<Peak Table>

PDA Ch1 254nm

No.	Ret. Time(min)	Height	Height%	Area	Area%
1	16.352	153625	95.991	4002564	94.987
2	17.817	6415	4.009	211221	5.013
Total		160040	100.000	4213785	100.000

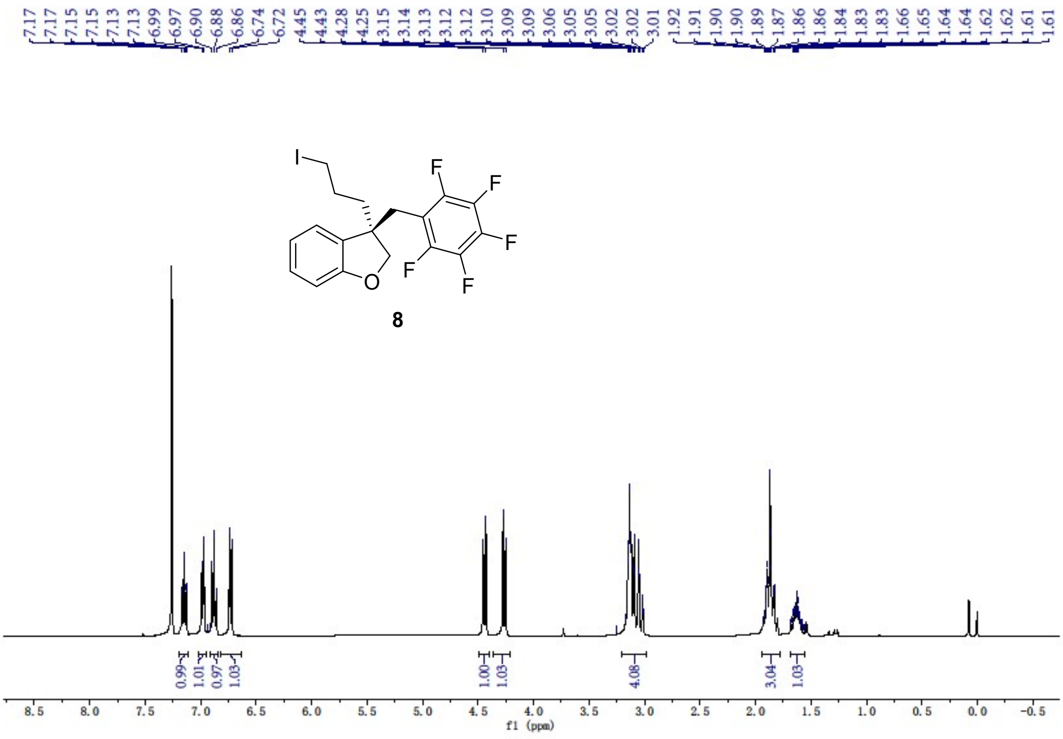

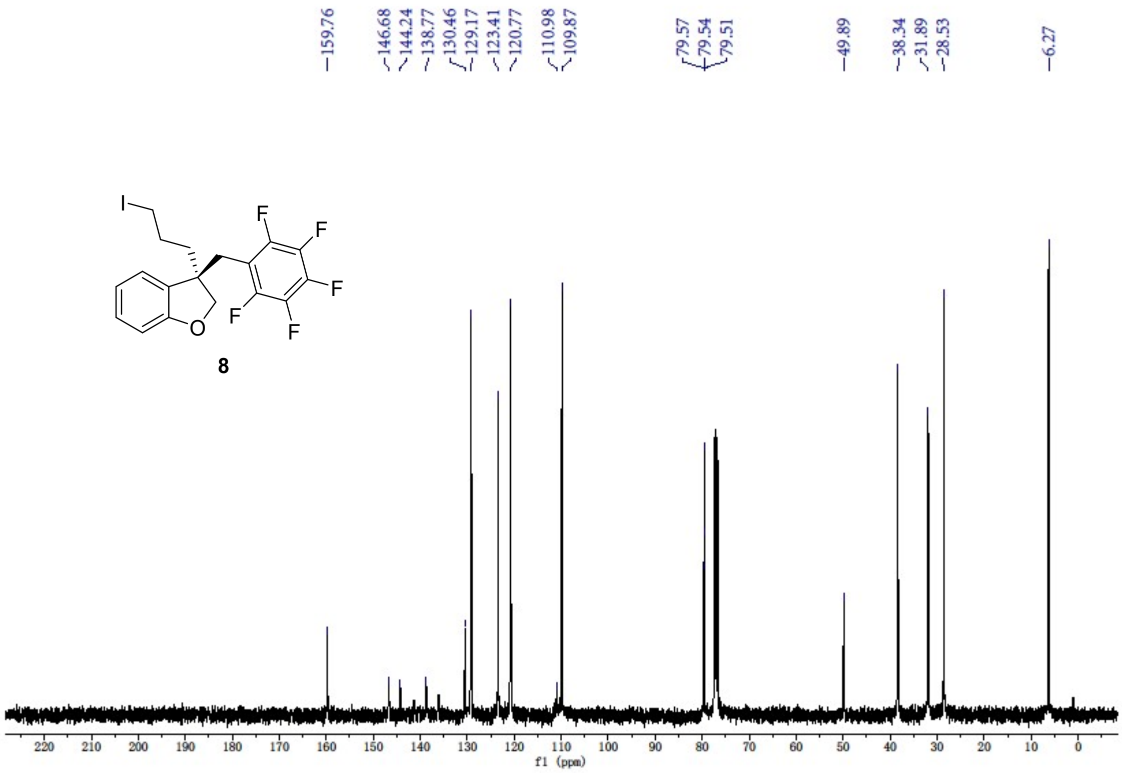
159.76
146.68
144.24
138.77
130.46
129.17
123.41
120.77
110.98
109.87
79.57
79.54
79.51
49.89
38.34
31.89
28.53
6.27
F
I
O
f1 (ppm)

8

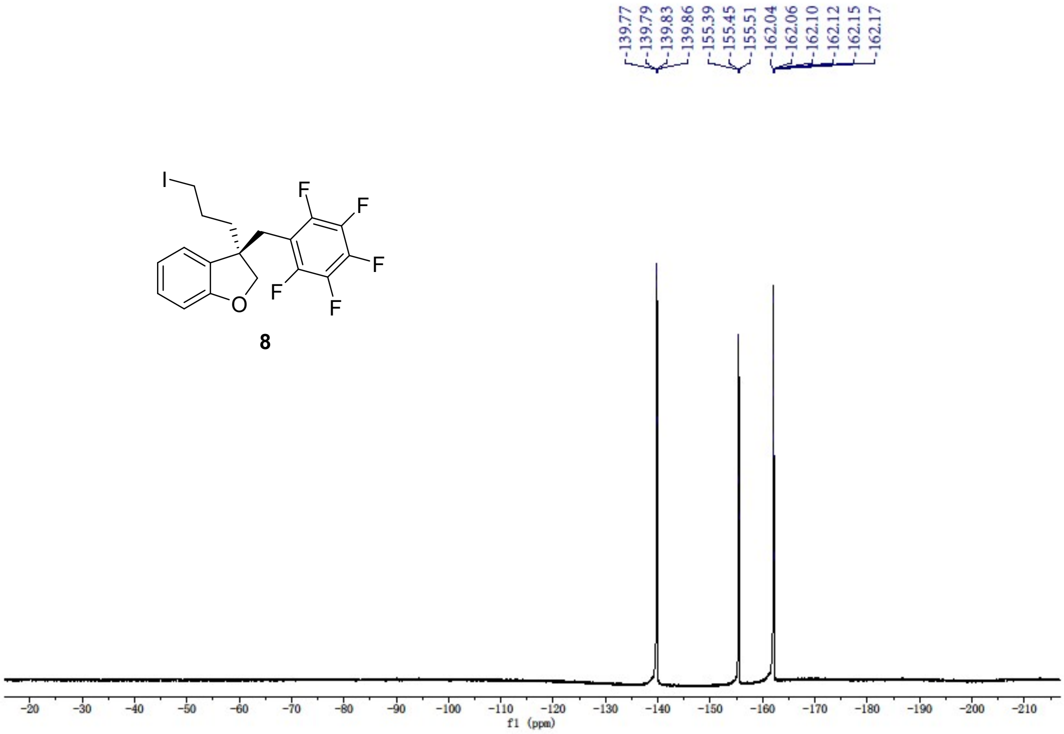
-139.77
-139.79
-139.83
-139.86
-155.39
-155.45
-155.51
-162.04
-162.06
-162.10
-162.12
-162.15
-162.17
F
I
O
f1 (ppm)

8

〈Chromatogram〉

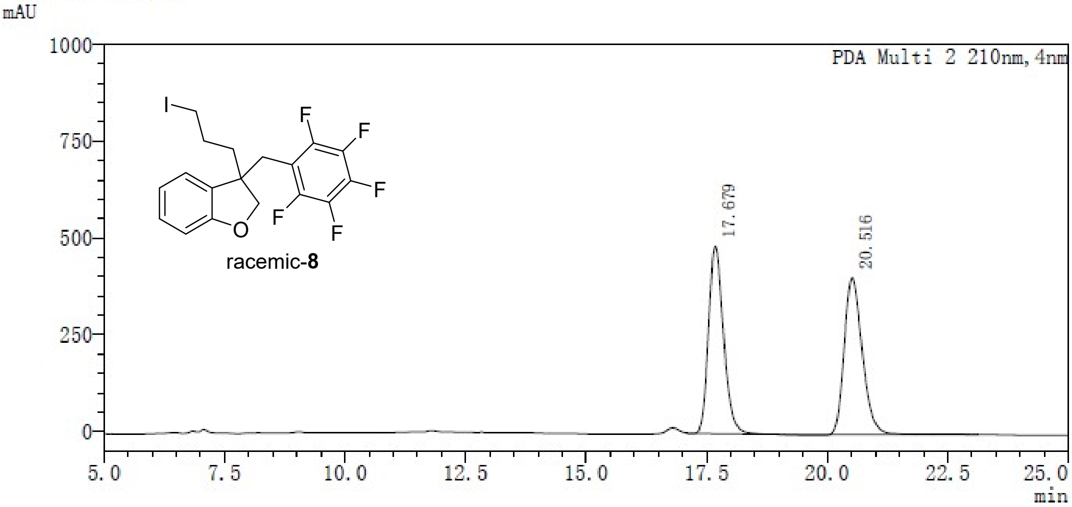

〈Peak Table〉

PDA Ch2 210nm

No.	Ret. Time (min)	Height	Height%	Area	Area%
1	17.679	484020	54.472	10520402	50.045
2	20.516	404540	45.528	10501433	49.955
Total		888560	100.000	21021835	100.000

〈Chromatogram〉

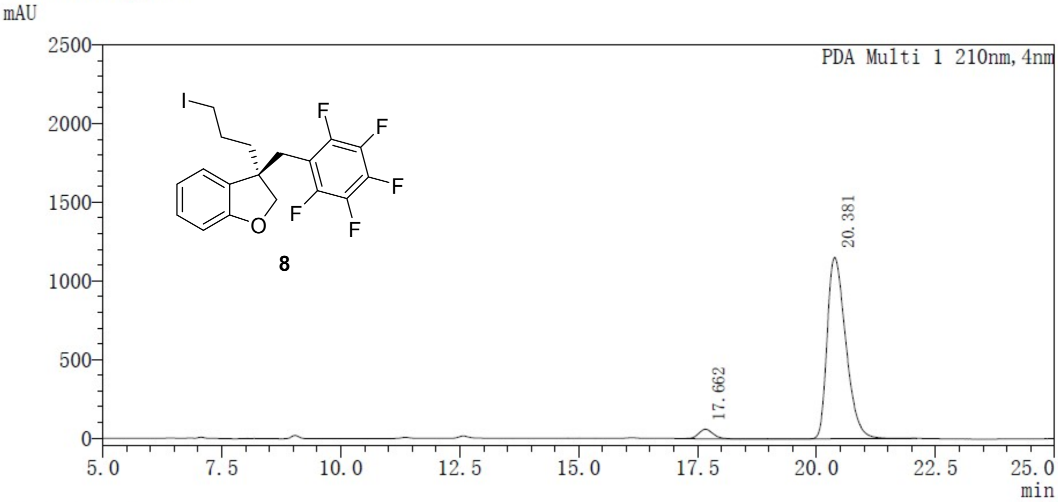

〈Peak Table〉

PDA Ch1 210nm

No.	Ret. Time (min)	Height	Height%	Area	Area%
1	17.662	59841	4.926	1239125	3.782
2	20.381	1155045	95.074	31524318	96.218
Total		1214886	100.000	32763443	100.000

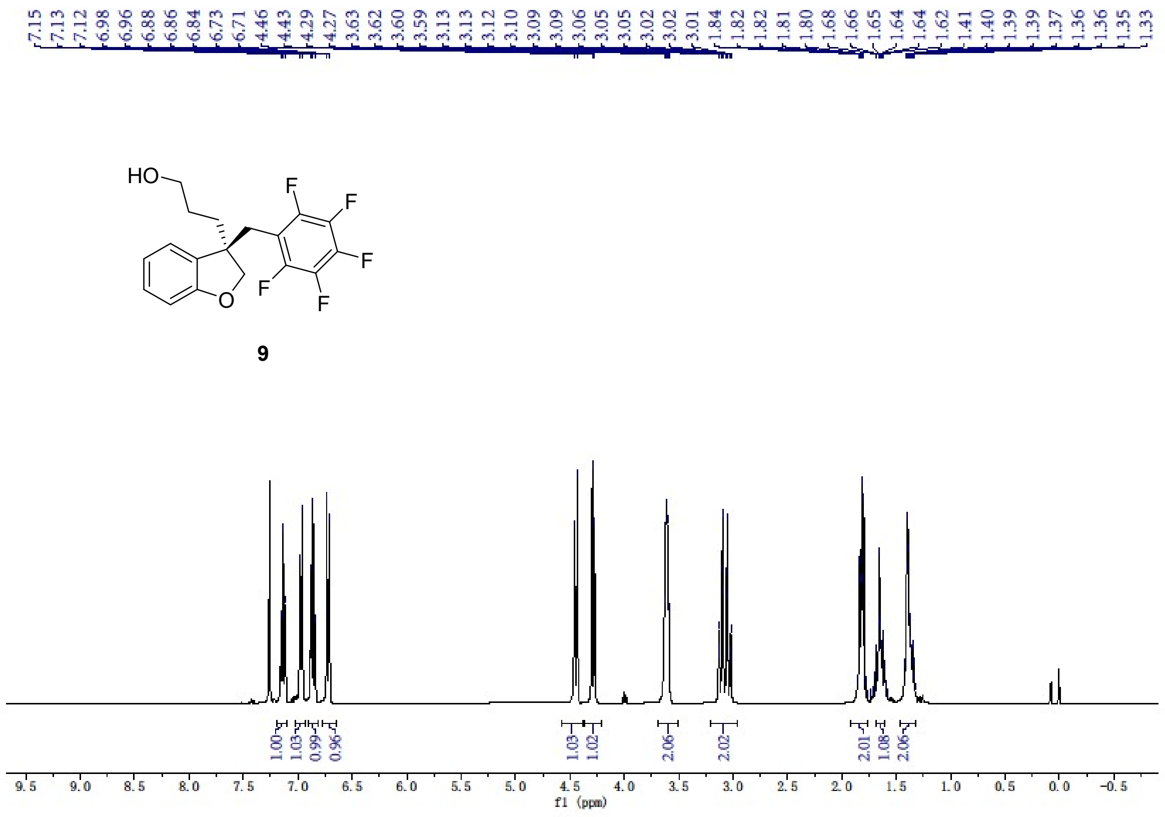
7.15 7.13 7.12 6.98 6.96 6.88 6.86 6.84 6.73 6.71 4.46 4.43 4.29 4.27 3.63 3.62 3.60 3.59 3.13 3.13 3.12 3.10 3.09 3.09 3.06 3.05 3.05 3.02 3.02 3.01 1.84 1.82 1.82 1.81 1.80 1.68 1.66 1.65 1.64 1.64 1.62 1.41 1.40 1.39 1.39 1.37 1.36 1.36 1.35 1.33
HO
F
F
F
F
F
O
9
1.00 1.03 0.99 0.96 1.03 1.02 2.06 2.02 2.01 1.08 2.06
9.5 9.0 8.5 8.0 7.5 7.0 6.5 6.0 5.5 5.0 4.5 4.0 3.5 3.0 2.5 2.0 1.5 1.0 0.5 0.0 -0.5
f1 (ppm)

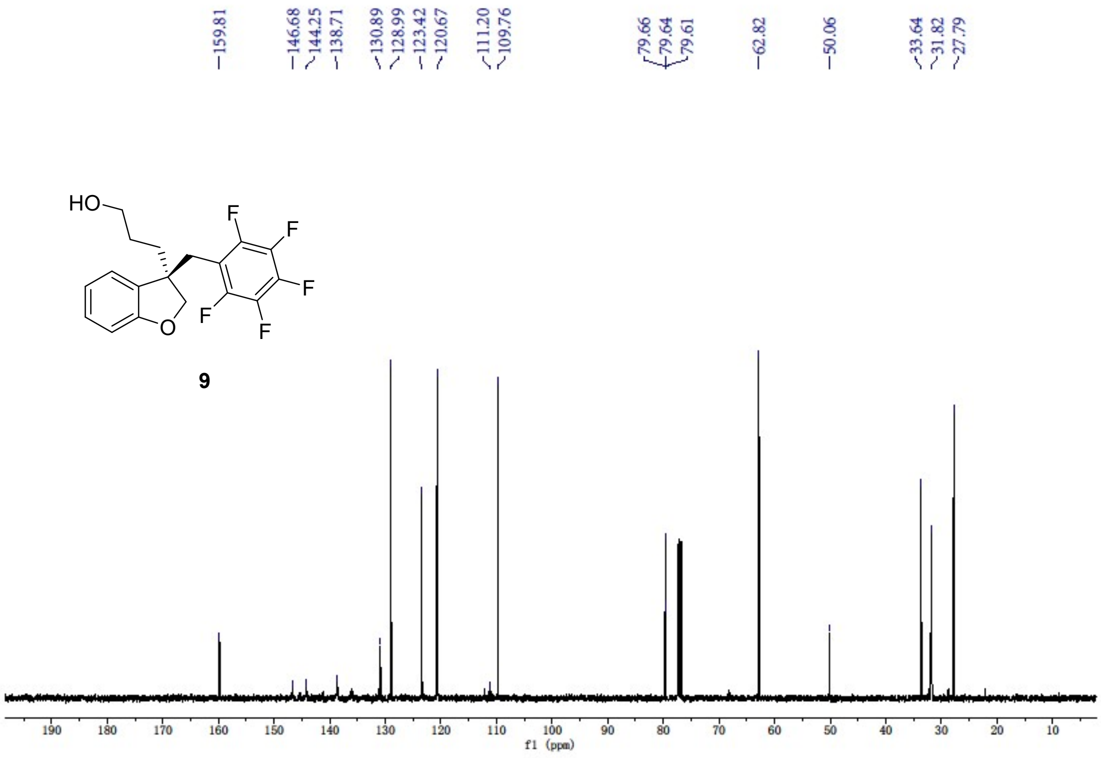
159.81 146.68 144.25 138.71 130.89 128.99 123.42 120.67 111.20 109.76 79.66 79.64 79.61 62.82 50.06 33.64 31.82 27.79
HO
F
F
F
F
F
O
9
190 180 170 160 150 140 130 120 110 100 90 80 70 60 50 40 30 20 10
f1 (ppm)

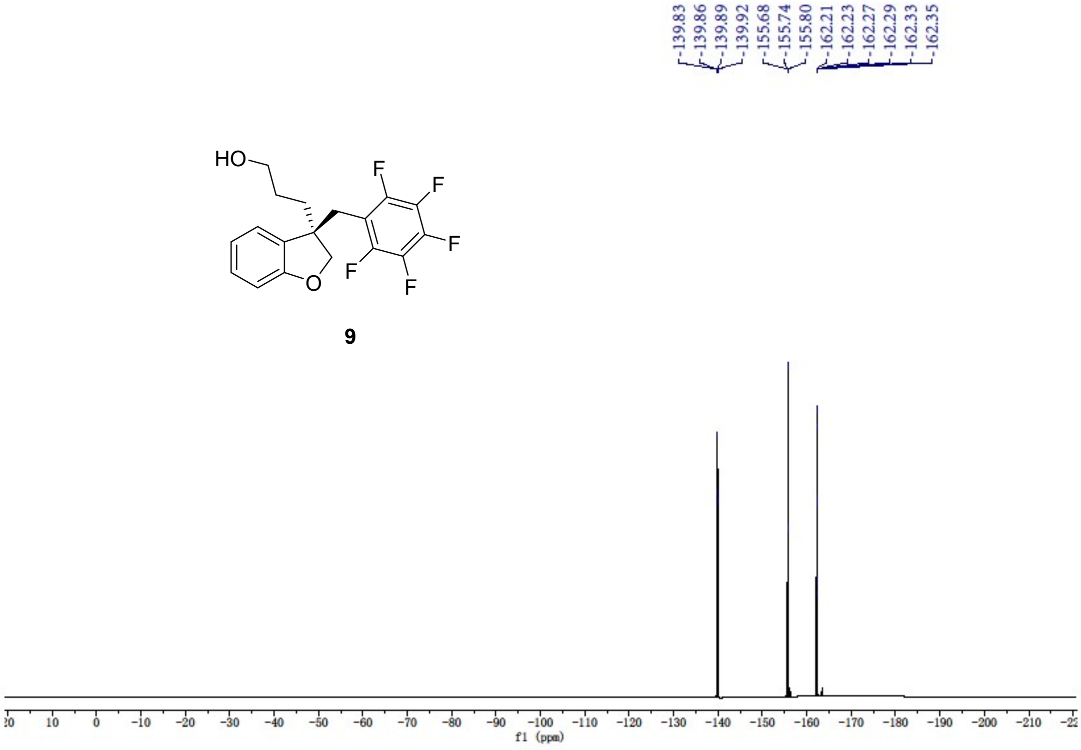

〈Chromatogram〉

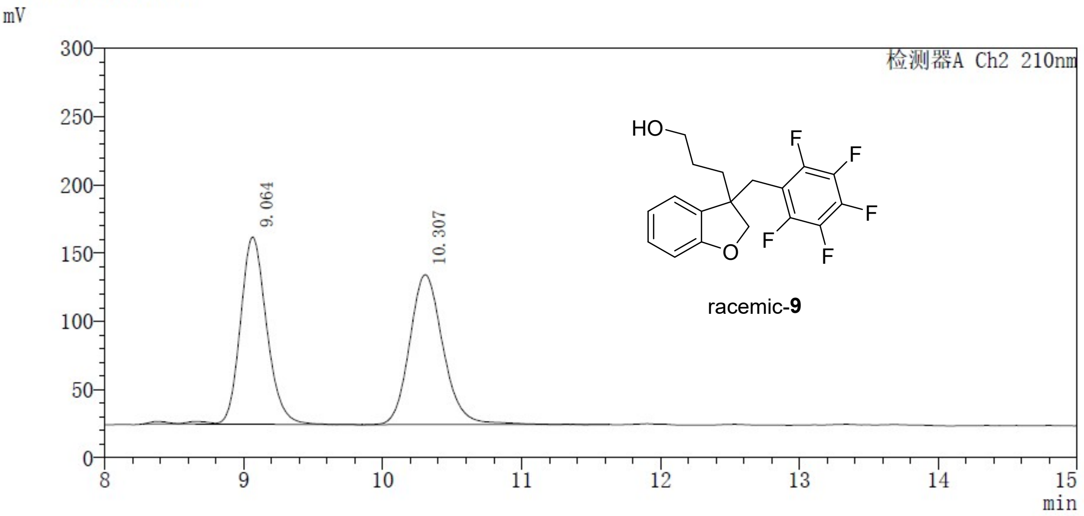

〈Peak Table〉

检测器A Ch2 210nm

No.	Ret.Time(min)	Height	Height%	Area	Area%
1	9.064	137137	55.514	1829053	49.802
2	10.307	109896	44.486	1843607	50.198
Total		247033	100.000	3672660	100.000

<Chromatogram>

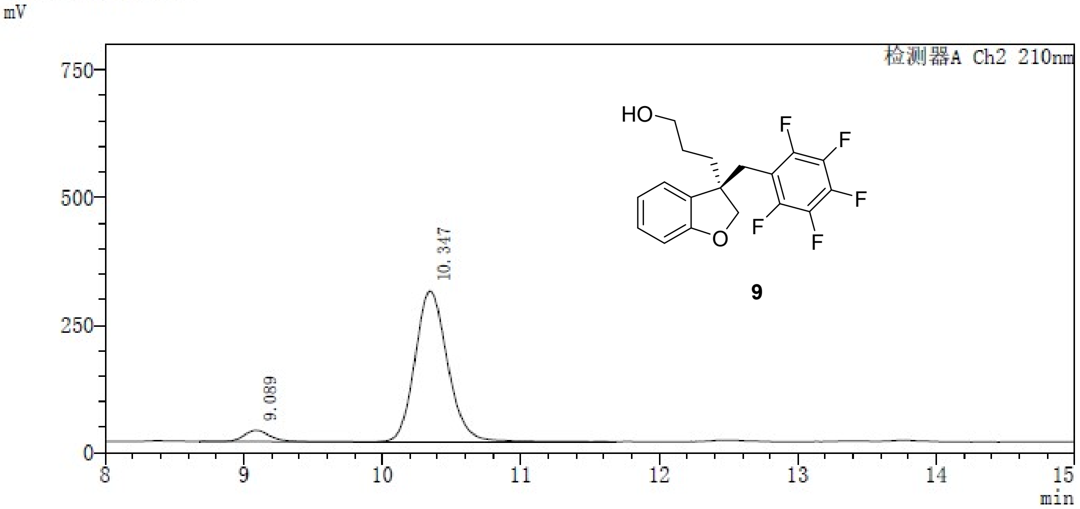

<Peak Table>

检测器A Ch2 210nm

No.	Ret. Time (min)	Height	Height%	Area	Area%
1	9.089	21857	6.887	282309	5.483
2	10.347	295502	93.113	4866438	94.517
Total		317359	100.000	5148747	100.000

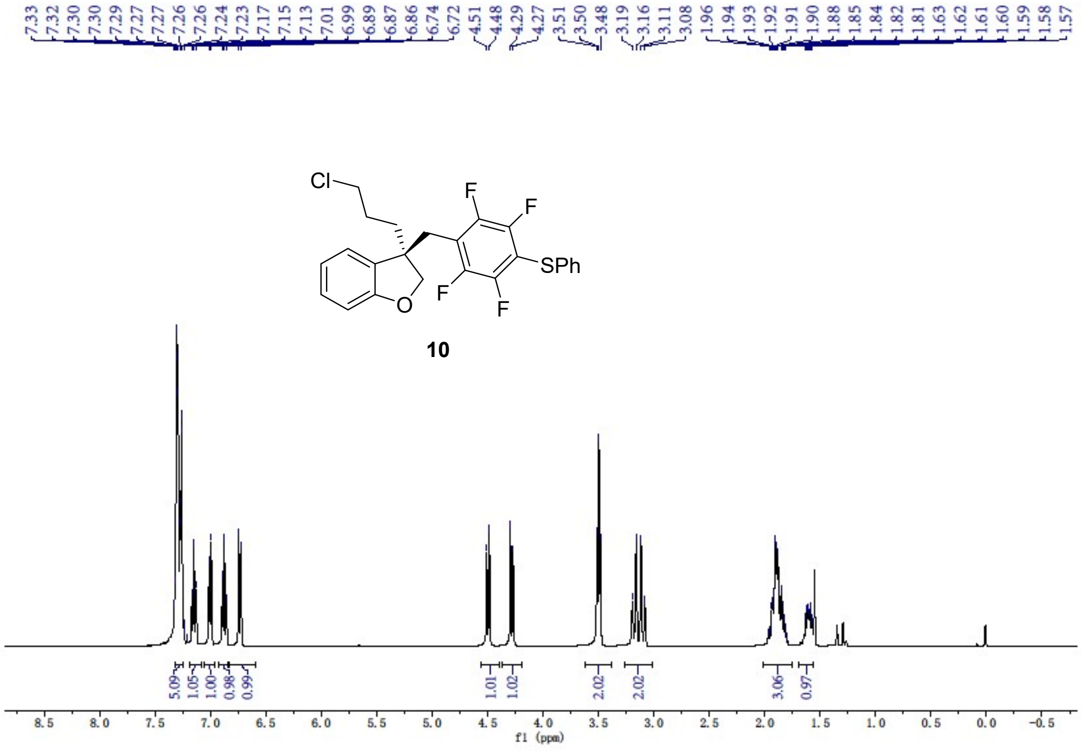

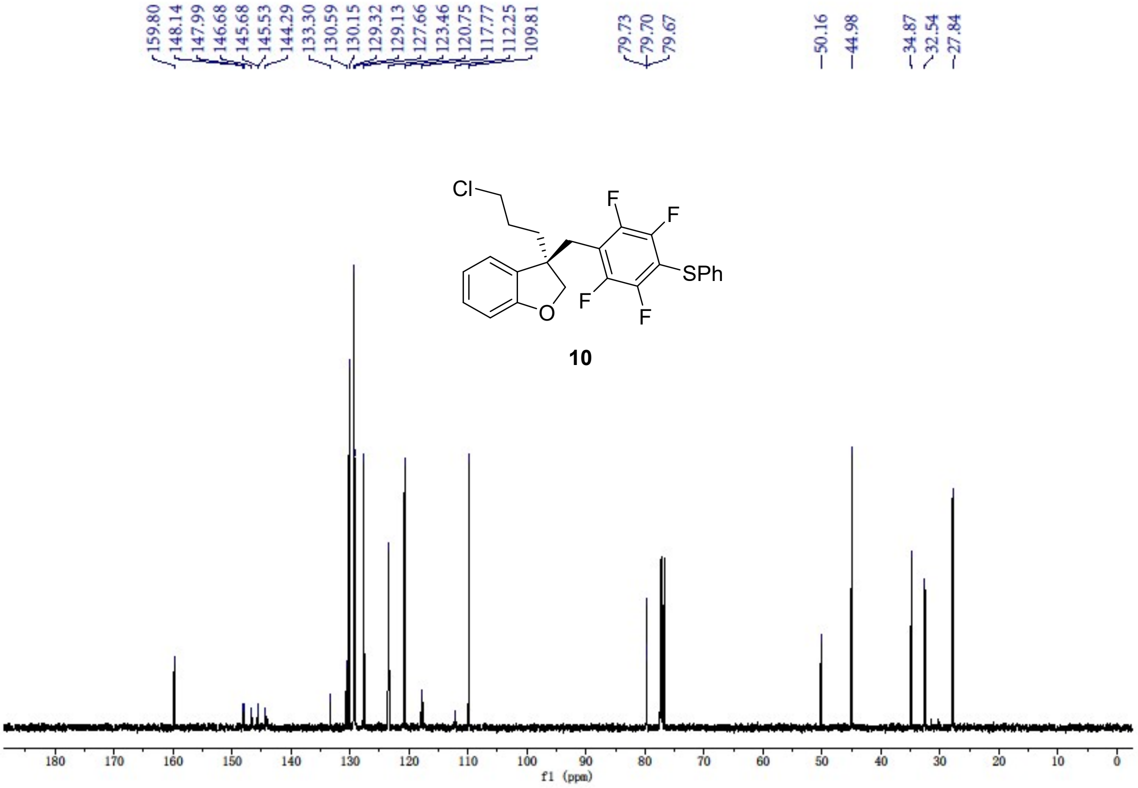
159.80
148.14
147.99
146.68
145.68
145.53
144.29
133.30
130.59
130.15
129.32
129.13
127.66
123.46
120.75
117.77
112.25
109.81
79.73
79.70
79.67
50.16
44.98
34.87
32.54
27.84
Cl
F
F
SPh
F
F
O
10
f1 (ppm)

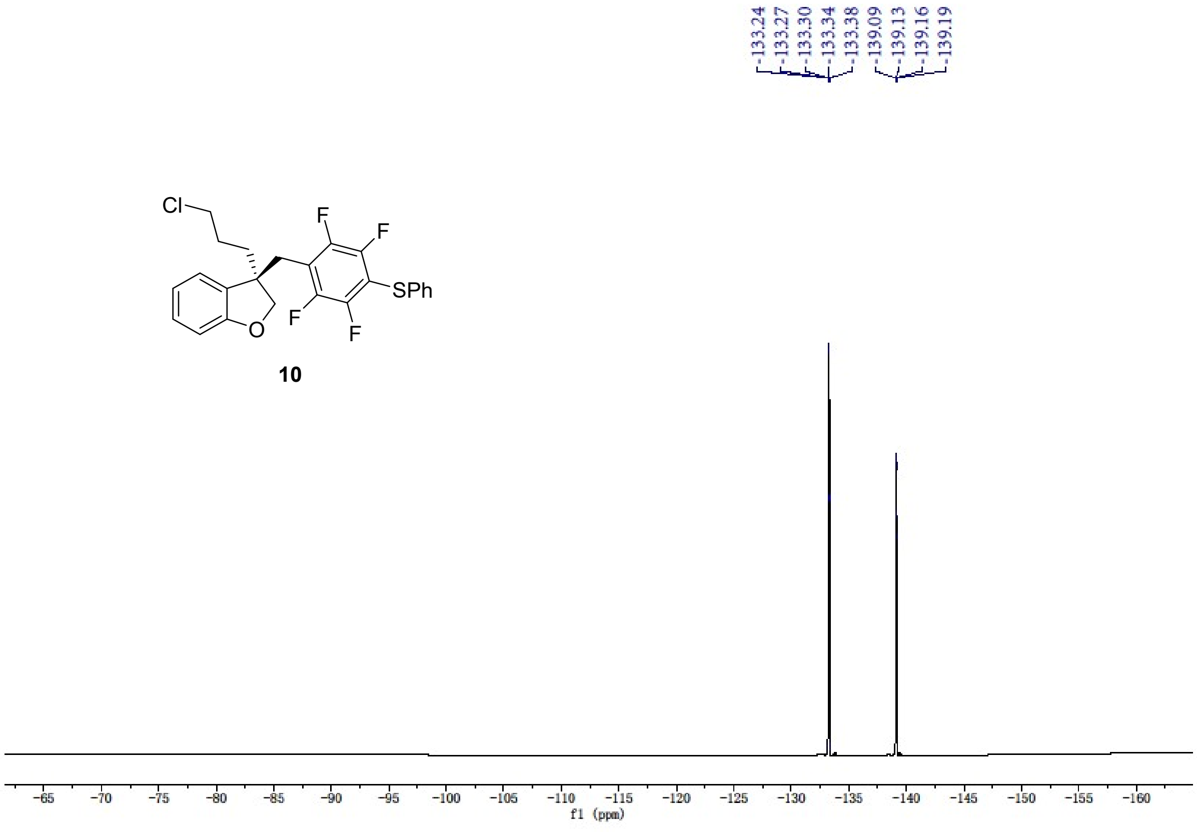
-133.24
-133.27
-133.30
-133.34
-133.38
-139.09
-139.13
-139.16
-139.19
Cl
F
F
SPh
F
F
O
10
f1 (ppm)

〈Chromatogram〉

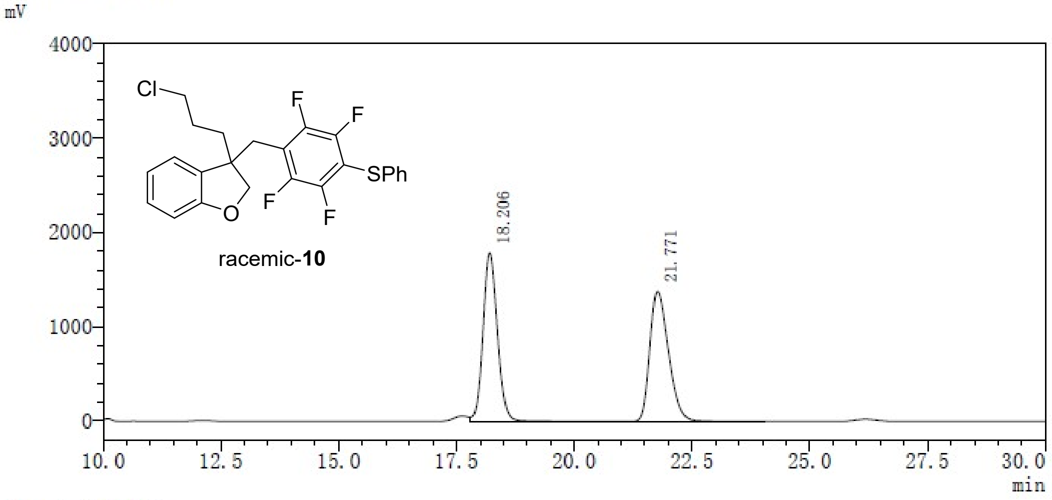

〈Peak Table〉

检测器A Ch2 210nm

No.	Ret. Time(min)	Height	Height%	Area	Area%
1	18.206	1784132	56.415	38058507	50.256
2	21.771	1378391	43.585	37670333	49.744
Total		3162522	100.000	75728840	100.000

〈Chromatogram〉

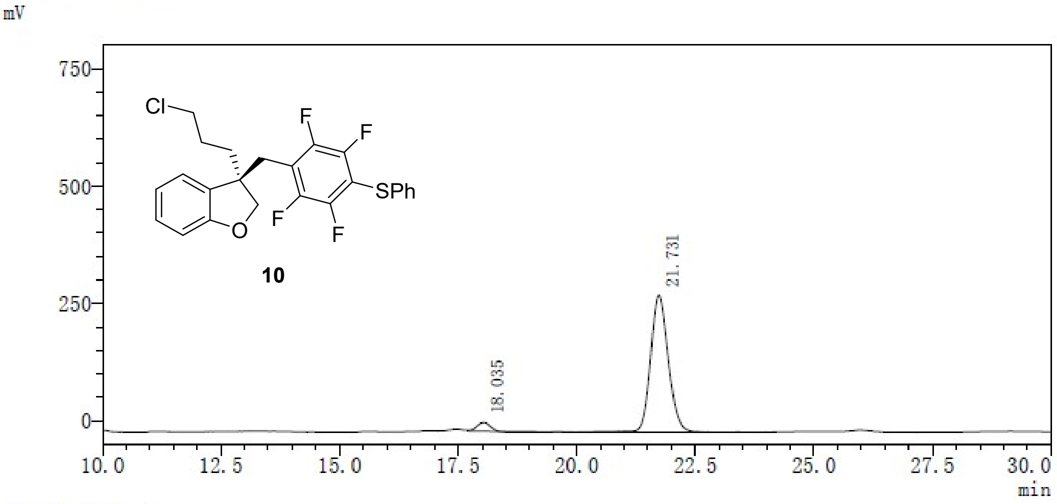

〈Peak Table〉

检测器A Ch2 210nm

No.	Ret. Time(min)	Height	Height%	Area	Area%
1	18.035	18265	5.914	276605	3.536
2	21.731	290607	94.086	7545019	96.464
Total		308872	100.000	7821624	100.000

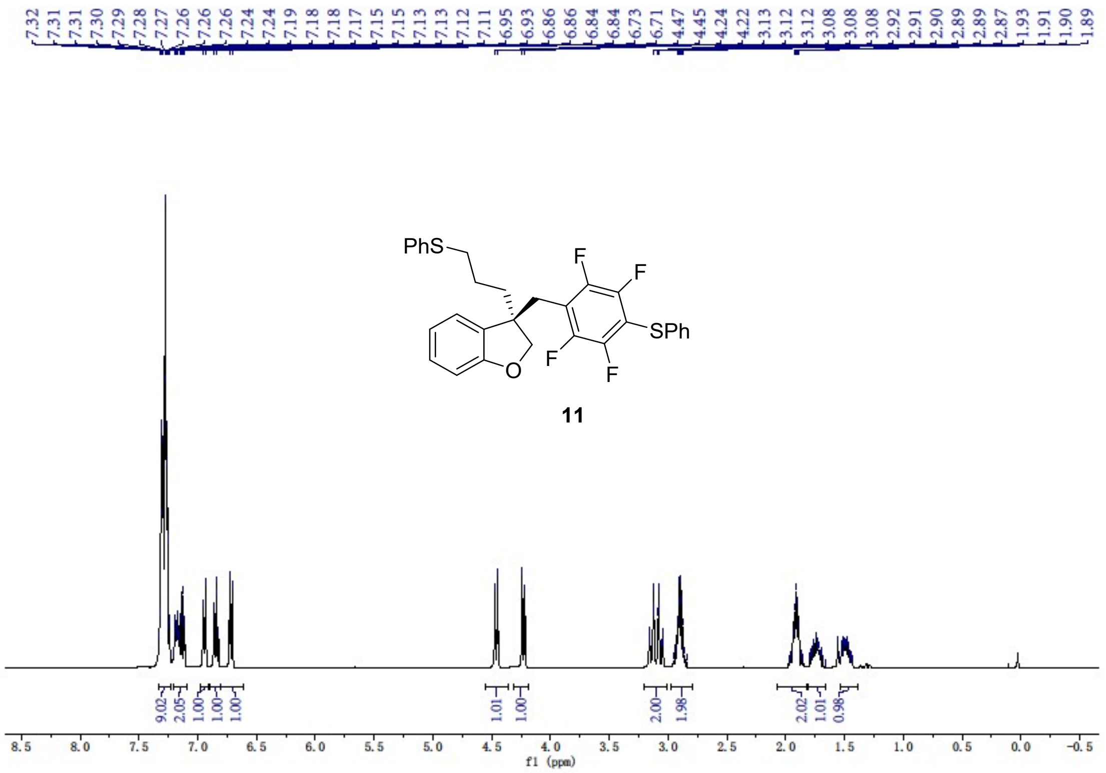
7.32 7.31 7.31 7.30 7.29 7.28 7.27 7.26 7.26 7.26 7.24 7.24 7.19 7.18 7.18 7.17 7.15 7.15 7.13 7.13 7.12 7.11 6.95 6.93 6.86 6.86 6.84 6.84 6.73 6.71 4.47 4.45 4.24 4.22 3.13 3.12 3.12 3.08 3.08 3.08 2.92 2.91 2.90 2.89 2.89 2.87 1.93 1.91 1.90 1.89
PhS
F
F
SPh
F
F
O
11
9.02 2.05 1.00 1.00 1.00 1.01 1.00 2.00 1.98 2.02 1.01 0.98
f1 (ppm)

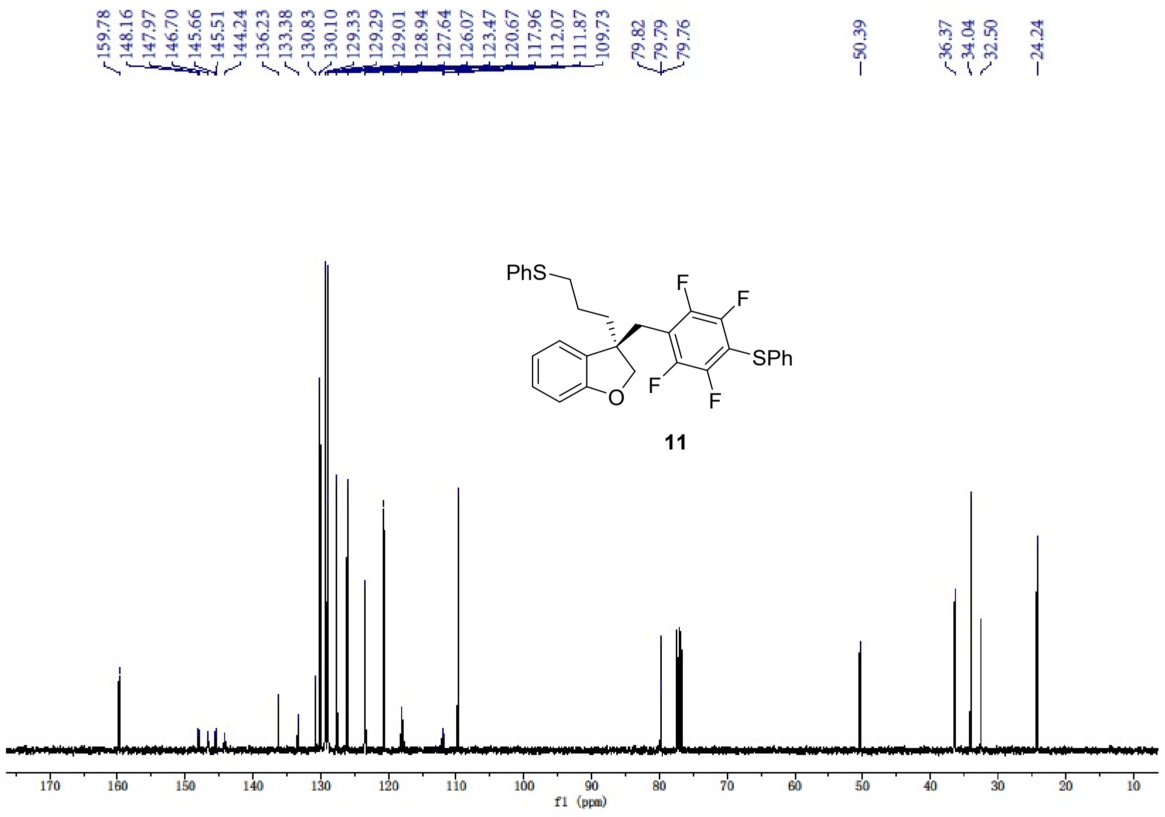
159.78 148.16 147.97 146.70 145.66 145.51 144.24 136.23 133.38 130.83 130.10 129.33 129.29 129.01 128.94 127.64 126.07 123.47 120.67 117.96 112.07 111.87 109.73 79.82 79.79 79.76 50.39 36.37 34.04 32.50 24.24
PhS
F
F
SPh
F
F
O
11
f1 (ppm)

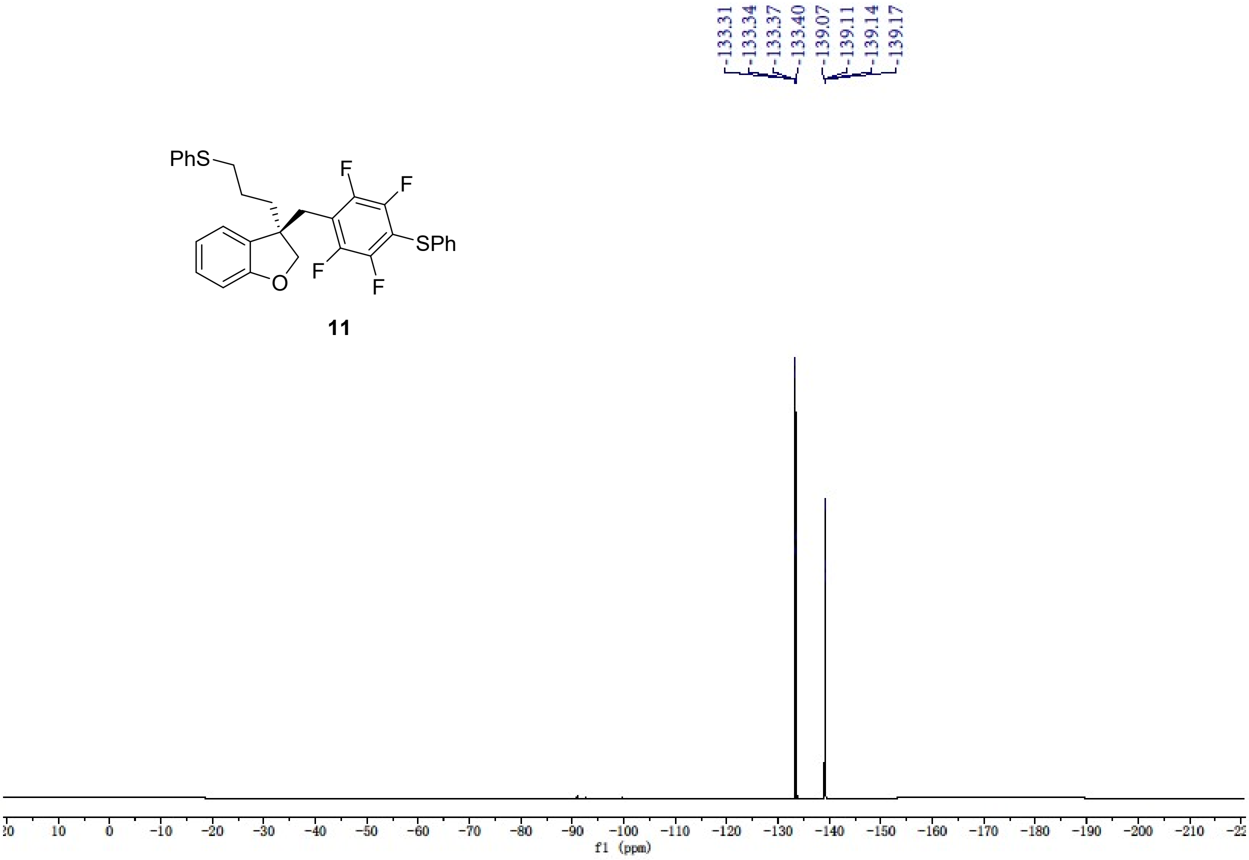

<Chromatogram>

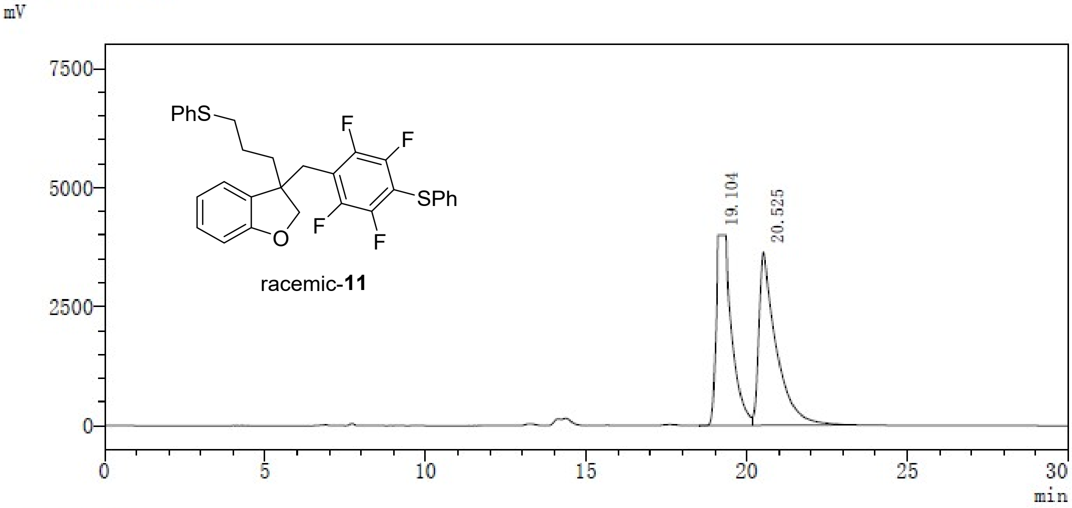

<Peak Table>

检测器A Ch2 210nm

No.	Ret. Time(min)	Height	Height%	Area	Area%
1	19.104	3996610	52.340	135286896	49.804
2	20.525	3639228	47.660	136351073	50.196
Total		7635838	100.000	271637970	100.000

<Chromatogram>

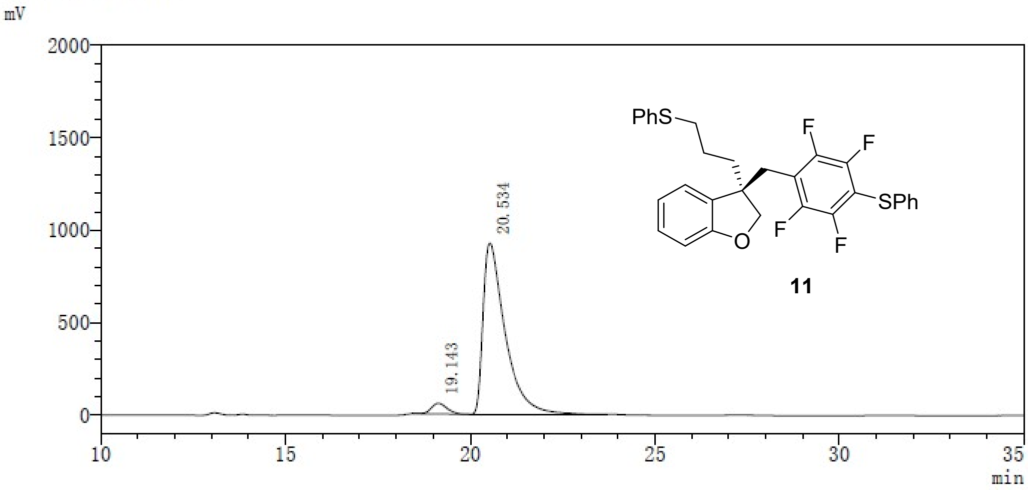

<Peak Table>

检测器A Ch2 210nm

No.	Ret. Time (min)	Height	Height%	Area	Area%
1	19.143	58078	5.912	1712786	4.260
2	20.534	924308	94.088	38491503	95.740
Total		982386	100.000	40204289	100.000

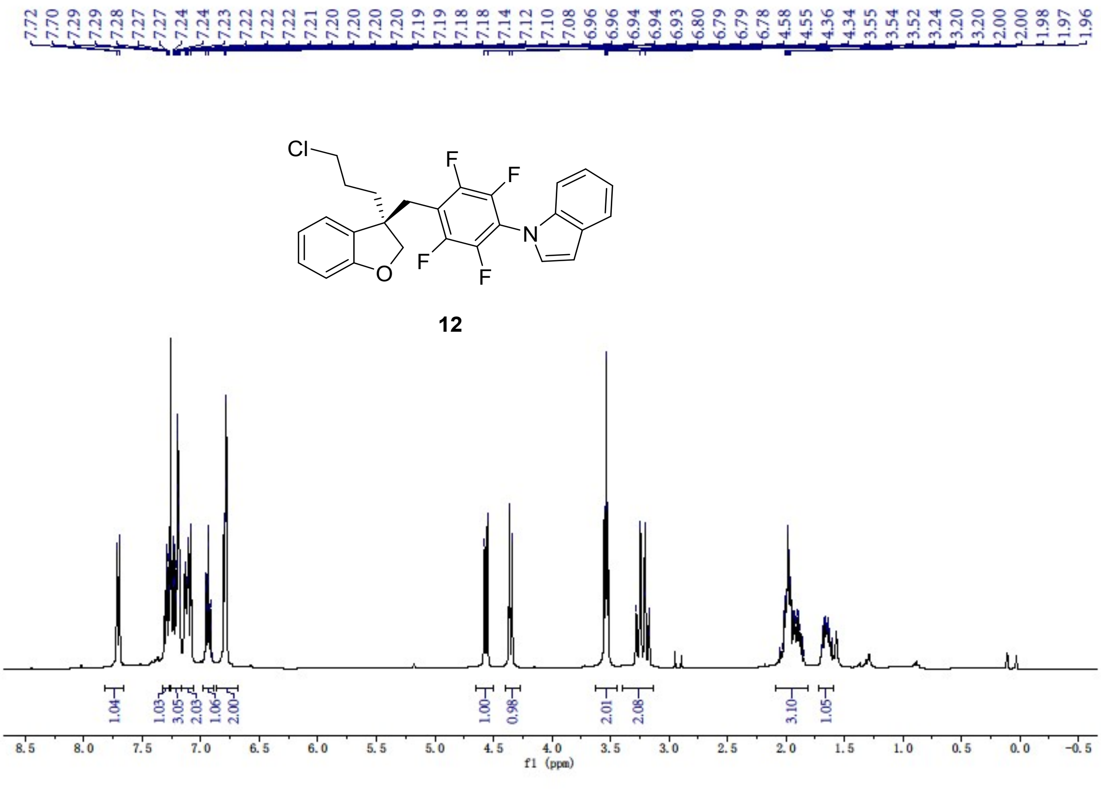

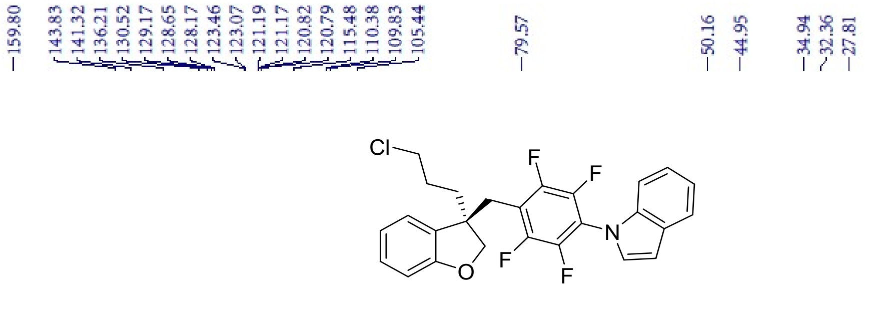
159.80
143.83
141.32
136.21
130.52
129.17
128.65
128.17
123.46
123.07
121.19
121.17
120.82
120.79
115.48
110.38
109.83
105.44
79.57
50.16
44.95
34.94
32.36
27.81
Cl
F
F
F
F
N
O

12

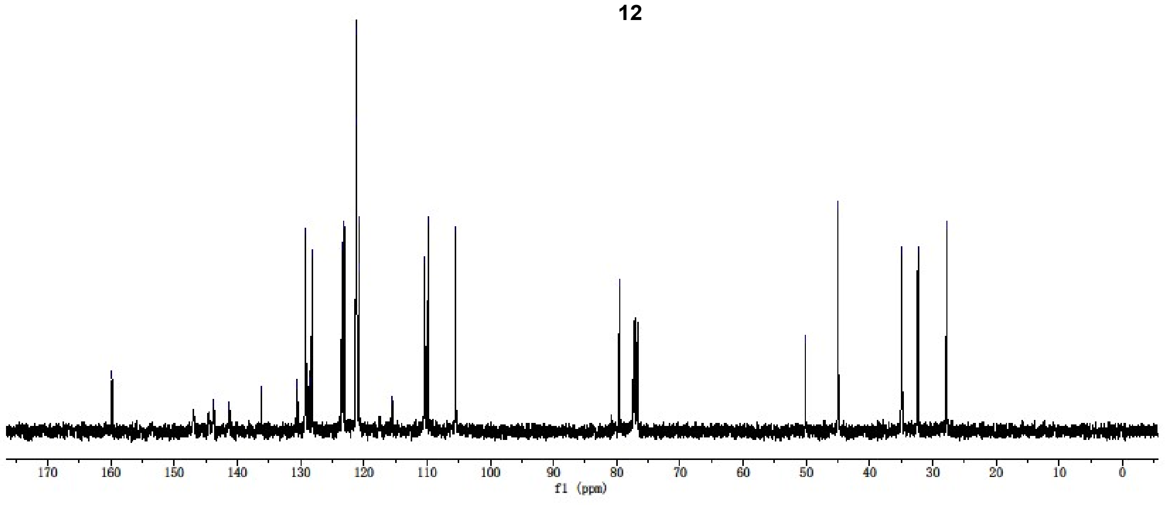
170
160
150
140
130
120
110
100
90
80
70
60
50
40
30
20
10
0
f1 (ppm)

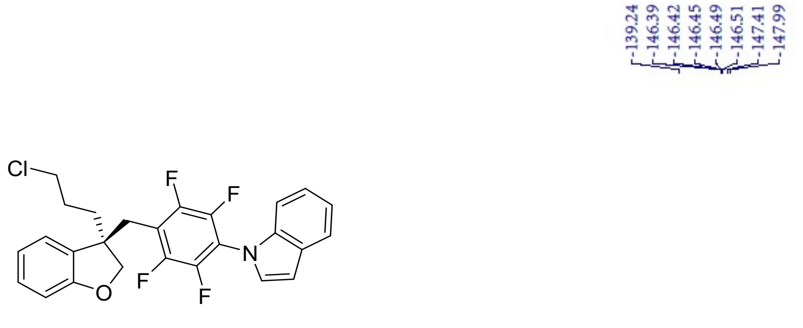
-139.24
-146.39
-146.42
-146.45
-146.49
-146.51
-147.41
-147.99
Cl
F
F
F
F
N
O

12

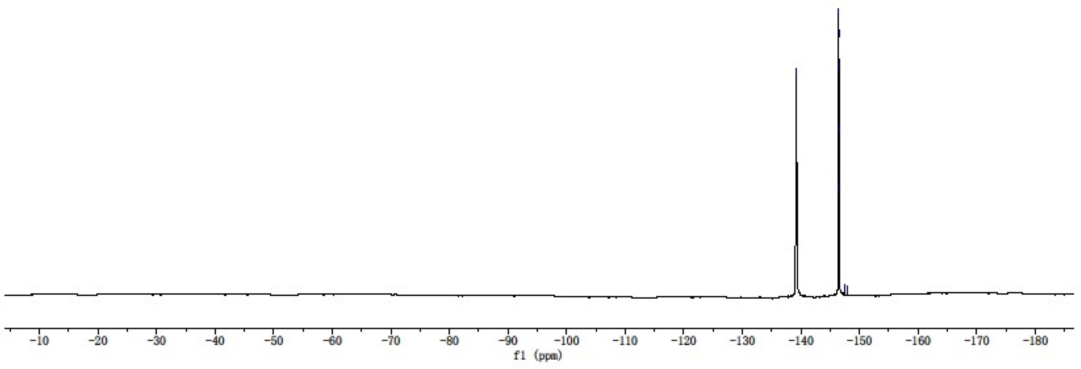
-10
-20
-30
-40
-50
-60
-70
-80
-90
-100
-110
-120
-130
-140
-150
-160
-170
-180
f1 (ppm)

<Chromatogram>

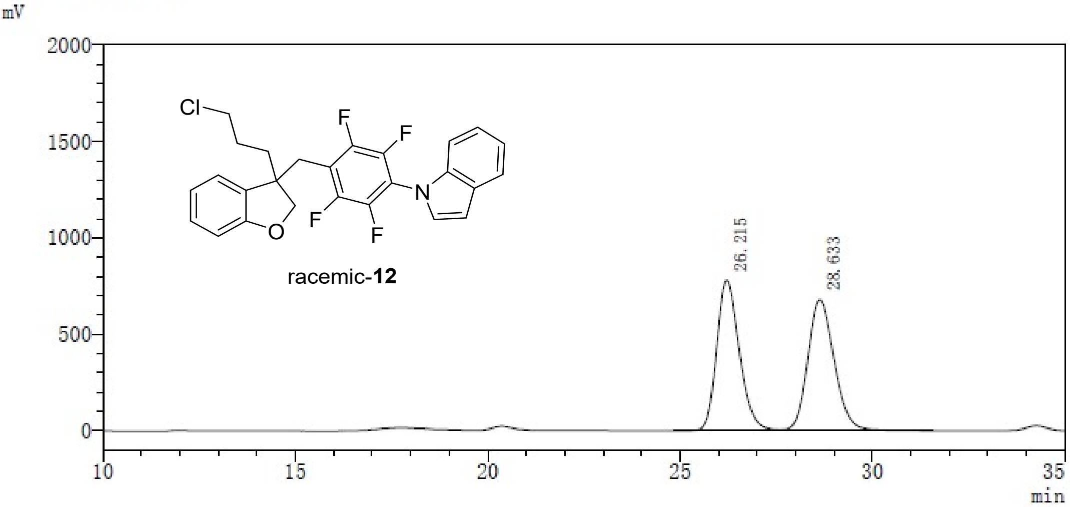

<Peak Table>

检测器A Ch2 210nm

No.	Ret. Time (min)	Height	Height%	Area	Area%
1	26.215	778161	53.425	32000741	49.609
2	28.633	678379	46.575	32505355	50.391
Total		1456541	100.000	64506097	100.000

<Chromatogram>

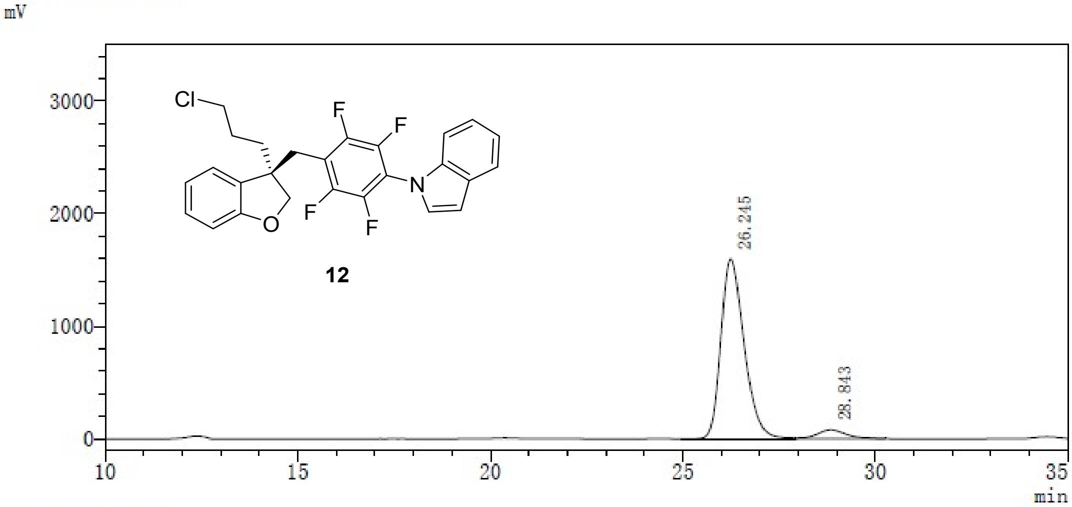

<Peak Table>

检测器A Ch2 210nm

No.	Ret. Time (min)	Height	Height%	Area	Area%
1	26.245	1596583	95.276	67045314	94.370
2	28.843	79159	4.724	3999910	5.630
Total		1675741	100.000	71045224	100.000

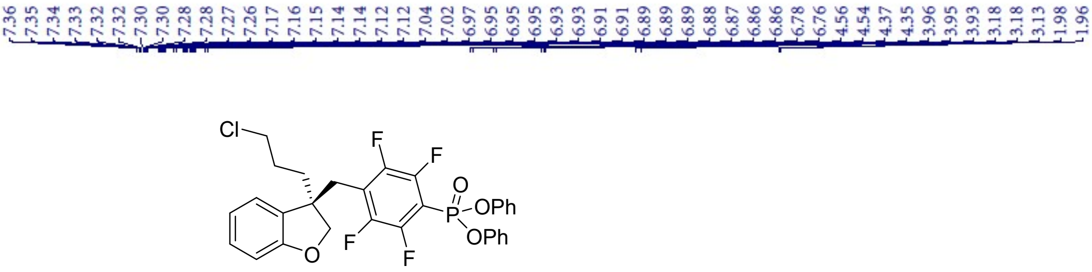
7.36 7.35 7.34 7.33 7.32 7.30 7.30 7.28 7.28 7.27 7.26 7.17 7.16 7.15 7.14 7.14 7.12 7.12 7.04 7.02 6.97 6.95 6.95 6.95 6.93 6.93 6.91 6.91 6.89 6.89 6.89 6.88 6.87 6.86 6.86 6.78 6.76 4.56 4.54 4.37 4.35 3.96 3.95 3.93 3.18 3.18 3.13 1.98 1.96
Cl
F
F
O
P
OPh
OPh
F
F
O

13

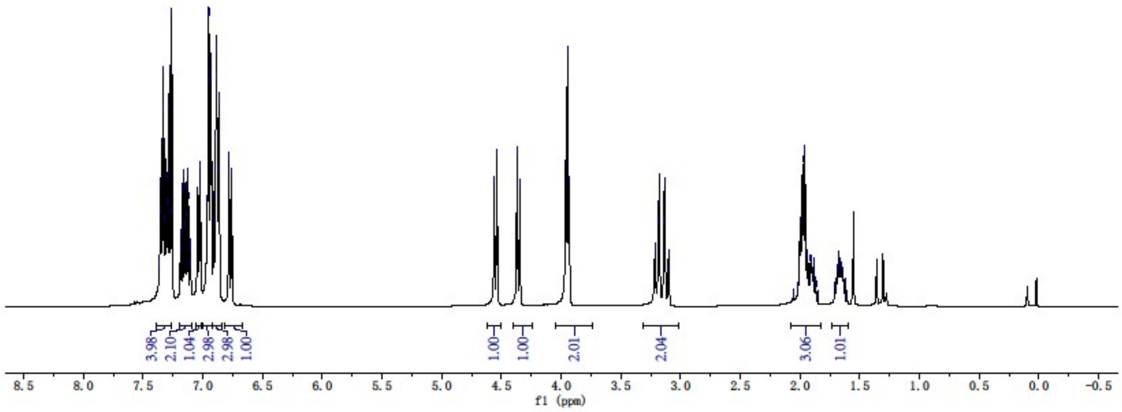
3.98
2.10
1.04
2.98
2.98
1.00
1.00
1.00
2.01
2.04
3.06
1.01
f1 (ppm)

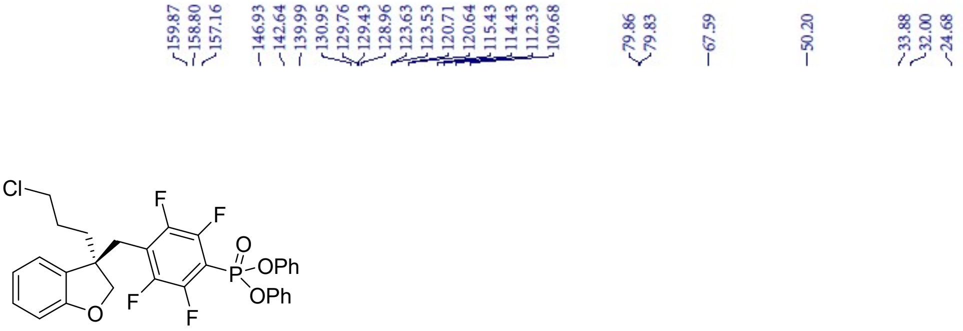
159.87 158.80 157.16 146.93 142.64 139.99 130.95 129.76 129.43 128.96 123.63 123.53 120.71 120.64 115.43 114.43 112.33 109.68 79.86 79.83 67.59 50.20 33.88 32.00 24.68
Cl
F
F
O
P
OPh
OPh
F
F
O

13

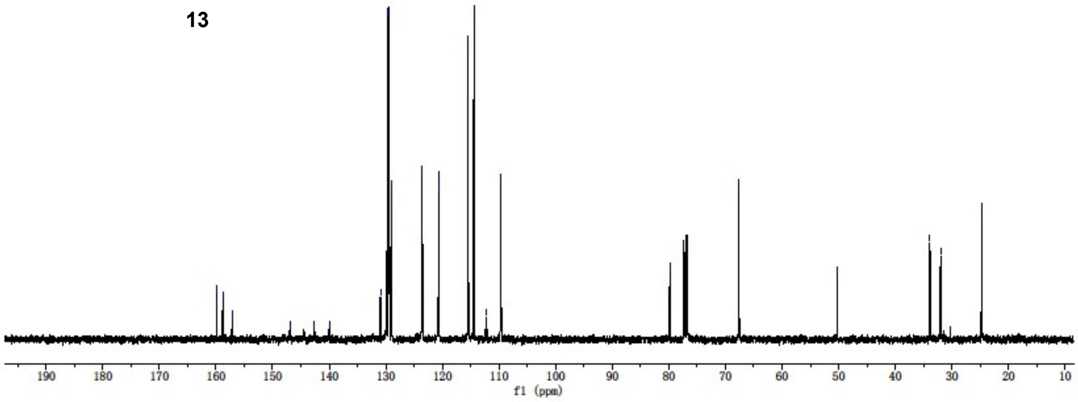
f1 (ppm)

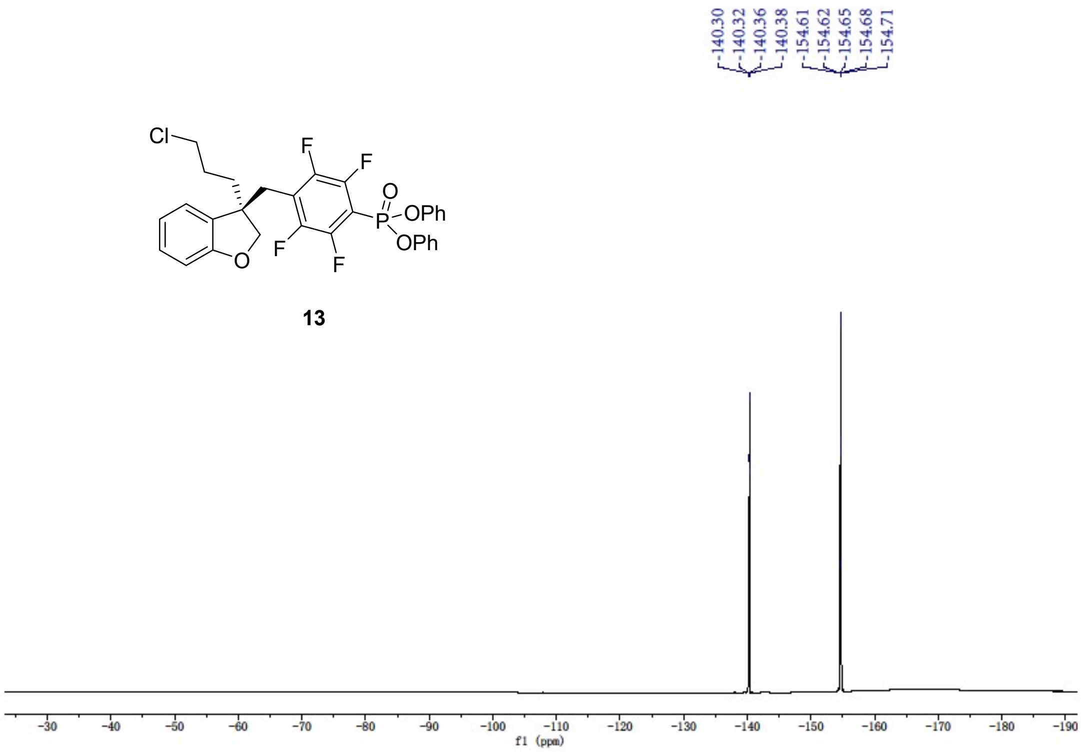
-140.30
-140.32
-140.36
-140.38
-154.61
-154.62
-154.65
-154.68
-154.71
Cl
F
F
F
F
O
P
OPh
OPh
O
13
f1 (ppm)

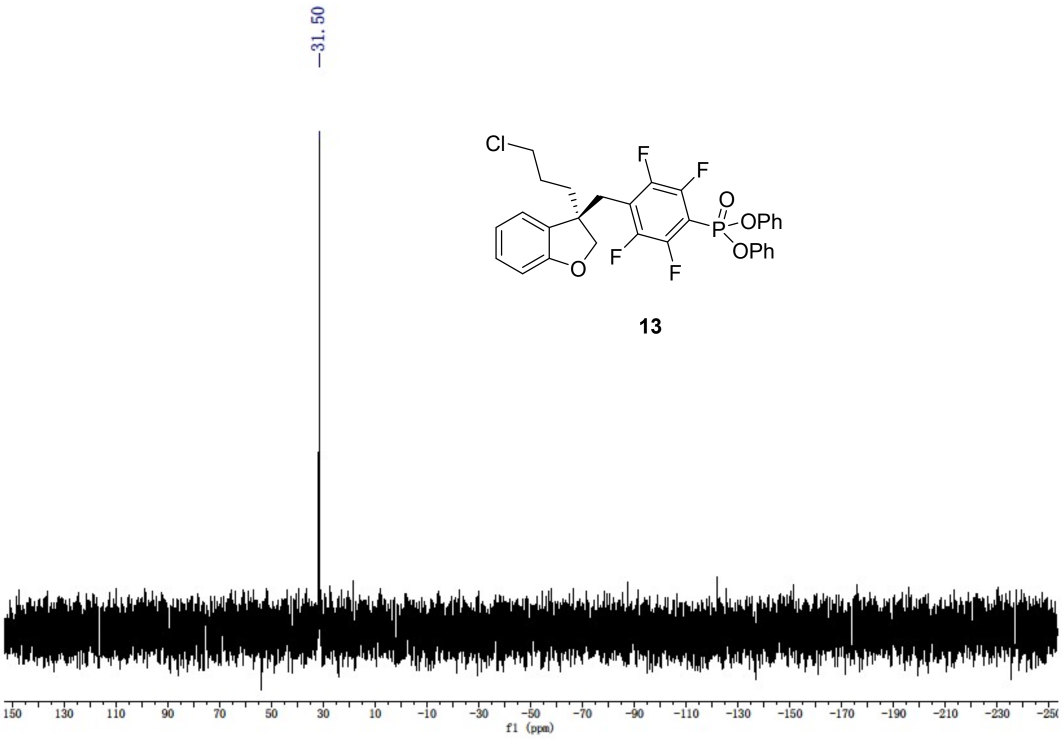
—31.50
Cl
F
F
F
F
O
P
OPh
OPh
O
13
f1 (ppm)

〈Chromatogram〉

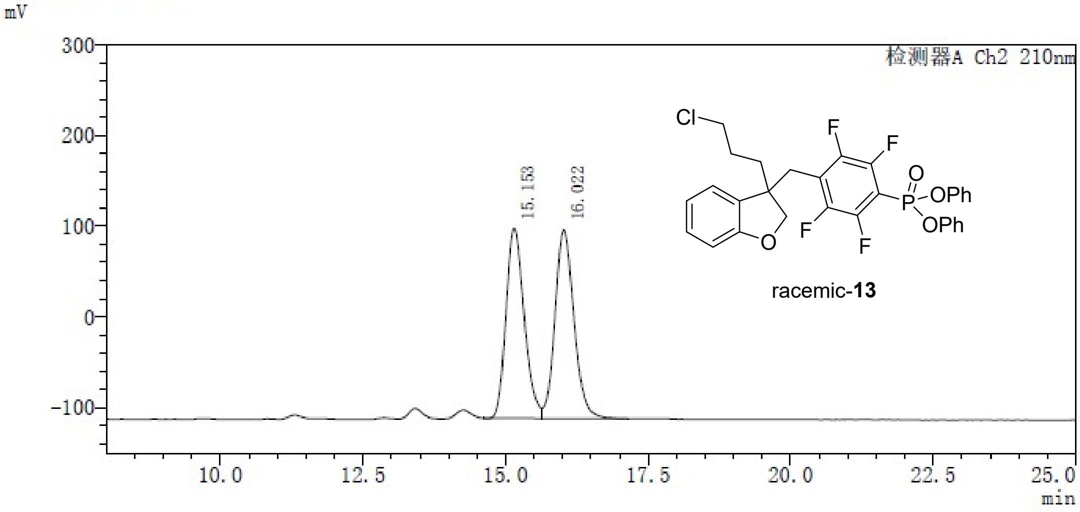

〈Peak Table〉

检测器A Ch2 210nm

No.	Ret. Time (min)	Height	Height%	Area	Area%
1	15.153	209174	50.159	4647445	49.158
2	16.022	207849	49.841	4806708	50.842
Total		417023	100.000	9454154	100.000

〈Chromatogram〉

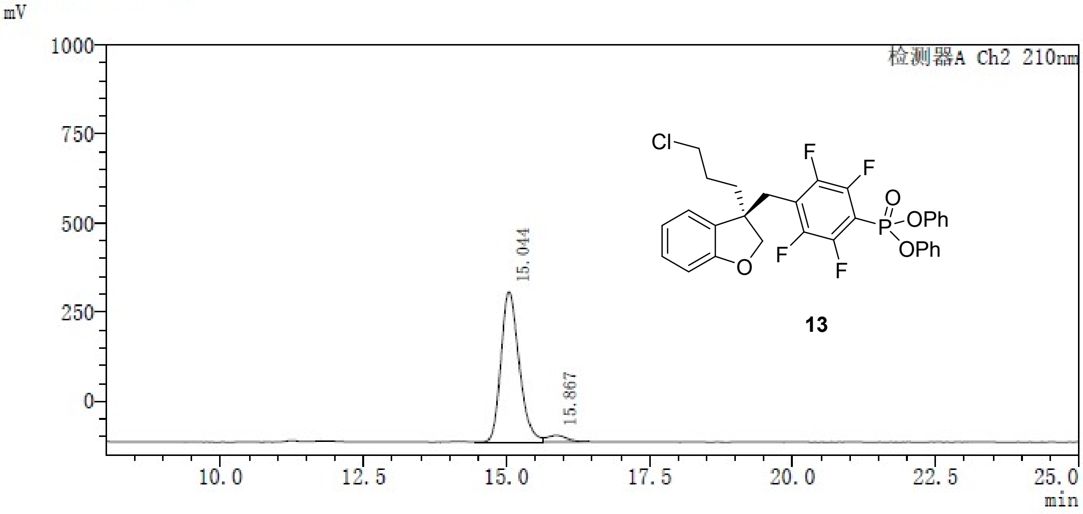

〈Peak Table〉

检测器A Ch2 210nm

No.	Ret. Time (min)	Height	Height%	Area	Area%
1	15.044	419979	95.999	9291599	95.822
2	15.867	17505	4.001	405083	4.178
Total		437483	100.000	9696682	100.000

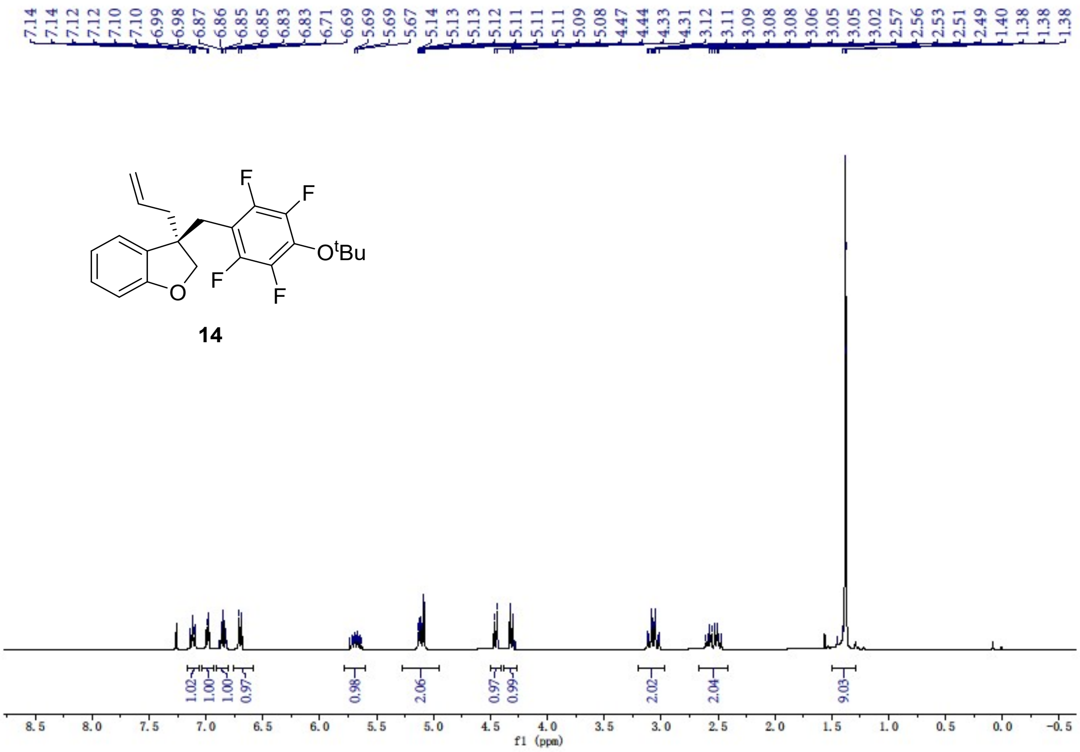
7.14 7.14 7.12 7.12 7.10 7.10 6.99 6.98 6.87 6.86 6.85 6.85 6.83 6.83 6.71 6.69 5.69 5.69 5.67 5.14 5.13 5.13 5.12 5.11 5.11 5.11 5.09 5.08 4.47 4.44 4.33 4.31 3.12 3.11 3.09 3.08 3.08 3.06 3.05 3.05 3.02 2.57 2.56 2.53 2.51 2.49 1.40 1.38 1.38 1.38
F
F
O^{t}Bu
F
F
O
14
1.02 1.00 1.00 0.97 0.98 2.06 0.97 0.99 2.02 2.04 9.03
f1 (ppm)

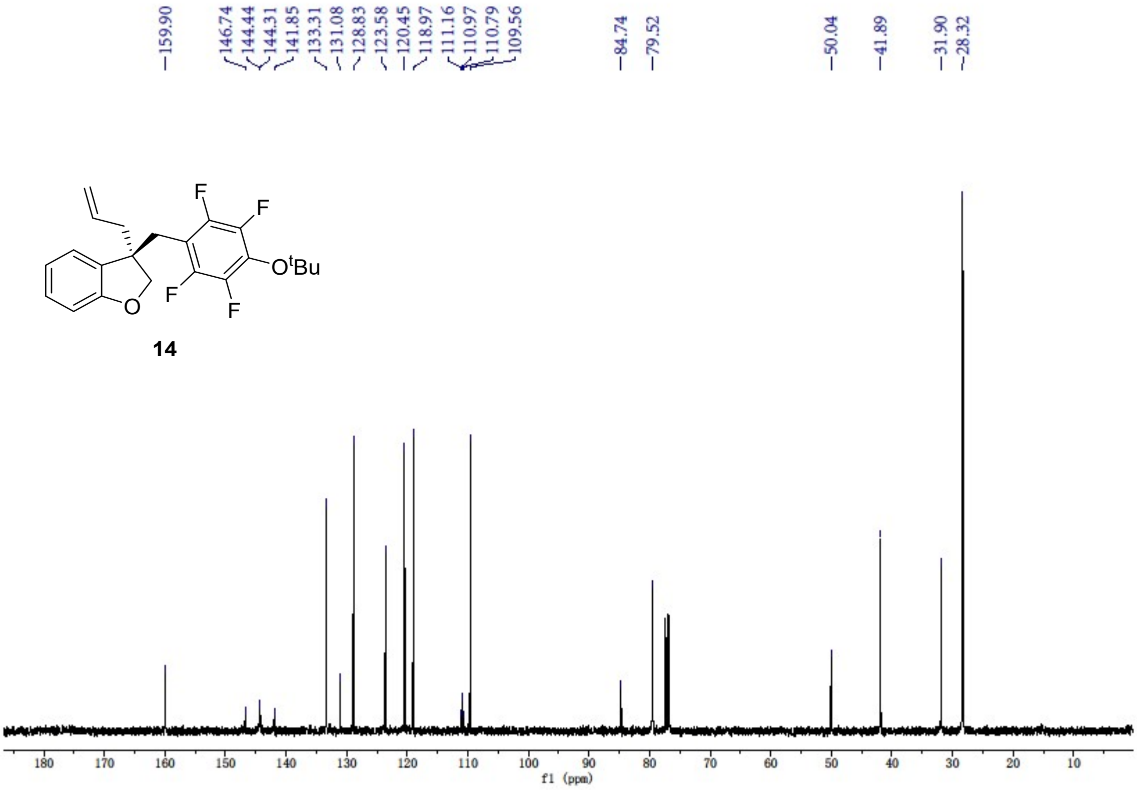
159.90 146.74 144.44 144.31 141.85 133.31 131.08 128.83 123.58 120.45 118.97 111.16 110.97 110.79 109.56 84.74 79.52 50.04 41.89 31.90 28.32
F
F
O^{t}Bu
F
F
O
14
f1 (ppm)

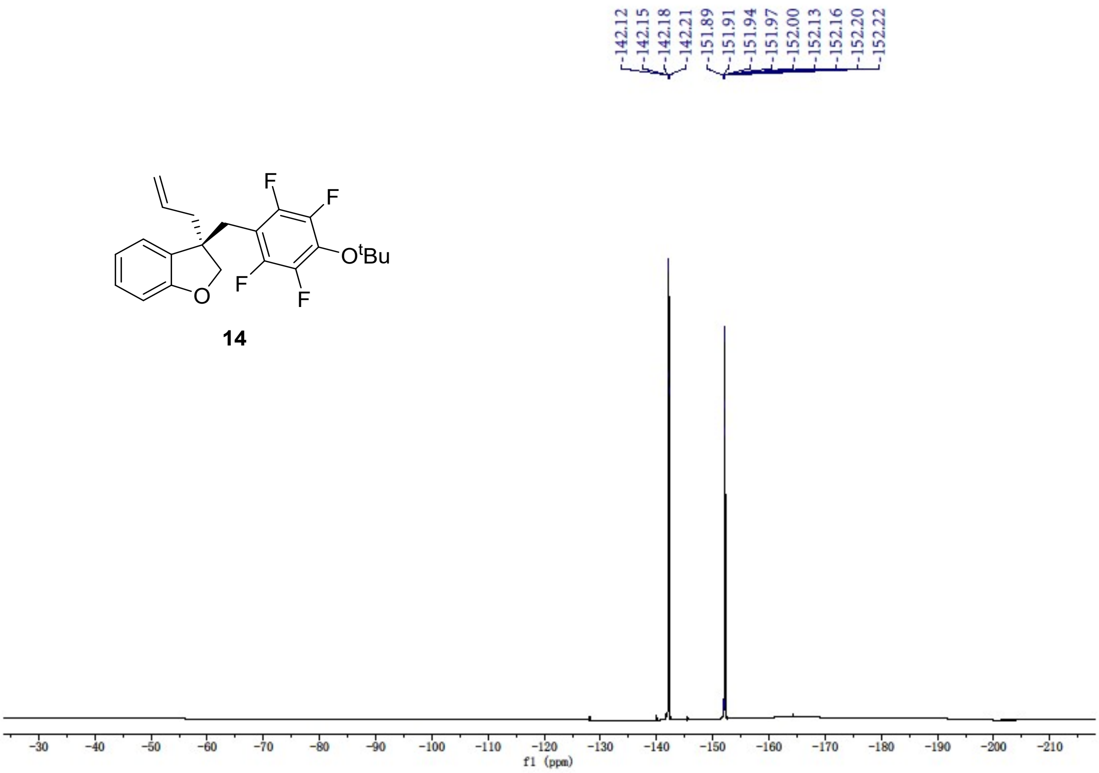

<Chromatogram>

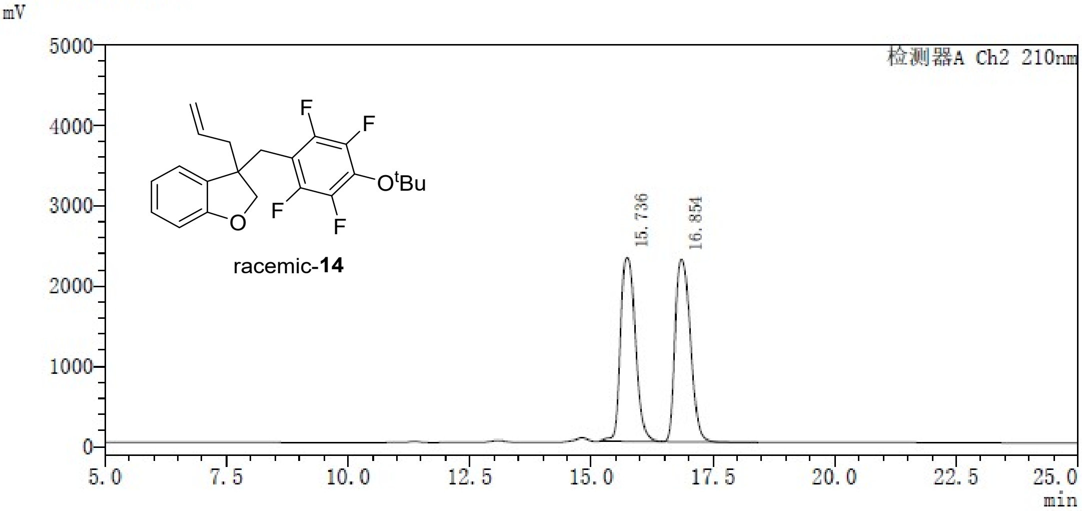

<Peak Table>

检测器A Ch2 210nm

No.	Ret.Time(min)	Height	Height%	Area	Area%
1	15.736	2287485	50.200	48643111	49.749
2	16.854	2269238	49.800	49133147	50.251
Total		4556723	100.000	97776258	100.000

<Chromatogram>

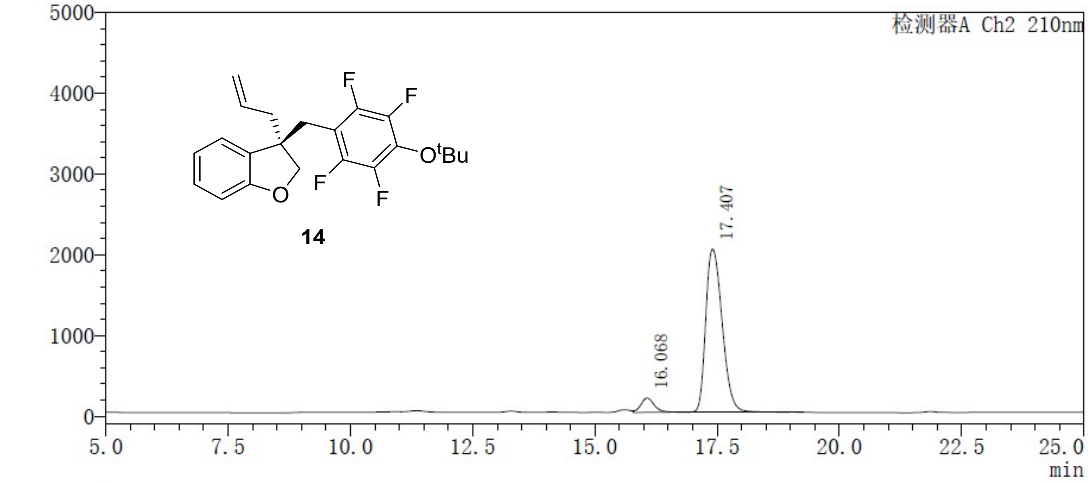

<Peak Table>

检测器A Ch2 210nm

No.	Ret. Time(min)	Height	Height%	Area	Area%
1	16.068	176423	8.028	3363185	6.609
2	17.407	2021272	91.972	47522120	93.391
Total		2197694	100.000	50885305	100.000

15. References

(1) Zhang, Z.-M., Xu, B., Wu, L., Zhou, L., Ji, D., Liu, Y., Li, Z., Zhang J. Palladium/XuPhos-Catalyzed Enantioselective Carboiodination of Olefin-Tethered Aryl Iodides. *J. Am. Chem. Soc.* **2019**, *141*, 8110−8115.

(2) Xie, W.; Heo, J.; Kim, D.; Chang, S. Copper-Catalyzed Direct C–H Alkylation of Polyfluoroarenes by Using Hydrocarbons as an Alkylating Source. *J. Am. Chem. Soc*. **2020**, *142*, 7487−7496.

(3) Li, S.-Q.; Xiong P.; Zhu L.; Qian X.-Y.; Xu H.-C. A General $CuCl_2$-Promoted Alkene Aminochlorination Reaction. *Eur. J. Org. Chem*. **2016**, 3449−3455.

(4) Menchikov, L. G.; Vorogushin, A. V.; Korneva, O. S.; Nefedov, D. M. An Effective Method for Alcohol Preparation by Hydrolysis of Organohalides in the Presence of Copper and its Salts in Aqueous DMSO. *Mendeleev Commun.* **1995**, *5*, 223–224.

(5) Zhao, X.; Yu, Z.; Yan, S.; Wu, S.; Liu, R.; He, W.; Wang, L. Ruthenium(III) Chloride Catalyzed Efficient Synthesis of Unsymmetrical Diorganyl Selenides via Cleavage of Dibenzyl and Diphenyl Diselenides in the Presence of Zinc. *J. Org. Chem.* **2005**, *70*, 7338–7341.

(6) Xie, W.; Heo, J.; Kim, D.; Chang, S. Copper-Catalyzed Direct C–H Alkylation of Polyfluoroarenes by Using Hydrocarbons as an Alkylating Source. *J. Am. Chem. Soc*. **2020**, *142*, 7487−7496.

(7) Cohen, R. J.; Fox, D. L.; Eubank, J. F.; Salvatore, R. N. Mild and efficient Cs_2CO_3-promoted synthesis of phosphonates. *Tetrahedron Lett.* **2003**, *44*, 8617−8621.

(8) Bartsch, R. A. Ionic Association in Base-Promoted β-Elimination Reactions. *Acc. Chem. Res.* **1975**, *8*, 239−245.

(9) Tsvetkov, E. N.; Bondarenko, N. A.; Malakhova, I. G.; Kabachnik, M. I. A Simple Synthesis and Some Synthetic Applications of Substituted Phosphide and Phosphinite Anions. *Synthesis.* **1986**, 198−208.

(10) Xu, B.; Ji, D.; Wu, L.; Zhou, L.; Liu, Y.; Zhang, Z.-M.; Zhang, J. Palladium/Xu-Phos-catalyzed enantioselective cascade Heck/remote C(sp^2) –H alkylation

reaction. *Chem.* **2022**, *8*, 836−849.

(11) Ishu, K.; Kumar. D.; Maurya, N. K.; Yadav, S.; Chaudhary, D.; Kuram, M. R. Dicarbofunctionalization of Unactivated Alkenes by Palladium-Catalyzed Domino Heck/Intermolecular Direct Hetero Arylation with Heteroarenes. *Org. Biomol. Chem.* **2021**,*19*, 2243−2253.
